# Oil palm phenolics attenuate changes caused by an atherogenic diet in mice

**DOI:** 10.1007/s00394-012-0346-0

**Published:** 2012-04-11

**Authors:** Soon-Sen Leow, Shamala Devi Sekaran, Kalyana Sundram, YewAi Tan, Ravigadevi Sambanthamurthi

**Affiliations:** 1 Malaysian Palm Oil Board, No. 6, Persiaran Institusi, Bandar Baru Bangi, 43000 Kajang, Selangor, Malaysia; 2 University of Malaya, 50603 Kuala Lumpur, Malaysia; 3 Malaysian Palm Oil Council, 2nd Floor, Wisma Sawit, Lot 6, SS6, Jalan Perbandaran, 47301 Kelana Jaya, Selangor, Malaysia

**Keywords:** Oil palm phenolics, Antioxidants, Atherosclerosis, Cardiovascular disease, Microarray, Gene expression

## Abstract

**Background:**

Water-soluble phenolics from the oil palm possess significant biological properties.

**Purpose:**

In this study, we aimed to discover the role of oil palm phenolics (OPP) in influencing the gene expression changes caused by an atherogenic diet in mice.

**Methods:**

We fed mice with either a low-fat normal diet (14.6 % kcal/kcal fat) with distilled water, or a high-fat atherogenic diet (40.5 % kcal/kcal fat) containing cholesterol. The latter group was given either distilled water or OPP. We harvested major organs such as livers, spleens and hearts for microarray gene expression profiling analysis. We determined how OPP changed the gene expression profiles caused by the atherogenic diet. In addition to gene expression studies, we carried out physiological observations, blood hematology as well as clinical biochemistry, cytokine profiling and antioxidant assays on their blood sera.

**Results:**

Using Illumina microarrays, we found that the atherogenic diet caused oxidative stress, inflammation and increased turnover of metabolites and cells in the liver, spleen and heart. In contrast, OPP showed signs of attenuating these effects. The extract increased unfolded protein response in the liver, attenuated antigen presentation and processing in the spleen and up-regulated antioxidant genes in the heart. Real-time quantitative reverse transcription-polymerase chain reaction validated the microarray gene expression fold changes observed. Serum cytokine profiling showed that OPP attenuated inflammation by modulating the Th1/Th2 axis toward the latter. OPP also increased serum antioxidant activity to normal levels.

**Conclusion:**

This study suggests that OPP may possibly attenuate atherosclerosis and other forms of cardiovascular disease.

**Electronic supplementary material:**

The online version of this article (doi:10.1007/s00394-012-0346-0) contains supplementary material, which is available to authorized users.

## Introduction

Cardiovascular disease, together with atherosclerosis, is the main cause of death in the world [1]. The oxidation of low-density lipoproteins (LDL) has been accepted as an important initial event in the development of atherosclerosis [2]. Diets with plant-based foods such as fruit, vegetables, nuts and whole grains have been associated with a significantly lower risk of cardiovascular disease [3, 4], either through indirect effects due to the replacement of high energy density foods with these typical low energy density materials or through direct effects due to the plant components present in these foods. Cardioprotection afforded by these plant compounds is in part attributed to their high antioxidant activities [5, 6], although actions other than their direct antioxidant activities may be more important [7, 8], such as their anti-inflammatory effects [9]. For example, phenolic compounds from soy [10], pomegranate [11, 12], ginger [13, 14], red wine [15, 16] and olive [17] were found to attenuate atherosclerosis either by LDL-dependent mechanisms such as reducing LDL levels, inhibiting LDL oxidation and increasing antioxidant status or via other LDL-independent mechanisms such as modulating vascular function, blood pressure, enzyme systems and signal transduction. Plant phenolics are thus promising candidates for the prevention of cardiovascular disease [18].

The oil palm (*Elaeis guineensis*) fruit contains various lipid-soluble phytochemicals such as carotenoids, tocopherols and tocotrienols, which possess significant antioxidant properties [19–21]. During palm oil milling, large volumes of aqueous vegetation liquor are produced. The extraction of water-soluble materials from the oil palm vegetation liquor recovers another class of phytochemicals from the oil palm [22], namely oil palm phenolics (OPP). OPP consist mainly of phenolic acids, including three caffeoylshikimic acid isomers, protocatechuic acid and *p*-hydroxybenzoic acid [23]. OPP have been shown to display antioxidant properties and confer positive outcomes on degenerative diseases in various animal models without evidence of toxicity [24–26]. OPP showed significant biological activities against copper-mediated LDL oxidation in vitro, promoted nitric oxide-mediated vascular relaxation ex vivo, reduced blood pressure in a nitric oxide-deficient rat model, prevented cardiac arrhythmia in coronary arterial ligation rat models, as well as reduced atherosclerotic plaques in blood vessels of atherogenic diet-fed rabbits in vivo [24, 25]. These bioactivities confer cardioprotection. In addition, we previously reported gene expression changes caused by OPP in mice fed a low-fat normal diet, in which the extract was indicated to have novel health-promoting properties including possible hepatoprotective, anti-dyslipidemic, anti-thrombotic and caloric restriction mimetic effects [27].

In this study, we hypothesized that OPP can attenuate atherosclerosis by modulating gene expression changes caused by an atherogenic diet. We thus tested this hypothesis by first feeding mice with either a low-fat normal diet (14.6 % kcal/kcal fat) with distilled water (ND + DW group) or a high-fat atherogenic diet (40.5 % kcal/kcal fat) containing cholesterol (0.15 % w/w). The latter group was given either distilled water (AD + DW group) or OPP (AD + OPP group). We harvested major organs such as livers, spleens and hearts for microarray gene expression profiling analysis. We then determined how OPP changed the gene expression profiles caused by the atherogenic diet.

## Materials and methods

### OPP samples

OPP samples were prepared according to the methods described in Sambanthamurthi et al. [22]. OPP contain numerous phenolic acids. Three isomers of caffeoylshikimic acid are major components of the extract [23]. Other phenolic acids include caffeic acid, protocatechuic acid and *p*-hydroxybenzoic acid. The detailed composition of OPP is as described earlier [24].

### Animal feeding and sample collection

All male inbred BALB/c mice that were designated for this study were purchased from the Institute of Medical Research, Kuala Lumpur, Malaysia, at around 5 weeks of age just after weaning. All animal procedures were approved by the Animal Care and Use Committee of the University of Malaya, Kuala Lumpur, Malaysia. The animals were randomly assigned into cages (*n* = 5 per cage) and acclimatized for 1 week, during which a standard chow diet purchased from the University of Malaya and distilled water were given ad libitum. At the start of the experiment, the diet of the animals was changed to a custom-made low-fat normal diet (58.2 % kcal/kcal carbohydrate, 27.2 % kcal/kcal protein and 14.6 % kcal/kcal fat, including cellulose, mineral mix, vitamin mix and DL-methionine) or a custom-made high-fat atherogenic diet (40.5 % kcal/kcal carbohydrate, 19.0 % kcal/kcal protein and 40.5 % kcal/kcal fat, including 0.15 % w/w cholesterol, cellulose, mineral mix, vitamin mix and DL-methionine) for 6 weeks ad libitum. The ND + DW group (*n* = 10) and the AD + DW group (*n* = 10) were supplemented with distilled water, while the AD + OPP group (*n* = 10) was supplemented with OPP, as drinking fluid ad libitum. The phenolic content of the OPP given was around 1,500 ppm gallic acid equivalent. Food and fluid were changed daily. During the animal feeding process, body weights were monitored every week, while fluid intake was monitored every day for 6 weeks. Food intake and fecal output were monitored for seven consecutive days in the middle of the designated feeding period, between week two to week three. The mice were killed via euthanasia with diethyl ether followed by exsanguination after 6 weeks of feeding following an overnight food fast (with fluids still provided). Blood samples were collected via cardiac puncture. Three major organs including livers, spleens and hearts were excised, blotted, weighed, snap-frozen in liquid nitrogen and stored at −80 °C until the total RNA extraction process.

### Hematology and clinical biochemistry analyses

Hematology and clinical biochemistry analyses were carried out by the Clinical Biochemistry and Hematology Laboratory, Department of Veterinary Pathology and Microbiology, Faculty of Veterinary Medicine, Universiti Putra Malaysia, Serdang, Selangor, Malaysia, using the Animal Blood Counter Vet Hematology Analyzer (Horiba ABX, France) and the Roche/Hitachi 902 Chemistry Analyzer (Roche/Hitachi, Switzerland), respectively. Aliquots (200 μL) of whole blood samples (*n* = 4) were stored in tubes containing ethylenediaminetetraacetic acid for hematology analysis. In order to obtain sera, blood samples were allowed to clot at room temperature for 2 h before being centrifuged at 1,000×*g* for 5 min, after which the supernatant layers were collected and stored at −20 °C. A portion (100 μL) of each serum sample (*n* = 6 per group) was kept in aliquots for cytokine profiling and antioxidant analysis. The remaining serum samples (around 200 μL per replicate) were subjected to clinical biochemistry analysis for eleven parameters. Two samples in the ND + DW group, two samples in the AD + DW group and three samples in the AD + OPP group were excluded due to blood lysis.

### Total RNA extraction

Total RNA isolation from mouse organs was carried out using the RNeasy Mini Kit (Qiagen, Inc., Valencia, CA) and QIAshredder homogenizers (Qiagen, Inc., Valencia, CA), preceded by grinding in liquid nitrogen using mortars and pestles. The total RNA samples obtained were subjected to NanoDrop 1000A Spectrophotometer (Thermo Fisher Scientific, Waltham, MA) measurement for yield and purity assessment. Integrity of the total RNA samples was then assessed using the Agilent 2100 Bioanalyzer (Agilent Technologies, Santa Clara, CA) and Agilent RNA 6000 Nano Chip Assay Kit (Agilent Technologies, Santa Clara, CA). Four total RNA samples with the highest RNA Integrity Numbers and 28S/18S rRNA ratios within each condition were then selected for microarray studies.

### Microarray gene expression analysis

Amplification of total RNA samples that were of high yield, purity and integrity was carried out using the Illumina TotalPrep RNA Amplification Kit (Ambion, Inc., Austin, TX). The biotinylated cRNA produced was then hybridized to the Illumina MouseRef-8 Version 1 Expression BeadChip (Illumina, Inc., San Diego, CA), using the Direct Hybridization Kit (Illumina, Inc., San Diego, CA). Microarray hybridization, washing and scanning were carried out according to the manufacturer’s instructions. The raw gene expression data obtained are available at Gene Expression Omnibus [28] (Accession number: GSE30908).

Quality control of the hybridization, microarray data extraction and initial analysis were carried out using the Illumina BeadStudio software (Illumina, Inc., San Diego, CA). Outlier samples were removed via hierarchical clustering analysis provided by the Illumina BeadStudio software and also using the TIGR MeV software (The Institute for Genomic Research, Rockville, MD) [29]. A minimum of three replicates per condition (with outliers removed) was then considered for further analysis. For livers, spleens and hearts of mice in the ND + DW group, four replicates were analyzed for each organ, respectively. On the other hand, for livers, spleens and hearts of mice in the AD + DW and AD + OPP groups, three replicates were analyzed for each organ, respectively, with the exception of livers in the AD + DW group, in which four replicates were analyzed. Three comparisons were made in this study, with the first comparison to find out gene expression changes caused by the atherogenic diet (AD + DW:ND + DW), the second comparison to identify gene expression changes caused by OPP (AD + OPP:AD + DW) and the third to identify genes that were differentially regulated by the two factors. The first two comparisons were carried out separately before the third comparison was made.

For the first two comparisons, gene expression values were normalized using the rank invariant method and genes that had Detection Levels of more than 0.99 in either condition (control or treatment) were considered significantly detected. To filter the data for genes that changed significantly in terms of statistics, the Illumina Custom error model was used and genes were considered significantly changed at a |Differential Score| of more than 20, which was equivalent to a *P* value of less than 0.01 [30]. The stringency of this filtering criterion was lowered to a |Differential Score| of more than 13, which was equivalent to a *P* value of less than 0.05, if less than 100 genes were found significantly changed. Since the results of this statistical analysis were to be used for functional analysis, it was relevant to include more genes by using a lower threshold to give statistical power to the functional analysis. The genes and their corresponding data were then exported into the Microsoft Excel software (Microsoft Corporation, Redmond, WA) for further analysis. To calculate fold changes, an arbitrary value of 10 was given to expression values which were less than 10. Fold changes were then calculated by dividing means of Signal Y (treatment) with means of Signal X (control) if the genes were up-regulated and vice versa if the genes were down-regulated. Two-way (gene and sample) hierarchical clustering of the significant genes was then performed using the TIGR MeV software to ensure that the replicates of each condition were clustered to each other. The Euclidean distance metric and average linkage method were used to carry out the hierarchical clustering analysis. Changes in biological pathways and gene ontologies were assessed via functional enrichment analysis, using the GenMAPP [31] and MAPPFinder [32] softwares (University of California at San Francisco, San Francisco, CA). The MAPPFinder software ranks GenMAPPs (pathways) and gene ontologies based on hypergeometric distribution. GenMAPPs and gene ontologies that had Permuted *P* values of less than 0.01, Numbers of Genes Changed of more than or equal to 2 and Z Scores of more than 2 were considered significant. A Permuted *P* value of less than 0.05 was used when genes were selected using a |Differential Score| of more than 13, in order to identify more GenMAPPs and gene ontologies affected. Boxes colored yellow indicate genes that were up-regulated, while those colored blue indicate genes that were down-regulated. The fold changes are indicated next to the boxes. Individual boxes that have different shadings within them indicate the presence of multiple probes (splice transcripts) within a single gene. Changes in regulatory networks were also analyzed through the use of the Ingenuity Pathways Analysis software (Ingenuity^®^ Systems, Redwood City, CA). A network is a graphical representation of the molecular relationships between genes or gene products. Genes or gene products were represented as nodes, and the biological relationship between two nodes was represented as an edge (line). The intensity of the node color indicates the degree of up-regulation (red) or down-regulation (green). Nodes were displayed using various shapes that represented the functional class of the gene product. Edges were displayed with various labels that described the nature of the relationship between the nodes.

In order to assess how OPP affected genes changed by the atherogenic diet, the significantly changed genes obtained in the first comparison were intersected with significantly changed genes obtained in the second comparison in order to obtain a set of genes that were significantly regulated by both factors (atherogenic diet and OPP), hence the third comparison. The directions of fold changes for genes in this intersecting set were then compared in order to identify the number of genes that were differentially regulated by both factors in terms of direction.

### Real-time qRT-PCR validation

Two-step real-time quantitative reverse transcription-polymerase chain reaction (qRT-PCR) was carried out on six target genes from the second comparison (Online Resource 1 in Supplementary Material 1), selected to represent the different organs used in the microarray experiments, using TaqMan Gene Expression Assays (Applied Biosystems, Foster City, CA). The same aliquots of total RNA samples used in the microarray experiments were utilized for this analysis. Primer and probe sets for the selected genes were obtained from the ABI Inventoried Assays-On-Demand (Applied Biosystems, Foster City, CA).

Briefly, reverse transcription to generate first-strand cDNA from total RNA was carried out using the High-Capacity cDNA Reverse Transcription Kit (Applied Biosystems, Foster City, CA). Real-time PCR was then carried out on the first-strand cDNA generated using a 25 μL reaction volume in an Applied Biosystems 7000 Real-Time PCR System (Applied Biosystems, Foster City, CA) with the following conditions: 50 °C, 2 min, 1 cycle; 95 °C, 10 min, 1 cycle; 95 °C, 15 s and 60 °C, 1 min, 40 cycles. For gene expression measurements, reactions for each biological replicate and non-template control (NTC) were carried out in duplicates. For amplification efficiency determination, reactions were carried out in triplicates.

Quality control of the replicates used, real-time qRT-PCR data extraction and initial analysis were carried out using the 7000 Sequence Detection System software (Applied Biosystems, Foster City, CA). A manual threshold of 0.6000 and an auto baseline were applied in order to obtain the threshold cycle (Ct) for each measurement taken. The threshold was chosen as it intersected the exponential phase of the amplification plots [33]. The criteria for quality control of the data obtained include ∆Ct less than 0.5 between technical replicates and ∆Ct more than 5.0 between samples and NTCs [34].

Relative quantification of the target genes of interest was carried out using the qBase 1.3.5 software (Center for Medical Genetics, Ghent University Hospital, Ghent, Belgium) [35], which takes into account the calculations of amplification efficiencies and multiple housekeeping genes. Expression levels of target genes were normalized to the geometric mean of three housekeeping genes, *Sfrs9*, *Guk1* and *Hnrpab*. Stability of these housekeeping genes was assessed using the geNorm 3.5 software (Center for Medical Genetics, Ghent University Hospital, Ghent, Belgium) [36].

### Cytokine profiling

Cytokine profiling on serum samples was performed using the Bio-Plex Suspension Array System (Bio-Rad Laboratories, Hercules, CA), available at the Faculty of Medicine, University of Malaya. This assay was carried out using the Bio-Plex Mouse Cytokine 23-Plex Cytokine Panel (Bio-Rad Laboratories, Hercules, CA), which contains antibody-conjugated beads (25× concentration) for 23 types of mouse cytokines including IL-1α, IL-1β, IL-2, IL-3, IL-4, IL-5, IL-6, IL-9, IL-10, IL-12 (p40), IL-12 (p70), IL-13, IL-17, eotaxin, G-CSF (granulocyte colony-stimulating factor), GM-CSF (granulocyte–macrophage colony-stimulating factor), IFN-γ (interferon-γ), KC (keratinocyte-derived chemokine), MCP-1 (monocyte chemoattractant protein-1 or also known as monocyte chemotactic and activating factor MCAF), MIP-1α (macrophage inflammatory protein-1α), MIP-1β, RANTES (regulated on activation, normal T cell expressed and secreted) and TNF-α (tumor necrosis factor-α). The kit also contains the necessary components such as detection antibodies (25× concentration) and standards. The experiment was carried out according to the manufacturer’s instructions. Each serum sample (*n* = 6) was tested in duplicates. The data were analyzed using the Bio-Plex Manager Version 4.0 software (Bio-Rad Laboratories, Hercules, CA). Generation of standard curves, averaging of duplicate fluorescence readings of each serum sample, background subtraction with the blank and calculation of concentration for each cytokine were carried out by the Bio-Plex Manager software. The averaged concentration readings were exported into the Microsoft Excel software (Microsoft Corporation, Redmond, WA) for statistical analysis.

### Antioxidant analysis

Serum antioxidant analysis was carried out using four assays, including the total phenolics content by Folin-Ciocalteu reagent (TP-FCR) assay [37, 38], the ferric reducing ability of plasma (FRAP) assay [39], the 2,2-diphenyl-1-picrylhydrazyl (DPPH) scavenging activity assay [40, 41] and the Trolox equivalent antioxidant capacity (TEAC) assay [42]. All these assays were carried out using the Infinite M200 microplate reader (Tecan, Austria). Each serum sample (*n* = 6) was tested in duplicates. Measurement settings and data acquisition were carried out using the Magellan Version 6.2 software (Tecan, Austria). Generation of standard curves, averaging of duplicate absorbance readings of each sample, background subtraction with the blank, calculation of concentration and statistical analysis for each assay were carried out in the Microsoft Excel software (Microsoft Corporation, Redmond, WA).

### Statistical analysis

Statistical analysis was carried out by using the two-tailed unpaired Student’s *t* test available in the Microsoft Excel software (Microsoft Corporation, Redmond, WA) unless otherwise stated. Differences with *P* values of less than 0.05 were considered statistically significant.

## Results

### Physiology and pathology studies on mice

The body weights of mice steadily increased every week throughout the 6 weeks of feeding, with animals in both the AD + DW and the AD + OPP groups showing a higher increase in weight gain compared to those in the ND + DW group (Online Resource 2a in Supplementary Material 1). When the organ weights from these animals were compared, mice in both the AD + DW and the AD + OPP groups had increased liver weights compared to those in the ND + DW group (Online Resource 2b in Supplementary Material 1). No significant differences in terms of body weights and organ weights were found when comparison was made only between the AD + DW and AD + OPP groups. Also, no differences in terms of fluid intake were found between the three groups (Online Resources 2c and 2d in Supplementary Material 1). In the AD + OPP group, the volume of OPP taken was around 1.75 mL/day. Based on an average mouse weight of 25 g, this would be equivalent to the consumption of about 350 mL (in volume) or 500 mg (in gallic acid equivalent weight) of OPP by a 60 kg human, calculated based on the body surface area normalization method [43]. Mice in both the AD + DW and AD + OPP groups ingested less food (Online Resources 2e and 2f in Supplementary Material 1) and produced less feces (Online Resources 2g and 2h in Supplementary Material 1) compared to the ND + DW group. No significant changes in terms of food intake and fecal output were found when comparison was made only between the AD + DW and AD + OPP groups, thus indicating that the effects caused by OPP in mice fed the atherogenic diet were due to the components of the extract and not due to alterations in food intake.

Hematology analysis revealed that mice in both the AD + DW and AD + OPP groups had a significant increase in the levels of white blood cells, neutrophils and lymphocytes compared to those in the ND + DW group (Online Resource 3 in Supplementary Material 1), indicating an inflammatory response. OPP did not affect hematology parameters in mice given the atherogenic diet. Mice in both the AD + DW and AD + OPP groups showed significant changes in terms of serum clinical biochemistry, including albumin (↓), globulin (↑), ratio of albumin to globulin (↓), total cholesterol (↑), LDL (↑) and HDL (↑) levels (Online Resource 3 in Supplementary Material 1), when compared to those in the ND + DW group. OPP also did not cause significant changes in these clinical biochemistry parameters measured, except for normalizing the glucose level in mice on the atherogenic diet similar to that of mice on the normal diet, probably due to the presence of fruit sugars.

### Microarray gene expression profiling of livers, spleens and hearts

For gene expression profiling, Illumina microarrays were used to study the effects of the atherogenic diet and OPP (when mice were on the atherogenic diet) in three major mouse organs, including the liver, spleen and heart. Genes considered significantly changed were further subjected to two-way (gene and sample) hierarchical clustering using the TIGR MeV software to ensure that the replicates of each condition were clustered to each other. Online Resource 4 in Supplementary Material 1 shows an example of the two-way hierarchical clustering analysis carried out on genes significantly changed by OPP in the liver.

Using a |Differential Score| of more than 20, which was equivalent to a *P* value of less than 0.01, the number of genes significantly changed by the atherogenic diet was highest in the liver (2,593 up-regulated and 451 down-regulated), followed by the spleen (990 up-regulated and 534 down-regulated) and the heart (1,441 up-regulated and 991 down-regulated). The number of genes significantly changed by OPP was highest in the spleen (327 up-regulated and 249 down-regulated), followed by the liver (35 up-regulated and 84 down-regulated) and the heart (19 up-regulated and 13 down-regulated). In the second comparison, as the heart showed the least number of genes significantly changed (32 genes) and thus would not give much information in further functional enrichment analysis, we reduced the stringency by filtering for significantly changed genes with a |Differential Score| of more than 13, which was equivalent to a *P* value of less than 0.05. This yielded 132 significantly changed genes in the heart (79 up-regulated and 53 down-regulated). The lists of genes, GenMAPPSs and gene ontologies significantly changed by the atherogenic diet in these mouse organs are provided in Supplementary Material 2, while those significantly changed by OPP are given in Supplementary Material 3.

The atherogenic diet increased the turnover of metabolites in the liver, as shown by an up-regulation of genes involved in the generation of precursor metabolites (anabolism) and energy (catabolism). It was also evident that genes involved in fatty acid beta oxidation, the tricarboxylic acid cycle and the electron transport chain were up-regulated, thus suggesting an increase in energy production due to the utilization of extra fat. In addition, the turnover of liver tissues was evident, from the up-regulation of nuclear receptors that stimulate hepatocyte growth such as *Hnf4a* (FC 2.57) (Online Resource 5a in Supplementary Material 1) and cytochrome c oxidases, complement genes and caspases involved in cell death (Online Resource 5b in Supplementary Material 1). Not unexpectedly, genes involved in cholesterol biosynthesis were down-regulated (Online Resource 5c in Supplementary Material 1). Genes involved in the immune response were also up-regulated by the atherogenic diet in the spleen, such as those regulated by tumor necrosis factor-alpha (*Tnfα*) (although *Tnfa* itself was not regulated) and signal transducer and activator of transcription 3 (*Stat3*) (FC 1.59) (Online Resource 6a in Supplementary Material 1). In addition, the apoptotic process was up-regulated. On the other hand, genes down-regulated by the atherogenic diet include those regulated by the tumor suppressor *Tp53* (FC −1.53) (Online Resource 6b in Supplementary Material 1) and transforming growth factor-beta (*Tgfb1*) (FC −2.33). *Tp53* is anti-proliferative, while *Tgfb1* is anti-inflammatory. The up-regulation of *Tnfa* and *Stat3*, coupled with the down-regulation of *Tp53* and *Tgfb1*, suggests the up-regulation of an inflammatory response toward the atherogenic diet in the spleen. In the heart, the atherogenic diet increased the expression of genes involved in fatty acid beta oxidation, proteasomal degradation, heme biosynthesis, as well as inflammation, including those regulated by *Tnfα* (although *Tnfa* itself was not regulated), cyclic adenosine monophosphate response element-binding protein (*Crebbp*) (FC 7.15) and *Jun* oncogene (FC 1.67), which is part of activator protein-1 (*Ap*-*1*) (Online Resource 7a in Supplementary Material 1). Down-regulated genes were found to be involved in glycolysis, circadian rhythm, muscle development and anti-inflammatory networks, such as those regulated by sirtuin 1 (*Sirt1*) (FC −2.03) and *Tgfb1* (FC −6.65) (Online Resource 7b in Supplementary Material 1).

**Fig. 1:**
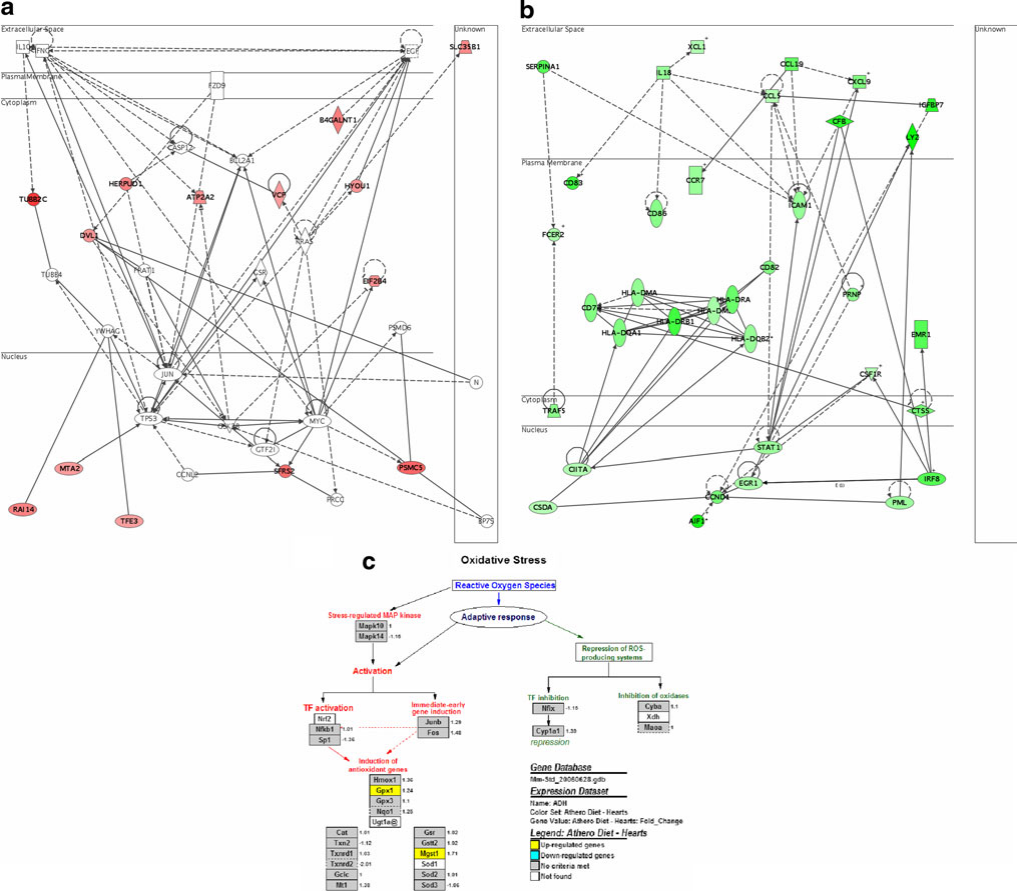
Genes regulated by OPP in the liver, spleen and heart. **a** Genes up-regulated in the liver unfolded protein response network. Genes involved in the unfolded protein response up-regulated by OPP include *Tra1* and *Vcp*. **b** Genes down-regulated by OPP in the spleen antigen presentation network. Genes encoding MHC II molecules such as those belonging to the HLA-D family, genes of antigenic markers such as *Cd74*, *Cd82*, *Cd83* and *Cd86* as well as genes encoding chemokines such as *Ccl5*, *Ccl19*, *Cxcl9* and *Xcl1* were down-regulated by OPP. The down-regulation of genes encoding MHC II molecules could be caused by the down-regulation of *C2ta*. **c** Genes up-regulated by OPP in the heart antioxidant pathway. Genes involved in antioxidant activity such as *Gpx1* and *Mgst1* were up-regulated by OPP

In livers of mice belonging to the AD + OPP group, genes involved in the unfolded protein response were up-regulated (Fig. 1a) compared to the AD + DW group. Down-regulation of genes involved in endogenous antigen presentation, fatty acid metabolism, arylsulfatase activity, reduced nicotinamide adenine dinucleotide (NADH) dehydrogenase (ubiquinone) activity and oxidoreductase activity was also observed, indicating a down-regulation of the inflammatory response and energy production. Compared to the AD + DW group, genes up-regulated in spleens of mice in the AD + OPP group were those involved in carbohydrate metabolism, glucose metabolism, glutathione metabolism as well as cytoskeleton organization and biogenesis. Genes down-regulated by OPP in spleens of mice are involved in antigen presentation (Fig. 1b), apoptosis, B cell receptor signaling, defence response, genes specific to blood and lymph tissues, heme biosynthesis, immune response, regulation of apoptosis, T-cell activation and differentiation as well as T-cell receptor signaling. In hearts of mice, genes up-regulated by OPP include those involved in antioxidant activities (Fig. 1c), circadian exercise and nucleosome assembly. Down-regulated genes, on the other hand, are involved in electron transport and signaling as well as cell proliferation and migration.

In order to assess how OPP affected genes changed by the atherogenic diet, the significantly changed genes obtained in the first comparison were intersected with significantly changed genes obtained in the second comparison to obtain a set of genes, which was significantly regulated by both factors (atherogenic diet and OPP). This comparison is given in Supplementary Material 4. The percentages of genes that were differentially regulated by both factors in terms of direction were then calculated, with the results for the liver, spleen and heart being 89.87 (71 out of 79 genes), 46.21 (67 out of 145 genes) and 58.46 % (38 out of 65 genes), respectively. A majority (>50 %) of the genes regulated by OPP in the different organs thus showed a difference in regulation direction when compared to the atherogenic diet. Also, the highest percentage of change was found in the liver, while the lowest percentage of change was found in the spleen. Online Resource 8 in Supplementary Material 1 shows a diagram to compare the fold change direction of genes significantly changed by the atherogenic diet and OPP, using the liver as an example.

### Real-time qRT-PCR validation

To confirm the microarray results, the expression levels of six target genes were measured using real-time quantitative reverse transcription-polymerase chain reaction (qRT-PCR). As the focus of this study was more to identify the changes caused by OPP rather than those caused by the atherogenic diet, genes chosen for real-time qRT-PCR were from the second comparison (AD + OPP:AD + DW). The direction and magnitude of fold changes obtained from the real-time qRT-PCR technique quantified by the qBase software [35] were comparable to those obtained from the microarray technique (Online Resource 9a in Supplementary Material 1). Correlation of fold changes obtained by the two gene expression profiling techniques was high (*R* ^2^ = 0.9920) (Online Resource 9b in Supplementary Material 1).

### Cytokine profiling and antioxidant analysis of blood serum samples

**Fig. 2:**
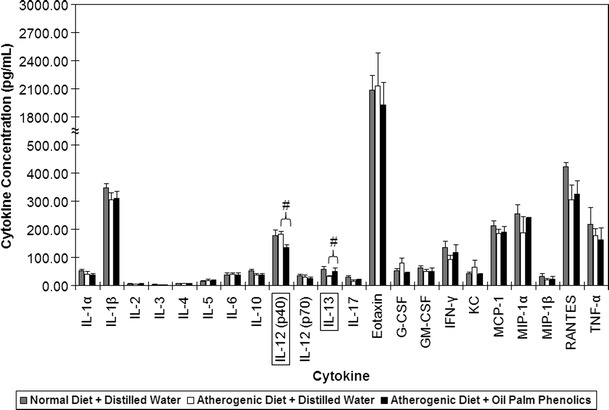
Results of cytokine profiling on blood serum samples from mice. ^#^ *P* < 0.05; *n* = 6. Values are means ± SEM

**Fig. 3:**
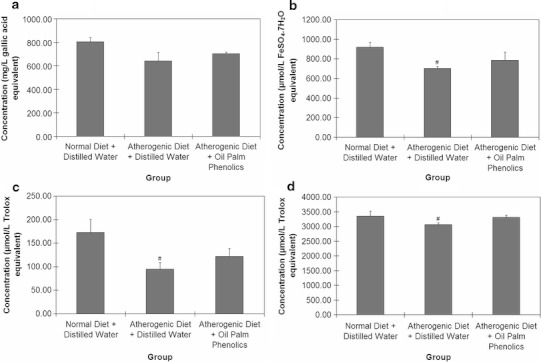
Results of antioxidant analysis on blood serum samples from mice. **a** TP-FCR. **b** FRAP. **c** DPPH. **d** TEAC. ^#^ *P* < 0.05 versus Normal Diet + Distilled Water; *n* = 6. Values are means ± SEM

For serum cytokine profiling, the levels of eotaxin were surprisingly high for all the animals (Fig. 2). This may be caused by the exposure of the animals to the non-sterile environment as they were not maintained in a specific pathogen-free facility. Although IL-9 was also present in the multiplex cytokine panel used, it was not detected in any of the blood serum samples tested. For those on the atherogenic diet, there was a significant decrease in IL-12 (p40) and a significant increase in IL-13 in the AD + OPP group when compared to the AD + DW group (Fig. 2). The antioxidant analysis carried out on the serum samples showed that for the AD + DW group, there was a significant decrease in antioxidant capacity compared to the ND + DW group, which indicates a higher oxidative stress (Fig. 3). The AD + OPP group, on the other hand, showed almost similar antioxidant capacity when compared to the ND + DW group, thus indicating that the antioxidant resistance of mice supplemented with OPP was still high, although they were also given the atherogenic diet.

## Discussion

### Effects of atherogenic diet

When livers of mice in the AD + DW group were compared to those in the ND + DW group, the fatty acid beta oxidation process was found to be up-regulated, presumably to metabolize extra fatty acids obtained from the atherogenic diet. When challenged with the atherogenic diet, the liver thus adjusts its metabolic processes in relation to lipid metabolism and energy production [44]. Nuclear receptors involved in tissue growth and genes involved in cell death were also up-regulated in the present study, thus suggesting that the atherogenic diet triggered hepatic inflammatory reprogramming and liver regeneration in the mice. This also explains the enlargement of livers that was observed in these animals. An example of a nuclear receptor up-regulated by the atherogenic diet in the present study is the hepatocyte nuclear factor 4-alpha (*Hnf4a*) (FC 2.57), which was also found to be up-regulated when ApoE3Leiden (E3L) mice (which have lipid profiles resembling those of humans) were fed an atherogenic diet [45]. Among the genes involved in cell death that were up-regulated in the present study were those encoding cytochrome c oxidases belonging to the mitochondrial electron transport chain, complement genes and caspases. The up-regulation of these genes suggests that cell death occurred via apoptosis as a result of complement-mediated cell damage. Interestingly, Recinos et al. [46] also showed that induction of the complement pathway in the liver was associated with lesion development in atherosclerosis-prone LDL receptor-deficient (LDLr^−/−^) mice when they were fed a high-fat Western style diet. As the atherogenic diet provided dietary cholesterol that further increased cholesterol levels in the blood circulation, genes involved in hepatic cholesterol biosynthesis were down-regulated in this study. This observation was not unexpected as de novo cholesterol biosynthesis is down-regulated when cholesterol is available from dietary intake [44, 47].

The immune system has long been implicated in atherosclerosis [48–50], due to the presence of inflammation. In response to the atherogenic diet, the spleen showed an up-regulation in the production and turnover of immune cells in the present study. A network significantly up-regulated involved the *Stat3* gene (FC 1.59). It is interesting to note that the *Stat3* gene was discovered because of its role in the acute phase response and that this is the only capacity in which its function in vivo can be clearly ascribed to its activity as a transcription factor [51]. Although apoptosis was up-regulated in this study, the tumor suppressor *Tp53* (FC −1.53) and other genes linked to it were down-regulated. Together with the up-regulation of the *Stat3* network and apoptosis, the down-regulation of the tumor suppressor *Tp53* implies that the atherogenic diet caused an increased turnover of immune cells in the spleen. This thus explains the increased production and deployment of immune cells in the blood circulation, which may further exacerbate the inflammatory effects of the atherogenic diet.

This study also revealed that two important networks were regulated by the atherogenic diet in the heart, with the first involving an up-regulated *Jun* oncogene (FC 1.67) and the second involving a down-regulated *Tgfb1* (FC −6.65). The JUN protein forms part of the transcription factor activator protein-1, which is pro-inflammatory as it has been implicated in oxidative stress [52]. Binding sites of the redox-regulated transcription factor activator protein-1 are located in the promoter region of a large variety of genes that are directly involved in the pathogenesis of diseases, including atherosclerosis. Activation of *Jun* via Jun amino-terminal kinase (*Jnk*) in response to various forms of stress causes arterial injury [53] and heart disease [54–59]. The down-regulation of the *Tgfb1* gene by the atherogenic diet in the present study also implies a pro-inflammatory response toward the diet in the heart. This is because *Tgfb* is anti-inflammatory in atherosclerosis [60], as it plays an important role in the maintenance of normal blood vessel structure, and defects in this gene have been linked to a range of cardiovascular syndromes including loss of healthy vessel architecture and aneurysm [61]. Microarray profiling carried out by Tabibiazar et al. [62] on the aortas of apolipoprotein E-deficient (apoE^−/−^) mice on a high-fat diet compared with control C57BL/6J and C3H mice across time also showed a decreased expression of an isoform of *Tgfb*.

In the present study, preliminary physiology studies carried out during animal feeding in order to identify the effects of the atherogenic diet on the well-being of mice showed several adverse effects of the diet, which include increases in inflammation and oxidative stress, similar to the observations found in previous studies [63–65].

### Effects of OPP

In the livers of mice belonging to the AD + OPP group, genes up-regulated when compared to those of mice belonging to the AD + DW group were found to be involved in the unfolded protein response. These genes include *Herpud1* (homocysteine-inducible, endoplasmic reticulum stress-inducible, ubiquitin-like domain member 1) (FC 1.51), *Tra1* (tumor rejection antigen gp96) (FC 1.35) and *Vcp* (valosin containing protein) (FC 1.23). The unfolded protein response can be promoted by the buildup of unfolded proteins in the endoplasmic reticulum, and it constitutes a mechanism to reduce this burden. The unfolded protein response acutely reduces translation of new proteins, followed by increased expression of chaperones to aid folding of existing proteins and enhanced elimination of proteins that cannot be refolded [66]. Endoplasmic reticulum stress responsive genes have been suggested to be a protective response to protein unfolding or protein damage resulting from cellular stress signals. In addition, accumulation of oxidatively modified proteins can elicit cellular damage and this is curtailed under normal conditions by intracellular protein degradation systems such as the ubiquitin–proteasome system [67]. Thus, OPP may help to reduce the amount of damaged proteins caused by the atherogenic diet in the liver.

Transketolase (*Tkt*), which controls the non-oxidative branch of the pentose phosphate pathway, provides reduced nicotinamide adenine dinucleotide phosphate (NADPH) for biosynthesis and reducing power of several antioxidant systems [68]. It was up-regulated in the spleens of mice by OPP (FC 1.85), together with glucose-6-phosphate dehydrogenase (X-linked) (*G6pdx*) (FC 3.59) and phosphogluconate dehydrogenase (*Pgd*) (FC 2.82), all of which are involved in the pentose phosphate pathway. The products of the pentose phosphate pathway are important for the biosynthesis of purine and for stimulating antioxidant response pathways in conjunction with the action of dietary phenolic antioxidants. This may also explain the up-regulation of antioxidant genes including *Mgst1* (microsomal glutathione S-transferase 1) (FC 1.79), *Mgst2* (microsomal glutathione S-transferase 2) (FC 3.08), *Gsr* (glutathione reductase 1) (FC 2.49) and *Gstm1* (glutathione S-transferase, mu 1) (FC 1.89) in the spleens of mice given OPP. Genes encoding MHC molecules such as *H2*-*Ab1* (FC −2.32) and *H2*-*Eb1* (FC −2.36), which have been implicated in atherosclerosis [62], were down-regulated in the spleens of mice, thus suggesting that OPP was able to attenuate the inflammatory response brought about by the atherogenic diet. Activated macrophages and smooth muscle cells express class II histocompatibility antigens such as HLA-DR that allow them to present antigens to T cells, which cause atherosclerosis [69]. The gene expression of MHC II molecules is transcriptionally regulated by the class II transcriptional activator (CIITA or *C2ta*) (FC −2.58). CIITA activates the expression of MHC II in all types of professional antigen-presenting cells (macrophages, dendritic cells and B lymphocytes), of which dendritic cells are the most potent among the three [70]. In line with the down-regulation of MHCs, the *C2ta* gene was down-regulated by OPP in mice fed the atherogenic diet in the present study. A mechanism of anti-inflammation brought about by antioxidants is through the modulation of cytokine induction during inflammation [71]. In agreement with this, cytokines and cytokine receptors such as *Ccl5*, *Ccl19* and *Ccr7* were down-regulated by OPP in the present study (FC −3.25, −2.89 and −2.28, respectively). The CCR7 receptor present on the surface of secondary lymphoid cells for instance functions to attract dendritic cells, which migrate to secondary lymphoid organs to present antigens for the activation of naive T cells. Hence, the down-regulation of cytokines and cytokine receptors by OPP in the present study suggests anti-inflammatory effects of the extract. Additionally, cluster of differentiation (CD) antigenic markers such as *Cd3d*, *Cd24a*, *Cd59b*, *Cd72*, *Cd79a*, *Cd79b*, *Cd83* and *Cd86* were also down-regulated by OPP (FC −1.55, −1.37, −3.02, −2.17, −1.97, −2.28, −3.14 and −2.00, respectively). *Cd83* and *Cd86* are specific markers of mature dendritic cells, which are up-regulated by oxidative stress through a nuclear factor kappa-B-dependent mechanism [70]. The down-regulation of MHC II genes and genes encoding antigenic markers in this study further suggests that OPP suppressed the inflammatory response associated with the atherogenic diet, and this may constitute a mechanism by which OPP ameliorates atherosclerosis.

In the hearts of mice belonging to the AD + OPP group, genes up-regulated when compared to those of mice belonging to the AD + DW group include antioxidant genes, such as *Mgst1* (microsomal glutathione *S*-transferase 1) (FC 1.71) and *Gpx1* (glutathione peroxidase 1) (FC 1.24). These antioxidant genes are essential in the detoxification of carcinogens and the scavenging of reactive oxygen species [72].

Despite the fact that mice in the AD + OPP group did not show significant changes in terms of body and liver weights as well as the hematology and clinical biochemistry parameters when compared to mice in the AD + DW group, further cytokine profiling and antioxidant analysis on the blood serum samples of these mice supported the in vivo anti-inflammatory and antioxidant effects of the extract. In contrast to the effects of OPP that down-regulated hepatic cholesterol biosynthesis genes in mice fed the normal diet found in our previous study [27], the extract did not down-regulate this group of hepatic genes in mice fed the atherogenic diet in the present study. This makes sense as administration of the atherogenic diet has already down-regulated cholesterol biosynthesis, and thus further down-regulation of the pathway would be futile to prevent atherosclerosis. On the other hand, OPP acted as an anti-inflammatory agent and an antioxidant in mice given the atherogenic diet to prevent oxidative stress and inflammation caused by the diet, and this is considered important in the prevention of atherosclerosis and cardiovascular disease.

As a component of the immune response, cytokines play an important role in mediating the inflammatory response in atherosclerosis. Atherosclerosis is normally associated with cytokines that promote a Type 1 helper T-cell (Th1) cellular immune response rather than a Type 2 helper T-cell (Th2) humoral immune response [73]. The modulation of the Th1/Th2 axis toward the latter may thus be atheroprotective [74]. In mice belonging to the AD + OPP group, a decrease in the pro-inflammatory IL-12 (p40 subunit) cytokine and an increase in the anti-inflammatory IL-13 cytokine in the sera were observed compared to the AD + DW group. This is believed to be an attenuation of the inflammatory response toward atherosclerosis. IL-12 is a cytokine of innate immunity, which is secreted by activated macrophages and dendritic cells, and is a key inducer of cell-mediated immunity as it stimulates the production of IFN-γ, stimulates the differentiation of CD4 + helper T lymphocytes into Th1 cells as well as enhances the cytolytic functions of activated natural killer cells and CD8 + cytolytic T lymphocytes [75]. It has been implicated in atherosclerosis [74, 76, 77] and other inflammatory diseases [78, 79]. IL-13 is a cytokine of adaptive immunity, which is secreted by CD4 + helper T lymphocytes (Th2 cells), and it inhibits macrophages and antagonizes IFN-γ [75]. In the present study, the anti-inflammatory effects observed in the serum samples were consistent with the gene expression changes seen in the spleens of mice given OPP, which indicate attenuation of the inflammatory response.

In addition, antioxidant analysis carried out on the mouse blood serum samples showed that OPP restored the antioxidant capacity of animals fed the atherogenic diet. This is in line with the gene expression changes observed in the liver, spleen and heart, in which antioxidant genes were up-regulated by OPP. While the effects observed in the present study are mainly attributed to phenolic compounds, the possible effects of other components in OPP cannot be discounted. What is important here is that the extract in its entirety confers the positive outcomes reported in the present study.

### Limitations of study

We acknowledge that the biggest limitation in this study is the fact that BALB/c mice were used as biological models for atherosclerosis, although rodents that are HDL animals in general are not suitable as they do not mimic the human atherosclerotic disease [80]. Nonetheless, microarray studies in which normal rodent models were used to test for the effects of high-fat or atherogenic diets have been carried out before [62, 81]. It was thus reasoned that OPP might still bring about gene expression changes in major organs of BALB/c mice (which have intermediate susceptibility to atherosclerosis compared to the C57BL/6 mice) on an atherogenic diet. It was also easier to compare the effects of the extract in this study with a previous one involving the normal diet, as animals with the same genetic background were used [27]. Thus, it would be interesting to extend this study to other mouse models of atherosclerosis, such as apoE^−/−^ and LDLr^−/−^ mice in the future.

Another limitation of this study is that fact that the aorta as a primary target of atherosclerosis was not subjected to atheroslerotic lesion and transcriptomic analyses to establish the anti-atherosclerotic mechanisms of OPP. Nevertheless, we have previously shown that OPP reduced atherosclerotic plaques in the aortas of atherogenic diet-fed rabbits [25]. When we first initiated this transcriptomic analysis, however, no commercial whole genome rabbit microarrays were available. Hence, we did not carry out transcriptomic analysis on the aortas of rabbits. We then decided to use whole genome mouse microarrays, as a first step toward identifying the gene expression changes caused by OPP. In relation to this, we previously published a transcriptomic analysis study on the effects of OPP in mice on a normal diet, in which we analyzed three particular organs, liver, spleen and heart [27]. This present study was not a standalone project but a part of this previous study, as we were interested to explore the gene expression changes caused by OPP when the mice were on an atherogenic diet, rather than on a normal diet. During the course of the present study, although we did intend to isolate aortas from the mice for atherosclerotic lesion and transcriptomic analyses, we faced technical difficulties in doing so due to the size limitation of this mouse model. Moreover, the animals that we had were not enough for pooling enough samples to obtain sufficient total RNA. Thus, we decided to carry out transcriptomic analysis on the three organs instead, and identify the gene expression changes that may provide initial clues to help explain how OPP confers protection against the effects of an atherogenic diet. Regardless, transcriptomic analysis on the aortas of any biological model is deemed necessary in the future to provide conclusive insights into the anti-atherosclerotic mechanisms of OPP.

## Electronic supplementary material

Below is the link to the electronic supplementary material.

Supplementary material 1 (PDF 2637 kb)

Supplementary material 2 (PDF 1122 kb)

Supplementary material 3 (PDF 219 kb)

Supplementary material 4 (PDF 84 kb)

## Abbreviations

AD: Atherogenic diet
CD: Cluster of differentiation
CIITA: Class II transcriptional activator
DW: Distilled water
FC: Fold change
HDL: High-density lipoproteins
IL: Interleukin
LDL: Low-density lipoproteins
MHC: Major histocompatibility complex
ND: Normal diet
OPP: Oil palm phenolics
qRT-PCR: Quantitative reverse transcription-polymerase chain reaction
SEM: Standard error of the mean
Th: Helper T
