# Supplementary material for: Oil palm phenolics attenuate changes caused by an atherogenic diet in mice
Source: Eur J Nutr. 2012 Apr 11;52(2):443–56. doi: 10.1007/s00394-012-0346-0 (PMC3573186; doi:10.1007/s00394-012-0346-0)
Supplement: Supplementary file 1 — Supplementary material 1 (PDF 2637 kb) [file 394_2012_346_MOESM1_ESM.pdf]

Article Title: Oil palm phenolics attenuate changes caused by an atherogenic diet in mice.

Journal Name: European Journal of Nutrition.

Author Names: Soon-Sen Leow, Shamala Devi Sekaran, Kalyana Sundram, YewAi Tan, Ravigadevi Sambanthamurthi.

Corresponding Author: Ravigadevi Sambanthamurthi; Malaysian Palm Oil Board, No. 6, Persiaran Institusi, Bandar Baru Bangi, 43000 Kajang, Selangor, Malaysia; E-mail: raviga@mpob.gov.my.

Supplementary Material 1

This file contains the online resources (figures and tables) described in the main manuscript.

Online Resource 1 Genes selected for the real-time qRT-PCR validation experiments

Organ	Symbol	Definition	Accession	Assay ID
Liver	*Herpud1*	*Mus musculus* ubiquitin-like domain member 1	NM_022331	Mm00445600_m1
Liver	*Fads2*	*Mus musculus* fatty acid desaturase 2	NM_019699	Mm00517221_m1
Spleen	*Anxa2*	*Mus musculus* annexin A2	NM_007585	Mm00500307_m1
Spleen	*Cfb*	*Mus musculus* histocompatibility 2, complement component factor B	NM_008198	Mm00433909_m1
Heart	*Fstl1*	*Mus musculus* follistatin-like 1	NM_008047	Mm00433371_m1
Heart	*Alas2*	*Mus musculus* aminolevulinic acid synthase 2, erythroid	NM_009653	Mm00802083_m1
All	*Sfrs9*	*Mus musculus* splicing factor, arginine/serine rich 9	NM_025573	Mm00470546_m1
All	*Guk1*	*Mus musculus* guanylate kinase 1	NM_008193	Mm00433888_m1
All	*Hnrpab*	*Mus musculus* heterogeneous nuclear ribonucleoprotein A/B	NM_010448	Mm00468938_m1

These six target genes were selected based on their functional significance, their statistical significance, their presence as single splice transcripts in microarrays and their availability as Taqman assays designed across splice junctions. *Sfrs9*, *Guk1* and *Hnrpab* were used as housekeeping genes as their expression levels were found to be quite stable across treatments in each of the organs tested.

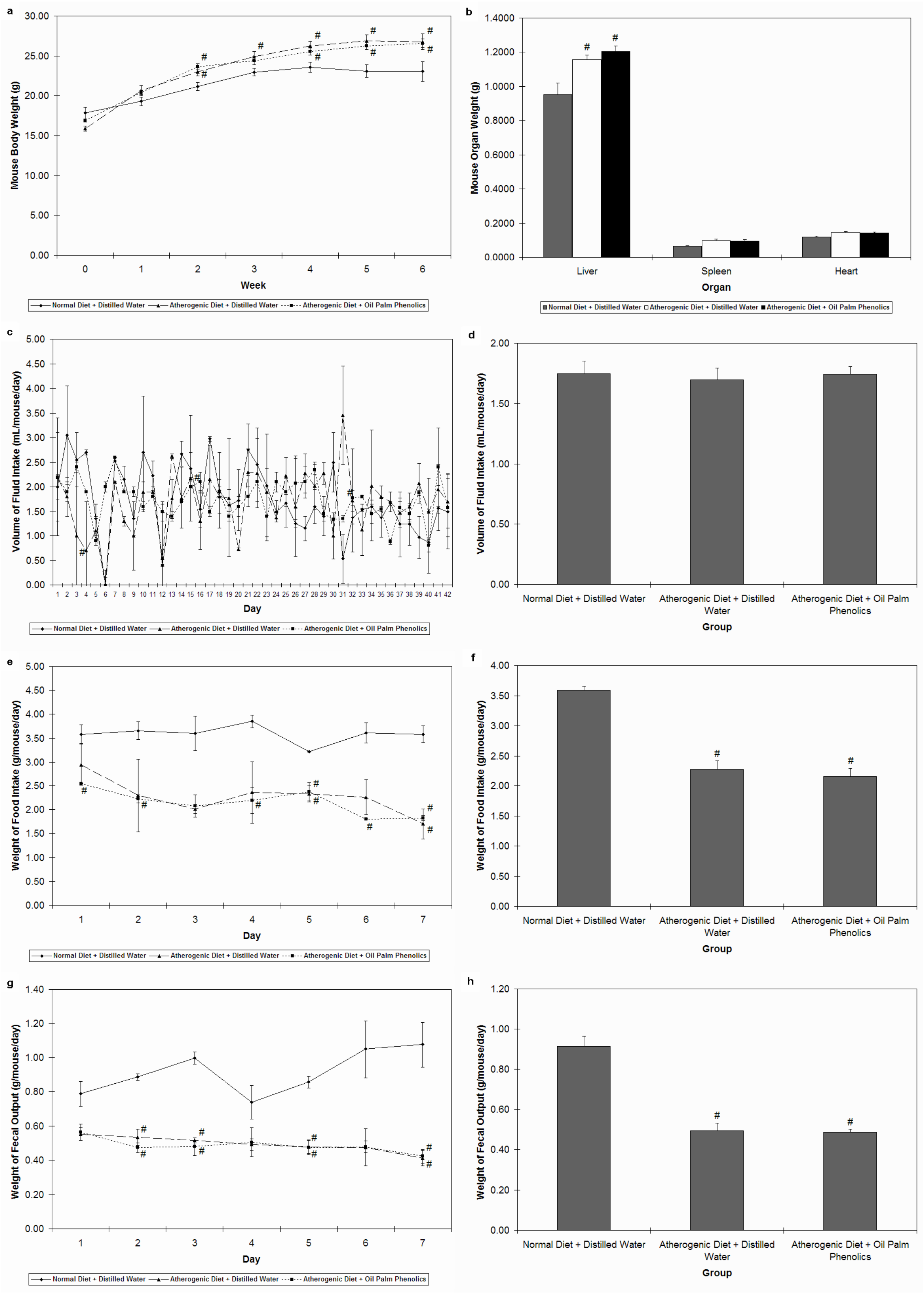

Online Resource 2 Physiology parameters of mice. **a** Body weights; n = 10. **b** Organ weights; n = 10. **c** Timeline of fluid intake; n = 2 cages (of 5 mice per cage). **d** Average daily fluid intake; n = 42 days. **e** Timeline of food intake, n = 2 cages (of 5 mice per cage). **f** Average daily food intake between week two to week three; n = 7 days. **g** Timeline of fecal output, n = 2 cages (of 5 mice per cage). **h** Average daily fecal output between week two to week three; n = 7 days. # $p < 0.05$ vs. Normal Diet + Distilled Water. Values are means ± SEM

Online Resource 3 Hematology and clinical biochemistry parameters of mouse blood samples

Test	Normal Diet + Distilled Water	Atherogenic Diet + Distilled Water	Atherogenic Diet + Oil Palm Phenolics
Hematology	(n = 4)	(n = 4)	(n = 4)
Red Blood Cells ($X10^{12}/L$)	9.93 ± 0.32 ^{a}	10.08 ± 0.21 ^{a}	10.02 ± 0.08 ^{a}
Hemoglobin (g/L)	148 ± 4 ^{a}	149 ± 3 ^{a}	149 ± 1 ^{a}
Hematocrit / Packed Cell Volume (L/L)	0.40 ± 0.01 ^{a}	0.40 ± 0.02 ^{a}	0.40 ± 0.00 ^{a}
Mean Corpuscular Volume (fL)	41 ± 1 ^{a}	40 ± 1 ^{a}	40 ± 1 ^{a}
Mean Corpuscular Hemoglobin Concentration (g/L)	369 ± 6 ^{a}	373 ± 7 ^{a}	372 ± 5 ^{a}
White Blood Cells ($X10^{9}/L$)	2.0 ± 0.6 ^{a}	3.3 ± 0.3 ^{b}	3.0 ± 0.1 ^{b}
Band Neutrophils ($X10^{9}/L$)	0.05 ± 0.01 ^{a}	0.10 ± 0.02 ^{b}	0.10 ± 0.02 ^{b}
Segmented Neutrophils ($X10^{9}/L$)	0.48 ± 0.17 ^{a}	0.83 ± 0.07 ^{b}	0.77 ± 0.06 ^{b}
Lymphocytes ($X10^{9}/L$)	1.36 ± 0.38 ^{a}	2.06 ± 0.22 ^{b}	1.99 ± 0.17 ^{b}
Monocytes ($X10^{9}/L$)	0.09 ± 0.02 ^{a}	0.08 ± 0.04 ^{a}	0.09 ± 0.03 ^{a}
Eosinophils ($X10^{9}/L$)	0.03 ± 0.01 ^{a}	0.06 ± 0.02 ^{a}	0.06 ± 0.03 ^{a}
Basophils ($X10^{9}/L$)	0.00 ± 0.00 ^{a}	0.00 ± 0.00 ^{a}	0.00 ± 0.00 ^{a}
Thrombocytes ($X10^{9}/L$)	533 ± 111 ^{a}	644 ± 37 ^{a}	619 ± 21 ^{a}
Prothrombin (g/L)	79 ± 2 ^{a}	78 ± 2 ^{a}	79 ± 2 ^{a}
Clinical Biochemistry	(n = 8)	(n = 8)	(n = 7)
Alanine Aminotransferase (U/L)	34.4 ± 3.3 ^{a}	41.8 ± 10.7 ^{a}	32.2 ± 5.1 ^{a}
Aspartate Aminotransferase (U/L)	175.2 ± 23.8 ^{a}	174.8 ± 29.3 ^{a}	157.2 ± 32.2 ^{a}
Glucose (mmol/L)	6.0 ± 1.1 ^{a}	5.3 ± 0.4 ^{a,b}	7.4 ± 0.4 ^{a,c}
Serum Total Protein (g/L)	53.8 ± 1.8 ^{a}	53.2 ± 0.9 ^{a}	54.8 ± 0.7 ^{a}
Albumin (g/L)	34.0 ± 0.9 ^{a}	29.4 ± 0.7 ^{b}	31.0 ± 0.7 ^{b}
Globulin (g/L)	19.8 ± 1.1 ^{a}	23.8 ± 0.7 ^{b}	23.7 ± 0.7 ^{b}
Albumin:Globulin	1.8 ± 0.1 ^{a}	1.2 ± 0.1 ^{b}	1.3 ± 0.1 ^{b}
Total Cholesterol (mmol/L)	3.46 ± 0.13 ^{a}	4.77 ± 0.15 ^{b}	4.76 ± 0.19 ^{b}
Triglycerides (mmol/L)	1.05 ± 0.08 ^{a}	1.13 ± 0.04 ^{a}	1.14 ± 0.15 ^{a}
Low-Density Lipoproteins (LDL) (mmol/L)	0.15 ± 0.02 ^{a}	0.26 ± 0.03 ^{b}	0.30 ± 0.06 ^{b}
High-Density Lipoproteins (HDL) (mmol/L)	2.79 ± 0.11 ^{a}	4.05 ± 0.11 ^{b}	3.93 ± 0.14 ^{b}

All data are expressed as means ± SEM. Means with different superscript letters are significantly different ($p < 0.05$). Significant changes caused by the atherogenic diet compared to the normal diet include levels of white blood cells (↑), neutrophils (↑), lymphocytes (↑), glucose (↓), albumin (↓), globulin (↑), ratio of albumin to globulin (↓), total cholesterol (↑), LDL (↑) and HDL (↑). OPP did not cause significant changes in the blood parameters measured, except for normalizing glucose levels in mice fed the atherogenic diet.

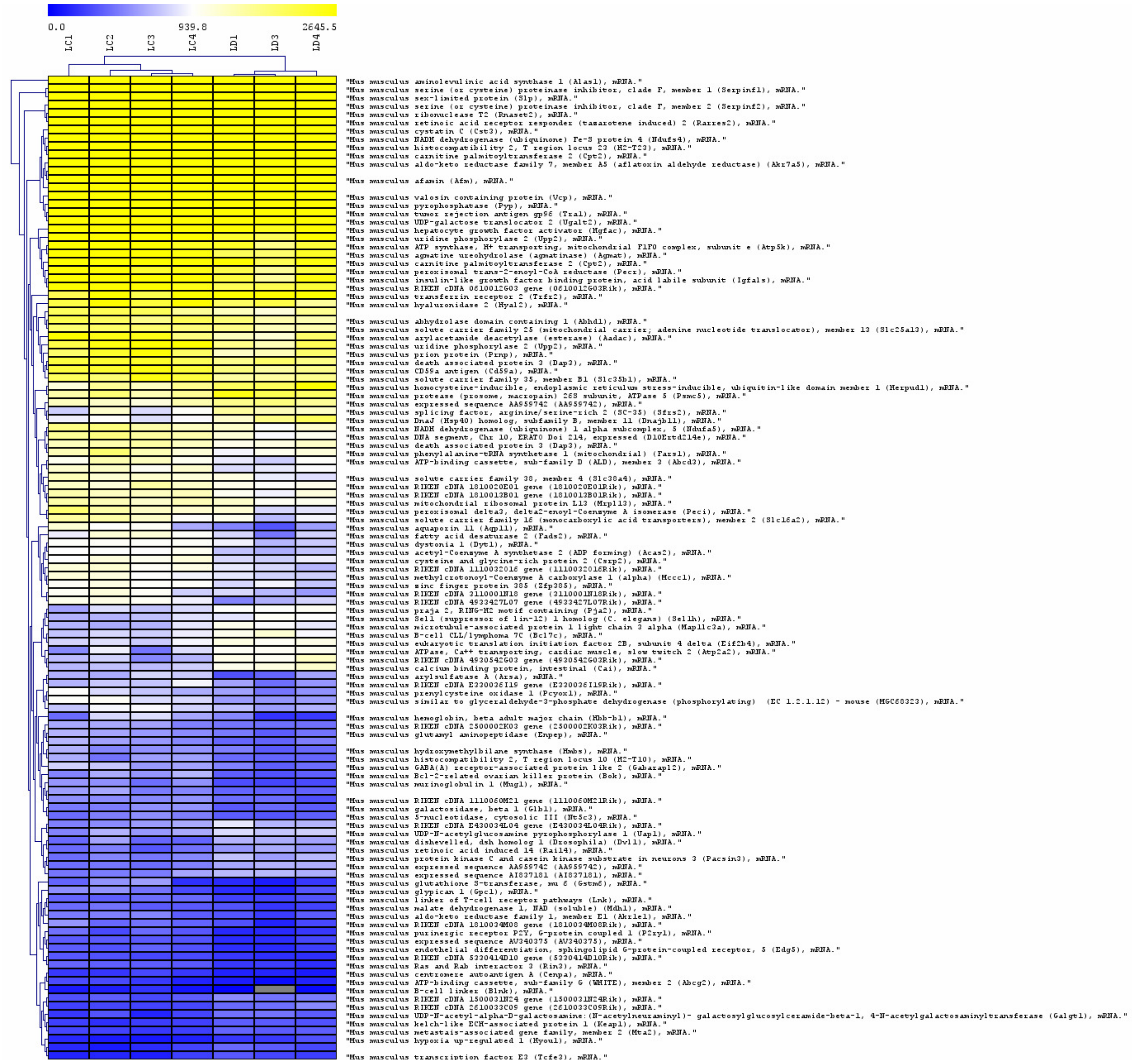

Online Resource 4 An example of the two-way hierarchical clustering analysis carried out on microarray data. Using microarray data obtained from livers of mice in the AD + OPP group versus the AD + DW group as an example, this figure shows the two-way hierarchical clustering analysis carried out on significantly changed genes. Single colour gene expression values are represented using a blue-white-yellow (0 to positive) colour scheme. Grey boxes indicate negative values. Note that the replicates of each group (LC1, LC2, LC3, LC4 in the AD + DW group and LD1, LD3, LD4 in the AD + OPP group) were clustered together within each group but separated from the other group, indicating that the gene expression values selected as significantly changed differentiated the two groups

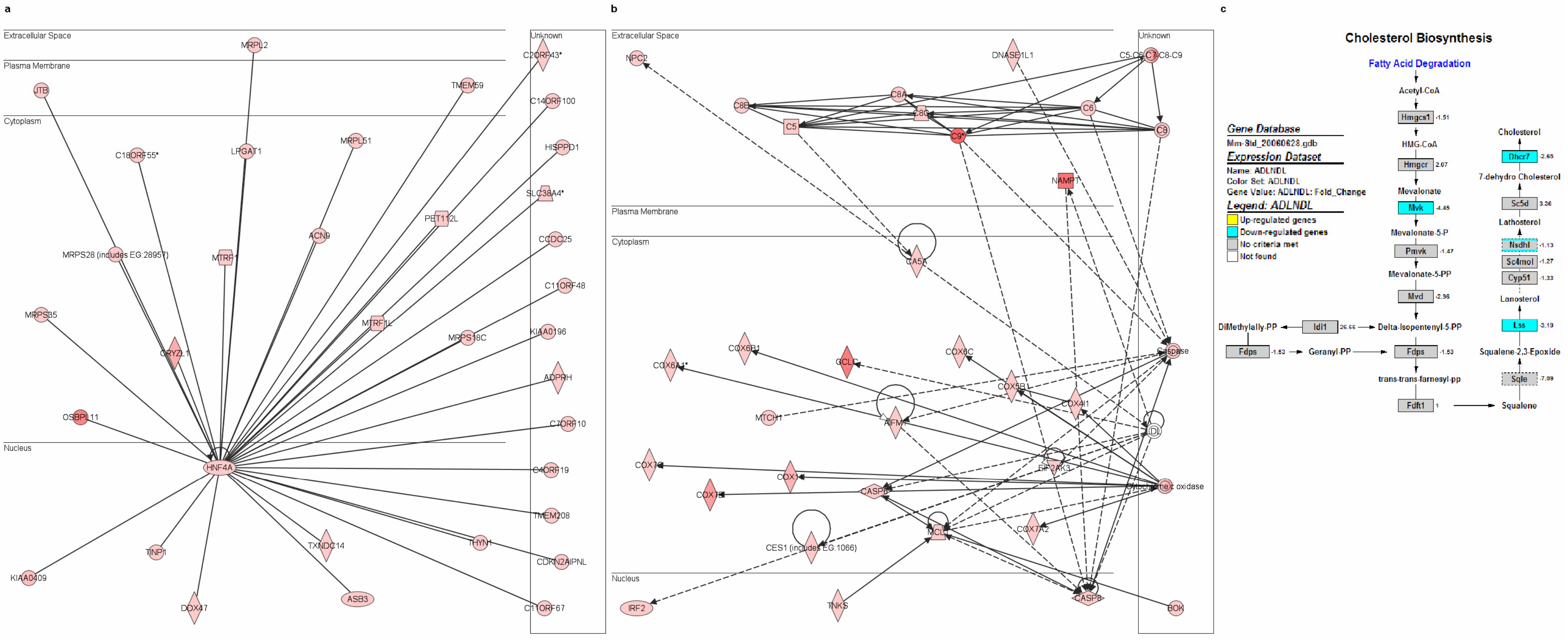

Online Resource 5 Genes regulated by the atherogenic diet in the liver. **a** Genes up-regulated are linked to *Hnf4a*, which is a nuclear factor involved in hepatocyte differentiation. **b** Genes up-regulated include cytochrome c oxidases, caspases and complement genes, which are involved in cell death via apoptosis. **c** Genes down-regulated by the atherogenic diet in the liver cholesterol biosynthesis pathway

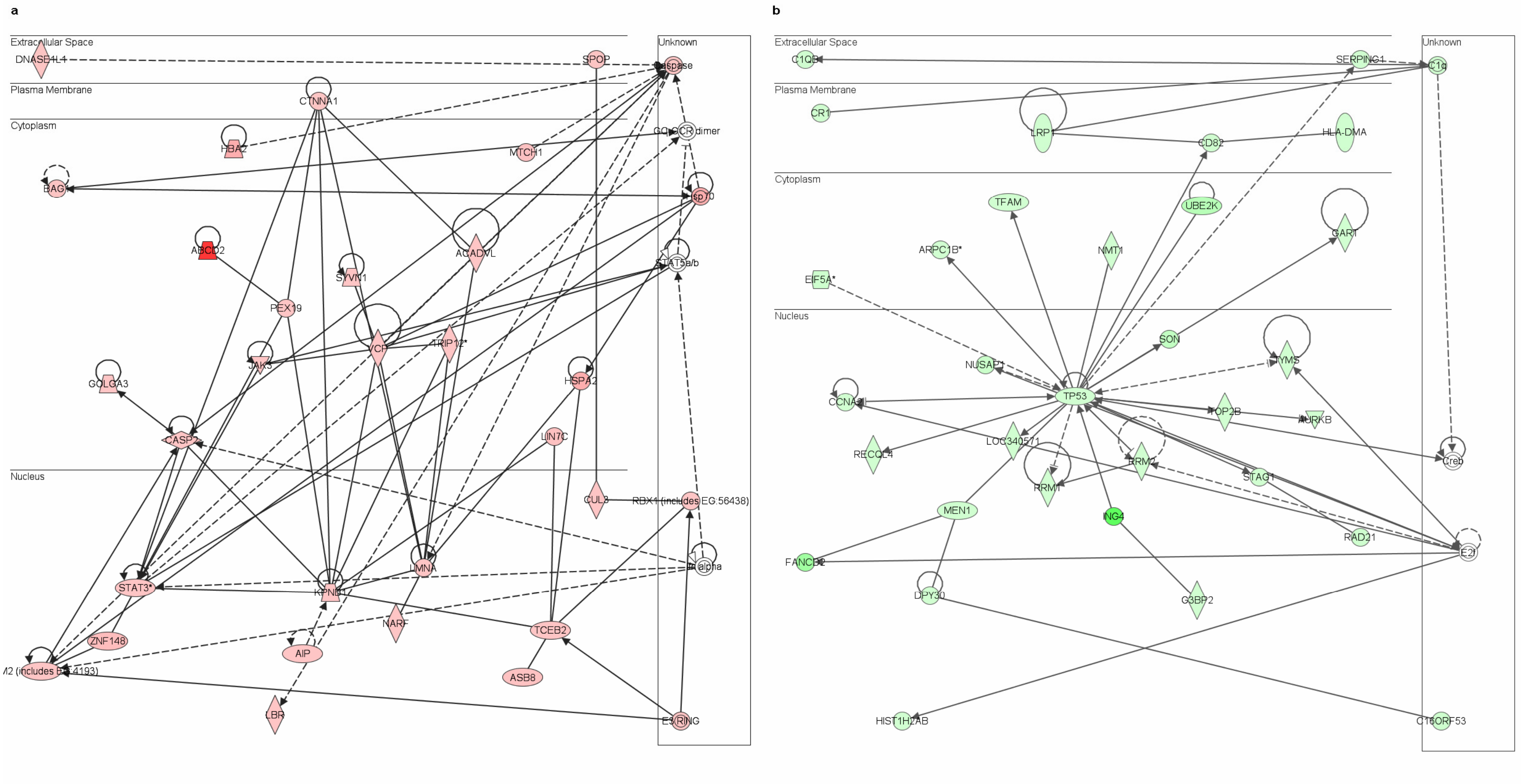

Online Resource 6 Genes regulated by the atherogenic diet in the spleen. **a** Genes up-regulated in the *Stat3* network. The *Stat3* gene plays an important role as a transcription activator in immune system development. However, it has also been implicated to have an inflammatory role as an acute phase response factor. **b** Genes down-regulated in the *Tp53* network. *Tp53* is a tumor suppressor which promotes apoptosis. Down-regulation of this gene suggests increased proliferation of immune cells caused by the atherogenic diet

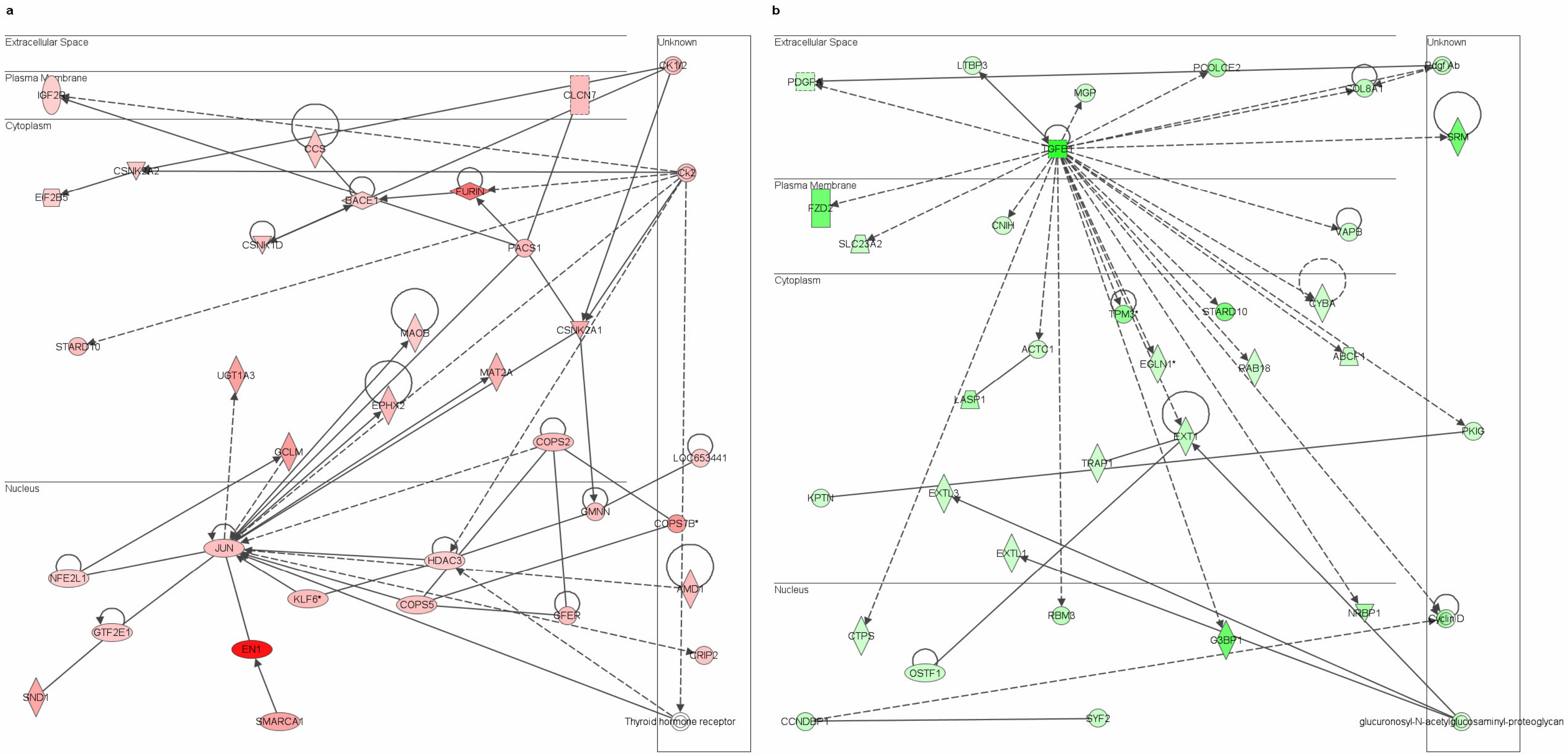

Online Resource 7 Genes regulated by the atherogenic diet in the heart. **a** Genes up-regulated in the *Jun* network. Activation of the *Jun* gene has been implicated in oxidative stress, inflammation, arterial injury and heart disease. **b** Genes down-regulated in the *Tgfb1* network. The *Tgfb* gene has an anti-inflammatory role and has been suggested to be protective in atherosclerosis

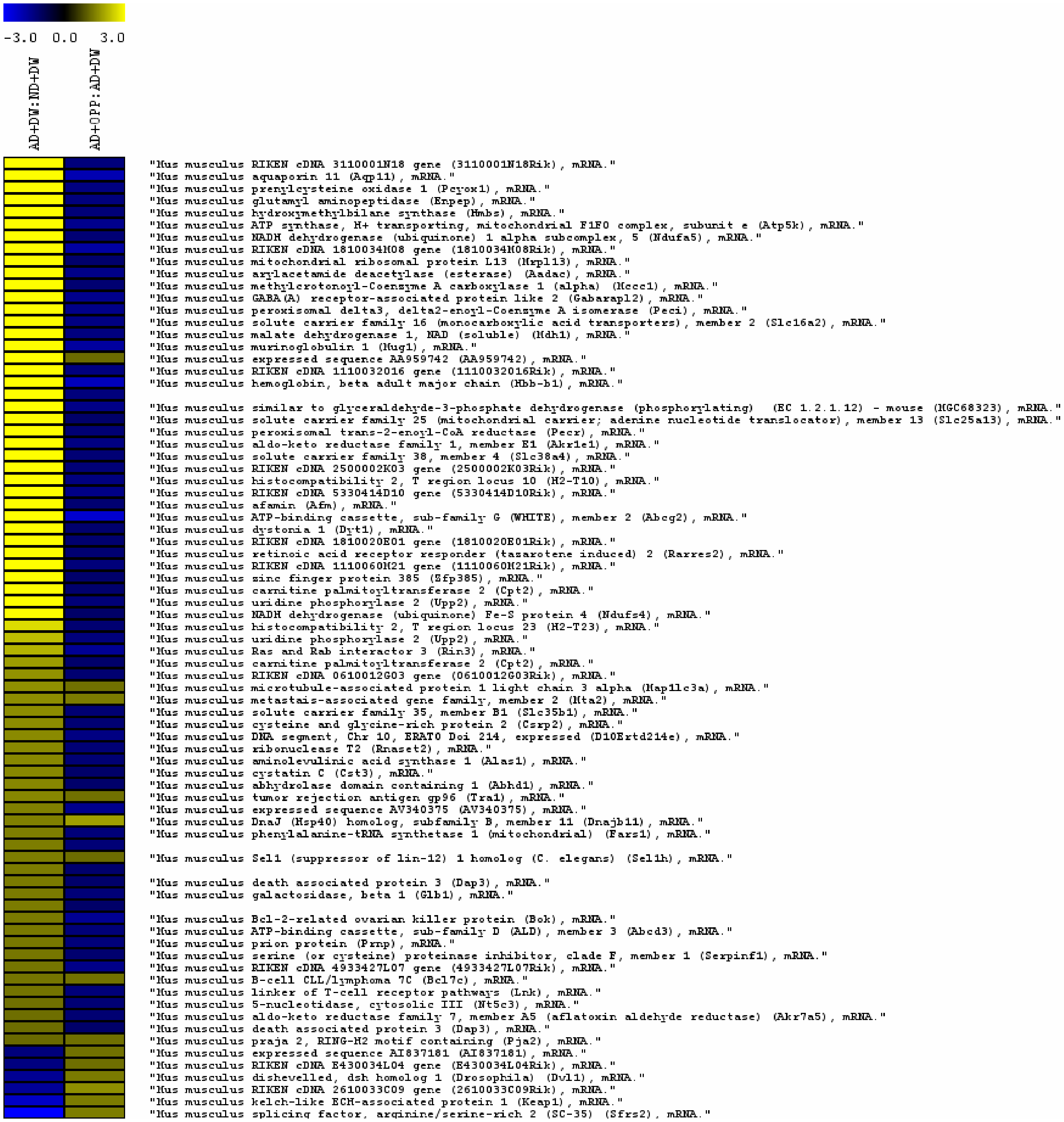

Online Resource 8 Comparison of genes significantly changed by the atherogenic diet and OPP in terms of the direction of fold changes (using data from livers as an example). Values of fold changes are represented using a blue-black-yellow (negative to positive) colour scheme. The |Differential Score| for all genes is more than 20, equivalent to a P Value of less than 0.01

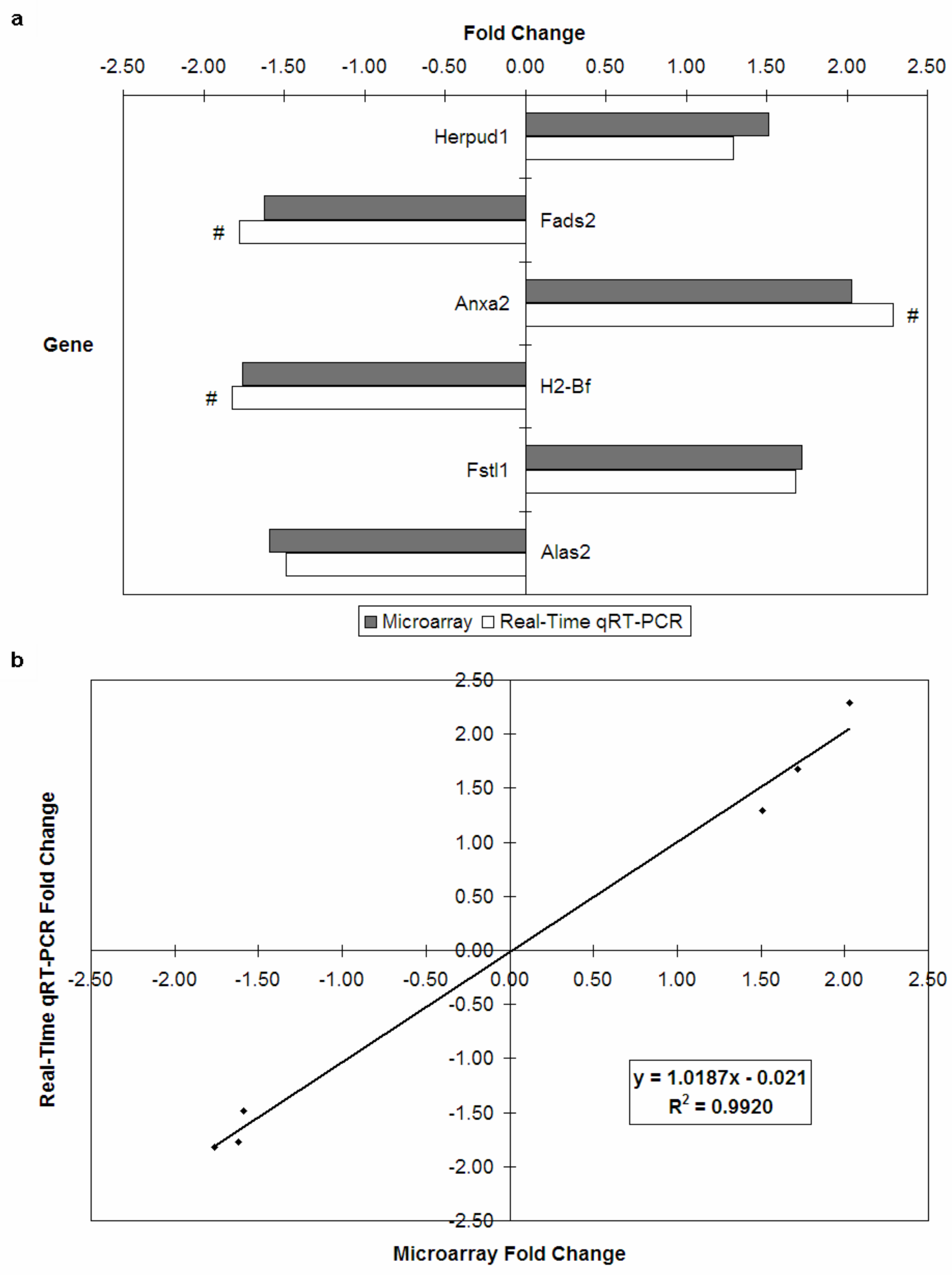

Online Resource 9 Gene expression fold changes of six target genes as determined by microarray and real-time qRT-PCR experiments and their correlation. **a** The direction and magnitude of fold changes obtained from the real-time qRT-PCR technique were comparable to those obtained from the microarray technique. # $p < 0.05$ for gene expression fold changes quantified by real-time PCR experiments as determined by two-tailed unpaired Student's t-test. **b** Validation of the microarray data via real-time qRT-PCR shows that correlation of fold changes obtained by these two gene expression profiling techniques was high with an $R^2 = 0.9920$
