# Supplementary material for: Oil palm phenolics attenuate changes caused by an atherogenic diet in mice
Source: Eur J Nutr. 2012 Apr 11;52(2):443–56. doi: 10.1007/s00394-012-0346-0 (PMC3573186; doi:10.1007/s00394-012-0346-0)
Supplement: Supplementary file 2 — Supplementary material 2 (PDF 1122 kb) [file 394_2012_346_MOESM2_ESM.pdf]

Article Title: Oil palm phenolics attenuate changes caused by an atherogenic diet in mice.

Journal Name: European Journal of Nutrition.

Author Names: Soon-Sen Leow, Shamala Devi Sekaran, Kalyana Sundram, YewAi Tan, Ravigadevi Sambanthamurthi.

Corresponding Author: Ravigadevi Sambanthamurthi; Malaysian Palm Oil Board, No. 6, Persiaran Institusi, Bandar Baru Bangi, 43000 Kajang, Selangor, Malaysia; E-mail: **raviga@mpob.gov.my**.

Supplementary Material 2

This file contains six tables which show lists of genes, as well as GenMAPPs and gene ontologies significantly changed by the atherogenic diet in livers, spleens and hearts of mice. The |Differential Score| for all genes is more than 20, equivalent to a P Value of less than 0.01. All GenMAPPs and gene ontologies had Permuted P Values of less than 0.01, Numbers of Genes Changed of more than or equal to 2 and Z Scores of more than 2.

Table A1 Genes significantly changed by the atherogenic diet in livers.

Table A2 GenMAPPs and gene ontologies significantly changed by the atherogenic diet in livers.

Table A3 Genes significantly changed by the atherogenic diet in spleens.

Table A4 GenMAPPs and gene ontologies significantly changed by the atherogenic diet in spleens.

Table A5 Genes significantly changed by the atherogenic diet in hearts.

Table A6 GenMAPPs and gene ontologies significantly changed by the atherogenic diet in hearts.

TABLE A1					
GENES UP-REGULATED BY THE ATHEROGENIC DIET IN LIVERS					
TargetID	Diff_Score	Fold_Change	Symbol	Accession	Definition
scl17355.9_0-S	371.3336	107.61	Glul	NM_008131.2	Mus musculus glutamate-ammonia ligase (glutamine synthase) (Glul), mRNA.
scl45125.3_164-S	371.3336	91.15	BC006662	NM_145466	Mus musculus cDNA sequence BC006662 (BC006662), mRNA.
scl45459.17_81-S	371.3336	86.72	Kpna3	NM_008466.2	Mus musculus karyopherin (importin) alpha 3 (Kpna3), mRNA.
scl33851.12.1_1-S	371.3336	72.49	1810047C23Rik	NM_138668.1	Mus musculus RIKEN cDNA 1810047C23 gene (1810047C23Rik), mRNA.
scl0012946.2_160-S	371.3336	72.08	Crry	NM_013499.1	Mus musculus complement receptor related protein (Crry), mRNA.
scl42345.1.5_1-S	371.3336	67.99	Tmem30b	NM_178715.2	
scl0073197.1_297-S	371.3336	66.48	D19Ertd703e	NM_029456.1	
scl012314.1_140-S	371.3336	61.53	Calm2	NM_007589	Mus musculus calmodulin 2 (Calm2), mRNA.
scl49312.6.1_7-S	371.3336	57.76	Kng1	NM_023125.2	
scl076959.8_48-S	371.3336	55.45	2210412K09Rik	NM_029814.1	Mus musculus RIKEN cDNA 2210412K09 gene (2210412K09Rik), mRNA.
scl013690.1_50-S	371.3336	51.88	Eif4g2	NM_013507.2	Mus musculus eukaryotic translation initiation factor 4, gamma 2 (Eif4g2), mRNA.
scl39983.12.1_110-S	371.3336	50.69	Nup88	NM_172394.1	Mus musculus nucleoporin 88 (Nup88), mRNA.
scl48279.10.1_23-S	371.3336	50.2	Mrpl39	NM_017404.2	Mus musculus mitochondrial ribosomal protein L39 (Mrpl39), mRNA.
scl0098221.1_97-S	371.3336	49.84	Ga17	NM_145380.1	Mus musculus dendritic cell protein GA17 (Ga17), mRNA.
scl32353.2.4_40-S	371.3336	47.48	Ndufc2	NM_024220.1	
scl015331.1_246-S	371.3336	46.5	Hmgn2	NM_016957.2	Mus musculus high mobility group nucleosomal binding domain 2 (Hmgn2), mRNA.
scl000764.1_2-S	371.3336	45.94	Sumo1	NM_009460.1	
scl40255.4.1_5-S	371.3336	45.52	0610009B22Rik	NM_025319.1	Mus musculus RIKEN cDNA 0610009B22 gene (0610009B22Rik), mRNA.
scl24063.27.1_21-S	371.3336	42.17	Jak1	NM_146145.1	Mus musculus Janus kinase 1 (Jak1), mRNA.
scl0104923.5_14-S	371.3336	39.15	AL024210	NM_134052.1	Mus musculus expressed sequence AL024210 (AL024210), mRNA.
scl0028185.2_148-S	371.3336	39.08	Tomm70a	NM_138599.2	
scl47189.8.1_128-S	371.3336	38.59	Mrpl13	NM_026759.2	Mus musculus mitochondrial ribosomal protein L13 (Mrpl13), mRNA.
scl0020479.2_290-S	371.3336	38.32	Vps4b	NM_009190.1	Mus musculus vacuolar protein sorting 4b (yeast) (Vps4b), mRNA.
scl40878.26_32-S	371.3336	33.32	Nbr1	NM_008676.1	Mus musculus neighbor of Brca1 gene 1 (Nbr1), mRNA.
scl0002761.1_5-S	371.3336	31.94	Ptp4a2	NM_008974.2	Mus musculus protein tyrosine phosphatase 4a2 (Ptp4a2), mRNA.
scl073024.7_20-S	371.3336	31.39	2900064A13Rik	NM_133749.1	Mus musculus RIKEN cDNA 2900064A13 gene (2900064A13Rik), mRNA.
scl28579.2_420-S	371.3336	30.24	Fin14		Mus musculus fibroblast growth factor inducible 14 (Fin14), mRNA.
scl000028.1_0-S	371.3336	29.63	Bccip	NM_025392.1	Mus musculus BRCA2 and CDKN1A interacting protein (Bccip), mRNA.
scl42561.14.1_88-S	371.3336	28.52	Dld	NM_007861.2	Mus musculus dihydrolipoamide dehydrogenase (Dld), mRNA.
scl018606.1_11-S	371.3336	27.74	Enpp2	NM_015744	Mus musculus ectonucleotide pyrophosphatase/phosphodiesterase 2 (Enpp2), mRNA.
scl0017859.1_306-S	371.3336	27.15	Mxi1	NM_010847.1	Mus musculus Max interacting protein 1 (Mxi1), mRNA.
scl19667.9.1_67-S	371.3336	24.95	Rsu1	NM_009105	Mus musculus Ras suppressor protein 1 (Rsu1), mRNA.
scl0015078.1_248-S	371.3336	24.16	H3f3a	NM_008210.2	Mus musculus H3 histone, family 3A (H3f3a), mRNA.
scl0002419.1_3-S	371.3336	24.04	Trappc6b	BC031464	Mus musculus trafficking protein particle complex 6B (Trappc6b), mRNA.
scl48508.5.1_8-S	371.3336	24.04	2310056P07Rik	XM_148611.1	Mus musculus RIKEN cDNA 2310056P07 gene (2310056P07Rik), mRNA.
scl20502.5_452-S	371.3336	22.89	Lin7c	NM_011699	Mus musculus lin 7 homolog c (C. elegans) (Lin7c), mRNA.
scl019231.6_192-S	371.3336	22.32	Ptma	NM_008972.1	Mus musculus prothymosin alpha (Ptma), mRNA.
scl022335.1_61-S	371.3336	22.09	Vdac3	NM_011696.1	Mus musculus voltage-dependent anion channel 3 (Vdac3), mRNA.
scl44723.2_201-S	371.3336	20.77	B230219D22Rik	NM_181278.2	Mus musculus RIKEN cDNA B230219D22 gene (B230219D22Rik), mRNA.
scl0245688.12_1-S	371.3336	20.63	Rbbp7	NM_009031.2	Mus musculus retinoblastoma binding protein 7 (Rbbp7), mRNA.
scl53773.1.1793_9-S	371.3336	20.57	Acsl4	NM_207625.1	Mus musculus acyl-CoA synthetase long-chain family member 4 (Acsl4), transcript variant 2, mRNA.
scl40787.10.7_5-S	371.3336	19.16	Psmd12	NM_025894.1	Mus musculus proteasome (prosome, macropain) 26S subunit, non-ATPase, 12 (Psmd12), mRNA.
scl55039.15_103-S	371.3336	19.02	Ddx3x	NM_010028	Mus musculus DEAD/H (Asp-Glu-Ala-Asp/His) box polypeptide 3, X-linked (Ddx3x), mRNA.
scl070356.1_235-S	371.3336	18.51	St13	NM_133726.1	Mus musculus suppression of tumorigenicity 13 (St13), mRNA.
scl020320.8_193-S	371.3336	18.34	Sdfr1	NM_009145.1	Mus musculus stromal cell derived factor receptor 1 (Sdfr1), mRNA.
scl030926.11_17-S	371.3336	17.86	Txnl2	NM_023140.3	Mus musculus thioredoxin-like 2 (Txnl2), mRNA.
scl076808.4_30-S	371.3336	17.45	Rpl18a	XM_194410.2	
scl28744.16_312-S	371.3336	16.99	Anxa4	NM_013471	Mus musculus annexin A4 (Anxa4), mRNA.
scl000086.1_135-S	371.3336	16.96	AA959742	NM_133807.1	Mus musculus expressed sequence AA959742 (AA959742), mRNA.
scl30665.8.1_14-S	371.3336	15.98	1110032O16Rik	XM_133813.4	Mus musculus RIKEN cDNA 1110032O16 gene (1110032O16Rik), mRNA.
scl20874.15.4_12-S	371.3336	15.55	Psmd14	NM_021526	Mus musculus proteasome (prosome, macropain) 26S subunit, non-ATPase, 14 (Psmd14), mRNA.
scl068051.5_64-S	371.3336	14.68	Nutf2	NM_026532.2	Mus musculus nuclear transport factor 2 (Nutf2), mRNA.
scl41197.5.1_16-S	371.3336	14.13	0610009H04Rik	NM_018854.3	Mus musculus RIKEN cDNA 0610009H04 gene (0610009H04Rik), mRNA.

scl30059.16.1_6-S	371.3336	13.31	Mpp6	NM_019939.1	Mus musculus membrane protein, palmitoylated 6 (MAGUK p55 subfamily member 6) (Mpp6), mRNA.
scl0014122.1_142-S	371.3336	12.83	Fbp3		
scl54930.9.1_1-S	371.3336	12.6	Hprt	NM_013556	
scl49695.8_112-S	371.3336	12.48	Vapa	NM_013933.2	Mus musculus vesicle-associated membrane protein, associated protein A (Vapa), mRNA.
scl20734.8_508-S	371.3336	12.39	Plekha3	NM_031256.2	Mus musculus pleckstrin homology domain-containing, family A (phosphoinositide binding specific) member 3 (Plekha3), mRNA.
scl0056351.1_126-S	371.3336	12.22	Tebp	NM_019766.2	Mus musculus telomerase binding protein, p23 (Tebp), mRNA.
scl31078.5_227-S	371.3336	12.02	2210412D01Rik	NM_133722.1	Mus musculus RIKEN cDNA 2210412D01 gene (2210412D01Rik), mRNA.
scl017749.3_11-S	371.3336	11.46	Polr2k	NM_023127	Mus musculus polymerase (RNA) II (DNA directed) polypeptide K (Polr2k), mRNA.
scl071955.1_257-S	371.3336	10.46	2400003C14Rik	NM_028018.1	Mus musculus RIKEN cDNA 2400003C14 gene (2400003C14Rik), mRNA.
scl29306.21.1_2-S	371.3336	10.32	Slc25a13	NM_015829.1	Mus musculus solute carrier family 25 (mitochondrial carrier; adenine nucleotide translocator), member 13 (Slc25a13), mRNA.
scl056433.4_275-S	371.3336	10.23	Vps29	NM_019780.1	Mus musculus vacuolar protein sorting 29 (S. pombe) (Vps29), mRNA.
scl066448.4_32-S	371.3336	9.84	Mrpl20	NM_025570.1	Mus musculus mitochondrial ribosomal protein L20 (Mrpl20), mRNA.
scl076846.5_166-S	371.3336	9.71	3010033P07Rik	NM_029767.1	
scl42239.4_289-S	371.3336	9.56	Npc2	NM_023409.3	Mus musculus Niemann Pick type C2 (Npc2), mRNA.
scl0069354.1_68-S	371.3336	9.5	Slc38a4	NM_027052.2	Mus musculus solute carrier family 38, member 4 (Slc38a4), mRNA.
scl25138.1_25-S	371.3336	9.42	1110001A12Rik	NM_029571.1	Mus musculus RIKEN cDNA 1110001A12 gene (1110001A12Rik), mRNA.
scl35003.22_120-S	371.3336	8.99	Adam9	NM_007404.1	Mus musculus a disintegrin and metalloproteinase domain 9 (meltrin gamma) (Adam9), mRNA.
scl0003847.1_4-S	371.3336	8.52	1110005A23Rik	NM_025364	Mus musculus RIKEN cDNA 1110005A23 gene (1110005A23Rik), mRNA.
scl000299.1_29-S	371.3336	8.18	Esd	NM_016903.2	
scl0020042.1_302-S	371.3336	7.99		AK002848.1	Mus musculus adult male kidney cDNA, RIKEN full-length enriched library, clone:0610039M20 product:ribosomal protein S12, full insert sequence.
scl069046.1_193-S	371.3336	7.99	Hbld2	NM_026921.2	Mus musculus HESB like domain containing 2 (Hbld2), mRNA.
scl39445.1.46_133-S	371.3336	7.92	Tex2	XM_203370.3	Mus musculus testis expressed gene 2 (Tex2), mRNA.
scl44057.9_18-S	371.3336	7.47	Elovl2	NM_019423.1	Mus musculus elongation of very long chain fatty acids (FEN1/Elo2, SUR4/Elo3, yeast)-like 2 (Elovl2), mRNA.
scl27606.14.1_65-S	371.3336	7.44	Afm	NM_145146.1	Mus musculus afamin (Afm), mRNA.
scl069354.1_30-S	371.3336	7.26	Slc38a4	NM_027052.2	Mus musculus solute carrier family 38, member 4 (Slc38a4), mRNA.
scl0319152.1_297-S	371.3336	7.23	Hist1h3h	NM_178206	Mus musculus histone 1, H3h (Hist1h3h), mRNA.
scl0002303.1_158-S	371.3336	7.03	Serpina1a	NM_009243	Mus musculus serine (or cysteine) proteinase inhibitor, clade A, member 1a (Serpina1a), mRNA.
scl33920.14_231-S	371.3336	6.87	Gsr	NM_010344.3	Mus musculus glutathione reductase 1 (Gsr), mRNA.
scl42464.2_123-S	371.3336	6.73	1110002B05Rik	NM_134054.1	Mus musculus RIKEN cDNA 1110002B05 gene (1110002B05Rik), mRNA.
scl35713.2.1_23-S	371.3336	6.6	2300009A05Rik	XM_204313.2	Mus musculus RIKEN cDNA 2300009A05 gene (2300009A05Rik), mRNA.
scl0066885.2_329-S	371.3336	6.33	Acadsb	NM_025826.1	Mus musculus acyl-Coenzyme A dehydrogenase, short/branched chain (Acadsb), mRNA.
scl41578.9_407-S	371.3336	5.67	Skp1a	NM_011543.2	Mus musculus S-phase kinase-associated protein 1A (Skp1a), mRNA.
scl0070231.1_327-S	371.3336	4.53	Gorasp2	NM_027352.2	Mus musculus golgi reassembly stacking protein 2 (Gorasp2), mRNA.
scl067248.2_33-S	371.3336	3.59	Rpl39	NM_026055.1	Mus musculus ribosomal protein L39 (Rpl39), mRNA.
scl019896.4_30-S	371.3336	3.27	Rpl10a	NM_011287.1	Mus musculus ribosomal protein L10A (Rpl10a), mRNA.
scl0110954.5_51-S	371.3336	3.12	Rpl10	NM_052835	Mus musculus ribosomal protein 10 (Rpl10), mRNA.
scl19726.14.1_76-S	371.0959	14.78	G431001I09Rik	NM_133837.2	Mus musculus RIKEN cDNA G431001I09 gene (G431001I09Rik), mRNA.
scl068735.6_63-S	370.6798	5.08	Mrps18c	XM_194230.3	
scl070456.4_0-S	370.0436	20.03	2610205H19Rik	NM_027430.1	Mus musculus RIKEN cDNA 2610205H19 gene (2610205H19Rik), mRNA.
scl0078655.1_180-S	366.8107	35.32	Eif3s1	NM_144545.1	Mus musculus eukaryotic translation initiation factor 3, subunit 1 alpha (Eif3s1), mRNA.
scl055980.1_58-S	363.3732	42.73	Impa1	NM_018864.3	Mus musculus inositol (myo)-1(or 4)-monophosphatase 1 (Impa1), mRNA.
scl072201.1_290-S	362.6922	20.14	2600013N14Rik	NM_152812.1	Mus musculus RIKEN cDNA 2600013N14 gene (2600013N14Rik), mRNA.
scl16639.44.189_5-S	361.2323	8.43	Fn1	XM_129845.3	Mus musculus fibronectin 1 (Fn1), mRNA.
scl43183.10.1_88-S	358.9626	23.99	Sip1	NM_025656.1	Mus musculus survivor of motor neuron protein interacting protein 1 (Sip1), mRNA.
scl013006.15_44-S	358.7571	43.53	Cspg6	NM_007790.2	Mus musculus chondroitin sulfate proteoglycan 6 (Cspg6), mRNA.
scl0114893.1_279-S	357.7791	9	Tes3	NM_033623.1	Mus musculus testis derived transcript 3 (Tes3), mRNA.
scl31053.2.1_38-S	356.1904	5.39	1810020E01Rik	NM_025460.1	Mus musculus RIKEN cDNA 1810020E01 gene (1810020E01Rik), mRNA.
scl30789.23.1_30-S	355.023	64.16	Copb1	NM_033370	Mus musculus coatomer protein complex, subunit beta 1 (Copb1), mRNA.
scl018538.1_192-S	354.4935	16.08	Pcna	NM_011045.1	Mus musculus proliferating cell nuclear antigen (Pcna), mRNA.
scl019988.6_33-S	354.2966	8.52	Rpl6	NM_011290	Mus musculus ribosomal protein L6 (Rpl6), mRNA.
scl52780.10_0-S	353.8468	37.83	Slc3a2	NM_008577.2	Mus musculus solute carrier family 3 (activators of dibasic and neutral amino acid transport), member 2 (Slc3a2), mRNA.
scl29313.9.1_4-S	351.7892	6.66	Pon3	NM_173006.1	Mus musculus paraoxonase 3 (Pon3), mRNA.
scl46659.10_50-S	351.5438	3.69	Zfp385	NM_013866.1	Mus musculus zinc finger protein 385 (Zfp385), mRNA.
scl16458.7.1_28-S	349.3582	14.03	Thap4	NM_025920	Mus musculus THAP domain containing 4 (Thap4), mRNA.
scl020430.29_22-S	345.3049	6.25	Cyfip1	NM_011370.1	Mus musculus cytoplasmic FMR1 interacting protein 1 (Cyfip1), mRNA.
scl0070351.2_206-S	345.0587	10.16	Ppp4r1	NM_146081.1	Mus musculus protein phosphatase 4, regulatory subunit 1 (Ppp4r1), mRNA.

scl00268996.2_185-S	344.1229	8.52	Ss18	NM_009280.1	Mus musculus synovial sarcoma translocation, Chromosome 18 (Ss18), mRNA.
scl0013809.2_54-S	344.0056	62.76	Enpep	NM_007934.1	Mus musculus glutamyl aminopeptidase (Enpep), mRNA.
scl020714.5_72-S	341.4685	3.01	Serpina3k	XM_127127.2	Mus musculus serine (or cysteine) proteinase inhibitor, clade A, member 3K (Serpina3k), mRNA.
scl017448.9_233-S	340.0377	46.43	Mdh2	NM_008617.2	Mus musculus malate dehydrogenase 2, NAD (mitochondrial) (Mdh2), mRNA.
scl35879.4_19-S	338.8509	10	Sdhd	NM_025848.1	Mus musculus succinate dehydrogenase complex, subunit D, integral membrane protein (Sdhd), mRNA.
scl0056334.1_201-S	338.6403	7.77	1110032D12Rik	NM_019770.1	Mus musculus RIKEN cDNA 1110032D12 gene (1110032D12Rik), mRNA.
scl43225.27.1_94-S	338.5223	10.03	Scfd1	XM_126906.2	
scl17471.3_7-S	337.0371	38.58	Ppp1r15b	XM_148953.1	Mus musculus protein phosphatase 1, regulatory (inhibitor) subunit 15b (Ppp1r15b), mRNA.
scl41687.5_165-S	336.4478	5.86	D11Ertd603e	NM_026023.3	Mus musculus DNA segment, Chr 11, ERATO Doi 603, expressed (D11Ertd603e), mRNA.
scl020091.3_53-S	335.8276	18.11	Rps3a	NM_016959.2	Mus musculus ribosomal protein S3a (Rps3a), mRNA.
scl17047.9.4_6-S	334.9648	13.03	BC013667	NM_172266.1	Mus musculus cDNA sequence BC013667 (BC013667), mRNA.
scl069010.2_129-S	332.558	4.66	Anapc13	NM_181394.1	
scl00102103.1_84-S	329.8437	37.94	Mtus1	NM_178902.3	Mus musculus mitochondrial tumor suppressor 1 (Mtus1), mRNA.
scl098766.1_292-S	326.9279	47.01	Ubadc1	NM_133835.1	Mus musculus ubiquitin associated domain containing 1 (Ubadc1), mRNA.
scl069082.11_146-S	326.0119	7.67	2610312B22Rik	NM_026934.2	Mus musculus RIKEN cDNA 2610312B22 gene (2610312B22Rik), mRNA.
scl35703.13_436-S	324.5334	54.22	Map2k1	NM_008927.1	Mus musculus mitogen activated protein kinase kinase 1 (Map2k1), mRNA.
scl38757.5.1_81-S	324.099	2.48	Ndg2	NM_175329.3	
scl36650.13.1_185-S	323.1563	10.24	Bckdhb	NM_199195.1	Mus musculus branched chain ketoacid dehydrogenase E1, beta polypeptide (Bckdhb), mRNA.
scl36089.6_17-S	321.0375	40.56	2310075A12Rik	NM_178027.3	
scl030931.1_245-S	321.0271	5.46	Dyt1	NM_144884.1	Mus musculus dystonia 1 (Dyt1), mRNA.
scl31746.2.26_98-S	317.7933	9.3	1500016L11Rik	NM_026885.1	Mus musculus RIKEN cDNA 1500016L11 gene (1500016L11Rik), mRNA.
scl43053.27.1_6-S	317.5293	37.45	Gphn	NM_172952.1	Mus musculus gephyrin (Gphn), mRNA.
scl098256.13_10-S	317.3327	9.2	Kmo	NM_133809.1	Mus musculus kynurenine 3-monooxygenase (kynurenine 3-hydroxylase) (Kmo), mRNA.
scl00319154.1_299-S	316.9737	16.05	Hist2h3b	NM_178215	Mus musculus histone 2, H3b (Hist2h3b), mRNA.
scl0001121.1_1-S	313.9537	49.86	Ndufa5	NM_026614.1	Mus musculus NADH dehydrogenase (ubiquinone) 1 alpha subcomplex, 5 (Ndufa5), mRNA.
scl0002995.1_2-S	313.3164	38.39	Hprt	NM_013556.1	Mus musculus hypoxanthine guanine phosphoribosyl transferase (Hprt), mRNA.
GI_6671508-S	310.7679	3.34	Actb	NM_007393.1	Mus musculus actin, beta, cytoplasmic (Actb), mRNA.
scl38222.21_67-S	310.0428	5.57	Sash1	NM_175155.3	
scl54409.6_0-S	309.438	5.96	Tcte1l	NM_025975.3	Mus musculus t-complex-associated-testis-expressed 1-like (Tcte1l), mRNA.
scl068098.1_43-S	307.6499	8.43	Rchy1	NM_026557.2	Mus musculus ring finger and CHY zinc finger domain containing 1 (Rchy1), mRNA.
scl014569.11_60-S	306.8387	15	Gdi3	NM_008112.2	Mus musculus guanosine diphosphate (GDP) dissociation inhibitor 3 (Gdi3), mRNA.
scl18614.9.1_72-S	303.6953	5.64	Hao1	NM_010403	Mus musculus hydroxyacid oxidase 1, liver (Hao1), mRNA.
scl0277333.1_280-S	303.2733	11.28	MGC68323	NM_199472.1	Mus musculus similar to glyceraldehyde-3-phosphate dehydrogenase (phosphorylating) (EC 1.2.1.12) - mouse (MGC68323), mRNA.
scl000567.1_25-S	303.1937	6.25	Clcn3	NM_007711.1	Mus musculus chloride channel 3 (Clcn3), transcript variant a, mRNA.
scl068581.2_286-S	301.7039	5.16	1110014C03Rik	NM_026775.2	Mus musculus RIKEN cDNA 1110014C03 gene (1110014C03Rik), mRNA.
scl000011.1_73-S	301.0297	48.05	Strn3	NM_052973	Mus musculus striatin, calmodulin binding protein 3 (Strn3), mRNA.
scl011964.1_274-S	300.3117	5.65	Atp6v1a1	NM_007508.2	Mus musculus ATPase, H+ transporting, V1 subunit A, isoform 1 (Atp6v1a1), mRNA.
scl014571.1_22-S	297.0123	35.86	Gpd2	NM_010274.2	Mus musculus glycerol phosphate dehydrogenase 2, mitochondrial (Gpd2), mRNA.
scl069713.3_39-S	296.841	6.72	Pin4	XM_135990	Mus musculus protein (peptidyl-prolyl cis/trans isomerase) NIMA-interacting, 4 (parvulin) (Pin4), mRNA.
scl0276770.1_104-S	296.1648	3.57	Eif5a	NM_181582.2	Mus musculus eukaryotic translation initiation factor 5A (Eif5a), mRNA.
scl29303.3.1_9-S	296.0498	2.67	Shfdg1	NM_009169.1	Mus musculus split hand/foot deleted gene 1 (Shfdg1), mRNA.
scl015289.1_26-S	294.1043	296.52	Hmgb1	NM_010439.2	Mus musculus high mobility group box 1 (Hmgb1), mRNA.
scl053610.12_290-S	293.4882	53.71	Nono	NM_023144.1	Mus musculus non-POU-domain-containing, octamer binding protein (Nono), mRNA.
scl47166.29.1_41-S	292.6075	8.01	E430025E21Rik	NM_153548.2	Mus musculus RIKEN cDNA E430025E21 gene (E430025E21Rik), mRNA.
scl37474.12.36_46-S	291.3305	16.21	Cct2	NM_007636.1	Mus musculus chaperonin subunit 2 (beta) (Cct2), mRNA.
scl066073.8_269-S	291.2558	5.67	0610040B21Rik	NM_025334.2	Mus musculus RIKEN cDNA 0610040B21 gene (0610040B21Rik), mRNA.
scl056330.3_4-S	289.6497	15.8	Pdcd5	NM_019746.2	Mus musculus programmed cell death 5 (Pdcd5), mRNA.
scl0056388.1_45-S	286.9483	20.14	Cyp3a25	NM_019792.1	Mus musculus cytochrome P450, family 3, subfamily a, polypeptide 25 (Cyp3a25), mRNA.
scl39118.3_388-S	285.9748	9.24	Perp	NM_022032.1	Mus musculus PERP, TP53 apoptosis effector (Perp), mRNA.
scl026374.20_270-S	285.6619	6.71	C80879	NM_011931	Mus musculus expressed sequence C80879 (C80879), mRNA.
scl081702.1_30-S	285.1045	34.83	Ankrd17	NM_198010.2	Mus musculus ankyrin repeat domain 17 (Ankrd17), transcript variant 1, mRNA.
scl0067089.2_186-S	284.9155	31.81	Psmc6	NM_025959.2	Mus musculus proteasome (prosome, macropain) 26S subunit, ATPase, 6 (Psmc6), mRNA.
scl31133.10.1_10-S	284.5258	7.47	Idh2	NM_173011.1	Mus musculus isocitrate dehydrogenase 2 (NADP+), mitochondrial (Idh2), mRNA.
scl27683.19_361-S	283.9397	5.7	Srp72	XM_132143.3	Mus musculus signal recognition particle 72 (Srp72), mRNA.
scl37402.3_73-S	282.4188	3.45	Sas	NM_025982	Mus musculus sarcoma amplified sequence (Sas), mRNA.
scl093692.3_322-S	281.5071	14.94	Glrx1	NM_053108	Mus musculus glutaredoxin 1 (thioltransferase) (Glrx1), mRNA.

scl067530.3_0-S	279.4085	15.67	Uqcrb	NM_026219.1	Mus musculus ubiquinol-cytochrome c reductase binding protein (Uqcrb), mRNA.
scl069257.1_70-S	277.9359	10.03	Elf2	NM_023502.1	Mus musculus E74-like factor 2 (Elf2), mRNA.
scl25713.4.87_24-S	275.9263	4.4	Chchd7	NM_181391.1	
scl30629.5.1_26-S	275.0739	2.35	Vkorc1	NM_178600.2	Mus musculus vitamin K epoxide reductase complex, subunit 1 (Vkorc1), mRNA.
scl067345.15_210-S	274.8502	40.94	1700056O17Rik	NM_026101.2	Mus musculus RIKEN cDNA 1700056O17 gene (1700056O17Rik), mRNA.
scl51339.12_2-S	273.9232	5.8	Fech	NM_007998.3	Mus musculus ferrochelatase (Fech), mRNA.
scl017836.18_18-S	273.8591	22.36	Mug1	NM_008645.2	Mus musculus murinoglobulin 1 (Mug1), mRNA.
scl0023808.2_287-S	272.4559	21.52	Ash2l	NM_011791.1	Mus musculus ash2 (absent, small, or homeotic)-like (Drosophila) (Ash2l), mRNA.
scl075705.2_53-S	271.6064	10.64	Eif4b	NM_145625.1	Mus musculus eukaryotic translation initiation factor 4B (Eif4b), mRNA.
scl00218442.1_227-S	269.9361	38.26	A130038L21Rik	NM_172588.2	Mus musculus RIKEN cDNA A130038L21 gene (A130038L21Rik), mRNA.
scl026451.5_30-S	269.1502	2.24	Rpl27a	NM_011975.2	Mus musculus ribosomal protein L27a (Rpl27a), mRNA.
scl0319195.6_117-S	268.358	2.82	Rpl17	NM_001002239	Mus musculus ribosomal protein L17 (Rpl17), mRNA.
scl0230484.9_241-S	267.856	6.5	Usp1	NM_146144.2	Mus musculus ubiquitin specific protease 1 (Usp1), mRNA.
scl000091.1_88-S	267.3836	3.27	Phf10	NM_024250.3	Mus musculus RIKEN cDNA 1600012H06 gene (1600012H06Rik), mRNA.
scl019946.1_13-S	266.7487	49.27	Rpl30	NM_009083.2	Mus musculus ribosomal protein L30 (Rpl30), mRNA.
scl22246.19.1_4-S	265.7365	36.37	Mccc1	NM_023644.2	Mus musculus methylcrotonoyl-Coenzyme A carboxylase 1 (alpha) (Mccc1), mRNA.
scl056275.3_18-S	265.1182	32.71	Rbm14	NM_019869.1	Mus musculus RNA binding motif protein 14 (Rbm14), mRNA.
gi_30794511_ref_NM_01	264.1065	59.11	Hmbs	NM_013551.1	Mus musculus hydroxymethylbilane synthase (Hmbs), mRNA.
scl050850.17_73-S	263.7929	5.33	Spg4	NM_016962.1	Mus musculus spastic paraplegia 4 homolog (human) (Spg4), mRNA.
scl0001735.1_49-S	262.9908	2.21	RPS18	NM_011296.1	Mus musculus ribosomal protein S18 (Rps18), mRNA.
scl21970.4.1_6-S	261.8288	4.37	Mapbpip	NM_031248.3	Mus musculus mitogen activated protein binding protein interacting protein (Mapbpip), mRNA.
scl28865.3.1_49-S	261.4618	2.49	Vamp8	NM_016794.2	Mus musculus vesicle-associated membrane protein 8 (Vamp8), mRNA.
scl068028.3_20-S	260.5836	104.83	3110001N18Rik	NM_026517.1	Mus musculus RIKEN cDNA 3110001N18 gene (3110001N18Rik), mRNA.
scl18742.7_10-S	260.5248	5.77	Slc30a4	NM_011774	Mus musculus solute carrier family 30 (zinc transporter), member 4 (Slc30a4), mRNA.
scl42361.4.1_12-S	260.2821	37.1	2810055F11Rik	NM_026038.1	Mus musculus RIKEN cDNA 2810055F11 gene (2810055F11Rik), mRNA.
scl0066660.1_237-S	259.2409	4.96	5730555F13Rik	NM_025690.1	Mus musculus RIKEN cDNA 5730555F13 gene (5730555F13Rik), mRNA.
scl0021969.2_46-S	258.9649	32.48	Top1	NM_009408.1	Mus musculus topoisomerase (DNA) I (Top1), mRNA.
scl0050908.1_300-S	258.809	38.39	C1s	NM_144938.1	Mus musculus complement component 1, s subcomponent (C1s), mRNA.
scl067529.7_122-S	256.0323	45.76	Fgfr1op2	NM_026218.1	Mus musculus FGFR1 oncogene partner 2 (Fgfr1op2), mRNA.
scl23236.14.1_102-S	255.8176	5.75	1700108L22Rik	NM_145988.1	Mus musculus RIKEN cDNA 1700108L22 gene (1700108L22Rik), mRNA.
scl013030.16_220-S	255.8056	3.18	Ctsb	NM_007798.1	Mus musculus cathepsin B (Ctsb), mRNA.
scl52776.8.1_198-S	254.2141	6.37	Polr2g	NM_026329.1	Mus musculus polymerase (RNA) II (DNA directed) polypeptide G (Polr2g), mRNA.
scl46593.3.1_27-S	253.4823	2.31	Chchd1	NM_025366.1	
scl014528.1_141-S	252.6571	3.03	Gch1	NM_008102.2	
scl19447.7.1_15-S	251.1485	92.74	2900010J23Rik	NM_175190.2	Mus musculus RIKEN cDNA 2900010J23 gene (2900010J23Rik), mRNA.
scl37771.20_206-S	250.6793	4.07	Agpat3	NM_053014.2	Mus musculus 1-acylglycerol-3-phosphate O-acyltransferase 3 (Agpat3), mRNA.
scl017449.1_1-S	250.4982	23.26	Mdh1	NM_008618.2	Mus musculus malate dehydrogenase 1, NAD (soluble) (Mdh1), mRNA.
scl21187.1.18_12-S	249.5795	5.02	C730025P13Rik	NM_177344	Mus musculus RIKEN cDNA C730025P13 gene (C730025P13Rik), mRNA.
scl078294.3_16-S	247.6458	2.15	Rps27a	NM_024277	Mus musculus ribosomal protein S27a (Rps27a), mRNA.
scl53474.8.1_75-S	247.3119	2.17	Ccs	NM_016892.2	Mus musculus copper chaperone for superoxide dismutase (Ccs), mRNA.
scl48140.10_39-S	247.1328	9.51	Sepp1	NM_009155.2	Mus musculus selenoprotein P, plasma, 1 (Sepp1), mRNA.
scl49219.13_574-S	246.6437	33.97	Osbpl11	NM_176840.2	Mus musculus oxysterol binding protein-like 11 (Osbpl11), mRNA.
scl014828.8_22-S	245.8875	63.2	Hspa5	NM_022310.2	Mus musculus heat shock 70kD protein 5 (glucose-regulated protein) (Hspa5), mRNA.
scl0027050.1_101-S	244.9384	6.01	Rps3	NM_012052.1	Mus musculus ribosomal protein S3 (Rps3), mRNA.
scl0067673.1_322-S	244.8039	2.53	Tceb2	NM_026305.1	Mus musculus transcription elongation factor B (SIII), polypeptide 2 (Tceb2), mRNA.
scl020918.4_11-S	243.7597	6.67	Sui1-rs1	NM_011508	Mus musculus suppressor of initiator codon mutations, related sequence 1 (S. cerevisiae) (Sui1-rs1), mRNA.
scl0378702.3_30-S	242.2302	2.27	Serf2	NM_011354.1	Mus musculus small EDRK-rich factor 2 (Serf2), mRNA.
scl067371.1_2-S	241.8995	29.42	2410016F19Rik	NM_026113.2	Mus musculus RIKEN cDNA 2410016F19 gene (2410016F19Rik), mRNA.
scl0016341.1_91-S	240.4897	29.9	Eif3s6	NM_008388.1	Mus musculus eukaryotic translation initiation factor 3, subunit 6 (Eif3s6), mRNA.
scl51111.10.24_9-S	238.821	2.29	Tcp1	NM_013686.1	Mus musculus t-complex protein 1 (Tcp1), mRNA.
scl000395.1_15-S	237.7921	4.86	Adk	NM_134079	Mus musculus adenosine kinase (Adk), mRNA.
scl012757.9_180-S	237.4521	11.53	Clta	NM_016760.1	Mus musculus clathrin, light polypeptide (Lca) (Clta), mRNA.
scl0380773.4_2-S	236.8167	9.07	1810035L17Rik	XM_354684.1	
scl0026908.1_233-S	236.4382	5.31	Eif2s3y	NM_012011.1	Mus musculus eukaryotic translation initiation factor 2, subunit 3, structural gene Y-linked (Eif2s3y), mRNA.
scl074255.2_27-S	236.1406	65.14	Smu1	NM_021535.2	
scl059048.1_115-S	235.1742	3.98	1500002I11Rik	NM_021550.2	Mus musculus RIKEN cDNA 1500002I11 gene (1500002I11Rik), mRNA.

scl41130.3.1_26-S	235.0674	7.53	1100001G20Rik	NM_183249.1	Mus musculus RIKEN cDNA 1100001G20 gene (1100001G20Rik), mRNA.
scl40580.5.1_111-S	234.5948	2.1	1110020P15Rik	NM_197979.1	Mus musculus RIKEN cDNA 1110020P15 gene (1110020P15Rik), mRNA.
scl067067.1_49-S	233.7093	2.27	2010100O12Rik	NM_025946.2	Mus musculus RIKEN cDNA 2010100O12 gene (2010100O12Rik), mRNA.
scl17096.10_189-S	233.6774	3.11	Bpnt1	NM_011794.2	Mus musculus bisphosphate 3-nucleotidase 1 (Bpnt1), mRNA.
scl00107371.2_186-S	233.452	14.35	Sec15l1	NM_175353.1	
scl017938.7_45-S	233.3289	8.7	Naca	NM_013608.2	Mus musculus nascent polypeptide-associated complex alpha polypeptide (Naca), mRNA.
scl00232449.2_167-S	232.4242	8.63	2500002K03Rik	NM_172733.1	Mus musculus RIKEN cDNA 2500002K03 gene (2500002K03Rik), mRNA.
scl00353208.1_64-S	231.9539	12.55	2810021G02Rik		
scl066609.1_124-S	231.8053	22.06	Cryzl1	NM_133679.1	Mus musculus crystallin, zeta (quinone reductase)-like 1 (Cryzl1), mRNA.
scl36203.11_30-S	231.1927	8.43	4931406C07Rik	NM_133732.1	Mus musculus RIKEN cDNA 4931406C07 gene (4931406C07Rik), mRNA.
scl065019.1_21-S	231.1595	2.08	Rpl23	NM_022891.1	Mus musculus ribosomal protein L23 (Rpl23), mRNA.
scl00320611.1_109-S	230.9861	7.44	9230101K24Rik	NM_177206	Mus musculus RIKEN cDNA 9230101K24 gene (9230101K24Rik), mRNA.
scl020044.5_19-S	230.8979	2.08	Rps14	NM_020600.2	Mus musculus ribosomal protein S14 (Rps14), mRNA.
scl39652.23.1463_24-S	230.3463	17.91	Kpnb1	NM_008379.2	Mus musculus karyopherin (importin) beta 1 (Kpnb1), mRNA.
scl47913.6_530-S	230.2826	6.96	LOC239447	NM_173422.1	Mus musculus collectin liver 1 (LOC239447), mRNA.
scl076654.7_194-S	230.1278	3.24	Upp2	NM_029692.1	Mus musculus uridine phosphorylase 2 (Upp2), mRNA.
scl000154.1_82-S	229.0593	2.91	Bccip	NM_025392	Mus musculus BRCA2 and CDKN1A interacting protein (Bccip), mRNA.
scl32136.14.1_5-S	228.3251	61.86	Bucs1	NM_054094.4	Mus musculus butyryl Coenzyme A synthetase 1 (Bucs1), mRNA.
scl15766.4.1_27-S	227.0694	4.27	1110060M21Rik	NM_025424.1	Mus musculus RIKEN cDNA 1110060M21 gene (1110060M21Rik), mRNA.
scl000397.1_19-S	226.7668	38.18	2400006A19Rik	NM_025550.2	Mus musculus RIKEN cDNA 2400006A19 gene (2400006A19Rik), mRNA.
scl012868.2_102-S	226.6568	2.9	Cox8a	NM_007750.1	Mus musculus cytochrome c oxidase, subunit VIIIa (Cox8a), mRNA.
scl0056307.2_142-S	226.5171	8.95	Metap2	NM_019648.2	Mus musculus methionine aminopeptidase 2 (Metap2), mRNA.
scl53834.2_155-S	226.0363	2.79	Timm8a	NM_013898.1	Mus musculus translocase of inner mitochondrial membrane 8 homolog a (yeast) (Timm8a), mRNA.
scl48956.26_1-S	225.8694	25.7	Usp25	NM_013918.2	Mus musculus ubiquitin specific protease 25 (Usp25), mRNA.
scl019983.8_15-S	225.6989	2.34	Rpl5	NM_016980.1	Mus musculus ribosomal protein L5 (Rpl5), mRNA.
scl17393.13.1_260-S	225.5108	2.51	F13b	NM_031164	Mus musculus coagulation factor XIII, beta subunit (F13b), mRNA.
scl0051810.1_209-S	225.0924	7.91	Hnrpu	NM_016805.1	Mus musculus heterogeneous nuclear ribonucleoprotein U (Hnrpu), mRNA.
scl079555.6_1-S	225.0538	3.9	BC005537	NM_024473.2	Mus musculus cDNA sequence BC005537 (BC005537), mRNA.
scl0019191.1_143-S	224.6878	3.81	Psme2b	NM_011191.1	Mus musculus protease (prosome, macropain) 28 subunit, beta, b (Psme2b), mRNA.
scl012520.7_274-S	224.2821	2.07	Cd81	NM_133655.1	Mus musculus CD 81 antigen (Cd81), mRNA.
scl093739.4_24-S	223.8067	32.78	Gabarapl2	NM_026693.2	Mus musculus GABA(A) receptor-associated protein like 2 (Gabarapl2), mRNA.
scl0068194.2_330-S	223.7576	108.82	Ndufb4	NM_026610.1	Mus musculus NADH dehydrogenase (ubiquinone) 1 beta subcomplex 4 (Ndufb4), mRNA.
scl35560.5_28-S	222.6532	3.38	Cox7a2	NM_009945.2	Mus musculus cytochrome c oxidase, subunit VIIa 2 (Cox7a2), mRNA.
scl0017993.1_168-S	222.3183	2.98	Ndufs4	NM_010887.1	Mus musculus NADH dehydrogenase (ubiquinone) Fe-S protein 4 (Ndufs4), mRNA.
scl0057296.1_256-S	222.0758	5.39	Psmd8	NM_026545.1	Mus musculus proteasome (prosome, macropain) 26S subunit, non-ATPase, 8 (Psmd8), mRNA.
scl42463.7.1_1-S	221.2828	13.55	1810011O16Rik	NM_025456.2	Mus musculus RIKEN cDNA 1810011O16 gene (1810011O16Rik), mRNA.
scl30362.8.2_277-S	221.1585	5.59	Kdt1	NM_175088.2	Mus musculus kidney cell line derived transcript 1 (Kdt1), mRNA.
scl066361.1_72-S	220.625	19.99	2310008M20Rik	NM_025512.2	Mus musculus RIKEN cDNA 2310008M20 gene (2310008M20Rik), mRNA.
scl0232087.1_5-S	220.25	7.71	Mat2a	NM_145569	Mus musculus methionine adenosyltransferase II, alpha (Mat2a), mRNA.
scl39096.7.1_9-S	220.2193	25.07	Aldh8a1	NM_178713.3	Mus musculus aldehyde dehydrogenase 8 family, member A1 (Aldh8a1), mRNA.
scl075617.3_32-S	219.7798	2.05	Rps25	NM_024266	Mus musculus ribosomal protein S25 (Rps25), mRNA.
scl54548.7.1_64-S	219.7003	2.5	Hadh2	NM_016763.1	Mus musculus hydroxyacyl-Coenzyme A dehydrogenase type II (Hadh2), mRNA.
scl27687.10.1_4-S	219.5598	7.22	Paics	NM_025939.2	Mus musculus phosphoribosylaminoimidazole carboxylase, phosphoribosylaminoribosylaminoimidazole, succinocarboxamide synthetase (Paics), mRNA.
scl019384.7_7-S	219.1941	2.19	Ran	NM_009391.2	Mus musculus RAN, member RAS oncogene family (Ran), mRNA.
scl016971.1_277-S	218.5725	34.75	Lrp1	NM_008512.1	Mus musculus low density lipoprotein receptor-related protein 1 (Lrp1), mRNA.
scl0020511.2_152-S	218.0085	4.18	Slc1a2	NM_011393.1	Mus musculus solute carrier family 1 (glial high affinity glutamate transporter), member 2 (Slc1a2), mRNA.
scl0013171.2_124-S	217.9673	25.74	Dbt	NM_010022.1	Mus musculus dihydrolipoamide branched chain transacylase E2 (Dbt), mRNA.
scl17803.10.1_30-S	216.3663	3.62	Arpc2	XM_129773.5	Mus musculus actin related protein 2/3 complex, subunit 2 (Arpc2), mRNA.
scl0050790.1_147-S	216.2081	17.52	Acsl4	NM_207625.1	Mus musculus acyl-CoA synthetase long-chain family member 4 (Acsl4), transcript variant 2, mRNA.
scl019068.1_323-S	215.5632	2.14	Erh	NM_007951.1	Mus musculus enhancer of rudimentary homolog (Drosophila) (Erh), mRNA.
scl41579.7_169-S	215.358	2.08	Ppp2ca	NM_019411.2	Mus musculus protein phosphatase 2a, catalytic subunit, alpha isoform (Ppp2ca), mRNA.
scl069178.1_5-S	214.8096	30.15	Snx5	NM_024225.2	Mus musculus sorting nexin 5 (Snx5), mRNA.
scl0002306.1_28-S	214.6447	18.84		XM_283061.1	Mus musculus RIKEN cDNA A630086P08 gene (A630086P08Rik), mRNA.
scl41251.8_435-S	214.5173	37.32	Ywhae	NM_009536.2	Mus musculus tyrosine 3-monooxygenase/tryptophan 5-monooxygenase activation protein, epsilon polypeptide (Ywhae), mRNA.
scl067869.4_264-S	212.568	12.01	Paip2	NM_026420.1	Mus musculus polyadenylate-binding protein-interacting protein 2 (Paip2), mRNA.
scl16633.10.1_180-S	212.461	9.87	Pecr	NM_023523.3	Mus musculus peroxisomal trans-2-enoyl-CoA reductase (Pecr), mRNA.

scl31237.29_375-S	212.4424	28.29	Tjp1	NM_009386.1	Mus musculus tight junction protein 1 (Tjp1), mRNA.
scl24338.5.1_130-S	211.8285	10.24	Baat	NM_007519	Mus musculus bile acid-Coenzyme A: amino acid N-acyltransferase (Baat), mRNA.
scl019933.5_3-S	211.6415	27.36	Rpl21	NM_019647.3	Mus musculus ribosomal protein L21 (Rpl21), mRNA.
scl29623.5_373-S	211.578	27.78	Vhlh	NM_009507	Mus musculus von Hippel-Lindau syndrome homolog (Vhlh), mRNA.
scl000794.1_5-S	211.5096	8.59	Tsn	NM_011650.2	Mus musculus translin (Tsn), mRNA.
scl42333.3_174-S	211.0549	4.95	Sgpp1	NM_030750.2	Mus musculus sphingosine-1-phosphate phosphatase 1 (Sgpp1), mRNA.
scl056771.4_27-S	210.7219	30.42	Usp49	NM_020048.2	Mus musculus ubiquitin specific protease 49 (Usp49), mRNA.
scl23219.3_221-S	209.8	5	Rab33b	NM_016858.1	Mus musculus RAB33B, member of RAS oncogene family (Rab33b), mRNA.
scl067912.2_23-S	209.6971	3.41	1600012H06Rik	NM_026451.1	Mus musculus RIKEN cDNA 1600012H06 gene (1600012H06Rik), mRNA.
scl47100.1.18_67-S	209.3599	5.25	Ptplb	NM_023587	Mus musculus protein tyrosine phosphatase-like (proline instead of catalytic arginine), member b (Ptplb), mRNA.
scl019941.2_3-S	209.2068	3.03	Rpl26	NM_009080	Mus musculus ribosomal protein L26 (Rpl26), mRNA.
scl0054683.2_242-S	208.4302	2.79	Prdx5	NM_012021.1	Mus musculus peroxiredoxin 5 (Prdx5), mRNA.
scl068052.3_30-S	208.2953	2.12	Rps13	NM_026533	Mus musculus ribosomal protein S13 (Rps13), mRNA.
scl0021761.2_320-S	208.0904	23.59	Morf4l1	NM_024431	Mus musculus mortality factor 4 like 1 (Morf4l1), mRNA.
scl012867.3_17-S	208.0499	2.11	Cox7c	NM_007749.1	Mus musculus cytochrome c oxidase, subunit VIIc (Cox7c), mRNA.
scl26699.18.1_4-S	207.9101	6.08	2610033H07Rik	NM_029278.1	Mus musculus RIKEN cDNA 2610033H07 gene (2610033H07Rik), mRNA.
scl38976.4.1_15-S	207.5169	12.6	Snx3	NM_017472.2	Mus musculus sorting nexin 3 (Snx3), mRNA.
scl018458.3_17-S	207.4557	3.38	Pabpc1	NM_008774.2	Mus musculus poly A binding protein, cytoplasmic 1 (Pabpc1), mRNA.
scl19885.7_41-S	206.8535	4.03	Zfp313	NM_030743.3	Mus musculus zinc finger protein 313 (Zfp313), mRNA.
scl056456.15_85-S	206.8049	8.86	Actl6a	NM_019673.1	
scl47786.6.1_30-S	205.8073	4.5	2310010G13Rik	NM_027122.2	Mus musculus RIKEN cDNA 2310010G13 gene (2310010G13Rik), mRNA.
scl25301.1.41_0-S	205.582	7.8	Rraga	NM_178376.2	Mus musculus Ras-related GTP binding A (Rraga), mRNA.
scl35945.10_223-S	205.4275	8.28	Arcn1	NM_145985.2	Mus musculus archain 1 (Arcn1), mRNA.
scl20689.4.1_4-S	205.2742	2.85	Timm13a	NM_013899.1	Mus musculus translocase of inner mitochondrial membrane 13 homolog a (yeast) (Timm13a), mRNA.
scl0018534.2_230-S	203.897	15.43	Pck1	NM_011044.1	Mus musculus phosphoenolpyruvate carboxykinase 1, cytosolic (Pck1), mRNA.
scl53077.5.8_20-S	203.6767	2.17	Gsto1	NM_010362.1	Mus musculus glutathione S-transferase omega 1 (Gsto1), mRNA.
scl0003437.1_0-S	203.5309	1.97	Rplp1	NM_018853.1	Mus musculus ribosomal protein, large, P1 (Rplp1), mRNA.
scl54996.3_58-S	203.3252	7.2	Pgrmc1	NM_016783.2	Mus musculus progesterone receptor membrane component 1 (Pgrmc1), mRNA.
scl017991.2_1-S	203.2114	2	Ndufa2	NM_010885.2	Mus musculus NADH dehydrogenase (ubiquinone) 1 alpha subcomplex, 2 (Ndufa2), mRNA.
scl51664.18.1_87-S	202.856	6.24	Usp14	NM_021522.2	Mus musculus ubiquitin specific protease 14 (Usp14), mRNA.
scl50010.18.1_105-S	202.1028	7.48	H2-Bf	NM_008198.1	Mus musculus histocompatibility 2, complement component factor B (H2-Bf), mRNA.
scl056748.8_105-S	201.5013	7.08	Hirip5	NM_020045.1	Mus musculus histone cell cycle regulation defective interacting protein 5 (Hirip5), mRNA.
scl38589.13.1_14-S	201.3432	5.83	Pah	NM_008777.1	Mus musculus phenylalanine hydroxylase (Pah), mRNA.
scl16469.4.1_83-S	198.9714	2.04	1110002M09Rik	XM_129957.1	Mus musculus RIKEN cDNA 1110002M09 gene (1110002M09Rik), mRNA.
scl056043.1_7-S	198.7379	9.6	Akr1e1	NM_018859.1	Mus musculus aldo-keto reductase family 1, member E1 (Akr1e1), mRNA.
scl067106.7_0-S	198.7334	57.89	Arch	NM_025970.1	Mus musculus archease (Arch), mRNA.
scl0015040.1_90-S	198.1948	2.61	H2-T23	NM_010398.1	Mus musculus histocompatibility 2, T region locus 23 (H2-T23), mRNA.
scl20479.1.60_10-S	198.0971	2.66	Nola3	NM_025403.1	Mus musculus nucleolar protein family A, member 3 (Nola3), mRNA.
scl0066671.2_25-S	197.9092	3.69	Ccnh	NM_023243.2	Mus musculus cyclin H (Ccnh), mRNA.
scl054127.2_67-S	197.4222	2.54	Rps28	NM_016844.1	Mus musculus ribosomal protein S28 (Rps28), mRNA.
scl056438.5_27-S	197.3712	2.58	Rbx1	NM_019712.2	Mus musculus ring-box 1 (Rbx1), mRNA.
scl55022.8.1_12-S	197.3585	53.6	Rgn	NM_009060.1	Mus musculus regucalcin (Rgn), mRNA.
scl017274.8_92-S	196.7946	26.14	Rab8a	NM_023126.2	Mus musculus RAB8A, member RAS oncogene family (Rab8a), mRNA.
scl00320541.1_138-S	196.4998	7.84	A530082C11Rik	NM_177186.3	Mus musculus RIKEN cDNA A530082C11 gene (A530082C11Rik), mRNA.
scl17443.8_550-S	195.4851	3.89	Arl10b	NM_026823.1	
scl22593.9.1_1-S	194.9876	5.02	1110013G13Rik	NM_146141	Mus musculus RIKEN cDNA 1110013G13 gene (1110013G13Rik), mRNA.
scl014227.3_23-S	194.6484	2.14	Fkbp2	NM_008020.2	Mus musculus FK506 binding protein 2 (Fkbp2), mRNA.
scl47326.13.2_27-S	193.9876	118.71	Cct5	NM_007637.1	Mus musculus chaperonin subunit 5 (epsilon) (Cct5), mRNA.
scl012385.18_225-S	193.8731	2	Catna1	NM_009818.1	Mus musculus catenin alpha 1 (Catna1), mRNA.
scl46570.12.1_20-S	193.6189	2.36	Vdac2	NM_011695.1	Mus musculus voltage-dependent anion channel 2 (Vdac2), mRNA.
scl013681.1_1-S	193.1396	2.23	Eif4a1	NM_144958.2	Mus musculus eukaryotic translation initiation factor 4A1 (Eif4a1), mRNA.
scl39022.13.1_2-S	193.115	3.56	Hdac2	NM_008229.1	Mus musculus histone deacetylase 2 (Hdac2), mRNA.
scl34532.9_187-S	192.7618	4.57	Dnaja2	NM_019794.1	Mus musculus DnaJ (Hsp40) homolog, subfamily A, member 2 (Dnaja2), mRNA.
scl012583.1_32-S	192.7073	51.66	Cdo1	NM_033037.2	Mus musculus cysteine dioxygenase 1, cytosolic (Cdo1), mRNA.
scl24787.5_386-S	192.2453	17.49	0610009K11Rik	NM_026689.3	Mus musculus RIKEN cDNA 0610009K11 gene (0610009K11Rik), mRNA.
scl0237898.1_282-S	191.4813	5.2	6430526O11Rik	XM_110937.4	

scl0245867.4_1-S	191.0705	8.15	5330414D10Rik	NM_153594.2	Mus musculus RIKEN cDNA 5330414D10 gene (5330414D10Rik), mRNA.
scl068908.1_119-S	190.869	4.55	9130005N14Rik	NM_026667.2	Mus musculus RIKEN cDNA 9130005N14 gene (9130005N14Rik), mRNA.
scl057294.3_33-S	190.6965	2.58	Rps27	NM_027015.1	Mus musculus ribosomal protein S27 (Rps27), mRNA.
scl0067171.2_285-S	190.2539	4.3	2610318G18Rik	NM_026013.1	Mus musculus RIKEN cDNA 2610318G18 gene (2610318G18Rik), mRNA.
scl33694.4.1_117-S	190.0098	2.05	Pgls	NM_025396.1	Mus musculus 6-phosphogluconolactonase (Pgls), mRNA.
scl056398.7_324-S	189.9005	3.35	1500003O03Rik	NM_019769.2	Mus musculus RIKEN cDNA 1500003O03 gene (1500003O03Rik), mRNA.
scl23150.4.1_11-S	189.8021	36.55	Aadac	NM_023383.1	Mus musculus arylacetamide deacetylase (esterase) (Aadac), mRNA.
scl0067096.2_233-S	189.7091	6.14	1810037K07Rik	NM_025962.1	Mus musculus RIKEN cDNA 1810037K07 gene (1810037K07Rik), mRNA.
scl00229096.1_119-S	189.6624	4.26	Ythdf3	NM_172677.2	
scl22677.22.1_1-S	189.6522	6.77	Dpyd	NM_170778.1	Mus musculus dihydropyrimidine dehydrogenase (Dpyd), mRNA.
scl0002359.1_0-S	188.7937	39.39	Pbef1	NM_021524.1	Mus musculus pre-B-cell colony-enhancing factor 1 (Pbef1), mRNA.
scl48200.7.1_9-S	188.2422	1.93	Atp5o	NM_138597	Mus musculus ATP synthase, H+ transporting, mitochondrial F1 complex, O subunit (Atp5o), mRNA.
scl022327.6_246-S	188.2009	5.21	Vbp1	NM_011692.1	Mus musculus von Hippel-Lindau binding protein 1 (Vbp1), mRNA.
scl30202.10.1_68-S	187.0585	24.71	Akr1d1	NM_145364.1	Mus musculus aldo-keto reductase family 1, member D1 (Akr1d1), mRNA.
scl0004148.1_17-S	187.0092	22.95	Lias	NM_024471.2	Mus musculus lipoic acid synthetase (Lias), mRNA.
scl31910.9.1_52-S	186.2139	1.99	Cyp2e1	NM_021282.1	Mus musculus cytochrome P450, family 2, subfamily e, polypeptide 1 (Cyp2e1), mRNA.
scl0109077.2_328-S	185.9648	3.9	1110055N21Rik	NM_176843	Mus musculus RIKEN cDNA 1110055N21 gene (1110055N21Rik), mRNA.
scl29636.20.1_18-S	185.5126	3.56	1110061O04Rik	NM_026849	Mus musculus RIKEN cDNA 1110061O04 gene (1110061O04Rik), mRNA.
scl0015473.1_93-S	184.8444	18.35	Hrsp12	NM_008287.2	Mus musculus heat-responsive protein 12 (Hrsp12), mRNA.
scl47892.24_470-S	184.3647	4.89	D15Ertd621e	NM_145959.1	
scl0001436.1_98-S	183.7651	4.36	Commd1	NM_144514.1	Mus musculus COMM domain containing 1 (Commd1), mRNA.
scl068730.1_55-S	183.4534	2.47	1110032N12Rik	NM_026824.2	Mus musculus RIKEN cDNA 1110032N12 gene (1110032N12Rik), mRNA.
scl36630.14.1_50-S	182.8342	2.08	Ctsh	NM_007801	Mus musculus cathepsin H (Ctsh), mRNA.
scl0003600.1_3-S	182.7859	1.9	MGC18837	NM_178577.3	Mus musculus hypothetical protein MGC18837 (MGC18837), mRNA.
scl074150.16_288-S	182.7312	16.29	Slc35f5	NM_028787.2	Mus musculus solute carrier family 35, member F5 (Slc35f5), mRNA.
scl0001621.1_13-S	182.7075	7.88	Tcp1	NM_013686	Mus musculus t-complex protein 1 (Tcp1), mRNA.
scl021357.9_262-S	182.6836	3.45	Tarbp2	NM_009319	Mus musculus TAR (HIV) RNA binding protein 2 (Tarbp2), mRNA.
scl00192657.1_320-S	182.3967	8.27	Ell2	NM_138953.1	Mus musculus elongation factor RNA polymerase II 2 (Ell2), mRNA.
scl19131.5.1_9-S	182.158	1.88	Atp5g3	NM_175015.1	Mus musculus ATP synthase, H+ transporting, mitochondrial F0 complex, subunit c (subunit 9), isoform 3 (Atp5g3), mRNA.
scl43593.17_243-S	181.6858	25.39	Rad17	NM_011233.1	Mus musculus RAD17 homolog (S. pombe) (Rad17), mRNA.
scl0056347.2_235-S	181.4383	7.94	Eif3s8	NM_019646.1	Mus musculus eukaryotic translation initiation factor 3, subunit 8 (Eif3s8), mRNA.
scl015384.1_1-S	181.3234	1.95	Hnrpab	NM_010448.2	Mus musculus heterogeneous nuclear ribonucleoprotein A/B (Hnrpab), mRNA.
scl25927.13_1-S	181.1365	16.26	Pom121	NM_148932.1	Mus musculus nuclear pore membrane protein 121 (Pom121), mRNA.
scl015528.3_20-S	180.7726	11.87	Hspe1	NM_008303.2	Mus musculus heat shock protein 1 (chaperonin 10) (Hspe1), mRNA.
scl0099730.2_131-S	180.6381	6.46	Taf13	NM_025444.1	Mus musculus TAF13 RNA polymerase II, TATA box binding protein (TBP)-associated factor (Taf13), mRNA.
scl47755.2_19-S	178.7951	29.12	H1f0	NM_008197.2	Mus musculus H1 histone family, member 0 (H1f0), mRNA.
scl40782.12.1_32-S	178.2747	11.15	Apoh	NM_013475.1	Mus musculus apolipoprotein H (Apoh), mRNA.
scl014897.1_29-S	177.9799	60.2	Trip12	NM_133975.2	
scl39554.8_358-S	177.4547	2.69	Rab5c	NM_024456	Mus musculus RAB5C, member RAS oncogene family (Rab5c), mRNA.
scl0017936.1_74-S	177.45	7.65	Nab1	NM_008667.2	Mus musculus Ngfi-A binding protein 1 (Nab1), mRNA.
scl52400.3.8_10-S	176.9056	13.88	Arl3	NM_019718.2	Mus musculus ADP-ribosylation factor-like 3 (Arl3), mRNA.
scl056720.1_140-S	176.5828	38.97	Tdo2	NM_019911.2	Mus musculus tryptophan 2,3-dioxygenase (Tdo2), mRNA.
scl067440.9_18-S	176.1384	6.6	Papd1	NM_026157.1	Mus musculus PAP associated domain containing 1 (Papd1), mRNA.
scl094061.9_0-S	176.0607	11.68	Mrpl1	NM_053158.1	Mus musculus mitochondrial ribosomal protein L1 (Mrpl1), mRNA.
scl25574.8.1_15-S	175.2792	8.88	Ube2j1	NM_019586.2	Mus musculus ubiquitin-conjugating enzyme E2, J1 (Ube2j1), mRNA.
scl066489.3_56-S	174.9443	1.88	Rpl35	NM_025592	Mus musculus ribosomal protein L35 (Rpl35), mRNA.
scl0394430.5_296-S	174.7052	28.12	Ugt1a13	NM_201641	
scl51004.4_55-S	174.319	5.91	Sepx1	NM_013759.1	
scl0268470.1_0-S	174.1059	32.41	D11Moh35	NM_172300	Mus musculus DNA segment, Chr 11, KL Mohlke 35 (D11Moh35), mRNA.
scl0216705.9_38-S	173.6347	23.93	AI642036	XM_109767.2	Mus musculus expressed sequence AI642036 (AI642036), mRNA.
scl54584.7_603-S	173.0252	2.5	Prps1	NM_021463.2	Mus musculus phosphoribosyl pyrophosphate synthetase 1 (Prps1), mRNA.
scl018102.1_15-S	172.9593	1.93	Nme1	NM_008704.2	Mus musculus expressed in non-metastatic cells 1, protein (Nme1), mRNA.
scl53400.8.1_51-S	172.1667	2.79	D19Ertd721e	NM_146093.1	Mus musculus DNA segment, Chr 19, ERATO Doi 721, expressed (D19Ertd721e), mRNA.
scl0064291.1_176-S	172.1151	8.73	Osbpl1a	NM_020573.1	Mus musculus oxysterol binding protein-like 1A (Osbpl1a), mRNA.
scl0002589.1_2-S	171.9854	2.18	Eef1d	NM_023240.1	Mus musculus eukaryotic translation elongation factor 1 delta (guanine nucleotide exchange protein) (Eef1d), mRNA.
scl54871.18_531-S	171.9159	4.74	Mtmr1	NM_016985.1	Mus musculus myotubularin related protein 1 (Mtmr1), mRNA.

scl0066881.2_144-S	171.8714	82.86	Pcyox1	NM_025823.3	Mus musculus prenylcysteine oxidase 1 (Pcyox1), mRNA.
scl51737.13.1_28-S	171.621	24.73	Atp5a1	NM_007505.1	Mus musculus ATP synthase, H+ transporting, mitochondrial F1 complex, alpha subunit, isoform 1 (Atp5a1), mRNA.
scl30187.2.1_182-S	171.1127	1.91	1110001J03Rik	NM_025363.2	Mus musculus RIKEN cDNA 1110001J03 gene (1110001J03Rik), mRNA.
scl37617.5_6-S	171.0719	2.59	Slc25a3	NM_133668.2	Mus musculus solute carrier family 25 (mitochondrial carrier; phosphate carrier), member 3 (Slc25a3), mRNA.
scl32898.7.1_80-S	170.9051	3.51	Blvrb	NM_144923	Mus musculus biliverdin reductase B (flavin reductase (NADPH)) (Blvrb), mRNA.
scl54148.25_5-S	170.5107	4.45	Hcfc1	NM_008224.2	Mus musculus host cell factor C1 (Hcfc1), mRNA.
scl42971.1_9-S	170.5074	47.24	0710001C05Rik	XM_203592.1	Mus musculus RIKEN cDNA 0710001C05 gene (0710001C05Rik), mRNA.
scl018744.1_4-S	170.1919	2.62	Pja1	NM_008853.1	Mus musculus praja1, RING-H2 motif containing (Pja1), mRNA.
scl019175.6_47-S	169.5758	1.87	Psmb6	NM_008946	Mus musculus proteasome (prosome, macropain) subunit, beta type 6 (Psmb6), mRNA.
scl47063.13.1_72-S	169.5622	6.39	Top1mt	XM_128145.2	
scl057423.2_11-S	169.5512	1.88	Atp5j2	NM_020582.1	Mus musculus ATP synthase, H+ transporting, mitochondrial F0 complex, subunit f, isoform 2 (Atp5j2), mRNA.
scl056351.11_60-S	169.4939	23.04	Tebp	NM_019766.2	Mus musculus telomerase binding protein, p23 (Tebp), mRNA.
scl0002356.1_173-S	169.3857	2.95	Sypl	NM_198710.1	Mus musculus synaptophysin-like protein (Sypl), transcript variant 1, mRNA.
scl31959.7.1_44-S	169.138	1.98	Bccip	NM_025392.1	Mus musculus BRCA2 and CDKN1A interacting protein (Bccip), mRNA.
scl022245.1_319-S	168.8761	3.14	Uck1	NM_011675.1	
scl019047.11_32-S	168.3982	39.1	Ppp1cc	NM_013636.2	Mus musculus protein phosphatase 1, catalytic subunit, gamma isoform (Ppp1cc), mRNA.
scl030057.2_1-S	167.0058	1.94	Timm8b	NM_013897.1	Mus musculus translocase of inner mitochondrial membrane 8 homolog b (yeast) (Timm8b), mRNA.
scl015024.2_236-S	166.7603	8.48	H2-T10	NM_010395.2	Mus musculus histocompatibility 2, T region locus 10 (H2-T10), mRNA.
scl070544.3_53-S	166.655	2.31	5730437N04Rik	NM_027457.2	Mus musculus RIKEN cDNA 5730437N04 gene (5730437N04Rik), mRNA.
scl011668.12_94-S	165.9671	5.88	Aldh1a1	NM_013467	Mus musculus aldehyde dehydrogenase family 1, subfamily A1 (Aldh1a1), mRNA.
scl020055.2_9-S	165.8618	1.84	Rps16	NM_013647.1	Mus musculus ribosomal protein S16 (Rps16), mRNA.
scl020729.4_276-S	165.5654	16.92	Spin	NM_011462	Mus musculus spindlin (Spin), mRNA.
scl0019246.2_230-S	165.3762	7.54	Ptpn1	NM_011201.1	Mus musculus protein tyrosine phosphatase, non-receptor type 1 (Ptpn1), mRNA.
scl0002748.1_8-S	165.3067	1.97	1110060F11Rik	NM_026395.1	Mus musculus RIKEN cDNA 1110060F11 gene (1110060F11Rik), mRNA.
scl066477.2_23-S	165.2128	132.35	Usmg5	XM_123431.1	Mus musculus upregulated during skeletal muscle growth 5 (Usmg5), mRNA.
scl32464.22.1_107-S	165.1463	2.77	Pde8a	NM_008803.1	Mus musculus phosphodiesterase 8A (Pde8a), mRNA.
scl015374.1_66-S	164.9824	5.93	Hn1	NM_008258.1	Mus musculus hematological and neurological expressed sequence 1 (Hn1), mRNA.
scl064657.6_146-S	164.9008	5.39	Mrps10	NM_183086	Mus musculus mitochondrial ribosomal protein S10 (Mrps10), mRNA.
scl0001079.1_24-S	164.7236	3.25	Vamp8	NM_016794.2	Mus musculus vesicle-associated membrane protein 8 (Vamp8), mRNA.
scl21503.2.1_16-S	164.5607	6.14	Hadhsc	NM_008212.1	Mus musculus L-3-hydroxyacyl-Coenzyme A dehydrogenase, short chain (Hadhsc), mRNA.
scl020090.2_29-S	164.4984	1.81	Rps29	NM_009093.1	Mus musculus ribosomal protein S29 (Rps29), mRNA.
scl28571.9_1-S	164.4616	27.86	Sumf1	NM_145937.1	
scl027370.3_85-S	164.3755	1.83	Rps26	NM_013765	Mus musculus ribosomal protein S26 (Rps26), mRNA.
scl0004113.1_27-S	164.2819	1.9	Mrpl33	NM_025796.1	Mus musculus mitochondrial ribosomal protein L33 (Mrpl33), mRNA.
scl48128.6_4-S	164.1115	28.54	2900001O04Rik	NM_026213.2	Mus musculus RIKEN cDNA 2900001O04 gene (2900001O04Rik), mRNA.
scl50229.6.1_24-S	164.1044	2.04	Tceb2	NM_026305.1	Mus musculus transcription elongation factor B (SIII), polypeptide 2 (Tceb2), mRNA.
scl54331.2.4_4-S	163.7939	1.85	Ndufa1	NM_019443.1	Mus musculus NADH dehydrogenase (ubiquinone) 1 alpha subcomplex, 1 (Ndufa1), mRNA.
scl022666.6_141-S	163.7727	14.56	Zfp161	NM_009547.2	Mus musculus zinc finger protein 161 (Zfp161), mRNA.
scl066680.1_207-S	163.6089	40.67	3230401D17Rik	NM_025699.2	Mus musculus RIKEN cDNA 3230401D17 gene (3230401D17Rik), mRNA.
scl056298.1_121-S	163.4343	12.79	Arl6ip2	NM_178050.2	Mus musculus ADP-ribosylation factor-like 6 interacting protein 2 (Arl6ip2), mRNA.
scl022630.1_91-S	163.2347	3.99	Ywhaq	NM_011739	Mus musculus tyrosine 3-monooxygenase/tryptophan 5-monooxygenase activation protein, theta polypeptide (Ywhaq), mRNA.
scl012861.2_54-S	163.1149	2.18	Cox6a1	NM_007748.2	Mus musculus cytochrome c oxidase, subunit VI a, polypeptide 1 (Cox6a1), mRNA.
scl054381.6_56-S	163.084	2.99	Pgcp	NM_176073.2	Mus musculus plasma glutamate carboxypeptidase (Pgcp), mRNA.
scl38900.29.1_38-S	162.8649	7.59	Ranbp2	NM_011240.2	Mus musculus RAN binding protein 2 (Ranbp2), mRNA.
scl066218.3_27-S	162.1077	24.38	Ndufb9	NM_023172.2	Mus musculus NADH dehydrogenase (ubiquinone) 1 beta subcomplex, 9 (Ndufb9), mRNA.
scl23508.1.1_311-S	161.8813	3.89	Kif1b	NM_207682.1	Mus musculus kinesin family member 1B (Kif1b), transcript variant 1, mRNA.
scl0011797.2_322-S	161.7823	25.81	Birc2	NM_007465.1	Mus musculus baculoviral IAP repeat-containing 2 (Birc2), mRNA.
scl013204.1_302-S	161.181	3.05	Dhx15	NM_007839.1	Mus musculus DEAH (Asp-Glu-Ala-His) box polypeptide 15 (Dhx15), mRNA.
scl53453.19_390-S	161.173	23.71	Adrbk1	NM_130863	Mus musculus adrenergic receptor kinase, beta 1 (Adrbk1), mRNA.
scl017904.3_16-S	161.1016	2.75	Myl6	NM_010860	Mus musculus myosin, light polypeptide 6, alkali, smooth muscle and non-muscle (Myl6), mRNA.
scl0192174.7_133-S	160.3723	22.28	BC016198	NM_203507.1	Mus musculus cDNA sequence BC016198 (BC016198), mRNA.
scl0003824.1_1258-S	160.354	2.17	Igf1	NM_184052.1	Mus musculus insulin-like growth factor 1 (Igf1), mRNA.
scl0001148.1_50-S	159.8422	4.52	Ing4	NM_133345.1	Mus musculus inhibitor of growth family, member 4 (Ing4), mRNA.
scl020104.1_26-S	159.5483	56.94	Rps6	NM_009096.1	Mus musculus ribosomal protein S6 (Rps6), mRNA.
scl0002537.1_7-S	159.3891	2.18	Pfdn5	NM_020031.1	Mus musculus prefoldin 5 (Pfdn5), mRNA.
scl017330.5_48-S	159.163	3.7	Minpp1	NM_010799.1	Mus musculus multiple inositol polyphosphate histidine phosphatase 1 (Minpp1), mRNA.

scl00213541.2_318-S	159.094	7.89	Ythdf2	NM_145393.2	
scl20381.3.1_57-S	158.5169	1.87	2310003F16Rik	NM_026318.1	Mus musculus RIKEN cDNA 2310003F16 gene (2310003F16Rik), mRNA.
scl23736.8.1_149-S	157.8082	65.31	1110007F05Rik	XM_355561.1	Mus musculus RIKEN cDNA 1110007F05 gene (1110007F05Rik), mRNA.
scl066046.6_22-S	157.6024	19.32	Ndufb5	NM_025316.2	Mus musculus NADH dehydrogenase (ubiquinone) 1 beta subcomplex, 5 (Ndufb5), mRNA.
scl26992.46_0-S	157.3337	29.59	AI481500	NM_133901.1	Mus musculus expressed sequence AI481500 (AI481500), mRNA.
scl44322.14.1_14-S	157.1661	4.86	4833420G17Rik	NM_026127.3	Mus musculus RIKEN cDNA 4833420G17 gene (4833420G17Rik), mRNA.
scl020102.3_1-S	157.0176	2.16	Rps4x	NM_009094	Mus musculus ribosomal protein S4, X-linked (Rps4x), mRNA.
scl29755.18.1_293-S	156.7379	2.93	BC022133	NM_144940.1	Mus musculus cDNA sequence BC022133 (BC022133), mRNA.
scl0059042.1_160-S	156.6568	2.03	Cope	NM_021538.1	Mus musculus coatomer protein complex, subunit epsilon (Cope), mRNA.
scl056724.5_33-S	156.4728	4.7	1200020A08Rik	NM_019936.1	Mus musculus RIKEN cDNA 1200020A08 gene (1200020A08Rik), mRNA.
scl26009.10.1_18-S	155.824	4.19	Slc15a4	NM_133895.1	Mus musculus solute carrier family 15, member 4 (Slc15a4), mRNA.
scl0094221.1_20-S	155.7843	16.76	Gopc	NM_053187.2	Mus musculus golgi associated PDZ and coiled-coil motif containing (Gopc), mRNA.
scl093684.5_156-S	155.6032	51.6	39340	NM_053102.1	Mus musculus selenoprotein (Sep15), mRNA.
scl24858.5.1_216-S	155.1856	4.97	AI838661	NM_133884.1	Mus musculus expressed sequence AI838661 (AI838661), mRNA.
scl41358.1_245-S	155.0683	3.71	1810027O10Rik	XM_109683.4	Mus musculus RIKEN cDNA 1810027O10 gene (1810027O10Rik), mRNA.
scl0067549.1_327-S	154.4746	14.13	Gpr89	NM_026229.1	
scl0231086.16_205-S	154.3099	2.46	Hadhb	NM_145558.1	Mus musculus hydroxyacyl-Coenzyme A dehydrogenase/3-ketoacyl-Coenzyme A thiolase/enoyl-Coenzyme A hydratase (trifunctional protein), beta subunit (Hadhb), mRNA.
scl53954.7_10-S	154.2499	24.1	Slc16a2	NM_009197.1	Mus musculus solute carrier family 16 (monocarboxylic acid transporters), member 2 (Slc16a2), mRNA.
scl019921.5_131-S	154.1346	1.78	Rpl19	NM_009078	Mus musculus ribosomal protein L19 (Rpl19), mRNA.
scl0353187.1_56-S	154.112	33.02	Nr1d2	NM_011584.2	Mus musculus nuclear receptor subfamily 1, group D, member 2 (Nr1d2), mRNA.
scl069786.2_30-S	152.9442	46.35	1810034M08Rik	NM_176842.2	Mus musculus RIKEN cDNA 1810034M08 gene (1810034M08Rik), mRNA.
scl0051788.1_274-S	152.4905	1.86	H2afz	NM_016750.1	Mus musculus H2A histone family, member Z (H2afz), mRNA.
scl0110960.1_77-S	152.4871	6.79	Tars	NM_033074.2	Mus musculus threonyl-tRNA synthetase (Tars), mRNA.
scl067414.12_0-S	152.437	1.9	Mfn1	NM_024200.2	Mus musculus mitofusin 1 (Mfn1), mRNA.
scl0020704.1_175-S	152.4283	9.15	Serpina1e	NM_009247.1	Mus musculus serine (or cysteine) proteinase inhibitor, clade A, member 1e (Serpina1e), mRNA.
scl24344.15.1_2-S	152.393	10.49	Txndc4	NM_029572.1	Mus musculus thioredoxin domain containing 4 (endoplasmic reticulum) (Txndc4), mRNA.
scl018103.3_14-S	152.3898	1.79	Nme2	NM_008705.3	Mus musculus expressed in non-metastatic cells 2, protein (Nme2), mRNA.
scl026901.3_41-S	151.885	1.99	Deb1	NM_026794.2	Mus musculus differentially expressed in B16F10 1 (Deb1), mRNA.
scl0030928.2_140-S	151.5433	6.21	Zfp238	NM_013915.1	Mus musculus zinc finger protein 238 (Zfp238), mRNA.
scl000389.1_0-S	150.664	3.27	Ebpl	NM_026598.1	Mus musculus emopamil binding protein-like (Ebpl), mRNA.
scl064660.1_30-S	150.61	1.78	Mrps24	NM_026080.1	Mus musculus mitochondrial ribosomal protein S24 (Mrps24), mRNA.
scl48261.13.240_30-S	150.2811	2.31	Cct8	NM_009840.2	Mus musculus chaperonin subunit 8 (theta) (Cct8), mRNA.
scl097820.1_227-S	150.1602	2.25	4833439L19Rik	NM_133797	Mus musculus RIKEN cDNA 4833439L19 gene (4833439L19Rik), mRNA.
scl29381.10.1_63-S	149.5917	2.54	Cmas	NM_009908.1	Mus musculus cytidine monophospho-N-acetylneuraminic acid synthetase (Cmas), mRNA.
scl00243382.2_254-S	149.3607	3.91	A930026L03Rik	NM_175523.2	Mus musculus RIKEN cDNA A930026L03 gene (A930026L03Rik), mRNA.
scl020973.4_189-S	149.1647	3.97	Syngr2	NM_009304.1	Mus musculus synaptogyrin 2 (Syngr2), mRNA.
scl15890.11.1_28-S	148.9019	14.25	Fh1	NM_010209	Mus musculus fumarate hydratase 1 (Fh1), mRNA.
scl0230789.1_243-S	148.7926	2.88	BC008163	NM_145553.1	Mus musculus cDNA sequence BC008163 (BC008163), mRNA.
scl40538.6.1_45-S	148.5656	2.32	H2afv	XM_126043.3	
scl0003853.1_11-S	148.0312	13.88	Lta4h	NM_008517.1	Mus musculus leukotriene A4 hydrolase (Lta4h), mRNA.
scl067281.3_0-S	147.7694	1.75	Rpl37	NM_026069.1	Mus musculus ribosomal protein L37 (Rpl37), mRNA.
scl36624.10.1_0-S	147.5892	5.66	Plscr2	NM_008880.2	Mus musculus phospholipid scramblase 2 (Plscr2), mRNA.
scl0020832.1_299-S	147.3481	1.79	Ssr4	NM_009279.1	Mus musculus signal sequence receptor, delta (Ssr4), mRNA.
scl0107141.5_21-S	147.1811	43.35	Cyp2c50	NM_134144	Mus musculus cytochrome P450, family 2, subfamily c, polypeptide 50 (Cyp2c50), mRNA.
scl0054673.1_296-S	147.0777	21.99	Sh3glb1	NM_019464.1	Mus musculus SH3-domain GRB2-like B1 (endophilin) (Sh3glb1), mRNA.
scl50698.5_378-S	146.8267	10.73	Enpp5	NM_032003.1	Mus musculus ectonucleotide pyrophosphatase/phosphodiesterase 5 (Enpp5), mRNA.
scl38024.23.1_253-S	146.8111	12.16	A530089I17Rik	NM_133999.1	Mus musculus RIKEN cDNA A530089I17 gene (A530089I17Rik), mRNA.
GI_23592945-S	146.7989	1.8	Eef1a1	XM_134967.2	Mus musculus eukaryotic translation elongation factor 1 alpha 1 (Eef1a1), mRNA.
scl011425.3_30-S	146.6244	2.01	Apoc4	NM_007385.2	Mus musculus apolipoprotein C-IV (Apoc4), mRNA.
scl43293.5.1_3-S	146.6013	11.7	Twistnb	NM_172253.1	Mus musculus TWIST neighbor (Twistnb), mRNA.
scl068135.3_22-S	146.5709	23.6	Eif3s3	NM_080635.1	Mus musculus eukaryotic translation initiation factor 3, subunit 3 (gamma) (Eif3s3), mRNA.
scl020384.11_30-S	146.1317	6.34	Sfrs5	NM_009159	Mus musculus splicing factor, arginine/serine-rich 5 (SRp40, HRS) (Sfrs5), mRNA.
scl51843.7.3_62-S	145.8873	4.56	1810029G24Rik	NM_025468.1	Mus musculus RIKEN cDNA 1810029G24 gene (1810029G24Rik), mRNA.
scl023986.1_240-S	145.5839	28.55	Peci	NM_011868.1	Mus musculus peroxisomal delta3, delta2-enoyl-Coenzyme A isomerase (Peci), mRNA.
scl50289.12.1_79-S	145.3249	2.32	Phf10	NM_024250.3	Mus musculus PHD finger protein 10 (Phf10), mRNA.
scl020826.1_68-S	144.5763	23	Ssfa1	XM_196564.2	Mus musculus sperm specific antigen 1 (Ssfa1), mRNA.

scl0021766.1_289-S	144.565	3.52	Tex261	NM_009357.1	Mus musculus testis expressed gene 261 (Tex261), mRNA.
scl070093.11_3-S	144.4982	2.91	Ube2q	NM_027315.2	
scl17336.3.1_84-S	144.4776	3.69	2810025M15Rik	NM_027274.2	Mus musculus RIKEN cDNA 2810025M15 gene (2810025M15Rik), mRNA.
scl00001.1_0-S	144.3809	27.48	2400010D15Rik	NM_029623.1	Mus musculus RIKEN cDNA 2400010D15 gene (2400010D15Rik), mRNA.
scl052898.2_0-S	144.0491	3.3	D11Bwg0434e	NM_173742.1	Mus musculus DNA segment, Chr 11, Brigham & Womens Genetics 0434 expressed (D11Bwg0434e), mRNA.
scl38628.18.8_108-S	143.9689	50.27	Txnrd1	NM_015762.1	Mus musculus thioredoxin reductase 1 (Txnrd1), mRNA.
scl0067427.2_328-S	143.9246	1.75	Rps20	NM_026147.3	Mus musculus ribosomal protein S20 (Rps20), mRNA.
scl066576.4_1-S	143.9102	2.06	Uqcrh	NM_025641.2	
scl00104570.2_297-S	143.7999	3.81	AW011752	NM_134034.1	Mus musculus expressed sequence AW011752 (AW011752), mRNA.
scl0003877.1_0-S	143.7575	23.01	Aldh8a1	NM_178713	Mus musculus aldehyde dehydrogenase 8 family, member A1 (Aldh8a1), mRNA.
scl49001.3_229-S	143.1698	2.05	Cggbp1	NM_178647.2	Mus musculus CGG triplet repeat binding protein 1 (Cggbp1), mRNA.
scl068349.3_1-S	143.0438	1.89	Ndufs3	XM_130347.1	Mus musculus NADH dehydrogenase (ubiquinone) Fe-S protein 3 (Ndufs3), mRNA.
scl022194.1_318-S	142.9654	10.29	Ube2e1	NM_009455.2	Mus musculus ubiquitin-conjugating enzyme E2E 1, UBC4/5 homolog (yeast) (Ube2e1), mRNA.
scl056529.1_95-S	142.7805	1.84	Spc18	NM_019951.1	Mus musculus signal peptidase complex (Spc18), mRNA.
scl35488.3_0-S	142.7587	3.09	Rnf7	XM_135065.1	Mus musculus ring finger protein 7 (Rnf7), mRNA.
scl0099650.1_60-S	142.7034	3.38	4933434E20Rik	NM_025762.1	Mus musculus RIKEN cDNA 4933434E20 gene (4933434E20Rik), mRNA.
scl011806.4_161-S	142.6252	1.72	Apoa1	NM_009692.1	Mus musculus apolipoprotein A-I (Apoa1), mRNA.
scl54681.4_59-S	142.6036	3.09	Sh3bgrl	NM_019989.2	Mus musculus SH3-binding domain glutamic acid-rich protein like (Sh3bgrl), mRNA.
scl23666.9_53-S	142.177	1.8	Lypla2	NM_011942.1	Mus musculus lysophospholipase 2 (Lypla2), mRNA.
scl011363.1_35-S	142.1475	9.77	Acadl	NM_007381.2	Mus musculus acetyl-Coenzyme A dehydrogenase, long-chain (Acadl), mRNA.
scl0002075.1_35-S	141.7597	12.37	Fgg	NM_133862.1	Mus musculus fibrinogen, gamma polypeptide (Fgg), mRNA.
scl21209.20_282-S	141.4516	4.37	Yme1l1	NM_013771.2	Mus musculus YME1-like 1 (S. cerevisiae) (Yme1l1), mRNA.
scl000022.1_12-S	141.3658	3.5	Cpt2	NM_009949	Mus musculus carnitine palmitoyltransferase 2 (Cpt2), mRNA.
scl47965.12.3_6-S	141.185	2.69	Atp6v1c1	NM_025494	Mus musculus ATPase, H+ transporting, V1 subunit C, isoform 1 (Atp6v1c1), mRNA.
scl29411.4.6_15-S	141.026	3.11	Mgst1	NM_019946.3	Mus musculus microsomal glutathione S-transferase 1 (Mgst1), mRNA.
scl056248.1_79-S	140.8964	10.32	Ak3l	NM_021299.1	Mus musculus adenylate kinase 3 alpha-like (Ak3l), mRNA.
scl069077.10_34-S	140.5943	28.22	Psmd11	NM_178616.2	Mus musculus proteasome (prosome, macropain) 26S subunit, non-ATPase, 11 (Psmd11), mRNA.
scl0015016.1_231-S	140.3435	3.96	H2-Q5	NM_010393.1	Mus musculus histocompatibility 2, Q region locus 5 (H2-Q5), mRNA.
scl0234875.1_259-S	140.2592	2.07	Ttc13	XM_358379.1	
scl45932.17_232-S	140.0715	20.44	Tm9sf2	NM_080556.2	Mus musculus transmembrane 9 superfamily member 2 (Tm9sf2), mRNA.
scl011532.9_123-S	139.8895	69.62	Adh5	NM_007410.2	Mus musculus alcohol dehydrogenase 5 (class III), chi polypeptide (Adh5), mRNA.
scl17783.10_25-S	139.8224	4.2	BC038286	NM_170755.1	Mus musculus cDNA sequence BC038286 (BC038286), mRNA.
scl0001364.1_49-S	139.6967	1.73	Ppia	XM_122180.1	Mus musculus peptidylprolyl isomerase A (Ppia), mRNA.
scl20221.14.1_27-S	139.2488	24.61	2310003L22Rik	NM_027093.1	Mus musculus RIKEN cDNA 2310003L22 gene (2310003L22Rik), mRNA.
scl000941.1_0-S	139.1501	2.67	Ndufs2	NM_153064.3	Mus musculus NADH dehydrogenase (ubiquinone) Fe-S protein 2 (Ndufs2), mRNA.
scl00268490.1_147-S	139.079	6.23	2600001B17Rik	NM_172947.1	
scl0223254.9_31-S	138.9522	2.87	Farp1		Mus musculus FERM, RhoGEF (Arhgef) and pleckstrin domain protein 1 (chondrocyte-derived) (Farp1), mRNA.
scl0002344.1_54-S	138.9404	1.71	Serpina1b	NM_009244.2	Mus musculus serine (or cysteine) proteinase inhibitor, clade A, member 1b (Serpina1b), mRNA.
scl28426.4.1_40-S	138.8875	4.29	Grcc2f	NM_013536.1	Mus musculus gene rich cluster, C2f gene (Grcc2f), mRNA.
scl36332.17_16-S	138.3714	2.56	Catnb	NM_007614.2	Mus musculus catenin beta (Catnb), mRNA.
scl47044.6.24_75-S	137.8896	2.8	0610041B22Rik	NM_025340.1	Mus musculus RIKEN cDNA 0610041B22 gene (0610041B22Rik), mRNA.
scl21986.3.1_30-S	137.602	2.75	Apoa1bp	NM_144897	Mus musculus apolipoprotein A-I binding protein (Apoa1bp), mRNA.
scl011737.7_8-S	137.5145	12.1	Anp32a	NM_009672.2	Mus musculus acidic (leucine-rich) nuclear phosphoprotein 32 family, member A (Anp32a), mRNA.
scl25160.11.1_73-S	137.2433	2.45	C030002N13Rik	NM_145550.1	Mus musculus RIKEN cDNA C030002N13 gene (C030002N13Rik), mRNA.
scl018786.2_1-S	137.2222	3.26	Plaa	NM_172695.1	Mus musculus phospholipase A2, activating protein (Plaa), mRNA.
scl0002287.1_881-S	137.0993	5.13	D12Ertd771e	NM_028262.1	Mus musculus DNA segment, Chr 12, ERATO Doi 771, expressed (D12Ertd771e), mRNA.
scl018655.12_25-S	136.9326	2.11	Pgk1	NM_008828	Mus musculus phosphoglycerate kinase 1 (Pgk1), mRNA.
scl40204.6.1_30-S	136.8272	1.75	Atox1	NM_009720.2	Mus musculus ATX1 (antioxidant protein 1) homolog 1 (yeast) (Atox1), mRNA.
scl39338.3_4-S	136.7061	2.71	Nt5c	NM_015807.1	Mus musculus 5,3-nucleotidase, cytosolic (Nt5c), mRNA.
scl0003990.1_13-S	135.9621	2.23	Hip2	NM_016786.2	Mus musculus huntingtin interacting protein 2 (Hip2), mRNA.
scl48385.2_92-S	135.931	3.37	Rg9mtd1	NM_029092.1	Mus musculus RNA (guanine-9-) methyltransferase domain containing 1 (Rg9mtd1), mRNA.
scl0018472.1_99-S	135.6979	5.74	Pafah1b1	NM_013625.1	Mus musculus platelet-activating factor acetylhydrolase, isoform 1b, beta1 subunit (Pafah1b1), mRNA.
scl0015568.1_223-S	135.3424	1.89	Elavl1	NM_010485.2	Mus musculus ELAV (embryonic lethal, abnormal vision, Drosophila)-like 1 (Hu antigen R) (Elavl1), mRNA.
scl023994.4_204-S	135.2242	2.8	Dazap2	NM_011873.2	Mus musculus DAZ associated protein 2 (Dazap2), mRNA.
scl0002776.1_66-S	135.0748	1.94	Eif3s2	NM_018799	Mus musculus eukaryotic translation initiation factor 3, subunit 2 (beta) (Eif3s2), mRNA.
scl47732.2_4-S	135.0481	3.55	Atf4	XM_139474.1	Mus musculus activating transcription factor 4 (Atf4), mRNA.

scl23930.5.1_96-S	134.9271	2.05	Atp6v0b	NM_033617.1	Mus musculus ATPase, H+ transporting, V0 subunit B (Atp6v0b), mRNA.
scl44185.3.1_24-S	134.81	63.18	Them2	NM_025790.1	Mus musculus thioesterase superfamily member 2 (Them2), mRNA.
scl0066142.1_255-S	134.8036	26.11	Cox7b	NM_025379.1	Mus musculus cytochrome c oxidase subunit VIIb (Cox7b), mRNA.
scl23478.14.23_30-S	134.773	1.72	Park7	NM_020569.1	Mus musculus Parkinson disease (autosomal recessive, early onset) 7 (Park7), mRNA.
scl027050.3_42-S	134.5845	1.73	Rps3	NM_012052.1	Mus musculus ribosomal protein S3 (Rps3), mRNA.
scl25152.5.1_78-S	134.5317	2.47	Magoh	NM_010760.1	Mus musculus mago-nashi homolog, proliferation-associated (Drosophila) (Magoh), mRNA.
scl36431.25.1_92-S	134.4012	2.37	Scap	XM_135190.4	Mus musculus Sreb cleavage-activating protein (Scap), mRNA.
scl32408.23_56-S	134.1222	25.73	Picalm	NM_146194	Mus musculus phosphatidylinositol binding clathrin assembly protein (Picalm), mRNA.
scl43155.10.40_7-S	134.1171	2.71	Klhdc2	NM_027117.1	Mus musculus kelch domain containing 2 (Klhdc2), mRNA.
scl24019.5_109-S	133.8132	1.87	Cpt2	NM_009949	Mus musculus carnitine palmitoyltransferase 2 (Cpt2), mRNA.
scl16202.3.56_30-S	133.803	36.58	Cfh	NM_009888.2	Mus musculus complement component factor h (Cfh), mRNA.
scl45457.5_178-S	133.677	8.12	6330409N04Rik	NM_025697.1	Mus musculus RIKEN cDNA 6330409N04 gene (6330409N04Rik), mRNA.
scl24797.1_325-S	133.4853	6.28	Usp48	NM_028344.1	
scl0020843.2_126-S	133.3416	17.37	Stag2	NM_021465.2	Mus musculus stromal antigen 2 (Stag2), mRNA.
scl030056.1_208-S	133.0351	2.01	Timm10	NM_013896.2	Mus musculus translocase of inner mitochondrial membrane 10 homolog (yeast) (Timm10), mRNA.
scl0012861.1_173-S	132.7867	2.26	Cox6a1	NM_007748.2	Mus musculus cytochrome c oxidase, subunit VI a, polypeptide 1 (Cox6a1), mRNA.
scl51769.42.1_6-S	132.6309	10.63	Myo5b	NM_201600.1	Mus musculus myosin Vb (Myo5b), transcript variant 1, mRNA.
scl0027407.2_308-S	131.7506	3.06	Abcf2	NM_013853.1	Mus musculus ATP-binding cassette, sub-family F (GCN20), member 2 (Abcf2), mRNA.
scl000287.1_1-S	131.6596	1.86	AI043088	NM_198613.1	Mus musculus expressed sequence AI043088 (AI043088), mRNA.
scl014619.1_28-S	131.6575	4.3	Gjb2	NM_008125.2	Mus musculus gap junction membrane channel protein beta 2 (Gjb2), mRNA.
scl027419.6_267-S	131.5771	2.13	Naglu	NM_013792.1	Mus musculus alpha-N-acetylglucosaminidase (Sanfilippo disease IIIB) (Naglu), mRNA.
scl23509.31_82-S	130.9359	1.93	Ube4b	NM_022022.1	Mus musculus ubiquitination factor E4B, UFD2 homolog (S. cerevisiae) (Ube4b), mRNA.
scl022187.2_0-S	130.576	1.78	Ubb	NM_011664.1	Mus musculus ubiquitin B (Ubb), mRNA.
scl19959.12_665-S	130.4915	2.57	Hnf4a	NM_008261.2	
scl44752.2.1_30-S	130.4167	1.71	2010110M21Rik	NM_025933.1	Mus musculus RIKEN cDNA 2010110M21 gene (2010110M21Rik), mRNA.
scl0001345.1_85-S	130.0494	15.25		XM_181312.3	Mus musculus clathrin, heavy polypeptide (Hc) (Cltc), mRNA.
scl013685.3_18-S	129.9886	1.74	Eif4ebp1	NM_007918.2	Mus musculus eukaryotic translation initiation factor 4E binding protein 1 (Eif4ebp1), mRNA.
scl23015.4.5_22-S	129.7524	1.79	Mrpl24	NM_026591.1	Mus musculus mitochondrial ribosomal protein L24 (Mrpl24), mRNA.
scl36067.18_4-S	129.7127	1.69	Aplp2	NM_009691.1	Mus musculus amyloid beta (A4) precursor-like protein 2 (Aplp2), mRNA.
scl35445.15.1_13-S	129.6613	3.18	Pccb	NM_025835.1	Mus musculus propionyl Coenzyme A carboxylase, beta polypeptide (Pccb), mRNA.
scl011727.2_300-S	129.4298	22.97	Ang1	NM_007447	Mus musculus angiogenin, ribonuclease A family, member 1 (Ang1), mRNA.
scl36472.2.242_20-S	129.4259	1.68	Gpx1	NM_008160.1	Mus musculus glutathione peroxidase 1 (Gpx1), mRNA.
scl52692.1_233-S	129.2072	3.14	Eif4a1	NM_144958	Mus musculus eukaryotic translation initiation factor 4A1 (Eif4a1), mRNA.
scl0020750.1_112-S	128.9623	3.32	Spp1	NM_009263.1	Mus musculus secreted phosphoprotein 1 (Spp1), mRNA.
scl38085.1.103_16-S	128.7314	2.34	Hint3	NM_025798.2	
scl37896.8_186-S	127.9328	14.3	2510003E04Rik	NM_028197.1	Mus musculus RIKEN cDNA 2510003E04 gene (2510003E04Rik), mRNA.
scl0003100.1_18-S	127.9121	19.79	2310047O13Rik	NM_024185.3	Mus musculus RIKEN cDNA 2310047O13 gene (2310047O13Rik), mRNA.
scl0017975.1_309-S	127.6514	4.18	Ncl	NM_010880.2	Mus musculus nucleolin (Ncl), mRNA.
scl28312.12.1_12-S	127.407	4.51	Csda	NM_139117.1	Mus musculus cold shock domain protein A (Csda), mRNA.
scl00170791.1_106-S	127.1285	2.97	Rnpc2	NM_133242.1	Mus musculus RNA-binding region (RNP1, RRM) containing 2 (Rnpc2), mRNA.
scl28129.1_67-S	126.8419	19.28	Fzd1	NM_021457.2	Mus musculus frizzled homolog 1 (Drosophila) (Fzd1), mRNA.
scl37183.6.1_31-S	126.4217	6.32	Thy28	NM_144543.1	Mus musculus thymocyte protein thy28 (Thy28), mRNA.
scl056404.1_12-S	125.9946	22.43	Trip4	NM_019797.2	Mus musculus thyroid hormone receptor interactor 4 (Trip4), mRNA.
scl067267.4_0-S	125.9123	1.97	2900010M23Rik	NM_026063.1	Mus musculus RIKEN cDNA 2900010M23 gene (2900010M23Rik), mRNA.
scl34743.1.1_137-S	125.8652	19.55	BC003498	NM_030263.2	Mus musculus cDNA sequence BC00398 (BC003498), mRNA.
scl0229096.2_166-S	125.699	19.36	Ythdf3	NM_172677.2	
scl0015516.2_144-S	125.2907	1.91	Hspcb	NM_008302.2	Mus musculus heat shock protein 1, beta (Hspcb), mRNA.
scl28291.1_56-S	124.7476	6.83	Tctex1	NM_009342	Mus musculus t-complex testis expressed 1 (Tctex1), mRNA.
scl0003279.1_9-S	124.6896	2.37	Dpm1	NM_010072.2	Mus musculus dolichol-phosphate (beta-D) mannosyltransferase 1 (Dpm1), mRNA.
scl0003286.1_154-S	124.4484	1.98	Edf1	NM_021519.1	Mus musculus endothelial differentiation-related factor 1 (Edf1), mRNA.
scl30651.1_27-S	124.0108	1.78	Sephs2	NM_009266.1	Mus musculus selenophosphate synthetase 2 (Sephs2), mRNA.
scl23678.5.1_21-S	123.9561	2.24	3200001F09Rik	NM_027935.1	Mus musculus RIKEN cDNA 3200001F09 gene (3200001F09Rik), mRNA.
scl015510.1_74-S	123.6325	2.47	Hspd1	NM_010477.2	Mus musculus heat shock protein 1 (chaperonin) (Hspd1), mRNA.
scl066167.1_147-S	123.0888	22.85	1110017O22Rik	NM_183250.1	Mus musculus RIKEN cDNA 1110017O22 gene (1110017O22Rik), mRNA.
scl0012934.1_152-S	123.0565	2.16	Dpysl2	NM_009955.2	Mus musculus dihydropyrimidinase-like 2 (Dpysl2), mRNA.
scl17120.3.641_166-S	122.9721	4.3	Srp9	NM_012058.2	Mus musculus signal recognition particle 9 (Srp9), mRNA.

scl21916.1_21-S	122.9349	3.33	S100a1	NM_011309.2	Mus musculus S100 calcium binding protein A1 (S100a1), mRNA.
scl014548.2_14-S	122.2921	1.9	Mrps33	NM_010270.1	Mus musculus mitochondrial ribosomal protein S33 (Mrps33), mRNA.
scl47390.18.104_38-S	122.1836	36.11	Tars	NM_033074.2	Mus musculus threonyl-tRNA synthetase (Tars), mRNA.
scl0011308.2_118-S	122.1369	2.24	Abi1	NM_007380.1	Mus musculus abl-interactor 1 (Abi1), mRNA.
scl29045.3.1_38-S	121.8732	4.34	Rarres2	NM_027852.1	Mus musculus retinoic acid receptor responder (tazarotene induced) 2 (Rarres2), mRNA.
scl49065.9.1_50-S	121.7838	2.69	Apg3l	NM_026402.1	
scl34280.9.1_28-S	121.7473	20.59	2310061C15Rik	NM_026844.2	
scl34266.7_421-S	121.5476	3.32	2700067E09Rik	NM_175185.2	Mus musculus RIKEN cDNA 2700067E09 gene (2700067E09Rik), mRNA.
scl0093762.1_285-S	121.5281	23.77	Smarca5	NM_053124.2	Mus musculus SWI/SNF related, matrix associated, actin dependent regulator of chromatin, subfamily a, member 5 (Smarca5), mRNA.
scl23946.8.1_41-S	121.4767	1.67	Urod	NM_009478.1	Mus musculus uroporphyrinogen decarboxylase (Urod), mRNA.
scl0001170.1_113-S	121.3348	16.48	Crbn	NM_175357.1	
scl0098711.1_0-S	120.9426	3.54	Rdh10	NM_133832.2	Mus musculus retinol dehydrogenase 10 (all-trans) (Rdh10), mRNA.
scl052469.1_143-S	120.6743	1.79	D11Ertd99e	NM_026618.1	Mus musculus DNA segment, Chr 11, ERATO Doi 99, expressed (D11Ertd99e), mRNA.
scl0014933.2_154-S	120.4834	2.38	Gyk	NM_008194	Mus musculus glycerol kinase (Gyk), transcript variant 2, mRNA.
scl51713.6.3_46-S	120.1373	1.64	Cyb5	NM_025797	Mus musculus cytochrome b-5 (Cyb5), mRNA.
scl51927.6.1_20-S	119.4644	2.75	Phax	NM_019996.2	Mus musculus phosphorylated adaptor for RNA export (Phax), mRNA.
scl30098.24.1_15-S	119.4635	2.92	Cul1	NM_012042.3	Mus musculus cullin 1 (Cul1), mRNA.
scl018789.3_4-S	119.4516	23.25	Papola	NM_011112.1	Mus musculus poly (A) polymerase alpha (Papola), mRNA.
scl0014567.2_263-S	119.2308	2.06	Gdi1	NM_010273.1	Mus musculus guanosine diphosphate (GDP) dissociation inhibitor 1 (Gdi1), mRNA.
scl29852.1_47-S	119.18	2.29	Mrpl53	NM_026744.1	Mus musculus mitochondrial ribosomal protein L53 (Mrpl53), mRNA.
scl028071.4_2-S	118.7323	19.83	Twistnb	NM_172253.1	Mus musculus TWIST neighbor (Twistnb), mRNA.
scl0020955.2_50-S	118.7056	18.56	Sybl1	NM_011515.1	Mus musculus synaptobrevin like 1 (Sybl1), mRNA.
scl017993.1_123-S	118.6799	2.49	Ndufs4	NM_010887.1	Mus musculus NADH dehydrogenase (ubiquinone) Fe-S protein 4 (Ndufs4), mRNA.
scl058809.2_14-S	118.1805	4.11	Rnase4	NM_021472.1	Mus musculus ribonuclease, RNase A family 4 (Rnase4), mRNA.
scl0002504.1_31-S	118.1242	19.02	Atf4	NM_009716	Mus musculus activating transcription factor 4 (Atf4), mRNA.
scl37813.3.1_14-S	118.0442	10.2	Ddt	NM_010027.1	Mus musculus D-dopachrome tautomerase (Ddt), mRNA.
scl36930.15.1_0-S	118.0117	3.33	3010021M21Rik	NM_180600.1	Mus musculus RIKEN cDNA 3010021M21 gene (3010021M21Rik), mRNA.
scl030963.1_56-S	118.0002	49.06	Ptpla	NM_013935.1	Mus musculus protein tyrosine phosphatase-like (proline instead of catalytic arginine), member a (Ptpla), mRNA.
scl000890.1_7-S	117.8697	27.34	Apoa2	NM_013474	Mus musculus apolipoprotein A-II (Apoa2), mRNA.
scl019981.2_48-S	117.8249	2.38	Rpl37a	NM_009084.2	Mus musculus ribosomal protein L37a (Rpl37a), mRNA.
scl011807.2_4-S	117.2787	1.72	Apoa2	NM_013474.1	Mus musculus apolipoprotein A-II (Apoa2), mRNA.
scl0027057.2_200-S	117.2382	10.73	Ncoa4	NM_019744.1	Mus musculus nuclear receptor coactivator 4 (Ncoa4), mRNA.
scl020813.1_157-S	117.1587	1.82	Srp14	NM_009273.2	Mus musculus signal recognition particle 14 (Srp14), mRNA.
scl54843.2_132-S	117.1099	1.65	Ssr4	NM_009279	Mus musculus signal sequence receptor, delta (Ssr4), mRNA.
scl48020.10.1_127-S	116.9264	3.54	2310016A09Rik	NM_181588.2	Mus musculus RIKEN cDNA 2310016A09 gene (2310016A09Rik), mRNA.
scl0076890.1_96-S	116.7646	8.6	0610016J10Rik	NM_133771.1	Mus musculus RIKEN cDNA 0610016J10 gene (0610016J10Rik), mRNA.
scl068195.1_129-S	116.6964	1.63	Rnaset2	NM_026611.1	Mus musculus ribonuclease T2 (Rnaset2), mRNA.
scl067201.1_106-S	116.5712	21.18	2700085E05Rik	NM_026029.1	Mus musculus RIKEN cDNA 2700085E05 gene (2700085E05Rik), mRNA.
scl000492.1_58-S	116.5116	27.17	Smbp	NM_133352.1	Mus musculus SM-11044 binding protein (Smbp), mRNA.
scl36833.9.1_209-S	116.47	6.14	Rpl4	NM_024212.2	Mus musculus ribosomal protein L4 (Rpl4), mRNA.
scl066359.2_17-S	116.4507	2.78	2310005N03Rik	NM_025511.1	Mus musculus RIKEN cDNA 2310005N03 gene (2310005N03Rik), mRNA.
scl0002714.1_31-S	116.3894	4.22	Nipsnap3b	NM_025623.1	Mus musculus nipsnap homolog 3B (C. elegans) (Nipsnap3b), mRNA.
scl067671.4_8-S	116.3012	2.02	Rpl38	NM_023372.1	Mus musculus ribosomal protein L38 (Rpl38), mRNA.
scl068177.1_35-S	116.2452	1.8	Ebpl	NM_026598.1	Mus musculus emopamil binding protein-like (Ebpl), mRNA.
scl0001502.1_0-S	116.1966	6.97	Hspa4	NM_008300.2	Mus musculus heat shock protein 4 (Hspa4), mRNA.
scl012727.1_314-S	116.101	15.61	Clcn4-2	NM_011334.2	Mus musculus chloride channel 4-2 (Clcn4-2), mRNA.
scl022017.1_28-S	116.0971	3.9	Tpmt	NM_016785	Mus musculus thiopurine methyltransferase (Tpmt), mRNA.
scl0235043.1_144-S	115.5335	1.76	MGC18837	NM_178577.3	
scl25250.2.1_9-S	115.4678	46.65	Angptl3	NM_013913.1	Mus musculus angiopoietin-like 3 (Angptl3), mRNA.
scl0002109.1_724-S	115.3457	21.07	Dnajb4	NM_025926.1	Mus musculus DnaJ (Hsp40) homolog, subfamily B, member 4 (Dnajb4), mRNA.
scl51125.19.1_152-S	115.3056	2.51	Plg	NM_008877.2	Mus musculus plasminogen (Plg), mRNA.
scl068045.1_243-S	115.304	1.64	2700060E02Rik	NM_026528.1	Mus musculus RIKEN cDNA 2700060E02 gene (2700060E02Rik), mRNA.
scl31056.12.1_57-S	115.2954	5.07	Eed	NM_021876.1	Mus musculus embryonic ectoderm development (Eed), mRNA.
scl015481.10_184-S	115.246	3.79	Hspa8	NM_031165	Mus musculus heat shock protein 8 (Hspa8), mRNA.
scl0102866.1_231-S	115.2035	4.05	Pls3	NM_145629.1	Mus musculus plastin 3 (T-isoform) (Pls3), mRNA.
scl0268373.4_12-S	114.9135	1.63	Ppia	NM_008907	Mus musculus peptidylprolyl isomerase A (Ppia), mRNA.

scl54430.4.1_35-S	114.8795	1.82	Ebp	NM_007898.2	Mus musculus phenylalkylamine Ca2+ antagonist (emopamil) binding protein (Ebp), mRNA.
scl0015191.2_38-S	114.7939	1.9	Hdgf	NM_008231.2	Mus musculus hepatoma-derived growth factor (Hdgf), mRNA.
scl29314.10.1_67-S	114.7519	1.7	Pon1	NM_011134.1	Mus musculus paraoxonase 1 (Pon1), mRNA.
scl011958.2_29-S	114.4457	57	Atp5k	NM_007507	Mus musculus ATP synthase, H+ transporting, mitochondrial F1F0 complex, subunit e (Atp5k), mRNA.
scl29872.10.1_133-S	114.28	95.59	Suclg1	NM_019879.1	Mus musculus succinate-CoA ligase, GDP-forming, alpha subunit (Suclg1), mRNA.
scl0098314.1_111-S	114.2686	18.36	AI325464	NM_178882.2	Mus musculus expressed sequence AI325464 (AI325464), mRNA.
scl24033.7.1_11-S	114.1212	1.73	Mrpl37	NM_025500.1	Mus musculus mitochondrial ribosomal protein L37 (Mrpl37), mRNA.
scl0067857.2_151-S	113.9903	1.84	Ppp6c	NM_024209.1	Mus musculus protein phosphatase 6, catalytic subunit (Ppp6c), mRNA.
scl0066377.1_24-S	113.9836	1.71	Ndufc1	NM_025523.1	Mus musculus NADH dehydrogenase (ubiquinone) 1, subcomplex unknown, 1 (Ndufc1), mRNA.
scl0019244.1_47-S	113.9319	2.06	Ptp4a2	NM_008974.2	Mus musculus protein tyrosine phosphatase 4a2 (Ptp4a2), mRNA.
scl013909.6_35-S	113.8314	5.51	LOC13909	NM_144511.1	Mus musculus esterase 31-like (LOC13909), mRNA.
scl068152.6_9-S	113.7815	6.33	5830415L20Rik	NM_026583.2	Mus musculus RIKEN cDNA 5830415L20 gene (5830415L20Rik), mRNA.
scl012864.3_58-S	113.488	2.67	Cox6c	NM_053071.1	Mus musculus cytochrome c oxidase, subunit VIc (Cox6c), mRNA.
scl018412.1_16-S	113.4459	2.02	Sqstm1	NM_011018.1	Mus musculus sequestosome 1 (Sqstm1), mRNA.
scl0026442.1_315-S	113.326	26.95	Psma5	NM_011967.1	Mus musculus proteasome (prosome, macropain) subunit, alpha type 5 (Psma5), mRNA.
scl014470.4_28-S	113.2398	2.03	Rabac1	NM_010261.1	Mus musculus Rab acceptor 1 (prenylated) (Rabac1), mRNA.
scl0027029.2_174-S	113.2144	4.8	Sgsh	NM_018822.2	Mus musculus N-sulfoglucosamine sulfohydrolase (sulfamidase) (Sgsh), mRNA.
scl067673.2_0-S	113.1564	1.66	Tceb2	NM_026305.1	Mus musculus transcription elongation factor B (SIII), polypeptide 2 (Tceb2), mRNA.
scl28216.21.1_39-S	113.1123	10.47	Sox5	NM_011444.1	Mus musculus SRY-box containing gene 5 (Sox5), mRNA.
scl53438.9.1_79-S	113.0242	1.63	Ndufv1	NM_133666.1	Mus musculus NADH dehydrogenase (ubiquinone) flavoprotein 1 (Ndufv1), mRNA.
scl0002737.1_15-S	112.963	3.52	Mrps15	NM_025544.1	Mus musculus mitochondrial ribosomal protein S15 (Mrps15), mRNA.
scl51768.11.1_9-S	112.7917	1.9	Acaa2	NM_177470.2	Mus musculus acetyl-Coenzyme A acyltransferase 2 (mitochondrial 3-oxoacyl-Coenzyme A thiolase) (Acaa2), mRNA.
scl46052.12.1_12-S	112.7718	36.49	Sugt1	NM_026474.2	Mus musculus SGT1, suppressor of G2 allele of SKP1 (S. cerevisiae) (Sugt1), mRNA.
scl068365.1_71-S	112.7393	1.69	Rab14	NM_026697.2	Mus musculus RAB14, member RAS oncogene family (Rab14), mRNA.
scl35194.5_5-S	112.6843	16.09	Hig1	NM_019814.2	Mus musculus hypoxia induced gene 1 (Hig1), mRNA.
scl067945.1_102-S	112.532	1.62	Rpl41	NM_018860.2	Mus musculus ribosomal protein L41 (Rpl41), mRNA.
scl066390.1_295-S	112.1549	1.67	2310042G06Rik	NM_025531.2	Mus musculus RIKEN cDNA 2310042G06 gene (2310042G06Rik), mRNA.
scl017113.8_48-S	112.01	2.15	M6pr	NM_010749.4	Mus musculus mannose-6-phosphate receptor, cation dependent (M6pr), mRNA.
scl27367.4.1_8-S	111.9313	1.83	Pop5	NM_026398.2	Mus musculus processing of precursor 5, ribonuclease P/MRP family (S. cerevisiae) (Pop5), mRNA.
scl25694.8.816_12-S	111.9223	1.66	Rab2	NM_021518.2	Mus musculus RAB2, member RAS oncogene family (Rab2), mRNA.
scl24399.13_176-S	111.9193	5.02	Gba2	NM_172692.1	Mus musculus glucosidase beta 2 (Gba2), mRNA.
scl34554.11.1_42-S	111.9156	15.56	Gcdh	NM_008097.1	Mus musculus glutaryl-Coenzyme A dehydrogenase (Gcdh), mRNA.
scl45887.9.1_15-S	111.8421	11.98	2400006A19Rik	NM_025550.2	Mus musculus RIKEN cDNA 2400006A19 gene (2400006A19Rik), mRNA.
scl066383.5_131-S	111.8416	1.74	2310020H20Rik	NM_025526.1	Mus musculus RIKEN cDNA 2310020H20 gene (2310020H20Rik), mRNA.
scl19764.11_583-S	111.8365	2.77	Tpd52l2	NM_025482.2	Mus musculus tumor protein D52-like 2 (Tpd52l2), mRNA.
scl016647.2_13-S	111.8019	14.14	Kpna2	XM_130821.1	Mus musculus karyopherin (importin) alpha 2 (Kpna2), mRNA.
scl21117.18_194-S	111.7538	5.19	AW060766	NM_198033.1	Mus musculus expressed sequence AW060766 (AW060766), mRNA.
scl020115.2_55-S	111.5267	1.61	Rps7	NM_011300	Mus musculus ribosomal protein S7 (Rps7), mRNA.
scl24046.11_327-S	111.4919	3.11	C8a	NM_146148.1	Mus musculus complement component 8, alpha polypeptide (C8a), mRNA.
scl19685.22.1_10-S	111.4467	1.72	Itih2	NM_010582.1	Mus musculus inter-alpha trypsin inhibitor, heavy chain 2 (Itih2), mRNA.
scl42309.7.1_14-S	111.2931	2.42	Vti1b	NM_016800.2	Mus musculus vesicle transport through interaction with t-SNAREs 1B homolog (Vti1b), mRNA.
scl0209039.30_312-S	111.2733	10.56	Tenc1	NM_153533.1	Mus musculus tensin like C1 domain-containing phosphatase (Tenc1), mRNA.
scl000144.1_17-S	111.2727	6.07	Mir16	NM_019580.3	Mus musculus membrane interacting protein of RGS16 (Mir16), mRNA.
scl28761.2_42-S	111.0416	3.78	Cml2	NM_053096.2	Mus musculus camello-like 2 (Cml2), mRNA.
scl0066999.2_129-S	110.8901	2.78	1500003D12Rik	NM_025895.2	Mus musculus RIKEN cDNA 1500003D12 gene (1500003D12Rik), mRNA.
scl0012192.2_103-S	110.786	6.57	Zfp36l1	NM_007564.2	Mus musculus zinc finger protein 36, C3H type-like 1 (Zfp36l1), mRNA.
scl071779.8_36-S	110.6117	5.53	Mir	NM_027920.3	
scl37626.20.1_66-S	110.51	17.85	D10Ertd802e	NM_198021.1	Mus musculus DNA segment, Chr 10, ERATO Doi 802, expressed (D10Ertd802e), mRNA.
scl37953.14_53-S	110.4288	4.24	Man1a	NM_008548.2	Mus musculus mannosidase 1, alpha (Man1a), mRNA.
scl0020442.2_266-S	110.416	6	Siat4a	NM_009177.2	Mus musculus sialyltransferase 4A (beta-galactoside alpha-2,3-sialytransferase) (Siat4a), mRNA.
scl18647.1_62-S	110.2587	2.12	Cenpb	NM_007682.2	Mus musculus centromere autoantigen B (Cenpb), mRNA.
scl029864.1_22-S	109.9708	3	Rnf11	NM_013876.2	Mus musculus ring finger protein 11 (Rnf11), mRNA.
scl057905.1_53-S	109.9411	3.91	5830446M03Rik	NM_133934.2	Mus musculus RIKEN cDNA 5830446M03 gene (5830446M03Rik), mRNA.
scl0056207.2_50-S	109.3773	3.02	Uchl5	NM_019562.1	Mus musculus ubiquitin carboxyl-terminal esterase L5 (Uchl5), mRNA.
scl28063.9.402_1-S	109.28	38.69	Psmc2	NM_011188.1	Mus musculus proteasome (prosome, macropain) 26S subunit, ATPase 2 (Psmc2), mRNA.
scl078653.2_11-S	109.1884	1.72	1810056O20Rik	NM_175277.2	Mus musculus RIKEN cDNA 1810056O20 gene (1810056O20Rik), mRNA.

scl0012793.2_38-S	109.1371	2.34	Cnih	NM_009919.1	Mus musculus cornichon homolog (Drosophila) (Cnih), mRNA.
scl068839.1_117-S	108.8572	8.07	1110054N06Rik	NM_175134.2	Mus musculus RIKEN cDNA 1110054N06 gene (1110054N06Rik), mRNA.
scl47780.3.9_1-S	108.8079	1.87	Rpl8	NM_012053.1	Mus musculus ribosomal protein L8 (Rpl8), mRNA.
scl013722.3_29-S	108.7114	2.02	Scye1	NM_007926.1	Mus musculus small inducible cytokine subfamily E, member 1 (Scye1), mRNA.
scl31132.7.1_7-S	108.6033	1.73	Cib1	NM_011870.2	Mus musculus calcium and integrin binding 1 (calmyrin) (Cib1), mRNA.
scl016784.1_5-S	108.563	1.61	Lamp2	NM_010685.2	Mus musculus lysosomal membrane glycoprotein 2 (Lamp2), mRNA.
scl42676.6_93-S	108.3838	3.38	Rab10	NM_016676.2	Mus musculus RAB10, member RAS oncogene family (Rab10), mRNA.
scl052690.1_86-S	108.3514	9.39	D12Ertd771e	NM_028262.1	Mus musculus DNA segment, Chr 12, ERATO Doi 771, expressed (D12Ertd771e), mRNA.
scl0108991.1_11-S	108.1684	2.07	1700001A24Rik	NM_175395.2	Mus musculus RIKEN cDNA 1700001A24 gene (1700001A24Rik), mRNA.
scl020463.2_41-S	107.6437	1.71	Cox7a2l	XM_123188.1	Mus musculus cytochrome c oxidase subunit VIIa polypeptide 2-like (Cox7a2l), mRNA.
scl011758.1_35-S	107.6316	3.78	Prdx6	NM_007453.2	Mus musculus peroxiredoxin 6 (Prdx6), mRNA.
scl0021915.2_168-S	107.6279	4.8	Dtymk	NM_023136.1	Mus musculus deoxythymidylate kinase (Dtymk), mRNA.
scl0001373.1_2-S	107.425	1.73	Sumo2	NM_133354	
scl068183.7_27-S	107.4238	7.87	Bcas2	NM_026602.1	Mus musculus breast carcinoma amplified sequence 2 (Bcas2), mRNA.
scl026446.4_108-S	107.3944	1.66	Psmb3	NM_011971.2	Mus musculus proteasome (prosome, macropain) subunit, beta type 3 (Psmb3), mRNA.
scl0110842.2_3-S	107.3684	2.56	Etfa	NM_145615.2	Mus musculus electron transferring flavoprotein, alpha polypeptide (Etfa), nuclear gene encoding mitochondrial protein, mRNA.
scl00212555.2_311-S	107.3377	2.04	Pqlc2	NM_145384.1	Mus musculus PQ loop repeat containing 2 (Pqlc2), mRNA.
scl000063.1_0-S	107.286	1.91	Nit1	NM_012049.1	Mus musculus nitrilase 1 (Nit1), mRNA.
scl015516.6_124-S	107.0782	3.1	Hspcb	NM_008302.2	Mus musculus heat shock protein 1, beta (Hspcb), mRNA.
scl40468.10_17-S	106.9284	1.88	9130023F12Rik	NM_181411.2	Mus musculus RIKEN cDNA 9130023F12 gene (9130023F12Rik), mRNA.
scl0001647.1_23-S	106.828	1.88		XM_128511.3	Mus musculus RIKEN cDNA 0610011F06 gene (0610011F06Rik), mRNA.
scl44239.8_168-S	106.7558	12.03	Zfp307	NM_023685.2	
scl39451.19.1_30-S	106.6891	3.64	Ftsj3	NM_025310.2	Mus musculus FtsJ homolog 3 (E. coli) (Ftsj3), mRNA.
scl37704.3.1_30-S	106.5407	1.85	Mrpl54	NM_025317.1	Mus musculus mitochondrial ribosomal protein L54 (Mrpl54), mRNA.
scl37311.9.1_1-S	106.5347	2.71	4833420K19Rik	NM_029775.1	Mus musculus RIKEN cDNA 4833420K19 gene (4833420K19Rik), mRNA.
scl0002291.1_1-S	106.2504	2.19	Trappc6b	XM_127025.2	Mus musculus trafficking protein particle complex 6B (Trappc6b), mRNA.
scl0026458.2_190-S	106.0078	1.59	Slc27a2	NM_011978.1	Mus musculus solute carrier family 27 (fatty acid transporter), member 2 (Slc27a2), mRNA.
scl0019087.2_280-S	105.9534	29.36	Prkar2a	NM_008924.1	Mus musculus protein kinase, cAMP dependent regulatory, type II alpha (Prkar2a), mRNA.
scl27722.12.1_0-S	105.9135	2.1	Asrij	NM_023429.2	
scl42853.2.1_24-S	105.8487	14.48	D230037D09Rik	NM_177140.2	Mus musculus RIKEN cDNA D230037D09 gene (D230037D09Rik), mRNA.
scl022224.14_3-S	105.501	12.03	Usp10	NM_009462.1	Mus musculus ubiquitin specific protease 10 (Usp10), mRNA.
scl2037.1.1_223-S	105.2301	15.64	Ang2	NM_007449	Mus musculus angiogenin, ribonuclease A family, member 2 (Ang2), mRNA.
scl25516.10_536-S	105.1693	2.44	Tesk1	NM_011571.2	Mus musculus testis specific protein kinase 1 (Tesk1), mRNA.
scl0116748.2_296-S	105.1606	2.04	Lsm10	NM_138721.1	Mus musculus U7 snRNP-specific Sm-like protein LSM10 (Lsm10), mRNA.
scl067738.10_4-S	105.1459	2.67	Ppid	NM_026352.2	Mus musculus peptidylprolyl isomerase D (cyclophilin D) (Ppid), mRNA.
scl25126.19.1_29-S	105.129	1.63	Faf1	NM_007983.2	Mus musculus Fas-associated factor 1 (Faf1), mRNA.
scl0003930.1_2-S	105.0337	1.72	Bloc1s1	NM_015740.2	
scl31884.10.2_33-S	104.9356	2.6	Tm4sf7	NM_053082.1	Mus musculus transmembrane 4 superfamily member 7 (Tm4sf7), mRNA.
scl0223773.9_321-S	104.8611	18.96	Zbed4	NM_181412.2	
scl0067899.2_259-S	104.8486	1.63	2010110K16Rik	NM_026442.2	Mus musculus RIKEN cDNA 2010110K16 gene (2010110K16Rik), mRNA.
scl20180.20_84-S	104.8211	5.24	Rin2	NM_028724.2	Mus musculus Ras and Rab interactor 2 (Rin2), mRNA.
scl0102143.1_151-S	104.7285	2.77	D130072O21Rik	NM_175322.2	Mus musculus RIKEN cDNA D130072O21 gene (D130072O21Rik), mRNA.
scl37898.15_404-S	104.6088	3.32	Ddx21	NM_019553.1	Mus musculus DEAD (Asp-Glu-Ala-Asp) box polypeptide 21 (Ddx21), mRNA.
scl33243.5.1_27-S	104.5608	1.75	Cox4i1	NM_009941.1	Mus musculus cytochrome c oxidase subunit IV isoform 1 (Cox4i1), mRNA.
scl077113.1_28-S	104.4485	18.42	Klhl2	NM_178633.1	Mus musculus kelch-like 2, Mayven (Drosophila) (Klhl2), mRNA.
scl20017.4.1_0-S	104.3816	1.62	1110008F13Rik	NM_026124.1	Mus musculus RIKEN cDNA 1110008F13 gene (1110008F13Rik), mRNA.
scl32299.7.1_25-S	104.2451	2.57	Stard10	NM_019990.1	Mus musculus START domain containing 10 (Stard10), mRNA.
scl0003895.1_38-S	104.2251	1.96	Myl6	NM_010860	Mus musculus myosin, light polypeptide 6, alkali, smooth muscle and non-muscle (Myl6), mRNA.
scl0023950.1_557-S	104.1281	19.24	Dnajb6	NM_011847.1	Mus musculus DnaJ (Hsp40) homolog, subfamily B, member 6 (Dnajb6), mRNA.
scl0106298.14_12-S	104.0618	6.43	Rrn3	XM_358764.1	
scl00347709.1_259-S	104.0167	5.58	Pramel4	NM_178248.2	Mus musculus preferentially expressed antigen in melanoma like 4 (Pramel4), mRNA.
scl0078825.1_275-S	103.992	22.29	5830417C01Rik	NM_024282.2	Mus musculus RIKEN cDNA 5830417C01 gene (5830417C01Rik), mRNA.
scl44819.5_313-S	103.9454	1.66	C78339	XM_127312.4	Mus musculus expressed sequence C78339 (C78339), mRNA.
scl000756.1_295-S	103.9407	12.43	Dst	NM_133833.1	Mus musculus dystonin (Dst), mRNA.
scl46280.17_461-S	103.8051	5.32	Wdr23	NM_133734.1	Mus musculus WD repeat domain 23 (Wdr23), mRNA.
scl21513.4.14_14-S	103.6444	1.68	2310008M10Rik	NM_025509.2	Mus musculus RIKEN cDNA 2310008M10 gene (2310008M10Rik), mRNA.

scl40588.14.79_66-S	103.6122	2.31	Sec14l2	NM_144520.1	Mus musculus SEC14-like 2 (S. cerevisiae) (Sec14l2), mRNA.
scl44261.19.1_20-S	103.5288	12.43	AF397014	NM_138654.2	Mus musculus cDNA sequence AF397014 (AF397014), mRNA.
scl41351.4.1_17-S	103.4482	1.78	Gabarap	NM_019749.3	Mus musculus gamma-aminobutyric acid receptor associated protein (Gabarap), mRNA.
scl31028.4.1_18-S	103.3321	101.84	Aqp11	NM_175105.2	Mus musculus aquaporin 11 (Aqp11), mRNA.
scl066481.4_0-S	102.8888	1.58	Rps21	NM_025587.1	Mus musculus ribosomal protein S21 (Rps21), mRNA.
scl0015129.1_300-S	102.8602	13.42	Hbb-b1	NM_008220.2	Mus musculus hemoglobin, beta adult major chain (Hbb-b1), mRNA.
scl00218214.2_131-S	102.6362	22	Aof1	NM_172262.1	Mus musculus amine oxidase, flavin containing 1 (Aof1), mRNA.
scl0002992.1_44-S	102.3982	1.94	Lamp2	NM_010685.2	Mus musculus lysosomal membrane glycoprotein 2 (Lamp2), mRNA.
scl0003299.1_11-S	102.358	6.75	2810410A08Rik	XM_130324.4	Mus musculus RIKEN cDNA 2810410A08 gene (2810410A08Rik), mRNA.
scl18576.11.3_13-S	102.3223	21.4	Snx5	NM_024225.2	Mus musculus sorting nexin 5 (Snx5), mRNA.
scl17563.43_11-S	101.8118	16.44	Clasp1		Mus musculus CLIP associating protein 1 (Clasp1), mRNA.
scl069367.4_58-S	101.8117	2.48	Glrx2	NM_023505.1	Mus musculus glutaredoxin 2 (thioltransferase) (Glrx2), mRNA.
scl056612.4_0-S	101.7735	1.68	Pfdn5	NM_020031.1	Mus musculus prefoldin 5 (Pfdn5), mRNA.
scl066407.8_34-S	101.7438	3.17	Mrps15		
scl0266692.1_279-S	101.6895	12.25	Cpne1	NM_170590.1	Mus musculus copine I (Cpne1), transcript variant 1, mRNA.
scl022631.1_10-S	101.4957	14.75	Ywhaz	NM_011740.2	Mus musculus tyrosine 3-monooxygenase/tryptophan 5-monooxygenase activation protein, zeta polypeptide (Ywhaz), mRNA.
scl44520.17.1_0-S	101.4202	7.71	Dmgdh	NM_028772.1	Mus musculus dimethylglycine dehydrogenase precursor (Dmgdh), mRNA.
scl41758.26.1_24-S	101.2455	15.17	Pnpt1	NM_027869.1	Mus musculus polyribonucleotide nucleotidyltransferase 1 (Pnpt1), mRNA.
scl28064.14.1_8-S	101.2383	25.92	Pmpcb	XM_131914.3	
scl28069.32.1_8-S	101.232	10.35	A530088I07Rik	NM_175437.2	Mus musculus RIKEN cDNA A530088I07 gene (A530088I07Rik), mRNA.
scl0223499.9_30-S	101.1577	2.35	Gm83	NM_198606.1	Mus musculus gene model 83, (NCBI) (Gm83), mRNA.
scl068178.1_250-S	101.0595	1.81	4933421H10Rik	NM_026599.3	Mus musculus RIKEN cDNA 4933421H10 gene (4933421H10Rik), mRNA.
scl00110842.1_108-S	100.8896	1.57	Etfa	NM_145615.2	Mus musculus electron transferring flavoprotein, alpha polypeptide (Etfa), nuclear gene encoding mitochondrial protein, mRNA.
scl0004156.1_115-S	100.8646	1.71	1600019D15Rik	NM_028975.2	Mus musculus RIKEN cDNA 1600019D15 gene (1600019D15Rik), mRNA.
scl53786.5.1_4-S	100.7952	2.79	Psmd10	NM_016883.3	Mus musculus proteasome (prosome, macropain) 26S subunit, non-ATPase, 10 (Psmd10), mRNA.
scl0003758.1_1237-S	100.7569	1.59	Ubqln1	NM_026842.3	Mus musculus ubiquilin 1 (Ubqln1), transcript variant 1, mRNA.
scl43245.1.1_35-S	100.6973	1.75	Immp2l	NM_053122.2	Mus musculus inner mitochondrial membrane peptidase 2-like (S. cerevisiae) (Immp2l), mRNA.
scl069466.1_2-S	100.5976	17.65	2300006M17Rik	XM_127388.3	Mus musculus RIKEN cDNA 2300006M17 gene (2300006M17Rik), mRNA.
scl0002708.1_0-S	100.5808	2.16	0610037L13Rik	NM_028754.1	Mus musculus RIKEN cDNA 0610037L13 gene (0610037L13Rik), mRNA.
scl00394435.1_2-S	100.5718	17.8	Ugt1a6	NM_145079	Mus musculus UDP glycosyltransferase 1 family, polypeptide A6 (Ugt1a6), mRNA.
scl0003006.1_310-S	100.1701	2.27	Fbxo3	NM_212433.1	Mus musculus F-box only protein 3 (Fbxo3), transcript variant 2, mRNA.
scl42451.6.1_16-S	100.1239	137.25	Nfkbia	NM_010907	Mus musculus nuclear factor of kappa light chain gene enhancer in B-cells inhibitor, alpha (Nfkbia), mRNA.
scl0071702.2_200-S	99.9285	1.86	Cdc5l	NM_152810.1	Mus musculus cell division cycle 5-like (S. pombe) (Cdc5l), mRNA.
scl44846.7_0-S	99.8955	5.57	Nol7	NM_023554.1	Mus musculus nucleolar protein 7 (Nol7), mRNA.
scl021371.3_17-S	99.8825	1.6	Tbca	NM_009321.1	Mus musculus tubulin cofactor a (Tbca), mRNA.
scl0003628.1_0-S	99.8584	1.65	Ndufs4	NM_010887.1	Mus musculus NADH dehydrogenase (ubiquinone) Fe-S protein 4 (Ndufs4), mRNA.
scl20384.11.8_5-S	99.8391	1.59	Grp58	NM_007952.1	Mus musculus glucose regulated protein (Grp58), mRNA.
scl42976.13.1_6-S	99.8112	2.26	Coq6	NM_172582.1	Mus musculus coenzyme Q6 homolog (yeast) (Coq6), mRNA.
scl000075.1_18_REVCON	99.5495	1.8	AA959742	NM_133807.1	Mus musculus expressed sequence AA959742 (AA959742), mRNA.
scl18004.30_24-S	99.5367	3.95	Tpp2	NM_009418.1	Mus musculus tripeptidyl peptidase II (Tpp2), mRNA.
scl027643.2_36-S	99.3623	2.1	Ubl4	NM_145405.1	Mus musculus ubiquitin-like 4 (Ubl4), mRNA.
scl0224904.1_72-S	99.3399	2.41	2410015M20Rik	NM_153152.2	Mus musculus RIKEN cDNA 2410015M20 gene (2410015M20Rik), mRNA.
scl21701.9.1_3-S	99.2889	21.23	Atp5f1	NM_009725.1	Mus musculus ATP synthase, H+ transporting, mitochondrial F0 complex, subunit b, isoform 1 (Atp5f1), mRNA.
scl070257.1_12-S	99.2306	2.18	2010107E04Rik	NM_027360.1	Mus musculus RIKEN cDNA 2010107E04 gene (2010107E04Rik), mRNA.
scl00217869.1_91-S	99.1394	1.91	Eif5	NM_173363.2	Mus musculus eukaryotic translation initiation factor 5 (Eif5), mRNA.
scl020335.2_33-S	99.1113	2.8	Sec61g	XM_122171.1	Mus musculus SEC61, gamma subunit (Sec61g), mRNA.
scl35401.5_32-S	99.0805	2.67	6230410P16Rik	NM_174846.3	Mus musculus RIKEN cDNA 6230410P16 gene (6230410P16Rik), mRNA.
scl31564.8.5_30-S	98.9379	4.91	1200009C21Rik	NM_028659.1	Mus musculus RIKEN cDNA 1200009C21 gene (1200009C21Rik), mRNA.
scl013010.2_9-S	98.9068	1.62	Cst3	NM_009976.2	Mus musculus cystatin C (Cst3), mRNA.
scl0014858.1_22-S	98.8111	8.46	Gsta2	NM_008182.1	Mus musculus glutathione S-transferase, alpha 2 (Yc2) (Gsta2), mRNA.
scl53939.16_446-S	98.7293	3.09	Abcb7	XM_356348.1	Mus musculus ATP-binding cassette, sub-family B (MDR/TAP), member 7 (Abcb7), mRNA.
scl45769.2.281_2-S	98.5551	1.66	1810004F21Rik	NM_026911.1	Mus musculus RIKEN cDNA 1810004F21 gene (1810004F21Rik), mRNA.
scl0056690.1_158-S	98.2134	1.86	Mlycd	NM_019966.1	Mus musculus malonyl-CoA decarboxylase (Mlycd), mRNA.
scl0067264.2_318-S	98.1887	1.87	Ndufb8	NM_026061.1	Mus musculus NADH dehydrogenase (ubiquinone) 1 beta subcomplex 8 (Ndufb8), mRNA.
gi_31981889_ref_NM_00	97.8842	1.55	B2m	NM_009735.2	Mus musculus beta-2 microglobulin (B2m), mRNA.
scl098985.1_28-S	97.8283	6.77	AI462438	NM_133840.1	Mus musculus expressed sequence AI462438 (AI462438), mRNA.

scl071238.2_50-S	97.8006	3.74	Acn9	XM_355744.1	
scl0110052.3_51-S	97.7839	3.25	Dek	NM_025900.1	Mus musculus DEK oncogene (DNA binding) (Dek), mRNA.
scl00061.1_10-S	97.7463	15.54	Ppp1r14a	NM_026731.2	Mus musculus protein phosphatase 1, regulatory (inhibitor) subunit 14A (Ppp1r14a), mRNA.
scl0011973.2_248-S	97.3908	1.61	Atp6v1e1	NM_007510.2	Mus musculus ATPase, H+ transporting, V1 subunit E isoform 1 (Atp6v1e1), mRNA.
scl0230700.13_329-S	97.3332	3.87	C330039G02Rik	NM_172699.2	Mus musculus RIKEN cDNA C330039G02 gene (C330039G02Rik), mRNA.
scl0210148.13_0-S	97.2211	2.44	Slc30a6	NM_144798.2	Mus musculus solute carrier family 30 (zinc transporter), member 6 (Slc30a6), mRNA.
scl0101867.1_89-S	97.0207	16.52	1500003O22Rik	NM_025897.1	Mus musculus RIKEN cDNA 1500003O22 gene (1500003O22Rik), mRNA.
scl18363.8_35-S	96.913	4.31	Sdc4	NM_011521.1	Mus musculus syndecan 4 (Sdc4), mRNA.
scl0002135.1_327-S	96.853	43.11	Rarres1	XM_130987.3	
scl36820.13_122-S	96.7951	3	2010321M09Rik	NM_175153.2	Mus musculus RIKEN cDNA 2010321M09 gene (2010321M09Rik), mRNA.
scl28280.10.1_35-S	96.4376	14.74	1100001H23Rik	NM_025806.1	Mus musculus RIKEN cDNA 1100001H23 gene (1100001H23Rik), mRNA.
scl36022.9.1_13-S	96.2131	20.16	Tbrg1	NM_025289.1	Mus musculus transforming growth factor beta regulated gene 1 (Tbrg1), mRNA.
scl0013909.2_193-S	96.2057	4.65	LOC13909	NM_144511.1	Mus musculus esterase 31-like (LOC13909), mRNA.
scl42564.18.1_22-S	96.2019	17.13	Sntg2	NM_172951.1	Mus musculus syntrophin, gamma 2 (Sntg2), mRNA.
scl00223696.2_201-S	96.1936	6.14	Tomm22	NM_172609.2	Mus musculus translocase of outer mitochondrial membrane 22 homolog (yeast) (Tomm22), mRNA.
scl18618.8_0-S	96.051	14.33	3300001M20Rik	NM_175113.1	Mus musculus RIKEN cDNA 3300001M20 gene (3300001M20Rik), mRNA.
scl0067618.2_236-S	96.0351	5.07	Aasdhppt	NM_026276.1	Mus musculus aminoadipate-semialdehyde dehydrogenase-phosphopantetheinyl transferase (Aasdhppt), mRNA.
scl32577.4.1_30-S	95.9867	1.69	Mcee	XM_133510.4	
scl22880.8.1_176-S	95.918	13.06	Ctss	NM_021281.1	Mus musculus cathepsin S (Ctss), mRNA.
scl0002881.1_47-S	95.8997	4.81	Pdcd8	NM_012019.2	Mus musculus programmed cell death 8 (Pdcd8), mRNA.
scl0017836.1_208-S	95.8678	8.03	Mug1	NM_008645.2	Mus musculus murinoglobulin 1 (Mug1), mRNA.
scl29521.11_18-S	95.8462	2.58	Grcc3f	NM_145130.1	Mus musculus gene rich cluster, C3f gene (Grcc3f), mRNA.
scl0052040.1_253-S	95.7043	3.4	Ppp1r10	NM_175934.2	Mus musculus protein phosphatase 1, regulatory subunit 10 (Ppp1r10), mRNA.
scl066958.2_91-S	95.7001	4.72	2310042M24Rik	NM_025868.1	Mus musculus RIKEN cDNA 2310042M24 gene (2310042M24Rik), mRNA.
scl0076654.1_225-S	95.655	2.26	Upp2	NM_029692.1	Mus musculus uridine phosphorylase 2 (Upp2), mRNA.
scl00224826.1_101-S	95.6443	3.69	E130209G04Rik	XM_358323.1	Mus musculus RIKEN cDNA E130209G04 gene (E130209G04Rik), mRNA.
scl066416.3_80-S	95.4084	1.68	Ndufa7	NM_023202.2	Mus musculus NADH dehydrogenase (ubiquinone) 1 alpha subcomplex, 7 (B14.5a) (Ndufa7), mRNA.
scl51735.42.647_134-S	95.3726	2.53	5430411K18Rik	XM_140491.4	Mus musculus RIKEN cDNA 5430411K18 gene (5430411K18Rik), mRNA.
scl33697.10.1_67-S	95.2793	2.11	1110012M11Rik	NM_028617.2	Mus musculus RIKEN cDNA 1110012M11 gene (1110012M11Rik), mRNA.
scl15797.5_597-S	95.0689	3.62	Lyplal1	NM_146106.1	Mus musculus lysophospholipase-like 1 (Lyplal1), mRNA.
scl0019647.2_60-S	94.7168	11.37	Rbbp6	NM_011247.1	Mus musculus retinoblastoma binding protein 6 (Rbbp6), mRNA.
scl0019646.2_143-S	94.6895	3.46	Rbbp4	NM_009030.1	Mus musculus retinoblastoma binding protein 4 (Rbbp4), mRNA.
scl066272.3_26-S	94.6672	1.79	1810020G14Rik	NM_025461.2	Mus musculus RIKEN cDNA 1810020G14 gene (1810020G14Rik), mRNA.
scl066340.3_30-S	94.522	1.95	1700023M09Rik	NM_025498.2	Mus musculus RIKEN cDNA 1700023M09 gene (1700023M09Rik), mRNA.
scl078929.1_104-S	94.3587	3.96	Polr3h	NM_030229.2	
scl000096.1_251-S	94.3016	7.69	1600012H06Rik	NM_026451.1	Mus musculus RIKEN cDNA 1600012H06 gene (1600012H06Rik), mRNA.
scl0094279.1_24-S	94.2111	3.86	Sfxn2	NM_053196.2	Mus musculus sideroflexin 2 (Sfxn2), mRNA.
scl093673.1_29-S	94.1543	42.59	Cml2	NM_053096.2	Mus musculus camello-like 2 (Cml2), mRNA.
scl0021843.2_5-S	94.1292	2.25	Tial1	NM_009383.1	Mus musculus Tial1 cytotoxic granule-associated RNA binding protein-like 1 (Tial1), mRNA.
scl24875.11.1_30-S	94.1203	12.19	Taf12	NM_025579.1	Mus musculus TAF12 RNA polymerase II, TATA box binding protein (TBP)-associated factor (Taf12), mRNA.
scl011814.2_27-S	94.03	1.72	Apoc3	NM_023114.2	Mus musculus apolipoprotein C-III (Apoc3), mRNA.
scl0001096.1_118-S	93.9421	4.61	Slco1a4	NM_030687.1	Mus musculus solute carrier organic anion transporter family, member 1a4 (Slco1a4), mRNA.
scl00109624.1_38-S	93.533	18.56	Cald1	NM_145575.1	Mus musculus caldesmon 1 (Cald1), mRNA.
scl44991.2.1_21-S	93.2651	2.05	Hist1h2bc	NM_023422	Mus musculus histone 1, H2bc (Hist1h2bc), mRNA.
scl0002513.1_19-S	93.2353	43.96	C9	NM_013485.1	Mus musculus complement component 9 (C9), mRNA.
scl0001252.1_55-S	92.802	20.97	Hnrpa2b1	NM_016806	Mus musculus heterogeneous nuclear ribonucleoprotein A2/B1 (Hnrpa2b1), transcript variant 2, mRNA.
scl0056349.2_77-S	92.5865	3.56	Net1	NM_019671.1	Mus musculus neuroepithelial cell transforming gene 1 (Net1), mRNA.
scl0099889.1_5-S	92.4892	2.58	Arfip1	XM_130985.2	
scl00215751.1_299-S	92.4155	3.02	BC013529	NM_145418.1	Mus musculus cDNA sequence BC013529 (BC013529), mRNA.
scl018472.2_44-S	92.4148	2.16	Pafah1b1	NM_013625.1	Mus musculus platelet-activating factor acetylhydrolase, isoform 1b, beta1 subunit (Pafah1b1), mRNA.
scl019167.11_85-S	92.3469	1.64	Psma3	NM_011184.2	Mus musculus proteasome (prosome, macropain) subunit, alpha type 3 (Psma3), mRNA.
scl0056628.1_326-S	92.2507	7.25	LOC56628	NM_019909.1	Mus musculus MHC (A.CA/J(H-2K-f) class I antigen (LOC56628), mRNA.
scl0022221.2_82-S	92.1816	14.28	Ubp1	NM_013699.1	Mus musculus upstream binding protein 1 (Ubp1), mRNA.
scl013480.1_38-S	92.0887	6.03	Dpm1	NM_010072.2	Mus musculus dolichol-phosphate (beta-D) mannosyltransferase 1 (Dpm1), mRNA.
scl0004118.1_555-S	91.7441	22.12	Rpo1-3	NM_181730.2	Mus musculus RNA polymerase 1-3 (Rpo1-3), mRNA.
scl36473.24.9_6-S	91.6897	7.12	Usp4	NM_011678.1	Mus musculus ubiquitin specific protease 4 (proto-oncogene) (Usp4), mRNA.

scl0230163.1_72-S	91.6841	4.91	Aldob	NM_144903.2	
scl20357.15.1_26-S	91.6776	7.99	Sqrdl	NM_021507.4	Mus musculus sulfide quinone reductase-like (yeast) (Sqrdl), mRNA.
scl059079.1_41-S	91.6699	7.91	Erbb2ip	XM_358312.1	Mus musculus Erbb2 interacting protein (Erbb2ip), mRNA.
scl0093673.1_94-S	91.5732	1.98	Cml2	NM_053096.2	Mus musculus camello-like 2 (Cml2), mRNA.
scl52515.14.1_211-S	91.4692	2.27	5730455O13Rik	XM_129261.5	Mus musculus RIKEN cDNA 5730455O13 gene (5730455O13Rik), mRNA.
scl24219.2.1_21-S	91.1849	1.58	3110001D03Rik	NM_025849.2	Mus musculus RIKEN cDNA 3110001D03 gene (3110001D03Rik), mRNA.
scl014779.1_116-S	91.0793	1.6	Gpx4	NM_008162	Mus musculus glutathione peroxidase 4 (Gpx4), mRNA.
scl0001816.1_0-S	91.0333	3.34	Eif4a2	XM_147230.1	Mus musculus eukaryotic translation initiation factor 4A2 (Eif4a2), mRNA.
scl0002044.1_26-S	90.7313	1.77	Alg5	NM_025442.1	Mus musculus asparagine-linked glycosylation 5 homolog (yeast, dolichyl-phosphate beta-glucosyltransferase) (Alg5), mRNA.
scl0066440.2_150-S	90.5231	22.13	Cdc26	NM_139291.1	Mus musculus cell division cycle 26 (Cdc26), mRNA.
scl072193.1_10-S	90.5064	2.01	Sfrs2ip	XM_128178.5	
scl0001572.1_0-S	90.1946	1.97	Rai12	NM_018740.1	Mus musculus retinoic acid induced 12 (Rai12), mRNA.
scl50967.4_2-S	90.1313	2.05	0610011F06Rik	XM_128511	Mus musculus RIKEN cDNA 0610011F06 gene (0610011F06Rik), mRNA.
scl011905.7_9-S	89.881	26.33	Serpinc1	NM_080844.2	Mus musculus serine (or cysteine) proteinase inhibitor, clade C (antithrombin), member 1 (Serpinc1), mRNA.
scl22909.7_136-S	89.7897	3.56	4921507I02Rik	XM_131022.4	Mus musculus RIKEN cDNA 4921507I02 gene (4921507I02Rik), mRNA.
scl43097.11.1_34-S	89.7689	15.21	Snapc1	NM_178392.2	Mus musculus small nuclear RNA activating complex, polypeptide 1 (Snapc1), mRNA.
18S_rRNA_X00686_523-S	89.5968	3.14	Pigt	NM_133779.1	Mus musculus phosphatidylinositol glycan, class T (Pigt), mRNA.
scl0066552.1_146-S	89.2254	2.81	2010106G01Rik	NM_023220.1	Mus musculus RIKEN cDNA 2010106G01 gene (2010106G01Rik), mRNA.
scl0002609.1_52-S	89.2089	20.08	Psmb2	NM_011970.2	Mus musculus proteasome (prosome, macropain) subunit, beta type 2 (Psmb2), mRNA.
scl53836.14_500-S	89.1931	19.48	4732479N06Rik	NM_172540.1	Mus musculus RIKEN cDNA 4732479N06 gene (4732479N06Rik), mRNA.
scl020703.2_0-S	89.1345	2.25	Serpina1d	NM_009246.1	Mus musculus serine (or cysteine) proteinase inhibitor, clade A, member 1d (Serpina1d), mRNA.
scl00280487.1_157-S	89.1156	30.35	LOC280487	NM_183299	Mus musculus hypothetical protein LOC280487 (LOC280487), mRNA.
scl22733.6.2_0-S	88.964	2.45	Gstm5	NM_010360.1	Mus musculus glutathione S-transferase, mu 5 (Gstm5), mRNA.
scl50198.3.1_17-S	88.9552	62.08	Ndufb10	XM_128594.4	Mus musculus NADH dehydrogenase (ubiquinone) 1 beta subcomplex, 10 (Ndufb10), mRNA.
scl0026367.1_126-S	88.9511	1.72	Ceacam2	NM_007543.2	Mus musculus CEA-related cell adhesion molecule 2 (Ceacam2), mRNA.
scl022333.10_87-S	88.752	1.52	Vdac1	NM_011694	Mus musculus voltage-dependent anion channel 1 (Vdac1), mRNA.
scl027207.2_8-S	88.7131	1.52	Rps11	XM_133477.1	Mus musculus ribosomal protein S11 (Rps11), mRNA.
scl49992.7.1_104-S	88.5161	21.86	Aif1	NM_019467.2	Mus musculus allograft inflammatory factor 1 (Aif1), mRNA.
scl0272027.1_270-S	88.3407	22.73	BC057893	NM_173033.2	
scl22876.3_595-S	88.3106	1.71	Mcl1	NM_008562.2	Mus musculus myeloid cell leukemia sequence 1 (Mcl1), mRNA.
scl030933.5_3-S	88.2343	2.81	Tor2a	NM_152800.1	Mus musculus torsin family 2, member A (Tor2a), mRNA.
scl23055.15.1_74-S	88.1788	1.61	Plrg1	NM_016784.2	Mus musculus pleiotropic regulator 1, PRL1 homolog (Arabidopsis) (Plrg1), mRNA.
scl0171284.1_49-S	88.1415	3.05	Timd2	NM_134249	Mus musculus T-cell immunoglobulin and mucin domain containing 2 (Timd2), mRNA.
scl066483.1_326-S	88.0664	132.65	Rpl36al	NM_025589.1	Mus musculus ribosomal protein L36a-like (Rpl36al), mRNA.
scl067398.15_77-S	88.0378	2.99	Srpr	NM_026130.1	Mus musculus signal recognition particle receptor (docking protein) (Srpr), mRNA.
scl0003512.1_8-S	87.8081	12.18	Hspa8	NM_031165	Mus musculus heat shock protein 8 (Hspa8), mRNA.
scl50963.5.1_38-S	87.8031	1.56	Mrpl28	NM_024227.2	Mus musculus mitochondrial ribosomal protein L28 (Mrpl28), nuclear gene encoding mitochondrial protein, mRNA.
scl50134.9_8-S	87.7809	11.56	Nudt3	NM_019837.1	Mus musculus nudix (nucleotide diphosphate linked moiety X)-type motif 3 (Nudt3), mRNA.
scl0001683.1_11-S	87.741	2.56	Skiv2l	NM_178062.2	Mus musculus superkiller viralicidic activity 2-like (S. cerevisiae) (Skiv2l), mRNA.
scl41046.4.1_69-S	87.5745	19.14	Cox11	NM_199008.1	
scl067437.2_28-S	87.5522	10.17	Ssr3	NM_026155.1	Mus musculus signal sequence receptor, gamma (Ssr3), mRNA.
scl0022070.1_31-S	87.4038	44.71	Tpt1	NM_009429	Mus musculus tumor protein, translationally-controlled 1 (Tpt1), mRNA.
scl0071900.2_287-S	87.2666	15.07	2310036D22Rik	NM_027992.1	Mus musculus RIKEN cDNA 2310036D22 gene (2310036D22Rik), mRNA.
scl0001320.1_85-S	87.2336	3.42	Mdh1	NM_008618.2	Mus musculus malate dehydrogenase 1, NAD (soluble) (Mdh1), mRNA.
scl52516.7.1_18-S	87.1022	8.73	Rbp4	NM_011255.1	Mus musculus retinol binding protein 4, plasma (Rbp4), mRNA.
scl31909.4.1_14-S	87.0793	1.51	1190003J15Rik	XM_133915.3	Mus musculus RIKEN cDNA 1190003J15 gene (1190003J15Rik), mRNA.
scl066860.1_6-S	86.9086	12.22	1200003E16Rik	XM_130249.3	Mus musculus RIKEN cDNA 1200003E16 gene (1200003E16Rik), mRNA.
scl40141.3_333-S	86.8982	1.6	5730466P16Rik	NM_173453.1	Mus musculus RIKEN cDNA 5730466P16 gene (5730466P16Rik), mRNA.
scl41635.8.225_0-S	86.6317	62.52	Gnb2-rs1	NM_008143.2	Mus musculus guanine nucleotide binding protein, beta 2, related sequence 1 (Gnb2-rs1), mRNA.
scl26678.1.8_16-S	86.5964	1.79	Cno	NM_133724.2	Mus musculus cappuccino (Cno), mRNA.
scl0108143.3_63-S	86.5596	21.87	Taf9	NM_027139.3	Mus musculus TAF9 RNA polymerase II, TATA box binding protein (TBP)-associated factor (Taf9), mRNA.
scl016010.7_252-S	86.5445	1.51	Igfbp4	NM_010517.2	Mus musculus insulin-like growth factor binding protein 4 (Igfbp4), mRNA.
scl25163.8.1_30-S	86.4864	1.54	ORF18	NM_029565.2	Mus musculus open reading frame 18 (ORF18), mRNA.
scl072656.1_14-S	86.3988	24.72	2810013E07Rik	NM_178112.3	Mus musculus RIKEN cDNA 2810013E07 gene (2810013E07Rik), mRNA.
scl26422.6_429-S	86.3879	6.68	1300012D20Rik	NM_152811.1	Mus musculus RIKEN cDNA 1300012D20 gene (1300012D20Rik), mRNA.
scl067941.3_4-S	86.3634	1.51	Rps27l	NM_026467.1	Mus musculus ribosomal protein S27-like (Rps27l), mRNA.

scl0012192.2_207-S	85.8562	4.72	Zfp36l1	NM_007564.2	Mus musculus zinc finger protein 36, C3H type-like 1 (Zfp36l1), mRNA.
scl00107358.2_164-S	85.7609	14.33	Smbp	NM_133352.1	Mus musculus SM-11044 binding protein (Smbp), mRNA.
scl35682.17.1_6-S	85.7308	22.78	Snx1	NM_019727.1	Mus musculus sorting nexin 1 (Snx1), mRNA.
scl26423.7_124-S	85.6687	3.83	AI788959	NM_153598.1	Mus musculus expressed sequence AI788959 (AI788959), mRNA.
scl020818.1_29-S	85.6468	11.82	Srprb	NM_009275.2	Mus musculus signal recognition particle receptor, B subunit (Srprb), mRNA.
scl40921.6_29-S	85.5731	1.59	Igfbp4	NM_010517.2	Mus musculus insulin-like growth factor binding protein 4 (Igfbp4), mRNA.
scl070396.3_74-S	85.509	6.91	2210409M21Rik	NM_133728.2	Mus musculus RIKEN cDNA 2210409M21 gene (2210409M21Rik), mRNA.
scl0012166.1_24-S	85.4545	2.97	Bmpr1a	NM_009758.3	Mus musculus bone morphogenetic protein receptor, type 1A (Bmpr1a), mRNA.
scl0070456.1_86-S	85.2528	1.56	2610205H19Rik	NM_027430.1	Mus musculus RIKEN cDNA 2610205H19 gene (2610205H19Rik), mRNA.
scl0066882.1_105-S	85.2128	2.02	Bzw1	NM_025824.2	Mus musculus basic leucine zipper and W2 domains 1 (Bzw1), mRNA.
scl46370.4_89-S	85.1477	3.52	Apex1	NM_009687	Mus musculus apurinic/apyrimidinic endonuclease 1 (Apex1), mRNA.
scl28573.11_3-S	85.0306	2.97	Crbn	NM_175357.1	
scl33796.16.1_75-S	84.8012	3.13	Aadat	NM_011834.1	
scl0069104.1_104-S	84.6643	2.24	2700055A20Rik	NM_027314.2	
scl066177.1_13-S	84.4699	1.63	Ubl5	NM_025401.1	Mus musculus ubiquitin-like 5 (Ubl5), mRNA.
scl066624.1_138-S	84.4599	22.98	5730406I15Rik	NM_025668.1	Mus musculus RIKEN cDNA 5730406I15 gene (5730406I15Rik), mRNA.
scl0014325.1_328-S	84.4202	3.89	Ftl1	NM_010240.1	Mus musculus ferritin light chain 1 (Ftl1), mRNA.
scl0001180.1_25-S	84.2334	4.22	1200009B18Rik	NM_026168.1	Mus musculus RIKEN cDNA 1200009B18 gene (1200009B18Rik), mRNA.
scl014385.8_306-S	84.2306	6.74	Slc37a4	NM_008063.1	Mus musculus solute carrier family 37 (glycerol-6-phosphate transporter), member 4 (Slc37a4), mRNA.
scl47445.7_14-S	84.2016	18.94	Copz1	NM_019817.1	Mus musculus coatomer protein complex, subunit zeta 1 (Copz1), mRNA.
scl021991.1_200-S	84.1055	1.62	Tpi1	NM_009415.1	
scl067025.2_6-S	83.8796	1.5	Rpl11	NM_025919	Mus musculus ribosomal protein L11 (Rpl11), mRNA.
scl36531.21.1_19-S	83.7126	2.22	5730439E10Rik	NM_175324.2	Mus musculus RIKEN cDNA 5730439E10 gene (5730439E10Rik), mRNA.
scl0003079.1_4-S	83.6358	2.61	Grb14	NM_016719.1	Mus musculus growth factor receptor bound protein 14 (Grb14), mRNA.
scl31009.10_67-S	83.626	1.54	Dgat2	NM_026384.2	Mus musculus diacylglycerol O-acyltransferase 2 (Dgat2), mRNA.
scl24880.7_98-S	83.4248	3	Sfrs4	NM_020587.1	Mus musculus splicing factor, arginine/serine-rich 4 (SRp75) (Sfrs4), mRNA.
scl0003137.1_53-S	83.3779	2.2	Drbp1	NM_178090.2	Mus musculus developmentally regulated RNA binding protein 1 (Drbp1), mRNA.
scl0074252.2_11-S	83.331	15.55	Armc1	NM_028840.2	
scl41277.14_78-S	83.1708	12.97	AW550801	NM_177325	Mus musculus expressed sequence AW550801 (AW550801), mRNA.
scl0020054.1_111-S	83.1364	1.7	Rps15	NM_009091.1	Mus musculus ribosomal protein S15 (Rps15), mRNA.
scl080795.5_227-S	83.0946	1.75	Selk	NM_019979.1	Mus musculus selenoprotein K (Selk), mRNA.
scl20206.6_190-S	83.0925	15.41	Dstn	NM_019771.1	Mus musculus destrin (Dstn), mRNA.
scl013138.1_18-S	82.9203	2.37	Dag1	NM_010017.1	Mus musculus dystroglycan 1 (Dag1), mRNA.
scl020701.2_3-S	82.8678	2.28	Serpina1b	NM_009244.2	Mus musculus serine (or cysteine) proteinase inhibitor, clade A, member 1b (Serpina1b), mRNA.
scl0001214.1_23-S	82.8158	12.21	Slc25a13	NM_015829.1	Mus musculus solute carrier family 25 (mitochondrial carrier; adenine nucleotide translocator), member 13 (Slc25a13), mRNA.
scl0003132.1_48-S	82.8005	7.15	0610009I22Rik	NM_025646.2	Mus musculus RIKEN cDNA 0610009I22 gene (0610009I22Rik), mRNA.
scl23667.4_0-S	82.6471	1.89	Pnrc2	NM_026383.1	Mus musculus proline-rich nuclear receptor coactivator 2 (Pnrc2), mRNA.
scl45536.7.1_0-S	82.6355	13.66	Tm9sf1	NM_028780.2	Mus musculus transmembrane 9 superfamily member 1 (Tm9sf1), mRNA.
scl018984.16_308-S	82.6212	2.65	Por	NM_008898.1	Mus musculus P450 (cytochrome) oxidoreductase (Por), mRNA.
scl34858.4_85-S	82.5716	6.75	Slc25a4	XM_134169.4	Mus musculus solute carrier family 25 (mitochondrial carrier; adenine nucleotide translocator), member 4 (Slc25a4), mRNA.
scl0218333.1_228-S	82.5404	3.72	BC018507	XM_358313.1	Mus musculus cDNA sequence BC018507 (BC018507), mRNA.
scl0021974.1_259-S	82.4734	17.42	Top2b	NM_009409.2	Mus musculus topoisomerase (DNA) II beta (Top2b), mRNA.
scl014319.1_33-S	82.3969	1.66	Fth1	NM_010239.1	
scl51108.6.3_0-S	82.3769	1.71	Sod2	NM_013671.2	Mus musculus superoxide dismutase 2, mitochondrial (Sod2), mRNA.
scl0002518.1_2-S	82.2853	15.04	Mrpl13	NM_026759.2	Mus musculus mitochondrial ribosomal protein L13 (Mrpl13), mRNA.
scl18935.13_58-S	82.2765	9.85	Cat	NM_009804	Mus musculus catalase (Cat), mRNA.
scl018703.11_156-S	82.0705	1.88	Pigr	NM_011082.2	Mus musculus polymeric immunoglobulin receptor (Pigr), mRNA.
scl0097112.2_117-S	82.0685	2.7	C87860	NM_133787.1	Mus musculus expressed sequence C87860 (C87860), mRNA.
scl17685.8.520_25-S	82.0528	3.47	Spp2	NM_029269.1	
scl52081.14_14-S	81.9897	1.74	Ik	NM_011879.1	Mus musculus IK cytokine (Ik), mRNA.
scl33163.3.1_18-S	81.9302	1.74	1810063B05Rik	NM_174987.2	Mus musculus RIKEN cDNA 1810063B05 gene (1810063B05Rik), mRNA.
scl0002578.1_9-S	81.8994	1.51	Eif3s6ip	NM_145139.1	Mus musculus eukaryotic translation initiation factor 3, subunit 6 interacting protein (Eif3s6ip), mRNA.
scl32101.7.1_5-S	81.8147	3.55	D7Wsu128e	NM_138589	Mus musculus DNA segment, Chr 7, Wayne State University 128, expressed (D7Wsu128e), mRNA.
scl0223631.4_104-S	81.6768	1.64	BC025446	NM_146058.1	Mus musculus cDNA sequence BC025446 (BC025446), mRNA.
scl28784.6_160-S	81.5564	1.65	Tex261	NM_009357.1	Mus musculus testis expressed gene 261 (Tex261), mRNA.
scl26403.11.1_10-S	81.3938	5.61	Gc	XM_132170.2	Mus musculus group specific component (Gc), mRNA.

scl011430.3_98-S	81.3113	24.82	Acox1	NM_015729	Mus musculus acyl-Coenzyme A oxidase 1, palmitoyl (Acox1), mRNA.
scl0002225.1_0-S	81.3085	14.6	Srp19	NM_025527.1	Mus musculus signal recognition particle 19 (Srp19), mRNA.
scl0066849.1_145-S	81.2467	2.09	Ppp1r2	NM_025800.1	Mus musculus protein phosphatase 1, regulatory (inhibitor) subunit 2 (Ppp1r2), mRNA.
scl0012116.2_283-S	81.0775	12.29	Bhmt	NM_016668.2	Mus musculus betaine-homocysteine methyltransferase (Bhmt), mRNA.
scl24398.4.1_27-S	81.0734	1.68	Hint2	XM_143732.2	
scl40603.7.49_6-S	80.9414	1.65	Drg1	NM_007879.1	Mus musculus developmentally regulated GTP binding protein 1 (Drg1), mRNA.
scl0022608.1_233-S	80.8666	1.49	Nsep1	NM_011732.1	Mus musculus nuclease sensitive element binding protein 1 (Nsep1), mRNA.
scl0001631.1_196-S	80.7895	12.56	Rpo1-1	NM_009085.1	Mus musculus RNA polymerase 1-1 (Rpo1-1), mRNA.
scl36904.3.10_19-S	80.7816	1.74	Cox5a	NM_007747	Mus musculus cytochrome c oxidase, subunit Va (Cox5a), mRNA.
scl19951.7_95-S	80.5669	2.06	Ywhab	NM_018753.3	Mus musculus tyrosine 3-monooxygenase/tryptophan 5-monooxygenase activation protein, beta polypeptide (Ywhab), mRNA.
scl52979.12_115-S	80.5424	1.71	Pdcd4	NM_011050	Mus musculus programmed cell death 4 (Pdcd4), mRNA.
scl0053413.2_143-S	80.3505	2.14	Exoc7	NM_016857.1	Mus musculus exocyst complex component 7 (Exoc7), mRNA.
scl46000.3_481-S	80.2517	2.69	Ndfip2	NM_029561.1	Mus musculus Nedd4 family interacting protein 2 (Ndfip2), mRNA.
scl0218100.1_36-S	80.2256	14.84	Zfp322a	NM_172586.2	Mus musculus zinc finger protein 322a (Zfp322a), mRNA.
scl38525.3.714_1-S	80.0521	13.01	Ube2n	NM_080560.2	Mus musculus ubiquitin-conjugating enzyme E2N (Ube2n), mRNA.
scl42858.13_56-S	79.8387	2.14	Rin3	NM_177620.2	Mus musculus Ras and Rab interactor 3 (Rin3), mRNA.
scl012825.37_20-S	79.7209	23.99	Col3a1	NM_009930.1	Mus musculus procollagen, type III, alpha 1 (Col3a1), mRNA.
scl40005.7.1_7-S	79.5194	1.8	Rai12	NM_018740.1	Mus musculus retinoic acid induced 12 (Rai12), mRNA.
scl0067921.2_259-S	79.4608	1.78	2510010F15Rik	NM_026454.2	Mus musculus RIKEN cDNA 2510010F15 gene (2510010F15Rik), mRNA.
scl000061.1_90_REVCOM	79.4531	4.42	Nme7	NM_138314.1	Mus musculus non-metastatic cells 7, protein expressed in (Nme7), mRNA.
scl068193.4_35-S	79.435	1.48	Rpl24	NM_024218.2	Mus musculus ribosomal protein L24 (Rpl24), mRNA.
scl00104681.2_288-S	79.377	6.41	Slc16a6	NM_134038.1	Mus musculus solute carrier family 16 (monocarboxylic acid transporters), member 6 (Slc16a6), mRNA.
scl0116891.1_127-S	79.333	2.81	BC005682	NM_033562.2	Mus musculus cDNA sequence BC005682 (BC005682), mRNA.
scl50160.5.1_23-S	79.3069	1.53	BC008155	NM_145410.1	Mus musculus cDNA sequence BC008155 (BC008155), mRNA.
scl000709.1_10-S	79.2408	7.62	Fbxo8	NM_015791.2	Mus musculus F-box only protein 8 (Fbxo8), mRNA.
scl42461.3_0-S	79.081	19.25	Cfl2	NM_007688.1	Mus musculus cofilin 2, muscle (Cfl2), mRNA.
scl013680.2_48-S	78.8301	3.48	Ddx19	NM_007916.1	Mus musculus DEAD (Asp-Glu-Ala-Asp) box polypeptide 19 (Ddx19), mRNA.
scl54140.7.1_25-S	78.804	5.01	Dnase1l1	NM_027109.1	
scl16210.3.1_71-S	78.6121	2.26	Cfhl1	NM_015780.1	Mus musculus complement component factor h-like 1 (Cfhl1), mRNA.
scl23680.13_149-S	78.5662	1.82	1110007C24Rik	NM_025382.3	Mus musculus RIKEN cDNA 1110007C24 gene (1110007C24Rik), mRNA.
scl18994.12.1_7-S	78.5461	1.55	F2	NM_010168.1	Mus musculus coagulation factor II (F2), mRNA.
scl0104771.9_30-S	78.4277	1.8	1200003C05Rik	NM_024205.1	Mus musculus RIKEN cDNA 1200003C05 gene (1200003C05Rik), mRNA.
scl0003772.1_20-S	78.3204	2.1	Pex7	NM_008822.1	Mus musculus peroxisome biogenesis factor 7 (Pex7), mRNA.
scl46082.11_140-S	78.31	11.96	Tgfb1i4	NM_009366.1	Mus musculus transforming growth factor beta 1 induced transcript 4 (Tgfb1i4), mRNA.
scl018139.26_3-S	78.275	1.71	Zfml	NM_008717.1	
scl0002689.1_21-S	78.2584	2.31	2210021A15Rik	NM_173719.1	Mus musculus RIKEN cDNA 2210021A15 gene (2210021A15Rik), mRNA.
scl067130.2_3-S	78.1684	13.97	Ndufa6	NM_025987.1	Mus musculus NADH dehydrogenase (ubiquinone) 1 alpha subcomplex, 6 (B14) (Ndufa6), mRNA.
scl50689.6_424-S	78.0741	1.66	Slc35b1	XM_128634.4	Mus musculus solute carrier family 35, member B1 (Slc35b1), mRNA.
scl0066310.1_231-S	77.9983	1.75	2810410M20Rik	NM_024428.2	Mus musculus RIKEN cDNA 2810410M20 gene (2810410M20Rik), mRNA.
scl0002381.1_12-S	77.8602	1.71	Dhrs7	NM_025522.1	Mus musculus dehydrogenase/reductase (SDR family) member 7 (Dhrs7), mRNA.
scl46398.42.1_4-S	77.8342	13.24	Ktn1	NM_008477	Mus musculus kinectin 1 (Ktn1), mRNA.
scl38289.2_239-S	77.7949	1.75	Apof	NM_133997.1	Mus musculus apolipoprotein F (Apof), mRNA.
scl50399.22_481-S	77.7654	3.93	Ttc7	NM_028639	Mus musculus tetratricopeptide repeat domain 7 (Ttc7), mRNA.
scl17134.7_259-S	77.6811	2.73	BC031781	NM_145943.1	Mus musculus cDNA sequence BC031781 (BC031781), mRNA.
scl33150.17.997_2-S	77.625	1.64	Itgb1	NM_010578.1	Mus musculus integrin beta 1 (fibronectin receptor beta) (Itgb1), mRNA.
scl020700.1_2-S	77.6011	10.85	Serpina1a	NM_009243	Mus musculus serine (or cysteine) proteinase inhibitor, clade A, member 1a (Serpina1a), mRNA.
scl45531.6.1_0-S	77.5446	1.53	Nedd8	NM_008683	Mus musculus neural precursor cell expressed, developmentally down-regulated gene 8 (Nedd8), mRNA.
scl0055946.2_3-S	77.5389	24.03	Ap3m1	NM_018829.2	Mus musculus adaptor-related protein complex 3, mu 1 subunit (Ap3m1), mRNA.
scl015473.1_6-S	77.4828	48.22	Hrsp12	NM_008287.2	Mus musculus heat-responsive protein 12 (Hrsp12), mRNA.
scl36601.18.1_52-S	77.3969	4.11	Atr	XM_356175.1	
scl078325.1_150-S	77.2667	1.59	2700092H06Rik	XM_132529.2	Mus musculus RIKEN cDNA 2700092H06 gene (2700092H06Rik), mRNA.
scl20056.4.1_63-S	77.009	1.7	Map1lc3a	NM_025735.1	Mus musculus microtubule-associated protein 1 light chain 3 alpha (Map1lc3a), mRNA.
scl0003820.1_78-S	76.9718	2.29	Chpt1	NM_144807.2	Mus musculus choline phosphotransferase 1 (Chpt1), mRNA.
scl48807.5.1_32-S	76.8107	4.95	Magmas	NM_025571.1	Mus musculus mitochondria-associated protein involved in granulocyte-macrophage colony-stimulating factor signal transduction (Magmas), mRNA.
scl40203.12_25-S	76.7583	16.02	Sparc	NM_009242	Mus musculus secreted acidic cysteine rich glycoprotein (Sparc), mRNA.
scl0269261.4_12-S	76.7463	2.32	Rpl12	NM_009076	Mus musculus ribosomal protein L12 (Rpl12), mRNA.

scl28628.30_135-S	76.7366	43.12	Frmd4b	NM_145148.1	
scl023837.2_17-S	76.6998	1.76	Cfdp1	NM_011801.1	
scl24987.27.1_4-S	76.67	16.49	Inpp5b	NM_008385.3	Mus musculus inositol polyphosphate-5-phosphatase B (Inpp5b), mRNA.
scl34540.19_229-S	76.575	1.64	Vps35	NM_022997	Mus musculus vacuolar protein sorting 35 (Vps35), mRNA.
scl0003896.1_252-S	76.5693	1.76	Igf1	NM_010512	Mus musculus insulin-like growth factor 1 (Igf1), mRNA.
scl32630.18_440-S	76.5564	2.22	Hrmt1l3	NM_133740.1	Mus musculus heterogeneous nuclear ribonucleoprotein methyltransferase-like 3 (S. cerevisiae) (Hrmt1l3), mRNA.
scl44323.5.1_4-S	76.5153	2.71	Paip1	NM_145457.2	Mus musculus polyadenylate binding protein-interacting protein 1 (Paip1), mRNA.
scl013095.9_315-S	76.4187	36.02	Cyp2c29	NM_007815	Mus musculus cytochrome P450, family 2, subfamily c, polypeptide 29 (Cyp2c29), mRNA.
scl31074.15.1_32-S	76.3341	1.46	Fah	NM_010176.1	Mus musculus fumarylacetoacetate hydrolase (Fah), mRNA.
scl021665.1_179-S	76.0794	13.48	Tdg	NM_172552.1	Mus musculus thymine DNA glycosylase (Tdg), mRNA.
scl014050.16_119-S	75.9731	12.19	Eya3	NM_010166.2	Mus musculus eyes absent 3 homolog (Drosophila) (Eya3), transcript variant 2, mRNA.
scl46440.4.1_161-S	75.9494	2.08	Lrrc21	NM_146245.1	Mus musculus leucine rich repeat containing 21 (Lrrc21), mRNA.
scl44635.2.1_29-S	75.9124	3.58	Mrpl36	NM_053163.1	Mus musculus mitochondrial ribosomal protein L36 (Mrpl36), mRNA.
scl0067288.2_208-S	75.8775	12.24	3110031B13Rik	NM_026075.1	Mus musculus RIKEN cDNA 3110031B13 gene (3110031B13Rik), mRNA.
scl51564.13.1_155-S	75.7449	1.46	Proc	NM_008934.1	Mus musculus protein C (Proc), mRNA.
scl0012870.2_25-S	75.6171	16.12	Cp	NM_007752.1	Mus musculus ceruloplasmin (Cp), mRNA.
scl0012453.1_227-S	75.5599	15.77	Ccni	NM_017367.2	Mus musculus cyclin I (Ccni), mRNA.
scl0019684.1_297-S	75.5546	13.89	Rdx	NM_009041.1	Mus musculus radixin (Rdx), mRNA.
scl38374.10.1_58-S	75.3363	1.48	0610007H07Rik	NM_026617.1	Mus musculus RIKEN cDNA 0610007H07 gene (0610007H07Rik), mRNA.
scl53686.6.11_16-S	75.2535	1.49	Prdx4	NM_016764.1	Mus musculus peroxiredoxin 4 (Prdx4), mRNA.
scl0019943.2_84-S	75.1324	1.47	Rpl28	NM_009081.1	Mus musculus ribosomal protein L28 (Rpl28), mRNA.
scl27078.4.1_81-S	75.0718	1.62	0910001L09Rik	XM_132434.2	Mus musculus RIKEN cDNA 0910001L09 gene (0910001L09Rik), mRNA.
scl29751.3_94-S	75.0174	2.83	Klf15	NM_023184.2	Mus musculus Kruppel-like factor 15 (Klf15), mRNA.
scl34487.14.1_100-S	74.9927	51.72	Ces1	NM_021456.1	Mus musculus carboxylesterase 1 (Ces1), mRNA.
scl0067184.2_306-S	74.8743	1.92	Grim19	NM_023312.1	Mus musculus genes associated with retinoid-IFN-induced mortality 19 (Grim19), mRNA.
scl50027.4.1_35-S	74.6916	36.87	Psmb9	NM_013585.1	Mus musculus proteosome (prosome, macropain) subunit, beta type 9 (large multifunctional protease 2) (Psmb9), mRNA.
scl31392.8.1_11-S	74.6249	1.49	Fcgrt	NM_010189	Mus musculus Fc receptor, IgG, alpha chain transporter (Fcgrt), mRNA.
scl54257.9.1_23-S	74.5268	16.04	Gpc4	NM_008150.1	Mus musculus glypican 4 (Gpc4), mRNA.
scl012558.4_4-S	74.518	14.61	Cdh2	NM_007664.1	Mus musculus cadherin 2 (Cdh2), mRNA.
scl014007.1_13-S	74.4124	4.15	Cugbp2	NM_010160.1	Mus musculus CUG triplet repeat,RNA binding protein 2 (Cugbp2), mRNA.
scl50155.9_2-S	74.3249	11.83	Decr2	NM_011933.1	Mus musculus 2-4-dienoyl-Coenzyme A reductase 2, peroxisomal (Decr2), mRNA.
scl068552.1_11-S	74.2968	1.84	1110003E01Rik	NM_133697.1	Mus musculus RIKEN cDNA 1110003E01 gene (1110003E01Rik), mRNA.
scl000857.1_11-S	74.2033	1.46	Hsd11b1	NM_008288.1	Mus musculus hydroxysteroid 11-beta dehydrogenase 1 (Hsd11b1), mRNA.
scl0230126.1_281-S	74.1679	25.52	Shb	XM_131399.4	Mus musculus src homology 2 domain-containing transforming protein B (Shb), mRNA.
scl0019224.1_106-S	74.0659	8	Ptgs1	NM_008969.1	Mus musculus prostaglandin-endoperoxide synthase 1 (Ptgs1), mRNA.
scl0107686.2_24-S	73.9878	1.63	Snrpd2	XM_133225.1	Mus musculus small nuclear ribonucleoprotein D2 (Snrpd2), mRNA.
scl0014390.1_51-S	73.9095	14.07	Gabpa	NM_008065.2	Mus musculus GA repeat binding protein, alpha (Gabpa), mRNA.
scl0012928.2_60-S	73.8695	13.12	Crk	NM_133656.2	Mus musculus v-crk sarcoma virus CT10 oncogene homolog (avian) (Crk), mRNA.
scl000669.1_31-S	73.8513	1.49	Gpsn2	NM_134118.1	Mus musculus glycoprotein, synaptic 2 (Gpsn2), mRNA.
scl066144.2_329-S	73.8248	1.49	Atp6v1f	NM_025381	Mus musculus ATPase, H+ transporting, V1 subunit F (Atp6v1f), mRNA.
scl34673.5.1_43-S	73.763	2.01	Bst2	NM_198095.1	
scl42484.15.73_1-S	73.7317	12.48	Strn3	NM_052973.1	Mus musculus striatin, calmodulin binding protein 3 (Strn3), mRNA.
scl013132.15_5-S	73.6441	2.28	Dab2	NM_023118.1	Mus musculus disabled homolog 2 (Drosophila) (Dab2), mRNA.
scl000929.1_30-S	73.4205	2.54	Ugt1a6	NM_145079	Mus musculus UDP glycosyltransferase 1 family, polypeptide A6 (Ugt1a6), mRNA.
scl18254.7.1_9-S	73.408	2.4	Ctsz	NM_022325.3	Mus musculus cathepsin Z (Ctsz), mRNA.
scl00021.1_10-S	73.2381	2.36	Apoc1	NM_007469.2	Mus musculus apolipoprotein C-I (Apoc1), mRNA.
scl0016158.2_239-S	73.2123	2.04	Il11ra2	NM_010550	Mus musculus interleukin 11 receptor, alpha chain 2 (Il11ra2), mRNA.
scl52549.14_1-S	73.1793	1.78	Atad1	NM_026487.2	Mus musculus ATPase family, AAA domain containing 1 (Atad1), mRNA.
scl0014857.1_108-S	73.1048	23.18	Gsta1	NM_008181.1	Mus musculus glutathione S-transferase, alpha 1 (Ya) (Gsta1), mRNA.
scl012457.3_248-S	72.8743	19.59	Ccrn4l	NM_009834.1	Mus musculus CCR4 carbon catabolite repression 4-like (S. cerevisiae) (Ccrn4l), mRNA.
scl018521.14_30-S	72.8216	1.46	Pcbp2	NM_011042.1	Mus musculus poly(rC) binding protein 2 (Pcbp2), mRNA.
scl19291.9.1_25-S	72.7544	17.18	Nmi	NM_019401.1	Mus musculus N-myc (and STAT) interactor (Nmi), mRNA.
scl020922.5_83-S	72.5611	2.58	Supt4h	NM_009296.1	Mus musculus suppressor of Ty 4 homolog (S. cerevisiae) (Supt4h), mRNA.
scl18631.16_116-S	72.433	15.22	Slc23a2	NM_018824.2	Mus musculus solute carrier family 23 (nucleobase transporters), member 2 (Slc23a2), mRNA.
scl000377.1_101-S	72.3315	1.71	Adk	NM_134079.1	Mus musculus adenosine kinase (Adk), mRNA.
scl0394435.7_126-S	72.1855	1.45	Ugt1a9	NM_201410	

scl19566.6.1_18-S	71.882	2.09	Dpp7	NM_031843.2	Mus musculus dipeptidylpeptidase 7 (Dpp7), mRNA.
scl33841.11_453-S	71.8758	1.83	Irf2	NM_008391.2	Mus musculus interferon regulatory factor 2 (Irf2), mRNA.
scl31001.17.1_88-S	71.8724	1.66	Slco2b1	NM_175316.2	Mus musculus solute carrier organic anion transporter family, member 2b1 (Slco2b1), mRNA.
scl0059043.2_22-S	71.8659	1.65	Wsb2	NM_021539.3	Mus musculus WD repeat and SOCS box-containing 2 (Wsb2), mRNA.
scl21598.9.1_53-S	71.8629	2.95	Snx7	XM_131162.3	Mus musculus sorting nexin 7 (Snx7), mRNA.
scl0056392.1_211-S	71.7316	2.09	Shoc2	NM_019658.2	Mus musculus soc-2 (suppressor of clear) homolog (C. elegans) (Shoc2), mRNA.
scl022218.1_29-S	71.5582	1.46	Sumo1	NM_009460.1	
scl0020455.1_36-S	71.5139	2.47	Sif1		
scl19560.8.1_32-S	71.5118	1.81	1700013L23Rik	XM_130127.3	Mus musculus RIKEN cDNA 1700013L23 gene (1700013L23Rik), mRNA.
scl068011.3_46-S	71.5047	1.49	Snrpg	NM_026506.1	Mus musculus small nuclear ribonucleoprotein polypeptide G (Snrpg), mRNA.
scl011813.3_68-S	71.4959	1.72	Apoc2	NM_009695.2	Mus musculus apolipoprotein C-II (Apoc2), mRNA.
scl0022218.1_0-S	71.3992	2.82	Sumo1	NM_009460.1	
scl42244.15_187-S	71.3922	2.36	Aldh6a1	NM_134042.1	Mus musculus aldehyde dehydrogenase family 6, subfamily A1 (Aldh6a1), mRNA.
scl00228829.1_258-S	71.3048	12.97	6820402O20Rik	XM_133187.4	Mus musculus RIKEN cDNA 6820402O20 gene (6820402O20Rik), mRNA.
scl35908.8_513-S	71.2544	4.45	4432416J03Rik	NM_030069	Mus musculus RIKEN cDNA 4432416J03 gene (4432416J03Rik), mRNA.
scl31737.12_81-S	71.2309	4.3	2810007J24Rik	NM_175250.3	Mus musculus RIKEN cDNA 2810007J24 gene (2810007J24Rik), mRNA.
scl068576.4_100-S	71.2026	2.05	Hbxip	XM_131076.1	
scl026912.9_26-S	71.168	2.25	Gcat	NM_013847	Mus musculus glycine C-acetyltransferase (2-amino-3-ketobutyrate-coenzyme A ligase) (Gcat), mRNA.
scl0011652.2_199-S	71.0001	1.77	Akt2	NM_007434.2	Mus musculus thymoma viral proto-oncogene 2 (Akt2), mRNA.
scl29907.19_469-S	70.9612	3.57	Eif2ak3	NM_010121.1	Mus musculus eukaryotic translation initiation factor 2 alpha kinase 3 (Eif2ak3), mRNA.
scl36033.13_6-S	70.8846	1.52	Ei24	NM_007915	Mus musculus etoposide induced 2.4 mRNA (Ei24), mRNA.
scl0019205.2_0-S	70.8483	1.59	Ptbp1	NM_008956.1	Mus musculus polypyrimidine tract binding protein 1 (Ptbp1), mRNA.
scl0003189.1_25-S	70.8145	1.48	2010323F13Rik	NM_177157.2	Mus musculus RIKEN cDNA 2010323F13 gene (2010323F13Rik), mRNA.
scl30008.5_1-S	70.7384	2.45	Aqp1	NM_007472.1	Mus musculus aquaporin 1 (Aqp1), mRNA.
scl27178.12.102_2-S	70.4289	1.48	Cct6a	NM_009838	Mus musculus chaperonin subunit 6a (zeta) (Cct6a), mRNA.
scl0067151.2_31-S	70.4154	6.81	Psmd9	NM_026000.1	Mus musculus proteasome (prosome, macropain) 26S subunit, non-ATPase, 9 (Psmd9), mRNA.
scl00268301.1_190-S	70.4061	4.79	4921515A04Rik	NM_172939.2	Mus musculus RIKEN cDNA 4921515A04 gene (4921515A04Rik), mRNA.
scl24979.20.1_9-S	70.3888	2.37	Gnl2	NM_145552	
scl30756.4.28_1-S	70.3864	2.13	Coq7	NM_009940.1	Mus musculus demethyl-Q 7 (Coq7), mRNA.
scl0013195.2_156-S	70.3664	2.46	Ddc	NM_016672.1	Mus musculus dopa decarboxylase (Ddc), mRNA.
scl016155.8_10-S	70.1493	5.06	Il10rb	NM_008349.1	Mus musculus interleukin 10 receptor, beta (Il10rb), mRNA.
scl011844.1_12-S	70.0836	1.86	Arf5	NM_007480.1	Mus musculus ADP-ribosylation factor 5 (Arf5), mRNA.
scl019073.1_109-S	70.0813	11.11	Prg	NM_011157.1	
scl22663.19.1_25-S	69.9451	27.62	Bcar3	NM_013867.1	Mus musculus breast cancer anti-estrogen resistance 3 (Bcar3), mRNA.
scl44313.2.1_9-S	69.8855	2.43	Akr1c12	NM_013777.1	Mus musculus aldo-keto reductase family 1, member C12 (Akr1c12), mRNA.
scl0002744.1_1-S	69.6105	1.78	Zfyve9	XM_131643.4	
scl0269399.1_1-S	69.5727	10.84	6720461J16Rik	NM_175551.2	Mus musculus RIKEN cDNA 6720461J16 gene (6720461J16Rik), mRNA.
scl20633.35.1_246-S	69.3662	1.87	Lrp4	NM_172668.2	Mus musculus low density lipoprotein receptor-related protein 4 (Lrp4), mRNA.
scl067270.3_15-S	69.2118	1.58	D10Ertd322e	NM_026065.2	
scl000898.1_12-S	69.122	1.77	Ivns1abp	NM_054102.1	Mus musculus influenza virus NS1A binding protein (Ivns1abp), mRNA.
scl45103.13_79-S	69.1164	11.92	9030611N15Rik	NM_134072.1	Mus musculus RIKEN cDNA 9030611N15 gene (9030611N15Rik), mRNA.
scl0001439.1_101-S	69.0049	2.43	Ube2g1	NM_025985.2	Mus musculus ubiquitin-conjugating enzyme E2G 1 (UBC7 homolog, C. elegans) (Ube2g1), mRNA.
scl52798.4.1_101-S	68.9799	2.07	Nudt8	NM_025529.2	Mus musculus nudix (nucleoside diphosphate linked moiety X)-type motif 8 (Nudt8), mRNA.
scl0065111.1_63-S	68.969	1.47	Dap3	NM_022994.2	Mus musculus death associated protein 3 (Dap3), mRNA.
scl52741.7_234-S	68.7982	2.32	1110006I15Rik	NM_134142.1	Mus musculus RIKEN cDNA 1110006I15 gene (1110006I15Rik), mRNA.
scl16257.7.73_24-S	68.7509	1.58	Timm17a	NM_011590.1	Mus musculus translocator of inner mitochondrial membrane 17a (Timm17a), mRNA.
scl0022034.1_201-S	68.7119	2.6	Traf6	NM_009424.2	Mus musculus Tnf receptor-associated factor 6 (Traf6), mRNA.
scl00103694.2_279-S	68.7111	2.25	1110014L17Rik	NM_134020.1	Mus musculus RIKEN cDNA 1110014L17 gene (1110014L17Rik), mRNA.
scl076938.2_0-S	68.5676	1.86	Rbm17	NM_152824.1	Mus musculus RNA binding motif protein 17 (Rbm17), mRNA.
scl00319263.1_175-S	68.5651	10.93	A030012M09Rik	NM_183028.1	Mus musculus RIKEN cDNA A030012M09 gene (A030012M09Rik), mRNA.
scl21950.8.1_214-S	68.4295	1.49	Mtx1	NM_013604.1	Mus musculus metaxin 1 (Mtx1), mRNA.
scl53770.17.1_35-S	68.2234	4.12	Acsl4	NM_207625.1	Mus musculus acyl-CoA synthetase long-chain family member 4 (Acsl4), transcript variant 2, mRNA.
scl0021763.1_228-S	68.2161	1.73	Tex2	NM_198292.2	Mus musculus testis expressed gene 2 (Tex2), mRNA.
scl45748.3_30-S	68.036	3.32	2610018L09Rik	NM_172254.2	
scl28314.7.1_50-S	68.0166	4	2010012C16Rik	NM_025564.1	Mus musculus RIKEN cDNA 2010012C16 gene (2010012C16Rik), mRNA.
scl0013046.1_235-S	67.941	1.99	Cugbp1	NM_198683.1	Mus musculus CUG triplet repeat, RNA binding protein 1 (Cugbp1), transcript variant 2, mRNA.

scl054624.14_263-S	67.6697	1.95	5730511K23Rik	NM_019458.2	Mus musculus RIKEN cDNA 5730511K23 gene (5730511K23Rik), mRNA.
scl54158.7.21_44-S	67.4874	32.03	Bcap31	NM_012060.2	Mus musculus B-cell receptor-associated protein 31 (Bcap31), mRNA.
scl0106583.1_2-S	67.3919	3.97	Rbm16	NM_134123.2	Mus musculus RNA binding motif protein 16 (Rbm16), mRNA.
scl45100.10.1_14-S	67.3054	42.17	Akr1c6	NM_030611.1	Mus musculus aldo-keto reductase family 1, member C6 (Akr1c6), mRNA.
scl35083.10.1_128-S	67.2963	15.96	Grtp1	NM_025768.1	Mus musculus GH regulated TBC protein 1 (Grtp1), mRNA.
scl057808.2_82-S	67.2876	3.68	Rpl35a	NM_021338.2	Mus musculus ribosomal protein L35a (Rpl35a), mRNA.
scl20520.4_157-S	67.1897	11.03	0610012H03Rik	NM_028747.1	Mus musculus RIKEN cDNA 0610012H03 gene (0610012H03Rik), mRNA.
scl35429.19.1_59-S	67.0611	1.59	1300017J02Rik	NM_027918.1	Mus musculus RIKEN cDNA 1300017J02 gene (1300017J02Rik), mRNA.
scl0001099.1_185-S	66.964	12.72	St7	NM_022332.1	Mus musculus suppression of tumorigenicity 7 (St7), mRNA.
scl45771.22.1_206-S	66.8976	1.69	Itih1	NM_008406.1	Mus musculus inter-alpha trypsin inhibitor, heavy chain 1 (Itih1), mRNA.
scl41761.12.1_9-S	66.8797	9.93	Fancl	NM_025923.2	Mus musculus Fanconi anemia, complementation group L (Fancl), mRNA.
scl16803.9_203-S	66.8479	1.46	Slc40a1	NM_016917.1	Mus musculus solute carrier family 40 (iron-regulated transporter), member 1 (Slc40a1), mRNA.
scl0002326.1_69-S	66.7615	6.26	Rab10	NM_016676.2	Mus musculus RAB10, member RAS oncogene family (Rab10), mRNA.
scl27877.10.4_15-S	66.5863	2.69	Lyar	NM_025281.1	Mus musculus Ly1 antibody reactive clone (Lyar), mRNA.
scl43154.2_481-S	66.5454	1.82	Arf6	NM_007481.1	Mus musculus ADP-ribosylation factor 6 (Arf6), mRNA.
scl0002742.1_56-S	66.5302	2.64	Slc31a1	NM_175090	Mus musculus solute carrier family 31, member 1 (Slc31a1), mRNA.
scl36120.9.10_29-S	66.5019	13.4	Acp5	NM_007388.1	Mus musculus acid phosphatase 5, tartrate resistant (Acp5), mRNA.
scl26971.4.541_0-S	66.4295	1.79	Rpo1-3	NM_009087.1	Mus musculus RNA polymerase 1-3 (Rpo1-3), mRNA.
scl077219.10_12-S	66.3659	2.52	Zadh1	NM_029880.1	
scl055949.3_17-S	66.3618	1.74	Eef1b2	NM_018796.2	Mus musculus eukaryotic translation elongation factor 1 beta 2 (Eef1b2), mRNA.
scl40848.1_563-S	66.3146	2.91	Clp1	NM_138753.1	Mus musculus cardiac lineage protein 1 (Clp1), mRNA.
scl19106.4.1_16-S	66.0496	11.65	Fkbp7	NM_010222.1	Mus musculus FK506 binding protein 7 (Fkbp7), mRNA.
scl055960.6_109-S	65.8453	12.51	Ebag9	NM_019480.3	Mus musculus estrogen receptor-binding fragment-associated gene 9 (Ebag9), mRNA.
scl50495.21.3_143-S	65.8009	7.75	2610511O17Rik	NM_152817.2	Mus musculus RIKEN cDNA 2610511O17 gene (2610511O17Rik), mRNA.
scl066878.10_113-S	65.5871	1.48	Riok3	NM_024182.2	Mus musculus RIO kinase 3 (yeast) (Riok3), mRNA.
scl014113.7_42-S	65.58	1.55	Fbl	NM_007991.2	Mus musculus fibrillarin (Fbl), mRNA.
scl46290.12.1_1-S	65.5606	1.82	1500001L15Rik	XM_127738.3	Mus musculus RIKEN cDNA 1500001L15 gene (1500001L15Rik), mRNA.
scl0066212.1_0-S	65.5184	2.67	Sec61b	NM_024171.1	Mus musculus Sec61 beta subunit (Sec61b), mRNA.
scl0017692.2_146-S	65.4085	10.32	Msl31	NM_010832.2	Mus musculus male-specific lethal-3 homolog 1 (Drosophila) (Msl31), mRNA.
scl011848.5_0-S	65.3929	1.87	Rhoa	NM_016802	Mus musculus ras homolog gene family, member A (Rhoa), mRNA.
scl0211660.12_302-S	65.3652	6.74	LOC211660	XM_129366.4	
scl41198.10_516-S	65.352	1.44	Poldip2	NM_026389.2	Mus musculus polymerase (DNA-directed), delta interacting protein 2 (Poldip2), mRNA.
scl00040.1_6-S	65.3151	5.33	Mef2a	NM_013597.2	Mus musculus myocyte enhancer factor 2A (Mef2a), transcript variant 1, mRNA.
scl25159.19.1_9-S	65.3037	3	2810475A17Rik	NM_028355.1	Mus musculus RIKEN cDNA 2810475A17 gene (2810475A17Rik), mRNA.
scl18252.2_52-S	65.286	62.72	Atp5e	NM_025983.2	Mus musculus ATP synthase, H+ transporting, mitochondrial F1 complex, epsilon subunit (Atp5e), mRNA.
scl00223455.2_26-S	65.1513	2.31	F830029L24Rik	NM_172606.1	Mus musculus RIKEN cDNA F830029L24 gene (F830029L24Rik), mRNA.
scl0067452.2_1-S	64.974	1.55	1200006O19Rik	NM_026164.1	Mus musculus RIKEN cDNA 1200006O19 gene (1200006O19Rik), mRNA.
scl30499.14.1_7-S	64.9485	1.42	Rnh1	NM_145135.2	Mus musculus ribonuclease/angiogenin inhibitor 1 (Rnh1), mRNA.
scl19759.21.1_156-S	64.8867	1.6	2610031L17Rik	NM_133701.1	Mus musculus RIKEN cDNA 2610031L17 gene (2610031L17Rik), mRNA.
scl0066105.2_68-S	64.7426	3.14	Ube2d3	NM_025356.2	Mus musculus ubiquitin-conjugating enzyme E2D 3 (UBC4/5 homolog, yeast) (Ube2d3), mRNA.
scl066190.1_22-S	64.7069	3.03	Phca	NM_025408.1	Mus musculus phytoceramidase, alkaline (Phca), mRNA.
scl23040.14.1_27-S	64.63	2.34	Pet112l	NM_144896.2	Mus musculus PET112-like (yeast) (Pet112l), mRNA.
scl35678.16.1_29-S	64.5016	2.02	Usp3	NM_144937.2	Mus musculus ubiquitin specific protease 3 (Usp3), mRNA.
scl052840.5_2-S	64.3388	1.71	D2Bwg0891e	NM_026797.1	Mus musculus DNA segment, Chr 2, Brigham & Womens Genetics 0891 expressed (D2Bwg0891e), mRNA.
scl014109.3_67-S	64.3148	1.57	Fau	NM_007990	Mus musculus Finkel-Biskis-Reilly murine sarcoma virus (FBR-MuSV) ubiquitously expressed (fox derived) (Fau), mRNA.
scl0059069.1_170-S	64.2895	2.39	Tpm3	NM_022314.2	Mus musculus tropomyosin 3, gamma (Tpm3), mRNA.
scl38416.13.1_1-S	64.2881	2.37	Ptprb	NM_029928.1	Mus musculus protein tyrosine phosphatase, receptor type, B (Ptprb), mRNA.
scl066302.10_6-S	64.2698	13.6	2410005O16Rik	NM_025476.3	Mus musculus RIKEN cDNA 2410005O16 gene (2410005O16Rik), mRNA.
scl0014682.1_7-S	64.2437	1.8	Gnaq	NM_008139.2	Mus musculus guanine nucleotide binding protein, alpha q polypeptide (Gnaq), mRNA.
scl0076846.1_142-S	64.2186	1.52	3010033P07Rik	NM_029767.1	
scl066989.6_1-S	64.2043	2.49	2410004N11Rik	NM_025888.2	Mus musculus RIKEN cDNA 2410004N11 gene (2410004N11Rik), mRNA.
scl20194.20.1_79-S	64.1904	9.21	Sec23b	NM_019787.2	Mus musculus SEC23B (S. cerevisiae) (Sec23b), mRNA.
scl19661.4.1_12-S	64.132	1.85	Ptpla	NM_013935.1	Mus musculus protein tyrosine phosphatase-like (proline instead of catalytic arginine), member a (Ptpla), mRNA.
scl0001193.1_40-S	64.1312	13.65	Pon3	NM_173006.1	Mus musculus paraoxonase 3 (Pon3), mRNA.
scl0321022.1_67-S	64.1028	1.47	Cdv3	NM_175833.1	Mus musculus carnitine deficiency-associated gene expressed in ventricle 3 (Cdv3), mRNA.
scl00103850.1_287-S	64.0594	1.58	Nt5m	NM_134029.1	Mus musculus 5,3-nucleotidase, mitochondrial (Nt5m), mRNA.

scl018416.10_18-S	64.0071	5.83	Otc	NM_008769.2	Mus musculus ornithine transcarbamylase (Otc), mRNA.
scl34701.2.1_7-S	63.9302	1.99	2810428I15Rik	XM_147329.1	Mus musculus RIKEN cDNA 2810428I15 gene (2810428I15Rik), mRNA.
scl41548.2.100_36-S	63.9253	1.64	Hint1	NM_008248.1	Mus musculus histidine triad nucleotide binding protein 1 (Hint1), mRNA.
scl52961.19_173-S	63.8648	6.99	Add3	NM_013758.2	Mus musculus adducin 3 (gamma) (Add3), mRNA.
scl068767.10_282-S	63.8226	6	ORF19	XM_128709.4	Mus musculus open reading frame 19 (ORF19), mRNA.
scl00109054.1_86-S	63.8055	4.51	Pfdn4	XM_355370	Mus musculus prefoldin 4 (Pfdn4), mRNA.
scl0002310.1_66-S	63.7907	1.67	1110057K04Rik	NM_172401.2	Mus musculus RIKEN cDNA 1110057K04 gene (1110057K04Rik), mRNA.
scl0001779.1_17-S	63.7708	1.98	Eif4a2	NM_013506	Mus musculus eukaryotic translation initiation factor 4A2 (Eif4a2), mRNA.
scl0002028.1_25-S	63.6832	1.52	2310066N05Rik	NM_027371.1	Mus musculus RIKEN cDNA 2310066N05 gene (2310066N05Rik), mRNA.
scl0106338.2_0-S	63.6519	12.34	Nsun3	NM_178925.2	
scl19394.13_183-S	63.6019	4.48	Ggta1	NM_010283.1	Mus musculus glycoprotein galactosyltransferase alpha 1, 3 (Ggta1), mRNA.
scl0067443.1_283-S	63.4993	2.92	Map1lc3b	NM_026160	Mus musculus microtubule-associated protein 1 light chain 3 beta (Map1lc3b), mRNA.
scl0003171.1_33-S	63.3331	1.86	Psmb7	NM_011187.1	Mus musculus proteasome (prosome, macropain) subunit, beta type 7 (Psmb7), mRNA.
scl32562.20.1_76-S	63.2912	1.62	1600012K10Rik	NM_025905.2	Mus musculus RIKEN cDNA 1600012K10 gene (1600012K10Rik), mRNA.
scl000978.1_0-S	63.1123	1.53	Cops5	NM_013715.1	Mus musculus COP9 (constitutive photomorphogenic) homolog, subunit 5 (Arabidopsis thaliana) (Cops5), mRNA.
scl0017841.1_127-S	63.0985	12.07	Mup2	NM_008647	Mus musculus major urinary protein 2 (Mup2), mRNA.
scl0002188.1_58-S	63.0931	11.92	Riok3	NM_024182.2	Mus musculus RIO kinase 3 (yeast) (Riok3), mRNA.
scl31573.3_3-S	63.0612	1.47	Mrps12	NM_011885	Mus musculus mitochondrial ribosomal protein S12 (Mrps12), mRNA.
scl48556.8.1_18-S	63.0437	1.47	2010319C14Rik	NM_024464.2	Mus musculus RIKEN cDNA 2010319C14 gene (2010319C14Rik), mRNA.
scl34607.9_25-S	63.0139	2.42	Smad1	NM_008539.3	
scl27364.5.1_30-S	62.9681	1.57	Sfrs9	NM_025573.2	Mus musculus splicing factor, arginine/serine rich 9 (Sfrs9), mRNA.
scl33788.4.1_0-S	62.9535	1.66	BC009118	NM_145595.1	Mus musculus cDNA sequence BC009118 (BC009118), mRNA.
scl0065246.1_38-S	62.9508	6.14	Xpo7	NM_023045.1	Mus musculus exportin 7 (Xpo7), mRNA.
scl19516.4.19_7-S	62.8706	2.09	Surf1	NM_013677.1	Mus musculus surfeit gene 1 (Surf1), mRNA.
scl066935.3_28-S	62.8565	3.21	1700023B02Rik	NM_025854.1	Mus musculus RIKEN cDNA 1700023B02 gene (1700023B02Rik), mRNA.
scl016432.2_9-S	62.7792	1.41	Itm2b	NM_008410.1	Mus musculus integral membrane protein 2B (Itm2b), mRNA.
scl0015481.2_296-S	62.7218	2.4	Hspa8	NM_031165.3	Mus musculus heat shock protein 8 (Hspa8), mRNA.
scl25435.3.1_50-S	62.6505	10.99	Nipsnap3b	NM_025623.1	
scl0235072.13_139-S	62.6025	14.86	39332	NM_009859.2	Mus musculus septin 7 (Sept7), mRNA.
scl00394432.2_137-S	62.4709	1.44	Ugt1a10	NM_201642	
scl31512.7.1_16-S	62.4385	1.46	5033425B17Rik	NM_027215	Mus musculus RIKEN cDNA 5033425B17 gene (5033425B17Rik), mRNA.
scl53365.18.1_112-S	62.4175	1.43	Prp19	NM_134129.2	Mus musculus PRP19/PSO4 homolog (S. cerevisiae) (Prp19), mRNA.
scl018648.4_76-S	62.2341	49.45	Pgam1	NM_023418.1	Mus musculus phosphoglycerate mutase 1 (Pgam1), mRNA.
scl50670.2.184_270-S	62.1709	1.51	2310039H08Rik	NM_025966.2	Mus musculus RIKEN cDNA 2310039H08 gene (2310039H08Rik), mRNA.
scl0076055.1_48-S	61.9075	5.57	Mgea5	NM_023799.2	Mus musculus meningioma expressed antigen 5 (hyaluronidase) (Mgea5), mRNA.
scl00268448.1_163-S	61.904	13.45	Phf12	NM_174852.2	Mus musculus PHD finger protein 12 (Phf12), mRNA.
scl0015130.1_284-S	61.81	7.53	Hbb-b2	NM_016956.2	Mus musculus hemoglobin, beta adult minor chain (Hbb-b2), mRNA.
scl31589.11.1_58-S	61.7718	1.55	Psmc4	NM_011874.1	Mus musculus proteasome (prosome, macropain) 26S subunit, ATPase, 4 (Psmc4), mRNA.
scl069470.6_81-S	61.7691	1.99	2310003P10Rik	NM_175145.2	Mus musculus RIKEN cDNA 2310003P10 gene (2310003P10Rik), mRNA.
scl027681.7_4-S	61.7549	2.65	D11Moh34	NM_033568.1	Mus musculus DNA segment, Chr 11, KL Mohlke 34 (D11Moh34), mRNA.
scl49610.9.216_11-S	61.7365	1.68	Fez2	NM_199448.1	Mus musculus fasciculation and elongation protein zeta 2 (zygin II) (Fez2), mRNA.
scl20006.8_238-S	61.6998	4.2	2610304G08Rik	NM_027434	Mus musculus RIKEN cDNA 2610304G08 gene (2610304G08Rik), mRNA.
scl017836.34_27-S	61.5092	2.06	Mug1	NM_008645	Mus musculus murinoglobulin 1 (Mug1), mRNA.
scl0067845.2_211-S	61.471	15.02	Zfp364	NM_026406.2	Mus musculus zinc finger protein 364 (Zfp364), mRNA.
scl49994.7.1_30-S	61.3466	1.45	Apom	NM_018816	Mus musculus apolipoprotein M (Apom), mRNA.
scl066594.3_10-S	61.3361	1.71	Uqcr	NM_025650.1	Mus musculus ubiquinol-cytochrome c reductase (6.4kD) subunit (Uqcr), mRNA.
scl0076281.1_330-S	61.3218	1.66	Tax1bp3	NM_029564	
scl00009.1_36-S	61.315	1.53	Vti1b	NM_016800.2	Mus musculus arginase type II (Arg2), mRNA.
scl0014528.1_45-S	61.2978	1.84	Gch1	NM_008102.2	
scl067938.1_193-S	61.2792	1.52	Mylc2b	NM_023402.1	
scl014897.1_33-S	61.2483	1.94	Trip12	NM_133975.2	
scl00230257.2_191-S	61.2357	11.6	Rod1	NM_144904.1	Mus musculus ROD1 regulator of differentiation 1 (S. pombe) (Rod1), mRNA.
scl0072193.1_269-S	61.1832	4.01	Sfrs2ip	XM_128178.5	
scl45482.13_126-S	61.1291	2.54	1110008L20Rik	NM_028643.1	Mus musculus RIKEN cDNA 1110008L20 gene (1110008L20Rik), mRNA.
scl49249.30_34-S	61.1286	7.1	Dlgh1	NM_007862.2	Mus musculus discs, large homolog 1 (Drosophila) (Dlgh1), mRNA.
scl16734.10.1_6-S	60.89	37.64	Clk1	NM_009905.1	

scl0170768.1_23-S	60.8859	2.9	Pfkfb3	NM_172976.1	Mus musculus 6-phosphofructo-2-kinase/fructose-2,6-biphosphatase 3 (Pfkfb3), mRNA.
scl018477.6_2-S	60.7606	1.4	Prdx1	NM_011034.2	Mus musculus peroxiredoxin 1 (Prdx1), mRNA.
scl068024.2_210-S	60.759	63.64	Hist1h2bc	NM_023422.1	Mus musculus histone 1, H2bc (Hist1h2bc), mRNA.
scl37421.7_120-S	60.7366	6.57	Rassf3	NM_138956	Mus musculus Ras association (RalGDS/AF-6) domain family 3 (Rassf3), mRNA.
scl000646.1_33-S	60.7362	2.04	2310061C15Rik	NM_026844.2	
scl0001267.1_72-S	60.662	5.4	Drg1	NM_007879.1	Mus musculus developmentally regulated GTP binding protein 1 (Drg1), mRNA.
scl023980.1_2-S	60.6343	1.97	Pbp	NM_018858.1	Mus musculus phosphatidylethanolamine binding protein (Pbp), mRNA.
scl071750.18_235-S	60.6008	17.22	1300003K24Rik	NM_027900.2	Mus musculus RIKEN cDNA 1300003K24 gene (1300003K24Rik), mRNA.
scl48561.1.13_8-S	60.5449	1.73	0610012G03Rik	NM_025320	Mus musculus RIKEN cDNA 0610012G03 gene (0610012G03Rik), mRNA.
scl47211.15_135-S	60.5325	13.07	Rad21	NM_009009.2	Mus musculus RAD21 homolog (S. pombe) (Rad21), mRNA.
scl066979.1_124-S	60.4163	3.48	Pole4	NM_025882.1	Mus musculus polymerase (DNA-directed), epsilon 4 (p12 subunit) (Pole4), mRNA.
scl067788.3_30-S	60.2846	14.5	6330577E15Rik	NM_026377.1	Mus musculus RIKEN cDNA 6330577E15 gene (6330577E15Rik), mRNA.
scl47686.9.1_164-S	60.2039	3.29	Cyp2d9	NM_010006.1	Mus musculus cytochrome P450, family 2, subfamily d, polypeptide 9 (Cyp2d9), mRNA.
scl41592.12.1_36-S	60.0901	2.1	2900006B13Rik	NM_028398.1	Mus musculus RIKEN cDNA 2900006B13 gene (2900006B13Rik), mRNA.
scl22700.8.1_2-S	60.0391	3.87	Olfm3	NM_153157.1	Mus musculus olfactomedin 3 (Olfm3), transcript variant B, mRNA.
scl22078.18_184-S	59.9763	7.63	Kpna4	NM_008467.2	Mus musculus karyopherin (importin) alpha 4 (Kpna4), mRNA.
scl28293.6.1_86-S	59.8521	1.58	Hebp1	NM_013546.1	Mus musculus heme binding protein 1 (Hebp1), mRNA.
scl25480.9.1_148-S	59.7757	1.46	Grhpr	NM_080289.1	Mus musculus glyoxylate reductase/hydroxypyruvate reductase (Grhpr), mRNA.
scl0067225.1_69-S	59.7297	11.43	2810441O16Rik	NM_026043.2	Mus musculus RIKEN cDNA 2810441O16 gene (2810441O16Rik), mRNA.
scl067332.3_15-S	59.7296	1.63	Snrpd3	NM_026095.3	Mus musculus small nuclear ribonucleoprotein D3 (Snrpd3), mRNA.
scl0066691.1_167-S	59.7258	1.52	4432404J10Rik	NM_025709.2	Mus musculus RIKEN cDNA 4432404J10 gene (4432404J10Rik), mRNA.
scl00108687.2_201-S	59.7009	1.44	9530090G24Rik	NM_145537.1	Mus musculus RIKEN cDNA 9530090G24 gene (9530090G24Rik), mRNA.
scl067876.4_28-S	59.6625	3.31	1500041J02Rik	NM_026424.2	Mus musculus RIKEN cDNA 1500041J02 gene (1500041J02Rik), mRNA.
scl0026900.1_130-S	59.6283	8.54	Ddx3y	NM_012008.1	Mus musculus DEAD (Asp-Glu-Ala-Asp) box polypeptide 3, Y-linked (Ddx3y), mRNA.
scl20901.11.1_2-S	59.503	2.34	Upp2	NM_029692.1	Mus musculus uridine phosphorylase 2 (Upp2), mRNA.
scl011480.11_306-S	59.4363	13.17	Acvr2	NM_007396.2	Mus musculus activin receptor IIA (Acvr2), mRNA.
scl056334.4_3-S	59.3974	1.78	1110032D12Rik	NM_019770	Mus musculus RIKEN cDNA 1110032D12 gene (1110032D12Rik), mRNA.
scl54170.6.1_1-S	59.2813	6.53	Cetn2	NM_019405.5	Mus musculus centrin 2 (Cetn2), mRNA.
scl40305.32_46-S	59.2267	4.68	Cyfip2	XM_147526.1	Mus musculus cytoplasmic FMR1 interacting protein 2 (Cyfip2), mRNA.
scl000094.1_33_REVCOM	59.0709	1.58	Mea1	NM_010787.1	Mus musculus male enhanced antigen 1 (Mea1), mRNA.
scl46904.9_7-S	59.0131	1.48	Poldip3	NM_178627.1	Mus musculus polymerase (DNA-directed), delta interacting protein 3 (Poldip3), mRNA.
scl017992.3_16-S	58.9186	1.4	Ndufa4	NM_010886.1	Mus musculus NADH dehydrogenase (ubiquinone) 1 alpha subcomplex, 4 (Ndufa4), mRNA.
scl27967.7.1_149-S	58.9119	1.6	Abhd1	NM_021304.2	Mus musculus abhydrolase domain containing 1 (Abhd1), mRNA.
scl019683.5_201-S	58.8294	2.16	Rdh6	NM_009040.1	Mus musculus retinol dehydrogenase 6 (Rdh6), mRNA.
scl022791.10_3-S	58.7772	12.58	Dnajc2	NM_009583	Mus musculus DnaJ (Hsp40) homolog, subfamily C, member 2 (Dnajc2), mRNA.
scl0330401.1_15-S	58.7668	4.14	3632431M01Rik	XM_132843.5	Mus musculus RIKEN cDNA 3632431M01 gene (3632431M01Rik), mRNA.
scl056282.5_222-S	58.7562	1.5	Mrpl12	NM_027204.2	Mus musculus mitochondrial ribosomal protein L12 (Mrpl12), mRNA.
scl22131.3_16-S	58.7522	13.55	D3Ucla1	NM_030685.1	Mus musculus DNA segment, Chr 3, University of California at Los Angeles 1 (D3Ucla1), mRNA.
scl0012859.2_116-S	58.7322	1.39	Cox5b	NM_009942.1	Mus musculus cytochrome c oxidase, subunit Vb (Cox5b), mRNA.
scl0067166.2_227-S	58.7149	1.8	Arl10c	NM_026011.2	Mus musculus ADP-ribosylation factor-like 10C (Arl10c), mRNA.
scl30978.11_3-S	58.6914	2.6	Plekhb1	NM_013746.1	Mus musculus pleckstrin homology domain containing, family B (evectins) member 1 (Plekhb1), mRNA.
scl20555.7.24_72-S	58.6863	2.09	Mmrp19	NM_019735.2	
scl068501.6_11-S	58.6506	3.27	1110014D18Rik	NM_026746.1	Mus musculus RIKEN cDNA 1110014D18 gene (1110014D18Rik), mRNA.
scl015107.1_301-S	58.6379	2.92	Hadhsc	NM_008212.1	Mus musculus L-3-hydroxyacyl-Coenzyme A dehydrogenase, short chain (Hadhsc), mRNA.
scl50795.8.1_75-S	58.6193	9.65	Ddah2	NM_016765	Mus musculus dimethylarginine dimethylaminohydrolase 2 (Ddah2), mRNA.
scl0214063.1_118-S	58.5996	6.04	4732437J24Rik	NM_172338.1	Mus musculus RIKEN cDNA 4732437J24 gene (4732437J24Rik), mRNA.
scl41231.2_247-S	58.4485	3.31	1300007F04Rik	NM_026185.1	Mus musculus RIKEN cDNA 1300007F04 gene (1300007F04Rik), mRNA.
scl020655.4_35-S	58.3569	1.49	Sod1	NM_011434.1	Mus musculus superoxide dismutase 1, soluble (Sod1), mRNA.
scl52209.6.1_16-S	58.3477	2.6	Ttr	NM_013697.1	Mus musculus transthyretin (Ttr), mRNA.
scl000090.1_16-S	58.3248	3.86	Tcfl4	NM_011550.2	Mus musculus transcription factor-like 4 (Tcfl4), mRNA.
scl29242.30.1_27-S	58.2945	10.11	Cadps2	NM_153163.2	Mus musculus Ca2+-dependent activator protein for secretion 2 (Cadps2), mRNA.
scl070510.9_205-S	58.2798	1.56	5730408C10Rik	NM_027445.1	
scl26445.6.1_1-S	58.2098	3.15	Igfbp7	NM_008048.1	Mus musculus insulin-like growth factor binding protein 7 (Igfbp7), mRNA.
scl0069654.1_290-S	58.0753	1.42	Dctn2	NM_027151.1	Mus musculus dynactin 2 (Dctn2), mRNA.
scl056367.1_256-S	58.0678	4.45	Scoc	XM_134455.1	Mus musculus short coiled-coil protein (Scoc), mRNA.
scl38496.5_152-S	58.0424	3.36	Dusp6	NM_026268.1	Mus musculus dual specificity phosphatase 6 (Dusp6), mRNA.

scl37947.10_418-S	58.0088	2.02	Tde2	NM_019760	Mus musculus tumor differentially expressed 2 (Tde2), mRNA.
scl083921.9_127-S	57.9796	11.83	Tmem2	NM_031997.2	Mus musculus transmembrane protein 2 (Tmem2), mRNA.
scl0027999.2_249-S	57.9621	1.65	D6Wsu176e	NM_138587.3	Mus musculus DNA segment, Chr 6, Wayne State University 176, expressed (D6Wsu176e), mRNA.
scl0001372.1_16-S	57.757	3.12	Clk4	NM_007714.2	Mus musculus CDC like kinase 4 (Clk4), mRNA.
scl018245.5_1-S	57.5971	1.67	Oaz1	NM_008753	Mus musculus ornithine decarboxylase antizyme (Oaz1), mRNA.
scl0012499.2_107-S	57.4802	1.61	Entpd5	NM_007647.1	Mus musculus ectonucleoside triphosphate diphosphohydrolase 5 (Entpd5), mRNA.
scl44728.4.1_7-S	57.436	6.72	2400003B06Rik	NM_026211.1	Mus musculus RIKEN cDNA 2400003B06 gene (2400003B06Rik), mRNA.
scl020068.3_1-S	57.2101	1.47	Rps17	NM_009092.2	Mus musculus ribosomal protein S17 (Rps17), mRNA.
scl51823.8.1_71-S	57.1557	13.61	Tnfsf5ip1	NM_134138.1	Mus musculus tumor necrosis factor superfamily, member 5-induced protein 1 (Tnfsf5ip1), mRNA.
scl26866.19_111-S	57.0733	3.51	Phtf2	NM_172992.2	Mus musculus putative homeodomain transcription factor 2 (Phtf2), mRNA.
scl27087.3.5_17-S	57.0318	1.57	Azgp1	NM_013478.1	Mus musculus alpha-2-glycoprotein 1, zinc (Azgp1), mRNA.
scl51245.13.1_81-S	57.0016	11.8	Ccdc5	NM_146089.1	Mus musculus coiled-coil domain containing 5 (Ccdc5), mRNA.
scl0002327.1_0-S	56.987	10.5	Mgea6	NM_146034.1	Mus musculus meningioma expressed antigen 6 (coiled-coil proline-rich) (Mgea6), mRNA.
scl31978.3.3_6-S	56.9121	10.97	5730458D16Rik	NM_177609	Mus musculus RIKEN cDNA 5730458D16 gene (5730458D16Rik), mRNA.
scl36548.17_86-S	56.8262	1.49	Ryk	NM_013649.2	Mus musculus receptor-like tyrosine kinase (Ryk), mRNA.
scl39079.4_283-S	56.8047	4.37	Ctgf	NM_010217	Mus musculus connective tissue growth factor (Ctgf), mRNA.
scl067180.1_177-S	56.7892	13.84	2610311I19Rik	NM_023311.2	Mus musculus RIKEN cDNA 2610311I19 gene (2610311I19Rik), mRNA.
scl37580.8_154-S	56.7862	1.41	Nudt4	NM_027722.2	Mus musculus nudix (nucleoside diphosphate linked moiety X)-type motif 4 (Nudt4), mRNA.
scl019951.1_25-S	56.7108	2.19	Rpl32	NM_172086	Mus musculus ribosomal protein L32 (Rpl32), mRNA.
scl00232087.1_95-S	56.6408	4.15	Mat2a	NM_145569	Mus musculus methionine adenosyltransferase II, alpha (Mat2a), mRNA.
scl50503.16.1_7-S	56.5631	4.02	Spg4	NM_016962	Mus musculus spastic paraplegia 4 homolog (human) (Spg4), mRNA.
scl22658.17.1_65-S	56.5476	3.3	Sec24d	NM_027135.2	Mus musculus SEC24 related gene family, member D (S. cerevisiae) (Sec24d), mRNA.
scl33261.5_41-S	56.5257	1.61	Hsbp1	NM_024219.1	Mus musculus heat shock factor binding protein 1 (Hsbp1), mRNA.
scl0013548.2_260-S	56.4909	8.76	Dyrk1a	NM_007890.1	Mus musculus dual-specificity tyrosine-(Y)-phosphorylation regulated kinase 1a (Dyrk1a), mRNA.
scl0012301.2_7-S	56.4848	28.12	Cacybp	NM_009786.1	Mus musculus calcyclin binding protein (Cacybp), mRNA.
scl43180.27.1_0-S	56.4784	1.39	Mgea6	NM_146034.1	Mus musculus meningioma expressed antigen 6 (coiled-coil proline-rich) (Mgea6), mRNA.
scl29903.3_37-S	56.47	1.98	AA792894	NM_145568.2	Mus musculus EST AA792894 (AA792894), mRNA.
scl0003902.1_2-S	56.4406	3.85	Snx3	NM_017472.2	Mus musculus sorting nexin 3 (Snx3), mRNA.
scl36686.18.1_100-S	56.4227	36.86	Gclc	NM_010295.1	Mus musculus glutamate-cysteine ligase, catalytic subunit (Gclc), mRNA.
scl022629.4_2-S	56.4081	9.71	Ywhah	NM_011738	Mus musculus tyrosine 3-monooxygenase/tryptophan 5-monooxygenase activation protein, eta polypeptide (Ywhah), mRNA.
scl38510.3.1_11-S	56.3316	12.13	Lum	NM_008524	Mus musculus lumican (Lum), mRNA.
scl40399.3_141-S	56.2791	12.36	2510006C20Rik	NM_026527.1	Mus musculus RIKEN cDNA 2510006C20 gene (2510006C20Rik), mRNA.
scl017835.20_21-S	56.2043	1.86	Mug-ps1		
scl0011737.2_249-S	56.0654	1.59	Anp32a	NM_009672.2	Mus musculus acidic (leucine-rich) nuclear phosphoprotein 32 family, member A (Anp32a), mRNA.
scl0056298.2_141-S	56.0215	2.32	Arl6ip2	NM_019717.1	Mus musculus ADP-ribosylation factor-like 6 interacting protein 2 (Arl6ip2), mRNA.
scl0114128.8_210-S	56.0073	18.92	Laptm4b	NM_033521.2	Mus musculus lysosomal-associated protein transmembrane 4B (Laptm4b), mRNA.
scl000987.1_58-S	56	11.72	Ifi203	NM_008328.1	Mus musculus interferon activated gene 203 (Ifi203), mRNA.
scl46489.21.1_17-S	55.924	2.6	Capn7	NM_009796.1	Mus musculus calpain 7 (Capn7), mRNA.
scl0003048.1_4-S	55.8624	5.81	Abl1	NM_009594.1	Mus musculus v-abl Abelson murine leukemia oncogene 1 (Abl1), mRNA.
scl40985.2.1_86-S	55.8437	2.63	Hoxb4	NM_010459	Mus musculus homeo box B4 (Hoxb4), mRNA.
scl25550.21.1_18-S	55.8244	2.63	Aco1	NM_007386.1	Mus musculus aconitase 1 (Aco1), mRNA.
scl37827.3_399-S	55.7602	1.64	D10Ertd214e	NM_134007.2	Mus musculus DNA segment, Chr 10, ERATO Doi 214, expressed (D10Ertd214e), mRNA.
scl0018569.1_4-S	55.732	1.51	Pdcd4	NM_011050.1	Mus musculus programmed cell death 4 (Pdcd4), mRNA.
scl26137.12_266-S	55.7247	1.49	2400003N08Rik	NM_178622.3	Mus musculus RIKEN cDNA 2400003N08 gene (2400003N08Rik), mRNA.
scl46938.9_3-S	55.7052	2.12	Slc25a17	NM_011399.1	Mus musculus solute carrier family 25 (mitochondrial carrier; peroxisomal membrane protein), member 17 (Slc25a17), mRNA.
scl26063.14.1_2-S	55.6617	1.38	Hpd	NM_008277.1	Mus musculus 4-hydroxyphenylpyruvic acid dioxygenase (Hpd), mRNA.
scl022129.32_83-S	55.625	7.28	Ttc3	NM_009441.1	Mus musculus tetratricopeptide repeat domain 3 (Ttc3), mRNA.
scl0001718.1_14-S	55.6168	1.99	Mylc2b	NM_023402.1	
scl070652.1_1-S	55.5236	10.78	5730537D05Rik	NM_027495.2	Mus musculus RIKEN cDNA 5730537D05 gene (5730537D05Rik), mRNA.
scl0066291.2_269-S	55.4696	1.84	1810030N24Rik	NM_025471.1	Mus musculus RIKEN cDNA 1810030N24 gene (1810030N24Rik), mRNA.
scl011816.1_11-S	55.2936	1.55	Apoe	NM_009696.2	Mus musculus apolipoprotein E (Apoe), mRNA.
scl22415.7_11-S	55.2659	1.45	Pxmp3	NM_008994.2	Mus musculus peroxisomal membrane protein 3 (Pxmp3), mRNA.
scl0066365.1_89-S	55.166	1.81	2310015N07Rik	NM_025515.1	Mus musculus RIKEN cDNA 2310015N07 gene (2310015N07Rik), mRNA.
scl0018537.2_170-S	55.1572	4.53	Pcmt1	NM_008786.1	Mus musculus protein-L-isoaspartate (D-aspartate) O-methyltransferase 1 (Pcmt1), mRNA.
scl21064.31_2-S	55.1566	1.6	5830434P21Rik	NM_172661.2	Mus musculus RIKEN cDNA 5830434P21 gene (5830434P21Rik), mRNA.
scl0110796.1_34-S	55.1546	1.52	Sdccag33	XM_129060.3	Mus musculus serologically defined colon cancer antigen 33 (Sdccag33), mRNA.

scl011544.1_20-S	55.1358	1.59	Adprh	NM_007414.2	Mus musculus ADP-ribosylarginine hydrolase (Adprh), mRNA.
scl0067732.1_253-S	55.0406	21.18	4833421E05Rik	NM_026347.2	Mus musculus RIKEN cDNA 4833421E05 gene (4833421E05Rik), mRNA.
scl16789.34_226-S	54.9855	2.35	Myo1b	NM_010863.1	Mus musculus myosin IB (Myo1b), mRNA.
scl0027364.1_305-S	54.937	1.64	Srr	NM_013761.2	Mus musculus serine racemase (Srr), mRNA.
scl0100732.7_1-S	54.93	7.06	Mapre3	NM_133350.1	Mus musculus microtubule-associated protein, RP/EB family, member 3 (Mapre3), mRNA.
scl0053872.2_123-S	54.9208	2.32	Gpiap1	NM_016739.2	Mus musculus GPI-anchored membrane protein 1 (Gpiap1), mRNA.
scl0054725.2_225-S	54.9055	2.91	Igsf4a	NM_018770.2	Mus musculus immunoglobulin superfamily, member 4A (Igsf4a), transcript variant 3, mRNA.
scl54119.26.1_18-S	54.8979	2.33	F8	NM_007977.1	Mus musculus coagulation factor VIII (F8), mRNA.
scl0070599.2_259-S	54.8975	4.34	Ssfa2	NM_080558.3	Mus musculus sperm specific antigen 2 (Ssfa2), mRNA.
scl54907.8_703-S	54.815	1.51	F9	XM_135893.2	Mus musculus coagulation factor IX (F9), mRNA.
scl016865.11_15-S	54.7917	2.68	Lgtn	NM_010709.1	Mus musculus ligatin (Lgtn), mRNA.
scl0020973.1_244-S	54.7345	1.67	Syngr2	NM_009304.1	Mus musculus synaptogyrin 2 (Syngr2), mRNA.
scl42842.7.1_0-S	54.6559	2.55	D12Ertd647e	NM_026790.1	Mus musculus DNA segment, Chr 12, ERATO Doi 647, expressed (D12Ertd647e), transcript variant 1, mRNA.
scl45278.7.1_11-S	54.5828	13.29	Dnajd1	NM_025384.3	Mus musculus DnaJ (Hsp40) homolog, subfamily D, member 1 (Dnajd1), mRNA.
scl19203.9.1_0-S	54.5758	2.88	Grb14	NM_016719.1	Mus musculus growth factor receptor bound protein 14 (Grb14), mRNA.
scl0207683.7_143-S	54.5554	3.88	Igsf11	NM_170599.2	Mus musculus immunoglobulin superfamily, member 11 (Igsf11), mRNA.
scl50961.11_474-S	54.5519	1.68	Axin	XM_128515.4	
scl36793.6.1_16-S	54.537	1.88	Ppib	NM_011149.1	Mus musculus peptidylprolyl isomerase B (Ppib), mRNA.
scl066656.2_23-S	54.469	1.4	Eef1d	NM_023240.1	Mus musculus eukaryotic translation elongation factor 1 delta (guanine nucleotide exchange protein) (Eef1d), mRNA.
scl20687.14.1_105-S	54.4148	1.76	Slc43a3	NM_021398.1	Mus musculus solute carrier family 43, member 3 (Slc43a3), mRNA.
scl014980.6_15-S	54.3829	1.74	H2-L		
scl0229473.1_11-S	54.2133	26.84	D930015E06Rik	NM_172681.2	Mus musculus RIKEN cDNA D930015E06 gene (D930015E06Rik), mRNA.
scl0394435.1_1-S	54.208	2.47	Ugt1a9	NM_201410	
scl066840.1_106-S	54.1883	10.31	0610008N23Rik	NM_025793.1	
scl013885.9_29-S	54.1641	1.48	Esd	NM_016903	
scl53212.12.1_83-S	54.1324	2.71	1810073H04Rik	XM_129159.3	
scl0246257.1_125-S	54.0995	2.09	Ovca2	NM_027136.2	Mus musculus candidate tumor suppressor OVCA2 (Ovca2), mRNA.
scl25565.5.123_2-S	54.0608	5.68	2700059D21Rik	XM_358352.1	
scl0002429.1_121-S	54.0387	2.61	D15Ertd785e	NM_025795.2	Mus musculus DNA segment, Chr 15, ERATO Doi 785, expressed (D15Ertd785e), mRNA.
scl0002052.1_0-S	53.9981	1.78	Cryz	NM_009968.1	Mus musculus crystallin, zeta (Cryz), mRNA.
scl19517.6_242-S	53.9811	1.48	Surf4	NM_011512.2	Mus musculus surfeit gene 4 (Surf4), mRNA.
scl38585.9.1_9-S	53.9603	2.36	2900091E11Rik	NM_026070.1	Mus musculus RIKEN cDNA 2900091E11 gene (2900091E11Rik), mRNA.
scl072692.1_23-S	53.9337	3.89	2810036L13Rik	NM_144802.2	Mus musculus RIKEN cDNA 2810036L13 gene (2810036L13Rik), mRNA.
scl0075423.2_59-S	53.873	2.62	2810410P22Rik	NM_182994.1	
scl0002097.1_75-S	53.8213	5.29	St7l	NM_153091.2	Mus musculus suppression of tumorigenicity 7-like (St7l), mRNA.
scl18838.17_213-S	53.7425	8.92	Mrg1	NM_010825	Mus musculus myeloid ecotropic viral integration site-related gene 1 (Mrg1), mRNA.
scl0014897.1_159-S	53.7147	1.8	Trip12	NM_133975.2	
scl000057.1_28-S	53.6815	3.86	Nme7	NM_138314.1	Mus musculus non-metastatic cells 7, protein expressed in (Nme7), mRNA.
scl028035.1_139-S	53.6371	1.51	Usp39	NM_138592.1	Mus musculus ubiquitin specific protease 39 (Usp39), mRNA.
scl011906.16_208-S	53.5375	1.7	Atbf1	NM_007496.1	Mus musculus AT motif binding factor 1 (Atbf1), mRNA.
scl012268.2_49-S	53.5067	2.11	C4	NM_009780	Mus musculus complement component 4 (within H-2S) (C4), mRNA.
scl066923.1_197-S	53.485	7.26	2610016F04Rik	NM_025847.1	Mus musculus RIKEN cDNA 2610016F04 gene (2610016F04Rik), mRNA.
scl22910.4.1_66-S	53.4744	4.82	S100a10	NM_009112.1	Mus musculus S100 calcium binding protein A10 (calpactin) (S100a10), mRNA.
scl056455.3_23-S	53.4679	13.71	Dnclc1	NM_019682	Mus musculus dynein, cytoplasmic, light chain 1 (Dnclc1), mRNA.
scl29671.63_470-S	53.4412	1.97	Itpr1	NM_010585.2	Mus musculus inositol 1,4,5-triphosphate receptor 1 (Itpr1), mRNA.
scl054217.2_13-S	53.4212	4.35	Rpl36	NM_018730	Mus musculus ribosomal protein L36 (Rpl36), mRNA.
scl0214489.1_247-S	53.4119	1.54	BC003965	XM_128528.2	Mus musculus cDNA sequence BC003965 (BC003965), mRNA.
scl23771.9.1_58-S	53.3428	1.37	Eif3s2	NM_018799.1	Mus musculus eukaryotic translation initiation factor 3, subunit 2 (beta) (Eif3s2), mRNA.
scl43301.1.2269_3-S	53.3354	12.86	Gdap10	NM_010268.1	Mus musculus ganglioside-induced differentiation-associated-protein 10 (Gdap10), mRNA.
scl066151.5_253-S	53.3261	1.67	1110020C13Rik	NM_025385.2	Mus musculus RIKEN cDNA 1110020C13 gene (1110020C13Rik), mRNA.
scl22543.4.4_13-S	53.2297	1.72	Adh5	NM_007410.2	Mus musculus alcohol dehydrogenase 5 (class III), chi polypeptide (Adh5), mRNA.
scl43363.13_112-S	53.1924	3.55	Txndc7	XM_126809.3	
scl000275.1_39-S	53.1737	1.58	3010033P07Rik	NM_029767.1	Mus musculus RIKEN cDNA 3010033P07 gene (3010033P07Rik), mRNA.
scl22689.16.1_37-S	53.1687	1.71	Sdfr2	NM_009146	Mus musculus stromal cell derived factor receptor 2 (Sdfr2), mRNA.
scl26467.3.1_2-S	53.1444	2.05	Chic2	NM_028850.1	Mus musculus cysteine-rich hydrophobic domain 2 (Chic2), mRNA.
scl30652.3_263-S	53.1367	3.76	2410015N17Rik	NM_023203.1	Mus musculus RIKEN cDNA 2410015N17 gene (2410015N17Rik), mRNA.

scl068713.2_9-S	53.1191	1.8	Ifitm1	NM_026820	Mus musculus interferon induced transmembrane protein 1 (Ifitm1), mRNA.
scl43223.12.1_25-S	53.0979	1.57	Ap4s1	NM_021710.1	Mus musculus adaptor-related protein complex AP-4, sigma 1 (Ap4s1), mRNA.
scl38291.1_10-S	53.0951	2.67	D10Ucla2	NM_133996.2	Mus musculus DNA segment, Chr 10, University of California at Los Angeles 2 (D10Ucla2), mRNA.
scl011722.3_4-S	53.0232	2.75	Amy1	NM_007446.1	Mus musculus amylase 1, salivary (Amy1), mRNA.
scl0002638.1_12-S	52.857	25.7	Sdhb	NM_023374.3	Mus musculus succinate dehydrogenase complex, subunit B, iron sulfur (Ip) (Sdhb), mRNA.
scl027364.2_20-S	52.7124	2.45	Srr	NM_013761.2	Mus musculus serine racemase (Srr), mRNA.
scl019243.1_42-S	52.7046	9.76	Ptp4a1	NM_011200	Mus musculus protein tyrosine phosphatase 4a1 (Ptp4a1), mRNA.
scl076279.3_16-S	52.6931	1.73	Cyp2d26	NM_029562.1	Mus musculus cytochrome P450, family 2, subfamily d, polypeptide 26 (Cyp2d26), mRNA.
scl0072117.2_260-S	52.6867	9.26	Mak3	XM_148131.1	
scl056876.17_315-S	52.6534	2.37	Nelf	NM_020276.2	Mus musculus nasal embryonic LHRH factor (Nelf), mRNA.
scl24599.7_209-S	52.633	2.24	Sdf4	NM_011341.3	Mus musculus stromal cell derived factor 4 (Sdf4), mRNA.
scl21684.2.5_0-S	52.5689	2.05	Rbm15	XM_131139.4	
scl0069740.2_2-S	52.5007	3.8	2410012M04Rik	NM_027193.2	Mus musculus RIKEN cDNA 2410012M04 gene (2410012M04Rik), mRNA.
scl0001353.1_10-S	52.4558	1.75	H2afv	XM_126043.3	
scl50582.4.1_9-S	52.3776	1.64	Clpp	NM_017393	Mus musculus caseinolytic protease, ATP-dependent, proteolytic subunit homolog (E. coli) (Clpp), mRNA.
scl49488.8_241-S	52.3285	1.89	Zfp263	NM_148924.2	Mus musculus zinc finger protein 263 (Zfp263), mRNA.
scl0014924.2_213-S	52.3259	13	Baiap1	NM_010367.1	Mus musculus BAI1-associated protein 1 (Baiap1), mRNA.
scl0054151.1_287-S	52.3179	1.59	Cyhr1	NM_019396.1	Mus musculus cysteine and histidine rich 1 (Cyhr1), mRNA.
scl18802.5.1_208-S	52.2326	1.85	Ndufaf1	NM_027175.2	Mus musculus NADH dehydrogenase (ubiquinone) 1 alpha subcomplex, assembly factor 1 (Ndufaf1), mRNA.
scl0018173.2_296-S	52.0924	1.92	Slc11a1	NM_013612.1	Mus musculus solute carrier family 11 (proton-coupled divalent metal ion transporters), member 1 (Slc11a1), mRNA.
scl0075991.1_191-S	52.0696	1.64	5033405K12Rik	NM_153567.1	Mus musculus RIKEN cDNA 5033405K12 gene (5033405K12Rik), mRNA.
scl078925.3_27-S	51.8978	3.88	Srd5a1	NM_175283.2	Mus musculus steroid 5 alpha-reductase 1 (Srd5a1), mRNA.
scl015018.6_2-S	51.8278	2.41	H2-Q7	XM_359282.1	Mus musculus histocompatibility 2, Q region locus 7 (H2-Q7), mRNA.
scl27567.9_262-S	51.8124	9.41	Ccng2	NM_007635.2	Mus musculus cyclin G2 (Ccng2), mRNA.
scl0076933.2_252-S	51.7347	4.69	2310061N23Rik	NM_029803.1	Mus musculus RIKEN cDNA 2310061N23 gene (2310061N23Rik), mRNA.
scl027045.1_0-S	51.6766	14.07	Nit1	NM_012049.1	Mus musculus nitrilase 1 (Nit1), mRNA.
scl019777.1_32-S	51.6602	1.92	C80913	NM_011274.2	Mus musculus expressed sequence C80913 (C80913), mRNA.
scl49651.7_46-S	51.6528	3.25	Tgif	NM_009372.2	Mus musculus TG interacting factor (Tgif), mRNA.
scl33330.12_227-S	51.5688	12.54	Tat	NM_146214.1	Mus musculus tyrosine aminotransferase (Tat), mRNA.
scl022121.1_118-S	51.5197	1.7	Rpl13a	NM_009438	Mus musculus ribosomal protein L13a (Rpl13a), mRNA.
scl011951.2_99-S	51.5057	1.66	Atp5g1	NM_007506.2	Mus musculus ATP synthase, H+ transporting, mitochondrial F0 complex, subunit c (subunit 9), isoform 1 (Atp5g1), mRNA.
scl26598.18_80-S	51.4711	1.83	Gpr125	XM_132089.2	
scl0003542.1_12-S	51.4677	2.11	Smfn	NM_024233.2	Mus musculus small fragment nuclease (Smfn), mRNA.
scl0077721.2_286-S	51.4204	1.44	Mrps5	NM_029963.2	Mus musculus mitochondrial ribosomal protein S5 (Mrps5), mRNA.
scl015953.2_220-S	51.4049	4	Ifi47	NM_008330.1	Mus musculus interferon gamma inducible protein (Ifi47), mRNA.
scl37831.9_401-S	51.3954	10.53	Tfam	NM_009360.2	Mus musculus transcription factor A, mitochondrial (Tfam), mRNA.
scl26190.16_67-S	51.347	1.75	Coro1c	NM_011779.2	Mus musculus coronin, actin binding protein 1C (Coro1c), mRNA.
scl0067238.2_233-S	51.2428	3.71	2810453I06Rik	NM_026050.1	Mus musculus RIKEN cDNA 2810453I06 gene (2810453I06Rik), mRNA.
scl47523.9_497-S	51.2088	1.36	Gpd1	NM_010271.2	Mus musculus glycerol-3-phosphate dehydrogenase 1 (soluble) (Gpd1), mRNA.
scl25240.12_194-S	51.1791	1.36	Pgm2	NM_028132	Mus musculus phosphoglucomutase 2 (Pgm2), mRNA.
scl52206.9_527-S	51.1604	4.38	Rnf138	NM_019706.2	Mus musculus ring finger protein 138 (Rnf138), transcript variant 2, mRNA.
scl066845.4_127-S	51.0131	1.4	Mrpl33	NM_025796.1	Mus musculus mitochondrial ribosomal protein L33 (Mrpl33), mRNA.
scl013118.12_302-S	50.6621	47.31	Cyp4a12	NM_172306.1	Mus musculus cytochrome P450, family 4, subfamily a, polypeptide 12 (Cyp4a12), mRNA.
scl000657.1_20-S	50.6002	2.09	Mrps31	NM_020560.1	Mus musculus mitochondrial ribosomal protein S31 (Mrps31), mRNA.
scl00243771.2_198-S	50.5868	2.3	Zc3hdc1	NM_172893.1	Mus musculus zinc finger CCCH type domain containing 1 (Zc3hdc1), mRNA.
scl076281.4_19-S	50.514	1.72	Tax1bp3	NM_029564	
scl0020923.1_202-S	50.4462	1.43	Supt4h2	NM_011509	Mus musculus suppressor of Ty 4 homolog 2 (S. cerevisiae) (Supt4h2), mRNA.
scl44563.12_95-S	50.3945	9.78	2810446P07Rik	NM_175187.3	Mus musculus RIKEN cDNA 2810446P07 gene (2810446P07Rik), mRNA.
scl0004044.1_9-S	50.3291	9.66	Ppp1cb	NM_172707.1	Mus musculus protein phosphatase 1, catalytic subunit, beta isoform (Ppp1cb), mRNA.
scl066154.6_169-S	50.3264	1.39	Tmem14c	NM_025387.1	
scl0014105.1_218-S	50.2129	2.54	Fusip1	NM_010178.1	Mus musculus FUS interacting protein (serine-arginine rich) 1 (Fusip1), mRNA.
scl0002273.1_3-S	50.1294	8.95	Actr10	NM_019785.1	Mus musculus ARP10 actin-related protein 10 homolog (S. cerevisiae) (Actr10), mRNA.
scl064656.5_1-S	50.079	1.99	Mrps23	NM_024174.4	Mus musculus mitochondrial ribosomal protein S23 (Mrps23), mRNA.
scl0016419.2_269-S	50.0207	2.97	Itgb5	NM_010580.1	Mus musculus integrin beta 5 (Itgb5), mRNA.
scl52593.10.1_23-S	50.0152	3.98	5033414D02Rik	NM_026362.1	Mus musculus RIKEN cDNA 5033414D02 gene (5033414D02Rik), mRNA.
scl0002033.1_5-S	49.8708	1.71	1110013G13Rik	NM_146141.1	Mus musculus RIKEN cDNA 1110013G13 gene (1110013G13Rik), mRNA.

scl068082.4_12-S	49.8623	2.66	Dusp19	NM_024438.2	Mus musculus dual specificity phosphatase 19 (Dusp19), mRNA.
scl0001961.1_16-S	49.7683	1.75	Acadm	NM_007382	Mus musculus acetyl-Coenzyme A dehydrogenase, medium chain (Acadm), mRNA.
scl00223664.2_280-S	49.7136	8.58	E130306I01Rik	NM_145471.1	Mus musculus RIKEN cDNA E130306I01 gene (E130306I01Rik), mRNA.
scl39911.9_64-S	49.6921	2.42	Gosr1	NM_016810.2	Mus musculus golgi SNAP receptor complex member 1 (Gosr1), mRNA.
scl021885.1_30-S	49.6449	1.57	Tle1	NM_011599.2	Mus musculus transducin-like enhancer of split 1, homolog of Drosophila E(spl) (Tle1), mRNA.
scl0109135.16_234-S	49.64	14.11	Plekha5	NM_144920.2	
scl022186.3_30-S	49.4544	1.93	Uba52	NM_019883	Mus musculus ubiquitin A-52 residue ribosomal protein fusion product 1 (Uba52), mRNA.
scl0000117.1_15-S	49.4446	4	Taf6	NM_009315.1	Mus musculus adaptor-related protein complex AP-4, mu 1 (Ap4m1), mRNA.
scl33434.12_331-S	49.3991	48.72	9130231C15Rik	NM_133960.2	Mus musculus RIKEN cDNA 9130231C15 gene (9130231C15Rik), mRNA.
scl24615.5.1_59-S	49.295	1.44	1500011L16Rik	NM_026899.1	Mus musculus RIKEN cDNA 1500011L16 gene (1500011L16Rik), mRNA.
scl0073834.1_147-S	49.2474	5.95	Atp6v1d	NM_023721.1	Mus musculus ATPase, H+ transporting, V1 subunit D (Atp6v1d), mRNA.
scl38446.16_40-S	49.1557	11.72	Nap1l1	NM_015781.2	Mus musculus nucleosome assembly protein 1-like 1 (Nap1l1), mRNA.
scl020916.11_281-S	49.0959	13.06	Sucla2	XM_127733.2	Mus musculus succinate-Coenzyme A ligase, ADP-forming, beta subunit (Sucla2), mRNA.
scl21857.5.1_13-S	49.0773	1.35	Psmb4	NM_008945.1	Mus musculus proteasome (prosome, macropain) subunit, beta type 4 (Psmb4), mRNA.
scl059038.1_0-S	49.0663	2.15	Pxmp4	NM_021534.2	Mus musculus peroxisomal membrane protein 4 (Pxmp4), mRNA.
scl000050.1_4-S	49.0642	13.86	Gpr124	NM_054044.1	Mus musculus G protein-coupled receptor 124 (Gpr124), mRNA.
scl074104.1_75-S	49.0562	2.03	Abcb6	NM_023732.2	Mus musculus ATP-binding cassette, sub-family B (MDR/TAP), member 6 (Abcb6), mRNA.
scl0078757.1_96-S	49.0505	1.87	4921505C17Rik	NM_030168.2	Mus musculus RIKEN cDNA 4921505C17 gene (4921505C17Rik), mRNA.
scl44140.2_304-S	48.9583	10.87	Uqcrfs1	NM_025710.1	Mus musculus ubiquinol-cytochrome c reductase, Rieske iron-sulfur polypeptide 1 (Uqcrfs1), mRNA.
scl0068198.2_271-S	48.9192	43.14	Ndufb2	NM_026612.1	Mus musculus NADH dehydrogenase (ubiquinone) 1 beta subcomplex, 2 (Ndufb2), mRNA.
scl070478.15_14-S	48.9142	1.9	Mipep	NM_027436.1	Mus musculus mitochondrial intermediate peptidase (Mipep), mRNA.
scl056278.9_3-S	48.8966	1.67	Gkap1	NM_019832.2	
scl18407.1_27-S	48.8811	11.5	Mafb	NM_010658.2	Mus musculus v-maf musculoaponeurotic fibrosarcoma oncogene family, protein B (avian) (Mafb), mRNA.
scl0020461.1_17-S	48.8548	2.88	Silg111		
scl51657.9.1_30-S	48.8499	1.51	Abhd3	NM_134130.1	Mus musculus abhydrolase domain containing 3 (Abhd3), mRNA.
scl068077.3_0-S	48.8055	4.96	Gltscr2	NM_133831.1	Mus musculus glioma tumor suppressor candidate region gene 2 (Gltscr2), mRNA.
scl0013669.1_3-S	48.8048	1.66	Eif3s10	NM_010123.2	Mus musculus eukaryotic translation initiation factor 3, subunit 10 (theta) (Eif3s10), mRNA.
scl019982.5_48-S	48.7756	1.46	Rpl36a	NM_019865.2	Mus musculus ribosomal protein L36a (Rpl36a), mRNA.
scl19749.5.1_9-S	48.7458	2.93	Meig1	NM_008579.4	Mus musculus meiosis expressed gene 1 (Meig1), mRNA.
scl49542.15_45-S	48.6257	2.62	D030028O16Rik	NM_145984.2	Mus musculus RIKEN cDNA D030028O16 gene (D030028O16Rik), mRNA.
scl014790.2_94-S	48.6066	1.96	Grcc10	NM_013535.1	Mus musculus gene rich cluster, C10 gene (Grcc10), mRNA.
scl019166.8_87-S	48.6023	1.35	Psma2	NM_008944.1	Mus musculus proteasome (prosome, macropain) subunit, alpha type 2 (Psma2), mRNA.
scl0229877.2_148-S	48.6009	1.6	Rap1gds1	NM_145544	Mus musculus RAP1, GTP-GDP dissociation stimulator 1 (Rap1gds1), mRNA.
scl40873.2_406-S	48.596	5.09	Arfl4	NM_025404.1	
scl058810.1_30-S	48.5116	2.65	Akr1a4	NM_021473.2	Mus musculus aldo-keto reductase family 1, member A4 (aldehyde reductase) (Akr1a4), mRNA.
scl056444.13_2-S	48.4248	1.56	Actr10	NM_019785.1	Mus musculus ARP10 actin-related protein 10 homolog (S. cerevisiae) (Actr10), mRNA.
scl054151.1_72-S	48.4015	9.92	Cyhr1	NM_019396	Mus musculus cysteine and histidine rich 1 (Cyhr1), mRNA.
scl54488.12.1_33-S	48.3357	3.14	Pir	XM_136134.3	
scl0116905.1_30-S	48.331	2.3	Dph2l1	NM_144491	Mus musculus diptheria toxin resistance protein required for diphthamide biosynthesis (Saccharomyces)-like 1 (Dph2l1), mRNA.
scl49316.10.1_12-S	48.2636	1.52	Dnajb11	XM_148071.1	Mus musculus DnaJ (Hsp40) homolog, subfamily B, member 11 (Dnajb11), mRNA.
scl0002469.1_386-S	48.2367	1.38	AI313915	NM_144845.1	Mus musculus expressed sequence AI313915 (AI313915), mRNA.
scl46602.21_8-S	48.2183	6.93	Nid2	NM_008695.1	Mus musculus nidogen 2 (Nid2), mRNA.
scl0024135.1_295-S	48.2163	2.47	Zfp68	NM_013844.1	Mus musculus zinc finger protein 68 (Zfp68), mRNA.
scl0012508.2_215-S	48.2006	13.96	Cd53	NM_007651.2	Mus musculus CD53 antigen (Cd53), mRNA.
scl0016597.1_0-S	48.1943	7.9	Klf12	NM_010636.2	Mus musculus Kruppel-like factor 12 (Klf12), mRNA.
scl29983.21.1_98-S	48.1823	5.51	Abcg2	NM_011920.1	Mus musculus ATP-binding cassette, sub-family G (WHITE), member 2 (Abcg2), mRNA.
scl52119.18_599-S	48.1154	5.42	Jmjd1b	NM_029518.1	Mus musculus jumonji domain containing 1B (Jmjd1b), mRNA.
scl0016396.1_119-S	48.0379	1.82	Itch	XM_192925.3	Mus musculus itchy (Itch), mRNA.
scl42032.3_211-S	48.0367	8.74	Bag5	XM_127149.3	Mus musculus BCL2-associated athanogene 5 (Bag5), mRNA.
scl42964.22.1_29-S	47.8992	3.6	Ylpm1	NM_178363.2	
scl24707.8.1_1-S	47.899	3.21	Mad2l2	NM_027985.1	Mus musculus MAD2 mitotic arrest deficient-like 2 (yeast) (Mad2l2), mRNA.
scl0140792.13_120-S	47.859	19.64	Colec12	NM_130449.1	Mus musculus collectin sub-family member 12 (Colec12), mRNA.
scl32097.7.1_17-S	47.7906	7.54	2010110P09Rik	XM_355937.1	
scl011787.1_19-S	47.7426	9.37	Apbb2	NM_009686.1	Mus musculus amyloid beta (A4) precursor protein-binding, family B, member 2 (Apbb2), mRNA.
scl47764.3_554-S	47.7422	3.39	Cdc42ep1	NM_027219.1	Mus musculus CDC42 effector protein (Rho GTPase binding) 1 (Cdc42ep1), mRNA.
scl00107141.1_179-S	47.6854	2.78	Cyp2c50	NM_134144.1	Mus musculus cytochrome P450, family 2, subfamily c, polypeptide 50 (Cyp2c50), mRNA.

scl52860.17_315-S	47.6497	1.55	Syvn1	NM_028769.4	
scl0000115.1_6-S	47.516	3.73	Fip1l1	NM_024183.3	Mus musculus FIP1 like 1 (S. cerevisiae) (Fip1l1), mRNA.
scl0066704.2_280-S	47.4768	1.98	4921506I22Rik	NM_025717.2	Mus musculus RIKEN cDNA 4921506I22 gene (4921506I22Rik), mRNA.
scl0108755.3_138-S	47.4692	12.7	2610208E05Rik	NM_175364.2	Mus musculus RIKEN cDNA 2610208E05 gene (2610208E05Rik), mRNA.
scl0015926.2_189-S	47.4655	1.45	Idh1	NM_010497.1	Mus musculus isocitrate dehydrogenase 1 (NADP+), soluble (Idh1), mRNA.
scl46304.5.1_86-S	47.2005	4.19	Mrpl52	NM_026851.1	Mus musculus mitochondrial ribosomal protein L52 (Mrpl52), mRNA.
scl0382053.6_30-S	47.1985	3.12	Es31	XM_356125.1	
scl000056.1_228-S	47.0959	7.99	C76566	NM_178879.2	
scl066292.2_43-S	47.0423	1.59	Mrps21	NM_078479.2	Mus musculus mitochondrial ribosomal protein S21 (Mrps21), mRNA.
scl013101.1_72-S	46.9911	1.89	Cyp2d10	NM_010005.2	Mus musculus cytochrome P450, family 2, subfamily d, polypeptide 10 (Cyp2d10), mRNA.
scl51160.13.1_27-S	46.9183	1.79	Tfb1m	NM_146074.1	Mus musculus transcription factor B1, mitochondrial (Tfb1m), mRNA.
scl33480.16_131-S	46.8458	10.9	4930470D19Rik	NM_026274.2	Mus musculus RIKEN cDNA 4930470D19 gene (4930470D19Rik), mRNA.
scl021858.1_100-S	46.7462	1.84	Timp2	NM_011594.2	Mus musculus tissue inhibitor of metalloproteinase 2 (Timp2), mRNA.
scl42329.4_2-S	46.7223	2.09	Zfp50	XM_127016.2	Mus musculus zinc finger protein 50 (Zfp50), mRNA.
scl0001033.1_52-S	46.6926	5.23	Slco1b2	NM_020495.1	Mus musculus solute carrier organic anion transporter family, member 1b2 (Slco1b2), transcript variant 1, mRNA.
scl46821.2.2_6-S	46.5607	1.56	2700088M22Rik	NM_026025	Mus musculus RIKEN cDNA 2700088M22 gene (2700088M22Rik), mRNA.
scl25806.10.1_16-S	46.5313	1.72	Zdhhc4	NM_028379.1	Mus musculus zinc finger, DHHC domain containing 4 (Zdhhc4), mRNA.
scl00319800.1_48-S	46.5249	3.75	C730048C13Rik	NM_177002.2	Mus musculus RIKEN cDNA C730048C13 gene (C730048C13Rik), mRNA.
scl0004155.1_21-S	46.4471	9.58	Hip2	NM_016786.2	Mus musculus huntingtin interacting protein 2 (Hip2), mRNA.
scl0110829.10_232-S	46.4234	10.69	Lims1	NM_026148.1	Mus musculus LIM and senescent cell antigen-like domains 1 (Lims1), mRNA.
scl34081.10.510_67-S	46.4158	1.37	0710008K08Rik	NM_026995.1	
scl42591.3.1_5-S	46.2871	3.05	Idb2	NM_010496.2	Mus musculus inhibitor of DNA binding 2 (Idb2), mRNA.
scl35530.18_2-S	46.2446	2.77	Mod1	NM_008615	Mus musculus malic enzyme, supernatant (Mod1), mRNA.
scl070380.1_33-S	46.2295	6.06	Mospd1	NM_027409	Mus musculus motile sperm domain containing 1 (Mospd1), mRNA.
scl0023821.2_49-S	46.2001	2.01	Bace1	NM_011792.3	Mus musculus beta-site APP cleaving enzyme 1 (Bace1), mRNA.
scl0213673.3_37-S	46.1844	1.39	9530068E07Rik	XM_203329.2	Mus musculus RIKEN cDNA 9530068E07 gene (9530068E07Rik), mRNA.
scl00227399.2_17-S	46.1279	9.62	AW555814	NM_173760.3	Mus musculus expressed sequence AW555814 (AW555814), mRNA.
scl012274.15_0-S	45.9552	3.33	C6	NM_016704.1	Mus musculus complement component 6 (C6), mRNA.
scl38536.23.1_129-S	45.9173	3.12	Fgd6	NM_053072	Mus musculus FYVE, RhoGEF and PH domain containing 6 (Fgd6), mRNA.
scl49981.15.1_56-S	45.9035	1.55	Gtf2h4	NM_010364.1	Mus musculus general transcription factor II H, polypeptide 4 (Gtf2h4), mRNA.
scl0170719.14_114-S	45.9008	1.83	Oxr1	NM_130885.1	Mus musculus oxidation resistance 1 (Oxr1), mRNA.
scl38453.7.1_27-S	45.8963	1.66	Csrp2	NM_007792.2	Mus musculus cysteine and glycine-rich protein 2 (Csrp2), mRNA.
scl0071911.2_148-S	45.8757	1.94	Bdh	NM_175177.3	Mus musculus 3-hydroxybutyrate dehydrogenase (heart, mitochondrial) (Bdh), mRNA.
scl00116891.1_125-S	45.8675	10.57	BC005682	NM_033562.2	Mus musculus cDNA sequence BC005682 (BC005682), mRNA.
scl029869.1_26-S	45.8459	1.91	Ulk2	NM_013881.3	Mus musculus Unc-51 like kinase 2 (C. elegans) (Ulk2), mRNA.
scl37787.43_431-S	45.8112	1.42	Col18a1	NM_009929.2	Mus musculus procollagen, type XVIII, alpha 1 (Col18a1), mRNA.
scl45906.4.1_41-S	45.7936	2.65	Fhit	NM_010210.1	Mus musculus fragile histidine triad gene (Fhit), mRNA.
scl068050.2_0-S	45.788	1.76	6330407G11Rik	XM_124343.1	Mus musculus RIKEN cDNA 6330407G11 gene (6330407G11Rik), mRNA.
scl0021873.2_51-S	45.7845	2.12	Tjp2	NM_011597.1	Mus musculus tight junction protein 2 (Tjp2), mRNA.
scl076178.1_20-S	45.7644	8.49	6330578E17Rik	NM_198006.1	Mus musculus RIKEN cDNA 6330578E17 gene (6330578E17Rik), mRNA.
scl0067891.1_266-S	45.689	10.19	Rpl4	NM_024212.2	Mus musculus ribosomal protein L4 (Rpl4), mRNA.
scl015032.1_236-S	45.6807	1.69	H2-T17	NM_010396	Mus musculus histocompatibility 2, T region locus 17 (H2-T17), mRNA.
scl0002138.1_36-S	45.6678	11.33	4833424P18Rik	NM_029017	Mus musculus RIKEN cDNA 4833424P18 gene (4833424P18Rik), mRNA.
scl52829.1_29-S	45.6429	1.57	B3gnt6	NM_175383.1	
scl011984.1_128-S	45.6308	10.39	Atp6v0c	NM_009729.1	Mus musculus ATPase, H+ transporting, V0 subunit C (Atp6v0c), mRNA.
scl022038.3_2-S	45.6214	2.58	Plscr1	NM_011636.1	Mus musculus phospholipid scramblase 1 (Plscr1), mRNA.
scl45165.9.4_29-S	45.5466	2.49	Dct	NM_010024	Mus musculus dopachrome tautomerase (Dct), mRNA.
scl0003463.1_14-S	45.5039	10.92	Senp6	NM_146003	Mus musculus SUMO/sentrin specific protease 6 (Senp6), mRNA.
scl012389.5_102-S	45.482	4.53	Cav1	NM_007616.2	
scl020955.1_87-S	45.3407	10.27	Sybl1	NM_011515.1	Mus musculus synaptobrevin like 1 (Sybl1), mRNA.
scl33924.6.9_11-S	45.3354	1.45	Ppp2cb	NM_017374.2	Mus musculus protein phosphatase 2a, catalytic subunit, beta isoform (Ppp2cb), mRNA.
scl46463.1.8_87-S	45.2719	3.12	Gdf2	NM_019506	Mus musculus growth differentiation factor 2 (Gdf2), mRNA.
scl49743.39.1_15-S	45.2475	1.83	C3	NM_009778.1	Mus musculus complement component 3 (C3), mRNA.
scl067755.12_300-S	45.2163	1.39	Ddx47	NM_026360.1	Mus musculus DEAD (Asp-Glu-Ala-Asp) box polypeptide 47 (Ddx47), mRNA.
scl0209645.9_0-S	45.1909	8.8	E130319B15Rik	NM_178663.2	Mus musculus RIKEN cDNA E130319B15 gene (E130319B15Rik), mRNA.
scl0012558.1_44-S	45.1212	1.82	Cdh2	NM_007664.1	Mus musculus cadherin 2 (Cdh2), mRNA.

scl066488.1_275-S	45.077	11.12	2010309E21Rik	XM_357858.1	Mus musculus RIKEN cDNA 2010309E21 gene (2010309E21Rik), mRNA.
scl067865.1_323-S	45.0732	4.17	Rgs10	NM_026418.1	Mus musculus regulator of G-protein signalling 10 (Rgs10), mRNA.
scl20301.11_1-S	44.9868	2.66	Slc20a1	NM_015747.1	Mus musculus solute carrier family 20, member 1 (Slc20a1), mRNA.
scl072429.2_21-S	44.9723	2.34	2010203O07Rik	XM_131409.3	Mus musculus RIKEN cDNA 2010203O07 gene (2010203O07Rik), mRNA.
scl0068073.2_190-S	44.8263	1.51	A930016P21Rik	NM_026546.1	Mus musculus RIKEN cDNA A930016P21 gene (A930016P21Rik), mRNA.
scl0056324.2_184-S	44.7078	8.87	Stam2	NM_019667.2	Mus musculus signal transducing adaptor molecule (SH3 domain and ITAM motif) 2 (Stam2), mRNA.
scl23237.22_584-S	44.6964	3.11	3110057O12Rik	NM_026622.1	Mus musculus RIKEN cDNA 3110057O12 gene (3110057O12Rik), mRNA.
scl0014870.1_15-S	44.6381	2.92	Gstp1	NM_013541.1	Mus musculus glutathione S-transferase, pi 1 (Gstp1), mRNA.
scl41582.8.1_74-S	44.6255	1.54	Sara2	NM_025535.1	Mus musculus SAR1a gene homolog 2 (S. cerevisiae) (Sara2), mRNA.
scl0330260.1_61-S	44.6058	1.4	Pon2	NM_183308.1	Mus musculus paraoxonase 2 (Pon2), mRNA.
scl0002901.1_9-S	44.5919	7.74	Psmd10	NM_016883.3	Mus musculus proteasome (prosome, macropain) 26S subunit, non-ATPase, 10 (Psmd10), mRNA.
scl0071755.2_316-S	44.5657	1.49	1300018L09Rik	NM_027903.1	
scl0020567.1_162-S	44.5394	1.83	Slp	NM_011413	Mus musculus sex-limited protein (Slp), mRNA.
scl000928.1_86-S	44.5299	2.35	Hspd1	NM_010477.2	Mus musculus heat shock protein 1 (chaperonin) (Hspd1), mRNA.
scl39960.2_268-S	44.51	1.35	0610009E20Rik	NM_025318.1	Mus musculus RIKEN cDNA 0610009E20 gene (0610009E20Rik), mRNA.
scl0014972.1_210-S	44.4694	2.66	H2-K1	NM_001001892	
scl0068033.1_52-S	44.4629	1.59	2810437L13Rik	NM_197980.1	Mus musculus RIKEN cDNA 2810437L13 gene (2810437L13Rik), mRNA.
scl011988.11_16-S	44.4513	4.08	Slc7a2	NM_007514	Mus musculus solute carrier family 7 (cationic amino acid transporter, y+ system), member 2 (Slc7a2), mRNA.
scl52508.10.1_140-S	44.4225	1.79	Cyp2c70	NM_145499.1	Mus musculus cytochrome P450, family 2, subfamily c, polypeptide 70 (Cyp2c70), mRNA.
scl46091.5.1_30-S	44.4172	3.02	Cpb2	NM_019775.2	Mus musculus carboxypeptidase B2 (plasma) (Cpb2), mRNA.
scl0013000.1_146-S	44.4161	2.33	Csnk2a2	NM_009974.2	Mus musculus casein kinase II, alpha 2, polypeptide (Csnk2a2), mRNA.
scl0223255.1_245-S	44.3547	2.06	Stk24	NM_145465.1	Mus musculus serine/threonine kinase 24 (STE20 homolog, yeast) (Stk24), mRNA.
scl38974.6_243-S	44.3514	1.64	Ostm1	NM_172416.2	Mus musculus osteopetrosis associated transmembrane protein 1 (Ostm1), mRNA.
scl017846.2_20-S	44.3406	1.4	Commd1	NM_144514.1	Mus musculus COMM domain containing 1 (Commd1), mRNA.
scl27972.10.1_31-S	44.2848	1.87	Khk	NM_008439.2	Mus musculus ketohexokinase (Khk), mRNA.
scl0067398.1_126-S	44.2727	1.99	Srpr	NM_026130.1	Mus musculus signal recognition particle receptor (docking protein) (Srpr), mRNA.
scl0014263.1_71-S	44.2467	2.34	Fmo5	NM_010232.3	Mus musculus flavin containing monooxygenase 5 (Fmo5), mRNA.
scl0003301.1_436-S	44.2425	10.07	Nfs1	NM_010911.1	Mus musculus nitrogen fixation gene 1 (S. cerevisiae) (Nfs1), mRNA.
scl28136.6_235-S	44.2077	1.82	Cldn12	NM_022890.1	Mus musculus claudin 12 (Cldn12), mRNA.
scl067116.1_6-S	44.1958	1.89	Cuedc2	NM_024192.1	Mus musculus CUE domain containing 2 (Cuedc2), mRNA.
scl24327.50_27-S	44.1865	14.77	Abca1	NM_013454.2	Mus musculus ATP-binding cassette, sub-family A (ABC1), member 1 (Abca1), mRNA.
scl013244.1_23-S	44.1561	1.55	Degs	NM_007853.2	Mus musculus degenerative spermatocyte homolog (Drosophila) (Degs), mRNA.
scl0001789.1_298-S	44.1007	4.17	Il1rap	NM_134103.1	Mus musculus interleukin 1 receptor accessory protein (Il1rap), mRNA.
scl21410.6.2_5-S	44.0221	2.25	2310066N05Rik	NM_027371.1	Mus musculus RIKEN cDNA 2310066N05 gene (2310066N05Rik), mRNA.
scl50575.22.1_51-S	43.9422	3.94	Emr1	NM_010130.1	Mus musculus EGF-like module containing, mucin-like, hormone receptor-like sequence 1 (Emr1), mRNA.
scl066169.3_11-S	43.9047	1.74	Tomm7	NM_025394	Mus musculus translocase of outer mitochondrial membrane 7 homolog (yeast) (Tomm7), mRNA.
scl20945.14_483-S	43.8573	1.82	Epc2	NM_172663.2	
scl000694.1_23-S	43.8256	3.2	F11	NM_028066.1	Mus musculus coagulation factor XI (F11), mRNA.
scl0056330.1_0-S	43.7273	1.77	Pdcd5	NM_019746.2	Mus musculus programmed cell death 5 (Pdcd5), mRNA.
scl46056.12.1_60-S	43.7231	11.1	Mtrf1	NM_145960	Mus musculus mitochondrial translational release factor 1 (Mtrf1), mRNA.
scl015519.1_32-S	43.7216	15.99	Hspca	NM_010480	Mus musculus heat shock protein 1, alpha (Hspca), mRNA.
scl53440.6.1_16-S	43.6909	5.4	BC021614	NM_144869.1	Mus musculus cDNA sequence BC021614 (BC021614), mRNA.
scl39384.39.1_1-S	43.6539	2.76	Abca8a	NM_153145.1	Mus musculus ATP-binding cassette, sub-family A (ABC1), member 8a (Abca8a), mRNA.
scl54743.5_163-S	43.6517	1.93	Gjb1	NM_008124.2	Mus musculus gap junction membrane channel protein beta 1 (Gjb1), mRNA.
scl24467.10_119-S	43.6422	1.81	Slc35a1	NM_011895.2	Mus musculus solute carrier family 35 (CMP-sialic acid transporter), member 1 (Slc35a1), mRNA.
scl072686.1_177-S	43.6403	1.42	Usp24	XM_131566.5	
scl000110.1_1-S	43.6007	3.34	Pex11a	NM_011068.1	Mus musculus peroxisomal biogenesis factor 11a (Pex11a), mRNA.
scl0394433.1_11-S	43.5986	2.19	Ugt1a2	NM_013701.1	
scl0378462.4_10-S	43.5587	10.58	Mopt	NM_194269.1	Mus musculus protein containing single MORN motif in testis (Mopt), mRNA.
scl24025.4.59_30-S	43.5141	10.83	Dio1	NM_007860	Mus musculus deiodinase, iodothyronine, type I (Dio1), mRNA.
scl0232087.7_6-S	43.4649	4	Mat2a	NM_145569	Mus musculus methionine adenosyltransferase II, alpha (Mat2a), mRNA.
scl34002.14.1_30-S	43.3883	1.66	2210415M20Rik	NM_027338.1	Mus musculus RIKEN cDNA 2210415M20 gene (2210415M20Rik), mRNA.
scl33549.18.1_0-S	43.3418	4.63	1300002A08Rik	NM_025827.2	Mus musculus RIKEN cDNA 1300002A08 gene (1300002A08Rik), mRNA.
scl00319800.2_57-S	43.3171	6.83	C730048C13Rik	NM_177002.2	Mus musculus RIKEN cDNA C730048C13 gene (C730048C13Rik), mRNA.
scl43828.6_15-S	43.2945	10.53	Zfp367	NM_175494.2	Mus musculus zinc finger protein 367 (Zfp367), mRNA.
scl39775.19.1_132-S	43.2814	4.19	Dhx40	NM_026191.1	Mus musculus DEAH (Asp-Glu-Ala-His) box polypeptide 40 (Dhx40), mRNA.

scl38867.11_23-S	43.2642	1.41	Sara1	NM_009120.1	Mus musculus SAR1a gene homolog 1 (S. cerevisiae) (Sara1), mRNA.
scl0068202.1_15-S	43.2328	1.5	Ndufa5	NM_026614.1	Mus musculus NADH dehydrogenase (ubiquinone) 1 alpha subcomplex, 5 (Ndufa5), mRNA.
scl017842.1_177-S	43.1846	5.19	Mup3	NM_010845	Mus musculus major urinary protein 3 (Mup3), mRNA.
scl43644.9.1_5-S	43.1715	9.98	Hexb	NM_010422.1	Mus musculus hexosaminidase B (Hexb), mRNA.
scl0071801.2_66-S	43.1603	12.13	Plekhf2	NM_175175.3	Mus musculus pleckstrin homology domain containing, family F (with FYVE domain) member 2 (Plekhf2), mRNA.
scl0017840.1_66-S	43.142	2.1	Mup1	NM_031188	Mus musculus major urinary protein 1 (Mup1), mRNA.
scl31224.16_382-S	43.1284	1.68	Lrrc28	NM_175124.3	
scl30402.11.1_13-S	43.121	1.92	BC020002	NM_145374.1	Mus musculus cDNA sequence BC020002 (BC020002), mRNA.
scl00243085.1_25-S	42.9805	2.45	C730031G17	NM_172881.1	Mus musculus hypothetical protein C730031G17 (C730031G17), mRNA.
scl055943.5_30-S	42.925	1.66	Stx8	NM_018768.1	Mus musculus syntaxin 8 (Stx8), mRNA.
scl50103.16_203-S	42.8883	1.49	Mtch1	NM_019880.2	Mus musculus mitochondrial carrier homolog 1 (C. elegans) (Mtch1), mRNA.
scl079560.1_5-S	42.8661	2.77	BC002236	NM_024475.2	Mus musculus cDNA sequence BC002236 (BC002236), mRNA.
scl44186.8.1_13-S	42.8492	11.53	Gmnn	NM_020567.1	Mus musculus geminin (Gmnn), mRNA.
scl15808.4.1_196-S	42.8339	1.46	C130074G19Rik	NM_178692.2	Mus musculus RIKEN cDNA C130074G19 gene (C130074G19Rik), mRNA.
scl39390.10.1_10-S	42.7787	2	BC029169	NM_153782.1	Mus musculus cDNA sequence BC029169 (BC029169), mRNA.
scl056088.2_28-S	42.7052	1.61	Dscr2	NM_019537.1	Mus musculus Down syndrome critical region homolog 2 (human) (Dscr2), mRNA.
scl37352.6_41-S	42.6526	1.54	Cdk2	NM_016756.1	Mus musculus cyclin-dependent kinase 2 (Cdk2), mRNA.
scl0066706.2_314-S	42.6272	1.39	4733401H18Rik	NM_023247.1	Mus musculus RIKEN cDNA 4733401H18 gene (4733401H18Rik), mRNA.
scl0056388.2_271-S	42.6043	8.55	Cyp3a25	NM_019792.1	Mus musculus cytochrome P450, family 3, subfamily a, polypeptide 25 (Cyp3a25), mRNA.
scl18596.16.1_32-S	42.5948	3.23	4930485D02Rik	NM_175225.2	Mus musculus RIKEN cDNA 4930485D02 gene (4930485D02Rik), mRNA.
scl23497.11_62-S	42.5695	1.37	Pex14	NM_019781.1	Mus musculus peroxisomal biogenesis factor 14 (Pex14), mRNA.
scl44051.9.1_139-S	42.5498	1.68	Tbc1d7	NM_025935.1	Mus musculus TBC1 domain family, member 7 (Tbc1d7), mRNA.
scl022644.5_265-S	42.3915	1.43	Rnf103	NM_009543.1	Mus musculus ring finger protein 103 (Rnf103), mRNA.
scl072795.9_17-S	42.3877	1.66	Ttc19	NM_028360.1	
scl020338.1_17-S	42.3559	1.49	Sel1h	NM_011344.1	Mus musculus Sel1 (suppressor of lin-12) 1 homolog (C. elegans) (Sel1h), mRNA.
scl0015039.1_320-S	42.2565	34.17	H2-T22	NM_010397	Mus musculus histocompatibility 2, T region locus 22 (H2-T22), mRNA.
scl48486.9.1_29-S	42.1676	1.48	Nr1i2	NM_010936.1	Mus musculus nuclear receptor subfamily 1, group I, member 2 (Nr1i2), mRNA.
scl069875.2_16-S	42.1648	1.41	Ndufa11	XM_128696.1	
scl0003351.1_17-S	42.1433	1.93	Mkks	NM_021527.1	Mus musculus McKusick-Kaufman syndrome protein (Mkks), mRNA.
scl0109552.5_10-S	42.096	1.53	Sri	NM_025618.1	Mus musculus sorcin (Sri), mRNA.
scl00218103.1_155-S	42.0923	1.36	Slc17a2	NM_144836.1	
scl33416.4.1_0-S	42.0811	2.39	Hspc171	NM_025486.1	
scl0001652.1_18-S	41.9759	1.57	E130307M08Rik	NM_026530.2	Mus musculus RIKEN cDNA E130307M08 gene (E130307M08Rik), mRNA.
scl026893.9_4-S	41.9687	1.46	Cops6	NM_012002.1	Mus musculus COP9 (constitutive photomorphogenic) homolog, subunit 6 (Arabidopsis thaliana) (Cops6), mRNA.
scl00117198.2_233-S	41.9347	4.93	Ivns1abp	NM_028582.2	Mus musculus influenza virus NS1A binding protein (Ivns1abp), mRNA.
scl000502.1_59-S	41.9228	2.51	Prdx5	NM_012021.1	Mus musculus peroxiredoxin 5 (Prdx5), mRNA.
scl000477.1_15-S	41.7222	1.57	Cutc	NM_025530.1	
scl0056248.2_157-S	41.7181	1.78	Ak3l	NM_021299.1	Mus musculus adenylate kinase 3 alpha-like (Ak3l), mRNA.
scl46585.22_265-S	41.7095	2.01	Vcl	NM_009502	Mus musculus vinculin (Vcl), mRNA.
scl000237.1_40-S	41.7084	1.35	Vkorc1	NM_178600.2	Mus musculus vitamin K epoxide reductase complex, subunit 1 (Vkorc1), mRNA.
scl051800.6_308-S	41.6178	1.45	Bok	NM_016778	Mus musculus Bcl-2-related ovarian killer protein (Bok), mRNA.
scl29041.8.1_24-S	41.5345	4.6	1810009M01Rik	NM_023056.2	Mus musculus RIKEN cDNA 1810009M01 gene (1810009M01Rik), mRNA.
scl011702.3_204-S	41.4724	18.9	Amd1	NM_009665.2	Mus musculus S-adenosylmethionine decarboxylase 1 (Amd1), mRNA.
scl39662.12.1_73-S	41.4102	1.45	Cdk5rap3	NM_030248.1	Mus musculus CDK5 regulatory subunit associated protein 3 (Cdk5rap3), mRNA.
scl0001335.1_10-S	41.4101	1.42	BC005682	NM_033562.2	Mus musculus cDNA sequence BC005682 (BC005682), mRNA.
scl54116.7.1_56-S	41.2997	10.03	Mtcp1	NM_010839.2	Mus musculus mature T-cell proliferation 1 (Mtcp1), mRNA.
scl00100273.2_58-S	41.2521	1.39	Osbpl9	NM_133885.1	Mus musculus oxysterol binding protein-like 9 (Osbpl9), mRNA.
scl066493.3_45-S	41.1645	1.48	Mrpl51	NM_025595.1	Mus musculus mitochondrial ribosomal protein L51 (Mrpl51), mRNA.
scl0028248.2_31-S	41.1584	65.93	Slco1a1	NM_013797.1	Mus musculus solute carrier organic anion transporter family, member 1a1 (Slco1a1), mRNA.
scl36137.8_30-S	41.1553	1.6	1300010K09Rik	NM_138303.1	Mus musculus RIKEN cDNA 1300010K09 gene (1300010K09Rik), mRNA.
scl0011658.2_92-S	41.1422	2.05	Alcam	NM_009655.1	Mus musculus activated leukocyte cell adhesion molecule (Alcam), mRNA.
scl39624.22.175_1-S	41.1026	2.68	Pparbp	NM_134027.1	Mus musculus peroxisome proliferator activated receptor binding protein (Pparbp), mRNA.
scl25332.2_140-S	41.0567	1.7	D4Bwg0951e	NM_026821.2	Mus musculus DNA segment, Chr 4, Brigham & Womens Genetics 0951 expressed (D4Bwg0951e), mRNA.
scl23528.23_0-S	41.0567	1.41	Mfn2	NM_133201.1	Mus musculus mitofusin 2 (Mfn2), mRNA.
scl0121022.3_149-S	41.0451	2.37	Mrps6	NM_080456.1	Mus musculus mitochondrial ribosomal protein S6 (Mrps6), mRNA.
scl0029869.2_146-S	40.9555	2.12	Ulk2	NM_013881.3	Mus musculus Unc-51 like kinase 2 (C. elegans) (Ulk2), mRNA.

scl38985.8_420-S	40.9283	1.31	Cd164	NM_016898.1	Mus musculus CD164 antigen (Cd164), mRNA.
scl068097.1_32-S	40.8096	6.44	6720463E02Rik	NM_026556.2	Mus musculus RIKEN cDNA 6720463E02 gene (6720463E02Rik), mRNA.
scl0050850.1_83-S	40.807	1.82	Spg4	NM_016962.1	Mus musculus spastic paraplegia 4 homolog (human) (Spg4), mRNA.
scl056258.4_7-S	40.7793	9.09	Hnrph2	NM_019868.2	Mus musculus heterogeneous nuclear ribonucleoprotein H2 (Hnrph2), mRNA.
scl27602.3.1_69-S	40.6715	9.18	Cxcl4	NM_019932.1	Mus musculus chemokine (C-X-C motif) ligand 4 (Cxcl4), mRNA.
scl074155.4_43-S	40.6602	1.48	1300002F13Rik	NM_133753.1	Mus musculus RIKEN cDNA 1300002F13 gene (1300002F13Rik), mRNA.
scl027556.1_96-S	40.6168	1.6	Clic4	XM_124389.1	Mus musculus chloride intracellular channel 4 (mitochondrial) (Clic4), mRNA.
scl00214498.1_240-S	40.4796	3.71	Hrpt2	NM_145991.1	
scl54163.9.1_4-S	40.4204	9.71	1110020L19Rik	NM_028633.1	Mus musculus RIKEN cDNA 1110020L19 gene (1110020L19Rik), mRNA.
scl016621.1_40-S	40.4044	1.47	Klkb1	NM_008455	Mus musculus kallikrein B, plasma 1 (Klkb1), mRNA.
scl0001351.1_1-S	40.3706	2.17	Afmid	NM_027827.2	Mus musculus arylformamidase (Afmid), mRNA.
scl0004104.1_48-S	40.2801	1.35	Ttc11	NM_025562.1	Mus musculus tetratricopeptide repeat domain 11 (Ttc11), mRNA.
scl22110.22.1_30-S	40.2155	1.5	Dhx36	NM_028136.1	Mus musculus DEAH (Asp-Glu-Ala-His) box polypeptide 36 (Dhx36), mRNA.
scl38781.10_357-S	40.1877	1.45	Zwint	NM_025635.1	
scl50306.11.1_132-S	40.1244	1.62	Slc22a1	NM_009202.2	Mus musculus solute carrier family 22 (organic cation transporter), member 1 (Slc22a1), mRNA.
scl22729.4.1_31-S	40.0945	2.73	Amigo	NM_146137.1	Mus musculus amphoterin induced gene and ORF (Amigo), mRNA.
scl014104.1_1-S	40.0814	2.22	Fasn	NM_007988.1	Mus musculus fatty acid synthase (Fasn), mRNA.
scl014979.1_326-S	40.0796	1.52	H2-Ke6	NM_013543.1	Mus musculus H2-K region expressed gene 6 (H2-Ke6), mRNA.
scl29249.10_180-S	39.9928	1.54	Tm4sf12	NM_173007.1	Mus musculus transmembrane 4 superfamily member 12 (Tm4sf12), mRNA.
scl018550.2_67-S	39.9726	1.62	Furin	NM_011046.1	Mus musculus furin (paired basic amino acid cleaving enzyme) (Furin), mRNA.
scl54178.10_163-S	39.9602	1.43	Mic2l1	NM_138309.1	Mus musculus MIC2 (monoclonal Imperial Cancer Research Fund 2)-like 1 (Mic2l1), mRNA.
scl41808.6_29-S	39.8865	1.37	Rab1	NM_008996.2	Mus musculus RAB1, member RAS oncogene family (Rab1), mRNA.
scl39705.9.188_7-S	39.8781	11.19	6820428D13	NM_177752.2	Mus musculus hypothetical protein 6820428D13 (6820428D13), mRNA.
scl0002141.1_52-S	39.8773	4.76	Cth	NM_145953.2	Mus musculus cystathionase (cystathionine gamma-lyase) (Cth), mRNA.
scl00228410.1_260-S	39.8766	4.83	Cstf3	NM_145529.1	Mus musculus cleavage stimulation factor, 3 pre-RNA, subunit 3 (Cstf3), mRNA.
scl0270802.1_64-S	39.8693	10.53	BC048403	NM_173022.2	Mus musculus cDNA sequence BC048403 (BC048403), mRNA.
scl22902.7.1_30-S	39.7862	1.97	Mrpl9	NM_030116.1	Mus musculus mitochondrial ribosomal protein L9 (Mrpl9), mRNA.
scl014870.5_30-S	39.6576	3.52	Gstp1	NM_013541.1	Mus musculus glutathione S-transferase, pi 1 (Gstp1), mRNA.
scl0077963.1_19-S	39.4568	2.6	Hook1	NM_030014.2	Mus musculus hook homolog 1 (Drosophila) (Hook1), mRNA.
scl47433.16.555_11-S	39.4529	1.88	Ghr	NM_010284.1	Mus musculus growth hormone receptor (Ghr), mRNA.
scl45915.9_517-S	39.4398	1.61	Dnase1l3	NM_007870.2	Mus musculus deoxyribonuclease 1-like 3 (Dnase1l3), mRNA.
scl00002.1_228_REVCOM	39.4259	2.54	2610024E20Rik	NM_146084.1	
scl50948.6.850_26-S	39.4102	1.6	Atp6v0e	NM_025272.1	Mus musculus ATPase, H+ transporting, V0 subunit (Atp6v0e), mRNA.
scl31674.3.1_6-S	39.3602	1.69	Apoc1	NM_007469.2	Mus musculus apolipoprotein C-I (Apoc1), mRNA.
scl0015547.2_255-S	39.3019	3.13	Htf9c	NM_008307.1	Mus musculus HpaII tiny fragments locus 9c (Htf9c), mRNA.
scl39898.9.1_17-S	39.2944	1.63	Pipox	NM_008952.1	Mus musculus pipecolic acid oxidase (Pipox), mRNA.
scl0002408.1_16-S	39.2848	2.19	Timm10	NM_013896.2	Mus musculus translocase of inner mitochondrial membrane 10 homolog (yeast) (Timm10), mRNA.
scl44090.4_104-S	39.2644	1.53	Nrn1	NM_153529.1	Mus musculus neuritin 1 (Nrn1), mRNA.
scl50009.17.1_17-S	39.0358	1.46	C2	NM_013484.1	Mus musculus complement component 2 (within H-2S) (C2), mRNA.
scl075007.4_290-S	38.9243	1.32	4930504E06Rik	NM_133858.2	Mus musculus RIKEN cDNA 4930504E06 gene (4930504E06Rik), mRNA.
scl29258.5.1_11-S	38.9171	6.8	Wnt2	NM_023653.3	Mus musculus wingless-related MMTV integration site 2 (Wnt2), mRNA.
scl0093765.1_4-S	38.8736	2.09	Ube2n	NM_080560.2	Mus musculus ubiquitin-conjugating enzyme E2N (Ube2n), mRNA.
scl011972.1_247-S	38.8354	1.47	Atp6v0d1	NM_013477.2	Mus musculus ATPase, H+ transporting, V0 subunit D isoform 1 (Atp6v0d1), mRNA.
scl0020365.1_201-S	38.7434	2.36	Serf1	NM_011353.1	Mus musculus small EDRK-rich factor 1 (Serf1), mRNA.
scl0210766.8_41-S	38.7328	8.2	C6.1A	NM_145956.2	Mus musculus c6.1a protein (C6.1A), mRNA.
scl32196.12_2-S	38.5992	13.62	Wee1	NM_009516.2	Mus musculus wee 1 homolog (S. pombe) (Wee1), mRNA.
scl019244.5_2-S	38.5624	1.3	Ptp4a2	NM_008974.2	Mus musculus protein tyrosine phosphatase 4a2 (Ptp4a2), mRNA.
scl54835.8.1_3-S	38.4603	1.86	D0HXS9928E	NM_138607.1	Mus musculus DNA segment, human DXS9928E (D0HXS9928E), mRNA.
scl0015081.1_185-S	38.4536	1.86	H3f3b	NM_008211.2	Mus musculus H3 histone, family 3B (H3f3b), mRNA.
scl54205.32.1_100-S	38.4249	8.62	Atp11c	XM_135900.3	
scl0110265.1_302-S	38.3812	24.68	Msra	NM_026322.2	Mus musculus methionine sulfoxide reductase A (Msra), mRNA.
scl0014751.2_296-S	38.3476	1.53	Gpi1	NM_008155.1	Mus musculus glucose phosphate isomerase 1 (Gpi1), mRNA.
scl00104479.1_248-S	38.3366	2.71	BC018601	NM_134033.1	Mus musculus cDNA sequence BC018601 (BC018601), mRNA.
scl070425.3_227-S	38.3105	1.4	Csnk1g3	NM_152809.1	Mus musculus casein kinase 1, gamma 3 (Csnk1g3), mRNA.
scl0002087.1_19-S	38.3068	1.74	BC037135	NM_173763.2	Mus musculus cDNA sequence BC037135 (BC037135), mRNA.
scl34274.4_380-S	38.297	1.45	4632417N05Rik	NM_028725.1	Mus musculus RIKEN cDNA 4632417N05 gene (4632417N05Rik), mRNA.

scl21980.4.1_109-S	38.2791	1.47	1700021C14Rik	XM_355444.1	Mus musculus RIKEN cDNA 1700021C14 gene (1700021C14Rik), mRNA.
scl33169.8.1_16-S	38.2705	1.58	2310079N02Rik	NM_025636.1	Mus musculus RIKEN cDNA 2310079N02 gene (2310079N02Rik), mRNA.
scl49378.5_417-S	38.2557	1.56	Snap29	NM_023348.3	Mus musculus synaptosomal-associated protein (Snap29), mRNA.
scl0068421.2_70-S	38.0946	4.26	0910001K20Rik	NM_026719.1	Mus musculus RIKEN cDNA 0910001K20 gene (0910001K20Rik), mRNA.
scl013445.1_266-S	38.0813	1.41	Cdk2ap1	NM_013812.1	Mus musculus CDK2 (cyclin-dependent kinase 2)-associated protein 1 (Cdk2ap1), mRNA.
scl0225256.19_24-S	38.0498	9.65	Dsg1b	NM_181682.1	Mus musculus desmoglein 1 beta (Dsg1b), mRNA.
scl0019211.1_81-S	38.0168	2.09	Pten	NM_008960.1	Mus musculus phosphatase and tensin homolog (Pten), mRNA.
scl54440.26.1_53-S	38.0061	3.02	Hdac6	NM_010413	Mus musculus histone deacetylase 6 (Hdac6), mRNA.
scl0012091.2_274-S	37.9884	1.46	Glb1	NM_009752.1	Mus musculus galactosidase, beta 1 (Glb1), mRNA.
scl0002653.1_3-S	37.9788	3.91	Gcipip	NM_026780.1	Mus musculus GCIP-interacting protein p29 (Gcipip), mRNA.
scl076295.3_96-S	37.9646	2.11	Atp11b	XM_358349.1	Mus musculus ATPase, Class VI, type 11B (Atp11b), mRNA.
scl28198.16.9_0-S	37.8425	2.57	4933424B01Rik	NM_138757.1	Mus musculus RIKEN cDNA 4933424B01 gene (4933424B01Rik), mRNA.
scl0001438.1_12-S	37.7821	1.69	Copz2	NM_019877.1	Mus musculus coatomer protein complex, subunit zeta 2 (Copz2), mRNA.
scl056508.28_32-S	37.7518	8.82	Rapgef4	NM_019688.1	Mus musculus Rap guanine nucleotide exchange factor (GEF) 4 (Rapgef4), mRNA.
scl40876.7.1_108-S	37.7239	2.02	Rad52b	NM_025654.1	Mus musculus RAD52 homolog B (S. cerevisiae) (Rad52b), mRNA.
scl0080898.1_95-S	37.7058	9.71	Arts1	NM_030711.2	Mus musculus type 1 tumor necrosis factor receptor shedding aminopeptidase regulator (Arts1), mRNA.
scl30691.10.4_70-S	37.6999	1.6	2210013K02Rik	NM_023712	Mus musculus RIKEN cDNA 2210013K02 gene (2210013K02Rik), mRNA.
scl42936.6.9_32-S	37.6127	1.74	Ahsa1	NM_146036.1	Mus musculus AHA1, activator of heat shock 90kDa protein ATPase homolog 1 (yeast) (Ahsa1), mRNA.
scl0001405.1_534-S	37.61	5.68	Spag9	NM_027569.1	Mus musculus sperm associated antigen 9 (Spag9), mRNA.
scl0003706.1_56-S	37.6062	9.93	4833420G17Rik	NM_026127	Mus musculus RIKEN cDNA 4833420G17 gene (4833420G17Rik), mRNA.
scl35797.7.1_123-S	37.5603	1.36	Commd4	NM_025417.1	Mus musculus COMM domain containing 4 (Commd4), mRNA.
scl0003818.1_1-S	37.5215	8.15	D10Ertd610e	NM_028027.1	Mus musculus DNA segment, Chr 10, ERATO Doi 610, expressed (D10Ertd610e), mRNA.
scl35165.21_648-S	37.4968	1.92	Fyco1	NM_148925.1	Mus musculus FYVE and coiled-coil domain containing 1 (Fyco1), mRNA.
scl0108112.2_25-S	37.4887	3.32	Eif4ebp3	NM_201256.2	Mus musculus eukaryotic translation initiation factor 4E binding protein 3 (Eif4ebp3), mRNA.
scl47043.17.1_62-S	37.4807	2.08	Bop1	NM_013481.1	Mus musculus block of proliferation 1 (Bop1), mRNA.
scl26959.5.1_79-S	37.3971	1.29	2510048O06Rik	NM_025624.1	Mus musculus RIKEN cDNA 2510048O06 gene (2510048O06Rik), mRNA.
scl0004139.1_41-S	37.3289	6.43	Asrij	NM_023429.2	Mus musculus asrij protein (Asrij), mRNA.
scl22956.4.1_1-S	37.328	1.33	Jtb	NM_206924.1	Mus musculus jumping translocation breakpoint (Jtb), mRNA.
scl0027059.2_196-S	37.3217	1.38	Sh3d19	NM_012059.2	Mus musculus SH3 domain protein D19 (Sh3d19), mRNA.
scl073373.8_41-S	37.3142	1.48	1700048E23Rik	NM_028521.1	Mus musculus RIKEN cDNA 1700048E23 gene (1700048E23Rik), mRNA.
scl00226016.2_58-S	37.3071	6.38	5730446C15Rik	NM_146096.2	Mus musculus RIKEN cDNA 5730446C15 gene (5730446C15Rik), mRNA.
scl21094.18.34_0-S	37.2625	1.53	Lrrc8	NM_177725.2	Mus musculus leucine-rich repeat-containing 8 (Lrrc8), mRNA.
scl27119.4.1_2-S	37.2507	1.37	Polr2j	NM_011293.1	Mus musculus polymerase (RNA) II (DNA directed) polypeptide J (Polr2j), mRNA.
scl53313.7_541-S	37.2015	2.98	Gna14	NM_008137.2	Mus musculus guanine nucleotide binding protein, alpha 14 (Gna14), mRNA.
scl0001042.1_14-S	37.0785	4.05	Mrps25	NM_025578.2	Mus musculus mitochondrial ribosomal protein S25 (Mrps25), mRNA.
scl0003111.1_1260-S	37.0609	3.23	Pfkfb3	NM_133232.1	Mus musculus 6-phosphofructo-2-kinase/fructose-2,6-biphosphatase 3 (Pfkfb3), mRNA.
scl0003179.1_0-S	36.8875	3.49	2310047O13Rik	NM_024185.3	Mus musculus RIKEN cDNA 2310047O13 gene (2310047O13Rik), mRNA.
scl0004146.1_45-S	36.8625	1.71	Dhrs8	NM_053262.2	Mus musculus dehydrogenase/reductase (SDR family) member 8 (Dhrs8), mRNA.
scl0068730.1_249-S	36.8473	1.44	1110032N12Rik	NM_026824.2	Mus musculus RIKEN cDNA 1110032N12 gene (1110032N12Rik), mRNA.
scl0017357.2_64-S	36.8353	3.24	Mlp	NM_010807.2	Mus musculus MARCKS-like protein (Mlp), mRNA.
scl070612.1_126-S	36.8302	1.66	5730494N06Rik	NM_027478.1	Mus musculus RIKEN cDNA 5730494N06 gene (5730494N06Rik), mRNA.
scl098366.1_53-S	36.826	3.34	Smap1	NM_028534.1	Mus musculus stromal membrane-associated protein 1 (Smap1), mRNA.
scl0013096.1_55-S	36.811	1.39	Cyp2c37	NM_010001.1	Mus musculus cytochrome P450, family 2. subfamily c, polypeptide 37 (Cyp2c37), mRNA.
scl066475.2_30-S	36.7992	2.94	Rps23	NM_024175	Mus musculus ribosomal protein S23 (Rps23), mRNA.
scl53577.7_510-S	36.7701	4.08	Prps2	NM_026662.2	Mus musculus phosphoribosyl pyrophosphate synthetase 2 (Prps2), mRNA.
scl020317.1_68-S	36.7345	1.41	Serpinf1	NM_011340.2	Mus musculus serine (or cysteine) proteinase inhibitor, clade F, member 1 (Serpinf1), mRNA.
scl000603.1_82-S	36.724	1.55	1810047C23Rik	NM_138668.1	Mus musculus RIKEN cDNA 1810047C23 gene (1810047C23Rik), mRNA.
scl015903.3_124-S	36.7126	1.62	Idb3	NM_008321.1	Mus musculus inhibitor of DNA binding 3 (Idb3), mRNA.
scl44304.18_267-S	36.6937	1.69	Gtpbp4	NM_027000.2	Mus musculus GTP binding protein 4 (Gtpbp4), mRNA.
scl067245.8_3-S	36.6677	3.74	Peli1	NM_030015.1	Mus musculus pellino 1 (Peli1), mRNA.
scl22547.4.5_11-S	36.6046	1.37	Adh1	NM_007409.2	Mus musculus alcohol dehydrogenase 1 (class I) (Adh1), mRNA.
scl0229905.15_25-S	36.5835	2.73	BC037135	NM_173763.2	Mus musculus cDNA sequence BC037135 (BC037135), mRNA.
scl52435.3.1_22-S	36.5236	1.32	Mrpl43	NM_053164.2	Mus musculus mitochondrial ribosomal protein L43 (Mrpl43), nuclear gene encoding mitochondrial protein, mRNA.
scl019942.4_1-S	36.523	1.29	Rpl27	NM_011289	Mus musculus ribosomal protein L27 (Rpl27), mRNA.
scl071782.13_18-S	36.493	10.17	D5Ertd585e	NM_027922.1	
scl41695.7_133-S	36.4434	2.92	Pank3	NM_145962.1	Mus musculus pantothenate kinase 3 (Pank3), mRNA.

scl0001073.1_120-S	36.4318	1.63	Cxcl12	NM_021704.1	Mus musculus chemokine (C-X-C motif) ligand 12 (Cxcl12), transcript variant 2, mRNA.
scl41419.7.1_12-S	36.3804	8.6	A730055C05Rik	NM_177392.1	Mus musculus RIKEN cDNA A730055C05 gene (A730055C05Rik), mRNA.
scl29171.13.1_11-S	36.338	14.45	Chchd3	NM_025336.1	Mus musculus coiled-coil-helix-coiled-coil-helix domain containing 3 (Chchd3), mRNA.
scl00380773.1_71-S	36.3375	1.34	1810035L17Rik	XM_354684.1	
scl41212.9.1_219-S	36.3113	5.87	Rab34	NM_033475.2	Mus musculus RAB34, member of RAS oncogene family (Rab34), mRNA.
scl26412.9_31-S	36.2483	1.97	Sult1d1	NM_016771.2	Mus musculus sulfotransferase family 1D, member 1 (Sult1d1), mRNA.
scl017776.1_270-S	36.2365	1.43	Mast2	NM_008641.1	Mus musculus microtubule associated serine/threonine kinase 2 (Mast2), mRNA.
scl30509.9.1_6-S	36.2361	1.98	Sirt3	NM_022433.1	Mus musculus sirtuin 3 (silent mating type information regulation 2, homolog) 3 (S. cerevisiae) (Sirt3), mRNA.
scl0076895.2_316-S	36.1352	7.84	Bicd2	NM_029791.2	Mus musculus bicaudal D homolog 2 (Drosophila) (Bicd2), mRNA.
scl39620.4_46-S	36.1326	1.31	1810046J19Rik	NM_025559.1	Mus musculus RIKEN cDNA 1810046J19 gene (1810046J19Rik), mRNA.
scl18778.5_150-S	36.1208	2.8	2810002D13Rik	NM_025657.2	Mus musculus RIKEN cDNA 2810002D13 gene (2810002D13Rik), mRNA.
scl078038.2_5-S	36.0694	1.64	Mccc2	XM_127493.3	
scl24777.7.1_203-S	36.0553	1.31	Akr7a5	NM_025337.2	Mus musculus aldo-keto reductase family 7, member A5 (aflatoxin aldehyde reductase) (Akr7a5), mRNA.
scl023825.3_146-S	36.04	1.62	Banf1	NM_011793.2	Mus musculus barrier to autointegration factor 1 (Banf1), mRNA.
scl00319800.2_168-S	35.931	10.28	C730048C13Rik	NM_177002.2	Mus musculus RIKEN cDNA C730048C13 gene (C730048C13Rik), mRNA.
scl49765.36_30-S	35.9148	5.29	Ptprs	NM_011218.1	Mus musculus protein tyrosine phosphatase, receptor type, S (Ptprs), mRNA.
scl21518.7.1_44-S	35.9116	1.45	Nola1	NM_026578.1	Mus musculus nucleolar protein family A, member 1 (H/ACA small nucleolar RNPs) (Nola1), mRNA.
gi_21070949_ref_NM_01	35.9078	2.68	Ubc	XM_147315.1	Mus musculus ubiquitin C (Ubc), mRNA.
scl0067179.2_115-S	35.9063	5.22	2610528H13Rik	NM_145944.1	Mus musculus RIKEN cDNA 2610528H13 gene (2610528H13Rik), mRNA.
scl059050.2_22-S	35.8524	1.34	5730427N09Rik	NM_021552.1	Mus musculus RIKEN cDNA 5730427N09 gene (5730427N09Rik), mRNA.
scl25467.19.1_1-S	35.7928	3.84	1300002K09Rik	XM_283962.2	Mus musculus RIKEN cDNA 1300002K09 gene (1300002K09Rik), mRNA.
scl34232.7.1_77-S	35.7911	1.73	Car5a	NM_007608.1	Mus musculus carbonic anhydrase 5a, mitochondrial (Car5a), mRNA.
scl0268741.7_16-S	35.7631	1.88	5730589K01Rik	NM_023434	Mus musculus RIKEN cDNA 5730589K01 gene (5730589K01Rik), mRNA.
scl21990.8.1_193-S	35.7604	1.67	Prcc	NM_033573.1	Mus musculus papillary renal cell carcinoma (translocation-associated) (Prcc), mRNA.
scl15945.4.1_0-S	35.7475	1.31	1110021H02Rik	NM_025388.1	Mus musculus RIKEN cDNA 1110021H02 gene (1110021H02Rik), mRNA.
scl30153.32_359-S	35.7339	1.73	Mgam	XM_133071.2	
scl50998.1_28-S	35.6952	1.31	Mrps34	NM_023260.1	Mus musculus mitochondrial ribosomal protein S34 (Mrps34), mRNA.
scl015500.15_97-S	35.6543	11.7	Hsf2	NM_008297.2	Mus musculus heat shock factor 2 (Hsf2), mRNA.
scl48728.3.1_20-S	35.6387	1.6	2410018G20Rik	XM_148413.1	Mus musculus RIKEN cDNA 2410018G20 gene (2410018G20Rik), mRNA.
scl40067.6.1_43-S	35.6251	2.03	2310004I24Rik	NM_025510.1	Mus musculus RIKEN cDNA 2310004I24 gene (2310004I24Rik), mRNA.
scl47912.4.1_215-S	35.6056	8.77	Mal2	NM_178920.2	
scl066552.3_24-S	35.5447	9.45	2010106G01Rik	NM_023220.1	Mus musculus RIKEN cDNA 2010106G01 gene (2010106G01Rik), mRNA.
scl40724.4.1_15-S	35.5447	1.54	Mrps7	NM_025305.1	Mus musculus mitchondrial ribosomal protein S7 (Mrps7), mRNA.
scl067153.8_8-S	35.4821	1.85	2610207P08Rik	XM_127746.2	Mus musculus RIKEN cDNA 2610207P08 gene (2610207P08Rik), mRNA.
scl0067246.1_211-S	35.4237	7.05	2810474O19Rik	XM_132966.3	Mus musculus RIKEN cDNA 2810474O19 gene (2810474O19Rik), mRNA.
scl0011552.1_302-S	35.3712	2.11	Adra2b	NM_009633.2	Mus musculus adrenergic receptor, alpha 2b (Adra2b), mRNA.
scl21831.10.1_25-S	35.3109	1.64	Ecm1	NM_007899	Mus musculus extracellular matrix protein 1 (Ecm1), mRNA.
scl014073.1_90-S	35.2632	1.34	Faah	NM_010173.2	Mus musculus fatty acid amide hydrolase (Faah), mRNA.
scl47735.7_191-S	35.2601	1.58	Syngr1	NM_207708.1	Mus musculus synaptogyrin 1 (Syngr1), transcript variant 1b, mRNA.
scl41644.4_98-S	35.2514	1.42	Crsp9	NM_025426.2	Mus musculus cofactor required for Sp1 transcriptional activation, subunit 9 (Crsp9), mRNA.
scl25510.19.1_73-S	35.2457	2.98	Npr2	NM_173788.2	Mus musculus natriuretic peptide receptor 2 (Npr2), mRNA.
scl0012055.2_161-S	35.2124	1.39	Bcl7c	NM_009746	Mus musculus B-cell CLL/lymphoma 7C (Bcl7c), mRNA.
scl072587.6_94-S	35.1696	9.43	2700050F09Rik	NM_028291.2	Mus musculus RIKEN cDNA 2700050F09 gene (2700050F09Rik), mRNA.
scl0019264.1_208-S	35.1624	9.62	Ptprc	NM_011210.1	Mus musculus protein tyrosine phosphatase, receptor type, C (Ptprc), mRNA.
gi_32129296_ref_NM_00	35.1546	1.63	Rpl13a	NM_009438.3	Mus musculus ribosomal protein L13a (Rpl13a), mRNA.
scl24957.6.1_12-S	35.1541	1.3	Psmb2	NM_011970.2	Mus musculus proteasome (prosome, macropain) subunit, beta type 2 (Psmb2), mRNA.
scl0001814.1_15-S	35.0991	1.63	Magmas	NM_025571.1	Mus musculus mitochondria-associated protein involved in granulocyte-macrophage colony-stimulating factor signal transduction (Magmas), mRNA.
scl0072544.1_107-S	35.0853	8.91	Exosc6	NM_028274.1	Mus musculus exosome component 6 (Exosc6), mRNA.
scl068763.4_12-S	35.0641	1.69	1110038B12Rik	XM_359415.1	Mus musculus RIKEN cDNA 1110038B12 gene (1110038B12Rik), mRNA.
scl068877.8_322-S	35.0571	1.33	Maf1	NM_026859.2	Mus musculus MAF1 homolog (yeast) (Maf1), mRNA.
scl50839.12.1_25-S	35.0422	9.12	Bing4	NM_020603.2	Mus musculus BING4 protein (Bing4), mRNA.
scl28771.4_14-S	35.0343	1.35	Spr	NM_011467.1	Mus musculus sepiapterin reductase (Spr), mRNA.
scl35013.8.1_0-S	35.0185	1.39	2810037C03Rik	NM_024240.3	Mus musculus RIKEN cDNA 2810037C03 gene (2810037C03Rik), mRNA.
scl51536.11_7-S	35.0089	1.45	Etf1	NM_144866.2	Mus musculus eukaryotic translation termination factor 1 (Etf1), mRNA.
scl014375.11_27-S	35.004	1.85	G22p1	NM_010247.1	Mus musculus thyroid autoantigen (G22p1), mRNA.
scl41586.4_460-S	34.997	1.45	D11Ertd497e	NM_029976.2	Mus musculus DNA segment, Chr 11, ERATO Doi 497, expressed (D11Ertd497e), mRNA.

scl23453.4_99-S	34.9745	1.4	1200015A19Rik	NM_026388.1	Mus musculus RIKEN cDNA 1200015A19 gene (1200015A19Rik), mRNA.
scl37374.5.1_9-S	34.9664	10.87	Hsd17b9	NM_013786.2	Mus musculus hydroxysteroid (17-beta) dehydrogenase 9 (Hsd17b9), mRNA.
scl31469.3.28_7-S	34.9586	1.49	D7Rp2e	NM_033080.1	Mus musculus DNA segment, Chr 7, Roswell Park 2 complex, expressed (D7Rp2e), mRNA.
scl50887.7_31-S	34.9354	2.01	Tex27	NM_148926.1	Mus musculus testis expressed gene 27 (Tex27), mRNA.
scl46955.19_187-S	34.9123	1.8	B230369L08Rik	NM_194342	
scl079264.1_0-S	34.9004	9.98	Ccm1	NM_030675.2	Mus musculus cerebral cavernous malformations 1 (Ccm1), mRNA.
scl29342.18_151-S	34.881	1.59	1810060J02Rik	NM_025911.1	Mus musculus RIKEN cDNA 1810060J02 gene (1810060J02Rik), mRNA.
scl50581.4.1_28-S	34.8346	1.53	2310045B01Rik	NM_025538.1	Mus musculus RIKEN cDNA 2310045B01 gene (2310045B01Rik), mRNA.
scl28581.11_0-S	34.828	8.9	Pdzrn3	NM_018884.1	
scl072121.12_86-S	34.8223	2.48	2010308M01Rik	XM_131083.2	Mus musculus RIKEN cDNA 2010308M01 gene (2010308M01Rik), mRNA.
scl059022.3_20-S	34.8218	1.32	Edf1	NM_021519.1	Mus musculus endothelial differentiation-related factor 1 (Edf1), mRNA.
scl53032.9_15-S	34.818	1.9	Habp2	NM_146101.1	Mus musculus hyaluronic acid binding protein 2 (Habp2), mRNA.
scl050790.1_73-S	34.8152	3.21	Acsl4	NM_207625.1	Mus musculus acyl-CoA synthetase long-chain family member 4 (Acsl4), transcript variant 2, mRNA.
scl000906.1_50-S	34.8082	1.82	Acadl	NM_007381.2	Mus musculus acetyl-Coenzyme A dehydrogenase, long-chain (Acadl), mRNA.
scl31073.10_61-S	34.753	1.54	3110005P07Rik	NM_022985.3	
scl056347.10_39-S	34.7313	1.77	Eif3s8	NM_146200	Mus musculus eukaryotic translation initiation factor 3, subunit 8 (Eif3s8), mRNA.
scl066659.9_78-S	34.714	1.55	Acp6	NM_019800.1	Mus musculus acid phosphatase 6, lysophosphatidic (Acp6), mRNA.
scl0066989.1_296-S	34.6937	1.59	2410004N11Rik	NM_025888.2	Mus musculus RIKEN cDNA 2410004N11 gene (2410004N11Rik), mRNA.
scl31031.8.1_28-S	34.6921	1.57	1810020D17Rik	NM_183251.2	Mus musculus RIKEN cDNA 1810020D17 gene (1810020D17Rik), mRNA.
scl099712.1_51-S	34.6319	1.6	Cept1	NM_133869.2	Mus musculus choline/ethanolaminephosphotransferase 1 (Cept1), mRNA.
scl0228983.14_82-S	34.6306	1.46	Osbpl2	NM_144500.1	Mus musculus oxysterol binding protein-like 2 (Osbpl2), mRNA.
scl21206.2.1_228-S	34.6143	1.76	Bri3	NM_018772.1	Mus musculus brain protein I3 (Bri3), mRNA.
scl0170716.1_183-S	34.5952	1.47	Cyp4f13	NM_130882.1	Mus musculus cytochrome P450, family 4, subfamily f, polypeptide 13 (Cyp4f13), mRNA.
scl17509.2_142-S	34.593	1.79	9930028C20Rik	NM_178691.2	Mus musculus RIKEN cDNA 9930028C20 gene (9930028C20Rik), mRNA.
scl18683.9.1_140-S	34.5667	3.74	Zc3hdc8	NM_020594.1	Mus musculus zinc finger CCCH type domain containing 8 (Zc3hdc8), mRNA.
scl22158.8.1_12-S	34.5561	2.84	Exosc8	NM_027148.2	Mus musculus exosome component 8 (Exosc8), mRNA.
scl49959.3.1_23-S	34.5556	1.48	Rpp21	NM_026308.1	Mus musculus ribonuclease P 21kDa subunit (human) (Rpp21), mRNA.
scl27541.6_227-S	34.552	5.67	2310057D15Rik	NM_026421.1	Mus musculus RIKEN cDNA 2310057D15 gene (2310057D15Rik), mRNA.
scl33375.10_5-S	34.493	1.54	Vps4a	NM_126165.1	Mus musculus vacuolar protein sorting 4a (yeast) (Vps4a), mRNA.
scl0067949.2_13-S	34.4877	1.69	Mki67ip	NM_026472.2	Mus musculus Mki67 (FHA domain) interacting nucleolar phosphoprotein (Mki67ip), mRNA.
scl020182.9_313-S	34.4743	1.63	Rxrb	NM_011306.2	Mus musculus retinoid X receptor beta (Rxrb), mRNA.
scl43410.8_467-S	34.4699	1.75	1110057K04Rik	NM_172401.2	Mus musculus RIKEN cDNA 1110057K04 gene (1110057K04Rik), mRNA.
scl46866.15_163-S	34.3895	1.42	Brd1	XM_128275.4	Mus musculus bromodomain containing 1 (Brd1), mRNA.
scl0003356.1_0-S	34.3833	1.83	Mrps5	NM_029963.2	Mus musculus mitochondrial ribosomal protein S5 (Mrps5), mRNA.
scl0068349.1_0-S	34.3784	1.86	Ndufs3	XM_130347.1	Mus musculus NADH dehydrogenase (ubiquinone) Fe-S protein 3 (Ndufs3), mRNA.
scl40004.19.1_140-S	34.256	1.27	Acadvl	NM_017366	Mus musculus acyl-Coenzyme A dehydrogenase, very long chain (Acadvl), mRNA.
scl0226026.1_6-S	34.1517	14.96	Smc5l1	NM_153808.1	Mus musculus SMC5 structural maintenance of chromosomes 5-like 1 (yeast) (Smc5l1), mRNA.
scl52451.21_19-S	34.1194	1.36	Chuk	NM_007700.1	Mus musculus conserved helix-loop-helix ubiquitous kinase (Chuk), mRNA.
scl0003035.1_7-S	34.0751	5.3	Pex16	NM_145122.1	Mus musculus peroxisome biogenesis factor 16 (Pex16), mRNA.
scl17695.4.1_211-S	34.0732	2.28	Neu2	NM_015750.1	Mus musculus neuraminidase 2 (Neu2), mRNA.
scl45614.46.1_71-S	34.0291	8.36	Tep1	NM_009351.1	Mus musculus telomerase associated protein 1 (Tep1), mRNA.
scl37341.3.1_8-S	33.9979	1.36	Bloc1s1	NM_015740.2	
scl00230249.2_159-S	33.9798	1.75	AI314180	NM_172381.2	Mus musculus expressed sequence AI314180 (AI314180), mRNA.
scl20979.15_678-S	33.971	2.62	Kynu	NM_027552.1	Mus musculus kynureninase (L-kynurenine hydrolase) (Kynu), mRNA.
scl0015982.2_105-S	33.9276	10.65	Ifrd1	NM_013562.1	Mus musculus interferon-related developmental regulator 1 (Ifrd1), mRNA.
scl0098910.2_26-S	33.9087	2.87	Usp6nl	NM_181399.2	
scl0001909.1_11-S	33.9041	3.36	Dscr3	NM_007834.1	Mus musculus Down syndrome critical region gene 3 (Dscr3), mRNA.
scl39882.5_436-S	33.8935	1.44	AI316787	NM_199199.1	Mus musculus expressed sequence AI316787 (AI316787), mRNA.
scl21409.6.1_1-S	33.8616	2.95	Dnase2b	NM_019957.2	Mus musculus deoxyribonuclease II beta (Dnase2b), mRNA.
scl00231464.2_19-S	33.8509	7.73	Cnot6l	NM_144910.1	
scl45540.5.1_3-S	33.7872	2.56	1500005A01Rik	NM_033146.1	Mus musculus RIKEN cDNA 1500005A01 gene (1500005A01Rik), mRNA.
scl073998.24_21-S	33.7828	5.14	Herc3	NM_028705.2	Mus musculus hect domain and RLD 3 (Herc3), mRNA.
scl47056.10.1_17-S	33.7621	1.32	Tsta3	NM_031201.1	Mus musculus tissue specific transplantation antigen P35B (Tsta3), mRNA.
scl000730.1_110-S	33.7437	5.6	Gcdh	NM_008097	Mus musculus glutaryl-Coenzyme A dehydrogenase (Gcdh), mRNA.
scl45393.10_71-S	33.7126	1.4	Bnip3l	NM_009761.2	Mus musculus BCL2/adenovirus E1B 19kDa-interacting protein 3-like (Bnip3l), mRNA.
scl26144.4_263-S	33.696	2.08	Hspb8	NM_030704.1	Mus musculus heat shock 27kDa protein 8 (Hspb8), mRNA.

scl068066.1_115-S	33.6096	1.27	D11Ertd333e	NM_026542.1	
scl0067440.1_72-S	33.6046	1.83	Papd1	NM_026157.1	Mus musculus PAP associated domain containing 1 (Papd1), mRNA.
scl50565.20_286-S	33.6009	1.68	Man2a1	NM_008549.1	Mus musculus mannosidase 2, alpha 1 (Man2a1), mRNA.
scl0067993.2_43-S	33.5909	2.47	Nudt12	NM_026497.1	Mus musculus nudix (nucleoside diphosphate linked moiety X)-type motif 12 (Nudt12), mRNA.
scl056485.12_106-S	33.5846	13.32	Slc2a5	NM_019741.2	Mus musculus solute carrier family 2 (facilitated glucose transporter), member 5 (Slc2a5), mRNA.
scl0011477.2_228-S	33.5527	1.56	Acvr1	NM_007394.2	Mus musculus activin A receptor, type 1 (Acvr1), mRNA.
scl46515.12.62_5-S	33.5489	13.51	Actr8	NM_027493.1	Mus musculus ARP8 actin-related protein 8 homolog (S. cerevisiae) (Actr8), mRNA.
scl0068379.1_280-S	33.5391	1.52	Ciz1	NM_028412.1	Mus musculus CDKN1A interacting zinc finger protein 1 (Ciz1), mRNA.
scl016396.2_0-S	33.4993	2.29	Itch	XM_192925.3	Mus musculus itchy (Itch), mRNA.
scl022142.1_236-S	33.4971	11.44	Tuba1	NM_011653	Mus musculus tubulin, alpha 1 (Tuba1), mRNA.
scl016822.21_5-S	33.4723	14.01	Lcp2	NM_010696.2	Mus musculus lymphocyte cytosolic protein 2 (Lcp2), mRNA.
scl0268903.1_0-S	33.3872	8.59	Nrip1	NM_173440.1	Mus musculus nuclear receptor interacting protein 1 (Nrip1), mRNA.
scl00102058.2_282-S	33.3719	1.59	Exoc8	NM_198103.1	
scl0094061.1_118-S	33.3613	1.53	Mrpl1	NM_053158.1	Mus musculus mitochondrial ribosomal protein L1 (Mrpl1), mRNA.
scl065111.1_137-S	33.355	1.3	Dap3	NM_022994.2	Mus musculus death associated protein 3 (Dap3), mRNA.
scl35262.19_0-S	33.3101	1.36	1300006C19Rik	XM_358385.1	
scl0001050.1_29-S	33.2668	1.6	Grcc2f	NM_013536.1	Mus musculus gene rich cluster, C2f gene (Grcc2f), mRNA.
scl42037.39_16-S	33.2533	1.61	Cdc42bpb	NM_183016.1	Mus musculus Cdc42 binding protein kinase beta (Cdc42bpb), mRNA.
scl53337.7_252-S	33.1753	19.61	Keg1	NM_029550.1	Mus musculus kidney expressed gene 1 (Keg1), mRNA.
scl31320.8.1_2-S	33.1478	1.43	Csrp3	NM_013808.3	Mus musculus cysteine and glycine-rich protein 3 (Csrp3), mRNA.
scl24592.15.1_12-S	33.0746	2.86	AF155546	NM_021303.1	Mus musculus cDNA sequence AF155546 (AF155546), mRNA.
scl52531.6_219-S	33.0487	1.55	Pank1	NM_023792.1	Mus musculus pantothenate kinase 1 (Pank1), mRNA.
scl38302.14.1_13-S	33.0291	2.6	Prim1	NM_008921.1	Mus musculus DNA primase, p49 subunit (Prim1), mRNA.
scl42371.18.1_30-S	33.0009	1.58	Pygl	NM_133198.1	Mus musculus liver glycogen phosphorylase (Pygl), mRNA.
scl072040.1_282-S	32.9928	1.49	Mucdhl	NM_028069.1	Mus musculus mucin and cadherin like (Mucdhl), mRNA.
scl0030934.2_247-S	32.9859	1.56	Tor1b	NM_133673.2	Mus musculus torsin family 1, member B (Tor1b), mRNA.
scl00329002.1_170-S	32.9795	2.29	Zfp236	NM_177832.2	Mus musculus zinc finger protein 236 (Zfp236), mRNA.
scl069583.1_127-S	32.9733	1.74	Tnfsf13	NM_023517.1	Mus musculus tumor necrosis factor (ligand) superfamily, member 13 (Tnfsf13), mRNA.
scl067667.2_1-S	32.9083	8.85	4930562C03Rik	XM_134612.3	Mus musculus RIKEN cDNA 4930562C03 gene (4930562C03Rik), mRNA.
scl0073046.2_136-S	32.8751	1.63	2900070E19Rik	NM_028419.1	Mus musculus RIKEN cDNA 2900070E19 gene (2900070E19Rik), mRNA.
scl0002499.1_186-S	32.868	1.89	Fbxo4	NM_134099	Mus musculus F-box only protein 4 (Fbxo4), mRNA.
scl49587.9_193-S	32.8567	2.92	Sfrs7	NM_146083.1	Mus musculus splicing factor, arginine/serine-rich 7 (Sfrs7), mRNA.
scl17276.4_366-S	32.8003	12.17	Dpt	NM_019759.1	Mus musculus dermatopontin (Dpt), mRNA.
scl30453.23.6_9-S	32.7724	6.73	Cars	NM_013742.2	Mus musculus cysteinyl-tRNA synthetase (Cars), mRNA.
scl0001834.1_12-S	32.7648	9.67	0610037P05Rik	XM_148359.1	Mus musculus RIKEN cDNA 0610037P05 gene (0610037P05Rik), mRNA.
scl38726.3.1_35-S	32.7538	1.29	Cstb	NM_007793.2	Mus musculus cystatin B (Cstb), mRNA.
scl00320487.1_23-S	32.7495	1.77	D930036F22Rik	NM_177171.3	Mus musculus RIKEN cDNA D930036F22 gene (D930036F22Rik), mRNA.
scl077619.4_0-S	32.7459	10.12	C330008K14Rik	XM_128979	
scl27876.6.1_53-S	32.6496	1.45	2810021O14Rik	NM_025480.2	Mus musculus RIKEN cDNA 2810021O14 gene (2810021O14Rik), mRNA.
scl0001537.1_132-S	32.617	9.47	Rtn4	NM_194054.1	Mus musculus reticulon 4 (Rtn4), transcript variant 5, mRNA.
scl0012846.1_253-S	32.6073	1.48	Comt	XM_147265.1	Mus musculus catechol-O-methyltransferase (Comt), mRNA.
scl0066870.1_13-S	32.5976	1.43	1200009K13Rik	NM_025814.1	Mus musculus RIKEN cDNA 1200009K13 gene (1200009K13Rik), mRNA.
scl0001551.1_0-S	32.4652	9.14	4933407N01Rik	NM_025745.2	Mus musculus RIKEN cDNA 4933407N01 gene (4933407N01Rik), mRNA.
scl0227290.1_240-S	32.4607	1.27	Aamp	NM_146110.1	Mus musculus angio-associated migratory protein (Aamp), mRNA.
scl18197.5_22-S	32.4122	4.63	Rgs19	NM_026446.2	Mus musculus regulator of G-protein signaling 19 (Rgs19), mRNA.
scl057321.4_17-S	32.4064	2.73	Terf2ip	NM_020584.1	Mus musculus telomeric repeat binding factor 2, interacting protein (Terf2ip), mRNA.
scl52455.6.1_68-S	32.4008	1.27	Cpn1	NM_030703	Mus musculus carboxypeptidase N, polypeptide 1 (Cpn1), mRNA.
scl0017222.2_194-S	32.3918	1.53	Anapc1	NM_008569.1	Mus musculus anaphase promoting complex subunit 1 (Anapc1), mRNA.
scl43413.30_169-S	32.3829	1.33	Apob	XM_137955.2	Mus musculus apolipoprotein B (Apob), mRNA.
GI_28476905-S	32.372	1.42	3010033P07Rik	XM_133123.4	Mus musculus RIKEN cDNA 3010033P07 gene (3010033P07Rik), mRNA.
scl34581.9.1_48-S	32.3475	1.52	Gpsn2	NM_134118.1	Mus musculus glycoprotein, synaptic 2 (Gpsn2), mRNA.
scl0017837.2_182-S	32.3147	6.43	Mug2	NM_008646.1	Mus musculus murinoglobulin 2 (Mug2), mRNA.
scl50308.28_413-S	32.2935	8.43	Map3k4	NM_011948.1	Mus musculus mitogen activated protein kinase kinase kinase 4 (Map3k4), mRNA.
scl0012747.1_67-S	32.2296	3.08	Clk1	NM_009905.1	
scl016974.1_230-S	32.221	2.59	Lrp6	NM_008514.1	Mus musculus low density lipoprotein receptor-related protein 6 (Lrp6), mRNA.
scl074410.2_34-S	32.2146	1.95	4932702F08Rik	XM_130221.2	Mus musculus RIKEN cDNA 4932702F08 gene (4932702F08Rik), mRNA.

scl066480.2_3-S	32.1934	1.27	Rpl15	NM_025586.1	Mus musculus ribosomal protein L15 (Rpl15), mRNA.
scl53392.18_161-S	32.1796	1.68	Mta2	NM_011842.2	Mus musculus metastais-associated gene family, member 2 (Mta2), mRNA.
scl50824.6.23_33-S	32.1711	1.77	H2-Eb1	NM_010382.1	Mus musculus histocompatibility 2, class II antigen E beta (H2-Eb1), mRNA.
scl28480.7_231-S	32.1605	1.46	Adipor2	NM_197985.2	
scl0014339.2_163-S	32.1477	9.9	Fts	NM_010241.1	Mus musculus retinoblastoma-like 2 (Rbl2), mRNA.
scl40050.12_410-S	32.1282	1.32	Ndel1	NM_023668.1	Mus musculus nuclear distribution gene E-like homolog 1 (A. nidulans) (Ndel1), mRNA.
scl0058909.2_289-S	32.0829	7.71	D430015B01Rik	NM_153574.1	Mus musculus RIKEN cDNA D430015B01 gene (D430015B01Rik), mRNA.
scl0002625.1_41-S	32.0308	2.08	2210012G02Rik	NM_025617.1	Mus musculus RIKEN cDNA 2210012G02 gene (2210012G02Rik), mRNA.
scl0070834.1_254-S	32.0237	2.86	Spag9	NM_027569.1	Mus musculus sperm associated antigen 9 (Spag9), mRNA.
scl22128.3_229-S	32.0061	2.31	Siah2	NM_009174.2	Mus musculus seven in absentia 2 (Siah2), mRNA.
scl0003304.1_37-S	31.9961	2.72	Xrn2	NM_011917	Mus musculus 5-3 exoribonuclease 2 (Xrn2), mRNA.
scl39223.3.102_51-S	31.9035	1.32	Dcxr	NM_026428.1	Mus musculus dicarbonyl L-xylulose reductase (Dcxr), mRNA.
scl0020928.2_272-S	31.8843	12.64	Abcc9	NM_011511.1	Mus musculus ATP-binding cassette, sub-family C (CFTR/MRP), member 9 (Abcc9), transcript variant 1, mRNA.
scl32844.9.1_17-S	31.8497	1.46	9430029K10Rik	XM_284281.2	Mus musculus RIKEN cDNA 9430029K10 gene (9430029K10Rik), mRNA.
scl29898.5_140-S	31.8431	1.59	Vps24	NM_025783.2	Mus musculus vacuolar protein sorting 24 (yeast) (Vps24), mRNA.
scl19403.42.1_262-S	31.8341	1.56	Hc	NM_010406.1	Mus musculus hemolytic complement (Hc), mRNA.
scl32203.4.1_10-S	31.7815	11.56	ICRFP703B1614Q5.	NM_020616.1	Mus musculus predicted gene ICRFP703B1614Q5.6 (ICRFP703B1614Q5.6), mRNA.
scl019122.2_199-S	31.775	1.43	Prnp	NM_011170.1	Mus musculus prion protein (Prnp), mRNA.
scl34795.4.1_9-S	31.7653	1.53	Sap30	NM_021788.1	Mus musculus sin3 associated polypeptide (Sap30), mRNA.
scl0234267.8_19-S	31.7623	2.97	Gpm6a	NM_153581.2	Mus musculus glycoprotein m6a (Gpm6a), mRNA.
scl38030.6.1_17-S	31.7551	2.3	2410016F19Rik	NM_026113.2	Mus musculus RIKEN cDNA 2410016F19 gene (2410016F19Rik), mRNA.
scl067374.11_26-S	31.7175	9.11	Jam2	NM_023844.2	Mus musculus junction adhesion molecule 2 (Jam2), mRNA.
scl013423.1_7-S	31.6953	2.16	Dnase2a	NM_010062	Mus musculus deoxyribonuclease II alpha (Dnase2a), mRNA.
scl27052.5.1_64-S	31.6902	1.5	Nudt1	NM_008637.1	Mus musculus nudix (nucleoside diphosphate linked moiety X)-type motif 1 (Nudt1), mRNA.
scl073327.1_204-S	31.6833	2.48	1700040I03Rik	XM_132652.1	Mus musculus RIKEN cDNA 1700040I03 gene (1700040I03Rik), mRNA.
scl18985.4_291-S	31.6259	1.37	Slc35c1	NM_145832.2	
scl0071766.1_324-S	31.6217	12.18	1300006N24Rik	NM_027911.2	Mus musculus RIKEN cDNA 1300006N24 gene (1300006N24Rik), mRNA.
scl00110323.1_173-S	31.5825	1.32	Cox6b	NM_025628.1	Mus musculus cytochrome c oxidase, subunit VIb (Cox6b), mRNA.
scl26367.12.1_1-S	31.5769	1.38	Scarb2	NM_007644.2	Mus musculus scavenger receptor class B, member 2 (Scarb2), mRNA.
scl0268449.4_2-S	31.4922	1.31	Rpl23a	NM_207523	
scl38242.7.1_214-S	31.4915	2.69	Mtrf1l	NM_175374.2	
scl0070579.1_22-S	31.4664	3.49	5730454B08Rik	NM_144530.3	Mus musculus RIKEN cDNA 5730454B08 gene (5730454B08Rik), mRNA.
scl0066125.2_261-S	31.4276	1.35	Sf3b5	NM_175102.2	
scl019821.1_18-S	31.349	8.58	Rnf2	NM_011277.1	Mus musculus ring finger protein 2 (Rnf2), mRNA.
scl9369.1.1_297-S	31.2447	2.37	Olfr713	NM_147034.1	Mus musculus olfactory receptor 713 (Olfr713), mRNA.
scl49271.23.1_11-S	31.2384	8.9	Opa1	NM_133752	Mus musculus optic atrophy 1 homolog (human) (Opa1), mRNA.
scl00242521.1_200-S	31.2122	1.95	C530050O22Rik	NM_172871.1	Mus musculus RIKEN cDNA C530050O22 gene (C530050O22Rik), mRNA.
scl012370.8_14-S	31.2095	1.52	Casp8	NM_009812.1	Mus musculus caspase 8 (Casp8), mRNA.
scl25953.10_428-S	31.2015	2.04	Wbscr16	NM_033572.1	Mus musculus Williams-Beuren syndrome chromosome region 16 homolog (human) (Wbscr16), mRNA.
scl0014082.2_318-S	31.1864	1.54	Fadd	NM_010175.2	Mus musculus Fas (TNFRSF6)-associated via death domain (Fadd), mRNA.
scl18441.3.11_50-S	31.1806	1.32	Scand1	NM_020255.1	Mus musculus SCAN domain-containing 1 (Scand1), mRNA.
scl066094.3_4-S	31.1685	1.63	Lsm7	NM_025349.1	Mus musculus LSM7 homolog, U6 small nuclear RNA associated (S. cerevisiae) (Lsm7), mRNA.
scl0002868.1_92-S	31.1154	1.27	MAp19	XM_358353.1	
scl066522.1_109-S	31.0544	1.53	Pgpep1	NM_023217.2	
scl52445.7_23-S	31.0478	2.62	Scd1	NM_009127.2	Mus musculus stearoyl-Coenzyme A desaturase 1 (Scd1), mRNA.
scl0228019.1_0-S	31.0173	2.21	BC004636	NM_145524.1	Mus musculus cDNA sequence BC004636 (BC004636), mRNA.
scl0002324.1_12-S	31.0156	2.16	Synj2bp	NM_025292.2	Mus musculus synaptojanin 2 binding protein (Synj2bp), mRNA.
scl054644.12_28-S	31.0138	1.66	DXImx46e	NM_138604.2	Mus musculus DNA segment, Chr X, Immunex 46, expressed (DXImx46e), mRNA.
scl068092.4_14-S	30.9807	2.24	Ncbp2	NM_026554.2	Mus musculus nuclear cap binding protein subunit 2 (Ncbp2), mRNA.
scl28701.8.1_115-S	30.9724	1.77	0710001P09Rik	NM_025351.2	Mus musculus RIKEN cDNA 0710001P09 gene (0710001P09Rik), mRNA.
scl0017938.1_31-S	30.9443	1.71	Naca	NM_013608.2	Mus musculus nascent polypeptide-associated complex alpha polypeptide (Naca), mRNA.
scl067680.5_8-S	30.8495	1.9	Sdhb	NM_023374.3	Mus musculus succinate dehydrogenase complex, subunit B, iron sulfur (Ip) (Sdhb), mRNA.
scl36350.20_250-S	30.8486	2.46	Xylb	XM_135223.2	
scl20428.3.1_133-S	30.8197	1.36	2010323F13Rik	NM_177157.2	
scl018226.2_9-S	30.7901	2.47	Nup62	NM_053074.1	Mus musculus nucleoporin 62 (Nup62), mRNA.
scl067956.13_1-S	30.7431	2.98	2410195B05Rik	NM_030241.2	Mus musculus RIKEN cDNA 2410195B05 gene (2410195B05Rik), mRNA.

scl0075292.1_77-S	30.7089	1.94	Prkcn	NM_029239.2	Mus musculus protein kinase C, nu (Prkcn), mRNA.
scl23193.10.1_8-S	30.6973	1.27	Alg5	NM_025442.1	Mus musculus asparagine-linked glycosylation 5 homolog (yeast, dolichyl-phosphate beta-glucosyltransferase) (Alg5), mRNA.
scl0170718.1_224-S	30.6415	1.78	Idh3b	NM_130884.1	Mus musculus isocitrate dehydrogenase 3 (NAD+) beta (Idh3b), mRNA.
scl000950.1_13-S	30.6273	1.36	Cyp27a1	NM_024264.3	Mus musculus cytochrome P450, family 27, subfamily a, polypeptide 1 (Cyp27a1), mRNA.
scl0056490.1_199-S	30.5748	1.65	Zfp288	XM_147215.1	
scl35222.4_425-S	30.5333	3.83	Myd88	NM_010851	Mus musculus myeloid differentiation primary response gene 88 (Myd88), mRNA.
scl016000.2_30-S	30.4979	1.26	Igf1	NM_010512.2	Mus musculus insulin-like growth factor 1 (Igf1), mRNA.
scl024135.1_208-S	30.4126	2.41	Zfp68	NM_013844.1	Mus musculus zinc finger protein 68 (Zfp68), mRNA.
scl0099311.1_306-S	30.3971	1.54	Commd7	NM_133850.1	Mus musculus COMM domain containing 7 (Commd7), mRNA.
scl26912.28.1_161-S	30.3848	1.4	Abcb4	NM_008830.1	Mus musculus ATP-binding cassette, sub-family B (MDR/TAP), member 4 (Abcb4), mRNA.
scl22977.3.91_193-S	30.379	1.44	Dpm3	XM_130951.1	Mus musculus dolichyl-phosphate mannosyltransferase polypeptide 3 (Dpm3), mRNA.
scl0021969.2_45-S	30.3397	2.97	Top1	NM_009408.1	Mus musculus topoisomerase (DNA) I (Top1), mRNA.
scl0059308.2_162-S	30.3284	1.77	Emcn	NM_016885.1	Mus musculus endomucin (Emcn), mRNA.
scl26859.18.1_18-S	30.3037	3.73	Ptpn12	NM_011203.2	Mus musculus protein tyrosine phosphatase, non-receptor type 12 (Ptpn12), mRNA.
scl000226.1_3-S	30.2994	1.68	Mrpl48	NM_026971.2	Mus musculus mitochondrial ribosomal protein L48 (Mrpl48), transcript variant 2, mRNA.
scl39638.4.1_92-S	30.284	1.34	Rpl23	NM_022891.1	Mus musculus ribosomal protein L23 (Rpl23), mRNA.
scl37892.17.17_22-S	30.2046	1.35	Ddx50	NM_053183.1	Mus musculus DEAD (Asp-Glu-Ala-Asp) box polypeptide 50 (Ddx50), mRNA.
scl28496.23.1_78-S	30.1985	2.16	Bms1l	NM_194339.1	Mus musculus BMS1-like, ribosome assembly protein (yeast) (Bms1l), mRNA.
scl00319192.1_1-S	30.1872	1.73	Hist2h2aa2	NM_178212	Mus musculus histone 2, H2aa2 (Hist2h2aa2), mRNA.
scl0003620.1_13-S	30.1543	2.37	Slc17a3	NM_134069.2	Mus musculus solute carrier family 17 (sodium phosphate), member 3 (Slc17a3), mRNA.
scl00235574.1_8-S	30.0647	3.87	Atp2c1	NM_175025.2	Mus musculus ATPase, Ca++-sequestering (Atp2c1), mRNA.
scl38688.8.1_12-S	29.9476	1.54	Ndufs7	NM_029272.1	Mus musculus NADH dehydrogenase (ubiquinone) Fe-S protein 7 (Ndufs7), mRNA.
scl38511.3.1_54-S	29.9473	2.65	Dcn	NM_007833.1	Mus musculus decorin (Dcn), mRNA.
scl33425.15_229-S	29.9326	1.28	D230025D16Rik	NM_145604.1	
scl36607.9.1_1-S	29.9296	8.38	Pcolce2	NM_029620.1	Mus musculus procollagen C-endopeptidase enhancer 2 (Pcolce2), mRNA.
scl023880.13_4-S	29.9003	4.14	Fyb	NM_011815.1	Mus musculus FYN binding protein (Fyb), mRNA.
scl0019299.2_231-S	29.8894	1.44	Abcd3	NM_008991.1	Mus musculus ATP-binding cassette, sub-family D (ALD), member 3 (Abcd3), mRNA.
scl0001612.1_9-S	29.8604	1.9	Angptl4	NM_020581	Mus musculus angiopoietin-like 4 (Angptl4), mRNA.
scl00230596.1_294-S	29.7776	9.78	2410002M20Rik	NM_172697.1	Mus musculus RIKEN cDNA 2410002M20 gene (2410002M20Rik), mRNA.
scl073736.7_23-S	29.694	1.3	1110008B24Rik	XM_177016.2	Mus musculus RIKEN cDNA 1110008B24 gene (1110008B24Rik), mRNA.
scl41798.24_462-S	29.6896	1.31	Vps54	NM_139061.2	Mus musculus vacuolar protein sorting 54 (yeast) (Vps54), mRNA.
scl28242.17.1_49-S	29.6866	10.24	Gys2	NM_145572.1	Mus musculus glycogen synthase 2 (Gys2), mRNA.
scl0072020.1_140-S	29.6854	1.73	1600021C16Rik	NM_028059.1	Mus musculus RIKEN cDNA 1600021C16 gene (1600021C16Rik), mRNA.
scl22501.2_29-S	29.6636	1.71	39340	NM_053102	Mus musculus selenoprotein (Sep15), mRNA.
scl30672.9.1_29-S	29.633	1.4	Ppp4c	NM_019674.2	Mus musculus protein phosphatase 4, catalytic subunit (Ppp4c), mRNA.
scl22226.9.1_81-S	29.5599	1.42	Nudt6	NM_153561.1	Mus musculus nudix (nucleoside diphosphate linked moiety X)-type motif 6 (Nudt6), mRNA.
scl0067239.2_94-S	29.5394	7.47	Bxdc1	NM_023323.2	Mus musculus brix domain containing 1 (Bxdc1), mRNA.
scl0026554.2_150-S	29.5195	1.27	Cul3	NM_016716.2	Mus musculus cullin 3 (Cul3), mRNA.
scl0003077.1_12-S	29.502	7.47	Raly	NM_023130.1	Mus musculus hnRNP-associated with lethal yellow (Raly), mRNA.
scl012367.6_5-S	29.4802	2.2	Casp3	NM_009810.1	Mus musculus caspase 3, apoptosis related cysteine protease (Casp3), mRNA.
scl0003604.1_30-S	29.4768	1.38	Etfa	NM_145615.2	Mus musculus electron transferring flavoprotein, alpha polypeptide (Etfa), nuclear gene encoding mitochondrial protein, mRNA.
scl22846.10_380-S	29.4714	2.15	Fmo5	NM_010232.3	Mus musculus flavin containing monooxygenase 5 (Fmo5), mRNA.
scl0065257.2_56-S	29.4593	1.79	Asb3	NM_023906.2	Mus musculus ankyrin repeat and SOCS box-containing protein 3 (Asb3), mRNA.
scl0002198.1_20-S	29.4001	1.44	Acaa2	NM_177470.2	Mus musculus acetyl-Coenzyme A acyltransferase 2 (mitochondrial 3-oxoacyl-Coenzyme A thiolase) (Acaa2), mRNA.
scl020610.4_145-S	29.373	1.56	Sumo3	NM_019929	
scl066310.2_4-S	29.3146	1.36	2810410M20Rik	NM_024428	Mus musculus RIKEN cDNA 2810410M20 gene (2810410M20Rik), mRNA.
scl38368.11_94-S	29.2995	6.83	Wif1	NM_011915.1	Mus musculus Wnt inhibitory factor 1 (Wif1), mRNA.
scl43736.6_95-S	29.2553	2.2	Nr2f1	NM_010151.1	Mus musculus nuclear receptor subfamily 2, group F, member 1 (Nr2f1), mRNA.
scl34490.14.1_30-S	29.2211	1.79	Ces3	NM_053200.1	Mus musculus carboxylesterase 3 (Ces3), mRNA.
scl026425.12_5-S	29.1885	1.28	Nubp1	NM_011955.1	Mus musculus nucleotide binding protein 1 (Nubp1), mRNA.
scl0001433.1_54-S	29.1749	1.62	Psmc5	NM_008950.1	Mus musculus protease (prosome, macropain) 26S subunit, ATPase 5 (Psmc5), mRNA.
scl33976.4_321-S	29.1741	1.57	2410018G23Rik	NM_027194.1	Mus musculus RIKEN cDNA 2410018G23 gene (2410018G23Rik), mRNA.
scl32211.7.1_6-S	29.1416	1.27	Eif3s5	NM_025344	Mus musculus eukaryotic translation initiation factor 3, subunit 5 (epsilon) (Eif3s5), mRNA.
scl25917.6_37-S	29.1281	1.32	Ywhag	NM_018871.2	Mus musculus 3-monooxgenase/tryptophan 5-monooxgenase activation protein, gamma polypeptide (Ywhag), mRNA.
scl019179.9_9-S	29.1176	1.67	Psmc1	NM_008947	Mus musculus protease (prosome, macropain) 26S subunit, ATPase 1 (Psmc1), mRNA.
scl40755.5.1_55-S	29.1173	1.93	D11Wsu47e	NM_177777.3	Mus musculus DNA segment, Chr 11, Wayne State University 47, expressed (D11Wsu47e), mRNA.

scl52208.4.1_8-S	29.1076	2.29	Rnf125	NM_026301.1	Mus musculus ring finger protein 125 (Rnf125), mRNA.
scl0002467.1_77-S	29.0862	1.81	Bzrp	NM_009775.2	Mus musculus benzodiazepine receptor, peripheral (Bzrp), mRNA.
scl38548.13.242_0-S	29.0557	4.09	Lta4h	NM_008517.1	Mus musculus leukotriene A4 hydrolase (Lta4h), mRNA.
scl026441.8_13-S	29.055	1.29	Psma4	NM_011966	Mus musculus proteasome (prosome, macropain) subunit, alpha type 4 (Psma4), mRNA.
scl054610.1_1-S	29.0544	24.27	Tbc1d8	NM_018775.1	Mus musculus TBC1 domain family, member 8 (Tbc1d8), mRNA.
scl52843.9.1_76-S	29.0147	3.14	Efemp2	NM_021474.2	Mus musculus epidermal growth factor-containing fibulin-like extracellular matrix protein 2 (Efemp2), mRNA.
scl077044.6_17-S	29.0066	1.54	Arid2	NM_175251	Mus musculus AT rich interactive domain 2 (Arid-rfx like) (Arid2), mRNA.
scl076073.9_113-S	28.9837	1.46	0610009F02Rik	NM_029508.1	
scl40883.5_194-S	28.9573	1.96	G6pc	NM_008061.2	Mus musculus glucose-6-phosphatase, catalytic (G6pc), mRNA.
scl0225912.8_241-S	28.9215	1.46	BC065078	NM_201351.1	Mus musculus cDNA sequence BC065078 (BC065078), mRNA.
scl0319160.1_25-S	28.9112	3.35	Hist1h4k	NM_178211.1	Mus musculus histone 1, H4k (Hist1h4k), mRNA.
scl40890.2_6-S	28.8681	1.75	Ramp2	NM_019444.1	Mus musculus receptor (calcitonin) activity modifying protein 2 (Ramp2), mRNA.
scl056448.1_20-S	28.8648	1.36	Cyp2d22	NM_019823.2	Mus musculus cytochrome P450, family 2, subfamily d, polypeptide 22 (Cyp2d22), mRNA.
scl0319187.1_0-S	28.8199	1.56	Hist1h2bn	NM_178201.1	Mus musculus histone 1, H2bn (Hist1h2bn), mRNA.
scl44183.10.1_0-S	28.8015	1.42	Aldh5a1	NM_172532.1	
scl0003640.1_4-S	28.7423	1.69	Ptcd2	XM_127497.2	
scl056397.1_158-S	28.7422	1.49	Morf4l2	NM_019768.2	Mus musculus mortality factor 4 like 2 (Morf4l2), mRNA.
scl27165.9_379-S	28.7269	1.69	Rabgef1	NM_019983.2	Mus musculus RAB guanine nucleotide exchange factor (GEF) 1 (Rabgef1), mRNA.
scl0003552.1_55-S	28.7024	7.47	Syncrip	NM_019796.2	Mus musculus synaptotagmin binding, cytoplasmic RNA interacting protein (Syncrip), mRNA.
scl0209200.1_109-S	28.6884	1.42	BC023741	XM_148582.3	Mus musculus cDNA sequence BC023741 (BC023741), mRNA.
scl000210.1_44-S	28.6521	5.41	Eif4g2	NM_013507.2	Mus musculus eukaryotic translation initiation factor 4, gamma 2 (Eif4g2), mRNA.
scl30948.10_348-S	28.6286	1.69	AI849286	XM_194372.3	Mus musculus expressed sequence AI849286 (AI849286), mRNA.
scl00227743.1_265-S	28.5615	1.3	Mapkap1	NM_177345.2	Mus musculus mitogen-activated protein kinase associated protein 1 (Mapkap1), mRNA.
scl00109113.1_250-S	28.5486	1.46	Uhrf2	NM_144873.1	Mus musculus ubiquitin-like, containing PHD and RING finger domains 2 (Uhrf2), mRNA.
scl015441.12_277-S	28.5331	1.28	Hp1bp3	NM_010470.1	Mus musculus heterochromatin protein 1, binding protein 3 (Hp1bp3), mRNA.
scl0076740.1_108-S	28.5288	1.47	C920006C10Rik	NM_133766.1	Mus musculus RIKEN cDNA C920006C10 gene (C920006C10Rik), mRNA.
scl37776.6_58-S	28.5229	1.28	D10Jhu81e	NM_138601.1	Mus musculus DNA segment, Chr 10, Johns Hopkins University 81 expressed (D10Jhu81e), mRNA.
scl0110279.7_217-S	28.499	2.91	Bcr	XM_125706.5	Mus musculus breakpoint cluster region homolog (Bcr), mRNA.
scl52628.17.1_63-S	28.472	1.82	BC018472	NM_146097.1	Mus musculus cDNA sequence BC018472 (BC018472), mRNA.
scl26392.12.1_51-S	28.4465	11.4	Rassf6	XM_132163.4	Mus musculus Ras association (RalGDS/AF-6) domain family 6 (Rassf6), mRNA.
scl069276.9_236-S	28.422	6.89	Tloc1	NM_027016.1	Mus musculus translocation protein 1 (Tloc1), mRNA.
scl0070396.1_30-S	28.4158	9.59	2210409M21Rik	NM_133728.2	Mus musculus RIKEN cDNA 2210409M21 gene (2210409M21Rik), mRNA.
scl40333.9_365-S	28.4069	2.92	Mat2b	NM_134017.1	Mus musculus methionine adenosyltransferase II, beta (Mat2b), mRNA.
scl0003709.1_47-S	28.3808	1.88	Tbc1d7	NM_025935.1	Mus musculus TBC1 domain family, member 7 (Tbc1d7), mRNA.
scl00105935.2_195-S	28.3703	4.06	AI987712	NM_178921.2	Mus musculus expressed sequence AI987712 (AI987712), mRNA.
scl075758.2_4-S	28.3123	8.64	9130401M01Rik	NM_029418.2	Mus musculus RIKEN cDNA 9130401M01 gene (9130401M01Rik), mRNA.
scl0026932.2_213-S	28.2669	1.61	Ppp2r5e	NM_012024.1	Mus musculus protein phosphatase 2, regulatory subunit B (B56), epsilon isoform (Ppp2r5e), mRNA.
scl44789.4.1_0-S	28.2207	12.77	Omd	NM_012050.1	Mus musculus osteomodulin (Omd), mRNA.
scl000078.1_211-S	28.2197	2.96	Epn2	NM_010148.1	Mus musculus epsin 2 (Epn2), mRNA.
scl22994.6_49-S	28.2159	1.26	Ssr2	NM_025448.2	Mus musculus signal sequence receptor, beta (Ssr2), mRNA.
scl0050908.1_128-S	28.1894	1.75	C1s	NM_144938.1	Mus musculus complement component 1, s subcomponent (C1s), mRNA.
scl16401.2_136-S	28.1403	1.86	9330132E09Rik	XM_283635.2	Mus musculus RIKEN cDNA 9330132E09 gene (9330132E09Rik), mRNA.
scl0108927.4_201-S	28.1302	2.61	2810489O06Rik	NM_175386.3	Mus musculus RIKEN cDNA 2810489O06 gene (2810489O06Rik), mRNA.
scl0003826.1_18-S	28.1219	2.23	Ilvbl	NM_173751.3	Mus musculus ilvB (bacterial acetolactate synthase)-like (Ilvbl), mRNA.
scl25892.5.96_2-S	28.0849	2.36	Ap1s1	NM_007457.1	Mus musculus adaptor protein complex AP-1, sigma 1 (Ap1s1), mRNA.
scl15913.2.1_30-S	28.0491	5.38	Apcs	NM_011318.1	Mus musculus serum amyloid P-component (Apcs), mRNA.
scl20333.19.1_3-S	28.0409	1.39	Usp8	NM_019729.2	Mus musculus ubiquitin specific protease 8 (Usp8), mRNA.
scl43387.27.1_23-S	28.0254	3.75	Smc6l1	NM_025695.2	Mus musculus SMC6 structural maintenance of chromosomes 6-like 1 (yeast) (Smc6l1), mRNA.
scl34868.15.1_160-S	28	1.62	F11	NM_028066.1	Mus musculus coagulation factor XI (F11), mRNA.
scl0019043.2_303-S	27.9896	1.25	Ppm1b	NM_011151.1	Mus musculus protein phosphatase 1B, magnesium dependent, beta isoform (Ppm1b), mRNA.
scl51764.24.1_222-S	27.9753	1.39	4933427L07Rik	NM_027727.1	Mus musculus RIKEN cDNA 4933427L07 gene (4933427L07Rik), mRNA.
scl22366.7.1_2-S	27.9344	3.49	Cyp7b1	NM_007825.1	Mus musculus cytochrome P450, family 7, subfamily b, polypeptide 1 (Cyp7b1), mRNA.
scl47751.3.4_1-S	27.9065	1.41	1810060D16Rik	XM_128110.3	Mus musculus RIKEN cDNA 1810060D16 gene (1810060D16Rik), mRNA.
scl069612.1_312-S	27.9051	8.56	2310037I24Rik	NM_133714.2	Mus musculus RIKEN cDNA 2310037I24 gene (2310037I24Rik), mRNA.
scl00192195.1_183-S	27.9036	1.65	Ash1l	NM_138679.2	Mus musculus ash1 (absent, small, or homeotic)-like (Drosophila) (Ash1l), mRNA.
scl35191.1.7_235-S	27.8995	1.92	Cyp8b1	NM_010012.2	Mus musculus cytochrome P450, family 8, subfamily b, polypeptide 1 (Cyp8b1), mRNA.

scl012406.1_153-S	27.8994	1.96	Serpinh1	NM_009825	Mus musculus serine (or cysteine) proteinase inhibitor, clade H, member 1 (Serpinh1), mRNA.
scl00212307.2_257-S	27.7877	1.71	Mapre2	NM_153058.2	Mus musculus microtubule-associated protein, RP/EB family, member 2 (Mapre2), mRNA.
scl013085.8_30-S	27.7871	1.87	Cyp2a12	NM_133657	Mus musculus cytochrome P450, family 2, subfamily a, polypeptide 12 (Cyp2a12), mRNA.
scl0019088.2_125-S	27.7622	2.98	Prkar2b	NM_011158.2	Mus musculus protein kinase, cAMP dependent regulatory, type II beta (Prkar2b), mRNA.
scl00394433.1_10-S	27.737	1.54	Ugt1a2	NM_013701.1	
scl076784.15_0-S	27.7242	5.43	Mtif2	NM_133767.1	Mus musculus mitochondrial translational initiation factor 2 (Mtif2), mRNA.
scl36159.7.1_126-S	27.7211	1.66	Angptl6	NM_145154	Mus musculus angiopoietin-like 6 (Angptl6), mRNA.
scl29389.16_66-S	27.7183	1.3	Slco1b2	NM_178235.1	Mus musculus solute carrier organic anion transporter family, member 1b2 (Slco1b2), transcript variant 1, mRNA.
scl00319263.2_23-S	27.6443	1.59	A030012M09Rik	NM_183028.1	Mus musculus RIKEN cDNA A030012M09 gene (A030012M09Rik), mRNA.
scl25801.8_88-S	27.6337	1.58	Rac1	NM_009007.1	Mus musculus RAS-related C3 botulinum substrate 1 (Rac1), mRNA.
scl30177.14.1_175-S	27.6278	4.66	Tbxas1	NM_011539.2	Mus musculus thromboxane A synthase 1, platelet (Tbxas1), mRNA.
scl0026895.1_34-S	27.6212	1.66	Cops7b	NM_172974.1	Mus musculus COP9 (constitutive photomorphogenic) homolog, subunit 7b (Arabidopsis thaliana) (Cops7b), mRNA.
scl34841.3_211-S	27.5936	2.84	4921511I16Rik	NM_172407.1	Mus musculus RIKEN cDNA 4921511I16 gene (4921511I16Rik), mRNA.
scl00232341.1_311-S	27.5774	1.36	Prkwnk1	NM_198703.1	Mus musculus protein kinase, lysine deficient 1 (Prkwnk1), mRNA.
scl18812.14.127_2-S	27.5539	1.7	1200015F23Rik	XM_203936.3	Mus musculus RIKEN cDNA 1200015F23 gene (1200015F23Rik), mRNA.
scl083962.1_77-S	27.5482	1.54	Btbd1	NM_146193	Mus musculus BTB (POZ) domain containing 1 (Btbd1), mRNA.
scl0002682.1_0-S	27.5468	9.84	Dio1	NM_007860	Mus musculus deiodinase, iodothyronine, type I (Dio1), mRNA.
scl47811.2_529-S	27.534	1.47	2610029D06Rik	NM_030199.2	
scl0050927.2_48-S	27.5154	1.74	Nasp	NM_016777.2	Mus musculus nuclear autoantigenic sperm protein (histone-binding) (Nasp), mRNA.
scl35434.19.159_3-S	27.489	1.8	AW107703	NM_198307.1	Mus musculus expressed sequence AW107703 (AW107703), mRNA.
scl19523.39_64-S	27.4827	2.4	Notch1	NM_008714.2	Mus musculus Notch gene homolog 1 (Drosophila) (Notch1), mRNA.
scl071693.1_122-S	27.4309	1.6	Colec11	XM_283054.2	Mus musculus collectin sub-family member 11 (Colec11), mRNA.
scl0001111.1_19-S	27.4117	1.77	Mrps35	NM_145573	Mus musculus mitochondrial ribosomal protein S35 (Mrps35), mRNA.
scl0020842.1_84-S	27.3951	9.41	Stag1	NM_009282.2	Mus musculus stromal antigen 1 (Stag1), mRNA.
scl0013039.1_128-S	27.377	5.46	Ctsl	NM_009984.2	Mus musculus cathepsin L (Ctsl), mRNA.
scl0018813.1_104-S	27.341	2.51	Pa2g4	NM_011119.1	Mus musculus proliferation-associated 2G4 (Pa2g4), mRNA.
scl066108.3_20-S	27.295	1.25	Ndufa9	NM_025358.1	Mus musculus NADH dehydrogenase (ubiquinone) 1 alpha subcomplex, 9 (Ndufa9), mRNA.
scl17924.1_368-S	27.2869	2.73	Fzd7	NM_008057.2	Mus musculus frizzled homolog 7 (Drosophila) (Fzd7), mRNA.
scl35643.12.1_48-S	27.2657	8.22	Lipc	NM_008280.2	Mus musculus lipase, hepatic (Lipc), mRNA.
scl0019301.2_74-S	27.2549	1.31	Pxmp2	NM_008993.1	Mus musculus peroxisomal membrane protein 2 (Pxmp2), mRNA.
scl31550.23.1_53-S	27.2485	2.1	2610511M17Rik	NM_028818.1	Mus musculus RIKEN cDNA 2610511M17 gene (2610511M17Rik), mRNA.
scl0015519.1_14-S	27.2259	12.15	Hspca	NM_010480	Mus musculus heat shock protein 1, alpha (Hspca), mRNA.
scl0003202.1_259-S	27.2243	1.66	Gpiap1	NM_016739.2	Mus musculus GPI-anchored membrane protein 1 (Gpiap1), mRNA.
scl0001953.1_80-S	27.1965	3.95	Smarca3	NM_009210.1	Mus musculus SWI/SNF related, matrix associated, actin dependent regulator of chromatin, subfamily a, member 3 (Smarca3), mRNA.
scl011777.6_49-S	27.145	1.61	Ap3s1	NM_009681.2	Mus musculus adaptor-related protein complex 3, sigma 1 subunit (Ap3s1), mRNA.
scl068276.1_0-S	27.1096	1.49	4930584N22Rik	NM_026654.1	Mus musculus RIKEN cDNA 4930584N22 gene (4930584N22Rik), mRNA.
scl33220.5.1_30-S	27.056	1.3	1810017G16Rik	NM_021502.1	Mus musculus RIKEN cDNA 1810017G16 gene (1810017G16Rik), mRNA.
scl45598.20_218-S	27.0522	1.49	Ndrg2	NM_013864	
scl35011.1.33_50-S	26.9773	3.25	1810011O10Rik	NM_026931.1	Mus musculus RIKEN cDNA 1810011O10 gene (1810011O10Rik), mRNA.
scl0001911.1_22-S	26.9389	1.99	Umps	NM_009471.1	Mus musculus uridine monophosphate synthetase (Umps), mRNA.
scl015381.1_4-S	26.9323	1.53	Hnrpc	NM_016884.1	Mus musculus heterogeneous nuclear ribonucleoprotein C (Hnrpc), mRNA.
scl28068.2_468-S	26.8987	7.43	Fgl2	NM_008013.2	Mus musculus fibrinogen-like protein 2 (Fgl2), mRNA.
scl39981.11_196-S	26.841	1.5	Dhx33	NM_178367.3	Mus musculus DEAH (Asp-Glu-Ala-His) box polypeptide 33 (Dhx33), mRNA.
scl0065973.1_101-S	26.7891	2.03	Asph	NM_023066.1	Mus musculus aspartate-beta-hydroxylase (Asph), mRNA.
scl0067475.1_65-S	26.7878	1.67	1300013B24Rik	NM_026184.2	Mus musculus RIKEN cDNA 1300013B24 gene (1300013B24Rik), mRNA.
scl0067456.1_141-S	26.7812	2.12	1200009B18Rik	NM_026168.1	Mus musculus RIKEN cDNA 1200009B18 gene (1200009B18Rik), mRNA.
scl41882.13_532-S	26.7549	1.26	Nipsnap1	NM_008698.1	Mus musculus 4-nitrophenylphosphatase domain and non-neuronal SNAP25-like protein homolog 1 (C. elegans) (Nipsnap1), mRNA.
scl24791.10_1-S	26.7285	1.28	Ddost	NM_007838.2	Mus musculus dolichyl-di-phosphooligosaccharide-protein glycotransferase (Ddost), mRNA.
scl018970.2_6-S	26.6676	1.88	Polb	NM_011130.1	Mus musculus polymerase (DNA directed), beta (Polb), mRNA.
scl00140629.2_137-S	26.6553	1.86	C330018L13Rik	NM_080562.2	Mus musculus RIKEN cDNA C330018L13 gene (C330018L13Rik), mRNA.
scl067105.1_68-S	26.5513	1.29	1700034H14Rik	NM_025969.1	Mus musculus RIKEN cDNA 1700034H14 gene (1700034H14Rik), mRNA.
scl53489.23_479-S	26.5091	3.02	Pacs1	XM_283545.1	Mus musculus phosphofurin acidic cluster sorting protein 1 (Pacs1), mRNA.
scl26323.5.1_40-S	26.4996	5.05	Plac8	NM_139198.1	Mus musculus placenta-specific 8 (Plac8), mRNA.
scl0102247.1_13-S	26.4344	1.39	AU041707	NM_018743.2	Mus musculus expressed sequence AU041707 (AU041707), mRNA.
scl30506.2_36-S	26.4261	1.41	Ifitm3	NM_025378.1	Mus musculus interferon induced transmembrane protein 3 (Ifitm3), mRNA.
scl067604.1_6-S	26.4178	1.34	1110007L15Rik	NM_026269.1	Mus musculus RIKEN cDNA 1110007L15 gene (1110007L15Rik), mRNA.

scl48484.12.1_8-S	26.4094	1.57	Pla1a	NM_134102	Mus musculus phospholipase A1 member A (Pla1a), mRNA.
scl0216622.2_35-S	26.3649	2.58	4931440F15Rik	NM_176829.1	Mus musculus RIKEN cDNA 4931440F15 gene (4931440F15Rik), mRNA.
scl34044.10_124-S	26.3585	1.66	Fbxo25	NM_025785.1	Mus musculus F-box only protein 25 (Fbxo25), mRNA.
scl0012763.1_321-S	26.3455	1.7	Cmah	NM_007717.1	Mus musculus cytidine monophospho-N-acetylneuraminic acid hydroxylase (Cmah), mRNA.
scl000701.1_5-S	26.3384	3.71	Appbp1	NM_144931.1	Mus musculus amyloid beta precursor protein binding protein 1 (Appbp1), mRNA.
scl0003786.1_57-S	26.1814	4.47	Prg	NM_011157.1	Mus musculus proteoglycan, secretory granule (Prg), mRNA.
scl42219.1_29-S	26.1584	1.46	0610007P14Rik	NM_021446	
scl00108735.2_193-S	26.095	1.44	2010005O13Rik	NM_145512.2	Mus musculus RIKEN cDNA 2010005O13 gene (2010005O13Rik), mRNA.
scl0002716.1_33-S	26.0571	1.22	Scp2	NM_011327.1	Mus musculus sterol carrier protein 2, liver (Scp2), mRNA.
scl00231327.1_147-S	26.0278	2.99	Ppat	NM_172146.1	Mus musculus phosphoribosyl pyrophosphate amidotransferase (Ppat), mRNA.
scl0067105.1_17-S	26.0211	3.73	1700034H14Rik	NM_025969.1	Mus musculus RIKEN cDNA 1700034H14 gene (1700034H14Rik), mRNA.
scl067704.2_8-S	26.0111	1.36	1810037I17Rik	NM_024461.1	Mus musculus RIKEN cDNA 1810037I17 gene (1810037I17Rik), mRNA.
scl0023806.2_110-S	26.0071	2.26	Arih1	NM_019927.1	Mus musculus ariadne ubiquitin-conjugating enzyme E2 binding protein homolog 1 (Drosophila) (Arih1), mRNA.
scl0214572.8_7-S	26.0042	2.32	BC006705	NM_145404.1	Mus musculus cDNA sequence BC006705 (BC006705), mRNA.
scl0013085.1_15-S	25.9998	1.76	Cyp2a12	NM_133657.1	Mus musculus cytochrome P450, family 2, subfamily a, polypeptide 12 (Cyp2a12), mRNA.
scl018693.12_2-S	25.9607	1.36	Prkcabp	NM_008837.1	Mus musculus protein kinase C, alpha binding protein (Prkcabp), mRNA.
scl0102462.1_25-S	25.9535	1.24	1190002L16Rik	NM_133976.1	Mus musculus RIKEN cDNA 1190002L16 gene (1190002L16Rik), mRNA.
scl0029809.2_171-S	25.9129	2.61	Rabgap1l	NM_013862.3	
scl20013.3_0-S	25.8736	1.82	Manbal	XM_130628.2	
scl0011852.2_145-S	25.8528	2.14	Rhob	NM_007483.2	Mus musculus ras homolog gene family, member B (Rhob), mRNA.
scl22402.6.1_12-S	25.8444	1.34	Mrps28	NM_025434.2	Mus musculus mitochondrial ribosomal protein S28 (Mrps28), mRNA.
scl0066206.2_25-S	25.7932	8.49	1110059E24Rik	NM_025423.1	Mus musculus RIKEN cDNA 1110059E24 gene (1110059E24Rik), mRNA.
scl066445.5_23-S	25.7879	1.41	Cyc1	NM_025567.1	Mus musculus cytochrome c-1 (Cyc1), mRNA.
scl45943.26_212-S	25.7832	1.85	Kpnb3	NM_023579.2	Mus musculus karyopherin (importin) beta 3 (Kpnb3), mRNA.
scl43496.27.1_1-S	25.7809	1.41	2610528A15Rik	NM_028151.1	Mus musculus RIKEN cDNA 2610528A15 gene (2610528A15Rik), mRNA.
scl23145.17_313-S	25.7226	1.24	Mbnl1	NM_020007.2	Mus musculus muscleblind-like 1 (Drosophila) (Mbnl1), mRNA.
scl23523.7.1_20-S	25.7131	1.28	Fbxo6b	NM_015797.1	Mus musculus F-box only protein 6b (Fbxo6b), mRNA.
scl23111.16.8_12-S	25.7032	1.52	Gfm	NM_138591.1	Mus musculus G elongation factor (Gfm), mRNA.
scl0014453.2_277-S	25.691	5.71	Gas2	NM_008087.1	Mus musculus growth arrest specific 2 (Gas2), mRNA.
scl31965.9_618-S	25.6526	1.27	C430003P19Rik	NM_198017.1	Mus musculus RIKEN cDNA C430003P19 gene (C430003P19Rik), mRNA.
scl34998.4_500-S	25.6402	1.65	B230378H13Rik	NM_177089.3	
scl48584.2_290-S	25.6282	1.54	1300018K11Rik	XM_148373.1	Mus musculus RIKEN cDNA 1300018K11 gene (1300018K11Rik), mRNA.
scl011536.1_57-S	25.6257	1.54	Admr	NM_007412.1	Mus musculus adrenomedullin receptor (Admr), mRNA.
scl067582.10_107-S	25.6222	1.5	Slc25a26	NM_026255.2	Mus musculus solute carrier family 25 (mitochondrial carrier; phosphate carrier), member 26 (Slc25a26), mRNA.
scl077781.2_11-S	25.6219	4.01	Epm2aip1	NM_175266.2	Mus musculus EPM2A (laforin) interacting protein 1 (Epm2aip1), mRNA.
scl0271278.1_67-S	25.5955	2.08	BC024139	NM_198172	Mus musculus cDNA sequence BC024139 (BC024139), mRNA.
scl44881.2_33-S	25.5952	2.21	5730453H04Rik	NM_175338.2	Mus musculus RIKEN cDNA 5730453H04 gene (5730453H04Rik), mRNA.
scl018105.7_5-S	25.5798	1.53	Nqo2	NM_020282.2	Mus musculus NAD(P)H dehydrogenase, quinone 2 (Nqo2), mRNA.
IGKV4-71_AJ231218_Ig_	25.5673	15.78			
scl00239318.2_162-S	25.5606	1.8	Plcxd3	NM_177355.2	
scl16460.33_2-S	25.5168	9.25	Hdlbp	NM_133808.2	Mus musculus high density lipoprotein (HDL) binding protein (Hdlbp), mRNA.
scl53421.25_339-S	25.5084	1.39	D19Ertd703e	NM_029456.1	Mus musculus DNA segment, Chr 19, ERATO Doi 703, expressed (D19Ertd703e), mRNA.
scl16174.14.1_64-S	25.5048	1.29	BC003331	NM_145511.1	Mus musculus cDNA sequence BC003331 (BC003331), mRNA.
scl0012848.2_316-S	25.4999	1.29	Cops2	NM_009939.1	Mus musculus COP9 (constitutive photomorphogenic) homolog, subunit 2 (Arabidopsis thaliana) (Cops2), mRNA.
scl25073.4.1_109-S	25.4941	1.62	Ipp	NM_008389.1	Mus musculus IAP promoted placental gene (Ipp), mRNA.
scl0017843.1_58-S	25.4741	11.65	Mup4	NM_008648.1	Mus musculus major urinary protein 4 (Mup4), mRNA.
scl019349.2_45-S	25.4563	1.24	Rab7	NM_009005.1	Mus musculus RAB7, member RAS oncogene family (Rab7), mRNA.
scl46303.7_279-S	25.427	1.24	Lrp10	NM_022993.2	Mus musculus low-density lipoprotein receptor-related protein 10 (Lrp10), mRNA.
scl34037.34.366_10-S	25.4254	2.72	Arhgef10	NM_172751.1	Mus musculus Rho guanine nucleotide exchange factor (GEF) 10 (Arhgef10), mRNA.
scl35398.12.2_77-S	25.388	1.62	Alas1	NM_020559.1	Mus musculus aminolevulinic acid synthase 1 (Alas1), mRNA.
scl22978.7.1_18-S	25.3739	1.23	Krtcap2	NM_025327.1	Mus musculus keratinocyte associated protein 2 (Krtcap2), mRNA.
scl23442.8_471-S	25.3689	1.26	1110060F11Rik	NM_026395.1	Mus musculus RIKEN cDNA 1110060F11 gene (1110060F11Rik), mRNA.
scl40322.5_155-S	25.3489	9.85	Pttg1	NM_013917.1	Mus musculus pituitary tumor-transforming 1 (Pttg1), mRNA.
scl40072.13_201-S	25.3456	1.29	Map2k4	NM_009157	Mus musculus mitogen activated protein kinase kinase 4 (Map2k4), mRNA.
scl00107146.2_299-S	25.3062	1.31	Glyat	NM_145935.2	Mus musculus glycine-N-acyltransferase (Glyat), mRNA.
scl014057.6_27-S	25.2869	1.41	Sfxn1	NM_027324.2	Mus musculus sideroflexin 1 (Sfxn1), mRNA.

scl020280.7_0-S	25.2764	1.61	Scp2	NM_011327.1	Mus musculus sterol carrier protein 2, liver (Scp2), mRNA.
scl32592.2.658_11-S	25.26	2.16	Ndn	NM_010882.2	Mus musculus necdin (Ndn), mRNA.
scl056378.6_20-S	25.255	1.28	Arpc3	NM_019824.2	Mus musculus actin related protein 2/3 complex, subunit 3 (Arpc3), mRNA.
scl0023802.2_62-S	25.2544	1.22	Amfr	NM_011787.1	Mus musculus autocrine motility factor receptor (Amfr), mRNA.
scl0018087.1_31-S	25.2264	2.1	Nktr	NM_010918.1	Mus musculus natural killer tumor recognition sequence (Nktr), mRNA.
scl099650.7_29-S	25.1874	1.34	4933434E20Rik	NM_025762.1	Mus musculus RIKEN cDNA 4933434E20 gene (4933434E20Rik), mRNA.
scl0011983.1_209-S	25.1809	1.29	Atpif1	NM_007512.2	
scl0015381.1_237-S	25.1724	1.49	Hnrpc	NM_016884.1	Mus musculus heterogeneous nuclear ribonucleoprotein C (Hnrpc), mRNA.
GI_6679936-S	25.1682	1.3	Gapd	NM_008084.1	Mus musculus glyceraldehyde-3-phosphate dehydrogenase (Gapd), mRNA.
scl41823.30_613-S	25.1484	2.06	Egfr	NM_207655.1	Mus musculus epidermal growth factor receptor (Egfr), transcript variant 1, mRNA.
scl00210146.1_172-S	25.1402	1.66	AF322649	NM_153134.2	Mus musculus cDNA sequence AF322649 (AF322649), mRNA.
scl0229279.5_20-S	25.0952	2.62	2610510D13Rik	NM_146130	
scl0066408.1_304-S	25.0798	1.91	Aptx	NM_025545.2	Mus musculus aprataxin (Aptx), mRNA.
scl0002903.1_3-S	25.0744	2.09	2700099C19Rik	NM_028303.1	Mus musculus RIKEN cDNA 2700099C19 gene (2700099C19Rik), mRNA.
scl012925.2_22-S	25.0616	2.14	Crip1	NM_007763	Mus musculus cysteine-rich protein 1 (intestinal) (Crip1), mRNA.
scl51719.2_118-S	25.0569	1.32	Zadh2	NM_146090.2	
scl50673.7.1_4-S	25.048	1.47	Mrpl2	NM_025302.2	Mus musculus mitochondrial ribosomal protein L2 (Mrpl2), mRNA.
scl26088.14.1_16-S	25.0468	1.22	Aldh2	NM_009656.1	Mus musculus aldehyde dehydrogenase 2, mitochondrial (Aldh2), mRNA.
scl0013982.1_185-S	25.0372	9.38	Esr1	NM_007956.2	Mus musculus estrogen receptor 1 (alpha) (Esr1), mRNA.
scl50860.12.324_21-S	25.0268	1.44	Cyp4f15	NM_134127.1	Mus musculus cytochrome P450, family 4, subfamily f, polypeptide 15 (Cyp4f15), mRNA.
scl28993.8_275-S	24.9742	1.37	Hibadh	NM_145567.1	Mus musculus 3-hydroxyisobutyrate dehydrogenase (Hibadh), mRNA.
scl38677.11_311-S	24.8907	1.24	Csnk1g2	NM_134002.1	Mus musculus casein kinase 1, gamma 2 (Csnk1g2), mRNA.
scl0242960.1_52-S	24.8813	4.14	Fbxl5	NM_178729.2	Mus musculus F-box and leucine-rich repeat protein 5 (Fbxl5), mRNA.
scl013209.11_23-S	24.8782	2.94	Ddx6	NM_007841.2	Mus musculus DEAD (Asp-Glu-Ala-Asp) box polypeptide 6 (Ddx6), mRNA.
scl0001304.1_8-S	24.8746	2.03	4930579A11Rik	NM_029478.2	Mus musculus RIKEN cDNA 4930579A11 gene (4930579A11Rik), mRNA.
scl0017314.1_97-S	24.8709	1.89	Mgmt	NM_008598.1	Mus musculus O-6-methylguanine-DNA methyltransferase (Mgmt), mRNA.
scl066276.2_21-S	24.8671	1.44	1810009A15Rik	NM_025463.1	Mus musculus RIKEN cDNA 1810009A15 gene (1810009A15Rik), mRNA.
scl000674.1_0-S	24.8365	1.81	Itgb1	NM_010578.1	Mus musculus integrin beta 1 (fibronectin receptor beta) (Itgb1), mRNA.
scl0015950.1_6-S	24.8335	2.15	Ifi203	NM_008328.1	Mus musculus interferon activated gene 203 (Ifi203), mRNA.
scl0074182.2_93-S	24.82	1.57	2310032D16Rik	NM_027096.1	Mus musculus RIKEN cDNA 2310032D16 gene (2310032D16Rik), mRNA.
scl070737.1_295-S	24.7991	37.35	Cgn	XM_131052.4	Mus musculus cingulin (Cgn), mRNA.
scl41201.7.1_21-S	24.7935	1.33	Vtn	NM_011707.1	Mus musculus vitronectin (Vtn), mRNA.
scl0107392.1_305-S	24.7901	1.46	Brms1	NM_134155.1	Mus musculus breast cancer metastasis-suppressor 1 (Brms1), mRNA.
scl0218236.1_149-S	24.7704	1.73	BC010304	XM_127326.3	Mus musculus cDNA sequence BC010304 (BC010304), mRNA.
scl48818.18.1_43-S	24.7494	1.35	Trap1	NM_026508.1	
scl30690.8_420-S	24.7406	3.3	Nfatc2ip	NM_010900.1	Mus musculus nuclear factor of activated T-cells, cytoplasmic, calcineurin-dependent 2 interacting protein (Nfatc2ip), mRNA.
scl37659.18.259_11-S	24.7142	1.6	Tra1	NM_011631.1	Mus musculus tumor rejection antigen gp96 (Tra1), mRNA.
scl46162.12.1_3-S	24.7082	9.47	4933425F03Rik	NM_028903.1	Mus musculus RIKEN cDNA 4933425F03 gene (4933425F03Rik), mRNA.
scl0027096.1_27-S	24.6957	2.36	Trappc3	NM_013718.2	Mus musculus trafficking protein particle complex 3 (Trappc3), mRNA.
scl0003169.1_64-S	24.6922	2.59	BC028278	XM_130696.2	
scl000779.1_49-S	24.6864	1.56	Lancl1	NM_021295.1	Mus musculus LanC (bacterial lantibiotic synthetase component C)-like 1 (Lancl1), mRNA.
scl067706.2_5-S	24.6587	1.4	1810059G22Rik	NM_026325.1	Mus musculus RIKEN cDNA 1810059G22 gene (1810059G22Rik), mRNA.
scl068180.6_62-S	24.5915	1.73	6430559E15Rik	XM_131537.4	Mus musculus RIKEN cDNA 6430559E15 gene (6430559E15Rik), mRNA.
scl26349.2.101_165-S	24.5911	4.51	Gk2	NM_010294.1	Mus musculus glycerol kinase 2 (Gk2), mRNA.
scl0003259.1_330-S	24.5819	2.31	Sh2d3c	NM_013781.2	Mus musculus SH2 domain containing 3C (Sh2d3c), mRNA.
scl39316.7.1_30-S	24.5768	1.27	Mrpl38	NM_024177.2	Mus musculus mitochondrial ribosomal protein L38 (Mrpl38), mRNA.
scl49313.9.1_27-S	24.5767	1.53	Hrg	NM_053176.1	Mus musculus histidine-rich glycoprotein (Hrg), mRNA.
scl0067480.2_9-S	24.569	2.97	1300013D05Rik	NM_026186.2	Mus musculus RIKEN cDNA 1300013D05 gene (1300013D05Rik), mRNA.
scl20330.11.1_76-S	24.5589	1.34	Blvra	NM_026678.3	Mus musculus biliverdin reductase A (Blvra), mRNA.
scl067942.2_64-S	24.5417	1.27	Atp5g2	NM_026468.1	Mus musculus ATP synthase, H+ transporting, mitochondrial F0 complex, subunit c (subunit 9), isoform 2 (Atp5g2), mRNA.
scl0066827.2_11-S	24.5209	1.52	Ttc1	NM_133795.1	Mus musculus tetratricopeptide repeat domain 1 (Ttc1), mRNA.
scl022147.3_29-S	24.5131	3	Tuba7	NM_009449.2	Mus musculus tubulin, alpha 7 (Tuba7), mRNA.
scl33705.5.1_110-S	24.5026	1.32	2010315L10Rik	NM_025917.1	Mus musculus RIKEN cDNA 2010315L10 gene (2010315L10Rik), mRNA.
scl30988.16_625-S	24.471	1.32	2700017M01Rik	NM_028292.1	Mus musculus RIKEN cDNA 2700017M01 gene (2700017M01Rik), mRNA.
scl0001327.1_163-S	24.4681	1.88	Trim41	NM_145377	Mus musculus tripartite motif-containing 41 (Trim41), mRNA.
scl00214290.1_251-S	24.4502	1.31	Zcchc6	NM_153538.1	Mus musculus zinc finger, CCHC domain containing 6 (Zcchc6), mRNA.

scl067549.1_82-S	24.4485	1.42	Gpr89	NM_026229.1	
scl0001105.1_0-S	24.44	2.28	4932409F11Rik	NM_029404.1	Mus musculus RIKEN cDNA 4932409F11 gene (4932409F11Rik), mRNA.
scl30429.2.1_68-S	24.3897	1.91	Gng11	NM_025331.2	Mus musculus guanine nucleotide binding protein (G protein), gamma 11 (Gng11), mRNA.
scl31333.17.1_37-S	24.3868	3.71	Ptpn5	NM_013643.1	Mus musculus protein tyrosine phosphatase, non-receptor type 5 (Ptpn5), mRNA.
scl0021859.1_62-S	24.3651	1.36	Timp3	NM_011595.1	Mus musculus tissue inhibitor of metalloproteinase 3 (Timp3), mRNA.
scl078121.4_47-S	24.334	2.05	9930116P15Rik	XM_127961.3	
scl071679.3_0-S	24.323	1.22	Atp5h	NM_027862	Mus musculus ATP synthase, H+ transporting, mitochondrial F0 complex, subunit d (Atp5h), mRNA.
scl52754.4_241-S	24.2928	1.44	0610038F07Rik	NM_025333.2	Mus musculus RIKEN cDNA 0610038F07 gene (0610038F07Rik), mRNA.
scl0014869.1_77-S	24.2803	2.35	Gstp1	NM_013541	Mus musculus glutathione S-transferase, pi 1 (Gstp1), mRNA.
scl0067916.1_299-S	24.2507	1.22	Ppap2b	NM_080555.1	Mus musculus phosphatidic acid phosphatase type 2B (Ppap2b), mRNA.
scl45372.3_35-S	24.2481	1.57	Mscp	NM_030054.2	Mus musculus mitochondrial solute carrier protein (Mscp), mRNA.
scl53380.11_494-S	24.2325	1.33	Fads1	NM_146094.1	Mus musculus fatty acid desaturase 1 (Fads1), mRNA.
scl0002876.1_12-S	24.232	1.39	Ard1	NM_019870.1	Mus musculus N-acetyltransferase ARD1 homolog (S. cerevisiae) (Ard1), mRNA.
scl000251.1_1-S	24.2308	2.02	Mrpl48	NM_026971.2	Mus musculus mitochondrial ribosomal protein L48 (Mrpl48), transcript variant 2, mRNA.
scl38270.7_86-S	24.2077	2.47	Dnajc14	NM_028873.2	
gi_21070949_ref_NM_01	24.2042	1.91	Ubc	XM_147315.1	Mus musculus ubiquitin C (Ubc), mRNA.
scl071664.1_311-S	24.1983	1.3	0610006F02Rik	NM_027853.1	Mus musculus RIKEN cDNA 0610006F02 gene (0610006F02Rik), mRNA.
scl48750.6_548-S	24.1935	1.24	C530044N13Rik	NM_146067	Mus musculus RIKEN cDNA C530044N13 gene (C530044N13Rik), mRNA.
scl28963.13.1_27-S	24.1769	1.33	Nt5c3	NM_026004.1	Mus musculus 5-nucleotidase, cytosolic III (Nt5c3), mRNA.
scl014961.2_4-S	24.0779	2.08	Rmcs1	NM_207105.1	Mus musculus histocompatibility 2, class II antigen A, beta 1 (H2-Ab1), mRNA.
scl29667.6_8-S	24.0714	1.6	Bhlhb2	NM_011498.2	Mus musculus basic helix-loop-helix domain containing, class B2 (Bhlhb2), mRNA.
scl17186.52.1_1-S	24.0655	3.03	Spna1	NM_011465.2	Mus musculus spectrin alpha 1 (Spna1), mRNA.
scl069038.2_4-S	24.0384	1.49	1810006K21Rik	XM_207074	Mus musculus RIKEN cDNA 1810006K21 gene (1810006K21Rik), mRNA.
scl30281.25_19-S	23.9663	1.84	4631427C17Rik	NM_021414.2	Mus musculus RIKEN cDNA 4631427C17 gene (4631427C17Rik), mRNA.
scl53217.16.138_16-S	23.9408	2.85	Uhrf2	NM_144873.1	Mus musculus ubiquitin-like, containing PHD and RING finger domains 2 (Uhrf2), mRNA.
scl078697.1_5-S	23.9293	1.98	C330017I15Rik	NM_178403.2	Mus musculus RIKEN cDNA C330017I15 gene (C330017I15Rik), mRNA.
scl48651.6_179-S	23.928	1.8	Ehhadh	NM_023737.2	Mus musculus enoyl-Coenzyme A, hydratase/3-hydroxyacyl Coenzyme A dehydrogenase (Ehhadh), mRNA.
scl49869.7.1_26-S	23.8533	2.03	Slc22a7	NM_144856.1	Mus musculus solute carrier family 22 (organic anion transporter), member 7 (Slc22a7), mRNA.
scl46638.4_82-S	23.839	1.73	Kctd6	NM_027782.1	Mus musculus potassium channel tetramerisation domain containing 6 (Kctd6), mRNA.
scl44070.4.1_3-S	23.83	3.28	Eef1e1	NM_025380.1	Mus musculus eukaryotic translation elongation factor 1 epsilon 1 (Eef1e1), mRNA.
scl0002777.1_339-S	23.8269	2.19	Ak2	NM_016895.2	Mus musculus adenylate kinase 2 (Ak2), mRNA.
scl48122.9.2_1-S	23.8149	1.64	C9	NM_013485.1	Mus musculus complement component 9 (C9), mRNA.
scl33324.19.245_0-S	23.7789	1.7	D8Wsu151e	NM_146216.2	Mus musculus DNA segment, Chr 8, Wayne State University 151, expressed (D8Wsu151e), mRNA.
scl22773.20.1_35-S	23.7218	2.83	Phtf1	XM_356222.1	Mus musculus putative homeodomain transcription factor 1 (Phtf1), mRNA.
scl52902.14.1_38-S	23.6983	2.41	Stip1	NM_016737.1	Mus musculus stress-induced phosphoprotein 1 (Stip1), mRNA.
scl24770.15_172-S	23.6971	1.33	Aldh4a1	NM_175438	Mus musculus aldehyde dehydrogenase 4 family, member A1 (Aldh4a1), mRNA.
scl071704.9_277-S	23.6832	1.63	Arhgef3	NM_027871.1	Mus musculus Rho guanine nucleotide exchange factor (GEF) 3 (Arhgef3), mRNA.
scl20329.42.1_43-S	23.6741	1.56	Ascc3l1	NM_177214.3	
scl018087.13_25-S	23.6539	5.1	Nktr	NM_010918.1	Mus musculus natural killer tumor recognition sequence (Nktr), mRNA.
scl072747.10_28-S	23.6531	2.4	2810439F02Rik	NM_028341.1	Mus musculus RIKEN cDNA 2810439F02 gene (2810439F02Rik), mRNA.
scl0002929.1_32-S	23.6479	1.66	2610029G23Rik	NM_026312.2	Mus musculus RIKEN cDNA 2610029G23 gene (2610029G23Rik), mRNA.
scl35702.23.1_49-S	23.6438	1.54	AV340375	NM_172519.1	Mus musculus expressed sequence AV340375 (AV340375), mRNA.
scl46929.4_12-S	23.6182	1.24	Phf5a	NM_026737.1	Mus musculus PHD finger protein 5A (Phf5a), mRNA.
scl0227800.12_249-S	23.5356	1.26	Rabgap1	NM_146121.1	Mus musculus RAB GTPase activating protein 1 (Rabgap1), mRNA.
scl38692.8.1_47-S	23.5309	1.42	Atp5d	NM_025313.1	Mus musculus ATP synthase, H+ transporting, mitochondrial F1 complex, delta subunit (Atp5d), mRNA.
scl0004181.1_42-S	23.5282	1.53	Vps29	NM_019780.1	Mus musculus vacuolar protein sorting 29 (S. pombe) (Vps29), mRNA.
scl32817.6.1_8-S	23.5196	3.35	Tyrobp	NM_011662.2	Mus musculus TYRO protein tyrosine kinase binding protein (Tyrobp), mRNA.
scl19300.3.1_12-S	23.5165	1.52	2010311D03Rik	NM_133839.1	Mus musculus RIKEN cDNA 2010311D03 gene (2010311D03Rik), mRNA.
scl53101.26.1_9-S	23.5038	1.52	Abcc2	NM_013806.1	Mus musculus ATP-binding cassette, sub-family C (CFTR/MRP), member 2 (Abcc2), mRNA.
scl22849.16.1_85-S	23.4869	1.3	Pdzk1	NM_021517	Mus musculus PDZ domain containing 1 (Pdzk1), mRNA.
scl00244202.2_30-S	23.4681	4.57	Nalp10	NM_175532.2	Mus musculus NACHT, leucine rich repeat and PYD containing 10 (Nalp10), mRNA.
scl29514.8.22_6-S	23.4546	1.27	Mlf2	NM_145385.1	Mus musculus myeloid leukemia factor 2 (Mlf2), mRNA.
scl19173.28.1_24-S	23.4364	1.56	Abcb11	NM_021022.2	Mus musculus ATP-binding cassette, sub-family B (MDR/TAP), member 11 (Abcb11), mRNA.
scl0140492.8_64-S	23.4104	2.01	Kcnn2	NM_080465.1	Mus musculus potassium intermediate/small conductance calcium-activated channel, subfamily N, member 2 (Kcnn2), mRNA.
scl35771.8.1_41-S	23.3525	7.2	2410076I21Rik	XM_134948.2	Mus musculus RIKEN cDNA 2410076I21 gene (2410076I21Rik), mRNA.
scl074114.18_49-S	23.3368	4.65	Crot	NM_023733.2	Mus musculus carnitine O-octanoyltransferase (Crot), mRNA.

scl0019188.1_50-S	23.3211	1.48	Psme2	NM_011190.2	Mus musculus proteasome (prosome, macropain) 28 subunit, beta (Psme2), mRNA.
scl16476.4_568-S	23.3063	1.59	Hes6	NM_019479.2	Mus musculus hairy and enhancer of split 6 (Drosophila) (Hes6), mRNA.
scl46038.17.1_26-S	23.2966	1.78	Tdrd3	NM_172605.2	Mus musculus tudor domain containing 3 (Tdrd3), mRNA.
scl00353187.1_48-S	23.2498	2.98	Nr1d2	NM_011584.2	Mus musculus nuclear receptor subfamily 1, group D, member 2 (Nr1d2), mRNA.
scl53554.5.129_30-S	23.2108	1.78	Bad	NM_007522.1	Mus musculus Bcl-associated death promoter (Bad), mRNA.
scl0067210.1_10-S	23.1875	5.65	Odag	NM_026033.1	Mus musculus ocular development associated gene (Odag), mRNA.
scl00224938.2_37-S	23.1864	1.27	Pja2	NM_144859.1	Mus musculus praja 2, RING-H2 motif containing (Pja2), mRNA.
scl00319713.1_52-S	23.1775	2.32	D930036B08Rik	NM_198649.1	Mus musculus RIKEN cDNA D930036B08 gene (D930036B08Rik), mRNA.
scl0234736.2_42-S	23.1609	2.7	BC027246	XM_355947.1	Mus musculus cDNA sequence BC027246 (BC027246), mRNA.
scl43966.3_250-S	23.1602	2.47	Nfil3	NM_017373.2	Mus musculus nuclear factor, interleukin 3, regulated (Nfil3), mRNA.
scl0001383.1_41-S	23.0575	1.41	Stx8	NM_018768.1	Mus musculus syntaxin 8 (Stx8), mRNA.
scl0217695.1_22-S	23.0566	1.87	Zfyve1	NM_183154.1	Mus musculus zinc finger, FYVE domain containing 1 (Zfyve1), mRNA.
scl00226432.1_235-S	23.0218	1.54	Ipo9	XM_129442.3	Mus musculus importin 9 (Ipo9), mRNA.
scl0107094.1_210-S	23.0082	1.37	AA408556	NM_199447.2	Mus musculus expressed sequence AA408556 (AA408556), mRNA.
scl42978.1_15-S	22.9926	1.38	2310020H19Rik	NM_025525	Mus musculus RIKEN cDNA 2310020H19 gene (2310020H19Rik), mRNA.
scl056631.7_231-S	22.9742	1.89	Trim17	NM_031172.1	Mus musculus tripartite motif protein 17 (Trim17), mRNA.
scl41427.25.1_159-S	22.9424	1.67	Elac2	NM_023479.1	Mus musculus elaC homolog 2 (E. coli) (Elac2), mRNA.
scl46231.15_279-S	22.8724	1.27	Cab39l	NM_026908.2	
scl36049.8_66-S	22.8435	2.44	Tirap	NM_054096.1	Mus musculus toll-interleukin 1 receptor (TIR) domain-containing adaptor protein (Tirap), mRNA.
scl45946.14_20-S	22.841	1.99	Mbnl2	NM_207515.1	Mus musculus muscleblind-like 2 (Mbnl2), transcript variant 1, mRNA.
scl022130.13_33-S	22.8178	2.23	Ttf1	NM_009442.1	Mus musculus transcription termination factor 1 (Ttf1), mRNA.
scl072508.1_0-S	22.7997	3.97	Rps6kb1	NM_028259.1	Mus musculus ribosomal protein S6 kinase, polypeptide 1 (Rps6kb1), mRNA.
scl00226747.2_221-S	22.7987	2.36	Elys	NM_026375.1	Mus musculus embryonic large molecule derived from yolk sac (Elys), mRNA.
scl50024.4.1_14-S	22.7341	1.77	H2-Ea	NM_010381.2	Mus musculus histocompatibility 2, class II antigen E alpha (H2-Ea), mRNA.
scl42012.5.1_30-S	22.7202	2.22	Nudt14	NM_025399.1	Mus musculus nudix (nucleoside diphosphate linked moiety X)-type motif 14 (Nudt14), mRNA.
scl074194.1_11-S	22.6782	8.39	Rhoe	NM_028810.1	Mus musculus ras homolog gene family, member E (Rhoe), mRNA.
scl0067684.1_211-S	22.6753	1.4	3300001P08Rik	NM_026313.1	Mus musculus RIKEN cDNA 3300001P08 gene (3300001P08Rik), mRNA.
scl0027410.2_52-S	22.6734	2.43	Abca3	NM_013855.1	Mus musculus ATP-binding cassette, sub-family A (ABC1), member 3 (Abca3), mRNA.
scl0003065.1_14-S	22.5996	1.41	H13	NM_010376.2	Mus musculus histocompatibility 13 (H13), mRNA.
scl30734.8.1_4-S	22.5926	1.35	Crym	NM_016669.1	Mus musculus crystallin, mu (Crym), mRNA.
scl028028.1_213-S	22.5826	1.25	Mrpl50	NM_178603.2	Mus musculus mitochondrial ribosomal protein L50 (Mrpl50), mRNA.
scl51888.24_434-S	22.5555	2.06	Pdgfrb	NM_008809.1	Mus musculus platelet derived growth factor receptor, beta polypeptide (Pdgfrb), mRNA.
scl0072098.1_29-S	22.5167	2.5	2010300G19Rik	NM_028097.2	Mus musculus RIKEN cDNA 2010300G19 gene (2010300G19Rik), mRNA.
scl066335.13_14-S	22.4898	1.4	Atp6v1c1	NM_025494.1	Mus musculus ATPase, H+ transporting, V1 subunit C, isoform 1 (Atp6v1c1), mRNA.
scl0002566.1_2-S	22.4875	1.64	Myg1	NM_021713.1	Mus musculus melanocyte prolifeating gene 1 (Myg1), mRNA.
scl0001820.1_53-S	22.4661	2.18	Son	NM_019973.1	Mus musculus Son cell proliferation protein (Son), transcript variant 1, mRNA.
scl0229227.1_8-S	22.461	2.65	D630029K19Rik	NM_172680.2	Mus musculus RIKEN cDNA D630029K19 gene (D630029K19Rik), mRNA.
scl55045.9_124-S	22.4553	1.79	Tm4sf2	NM_019634.1	Mus musculus transmembrane 4 superfamily member 2 (Tm4sf2), mRNA.
IGKV4-62_AJ231210_Ig_	22.4407	7.78			
scl0076497.2_72-S	22.3867	1.34	Ppp1r11	NM_029632.1	Mus musculus protein phosphatase 1, regulatory (inhibitor) subunit 11 (Ppp1r11), mRNA.
scl019885.11_24-S	22.3751	2.29	Rorc	NM_011281.1	Mus musculus RAR-related orphan receptor gamma (Rorc), mRNA.
scl29631.10_10-S	22.3651	1.36	Creld1	NM_133930.1	Mus musculus cysteine-rich with EGF-like domains 1 (Creld1), mRNA.
scl22672.6.1_17-S	22.3629	4.29	F3	NM_010171.2	Mus musculus coagulation factor III (F3), mRNA.
scl0223672.1_326-S	22.3415	2.13	LOC223672	XM_128064.4	Mus musculus hypothetical protein LOC223672 (LOC223672), mRNA.
scl000176.1_12-S	22.3405	1.56	Psma1	NM_011965	Mus musculus proteasome (prosome, macropain) subunit, alpha type 1 (Psma1), mRNA.
scl27370.13_382-S	22.3063	1.28	Sppl3	NM_029012.1	
scl0066884.2_148-S	22.2722	1.28	Appbp2	NM_025825.2	Mus musculus amyloid beta precursor protein (cytoplasmic tail) binding protein 2 (Appbp2), mRNA.
scl50156.18_539-S	22.254	1.79	Rab11fip3	NM_153140.1	
scl41711.15_185-S	22.2497	3.08	Fbxw11	NM_134015.1	
scl40710.6.1_96-S	22.243	1.91	2310004N24Rik	XM_181318.4	Mus musculus RIKEN cDNA 2310004N24 gene (2310004N24Rik), mRNA.
scl0011938.1_293-S	22.2344	3.31	Atp2a2	NM_009722.1	Mus musculus ATPase, Ca++ transporting, cardiac muscle, slow twitch 2 (Atp2a2), mRNA.
scl066922.3_13-S	22.2337	1.84	Rras2	NM_025846.1	Mus musculus related RAS viral (r-ras) oncogene homolog 2 (Rras2), mRNA.
scl40850.12.1_163-S	22.2334	1.24	Nmt1	NM_008707.2	Mus musculus N-myristoyltransferase 1 (Nmt1), mRNA.
scl0320204.2_97-S	22.227	1.99	4833442J19Rik	NM_177101.2	Mus musculus RIKEN cDNA 4833442J19 gene (4833442J19Rik), mRNA.
scl22833.10_29-S	22.2259	1.35	Hmgcs2	NM_008256.2	Mus musculus 3-hydroxy-3-methylglutaryl-Coenzyme A synthase 2 (Hmgcs2), mRNA.
scl27787.7_486-S	22.2233	1.49	0610040J01Rik	NM_029554.2	Mus musculus RIKEN cDNA 0610040J01 gene (0610040J01Rik), mRNA.

scl000968.1_12-S	22.2111	2.31	Uchl5	NM_019562.1	Mus musculus ubiquitin carboxyl-terminal esterase L5 (Uchl5), mRNA.
scl41115.27.1_34-S	22.1998	4.1	Myohd1	NM_025414.2	Mus musculus myosin head domain containing 1 (Myohd1), mRNA.
scl0014664.2_299-S	22.145	1.35	Slc6a9	NM_008135.1	Mus musculus solute carrier family 6 (neurotransmitter transporter, glycine), member 9 (Slc6a9), mRNA.
scl47072.5_281-S	22.1377	2.59	Ly6a	NM_010738.2	Mus musculus lymphocyte antigen 6 complex, locus A (Ly6a), mRNA.
scl47123.18_98-S	22.1174	2.02	Ndrg1	NM_010884.1	
scl0015379.2_121-S	22.1063	2.8	Onecut1	NM_008262.2	Mus musculus one cut domain, family member 1 (Onecut1), mRNA.
scl27764.24_495-S	22.0975	1.25	B3bp	XM_132065.3	
scl53403.26.13_76-S	22.0949	1.21	Bscl2	NM_008144.3	Mus musculus Bernardinelli-Seip congenital lipodystrophy 2 homolog (human) (Bscl2), mRNA.
scl0002700.1_45-S	21.9907	1.43	Nbn	NM_013752	Mus musculus nibrin (Nbn), mRNA.
scl31487.7.1_48-S	21.9842	1.62	6030457N17Rik	XM_133378.3	Mus musculus RIKEN cDNA 6030457N17 gene (6030457N17Rik), mRNA.
scl45926.20.1_29-S	21.9691	1.6	Pcca	NM_144844.1	Mus musculus propionyl-Coenzyme A carboxylase, alpha polypeptide (Pcca), mRNA.
gi_6679936_ref_NM_008(	21.9672	1.33			
scl00244713.2_109-S	21.9267	2.61	Zfp75	NM_172918.3	
scl35384.3.9_24-S	21.9256	2.44	Armet	NM_029103.1	Mus musculus arginine-rich, mutated in early stage tumors (Armet), mRNA.
scl45724.3.1_2-S	21.9166	1.77	2200001I15Rik	NM_183278.1	Mus musculus RIKEN cDNA 2200001I15 gene (2200001I15Rik), mRNA.
scl28759.2_148-S	21.9	1.54	Cml1	NM_023160.1	Mus musculus camello-like 1 (Cml1), mRNA.
scl067246.4_216-S	21.8792	2.79	2810474O19Rik	XM_132966.3	Mus musculus RIKEN cDNA 2810474O19 gene (2810474O19Rik), mRNA.
scl40200.14_192-S	21.8526	1.38	1810073G14Rik	NM_026342.1	Mus musculus RIKEN cDNA 1810073G14 gene (1810073G14Rik), mRNA.
scl066916.1_19-S	21.8417	2	Ndufb7	NM_025843.1	Mus musculus NADH dehydrogenase (ubiquinone) 1 beta subcomplex, 7 (Ndufb7), mRNA.
scl0170658.2_22-S	21.839	1.21	Ndufs5	XM_355517.1	Mus musculus NADH dehydrogenase (ubiquinone) Fe-S protein 5 (Ndufs5), mRNA.
scl25188.13.1_0-S	21.8141	1.59	C8b	NM_133882.1	Mus musculus complement component 8, beta subunit (C8b), mRNA.
scl44903.11.3_96-S	21.8038	1.5	Fars1	NM_024274.1	Mus musculus phenylalanine-tRNA synthetase 1 (mitochondrial) (Fars1), mRNA.
scl022190.1_154-S	21.7656	2.24	Ubc	XM_147315.1	Mus musculus ubiquitin C (Ubc), mRNA.
IGKV4-79_AJ231214_Ig_	21.7527	11.03	LOC213684	XM_135590.2	Mus musculus similar to immunoglobulin light chain variable region (LOC213684), mRNA.
scl068943.2_5-S	21.742	1.27	Pink1	NM_026880	Mus musculus PTEN induced putative kinase 1 (Pink1), mRNA.
scl080751.6_5-S	21.7369	1.43	Rnf34	NM_030564.1	Mus musculus ring finger protein 34 (Rnf34), mRNA.
scl50218.10.128_2-S	21.6968	1.82	5730457F11Rik	NM_172935	Mus musculus RIKEN cDNA 5730457F11 gene (5730457F11Rik), mRNA.
scl26903.5_40-S	21.6804	23.27	AI481214	NM_054098.2	
scl0003493.1_408-S	21.6717	2.56	Ppp1r1c	NM_033264.1	Mus musculus protein phosphatase 1, regulatory (inhibitor) subunit 1C (Ppp1r1c), mRNA.
scl0002080.1_55-S	21.6696	1.48	39340	NM_053102.1	
scl34059.10_20-S	21.6624	1.21	Lamp1	NM_010684	Mus musculus lysosomal membrane glycoprotein 1 (Lamp1), mRNA.
scl21036.31.1_8-S	21.6537	1.88	Mapkap1	NM_177345.2	Mus musculus mitogen-activated protein kinase associated protein 1 (Mapkap1), mRNA.
scl32648.17_169-S	21.5889	1.22	Gtf2h1	NM_008186.2	Mus musculus general transcription factor II H, polypeptide 1 (Gtf2h1), mRNA.
scl34420.4.1_19-S	21.5764	2.68	Cklfsf4	NM_153582.3	Mus musculus chemokine-like factor super family 4 (Cklfsf4), mRNA.
scl0074144.2_313-S	21.5171	1.28	Robo4	NM_028783.2	Mus musculus roundabout homolog 4 (Drosophila) (Robo4), mRNA.
scl19399.8_192-S	21.4709	1.33	Epb7.2	NM_013515.1	Mus musculus erythrocyte protein band 7.2 (Epb7.2), mRNA.
scl45512.6.1_7-S	21.4475	2.01	Gzmn	NM_153052.1	Mus musculus granzyme N (Gzmn), mRNA.
scl0015446.2_268-S	21.4433	1.53	Hpgd	NM_008278.1	Mus musculus hydroxyprostaglandin dehydrogenase 15 (NAD) (Hpgd), mRNA.
scl00225358.2_258-S	21.441	1.75	2610024E20Rik	NM_146084.1	
scl076014.3_8-S	21.4244	1.46	5830416A07Rik	NM_024268.1	Mus musculus RIKEN cDNA 5830416A07 gene (5830416A07Rik), mRNA.
scl21095.3.1_3-S	21.4189	1.28	Endog	NM_007931.1	Mus musculus endonuclease G (Endog), mRNA.
scl00319157.1_0-S	21.4106	1.9	Hist1h4f	NM_175655.1	Mus musculus histone 1, H4f (Hist1h4f), mRNA.
scl0011677.2_223-S	21.404	3.04	Akr1b3	NM_009658.2	Mus musculus aldo-keto reductase family 1, member B3 (aldose reductase) (Akr1b3), mRNA.
scl019298.8_30-S	21.3784	1.21	Pex19	NM_023041.2	Mus musculus peroxisome biogenesis factor 19 (Pex19), mRNA.
scl066242.2_14-S	21.3777	1.49	Mrps16	NM_025440.1	Mus musculus mitochondrial ribosomal protein S16 (Mrps16), mRNA.
scl079464.11_51-S	21.3753	1.42	Lias	NM_024471.2	Mus musculus lipoic acid synthetase (Lias), mRNA.
scl093836.1_167-S	21.3678	2.22	Rnf111	NM_033604.1	Mus musculus ring finger 111 (Rnf111), mRNA.
scl41336.2.1_23-S	21.3071	1.25	C730027E14Rik	NM_146020.1	Mus musculus RIKEN cDNA C730027E14 gene (C730027E14Rik), mRNA.
scl0102294.1_224-S	21.2934	1.95	Cyp4v3	NM_133969.1	Mus musculus cytochrome P450, family 4, subfamily v, polypeptide 3 (Cyp4v3), mRNA.
scl0070503.2_198-S	21.2932	1.82	5730402C02Rik	NM_027442.3	
scl0231386.4_56-S	21.2858	1.33	A730098D12Rik	NM_177680.2	Mus musculus RIKEN cDNA A730098D12 gene (A730098D12Rik), mRNA.
scl068776.1_229-S	21.2319	1.29	Taf11	NM_026836.1	Mus musculus TAF11 RNA polymerase II, TATA box binding protein (TBP)-associated factor (Taf11), mRNA.
scl0001737.1_18-S	21.2235	1.33		AK002608.1	Mus musculus adult male kidney cDNA, RIKEN full-length enriched library, clone:0610012J07 product:3-hydroxyanthranilate 3,4-dioxygenase, full insert sequence.
scl019186.11_4-S	21.2056	1.26	Psme1	NM_011189.1	Mus musculus proteasome (prosome, macropain) 28 subunit, alpha (Psme1), mRNA.
scl000093.1_57_REVCOM	21.2018	1.29	0610007P22Rik	NM_026676.1	Mus musculus RIKEN cDNA 0610007P22 gene (0610007P22Rik), mRNA.
scl25991.5.1_3-S	21.2001	1.24	Sbds	NM_023248.1	Mus musculus Shwachman-Bodian-Diamond syndrome homolog (human) (Sbds), mRNA.

scl0217837.1_325-S	21.1994	1.31	Itpk1	NM_172584.1	Mus musculus inositol 1,3,4-triphosphate 5/6 kinase (Itpk1), mRNA.
scl012799.3_0-S	21.1582	2.54	Cnp1	NM_009923.1	Mus musculus cyclic nucleotide phosphodiesterase 1 (Cnp1), mRNA.
scl53274.2_128-S	21.1533	1.27	Bteb1	NM_010638.2	Mus musculus basic transcription element binding protein 1 (Bteb1), mRNA.
scl18545.1_131-S	21.15	7.4	Thbd	NM_009378.1	Mus musculus thrombomodulin (Thbd), mRNA.
scl026889.3_52-S	21.1477	1.42	Cln8	NM_012000.2	Mus musculus ceroid-lipofuscinosis, neuronal 8 (Cln8), mRNA.
scl40132.19.1_31-S	21.132	1.5	1300013J15Rik	NM_026183.2	Mus musculus RIKEN cDNA 1300013J15 gene (1300013J15Rik), mRNA.
scl00269587.2_100-S	21.1243	1.66	Epb4.1	NM_183428.2	Mus musculus erythrocyte protein band 4.1 (Epb4.1), mRNA.
scl35712.22.1_80-S	21.1141	2.46	4921504K03Rik	XM_134960.2	Mus musculus RIKEN cDNA 4921504K03 gene (4921504K03Rik), mRNA.
scl0066973.1_60-S	21.1028	1.67	Mrps18b	NM_025878.1	Mus musculus mitochondrial ribosomal protein S18B (Mrps18b), mRNA.
scl020926.1_330-S	21.076	1.27	Supt6h	NM_009297.1	Mus musculus suppressor of Ty 6 homolog (S. cerevisiae) (Supt6h), mRNA.
scl0098366.1_13-S	21.0581	1.39	Smap1	NM_028534.1	Mus musculus stromal membrane-associated protein 1 (Smap1), mRNA.
scl38713.11.10_6-S	21.0062	1.27	Bsg	NM_009768.1	Mus musculus basigin (Bsg), mRNA.
scl29584.3_468-S	21.0034	1.24	Zfp637	NM_177684.2	
scl30348.4.1_28-S	20.9961	1.73	Lsm8	NM_133939.1	Mus musculus LSM8 homolog, U6 small nuclear RNA associated (S. cerevisiae) (Lsm8), mRNA.
scl00048.1_13-S	20.9943	1.4	Sirt3	NM_022433	Mus musculus sirtuin 3 (silent mating type information regulation 2, homolog) 3 (S. cerevisiae) (Sirt3), mRNA.
scl078090.2_4-S	20.9427	2.99	Golgb1	XM_148244.4	
scl0003793.1_0-S	20.9384	2.9	Metap2	NM_019648.2	Mus musculus methionine aminopeptidase 2 (Metap2), mRNA.
scl51145.31.1_12-S	20.9348	3.34	Pde10a	NM_011866.1	Mus musculus phosphodiesterase 10A (Pde10a), mRNA.
scl0014863.1_0-S	20.9226	2.58	Gstm2	NM_008183.2	Mus musculus glutathione S-transferase, mu 2 (Gstm2), mRNA.
scl070153.1_299-S	20.904	3.57	2210016F16Rik	XM_354755.1	Mus musculus RIKEN cDNA 2210016F16 gene (2210016F16Rik), mRNA.
scl45409.11_50-S	20.899	1.32	Gulo	NM_178747.2	Mus musculus gulonolactone (L-) oxidase (Gulo), mRNA.
scl000989.1_15-S	20.8808	2.23	sty	U21209	
scl016418.1_114-S	20.8654	1.48	Itgb4bp	NM_010579.1	Mus musculus integrin beta 4 binding protein (Itgb4bp), mRNA.
scl00319513.1_270-S	20.8547	2.55	A930025D01Rik	NM_178762.2	Mus musculus RIKEN cDNA A930025D01 gene (A930025D01Rik), mRNA.
scl0103554.4_30-S	20.8449	1.28	Psme4	NM_134013.2	Mus musculus proteasome (prosome, macropain) activator subunit 4 (Psme4), mRNA.
scl027096.1_199-S	20.8354	1.25	Trappc3	NM_013718.2	Mus musculus trafficking protein particle complex 3 (Trappc3), mRNA.
scl25637.7_145-S	20.832	1.52	Ttpa	NM_015767.1	Mus musculus tocopherol (alpha) transfer protein (Ttpa), mRNA.
scl0001527.1_23-S	20.8271	2.24	Sat2	XM_181304.3	Mus musculus spermidine/spermine N1-acetyl transferase 2 (Sat2), mRNA.
scl0231571.13_44-S	20.8159	1.49	AW060207	NM_144911.1	Mus musculus expressed sequence AW060207 (AW060207), mRNA.
scl44578.8.1_13-S	20.8059	1.29	Cetn3	NM_007684.2	Mus musculus centrin 3 (Cetn3), mRNA.
scl25735.19_37-S	20.7991	5.73	Hsp105	NM_013559.1	Mus musculus heat shock protein 105 (Hsp105), mRNA.
scl49191.18.1_6-S	20.7966	2.19	Sema5b	XM_147227.1	Mus musculus sema domain, seven thrombospondin repeats (type 1 and type 1-like), transmembrane domain (TM) and short cytoplasmic domain, (semaphorin) 5B (Sema5b), mRNA.
scl021783.10_20-S	20.7962	3.17	A330080J22Rik	NM_178667.2	
scl00213484.2_217-S	20.7734	1.6	BC036718	NM_153136.1	Mus musculus cDNA sequence BC036718 (BC036718), mRNA.
scl29246.24.1_18-S	20.7555	1.96	Aass	NM_013930.2	Mus musculus aminoadipate-semialdehyde synthase (Aass), mRNA.
scl0001867.1_58-S	20.7268	1.56	Sfrs10	NM_009186	Mus musculus splicing factor, arginine/serine-rich 10 (transformer 2 homolog, Drosophila) (Sfrs10), mRNA.
scl22085.6.1_94-S	20.7199	1.44	Rarres1	XM_130987.3	
scl34048.16.227_13-S	20.6888	3.34	Cdc16	NM_027276.1	Mus musculus CDC16 cell division cycle 16 homolog (S. cerevisiae) (Cdc16), mRNA.
scl018221.7_88-S	20.6876	1.78	Nudc	NM_010948.1	Mus musculus nuclear distribution gene C homolog (Aspergillus) (Nudc), mRNA.
scl46411.5.1_45-S	20.6633	1.53	1810009H17Rik	XM_283205.1	
scl0002178.1_304-S	20.6264	1.32	2010005A06Rik	NM_029357.2	Mus musculus RIKEN cDNA 2010005A06 gene (2010005A06Rik), mRNA.
scl24984.5.1_10-S	20.6143	1.61	BC023823	NM_153566.1	Mus musculus cDNA sequence BC023823 (BC023823), mRNA.
scl022770.1_29-S	20.5992	1.55	Zhx1	NM_009572.2	Mus musculus zinc fingers and homeoboxes protein 1 (Zhx1), mRNA.
scl0067554.2_226-S	20.5979	2.62	Slc25a30	NM_026232.1	
scl0266632.2_59-S	20.5961	2.27	Irak4	NM_029926.3	Mus musculus interleukin-1 receptor-associated kinase 4 (Irak4), mRNA.
scl32610.6.1_5-S	20.5817	2.26	6430529G09Rik	NM_178706.2	Mus musculus RIKEN cDNA 6430529G09 gene (6430529G09Rik), mRNA.
scl23003.7.28_144-S	20.5608	1.23	0610031J06Rik	NM_020003.1	Mus musculus RIKEN cDNA 0610031J06 gene (0610031J06Rik), mRNA.
scl0002068.1_1-S	20.5459	2.14	Casp6	NM_009811.2	Mus musculus caspase 6 (Casp6), mRNA.
scl21752.24.8_3-S	20.5056	1.29	Atp1a1	NM_144900.1	Mus musculus ATPase, Na+/K+ transporting, alpha 1 polypeptide (Atp1a1), mRNA.
scl067072.6_68-S	20.4957	1.42	Cdc37l	NM_025950.1	
scl28971.3_6-S	20.4861	2.79	Temt	NM_009349	Mus musculus thioether S-methyltransferase (Temt), mRNA.
scl49589.13.8_8-S	20.4719	1.4	2810036L13Rik	NM_144802.2	Mus musculus RIKEN cDNA 2810036L13 gene (2810036L13Rik), mRNA.
scl069202.1_161-S	20.4615	1.71	2610009E16Rik	XM_355811	Mus musculus RIKEN cDNA 2610009E16 gene (2610009E16Rik), mRNA.
scl066469.2_10-S	20.4384	1.75	2810405K02Rik	NM_025582.2	Mus musculus RIKEN cDNA 2810405K02 gene (2810405K02Rik), mRNA.
scl0003593.1_5-S	20.4295	2.39	Armc8	NM_028768.1	
scl0001455.1_38-S	20.4253	1.82	Slc25a11	NM_024211.2	Mus musculus solute carrier family 25 (mitochondrial carrier; oxoglutarate carrier), member 11 (Slc25a11), mRNA.

scl0003843.1_1-S	20.4093	2.75	Dcn	NM_007833.1	Mus musculus decorin (Dcn), mRNA.
scl25601.18_410-S	20.3956	1.64	Map3k7	NM_172688.1	Mus musculus mitogen activated protein kinase kinase kinase 7 (Map3k7), mRNA.
scl0002552.1_594-S	20.3908	1.21	Triobp	NM_138579.2	Mus musculus TRIO and F-actin binding protein (Triobp), mRNA.
scl00235315.2_221-S	20.3613	1.73	D130054N24Rik	NM_178709.2	Mus musculus RIKEN cDNA D130054N24 gene (D130054N24Rik), mRNA.
scl0015516.2_121-S	20.3242	1.69	Hspcb	NM_008302.2	Mus musculus heat shock protein 1, beta (Hspcb), mRNA.
scl0003223.1_29-S	20.3112	1.77	Mrrf	NM_026422.1	Mus musculus mitochondrial ribosome recycling factor (Mrrf), mRNA.
scl0002832.1_59-S	20.301	1.73	Ptp4a2	NM_008974.2	Mus musculus protein tyrosine phosphatase 4a2 (Ptp4a2), mRNA.
scl52458.4_47-S	20.2867	1.25	Slc25a28	NM_145156.1	Mus musculus solute carrier family 25, member 28 (Slc25a28), mRNA.
scl15896.3_346-S	20.2691	5.06	Grem2	NM_011825.1	
scl33916.7_57-S	20.2512	1.25	1810045K07Rik	NM_026432.2	Mus musculus RIKEN cDNA 1810045K07 gene (1810045K07Rik), mRNA.
scl42862.16_16-S	20.2377	3.49	Cpsf2	NM_016856.2	Mus musculus cleavage and polyadenylation specific factor 2 (Cpsf2), mRNA.
scl015040.2_130-S	20.2297	1.32	H2-T23	NM_010398	Mus musculus histocompatibility 2, T region locus 23 (H2-T23), mRNA.
scl24881.12.1_60-S	20.2191	1.45	Nrbf1	NM_025297.1	Mus musculus nuclear receptor binding factor 1 (Nrbf1), mRNA.
scl35396.14.1_38-S	20.1006	1.71	Acy1	NM_025371.1	Mus musculus aminoacylase 1 (Acy1), mRNA.
scl0014007.1_233-S	20.0757	1.66	Cugbp2	NM_010160.1	Mus musculus CUG triplet repeat,RNA binding protein 2 (Cugbp2), mRNA.
scl066079.4_60-S	20.0686	1.31	0610027O18Rik	NM_025339.2	Mus musculus RIKEN cDNA 0610027O18 gene (0610027O18Rik), mRNA.
scl0067444.2_48-S	20.0671	1.29	0710007A14Rik	NM_023343.1	Mus musculus RIKEN cDNA 0710007A14 gene (0710007A14Rik), mRNA.
scl33531.19_441-S	20.0415	1.41	Cyld	NM_173369.1	Mus musculus cylindromatosis (turban tumor syndrome) (Cyld), mRNA.
scl21165.1.12_85-S	20.0112	8.55	Bmyc	NM_023326	Mus musculus brain expressed myelocytomatosis oncogene (Bmyc), mRNA.
scl0016923.1_194-S	20.006	1.37	Lnk	NM_008507.2	Mus musculus linker of T-cell receptor pathways (Lnk), mRNA.
					**GENES DOWN-REGULATED BY THE ATHEROGENIC DIET IN LIVERS**
**TargetID**	**Diff_Score**	**Fold_Change**	**Symbol**	**Accession**	**Definition**
scl0002105.1_331-S	-338.8377	-69.04	Kcnn3	XM_147305.1	Mus musculus potassium intermediate/small conductance calcium-activated channel, subfamily N, member 3 (Kcnn3), mRNA.
scl0001.1_3-S	-336.6852	-8.98	9130229H14Rik	XM_135706.3	Mus musculus RIKEN cDNA 9130229H14 gene (9130229H14Rik), mRNA.
scl015204.23_217-S	-322.8666	-5.29	Herc2	NM_010418.1	Mus musculus hect (homologous to the E6-AP (UBE3A) carboxyl terminus) domain and RCC1 (CHC1)-like domain (RLD) 2 (Herc2), mRNA.
scl43919.15.1_45-S	-260.9584	-4.89	Ddx41	NM_134059.1	Mus musculus DEAD (Asp-Glu-Ala-Asp) box polypeptide 41 (Ddx41), mRNA.
scl020382.2_66-S	-252.3905	-3.04	Sfrs2	NM_011358.1	Mus musculus splicing factor, arginine/serine-rich 2 (SC-35) (Sfrs2), mRNA.
scl000233.1_72-S	-167.5482	-3.76	Bcl2l12	NM_029410.1	Mus musculus BCL2-like 12 (proline rich) (Bcl2l12), mRNA.
scl00094.1_14-S	-152.7796	-2	Hpn	NM_008281.1	Mus musculus hepsin (Hpn), mRNA.
scl078779.1_155-S	-150.1237	-3	2610039E05Rik	NM_030176.2	Mus musculus RIKEN cDNA 2610039E05 gene (2610039E05Rik), mRNA.
scl0001670.1_34-S	-139.8516	-3.64	Cbs	NM_144855.1	Mus musculus cystathionine beta-synthase (Cbs), transcript variant 1, mRNA.
scl0003961.1_4-S	-134.7176	-7.89	Cdkl2	NM_016912.1	Mus musculus cyclin-dependent kinase-like 2 (CDC2-related kinase) (Cdkl2), mRNA.
scl0003591.1_99-S	-134.4637	-2.32	Keap1	NM_016679.2	Mus musculus kelch-like ECH-associated protein 1 (Keap1), mRNA.
scl0016570.1_92-S	-132.5514	-23.66	Kif3c	NM_008445.1	Mus musculus kinesin family member 3C (Kif3c), mRNA.
scl46590.24_108-S	-122.3603	-2.24	Sec24c	NM_172596.1	
scl0013688.1_69-S	-120.4288	-3.19	Eif4ebp2	NM_010124.1	Mus musculus eukaryotic translation initiation factor 4E binding protein 2 (Eif4ebp2), mRNA.
scl00225339.2_67-S	-118.952	-1.88	E230022H04Rik	NM_153515.2	Mus musculus RIKEN cDNA E230022H04 gene (E230022H04Rik), mRNA.
scl000274.1_354-S	-112.5034	-2.03	Tnrc6	NM_144925.2	Mus musculus trinucleotide repeat containing 6 (Tnrc6), mRNA.
scl0241638.1_112-S	-100.6696	-1.88	Prosapip1	NM_197945.2	Mus musculus ProSAPiP1 protein (Prosapip1), mRNA.
scl46996.5.2_2-S	-100.2137	-14.2	Pvalb	NM_013645.2	
scl52906.7.1_110-S	-100.1966	-2.07	Vegfb	NM_011697.1	Mus musculus vascular endothelial growth factor B (Vegfb), mRNA.
scl21063.24_624-S	-98.0146	-1.74	Golga2	NM_133852.1	Mus musculus golgi autoantigen, golgin subfamily a, 2 (Golga2), mRNA.
scl45859.16.1_70-S	-96.3327	-1.94	5730461K03Rik	NM_027475.1	Mus musculus RIKEN cDNA 5730461K03 gene (5730461K03Rik), mRNA.
scl014661.15_41-S	-94.8377	-1.58	Glud1	NM_008133.2	
scl36214.3_394-S	-88.1682	-1.91	Gbif	NM_019683.2	Mus musculus globin inducing factor, fetal (Gbif), mRNA.
scl29749.21.1_4-S	-86.968	-1.59	Fthfd	NM_027406.1	Mus musculus formyltetrahydrofolate dehydrogenase (Fthfd), mRNA.
scl052120.1_108-S	-85.7551	-1.55	D8Ertd354e	NM_133970	Mus musculus DNA segment, Chr 8, ERATO Doi 354, expressed (D8Ertd354e), mRNA.
scl48906.7.1_24-S	-85.4812	-12.27	4930590A17Rik	XM_148730.1	Mus musculus RIKEN cDNA 4930590A17 gene (4930590A17Rik), mRNA.
scl0071752.2_61-S	-85.4363	-1.84	Gtf3c2	NM_027901.1	
scl50076.19.1_35-S	-85.0709	-1.99	Cbs	NM_144855.1	Mus musculus cystathionine beta-synthase (Cbs), transcript variant 1, mRNA.
scl37031.1_4-S	-83.2238	-5.1	Bcl9l	NM_030256.1	Mus musculus B-cell CLL/lymphoma 9-like (Bcl9l), mRNA.
scl44033.1.1176_137-S	-82.2412	-8.37	2900016G23Rik	XM_147738.1	Mus musculus RIKEN cDNA 2900016G23 gene (2900016G23Rik), mRNA.
scl21077.16.1_122-S	-81.3408	-15.99	BC034076	NM_177649.3	Mus musculus cDNA sequence BC034076 (BC034076), mRNA.
scl39936.8.1_29-S	-79.9242	-1.81	Serpinf2	NM_008878.1	Mus musculus serine (or cysteine) proteinase inhibitor, clade F, member 2 (Serpinf2), mRNA.
scl29407.39.1_52-S	-79.4838	-2.44	Pik3c2g	NM_011084	Mus musculus phosphatidylinositol 3-kinase, C2 domain containing, gamma polypeptide (Pik3c2g), transcript variant 2, mRNA.
scl0230857.22_98-S	-78.1303	-1.64	Ece1	NM_199307.1	Mus musculus endothelin converting enzyme 1 (Ece1), mRNA.

scl26582.30.1_36-S	-78.0537	-2.13	2310045A20Rik	NM_172710.1	Mus musculus RIKEN cDNA 2310045A20 gene (2310045A20Rik), mRNA.
scl33472.9.1_1-S	-76.597	-1.58	2310005O14Rik	NM_026452	Mus musculus RIKEN cDNA 2310005O14 gene (2310005O14Rik), mRNA.
scl0004096.1_49-S	-76.5573	-2.41	Trfr2	NM_015799.2	Mus musculus transferrin receptor 2 (Trfr2), mRNA.
scl0192166.3_30-S	-75.265	-1.87	Sardh	NM_138665.1	Mus musculus sarcosine dehydrogenase (Sardh), mRNA.
scl34521.8.1_20-S	-75.1718	-2.27	BC004022	XM_134557.1	Mus musculus cDNA sequence BC004022 (BC004022), mRNA.
scl39361.6_168-S	-75.0843	-1.92	Cdc42ep4	NM_020006.1	Mus musculus CDC42 effector protein (Rho GTPase binding) 4 (Cdc42ep4), mRNA.
scl0227648.1_142-S	-73.029	-1.64	AU024582	NM_153125.1	Mus musculus expressed sequence AU024582 (AU024582), mRNA.
scl0050907.2_191-S	-72.3812	-1.68	Preb	NM_016703.1	Mus musculus prolactin regulatory element binding (Preb), mRNA.
scl015586.4_7-S	-72.0823	-2.48	Hyal1	NM_008317.2	Mus musculus hyaluronidase 1 (Hyal1), mRNA.
scl32698.6.1_30-S	-71.6796	-1.5	Irf3	NM_016849.2	Mus musculus interferon regulatory factor 3 (Irf3), mRNA.
scl0001280.1_1-S	-69.547	-1.65	Pcyt2	NM_024229.2	Mus musculus phosphate cytidylyltransferase 2, ethanolamine (Pcyt2), mRNA.
scl068035.2_9-S	-69.0257	-2.94	3100004P22Rik	NM_133693.1	Mus musculus RIKEN cDNA 3100004P22 gene (3100004P22Rik), mRNA.
scl0016987.2_34-S	-68.8042	-3.19	Lss	NM_146006	Mus musculus lanosterol synthase (Lss), mRNA.
scl054325.6_160-S	-68.4706	-2.34	Elovl1	NM_019422.1	Mus musculus elongation of very long chain fatty acids (FEN1/Elo2, SUR4/Elo3, yeast)-like 1 (Elovl1), mRNA.
scl51123.11.1_42-S	-67.8131	-13.44	Slc22a2	NM_013667.1	Mus musculus solute carrier family 22 (organic cation transporter), member 2 (Slc22a2), mRNA.
scl000480.1_1346-S	-67.5859	-11.29	C630029K18Rik	NM_144871.2	Mus musculus RIKEN cDNA C630029K18 gene (C630029K18Rik), mRNA.
scl46309.9_175-S	-67.5534	-1.94	Abhd4	NM_134076.1	Mus musculus abhydrolase domain containing 4 (Abhd4), mRNA.
scl0077683.1_104-S	-67.4394	-2.31	Ehmt1	NM_172545.1	
scl0078920.2_103-S	-66.9032	-1.49	Dlst	NM_030225.3	Mus musculus dihydrolipoamide S-succinyltransferase (E2 component of 2-oxo-glutarate complex) (Dlst), mRNA.
scl050880.12_63-S	-66.7111	-1.65	Scly	NM_016717.2	Mus musculus selenocysteine lyase (Scly), mRNA.
scl027041.12_171-S	-66.6061	-1.67	G3bp	NM_013716	Mus musculus Ras-GTPase-activating protein SH3-domain binding protein (G3bp), mRNA.
scl014673.1_90-S	-66.4029	-5.15	Gna12	NM_010302.1	Mus musculus guanine nucleotide binding protein, alpha 12 (Gna12), mRNA.
scl54754.6.1_12-S	-65.9046	-1.54	Igbp1	XM_196586.3	Mus musculus immunoglobulin (CD79A) binding protein 1 (Igbp1), mRNA.
scl38366.14_414-S	-63.5984	-1.83	Gns	NM_029364.1	Mus musculus glucosamine (N-acetyl)-6-sulfatase (Gns), mRNA.
scl32855.14.1_3-S	-63.4595	-1.48	Hnrpl	NM_177301.3	Mus musculus heterogeneous nuclear ribonucleoprotein L (Hnrpl), mRNA.
scl0018571.2_141-S	-62.2229	-4.54	Pdcd6ip	NM_011052.1	Mus musculus programmed cell death 6 interacting protein (Pdcd6ip), mRNA.
scl0077038.2_139-S	-61.7089	-1.97	Zfp289	NM_023854.1	Mus musculus zinc finger protein 289 (Zfp289), mRNA.
scl0219140.10_154-S	-61.5436	-1.6	Spata13	XM_147847.4	
scl53371.28_117-S	-61.1168	-1.44	Ddb1	NM_015735.1	Mus musculus damage specific DNA binding protein 1 (Ddb1), mRNA.
scl0227570.7_253-S	-60.7373	-1.92	2810455F06Rik	XM_130011.5	Mus musculus RIKEN cDNA 2810455F06 gene (2810455F06Rik), mRNA.
scl072090.10_13-S	-60.6883	-1.71	Entpd8	XM_203832.2	Mus musculus ectonucleoside triphosphate diphosphohydrolase 6 (Entpd8), mRNA.
scl0023881.1_86-S	-59.7832	-1.51	E430034L04Rik	NM_011816.2	Mus musculus RIKEN cDNA E430034L04 gene (E430034L04Rik), mRNA.
scl39304.10.1_23-S	-59.658	-2.04	Prpsap1	XM_181343.1	
scl21181.13_417-S	-59.3177	-1.7	E430019H13Rik	XM_130073.3	
scl15914.2_382-S	-58.9698	-2.69	Dusp23	XM_129566	
scl015235.16_24-S	-58.8242	-1.65	Mst1	NM_008243.2	Mus musculus macrophage stimulating 1 (hepatocyte growth factor-like) (Mst1), mRNA.
scl066193.1_138-S	-58.1146	-1.47	1110049F12Rik	NM_025411.2	Mus musculus RIKEN cDNA 1110049F12 gene (1110049F12Rik), mRNA.
scl0004207.1_37-S	-56.9116	-1.98	Fastk	NM_023229.1	Mus musculus Fas-activated serine/threonine kinase (Fastk), mRNA.
scl39617.28.1_49-S	-56.6631	-1.63	Thrap4	NM_011869.1	Mus musculus thyroid hormone receptor associated protein 4 (Thrap4), mRNA.
scl0013589.1_127-S	-56.5365	-1.7	Mapre1	NM_007896.2	Mus musculus microtubule-associated protein, RP/EB family, member 1 (Mapre1), mRNA.
scl0015925.2_2-S	-56.2361	-1.58	Ide	NM_031156.1	Mus musculus insulin degrading enzyme (Ide), mRNA.
scl27607.17.1_41-S	-55.7766	-1.59	Alb1	NM_009654.1	Mus musculus albumin 1 (Alb1), mRNA.
scl30510.5_349-S	-55.7634	-1.61	Bet1l	NM_018742.3	Mus musculus blocked early in transport 1 homolog (S. cerevisiae)-like (Bet1l), mRNA.
scl0114896.3_29-S	-55.5304	-1.51	Afg3l1	NM_054070.1	Mus musculus AFG3(ATPase family gene 3)-like 1 (yeast) (Afg3l1), mRNA.
scl52956.24.4_13-S	-55.3277	-3.61	Nfkb2	NM_019408.1	Mus musculus nuclear factor of kappa light polypeptide gene enhancer in B-cells 2, p49/p100 (Nfkb2), mRNA.
scl41344.9.1_32-S	-55.2514	-1.48	Asgr2	NM_007493.2	Mus musculus asialoglycoprotein receptor 2 (Asgr2), mRNA.
scl49180.5.1_127-S	-55.1092	-11.48	2010005H15Rik	NM_029733.1	Mus musculus RIKEN cDNA 2010005H15 gene (2010005H15Rik), mRNA.
scl27818.30.1_7-S	-54.7295	-2.27	Anapc4	NM_024213.1	Mus musculus anaphase promoting complex subunit 4 (Anapc4), mRNA.
scl057261.4_30-S	-54.6091	-1.96	Brd4	NM_020508.2	Mus musculus bromodomain containing 4 (Brd4), mRNA.
scl41080.12.669_137-S	-53.1	-1.73	4732452J19Rik	NM_172448.1	Mus musculus RIKEN cDNA 4732452J19 gene (4732452J19Rik), mRNA.
scl013864.1_5-S	-52.8686	-1.43	Nr2f6	NM_010150	Mus musculus nuclear receptor subfamily 2, group F, member 6 (Nr2f6), mRNA.
scl020849.21_14-S	-52.855	-9.84	Stat4	NM_011487.1	Mus musculus signal transducer and activator of transcription 4 (Stat4), mRNA.
scl019182.11_5-S	-52.743	-1.44	Psmc3	NM_008948.1	Mus musculus proteasome (prosome, macropain) 26S subunit, ATPase 3 (Psmc3), mRNA.
scl011512.2_0-S	-52.7201	-3.58	Adcy6	NM_007405.1	Mus musculus adenylate cyclase 6 (Adcy6), mRNA.
scl0013629.1_11-S	-52.4501	-1.51	Eef2	NM_007907.1	Mus musculus eukaryotic translation elongation factor 2 (Eef2), mRNA.
scl19160.22_222-S	-52.2361	-1.57	Tlk1	NM_172664.2	Mus musculus tousled-like kinase 1 (Tlk1), mRNA.

scl27388.28_359-S	-52.1723	-1.43	Ube3b	NM_054093.1	Mus musculus ubiquitin protein ligase E3B (Ube3b), mRNA.
scl00233908.1_1058-S	-51.6149	-2.18	Fus	NM_139149.1	Mus musculus fusion, derived from t(12;16) malignant liposarcoma (human) (Fus), mRNA.
scl31141.20.4_13-S	-51.3764	-2.1	Anpep	NM_008486.1	Mus musculus alanyl (membrane) aminopeptidase (Anpep), mRNA.
scl00235567.1_50-S	-51.0199	-1.9	Dnajc13	XM_135146.4	
scl0001240.1_5-S	-50.9382	-10.28	Tnfrsf1a	NM_011609.2	Mus musculus tumor necrosis factor receptor superfamily, member 1a (Tnfrsf1a), mRNA.
scl42954.11_322-S	-50.8258	-1.83	BC011209	NM_145447.1	Mus musculus cDNA sequence BC011209 (BC011209), mRNA.
scl072462.6_25-S	-50.5643	-2.69	2600005C20Rik	NM_028244.1	Mus musculus RIKEN cDNA 2600005C20 gene (2600005C20Rik), mRNA.
scl23013.4.1_25-S	-49.9953	-7.27	Crabp2	NM_007759.1	Mus musculus cellular retinoic acid binding protein II (Crabp2), mRNA.
scl098999.1_128-S	-49.4987	-2.24	AI481105	XM_130703.4	Mus musculus expressed sequence AI481105 (AI481105), mRNA.
scl0002696.1_5-S	-49.4033	-2.22	Oprs1	NM_011014.1	Mus musculus opioid receptor, sigma 1 (Oprs1), mRNA.
scl0019294.1_121-S	-49.382	-1.75	Pvrl2	NM_008990.2	Mus musculus poliovirus receptor-related 2 (Pvrl2), mRNA.
scl0002699.1_4-S	-49.2162	-1.52	Nol6	NM_139237.1	Mus musculus nucleolar protein family 6 (RNA-associated) (Nol6), transcript variant alpha, mRNA.
scl49146.12.1_4-S	-48.9455	-1.78	2610033C09Rik	NM_026407.2	Mus musculus RIKEN cDNA 2610033C09 gene (2610033C09Rik), mRNA.
scl42553.13_222-S	-48.9096	-1.39	Hbp1	NM_153198	Mus musculus high mobility group box transcription factor 1 (Hbp1), mRNA.
scl013629.6_24-S	-48.7012	-1.69	Eef2	NM_007907.1	Mus musculus eukaryotic translation elongation factor 2 (Eef2), mRNA.
scl0002409.1_31-S	-48.5304	-6.26	Dio2	NM_010050.1	Mus musculus deiodinase, iodothyronine, type II (Dio2), mRNA.
scl0069219.2_49-S	-48.5246	-3.36	Ddah1	NM_026993.1	Mus musculus dimethylarginine dimethylaminohydrolase 1 (Ddah1), mRNA.
scl068505.1_329-S	-48.508	-1.78	1110014N23Rik	XM_283543.2	
scl0001809.1_7-S	-48.476	-9.5	Dlgh1	NM_007862.2	Mus musculus discs, large homolog 1 (Drosophila) (Dlgh1), mRNA.
scl53116.10_62-S	-48.1469	-1.45	Pi4k2a	NM_145501.1	Mus musculus phosphatidylinositol 4-kinase type 2 alpha (Pi4k2a), mRNA.
scl077038.16_27-S	-47.9241	-2.09	Zfp289	NM_023854.1	Mus musculus zinc finger protein 289 (Zfp289), mRNA.
scl47431.10_195-S	-47.3428	-1.65	AW549877	NM_145930	Mus musculus expressed sequence AW549877 (AW549877), mRNA.
scl25784.5.1_54-S	-47.2067	-1.46	Pdap1	XM_132501.2	Mus musculus PDGFA associated protein 1 (Pdap1), mRNA.
scl31481.11_594-S	-46.7405	-1.66	2700023B17Rik	NM_025948.1	Mus musculus RIKEN cDNA 2700023B17 gene (2700023B17Rik), mRNA.
scl00224613.2_5-S	-46.3405	-2.52	E030034P13Rik	NM_153791.1	Mus musculus RIKEN cDNA E030034P13 gene (E030034P13Rik), mRNA.
scl071774.7_27-S	-46.2024	-1.96	1300007L22Rik	NM_027917.1	Mus musculus RIKEN cDNA 1300007L22 gene (1300007L22Rik), mRNA.
scl066989.6_85-S	-46.1924	-10.62	2410004N11Rik	NM_025888.2	Mus musculus RIKEN cDNA 2410004N11 gene (2410004N11Rik), mRNA.
scl24607.15_131-S	-46.1205	-1.74	Dvl1	NM_010091.2	Mus musculus dishevelled, dsh homolog 1 (Drosophila) (Dvl1), mRNA.
scl0232371.6_16-S	-45.954	-1.48	C1rl	NM_181344.2	Mus musculus complement component 1, r subcomponent-like (C1rl), mRNA.
scl40849.10_252-S	-45.9206	-1.48	Acbd4	NM_025988.1	Mus musculus acyl-Coenzyme A binding domain containing 4 (Acbd4), mRNA.
scl33214.20.1_19-S	-45.8024	-1.74	Spg7	NM_153176.3	Mus musculus spastic paraplegia 7 homolog (human) (Spg7), mRNA.
scl00110959.1_154-S	-45.7717	-1.38	D7Rp2e	NM_033080.1	Mus musculus DNA segment, Chr 7, Roswell Park 2 complex, expressed (D7Rp2e), mRNA.
scl34132.19.1_1-S	-45.5647	-1.54	Stxbp2	NM_011503.2	Mus musculus syntaxin binding protein 2 (Stxbp2), mRNA.
scl17782.7.1_158-S	-45.4152	-1.61	1110060O18Rik	NM_026846.1	Mus musculus RIKEN cDNA 1110060O18 gene (1110060O18Rik), mRNA.
scl44251.7.1_3-S	-45.2518	-4.34	Stard3nl	NM_024270.1	Mus musculus STARD3 N-terminal like (Stard3nl), mRNA.
scl45842.16.1_36-S	-44.8443	-1.79	Ndst2	NM_010811.1	Mus musculus N-deacetylase/N-sulfotransferase (heparan glucosaminyl) 2 (Ndst2), mRNA.
scl39472.24_150-S	-44.7949	-1.6	Nsf	NM_008740.2	Mus musculus N-ethylmaleimide sensitive fusion protein (Nsf), mRNA.
scl34974.1.1_106-S	-44.7597	-9.54	Adrb3	NM_013462.1	Mus musculus adrenergic receptor, beta 3 (Adrb3), mRNA.
scl0012606.2_178-S	-44.7546	-2.55	Cebpa	NM_007678.1	Mus musculus CCAAT/enhancer binding protein (C/EBP), alpha (Cebpa), mRNA.
scl0018111.2_84-S	-44.4641	-10.63	Nnat	NM_010923.1	Mus musculus neuronatin (Nnat), transcript variant 2, mRNA.
scl0234854.13_245-S	-44.0265	-1.58	Cdk10	NM_194444.1	Mus musculus cyclin-dependent kinase (CDC2-like) 10 (Cdk10), transcript variant 2, mRNA.
scl52839.2_29-S	-43.5848	-1.46	AI837181	NM_134149.1	Mus musculus expressed sequence AI837181 (AI837181), mRNA.
scl0268417.1_220-S	-43.1845	-1.41	Zfp496	NM_172941	
scl27224.3.7_29-S	-43.0226	-1.49	Arl6ip4	NM_144509.1	Mus musculus ADP-ribosylation factor-like 6 interacting protein 4 (Arl6ip4), mRNA.
scl36902.11_284-S	-42.8529	-2.05	Scamp2	NM_022813.2	Mus musculus secretory carrier membrane protein 2 (Scamp2), mRNA.
scl00110593.1_306-S	-42.5863	-9.49	Prdm2	XM_204027.3	
scl00268515.2_81-S	-42.4409	-7.79	BC060615	NM_198423.1	Mus musculus cDNA sequence BC060615 (BC060615), mRNA.
scl00242291.2_165-S	-42.437	-2.37	1110001C20Rik	NM_177730.2	Mus musculus RIKEN cDNA 1110001C20 gene (1110001C20Rik), mRNA.
scl0014571.1_161-S	-42.2736	-1.75	Gpd2	NM_010274.2	Mus musculus glycerol phosphate dehydrogenase 2, mitochondrial (Gpd2), mRNA.
scl0223665.1_9-S	-42.2611	-3.66	C030006K11Rik	NM_145472.1	Mus musculus RIKEN cDNA C030006K11 gene (C030006K11Rik), mRNA.
scl40257.8.9_25-S	-41.8872	-2.12	0610039K22Rik	NM_025346.1	Mus musculus RIKEN cDNA 0610039K22 gene (0610039K22Rik), mRNA.
scl0001722.1_31-S	-41.8174	-1.68	Plg	NM_008877.2	Mus musculus plasminogen (Plg), mRNA.
scl0069109.2_11-S	-41.7812	-1.72	1810009O10Rik	NM_197989.1	Mus musculus RIKEN cDNA 1810009O10 gene (1810009O10Rik), mRNA.
scl080287.9_33-S	-41.7059	-9.65	Apobec3	NM_030255.1	Mus musculus apolipoprotein B editing complex 3 (Apobec3), mRNA.
scl31848.11_248-S	-41.6129	-2.65	Dhcr7	NM_007856.2	Mus musculus 7-dehydrocholesterol reductase (Dhcr7), mRNA.
scl0002836.1_427-S	-41.5653	-2.53	Prdm2	XM_204027.3	

scl19818.14.1_56-S	-41.2061	-3.28	Th1l	NM_020580.1	Mus musculus TH1-like homolog (Drosophila) (Th1l), mRNA.
scl41270.2_525-S	-41.1172	-2.02	Rtn4rl1	NM_177708.3	Mus musculus reticulon 4 receptor-like 1 (Rtn4rl1), mRNA.
scl22887.9_86-S	-40.883	-1.82	4930504E06Rik	NM_133858.2	Mus musculus RIKEN cDNA 4930504E06 gene (4930504E06Rik), mRNA.
scl39939.4_200-S	-40.6535	-4.95	Hic1	NM_010430.1	Mus musculus hypermethylated in cancer 1 (Hic1), mRNA.
scl0029861.1_97-S	-40.5923	-8.66	Neud4	NM_013874.1	Mus musculus neuronal d4 domain family member (Neud4), mRNA.
scl36804.10.1_24-S	-40.5438	-1.56	Spg21	NM_138584.1	
scl49387.17.1_22-S	-40.4406	-4.78	Top3b	NM_011624.2	Mus musculus topoisomerase (DNA) III beta (Top3b), mRNA.
scl49862.18.1_0-S	-40.3423	-1.61	1200014P03Rik	NM_029091.1	Mus musculus RIKEN cDNA 1200014P03 gene (1200014P03Rik), mRNA.
scl0018789.1_2-S	-40.3187	-2.08	Papola	NM_011112.1	Mus musculus poly (A) polymerase alpha (Papola), mRNA.
scl0068585.2_67-S	-40.307	-1.63	Rtn4	NM_194054.1	Mus musculus reticulon 4 (Rtn4), transcript variant 5, mRNA.
scl0017274.1_278-S	-40.2648	-1.72	Rab8a	NM_023126.2	Mus musculus RAB8A, member RAS oncogene family (Rab8a), mRNA.
scl31411.7.1_30-S	-40.2214	-1.7	Nr1h2	NM_009473	Mus musculus nuclear receptor subfamily 1, group H, member 2 (Nr1h2), mRNA.
scl25538.5_299-S	-40.1828	-6.24	Ube2r2	NM_026275.2	Mus musculus ubiquitin-conjugating enzyme E2R 2 (Ube2r2), mRNA.
scl000039.1_11-S	-40.1632	-1.95	BC023957	NM_172257.1	Mus musculus cDNA sequence BC023957 (BC023957), mRNA.
scl24870.7.1_13-S	-40.1009	-1.82	D030015G18Rik	NM_146155.2	Mus musculus RIKEN cDNA D030015G18 gene (D030015G18Rik), mRNA.
scl40925.4.3563_8-S	-40.0849	-2.38	5730509C05Rik	NM_197940.1	
scl0003230.1_8-S	-39.782	-2.56	Fpgs	NM_010236.1	Mus musculus folylpolyglutamyl synthetase (Fpgs), mRNA.
scl069940.12_93-S	-39.5057	-2.06	2810407P21Rik	XM_132150.5	
scl34388.19_15-S	-39.375	-1.49	Ranbp10	NM_145824.3	
scl16275.2_366-S	-39.373	-3.34	Adora1	NM_009629.1	Mus musculus adenosine A1 receptor (Adora1), mRNA.
scl35577.16.1_50-S	-39.3402	-1.35	Fbxo9	NM_023605.1	Mus musculus f-box only protein 9 (Fbxo9), mRNA.
scl0114716.6_81-S	-39.0477	-3.33	Spred2	NM_033523.2	Mus musculus sprouty protein with EVH-1 domain 2, related sequence (Spred2), mRNA.
scl0001430.1_149-S	-38.9579	-1.45	Nags	NM_178053.2	Mus musculus N-acetylglutamate synthase (Nags), mRNA.
scl37745.17_21-S	-38.8482	-2.02	Thrap5	NM_198107.1	Mus musculus thyroid hormone receptor associated protein 5 (Thrap5), mRNA.
scl069326.1_76-S	-38.7117	-2.02	C330008K14Rik	XM_128979.4	
scl051792.12_26-S	-38.5785	-1.65	Ppp2r1a	NM_016891.2	Mus musculus protein phosphatase 2 (formerly 2A), regulatory subunit A (PR 65), alpha isoform (Ppp2r1a), mRNA.
scl0001519.1_21-S	-38.4889	-1.64	Syngr2	NM_009304.1	Mus musculus synaptogyrin 2 (Syngr2), mRNA.
scl013424.9_0-S	-38.1964	-1.86	Dnchc1	NM_030238.1	Mus musculus dynein, cytoplasmic, heavy chain 1 (Dnchc1), mRNA.
scl0231103.18_1-S	-38.1464	-1.55	Gckr	NM_144909	Mus musculus glucokinase regulatory protein (Gckr), mRNA.
scl50930.18_166-S	-38.1259	-3.49	Anks1	NM_181413.2	Mus musculus ankyrin repeat and SAM domain containing 1 (Anks1), mRNA.
scl071452.5_105-S	-38.1153	-1.49	5530600A18Rik	NM_027799.1	Mus musculus RIKEN cDNA 5530600A18 gene (5530600A18Rik), mRNA.
scl0003260.1_9-S	-38.0335	-9.39	Nfatc2	NM_010899.1	Mus musculus nuclear factor of activated T-cells, cytoplasmic, calcineurin-dependent 2 (Nfatc2), mRNA.
scl22954.14.1_9-S	-37.9739	-2.18	4632407F12Rik	XM_110709.2	Mus musculus RIKEN cDNA 4632407F12 gene (4632407F12Rik), mRNA.
scl0027376.2_9-S	-37.9614	-1.44	Slc25a10	NM_013770.1	Mus musculus solute carrier family 25 (mitochondrial carrier; dicarboxylate transporter), member 10 (Slc25a10), mRNA.
scl066870.7_39-S	-37.946	-1.37	1200009K13Rik	NM_025814.1	Mus musculus RIKEN cDNA 1200009K13 gene (1200009K13Rik), mRNA.
scl20894.20.1_21-S	-37.821	-2.71	Pkp4	XM_130277.3	Mus musculus plakophilin 4 (Pkp4), mRNA.
scl19390.3.3_3-S	-37.5129	-1.32	Ndufa8	NM_026703.1	Mus musculus NADH dehydrogenase (ubiquinone) 1 alpha subcomplex, 8 (Ndufa8), mRNA.
scl33707.42_67-S	-37.2831	-1.43	Myo9b	NM_015742.1	Mus musculus myosin IXb (Myo9b), mRNA.
scl26219.29_122-S	-37.2607	-1.6	Ulk1	NM_009469.3	Mus musculus Unc-51 like kinase 1 (C. elegans) (Ulk1), mRNA.
scl39557.17.1_8-S	-37.2408	-1.4	Dnajc7	NM_019795.3	Mus musculus DnaJ (Hsp40) homolog, subfamily C, member 7 (Dnajc7), mRNA.
scl45764.20_72-S	-36.9214	-1.41	Nisch	NM_022656.1	Mus musculus nischarin (Nisch), mRNA.
scl066306.4_122-S	-36.6176	-2.96	2810012G03Rik	NM_175104.3	Mus musculus RIKEN cDNA 2810012G03 gene (2810012G03Rik), mRNA.
scl00227334.1_76-S	-36.3976	-4.53	Usp40	XM_129956.2	
scl0003913.1_65-S	-36.1995	-2.22	Cdk4	NM_009870.2	Mus musculus cyclin-dependent kinase 4 (Cdk4), mRNA.
scl0074781.2_60-S	-36.1852	-1.93	2510001I10Rik	NM_178398.2	
scl27911.17_304-S	-36.0663	-1.46	Sh3bp2	NM_011893	Mus musculus SH3-domain binding protein 2 (Sh3bp2), mRNA.
scl21170.7.1_39-S	-36.0303	-6.58	9230106L18Rik	NM_033145.1	Mus musculus RIKEN cDNA 9230106L18 gene (9230106L18Rik), mRNA.
scl51774.11.3_64-S	-36.0248	-1.33	Cxxc1	NM_028868.1	Mus musculus CXXC finger 1 (PHD domain) (Cxxc1), mRNA.
scl026456.19_173-S	-35.9376	-1.8	Sema4g	NM_011976.1	Mus musculus sema domain, immunoglobulin domain (Ig), transmembrane domain (TM) and short cytoplasmic domain, (semaphorin) 4G (Sema4g), mRNA.
scl0233870.10_246-S	-35.7815	-1.43	Tufm	NM_172745.1	
scl28277.12_197-S	-35.7582	-1.39	Wbp11	NM_021714.3	Mus musculus WW domain binding protein 11 (Wbp11), mRNA.
scl0003929.1_0-S	-35.7466	-1.47	ORF61	XM_109575.3	
scl093760.1_28-S	-35.6994	-1.64	Arid1a	NM_033566.1	Mus musculus AT rich interactive domain 1A (Swi1 like) (Arid1a), mRNA.
scl070302.4_170-S	-35.6664	-1.98	3110050K21Rik	XM_127780.4	Mus musculus RIKEN cDNA 3110050K21 gene (3110050K21Rik), mRNA.
scl43917.11.1_43-S	-35.5844	-2.47	BC021381	NM_145382.2	Mus musculus cDNA sequence BC021381 (BC021381), mRNA.
scl018220.1_69-S	-35.4949	-1.31	Nucb1	NM_008749.1	

scl012013.5_256-S	-35.4642	-1.71	Bach1	NM_007520.1	Mus musculus BTB and CNC homology 1 (Bach1), mRNA.
scl48388.1.45_188-S	-35.3937	-1.52	2310061J03Rik	XM_148411.1	Mus musculus RIKEN cDNA 2310061J03 gene (2310061J03Rik), mRNA.
scl0018045.2_49-S	-35.0885	-1.61	Nfyb	NM_010914.1	Mus musculus nuclear transcription factor-Y beta (Nfyb), mRNA.
scl36629.23.1_130-S	-35.0727	-4.8	Adamts7	XM_135041.5	Mus musculus a disintegrin-like and metalloprotease (reprolysin type) with thrombospondin type 1 motif, 7 (Adamts7), mRNA.
scl0001566.1_2-S	-34.9467	-8.3	Tcf7	NM_009331.2	Mus musculus transcription factor 7, T-cell specific (Tcf7), mRNA.
scl49333.35.1_32-S	-34.8684	-1.38	Eif4g1	XM_358318	Mus musculus eukaryotic translation initiation factor 4, gamma 1 (Eif4g1), mRNA.
scl070333.1_111-S	-34.676	-3.81	Ase1	NM_145822.1	
scl21972.20_222-S	-34.628	-1.51	Sema4a	NM_013658.2	Mus musculus sema domain, immunoglobulin domain (Ig), transmembrane domain (TM) and short cytoplasmic domain, (semaphorin) 4A (Sema4a), mRNA.
scl0054711.1_45-S	-34.4435	-3.83	Plagl2	NM_018807.3	Mus musculus pleiomorphic adenoma gene-like 2 (Plagl2), mRNA.
scl34303.2_339-S	-34.4226	-7.21	Chst5	NM_019950.1	Mus musculus carbohydrate (N-acetylglucosamine 6-O) sulfotransferase 5 (Chst5), mRNA.
scl33725.12_115-S	-34.2515	-1.5	Fkbp8	NM_010223.1	Mus musculus FK506 binding protein 8 (Fkbp8), mRNA.
scl32925.7_312-S	-34.212	-1.57	2310004L02Rik	NM_025504.2	Mus musculus RIKEN cDNA 2310004L02 gene (2310004L02Rik), mRNA.
scl0020678.1_3-S	-34.1316	-1.41	Sox5	NM_011444.1	Mus musculus SRY-box containing gene 5 (Sox5), mRNA.
scl000851.1_458-S	-33.7972	-9.31	Irf6	NM_178083.2	Mus musculus interferon regulatory factor 6 (Irf6), mRNA.
scl0022123.1_274-S	-33.6485	-1.35	Psmd3	NM_009439.1	Mus musculus proteasome (prosome, macropain) 26S subunit, non-ATPase, 3 (Psmd3), mRNA.
scl0001626.1_14-S	-33.6117	-8.79	AW046014	NM_174989.1	Mus musculus expressed sequence AW046014 (AW046014), mRNA.
scl0003720.1_47-S	-33.452	-1.68	F12	NM_021489.1	Mus musculus coagulation factor XII (Hageman factor) (F12), mRNA.
scl0218397.1_12-S	-33.3619	-1.4	Rasa1	NM_145452	Mus musculus RAS p21 protein activator 1 (Rasa1), mRNA.
scl0227738.4_11-S	-33.319	-7.43	Lrsam1	NM_199302.1	Mus musculus leucine rich repeat and sterile alpha motif containing 1 (Lrsam1), mRNA.
scl015013.2_70-S	-33.138	-1.33	H2-Q2	NM_010392	Mus musculus histocompatibility 2, Q region locus 2 (H2-Q2), mRNA.
scl47648.13.1_3-S	-33.1274	-3.12	AW124722	XM_139515.3	
scl34222.7_318-S	-33.0674	-1.53	Rnf166	XM_134599.3	
scl067605.5_228-S	-33.0611	-1.41	Akt1s1	NM_026270.1	Mus musculus AKT1 substrate 1 (proline-rich) (Akt1s1), mRNA.
scl37690.5.564_26-S	-33.0545	-1.47	Gna11	NM_010301.1	Mus musculus guanine nucleotide binding protein, alpha 11 (Gna11), mRNA.
scl0019377.2_133-S	-32.9198	-8.46	Rai1	NM_009021.1	Mus musculus retinoic acid induced 1 (Rai1), mRNA.
scl020443.1_125-S	-32.8137	-1.62	Siat4c	NM_009178.2	Mus musculus sialyltransferase 4C (beta-galactoside alpha-2,3-sialytransferase) (Siat4c), mRNA.
scl53012.17_160-S	-32.8047	-1.32	AI450540	NM_145505.2	Mus musculus expressed sequence AI450540 (AI450540), mRNA.
scl0231807.1_240-S	-32.7528	-1.8	BC037034	NM_153161.1	Mus musculus cDNA sequence BC037034 (BC037034), mRNA.
scl069890.1_204-S	-32.6438	-2.07	Zfp219	NM_027248	Mus musculus zinc finger protein 219 (Zfp219), mRNA.
scl0002342.1_0-S	-32.578	-6.49	G630009D10Rik	NM_177354.2	Mus musculus RIKEN cDNA G630009D10 gene (G630009D10Rik), mRNA.
scl018632.5_33-S	-32.5723	-1.33	Pex11b	NM_011069.2	Mus musculus peroxisomal biogenesis factor 11b (Pex11b), mRNA.
scl020535.23_4-S	-32.4368	-1.58	Slc4a2	NM_009207.1	Mus musculus solute carrier family 4 (anion exchanger), member 2 (Slc4a2), mRNA.
scl40867.4.1_55-S	-32.2301	-1.38	Nags	NM_178053.2	Mus musculus N-acetylglutamate synthase (Nags), mRNA.
scl026403.1_197-S	-31.9399	-1.39	Map3k11	NM_022012.2	Mus musculus mitogen activated protein kinase kinase kinase 11 (Map3k11), mRNA.
scl31790.5_79-S	-31.9356	-1.51	Fiz1	NM_011813.2	Mus musculus Flt3 interacting zinc finger protein 1 (Fiz1), mRNA.
scl34708.22.13_8-S	-31.93	-7.81	Rent1	NM_030680	Mus musculus regulator of nonsense transcripts 1 (Rent1), mRNA.
scl0116871.14_11-S	-31.8209	-1.77	Mta3	NM_054082.1	Mus musculus metastasis associated 3 (Mta3), mRNA.
scl25185.2.323_16-S	-31.7308	-1.79	Ppap2b	NM_080555.1	Mus musculus phosphatidic acid phosphatase type 2B (Ppap2b), mRNA.
scl00228770.2_82-S	-31.6403	-8.44	A930029K19Rik	NM_175468.2	Mus musculus RIKEN cDNA A930029K19 gene (A930029K19Rik), mRNA.
scl0002451.1_30-S	-31.6152	-2.91	5730502D15Rik	NM_026485.1	Mus musculus RIKEN cDNA 5730502D15 gene (5730502D15Rik), mRNA.
scl078887.5_29-S	-31.5086	-1.83	2310047I15Rik	NM_030207.1	Mus musculus RIKEN cDNA 2310047I15 gene (2310047I15Rik), mRNA.
scl013356.3_25-S	-31.4893	-1.38	Dgcr2	XM_148524.1	Mus musculus DiGeorge syndrome critical region gene 2 (Dgcr2), mRNA.
scl0026936.2_165-S	-31.388	-1.69	AA536749	NM_012027.1	Mus musculus expressed sequence AA536749 (AA536749), mRNA.
scl012153.1_23-S	-31.336	-1.36	Bmp1	NM_009755.2	Mus musculus bone morphogenetic protein 1 (Bmp1), mRNA.
scl000319.1_7-S	-31.1614	-2.19	Acin1	NM_019567.1	Mus musculus apoptotic chromatin condensation inducer 1 (Acin1), mRNA.
scl21104.8_445-S	-31.1363	-1.81	2900073H19Rik	NM_026615.2	Mus musculus RIKEN cDNA 2900073H19 gene (2900073H19Rik), mRNA.
scl50683.12_301-S	-31.1113	-1.38	Gtpbp2	NM_019581.2	Mus musculus GTP binding protein 2 (Gtpbp2), mRNA.
scl000403.1_95-S	-31.0293	-2.68	Slc39a14	NM_144808.1	Mus musculus solute carrier family 39 (zinc transporter), member 14 (Slc39a14), mRNA.
scl23133.14.29_13-S	-30.9071	-1.69	Gmps	XM_130877.3	Mus musculus guanine monphosphate synthetase (Gmps), mRNA.
scl41519.8_316-S	-30.8108	-1.35	BC003251	NM_024480.2	Mus musculus cDNA sequence BC003251 (BC003251), mRNA.
scl31668.16.1_1-S	-30.7891	-6.39	Lu	NM_020486.1	Mus musculus Lutheran blood group (Auberger b antigen included) (Lu), mRNA.
scl20482.12_3-S	-30.7239	-7.09	2410042D21Rik	NM_024254.2	Mus musculus RIKEN cDNA 2410042D21 gene (2410042D21Rik), mRNA.
scl020969.4_58-S	-30.576	-8.9	Sdc1	NM_011519.1	Mus musculus syndecan 1 (Sdc1), mRNA.
scl41352.8_186-S	-30.5223	-1.48	Dullard	NM_026017.1	Mus musculus Dullard homolog (Xenopus laevis) (Dullard), mRNA.
scl0074043.2_32-S	-30.5169	-2.47	4632428M11Rik	NM_028730.2	Mus musculus RIKEN cDNA 4632428M11 gene (4632428M11Rik), mRNA.
scl0001506.1_275-S	-30.4513	-1.83	AA536749	NM_012027.1	Mus musculus expressed sequence AA536749 (AA536749), mRNA.

scl36492.10.1_4-S	-30.3827	-1.82	Ifrd2	NM_025903.1	Mus musculus interferon-related developmental regulator 2 (Ifrd2), mRNA.
scl000532.1_17-S	-30.3517	-1.35	Yif1	NM_026553.2	Mus musculus Yip1 interacting factor homolog (S. cerevisiae) (Yif1), mRNA.
scl0018021.2_84-S	-30.2767	-1.59	Nfatc3	NM_010901.1	Mus musculus nuclear factor of activated T-cells, cytoplasmic, calcineurin-dependent 3 (Nfatc3), mRNA.
scl29861.5_188-S	-30.2693	-1.29	BC014699	NM_145570.1	Mus musculus cDNA sequence BC014699 (BC014699), mRNA.
scl29553.16.1_150-S	-30.1916	-1.35	Slc6a13	NM_144512.1	Mus musculus solute carrier family 6 (neurotransmitter transporter, GABA), member 13 (Slc6a13), mRNA.
scl0319601.1_34-S	-30.1465	-5.13	E430039K05Rik	NM_177318.2	Mus musculus RIKEN cDNA E430039K05 gene (E430039K05Rik), mRNA.
scl0067231.2_265-S	-30.0805	-1.71	2810442O16Rik	NM_024196.1	Mus musculus RIKEN cDNA 2810442O16 gene (2810442O16Rik), mRNA.
scl51338.13_19-S	-30.0137	-1.3	Nars	NM_027350.1	Mus musculus asparaginyl-tRNA synthetase (Nars), mRNA.
scl0004055.1_79-S	-29.6978	-2.92	Bcl7b	NM_009745.1	Mus musculus B-cell CLL/lymphoma 7B (Bcl7b), mRNA.
scl36111.9.1_0-S	-29.619	-1.32	Rp9h	NM_018739.1	Mus musculus retinitis pigmentosa 9 homolog (human) (Rp9h), mRNA.
scl50151.16_294-S	-29.4647	-1.55	AI429612	XM_128606.4	Mus musculus expressed sequence AI429612 (AI429612), mRNA.
scl36570.9.1_64-S	-29.4522	-1.55	Dbr1	NM_031403.1	Mus musculus debranching enzyme homolog 1 (S. cerevisiae) (Dbr1), mRNA.
scl31390.16.1_7-S	-29.4058	-1.54	2410004H02Rik	NM_145954.1	Mus musculus RIKEN cDNA 2410004H02 gene (2410004H02Rik), mRNA.
scl0071805.2_3-S	-29.2842	-2.75	Nup93	NM_172410.1	Mus musculus nucleoporin 93 (Nup93), mRNA.
scl32350.23.1_118-S	-29.1505	-1.41	2610034N24Rik	NM_027256.1	Mus musculus RIKEN cDNA 2610034N24 gene (2610034N24Rik), mRNA.
scl0209318.14_24-S	-29.137	-1.32	Gps1	NM_145370.1	Mus musculus G protein pathway suppressor 1 (Gps1), mRNA.
scl0068493.2_155-S	-29.0856	-1.39	1110007M04Rik	NM_026742.1	Mus musculus RIKEN cDNA 1110007M04 gene (1110007M04Rik), mRNA.
scl0004117.1_32-S	-29.073	-1.74	Wdr1	NM_011715.1	Mus musculus WD repeat domain 1 (Wdr1), mRNA.
scl068490.3_256-S	-29.0666	-2.13	1110003A17Rik	XM_358661.1	Mus musculus RIKEN cDNA 1110003A17 gene (1110003A17Rik), mRNA.
scl47090.1_141-S	-28.9756	-1.66	Sf3b4	NM_153053.3	Mus musculus splicing factor 3b, subunit 4 (Sf3b4), mRNA.
scl44288.11_154-S	-28.8162	-1.29	Lgals8	NM_018886.2	Mus musculus lectin, galactose binding, soluble 8 (Lgals8), mRNA.
scl33753.14_134-S	-28.7948	-1.42	Atp6v1b2	NM_007509.2	Mus musculus ATPase, H+ transporting, V1 subunit B, isoform 2 (Atp6v1b2), mRNA.
scl0001610.1_0-S	-28.741	-1.44	Decr2	NM_011933.1	Mus musculus 2-4-dienoyl-Coenzyme A reductase 2, peroxisomal (Decr2), mRNA.
scl0021339.1_180-S	-28.6953	-1.86	Taf1a	NM_021466.1	Mus musculus TATA box binding protein (Tbp)-associated factor, RNA polymerase I, A (Taf1a), mRNA.
scl0076850.1_143-S	-28.688	-6.94	Eif2c4	NM_153177.1	Mus musculus eukaryotic translation initiation factor 2C, 4 (Eif2c4), mRNA.
scl0003679.1_4-S	-28.6059	-7.84	Col4a3bp	NM_023420.1	Mus musculus procollagen, type IV, alpha 3 (Goodpasture antigen) binding protein (Col4a3bp), mRNA.
scl52453.11_571-S	-28.5547	-1.35	Keo4	NM_145502.2	Mus musculus similar to Caenorhabditis elegans protein C42C1.9 (Keo4), mRNA.
scl000151.1_308-S	-28.5339	-2.37	AW538196	NM_133952.1	Mus musculus expressed sequence AW538196 (AW538196), mRNA.
scl46998.15.1_4-S	-28.3945	-1.29	Eif3s7	NM_018749	Mus musculus eukaryotic translation initiation factor 3, subunit 7 (zeta) (Eif3s7), mRNA.
scl054403.19_44-S	-28.2927	-2.36	Slc4a4	NM_018760	Mus musculus solute carrier family 4 (anion exchanger), member 4 (Slc4a4), mRNA.
scl33072.15.1_0-S	-28.0501	-1.35	Trim28	NM_011588.1	Mus musculus tripartite motif protein 28 (Trim28), mRNA.
scl41225.47_412-S	-28.0317	-1.71	Myo18a	NM_011586.1	Mus musculus myosin XVIIIa (Myo18a), mRNA.
scl21096.14_260-S	-27.9065	-1.87	Tbc1d13	NM_146252.1	Mus musculus TBC1 domain family, member 13 (Tbc1d13), mRNA.
scl34310.8_274-S	-27.7082	-1.46	Bcar1	NM_009954.2	Mus musculus breast cancer anti-estrogen resistance 1 (Bcar1), mRNA.
scl50143.3.1_18-S	-27.6911	-1.3	0610039D01Rik	NM_026307.2	Mus musculus RIKEN cDNA 0610039D01 gene (0610039D01Rik), mRNA.
scl013806.12_49-S	-27.579	-1.51	Eno1	NM_023119	Mus musculus enolase 1, alpha non-neuron (Eno1), mRNA.
scl067118.7_5-S	-27.4055	-1.47	Bfar	XM_148088.1	Mus musculus bifunctional apoptosis regulator (Bfar), mRNA.
scl0021416.1_5-S	-27.3796	-1.49	Tcf7l2	NM_009333.2	Mus musculus transcription factor 7-like 2, T-cell specific, HMG-box (Tcf7l2), mRNA.
scl0382073.1_20-S	-27.1531	-1.99	D630044F24Rik	NM_201372.1	
scl29614.24_170-S	-27.1171	-2.16	Apg7l	NM_028835.1	Mus musculus autophagy 7-like (S. cerevisiae) (Apg7l), mRNA.
scl0016480.2_126-S	-27.1155	-1.97	Jup	NM_010593.1	Mus musculus junction plakoglobin (Jup), mRNA.
scl34655.25.1_28-S	-27.0516	-1.82	Eps15-rs	NM_007944.1	Mus musculus epidermal growth factor receptor pathway substrate 15, related sequence (Eps15-rs), mRNA.
scl0050926.1_17-S	-27.0377	-1.86	Hnrpdl	NM_016690.2	Mus musculus heterogeneous nuclear ribonucleoprotein D-like (Hnrpdl), mRNA.
scl0003011.1_11-S	-27.0199	-1.31	Agpat2	NM_026212.1	Mus musculus 1-acylglycerol-3-phosphate O-acyltransferase 2 (lysophosphatidic acid acyltransferase, beta) (Agpat2), mRNA.
scl00108989.1_82-S	-26.8274	-1.5	Tpr	NM_133780.2	Mus musculus translocated promoter region (Tpr), mRNA.
scl000196.1_9-S	-26.4598	-1.84	Actn4	NM_021895.2	Mus musculus actinin alpha 4 (Actn4), mRNA.
scl0003521.1_94-S	-26.4498	-1.53	Keap1	NM_016679.2	Mus musculus kelch-like ECH-associated protein 1 (Keap1), mRNA.
scl018641.1_29-S	-26.3063	-1.56	Pfkl	NM_008826.2	Mus musculus phosphofructokinase, liver, B-type (Pfkl), mRNA.
scl24799.82_95-S	-26.2236	-1.97	Hspg2		Mus musculus perlecan (heparan sulfate proteoglycan 2) (Hspg2), mRNA.
scl0001535.1_334-S	-26.1473	-2.35	Nags	NM_145829.1	Mus musculus N-acetylglutamate synthase (Nags), mRNA.
scl16482.8_0-S	-26.1399	-1.36	Rab17	NM_008998.2	Mus musculus RAB17, member RAS oncogene family (Rab17), mRNA.
scl0003767.1_12-S	-26.0289	-1.35	Gna11	NM_010301.1	Mus musculus guanine nucleotide binding protein, alpha 11 (Gna11), mRNA.
scl0003203.1_308-S	-25.9456	-1.63	3632413B07Rik	NM_172270.1	Mus musculus RIKEN cDNA 3632413B07 gene (3632413B07Rik), mRNA.
scl0022195.1_278-S	-25.9304	-1.3	Ube2l3	XM_148568.1	Mus musculus ubiquitin-conjugating enzyme E2L 3 (Ube2l3), mRNA.
scl39551.10.1_228-S	-25.8878	-7.65	A930006D11	NM_177790.2	Mus musculus hypothetical protein A930006D11 (A930006D11), mRNA.
scl0020848.1_188-S	-25.8082	-1.8	Stat3	NM_011486.2	Mus musculus signal transducer and activator of transcription 3 (Stat3), mRNA.

scl18457.19.17_30-S	-25.7187	-1.63	Trpc4ap	NM_019828.1	Mus musculus transient receptor potential cation channel, subfamily C, member 4 associated protein (Trpc4ap), mRNA.
scl26665.22_394-S	-25.6999	-2.73	Slc2a9	NM_145559.1	Mus musculus solute carrier family 2 (facilitated glucose transporter), member 9 (Slc2a9), mRNA.
scl0004021.1_968-S	-25.6294	-1.46	Pi4k2b	NM_025951.1	Mus musculus phosphatidylinositol 4-kinase type 2 beta (Pi4k2b), mRNA.
scl39853.10_345-S	-25.5447	-1.82	5730455P16Rik	XM_126361.2	Mus musculus RIKEN cDNA 5730455P16 gene (5730455P16Rik), mRNA.
scl0074754.1_5-S	-25.5411	-1.61	Dhcr24	NM_053272.1	Mus musculus 24-dehydrocholesterol reductase (Dhcr24), mRNA.
scl49771.23.1_24-S	-25.5162	-2.25	Dpp9	NM_172624.1	Mus musculus dipeptidylpeptidase 9 (Dpp9), mRNA.
scl21992.22_340-S	-25.23	-1.87	3110045G13Rik	NM_028460.1	Mus musculus RIKEN cDNA 3110045G13 gene (3110045G13Rik), mRNA.
scl40083.7.1_85-S	-25.1192	-1.5	Cox10	NM_178379.2	Mus musculus COX10 homolog, cytochrome c oxidase assembly protein, heme A: farnesyltransferase (yeast) (Cox10), mRNA.
scl0074525.2_276-S	-25.0991	-2.86	8430419L09Rik	NM_028982.2	Mus musculus RIKEN cDNA 8430419L09 gene (8430419L09Rik), mRNA.
scl0003447.1_0-S	-25.0163	-1.83	Rbm5	NM_148930.2	Mus musculus RNA binding motif protein 5 (Rbm5), mRNA.
scl0003920.1_1369-S	-24.954	-7.52	Rab5b	NM_177411.2	Mus musculus RAB5B, member RAS oncogene family (Rab5b), mRNA.
scl027993.9_8-S	-24.9073	-1.51	D1Wsu40e	NM_178601.2	Mus musculus DNA segment, Chr 1, Wayne State University 40, expressed (D1Wsu40e), mRNA.
scl0027886.2_220-S	-24.6803	-5.87	Es2el	XM_147209.1	Mus musculus expressed sequence 2 embryonic lethal (Es2el), mRNA.
scl0107173.1_330-S	-24.642	-1.31	AI428855	NM_207220.1	Mus musculus expressed sequence AI428855 (AI428855), mRNA.
scl016362.11_28-S	-24.6112	-1.79	Irf1	NM_008390.1	Mus musculus interferon regulatory factor 1 (Irf1), mRNA.
scl49327.15.1_14-S	-24.5112	-6.76	Ephb3	NM_010143.1	Mus musculus Eph receptor B3 (Ephb3), mRNA.
scl49605.16_306-S	-24.4905	-1.44	Prkr	NM_011163.2	Mus musculus protein kinase, interferon-inducible double stranded RNA dependent (Prkr), mRNA.
scl0192160.16_158-S	-24.4823	-1.44	Casc3	NM_138660.1	Mus musculus cancer susceptibility candidate 3 (Casc3), mRNA.
scl068708.1_138-S	-24.4776	-1.65	Rabl2a	NM_026817.1	Mus musculus RAB, member of RAS oncogene family-like 2A (Rabl2a), mRNA.
scl0001776.1_145-S	-24.4733	-1.38	Mapk1	NM_011949.2	Mus musculus mitogen activated protein kinase 1 (Mapk1), mRNA.
scl33204.17_108-S	-24.3818	-1.34	D8Ertd325e	NM_025804	Mus musculus DNA segment, Chr 8, ERATO Doi 325, expressed (D8Ertd325e), mRNA.
scl013094.10_95-S	-24.334	-151.53	Cyp2b9	NM_010000.1	Mus musculus cytochrome P450, family 2, subfamily b, polypeptide 9 (Cyp2b9), mRNA.
scl26162.14.72_6-S	-24.26	-1.47	Rnf10	NM_016698	Mus musculus ring finger protein 10 (Rnf10), mRNA.
scl0077038.2_31-S	-24.2434	-5.67	Zfp289	NM_023854.1	Mus musculus zinc finger protein 289 (Zfp289), mRNA.
scl052009.2_30-S	-24.1585	-2.35	D17Ertd441e	NM_198937.1	Mus musculus DNA segment, Chr 17, ERATO Doi 441, expressed (D17Ertd441e), mRNA.
scl00140721.2_267-S	-24.1402	-3	Caskin2	NM_080643.2	Mus musculus cask-interacting protein 2 (Caskin2), mRNA.
scl39616.9.169_52-S	-24.1143	-2.85	Nr1d1	NM_145434.1	Mus musculus nuclear receptor subfamily 1, group D, member 1 (Nr1d1), mRNA.
scl0021871.2_111-S	-24.1083	-1.41	Atp6v0a2	NM_011596.1	Mus musculus ATPase, H+ transporting, lysosomal V0 subunit a isoform 2 (Atp6v0a2), mRNA.
scl0050798.2_33-S	-24.0909	-1.32	Gne	NM_015828.2	Mus musculus glucosamine (Gne), mRNA.
scl021351.9_19-S	-24.0658	-1.23	Taldo1	NM_011528.1	Mus musculus transaldolase 1 (Taldo1), mRNA.
scl54747.23_352-S	-23.9598	-1.47	Dlgh3	NM_016747.1	Mus musculus discs, large homolog 3 (Drosophila) (Dlgh3), mRNA.
scl0011975.2_216-S	-23.9234	-1.4	Atp6v0a1	NM_016920.1	Mus musculus ATPase, H+ transporting, lysosomal V0 subunit a isoform 1 (Atp6v0a1), mRNA.
scl27067.26_61-S	-23.9172	-2.37	Unc84a	NM_024451.1	Mus musculus unc-84 homolog A (C. elegans) (Unc84a), mRNA.
scl0001633.1_3-S	-23.8688	-1.58	Ubxd1	NM_024432.2	Mus musculus UBX domain containing 1 (Ubxd1), mRNA.
scl0067123.2_83-S	-23.8517	-1.53	Ubap1	NM_023305.2	Mus musculus ubiquitin-associated protein 1 (Ubap1), mRNA.
scl0069994.1_21-S	-23.7947	-1.65	1700027M01Rik	NM_023544.2	Mus musculus RIKEN cDNA 1700027M01 gene (1700027M01Rik), mRNA.
scl43914.11.4_21-S	-23.678	-1.29	H2afy	NM_012015.1	Mus musculus H2A histone family, member Y (H2afy), mRNA.
scl0018194.1_157-S	-23.653	-6.74	Nsdhl	NM_010941.3	Mus musculus NAD(P) dependent steroid dehydrogenase-like (Nsdhl), mRNA.
scl25528.7.1_41-S	-23.5724	-2.06	Galt	NM_016658.1	Mus musculus galactose-1-phosphate uridyl transferase (Galt), mRNA.
scl34568.25_184-S	-23.563	-1.37	BC016188	NM_145970.1	Mus musculus cDNA sequence BC016188 (BC016188), mRNA.
scl0002910.1_314-S	-23.5154	-1.62	DXImx47e	NM_207670.1	Mus musculus DNA segment, Chr X, Immunex 47, expressed (DXImx47e), mRNA.
scl00381314.1_0-S	-23.5076	-1.26	2010002H18Rik	NM_198653.1	Mus musculus RIKEN cDNA 2010002H18 gene (2010002H18Rik), mRNA.
scl37657.36.1_139-S	-23.5033	-1.41	Stab2	NM_138673.1	Mus musculus stabilin 2 (Stab2), mRNA.
scl21371.8.1_29-S	-23.4736	-1.24	Rabggtb	NM_011231.1	Mus musculus RAB geranylgeranyl transferase, b subunit (Rabggtb), mRNA.
scl0054403.2_10-S	-23.4479	-4.14	Slc4a4	NM_018760.1	Mus musculus solute carrier family 4 (anion exchanger), member 4 (Slc4a4), mRNA.
scl013495.13_174-S	-23.4405	-1.38	Drg2	NM_021354.1	Mus musculus developmentally regulated GTP binding protein 2 (Drg2), mRNA.
scl027399.1_171-S	-23.4146	-1.35	Ihpk1	NM_013785.2	Mus musculus inositol hexaphosphate kinase 1 (Ihpk1), mRNA.
scl00328365.2_167-S	-23.3616	-2.87	BC065120	NM_183208.2	
scl19777.13_152-S	-23.2343	-3.47	Arfgap1	NM_145760.2	Mus musculus ADP-ribosylation factor GTPase activating protein 1 (Arfgap1), mRNA.
scl00227682.2_166-S	-23.2236	-1.28	G430055L02Rik	NM_145520.2	
scl55078.9.1_18-S	-23.2155	-2.23	Lmo6	NM_175097.2	Mus musculus LIM domain only 6 (Lmo6), mRNA.
scl51531.20.1_18-S	-23.1803	-1.47	1810057E01Rik	NM_030749.1	Mus musculus RIKEN cDNA 1810057E01 gene (1810057E01Rik), mRNA.
scl29552.17.1_293-S	-23.1737	-1.24	Slc6a12	NM_133661.1	Mus musculus solute carrier family 6 (neurotransmitter transporter, betaine/GABA), member 12 (Slc6a12), mRNA.
scl00224133.1_32-S	-23.1131	-2.32	Parp14	NM_145481.1	
scl22694.6_358-S	-23.1113	-1.41	Extl2	NM_021388.2	Mus musculus exotoses (multiple)-like 2 (Extl2), mRNA.
scl0219158.1_104-S	-23.1096	-1.52	2610301G19Rik	XM_358314.1	Mus musculus RIKEN cDNA 2610301G19 gene (2610301G19Rik), mRNA.

scl40612.43.1_21-S	-23.0988	-1.46	Tbcd		
scl00114896.1_45-S	-23.0065	-1.36	Afg3l1	NM_054070.1	Mus musculus AFG3(ATPase family gene 3)-like 1 (yeast) (Afg3l1), mRNA.
scl29713.3_34-S	-22.994	-2.14	Arl6ip5	NM_022992.1	Mus musculus ADP-ribosylation factor-like 6 interacting protein 5 (Arl6ip5), mRNA.
scl27250.2_266-S	-22.9058	-1.5	D730049H07Rik	NM_175423	Mus musculus RIKEN cDNA D730049H07 gene (D730049H07Rik), mRNA.
scl26987.8.1779_4-S	-22.8989	-4.22	Cpsf4	NM_178576.1	Mus musculus cleavage and polyadenylation specific factor 4 (Cpsf4), mRNA.
scl0399566.4_97-S	-22.8756	-1.44	Btbd6	NM_201646	Mus musculus BTB (POZ) domain containing 6 (Btbd6), mRNA.
scl0210711.2_41-S	-22.8246	-1.25	1110007A13Rik	NM_145955.2	Mus musculus RIKEN cDNA 1110007A13 gene (1110007A13Rik), mRNA.
scl24706.58_26-S	-22.7663	-1.63	Frap1	NM_020009.1	Mus musculus FK506 binding protein 12-rapamycin associated protein 1 (Frap1), mRNA.
scl0020874.2_150-S	-22.7603	-1.91	Stk2	NM_009289.1	
scl0002187.1_532-S	-22.7357	-1.32	Rab18	NM_181070.2	Mus musculus RAB18, member RAS oncogene family (Rab18), mRNA.
scl016828.6_330-S	-22.7139	-2.09	Ldh1	NM_010699.1	Mus musculus lactate dehydrogenase 1, A chain (Ldh1), mRNA.
scl50283.7.5_2-S	-22.6717	-1.27	Psmb1	NM_011185	Mus musculus proteasome (prosome, macropain) subunit, beta type 1 (Psmb1), mRNA.
scl016973.22_185-S	-22.6111	-1.72	Lrp5	NM_008513.1	Mus musculus low density lipoprotein receptor-related protein 5 (Lrp5), mRNA.
scl47650.13_510-S	-22.5798	-1.7	Ppara	NM_011144.2	Mus musculus peroxisome proliferator activated receptor alpha (Ppara), mRNA.
scl056371.1_4-S	-22.561	-2.31	Fzr1	NM_019757.1	Mus musculus fizzy/cell division cycle 20 related 1 (Drosophila) (Fzr1), mRNA.
scl27050.15.6_2-S	-22.5481	-1.52	Eif3s9	NM_133916.1	Mus musculus eukaryotic translation initiation factor 3, subunit 9 (eta) (Eif3s9), mRNA.
scl16430.4.1_256-S	-22.5311	-2.12	A930029B02Rik	NM_178779.2	
scl013089.9_98-S	-22.4761	-23.87	Cyp2b13	NM_007813.1	Mus musculus cytochrome P450, family 2, subfamily b, polypeptide 13 (Cyp2b13), mRNA.
scl0230861.11_53-S	-22.4737	-1.29	Eif4g3	NM_172703.1	Mus musculus eukaryotic translation initiation factor 4 gamma, 3 (Eif4g3), mRNA.
scl0022722.1_112-S	-22.2602	-2.53	Zfp64	NM_009564.1	Mus musculus zinc finger protein 64 (Zfp64), mRNA.
scl0050909.2_55-S	-22.1176	-1.29	C1r	NM_023143.1	Mus musculus complement component 1, r subcomponent (C1r), mRNA.
scl45535.5_307-S	-22.1081	-1.46	1810034K20Rik	NM_023397.3	Mus musculus RIKEN cDNA 1810034K20 gene (1810034K20Rik), mRNA.
scl000238.1_112-S	-22.0891	-1.28	Stard10	NM_019990.1	Mus musculus START domain containing 10 (Stard10), mRNA.
scl00276919.2_158-S	-22.0402	-1.53	Gemin4	NM_177367.2	Mus musculus gem (nuclear organelle) associated protein 4 (Gemin4), mRNA.
scl52297.3.1_24-S	-21.9703	-2.83	9430020K01Rik	XM_140198.3	
scl0021812.1_48-S	-21.9269	-1.26	Tgfbr1	NM_009370.2	Mus musculus transforming growth factor, beta receptor I (Tgfbr1), mRNA.
scl022757.8_328-S	-21.8373	-1.32	Zfp95	XM_355663.1	Mus musculus zinc finger protein 95 (Zfp95), mRNA.
scl0233208.1_10-S	-21.8352	-1.74	AI480556	XM_133474.4	Mus musculus expressed sequence AI480556 (AI480556), mRNA.
scl46892.6_503-S	-21.8168	-1.56	BC025519	XM_110101.3	Mus musculus cDNA sequence BC025519 (BC025519), mRNA.
scl52940.15.185_1-S	-21.5904	-3.52	Pprc1	XM_359412.1	
scl52406.11.1_60-S	-21.5903	-1.29	Actr1a	NM_016860.1	Mus musculus ARP1 actin-related protein 1 homolog A (yeast) (Actr1a), mRNA.
scl0105440.12_47-S	-21.5889	-2.67	Kctd9	NM_134073.1	Mus musculus potassium channel tetramerisation domain containing 9 (Kctd9), mRNA.
scl37395.7.349_6-S	-21.5127	-2	Dtx3	NM_030714.1	Mus musculus deltex 3 homolog (Drosophila) (Dtx3), mRNA.
scl31727.20.1_54-S	-21.4667	-3.14	Dhx34	NM_027883.1	Mus musculus DEAH (Asp-Glu-Ala-His) box polypeptide 34 (Dhx34), mRNA.
scl028019.8_226-S	-21.4474	-2.61	Ing4	NM_133345.1	Mus musculus inhibitor of growth family, member 4 (Ing4), mRNA.
scl0002072.1_701-S	-21.4058	-3.44	Nr1h5	NM_198658.1	Mus musculus nuclear receptor subfamily 1, group H, member 5 (Nr1h5), transcript variant 1, mRNA.
scl0235169.2_28-S	-21.3892	-1.77	BC024806	NM_172291.1	Mus musculus cDNA sequence BC024806 (BC024806), mRNA.
scl000224.1_0-S	-21.3139	-2.61	Sult2b1	NM_017465.1	Mus musculus sulfotransferase family, cytosolic, 2B, member 1 (Sult2b1), mRNA.
scl0075751.2_140-S	-21.3138	-1.52	Ipo4	NM_024267.4	Mus musculus importin 4 (Ipo4), mRNA.
scl0003167.1_1-S	-21.2658	-2.67	Il15ra	NM_133836.1	Mus musculus interleukin 15 receptor, alpha chain (Il15ra), mRNA.
scl28154.19_171-S	-21.2378	-1.37	Dmtf1	NM_011806.2	Mus musculus cyclin D binding myb-like transcription factor 1 (Dmtf1), mRNA.
scl0276846.12_121-S	-21.2303	-2.67	Pigs	NM_201406.1	Mus musculus phosphatidylinositol glycan, class S (Pigs), mRNA.
scl0066052.1_175-S	-21.2109	-1.23	Sdhc	NM_025321.1	Mus musculus succinate dehydrogenase complex, subunit C, integral membrane protein (Sdhc), mRNA.
scl053607.7_2-S	-21.1765	-2.02	Snrpa	NM_015782.2	Mus musculus small nuclear ribonucleoprotein polypeptide A (Snrpa), mRNA.
scl46991.17.2_22-S	-21.1316	-1.49	Tmprss6	NM_027902.1	Mus musculus transmembrane serine protease 6 (Tmprss6), mRNA.
scl0076142.1_0-S	-21.0366	-4.08	Ppp1r14c	NM_133485.1	Mus musculus protein phosphatase 1, regulatory (inhibitor) subunit 14c (Ppp1r14c), mRNA.
scl067581.1_259-S	-21.0189	-1.39	4930451A13Rik	NM_026254.2	Mus musculus RIKEN cDNA 4930451A13 gene (4930451A13Rik), mRNA.
scl25881.20.1_16-S	-21.001	-1.67	Ars2	NM_031405.1	Mus musculus arsenate resistance protein 2 (Ars2), mRNA.
scl0019336.1_63-S	-20.9564	-1.33	Rab24	NM_009000.2	Mus musculus RAB24, member RAS oncogene family (Rab24), mRNA.
scl056628.1_171-S	-20.9399	-1.23	LOC56628		
scl000671.1_1-S	-20.922	-1.49	Ryk	NM_013649	Mus musculus receptor-like tyrosine kinase (Ryk), mRNA.
scl072106.9_198-S	-20.8978	-1.32	2610003J06Rik	NM_028101.1	Mus musculus RIKEN cDNA 2610003J06 gene (2610003J06Rik), mRNA.
scl43064.12_579-S	-20.8665	-1.73	Fntb	NM_145927.1	Mus musculus farnesyltransferase, CAAX box, beta (Fntb), mRNA.
scl51509.11.1_166-S	-20.8456	-2.45	Apbb3	NM_146085.1	Mus musculus amyloid beta (A4) precursor protein-binding, family B, member 3 (Apbb3), mRNA.
scl0068695.1_200-S	-20.8091	-1.36	1110033O09Rik	NM_026812.1	Mus musculus RIKEN cDNA 1110033O09 gene (1110033O09Rik), mRNA.
scl38697.44.1_55-S	-20.7754	-3.2	Abca7	NM_013850.1	Mus musculus ATP-binding cassette, sub-family A (ABC1), member 7 (Abca7), mRNA.

scl0055989.2_71-S	-20.7535	-1.46	Nol5	NM_018868.1	Mus musculus nucleolar protein 5 (Nol5), mRNA.
scl0353371.2_330-S	-20.7507	-2.56	Oxct2b	NM_181859.1	Mus musculus 3-oxoacid CoA transferase 2B (Oxct2b), mRNA.
scl39227.8.1_2-S	-20.7348	-2.55	Pycr1	NM_144795.1	Mus musculus pyrroline-5-carboxylate reductase 1 (Pycr1), mRNA.
scl44724.21.5_2-S	-20.7315	-1.66	Ddx46	NM_145975.1	Mus musculus DEAD (Asp-Glu-Ala-Asp) box polypeptide 46 (Ddx46), mRNA.
scl48775.5_265-S	-20.656	-1.71	Emp2	NM_007929.1	Mus musculus epithelial membrane protein 2 (Emp2), mRNA.
scl094093.7_13-S	-20.6516	-3.78	Trim33	NM_053170.1	Mus musculus tripartite motif protein 33 (Trim33), mRNA.
scl0234385.1_330-S	-20.6456	-1.58	Mast3	NM_199308.1	
scl00228602.2_5-S	-20.6015	-1.69	4930402H24Rik	NM_029432.1	Mus musculus RIKEN cDNA 4930402H24 gene (4930402H24Rik), mRNA.
scl23927.19.1_34-S	-20.5666	-1.36	Ipo13	NM_146152.2	Mus musculus importin 13 (Ipo13), mRNA.
scl021937.10_167-S	-20.5334	-1.51	Tnfrsf1a	NM_011609.2	Mus musculus tumor necrosis factor receptor superfamily, member 1a (Tnfrsf1a), mRNA.
scl20266.8_58-S	-20.48	-1.52	D430028G21Rik	NM_144888.1	Mus musculus RIKEN cDNA D430028G21 gene (D430028G21Rik), mRNA.
scl0065970.2_260-S	-20.4688	-1.49	D15Ertd366e	NM_023063.1	Mus musculus DNA segment, Chr 15, ERATO Doi 366, expressed (D15Ertd366e), mRNA.
scl066812.1_16-S	-20.4681	-1.53	8430432M10Rik	NM_176831.2	Mus musculus RIKEN cDNA 8430432M10 gene (8430432M10Rik), mRNA.
scl46720.13_574-S	-20.4648	-5.93	Pou6f1	NM_010127.2	Mus musculus POU domain, class 6, transcription factor 1 (Pou6f1), mRNA.
scl011898.16_99-S	-20.435	-1.31	Ass1	NM_007494.2	Mus musculus argininosuccinate synthetase 1 (Ass1), mRNA.
scl0170826.1_122-S	-20.4124	-2.39	Ppargc1b	NM_133249	Mus musculus peroxisome proliferative activated receptor, gamma, coactivator 1 beta (Ppargc1b), mRNA.
scl27387.5.1_3-S	-20.3974	-4.45	Mvk	NM_023556.2	Mus musculus mevalonate kinase (Mvk), mRNA.
scl00224727.2_315-S	-20.3613	-1.28	Bat3	NM_057171.1	Mus musculus HLA-B-associated transcript 3 (Bat3), mRNA.
scl44632.16.1_30-S	-20.2201	-1.22	C130052I12Rik	NM_146047.1	Mus musculus RIKEN cDNA C130052I12 gene (C130052I12Rik), mRNA.
scl49447.9_394-S	-20.1919	-1.4	Pmm2	XM_147219.1	Mus musculus phosphomannomutase 2 (Pmm2), mRNA.
scl0050771.2_184-S	-20.1489	-1.65	Atp9b	NM_015805.2	Mus musculus ATPas, class II, type 9B (Atp9b), mRNA.
scl074754.9_24-S	-20.1472	-1.5	Dhcr24	NM_053272.1	Mus musculus 24-dehydrocholesterol reductase (Dhcr24), mRNA.
scl0012334.2_163-S	-20.1347	-1.28	Capn2	NM_009794.1	Mus musculus calpain 2 (Capn2), mRNA.
scl17296.11_403-S	-20.0497	-2.02	Vamp4	NM_016796.2	Mus musculus vesicle-associated membrane protein 4 (Vamp4), mRNA.
scl44775.16.1_15-S	-20.0356	-1.35	Secisbp2	XM_127336.2	

TABLE A2

GENMAPPS AND GENE ONTOLOGIES SIGNIFICANTLY CHANGED BY THE ATHEROGENIC DIET IN LIVERS

UP-REGULATED - GENMAPP

No.	MAPP Name	Number Changed	Number Measured	Number on MAPP	Percent Changed	Percent Present	Z Score	Permuted P Value
1	Mm_Ribosomal_Proteins	55	78	80	70.5128	97.5000	11.6880	0.0000
2	Mm_Electron_Transport_Chain	41	66	83	62.1212	79.5181	8.9860	0.0000
3	Mm_Translation_Factors	25	49	50	51.0204	98.0000	5.7370	0.0000
4	Mm_Proteasome_Degradation	32	74	81	43.2432	91.3580	5.3520	0.0000
5	Mm_Complement_and_Coagulation_Cascades_KEGG	25	60	62	41.6667	96.7742	4.4980	0.0000
6	Mm_mRNA_processing_binding_Reactome	115	427	551	26.9321	77.4955	4.3810	0.0000
7	Mm_Citrate_cycle_TCA_cycle_	9	14	27	64.2857	51.8519	4.3200	0.0000
8	Mm_TNF-alpha-NF-kB_NetPath_9	48	167	176	28.7425	94.8864	3.2610	0.0000
9	Mm_Fatty_Acid_Beta_Oxidation_Meta_BiGCaT	14	31	32	45.1613	96.8750	3.7190	0.0010
10	Mm_Fatty_acid_metabolism	20	51	66	39.2157	77.2727	3.6940	0.0010
11	Mm_Valine_leucine_and_isoleucine_degradation	12	27	44	44.4444	61.3636	3.3740	0.0020
12	Mm_Mitochondrial_fatty_acid_betaoxidation	9	16	16	56.2500	100.0000	3.7990	0.0030
13	Mm_Reductive_carboxylate_cycle_CO2_fixation_	4	5	15	80.0000	33.3333	3.4750	0.0030
14	Mm_Butanoate_metabolism	11	26	69	42.3077	37.6812	3.0330	0.0030
15	Mm_Complement_Activation_Classical	8	15	16	53.3333	93.7500	3.3900	0.0060
16	Mm_Propanoate_metabolism	7	14	52	50.0000	26.9231	2.9560	0.0060
17	Mm_2-Tissues-Internal_Organs	50	187	202	26.7380	92.5743	2.7450	0.0060
18	Mm_Fatty_Acid_Beta_Oxidation_1_BiGCaT	11	26	27	42.3077	96.2963	3.0330	0.0070
19	Mm_Lysine_degradation	13	34	69	38.2353	49.2754	2.8640	0.0070
20	Mm_Irinotecan_pathway_PharmGKB	6	10	12	60.0000	83.3333	3.3040	0.0080

UP-REGULATED - GENE ONTOLOGY

No.	GO Name	GO Type	Number Changed	Number Measured	Number in GO	Percent Changed	Percent Present	Z Score	Permuted P Value
1	biosynthesis	P	313	1154	1428	27.1231	80.8123	13.1310	0.0000
2	metabolism	P	1222	6721	7938	18.1818	84.6687	12.7510	0.0000
3	cellular physiological process	P	1513	8935	10403	16.9334	85.8887	11.8860	0.0000
4	generation of precursor metabolites and energy	P	179	598	722	29.9331	82.8255	11.2760	0.0000
5	protein biosynthesis	P	169	588	801	28.7415	73.4082	10.3320	0.0000
6	physiological process	P	1606	9962	11632	16.1213	85.6431	9.7540	0.0000
7	protein metabolism	P	566	2850	3423	19.8597	83.2603	9.6810	0.0000
8	electron transport	P	114	386	461	29.5337	83.7310	8.7640	0.0000
9	mitochondrial electron transport\, NADH to ubiquinone	P	12	14	25	85.7143	56.0000	7.6770	0.0000
10	ubiquitin cycle	P	114	438	511	26.0274	85.7143	7.2170	0.0000
11	ubiquitin-dependent protein catabolism	P	46	132	149	34.8485	88.5906	6.8390	0.0000
12	transport	P	469	2545	2884	18.4283	88.2455	6.7580	0.0000
13	ATP synthesis coupled proton transport	P	22	45	60	48.8889	75.0000	6.6850	0.0000
14	protein transport	P	139	590	654	23.5593	90.2141	6.6680	0.0000
15	ATP biosynthesis	P	23	49	64	46.9388	76.5625	6.5850	0.0000
16	protein localization	P	148	645	714	22.9457	90.3361	6.5280	0.0000
17	intracellular protein transport	P	104	416	463	25.0000	89.8488	6.4190	0.0000
18	intracellular transport	P	146	650	740	22.4615	87.8378	6.1930	0.0000
19	nucleotide metabolism	P	57	194	236	29.3814	82.2034	6.1110	0.0000
20	purine nucleotide biosynthesis	P	29	75	96	38.6667	78.1250	6.0950	0.0000
21	protein folding	P	58	200	227	29.0000	88.1057	6.0500	0.0000
22	protein catabolism	P	56	192	215	29.1667	89.3023	5.9930	0.0000
23	lipid metabolism	P	130	581	616	22.3752	94.3182	5.7800	0.0000
24	translational initiation	P	23	58	72	39.6552	80.5556	5.5730	0.0000
25	fatty acid metabolism	P	46	156	164	29.4872	95.1220	5.5110	0.0000
26	complement activation\, alternative pathway	P	7	9	10	77.7778	90.0000	5.4710	0.0000
27	aromatic compound metabolism	P	33	101	108	32.6733	93.5185	5.3470	0.0000
28	protein targeting	P	60	228	261	26.3158	87.3563	5.2950	0.0000
29	RNA processing	P	79	329	371	24.0122	88.6792	5.1720	0.0000
30	main pathways of carbohydrate metabolism	P	32	100	132	32.0000	75.7576	5.1270	0.0000
31	regulation of translation	P	25	71	76	35.2113	93.4211	5.0930	0.0000
32	nucleoside metabolism	P	13	27	44	48.1482	61.3636	5.0650	0.0000
33	tricarboxylic acid cycle	P	12	24	24	50.0000	100.0000	5.0350	0.0000
34	nucleotide biosynthesis	P	38	129	155	29.4574	83.2258	4.9970	0.0000
35	RNA splicing	P	41	144	167	28.4722	86.2276	4.9420	0.0000
36	proton transport	P	25	74	92	33.7838	80.4348	4.8470	0.0000
37	mRNA metabolism	P	51	196	223	26.0204	87.8924	4.7850	0.0000
38	RNA metabolism	P	91	412	465	22.0874	88.6022	4.6680	0.0000
39	lipid transport	P	23	68	76	33.8235	89.4737	4.6550	0.0000
40	gluconeogenesis	P	10	20	20	50.0000	100.0000	4.5960	0.0000
41	regulation of translational initiation	P	13	30	30	43.3333	100.0000	4.5830	0.0000
42	nuclear mRNA splicing\, via spliceosome	P	34	118	135	28.8136	87.4074	4.5760	0.0000
43	acetyl-CoA metabolism	P	14	34	34	41.1765	100.0000	4.5180	0.0000
44	mRNA processing	P	45	173	198	26.0116	87.3737	4.4890	0.0000
45	energy derivation by oxidation of organic compounds	P	40	150	182	26.6667	82.4176	4.4070	0.0000
46	nitric oxide biosynthesis	P	9	18	19	50.0000	94.7368	4.3600	0.0000
47	response to oxidative stress	P	20	59	63	33.8983	93.6508	4.3510	0.0000
48	antigen presentation\, exogenous peptide antigen	P	4	5	6	80.0000	83.3333	4.2200	0.0000
49	copper ion homeostasis	P	4	5	5	80.0000	100.0000	4.2200	0.0000
50	response to unfolded protein	P	16	46	55	34.7826	83.6364	4.0130	0.0000
51	peroxisome organization and biogenesis	P	8	17	18	47.0588	94.4444	3.8890	0.0000
52	cell redox homeostasis	P	7	14	16	50.0000	87.5000	3.8440	0.0000
53	amino acid and derivative metabolism	P	62	292	325	21.2329	89.8462	3.4910	0.0000
54	amino acid metabolism	P	53	245	273	21.6327	89.7436	3.3730	0.0000
55	nitrogen compound metabolism	P	76	383	421	19.8433	90.9739	3.2210	0.0000
56	mitochondrial fusion	P	3	3	3	100.0000	100.0000	4.2620	0.0010
57	deoxyribonucleotide catabolism	P	4	5	5	80.0000	100.0000	4.2200	0.0010
58	ER to Golgi vesicle-mediated transport	P	18	53	59	33.9623	89.8305	4.1370	0.0010
59	response to reactive oxygen species	P	8	16	16	50.0000	100.0000	4.1100	0.0010
60	aromatic amino acid family metabolism	P	10	23	23	43.4783	100.0000	4.0320	0.0010
61	protein ubiquitination	P	36	147	173	24.4898	84.9711	3.6020	0.0010

62	secretory pathway	P	42	179	194	23.4637	92.2680	3.5840	0.0010
63	rRNA processing	P	19	65	70	29.2308	92.8571	3.4870	0.0010
64	vesicle-mediated transport	P	74	379	406	19.5251	93.3498	3.0240	0.0010
65	cotranslational protein targeting to membrane	P	7	12	13	58.3333	92.3077	4.3870	0.0020
66	spliceosome assembly	P	10	25	30	40.0000	83.3333	3.7040	0.0020
67	fatty acid biosynthesis	P	16	54	57	29.6296	94.7368	3.2610	0.0030
68	glucose metabolism	P	25	100	135	25.0000	74.0741	3.1130	0.0030
69	antigen presentation	P	15	52	63	28.8462	82.5397	3.0380	0.0030
70	glycerol metabolism	P	7	15	19	46.6667	78.9474	3.6090	0.0040
71	aldehyde metabolism	P	5	9	10	55.5556	90.0000	3.5600	0.0040
72	ribosome biogenesis	P	21	79	84	26.5823	94.0476	3.1690	0.0040
73	steroid metabolism	P	32	136	142	23.5294	95.7747	3.1410	0.0040
74	amino acid biosynthesis	P	13	42	46	30.9524	91.3043	3.1210	0.0040
75	protein import into nucleus	P	18	66	72	27.2727	91.6667	3.0570	0.0040
76	mitochondrial electron transport\, ubiquinol to cytochrome c	P	3	3	5	100.0000	60.0000	4.2620	0.0050
77	signal peptide processing	P	4	5	7	80.0000	71.4286	4.2200	0.0050
78	pyrimidine nucleoside metabolism	P	5	9	24	55.5556	37.5000	3.5600	0.0050
79	translational elongation	P	9	23	28	39.1304	82.1429	3.4330	0.0050
80	antigen presentation\, exogenous antigen via MHC class II	P	5	10	12	50.0000	83.3333	3.2490	0.0050
81	protein modification	P	245	1481	1665	16.5429	88.9490	2.7510	0.0050
82	protein targeting to mitochondrion	P	7	16	21	43.7500	76.1905	3.3930	0.0060
83	triacylglycerol metabolism	P	6	14	14	42.8571	100.0000	3.0780	0.0060
84	iron ion homeostasis	P	9	26	34	34.6154	76.4706	2.9900	0.0060
85	proteolysis	P	116	651	757	17.8187	85.9974	2.7240	0.0060
86	translational termination	P	4	6	6	66.6667	100.0000	3.6870	0.0070
87	fatty acid oxidation	P	10	32	32	31.2500	100.0000	2.7720	0.0070
88	complement activation\, classical pathway	P	8	22	25	36.3636	88.0000	2.9850	0.0080
89	DNA catabolism	P	5	11	12	45.4545	91.6667	2.9750	0.0080
90	glycerol-3-phosphate metabolism	P	4	6	8	66.6667	75.0000	3.6870	0.0090
91	positive regulation of nitric oxide biosynthesis	P	3	4	4	75.0000	100.0000	3.4880	0.0090
92	L-phenylalanine catabolism	P	4	7	7	57.1429	100.0000	3.2600	0.0090
93	cytolysis	P	7	19	20	36.8421	95.0000	2.8340	0.0090
94	structural constituent of ribosome	F	93	183	354	50.8197	51.6949	14.2990	0.0000
95	oxidoreductase activity	F	198	663	775	29.8643	85.5484	11.8480	0.0000
96	catalytic activity	F	871	4696	5420	18.5477	86.6421	10.3970	0.0000
97	NADH dehydrogenase (ubiquinone) activity	F	22	27	39	81.4815	69.2308	10.0350	0.0000
98	NADH dehydrogenase activity	F	23	31	43	74.1936	72.0930	9.5890	0.0000
99	electron carrier activity	F	36	67	80	53.7313	83.7500	9.3030	0.0000
100	RNA binding	F	132	466	548	28.3262	85.0365	8.8970	0.0000
101	hydrogen ion transporter activity	F	42	104	130	40.3846	80.0000	7.6890	0.0000
102	rRNA binding	F	12	15	18	80.0000	83.3333	7.3120	0.0000
103	cytochrome-c oxidase activity	F	14	21	27	66.6667	77.7778	6.9000	0.0000
104	hydrogen-transporting ATPase activity\, rotational mechanism	F	22	45	58	48.8889	77.5862	6.6850	0.0000
105	translation regulator activity	F	44	127	151	34.6457	84.1060	6.6410	0.0000
106	hydrogen-transporting ATP synthase activity\, rotational mechanism	F	21	43	56	48.8372	76.7857	6.5250	0.0000
107	threonine endopeptidase activity	F	12	18	21	66.6667	85.7143	6.3880	0.0000
108	unfolded protein binding	F	47	146	159	32.1918	91.8239	6.2710	0.0000
109	oxidoreductase activity\, acting on the CH-OH group of donors\, NAD or NADP as acceptor	F	34	95	108	35.7895	87.9630	6.0580	0.0000
110	iron ion binding	F	68	250	293	27.2000	85.3242	5.9530	0.0000
111	hydrolase activity	F	354	1931	2234	18.3325	86.4369	5.6140	0.0000
112	translation initiation factor activity	F	25	66	86	37.8788	76.7442	5.5320	0.0000
113	ubiquitin conjugating enzyme activity	F	14	33	38	42.4242	86.8421	4.6570	0.0000
114	isomerase activity	F	38	136	153	27.9412	88.8889	4.6230	0.0000
115	electron transporter activity	F	46	175	195	26.2857	89.7436	4.6190	0.0000
116	iron-sulfur cluster binding	F	11	24	25	45.8333	96.0000	4.4490	0.0000
117	ATPase activity	F	71	314	360	22.6115	87.2222	4.3310	0.0000
118	ATPase activity\, coupled to transmembrane movement of substances	F	36	134	158	26.8657	84.8101	4.2300	0.0000
119	structural molecule activity	F	126	633	893	19.9052	70.8847	4.2230	0.0000
120	hydrolase activity\, acting on acid anhydrides\, catalyzing transmembrane movement of substances	F	36	136	161	26.4706	84.4721	4.1290	0.0000
121	NAD binding	F	12	30	53	40.0000	56.6038	4.0590	0.0000
122	nucleoside-triphosphatase activity	F	102	500	565	20.4000	88.4956	4.0580	0.0000
123	ubiquitin-protein ligase activity	F	51	219	259	23.2877	84.5560	3.8940	0.0000
124	ATPase activity\, coupled	F	58	267	301	21.7229	88.7043	3.5670	0.0000
125	lipid transporter activity	F	18	60	65	30.0000	92.3077	3.5210	0.0000
126	nitric-oxide synthase regulator activity	F	5	5	6	100.0000	83.3333	5.5020	0.0010
127	RNA splicing factor activity\, transesterification mechanism	F	9	20	23	45.0000	86.9565	3.9540	0.0010
128	ligase activity	F	79	382	449	20.6806	85.0780	3.6920	0.0010
129	protein serine/threonine phosphatase activity	F	12	33	36	36.3636	91.6667	3.6580	0.0010
130	hydrolase activity\, acting on ester bonds	F	111	583	680	19.0395	85.7353	3.4340	0.0010
131	arylesterase activity	F	3	3	3	100.0000	100.0000	4.2620	0.0020
132	protein transporter activity	F	31	120	132	25.8333	90.9091	3.6750	0.0020
133	magnesium ion binding	F	54	254	273	21.2598	93.0403	3.2640	0.0020
134	S-adenosylmethionine-dependent methyltransferase activity	F	20	78	87	25.6410	89.6552	2.9100	0.0020
135	SUMO conjugating enzyme activity	F	3	3	6	100.0000	50.0000	4.2620	0.0030
136	translation release factor activity\, codon specific	F	3	3	3	100.0000	100.0000	4.2620	0.0030
137	ubiquinol-cytochrome-c reductase activity	F	5	8	11	62.5000	72.7273	3.9190	0.0030
138	acyl-CoA dehydrogenase activity	F	7	16	16	43.7500	100.0000	3.3930	0.0030
139	protein-L-isoaspartate (D-aspartate) O-methyltransferase activity	F	3	3	3	100.0000	100.0000	4.2620	0.0040
140	signal sequence binding	F	7	17	17	41.1765	100.0000	3.1930	0.0040
141	selenium binding	F	9	25	27	36.0000	92.5926	3.1310	0.0040
142	protein phosphatase type 1 activity	F	5	9	10	55.5556	90.0000	3.5600	0.0050
143	voltage-gated ion-selective channel activity	F	3	4	5	75.0000	80.0000	3.4880	0.0070
144	microtubule binding	F	12	42	44	28.5714	95.4545	2.6780	0.0070
145	monooxygenase activity	F	24	105	128	22.8571	82.0313	2.5590	0.0070
146	nuclear localization sequence binding	F	4	7	7	57.1429	100.0000	3.2600	0.0080
147	glutathione transferase activity	F	8	21	26	38.0952	80.7692	3.1440	0.0080
148	MHC class II receptor activity	F	4	7	11	57.1429	63.6364	3.2600	0.0090
149	ubiquitin binding	F	4	8	9	50.0000	88.8889	2.9050	0.0090
150	cytoplasm	C	863	3488	3969	24.7420	87.8811	20.4670	0.0000
151	mitochondrion	C	279	730	822	38.2192	88.8078	19.1000	0.0000

152	intracellular	C	1299	6945	8118	18.7041	85.5506	14.8610	0.0000
153	ribonucleoprotein complex	C	143	355	550	40.2817	64.5455	14.2720	0.0000
154	ribosome	C	90	176	345	51.1364	51.0145	14.1410	0.0000
155	protein complex	C	435	1754	2202	24.8005	79.6549	13.5870	0.0000
156	mitochondrial envelope	C	117	280	329	41.7857	85.1064	13.3710	0.0000
157	mitochondrial inner membrane	C	100	232	275	43.1035	84.3636	12.7310	0.0000
158	organelle inner membrane	C	104	250	294	41.6000	85.0340	12.5360	0.0000
159	cytosol	C	111	345	383	32.1739	90.0783	9.6970	0.0000
160	small ribosomal subunit	C	23	32	45	71.8750	71.1111	9.3670	0.0000
161	proteasome complex (sensu Eukaryota)	C	25	38	42	65.7895	90.4762	9.1330	0.0000
162	endoplasmic reticulum	C	137	529	561	25.8979	94.2959	7.8700	0.0000
163	cytosolic small ribosomal subunit (sensu Eukaryota)	C	12	14	16	85.7143	87.5000	7.6770	0.0000
164	mitochondrial matrix	C	32	74	76	43.2432	97.3684	7.1860	0.0000
165	large ribosomal subunit	C	17	29	42	58.6207	69.0476	6.8680	0.0000
166	proton-transporting two-sector ATPase complex	C	23	47	61	48.9362	77.0492	6.8420	0.0000
167	mitochondrial ribosome	C	19	35	37	54.2857	94.5946	6.8100	0.0000
168	mitochondrial electron transport chain	C	17	30	40	56.6667	75.0000	6.6780	0.0000
169	cytosolic large ribosomal subunit (sensu Eukaryota)	C	10	13	18	76.9231	72.2222	6.4880	0.0000
170	microsome	C	46	138	148	33.3333	93.2432	6.4810	0.0000
171	proteasome core complex (sensu Eukaryota)	C	11	18	21	61.1111	85.7143	5.7120	0.0000
172	peroxisome	C	26	68	71	38.2353	95.7747	5.7000	0.0000
173	proton-transporting ATP synthase complex\, coupling factor F(o)	C	6	8	13	75.0000	61.5385	4.9330	0.0000
174	respiratory chain complex I (sensu Eukaryota)	C	6	8	14	75.0000	57.1429	4.9330	0.0000
175	membrane attack complex	C	4	4	6	100.0000	66.6667	4.9210	0.0000
176	endoplasmic reticulum membrane	C	32	104	109	30.7692	95.4128	4.8680	0.0000
177	peroxisomal membrane	C	10	21	22	47.6191	95.4545	4.3960	0.0000
178	eukaryotic translation initiation factor 3 complex	C	4	5	5	80.0000	100.0000	4.2200	0.0000
179	membrane fraction	C	117	588	630	19.8980	93.3333	4.0580	0.0000
180	vacuole	C	34	141	148	24.1135	95.2703	3.3990	0.0000
181	signal recognition particle (sensu Eukaryota)	C	6	10	12	60.0000	83.3333	4.1550	0.0010
182	mitochondrial intermembrane space	C	5	8	14	62.5000	57.1429	3.9190	0.0010
183	integral to endoplasmic reticulum membrane	C	12	34	35	35.2941	97.1429	3.5340	0.0010
184	ubiquitin ligase complex	C	36	152	182	23.6842	83.5165	3.3770	0.0010
185	mitochondrial outer membrane	C	11	30	33	36.6667	90.9091	3.5350	0.0020
186	mitochondrial small ribosomal subunit	C	6	9	9	66.6667	100.0000	4.5160	0.0030
187	clathrin vesicle coat	C	12	36	40	33.3333	90.0000	3.2990	0.0030
188	proteasome regulatory particle (sensu Eukaryota)	C	4	6	7	66.6667	85.7143	3.6870	0.0040
189	Golgi apparatus	C	82	436	471	18.8073	92.5690	2.8130	0.0040
190	proton-transporting ATP synthase complex (sensu Eukaryota)	C	5	8	10	62.5000	80.0000	3.9190	0.0050
191	proton-transporting ATP synthase complex\, catalytic core F(1)	C	4	6	6	66.6667	100.0000	3.6870	0.0050
192	histone deacetylase complex	C	8	22	26	36.3636	84.6154	2.9850	0.0050
193	nucleolus	C	29	133	147	21.8045	90.4762	2.5330	0.0080

DOWN-REGULATED - GENMAPP

No.	MAPP Name	Number Changed	Number Measured	Number on MAPP	Percent Changed	Percent Present	Z Score	Permuted P Value
1	Mm_Cholesterol_Biosynthesis	4	15	15	26.6667	100.0000	5.2800	0.0010
2	Mm_Translation_Factors	7	49	50	14.2857	98.0000	4.5490	0.0020
3	Mm_Urea_cycle_and_metabolism_of_amino_groups_KEGG	4	19	20	21.0526	95.0000	4.5300	0.0030
4	Mm_Nucleotide_sugars_metabolism	2	5	33	40.0000	15.1515	4.7680	0.0050

DOWN-REGULATED - GENE ONTOLOGY

No.	GO Name	GO Type	Number Changed	Number Measured	Number in GO	Percent Changed	Percent Present	Z Score	Permuted P Value
1	RNA processing	P	23	329	371	6.9909	88.6792	4.9880	0.0000
2	metabolism	P	225	6719	7938	3.3487	84.6435	4.9470	0.0000
3	biosynthesis	P	51	1154	1428	4.4194	80.8123	3.9410	0.0000
4	proteolysis	P	30	651	757	4.6083	85.9974	3.2150	0.0030
5	rRNA processing	P	6	65	70	9.2308	92.8571	3.3270	0.0050
6	regulation of translational initiation	P	4	30	30	13.3333	100.0000	3.6620	0.0070
7	protein transport	P	26	590	654	4.4068	90.2141	2.7420	0.0080
8	amino acid metabolism	P	13	245	273	5.3061	89.7436	2.6320	0.0080
9	binding	F	280	9121	10565	3.0698	86.3322	4.1930	0.0000
10	nucleotide binding	F	72	1754	1959	4.1049	89.5355	4.0940	0.0000
11	zinc ion binding	F	62	1560	1866	3.9744	83.6013	3.4920	0.0000
12	RNA binding	F	23	466	548	4.9356	85.0365	3.1500	0.0000
13	peptide antigen binding	F	3	12	12	25.0000	100.0000	4.8390	0.0010
14	catalytic activity	F	153	4696	5420	3.2581	86.6421	3.2270	0.0010
15	gamma-aminobutyric acid\:sodium symporter activity	F	2	4	4	50.0000	100.0000	5.9150	0.0030
16	ATP binding	F	45	1184	1331	3.8007	88.9557	2.6110	0.0070
17	thyroid hormone receptor binding	F	3	17	18	17.6471	94.4444	3.8660	0.0080
18	steroid hormone receptor activity	F	5	49	50	10.2041	98.0000	3.3130	0.0090
19	intracellular	C	232	6943	8118	3.3415	85.5260	5.0490	0.0000
20	cytoplasm	C	127	3488	3969	3.6411	87.8811	4.2440	0.0000
21	nucleolus	C	10	133	147	7.5188	90.4762	3.5320	0.0010
22	protein complex	C	67	1752	2202	3.8242	79.5640	3.3100	0.0010
23	nucleus	C	112	3283	3798	3.4115	86.4402	3.1490	0.0030
24	RNA polymerase I transcription factor complex	C	2	5	5	40.0000	100.0000	5.2170	0.0040

TABLE A3

GENES UP-REGULATED BY THE ATHEROGENIC DIET IN SPLEENS

TargetID	Diff_Score	Fold_Change	Symbol	Accession	Definition
scl21429.6_290-S	180.1715	2.48	Lmo4	NM_010723.2	Mus musculus LIM domain only 4 (Lmo4), mRNA.
scl24880.7_98-S	144.5229	1.99	Sfrs4	NM_020587.1	Mus musculus splicing factor, arginine/serine-rich 4 (SRp75) (Sfrs4), mRNA.
scl24261.12.1_29-S	136.2332	1.88	Alad	NM_008525.3	Mus musculus aminolevulinate, delta-, dehydratase (Alad), mRNA.
scl43920.5.585_30-S	127.4582	2.24	Dok3	NM_013739.1	
scl28709.9_141-S	126.4749	2.11	Abtb1	NM_030251.1	Mus musculus ankyrin repeat and BTB (POZ) domain containing 1 (Abtb1), mRNA.
scl022190.1_154-S	123.464	1.84	Ubc	XM_147315.1	Mus musculus ubiquitin C (Ubc), mRNA.
gi_21070949_ref_NM_01	122.4365	1.84	Ubc	XM_147315.1	Mus musculus ubiquitin C (Ubc), mRNA.
scl019982.5_48-S	121.8057	1.81	Rpl36a	NM_019865.2	Mus musculus ribosomal protein L36a (Rpl36a), mRNA.
scl0012846.1_253-S	121.2994	1.87	Comt	XM_147265.1	Mus musculus catechol-O-methyltransferase (Comt), mRNA.
scl067684.2_37-S	113.8468	1.81	3300001P08Rik	NM_026313.1	Mus musculus RIKEN cDNA 3300001P08 gene (3300001P08Rik), mRNA.
scl00319171.1_322-S	113.8282	1.99	Hist1h2ao	NM_178185.1	Mus musculus histone 1, H2ao (Hist1h2ao), mRNA.
scl50103.16_203-S	112.5177	1.83	Mtch1	NM_019880.2	Mus musculus mitochondrial carrier homolog 1 (C. elegans) (Mtch1), mRNA.
scl51536.11_7-S	107.3017	1.84	Etf1	NM_144866.2	Mus musculus eukaryotic translation termination factor 1 (Etf1), mRNA.
scl00192197.1_323-S	105.4942	1.98	Bcas3	NM_138681.2	Mus musculus breast carcinoma amplified sequence 3 (Bcas3), mRNA.
scl018786.2_1-S	103.8338	1.85	Plaa	NM_172695.1	Mus musculus phospholipase A2, activating protein (Plaa), mRNA.
scl0326622.12_322-S	102.1663	2.17	Upf2	XM_140801.3	Mus musculus UPF2 regulator of nonsense transcripts homolog (yeast) (Upf2), mRNA.
scl0214290.2_34-S	101.725	1.99	Zcchc6	NM_153538.1	Mus musculus zinc finger, CCHC domain containing 6 (Zcchc6), mRNA.
scl00230249.2_159-S	99.8888	1.84	AI314180	NM_172381.2	Mus musculus expressed sequence AI314180 (AI314180), mRNA.
scl21063.24_624-S	98.3369	1.91	Golga2	NM_133852.1	Mus musculus golgi autoantigen, golgin subfamily a, 2 (Golga2), mRNA.
scl37402.3_73-S	98.2764	1.82	Sas	NM_025982	Mus musculus sarcoma amplified sequence (Sas), mRNA.
scl41018.11_650-S	98.135	1.85	BC034054	NM_178049.2	Mus musculus cDNA sequence BC034054 (BC034054), mRNA.
scl022218.1_29-S	96.9332	1.7	Sumo1	NM_009460.1	
GI_21070949-S	96.7979	1.74	Ubc	NM_019639.2	Mus musculus ubiquitin C (Ubc), mRNA.
scl27412.8.1_4-S	96.439	1.87	Tpst2	NM_009419	Mus musculus protein-tyrosine sulfotransferase 2 (Tpst2), mRNA.
scl0004093.1_54-S	96.2774	3.37	Mcm7	NM_008568.1	Mus musculus minichromosome maintenance deficient 7 (S. cerevisiae) (Mcm7), mRNA.
scl20297.9_280-S	94.7301	1.69	Ptpns1	XM_149178.1	Mus musculus protein tyrosine phosphatase, non-receptor type substrate 1 (Ptpns1), mRNA.
scl50160.5.1_23-S	94.0495	1.78	BC008155	NM_145410.1	Mus musculus cDNA sequence BC008155 (BC008155), mRNA.
scl35001.15_103-S	93.2162	1.95	Plekha2	NM_031257.2	Mus musculus pleckstrin homology domain-containing, family A (phosphoinositide binding specific) member 2 (Plekha2), mRNA.
scl0003150.1_14-S	92.4713	4.56	Golga2	NM_133852.1	Mus musculus golgi autoantigen, golgin subfamily a, 2 (Golga2), mRNA.
scl00227682.2_166-S	91.1387	2.62	G430055L02Rik	NM_145520.2	
scl00215751.1_299-S	91.1283	1.91	BC013529	NM_145418.1	Mus musculus cDNA sequence BC013529 (BC013529), mRNA.
scl0067379.1_289-S	91.0477	1.77	Dedd2	NM_207677.1	Mus musculus death effector domain-containing DNA binding protein 2 (Dedd2), transcript variant 2, mRNA.
scl0015183.2_163-S	90.7275	1.99	Hdac3	NM_010411.1	Mus musculus histone deacetylase 3 (Hdac3), mRNA.
scl0002552.1_594-S	90.4584	1.96	Triobp	NM_138579.2	Mus musculus TRIO and F-actin binding protein (Triobp), mRNA.
scl41012.9.1_84-S	89.1561	1.67	Ugalt2	NM_016752.1	Mus musculus UDP-galactose translocator 2 (Ugalt2), mRNA.
scl012864.3_58-S	88.1809	1.72	Cox6c	NM_053071.1	Mus musculus cytochrome c oxidase, subunit VIc (Cox6c), mRNA.
scl019244.5_2-S	87.2207	1.64	Ptp4a2	NM_008974.2	Mus musculus protein tyrosine phosphatase 4a2 (Ptp4a2), mRNA.
scl47805.7_5-S	86.52	1.77	Grina	NM_023168.2	Mus musculus glutamate receptor, ionotropic, N-methyl D-asparate-associated protein 1 (glutamate binding) (Grina), mRNA.
scl41300.23_511-S	85.6059	2.13	Atp2a3	NM_016745.2	Mus musculus ATPase, Ca++ transporting, ubiquitous (Atp2a3), mRNA.
scl39318.8_348-S	84.4304	1.79	Wbp2	NM_016852.1	Mus musculus WW domain binding protein 2 (Wbp2), mRNA.
scl0064934.1_269-S	84.2458	2.51	Pes1	NM_022889.2	Mus musculus pescadillo homolog 1, containing BRCT domain (zebrafish) (Pes1), mRNA.
scl00239743.2_208-S	81.4228	1.76	Klhl6	NM_183390.1	Mus musculus kelch-like 6 (Drosophila) (Klhl6), mRNA.
scl0055943.1_284-S	81.3076	1.88	Stx8	NM_018768.1	Mus musculus syntaxin 8 (Stx8), mRNA.
scl33408.13_324-S	81.2314	1.7	Ctcf	NM_007794.1	Mus musculus CCCTC-binding factor (Ctcf), mRNA.
scl023849.4_8-S	79.8854	2.17	Copeb	NM_011803.1	Mus musculus core promoter element binding protein (Copeb), mRNA.
scl50763.13.1_199-S	79.8823	1.89	Flot1	NM_008027.1	Mus musculus flotillin 1 (Flot1), mRNA.
scl0104771.9_30-S	79.3192	1.93	1200003C05Rik	NM_024205.1	Mus musculus RIKEN cDNA 1200003C05 gene (1200003C05Rik), mRNA.
scl0069654.1_290-S	79.2864	1.61	Dctn2	NM_027151.1	Mus musculus dynactin 2 (Dctn2), mRNA.
scl45683.9_474-S	78.5062	1.75	5730469M10Rik	NM_027464	Mus musculus RIKEN cDNA 5730469M10 gene (5730469M10Rik), mRNA.
scl41358.1_245-S	78.3159	1.87	1810027O10Rik	XM_109683.4	Mus musculus RIKEN cDNA 1810027O10 gene (1810027O10Rik), mRNA.
scl52614.18.1_13-S	77.982	1.66	D19Bwg1357e	NM_177474.2	Mus musculus DNA segment, Chr 19, Brigham & Womens Genetics 1357 expressed (D19Bwg1357e), mRNA.
scl066840.3_20-S	77.4476	3.75	0610008N23Rik	NM_025793.1	
scl22011.14.1179_292-S	77.0891	1.74	6330505N24Rik	XM_130991.3	

scl011544.1_20-S	76.3655	1.63	Adprh	NM_007414.2	Mus musculus ADP-ribosylarginine hydrolase (Adprh), mRNA.
scl0012848.2_316-S	76.3185	1.81	Cops2	NM_009939.1	Mus musculus COP9 (constitutive photomorphogenic) homolog, subunit 2 (Arabidopsis thaliana) (Cops2), mRNA.
scl075786.2_11-S	76.2723	5.76	4930432B04Rik	XM_130287.5	Mus musculus RIKEN cDNA 4930432B04 gene (4930432B04Rik), mRNA.
scl0017859.1_306-S	76.1169	1.75	Mxi1	NM_010847.1	Mus musculus Max interacting protein 1 (Mxi1), mRNA.
scl30036.21_295-S	75.7934	1.79	Tax1bp1	NM_025816.1	Mus musculus Tax1 (human T-cell leukemia virus type I) binding protein 1 (Tax1bp1), mRNA.
scl29636.20.1_18-S	75.4743	1.78	1110061O04Rik	NM_026849	Mus musculus RIKEN cDNA 1110061O04 gene (1110061O04Rik), mRNA.
scl34266.7_421-S	75.0922	1.73	2700067E09Rik	NM_175185.2	Mus musculus RIKEN cDNA 2700067E09 gene (2700067E09Rik), mRNA.
scl26191.2_17-S	74.8581	1.65	Selpl	NM_009151.2	Mus musculus selectin, platelet (p-selectin) ligand (Selpl), mRNA.
scl011964.1_274-S	74.8038	1.72	Atp6v1a1	NM_007508.2	Mus musculus ATPase, H+ transporting, V1 subunit A, isoform 1 (Atp6v1a1), mRNA.
scl0227290.1_240-S	74.7304	1.61	Aamp	NM_146110.1	Mus musculus angio-associated migratory protein (Aamp), mRNA.
scl24399.13_176-S	74.4773	1.84	Gba2	NM_172692.1	Mus musculus glucosidase beta 2 (Gba2), mRNA.
scl32033.10.26_108-S	74.3831	1.63	BC006909	NM_145589.2	Mus musculus cDNA sequence BC006909 (BC006909), mRNA.
scl24433.7.1_53-S	72.3243	1.69	Bag1	NM_009736.1	Mus musculus Bcl2-associated athanogene 1 (Bag1), mRNA.
scl23771.9.1_58-S	71.5908	1.65	Eif3s2	NM_018799.1	Mus musculus eukaryotic translation initiation factor 3, subunit 2 (beta) (Eif3s2), mRNA.
scl00213484.2_217-S	71.3883	1.85	BC036718	NM_153136.1	Mus musculus cDNA sequence BC036718 (BC036718), mRNA.
scl067905.1_18-S	70.8503	1.62	2810423O19Rik	NM_026447	Mus musculus RIKEN cDNA 2810423O19 gene (2810423O19Rik), transcript variant 1, mRNA.
scl000063.1_0-S	70.7269	2.06	Nit1	NM_012049.1	Mus musculus nitrilase 1 (Nit1), mRNA.
scl012366.11_305-S	70.2833	1.59	Casp2	NM_007610.1	Mus musculus caspase 2 (Casp2), mRNA.
scl068195.1_129-S	70.1945	1.55	Rnaset2	NM_026611.1	Mus musculus ribonuclease T2 (Rnaset2), mRNA.
scl51735.42.647_134-S	69.818	2.04	5430411K18Rik	XM_140491.4	Mus musculus RIKEN cDNA 5430411K18 gene (5430411K18Rik), mRNA.
scl43073.2_375-S	69.5982	2.42	Hspa2	NM_008301.2	Mus musculus heat shock protein 2 (Hspa2), mRNA.
scl022793.9_329-S	69.4671	1.79	Zyx	NM_011777.1	Mus musculus zyxin (Zyx), mRNA.
scl0216792.1_314-S	68.2324	1.85	A230051G13Rik	NM_173785.2	
scl0016599.2_295-S	67.7636	1.56	Klf3	NM_008453.2	Mus musculus Kruppel-like factor 3 (basic) (Klf3), mRNA.
scl40305.32_46-S	67.4939	1.77	Cyfip2	XM_147526.1	Mus musculus cytoplasmic FMR1 interacting protein 2 (Cyfip2), mRNA.
scl00217864.2_195-S	67.2196	1.94	D12Wsu95e	NM_198023.1	Mus musculus DNA segment, Chr 12, Wayne State University 95, expressed (D12Wsu95e), mRNA.
scl068877.8_322-S	66.3113	1.54	Maf1	NM_026859.2	Mus musculus MAF1 homolog (yeast) (Maf1), mRNA.
scl067283.1_285-S	66.0702	1.91	Slc25a19	NM_026071.1	Mus musculus solute carrier family 25 (mitochondrial deoxynucleotide carrier), member 19 (Slc25a19), mRNA.
scl36580.22.1_188-S	65.7725	1.55	Copb2	NM_015827.1	Mus musculus coatomer protein complex, subunit beta 2 (beta prime) (Copb2), mRNA.
scl48796.17_240-S	65.6931	2.29	2700067D09Rik	NM_028301.1	Mus musculus RIKEN cDNA 2700067D09 gene (2700067D09Rik), mRNA.
scl0067673.1_322-S	65.2968	1.59	Tceb2	NM_026305.1	Mus musculus transcription elongation factor B (SIII), polypeptide 2 (Tceb2), mRNA.
scl21986.3.1_30-S	65.0483	2.23	Apoa1bp	NM_144897	Mus musculus apolipoprotein A-I binding protein (Apoa1bp), mRNA.
scl0011677.2_223-S	65.0128	1.59	Akr1b3	NM_009658.2	Mus musculus aldo-keto reductase family 1, member B3 (aldose reductase) (Akr1b3), mRNA.
scl39506.28.1461_10-S	64.8115	1.68	Slc4a1	NM_011403.1	Mus musculus solute carrier family 4 (anion exchanger), member 1 (Slc4a1), mRNA.
scl38917.7_7-S	64.7129	1.64	Gja1	NM_010288.2	Mus musculus gap junction membrane channel protein alpha 1 (Gja1), mRNA.
scl0067590.2_244-S	64.4775	2.39	4930521E07Rik	NM_026260.2	Mus musculus RIKEN cDNA 4930521E07 gene (4930521E07Rik), mRNA.
scl013728.1_329-S	64.2852	1.58	Mark2	NM_007928.1	Mus musculus MAP/microtubule affinity-regulating kinase 2 (Mark2), mRNA.
scl022642.16_28-S	64.2714	2.21	Zbtb17	NM_009541.1	
scl25133.32.1_60-S	64.2364	1.59	Nrd1	NM_146150.2	Mus musculus nardilysin, N-arginine dibasic convertase, NRD convertase 1 (Nrd1), mRNA.
scl52850.12_273-S	64.0154	1.66	Rela	NM_009045.2	Mus musculus v-rel reticuloendotheliosis viral oncogene homolog A (avian) (Rela), mRNA.
scl50203.3_209-S	63.8136	1.64	Gfer	NM_023040.2	Mus musculus growth factor, erv1 (S. cerevisiae)-like (augmenter of liver regeneration) (Gfer), transcript variant 2, mRNA.
scl33269.35.1_230-S	63.5049	1.97	Plcg2	NM_172285.1	Mus musculus phospholipase C, gamma 2 (Plcg2), mRNA.
scl25917.6_37-S	63.5011	1.52	Ywhag	NM_018871.2	Mus musculus 3-monooxgenase/tryptophan 5-monooxgenase activation protein, gamma polypeptide (Ywhag), mRNA.
scl018022.1_253-S	63.3787	1.75	Nfe2	NM_008685.2	Mus musculus nuclear factor, erythroid derived 2 (Nfe2), mRNA.
scl017169.9_120-S	63.3575	1.68	Mark3	NM_021516.1	Mus musculus MAP/microtubule affinity-regulating kinase 3 (Mark3), mRNA.
scl48582.2_713-S	63.2558	3.1	Gp5	NM_008148.2	Mus musculus glycoprotein 5 (platelet) (Gp5), mRNA.
scl0021372.2_85-S	63.0921	2.11	Tbl1x	NM_020601.1	Mus musculus transducin (beta)-like 1 X-linked (Tbl1x), mRNA.
scl51588.6_379-S	62.9236	1.59	D030070L09Rik	NM_172625.1	Mus musculus RIKEN cDNA D030070L09 gene (D030070L09Rik), mRNA.
scl29854.5.1_26-S	62.8378	1.55	Aup1	NM_007517.1	Mus musculus ancient ubiquitous protein (Aup1), mRNA.
scl0065111.1_63-S	62.448	1.54	Dap3	NM_022994.2	Mus musculus death associated protein 3 (Dap3), mRNA.
scl00212307.2_257-S	62.2433	1.82	Mapre2	NM_153058.2	Mus musculus microtubule-associated protein, RP/EB family, member 2 (Mapre2), mRNA.
scl0014897.1_159-S	62.0123	1.58	Trip12	NM_133975.2	
scl0057261.1_145-S	61.993	1.67	Brd4	NM_020508.2	Mus musculus bromodomain containing 4 (Brd4), mRNA.
scl52908.26.6_30-S	61.7588	2.33	2610041P08Rik	NM_198008.1	Mus musculus RIKEN cDNA 2610041P08 gene (2610041P08Rik), mRNA.
scl40468.10_17-S	61.7131	1.55	9130023F12Rik	NM_181411.2	Mus musculus RIKEN cDNA 9130023F12 gene (9130023F12Rik), mRNA.
scl0066775.2_265-S	61.677	3.32	4933428I03Rik	NM_025760.2	Mus musculus RIKEN cDNA 4933428I03 gene (4933428I03Rik), mRNA.

scl46775.6_475-S	61.5226	1.77	Asb8	NM_030121.2	Mus musculus ankyrin repeat and SOCS box-containing protein 8 (Asb8), mRNA.
scl0170768.1_23-S	61.1346	1.69	Pfkfb3	NM_172976.1	Mus musculus 6-phosphofructo-2-kinase/fructose-2,6-biphosphatase 3 (Pfkfb3), mRNA.
scl0077480.1_264-S	61.0758	1.65	C330002I19Rik	XM_126866.5	
scl0105445.1_143-S	61.0003	2.43	Dock9	XM_358315.1	
scl0117599.1_293-S	60.7499	1.74	Helb	NM_080446.1	Mus musculus helicase (DNA) B (Helb), mRNA.
scl35429.19.1_59-S	60.7196	1.56	1300017J02Rik	NM_027918.1	Mus musculus RIKEN cDNA 1300017J02 gene (1300017J02Rik), mRNA.
scl20431.14.1_58-S	60.6922	2.76	2310043D08Rik	XM_130428.2	
scl00109113.1_250-S	60.6331	1.56	Uhrf2	NM_144873.1	Mus musculus ubiquitin-like, containing PHD and RING finger domains 2 (Uhrf2), mRNA.
scl26137.12_266-S	60.5946	1.56	2400003N08Rik	NM_178622.3	Mus musculus RIKEN cDNA 2400003N08 gene (2400003N08Rik), mRNA.
scl0067921.2_259-S	60.1835	1.61	2510010F15Rik	NM_026454.2	Mus musculus RIKEN cDNA 2510010F15 gene (2510010F15Rik), mRNA.
scl38677.11_311-S	59.9989	1.79	Csnk1g2	NM_134002.1	Mus musculus casein kinase 1, gamma 2 (Csnk1g2), mRNA.
scl022333.10_87-S	59.2893	1.5	Vdac1	NM_011694	Mus musculus voltage-dependent anion channel 1 (Vdac1), mRNA.
scl50767.4_663-S	59.0844	1.57	Nrm	NM_134122.1	Mus musculus nurim (nuclear envelope membrane protein) (Nrm), mRNA.
scl36803.7_641-S	58.968	1.81	AI449441	NM_172453.1	Mus musculus expressed sequence AI449441 (AI449441), mRNA.
scl073122.1_292-S	58.9044	1.67	3110018K12Rik	XM_129857.4	Mus musculus RIKEN cDNA 3110018K12 gene (3110018K12Rik), mRNA.
scl26182.5_119-S	58.5998	1.7	Gltp	NM_019821.2	Mus musculus glycolipid transfer protein (Gltp), mRNA.
scl43536.32_392-S	58.3743	1.72	Ipo11	NM_029665.2	Mus musculus importin 11 (Ipo11), mRNA.
scl0208606.2_109-S	57.8282	2.68	1500011J06Rik	XM_132343.3	
scl17286.15.1_2-S	57.384	6.28	Selp	NM_011347.1	Mus musculus selectin, platelet (Selp), mRNA.
scl54639.14_151-S	57.3061	1.67	Cstf2	NM_133196.3	Mus musculus cleavage stimulation factor, 3 pre-RNA subunit 2 (Cstf2), mRNA.
scl067865.1_323-S	57.1896	1.51	Rgs10	NM_026418.1	Mus musculus regulator of G-protein signalling 10 (Rgs10), mRNA.
scl24427.29_125-S	56.877	1.54	Ubap2	NM_026872.1	Mus musculus ubiquitin-associated protein 2 (Ubap2), mRNA.
scl34132.19.1_1-S	56.6508	1.72	Stxbp2	NM_011503.2	Mus musculus syntaxin binding protein 2 (Stxbp2), mRNA.
scl42747.5_440-S	56.5765	1.77	6720458F09Rik	NM_177374.2	Mus musculus RIKEN cDNA 6720458F09 gene (6720458F09Rik), mRNA.
scl014057.6_27-S	56.5659	1.5	Sfxn1	NM_027324.2	Mus musculus sideroflexin 1 (Sfxn1), mRNA.
scl38697.44.1_55-S	55.8142	1.76	Abca7	NM_013850.1	Mus musculus ATP-binding cassette, sub-family A (ABC1), member 7 (Abca7), mRNA.
scl33146.4.49_0-S	55.6687	1.64	Ndufa3	NM_025348	Mus musculus NADH dehydrogenase (ubiquinone) 1 alpha subcomplex, 3 (Ndufa3), mRNA.
scl068066.1_115-S	55.3543	1.46	D11Ertd333e	NM_026542.1	
scl50197.1.198_198-S	55.3465	1.66	Fahd1	NM_023480.1	
scl067049.4_280-S	55.3196	1.99	Pus3	NM_023292.2	
scl0269523.2_3-S	55.2191	1.47	Vcp	NM_009503.2	Mus musculus valosin containing protein (Vcp), mRNA.
scl53441.6.1_144-S	55.1648	1.5	Aip	NM_016666	Mus musculus aryl-hydrocarbon receptor-interacting protein (Aip), mRNA.
scl49807.23_539-S	54.9198	2.42	Tbc1d5	NM_028162.2	Mus musculus TBC1 domain family, member 5 (Tbc1d5), mRNA.
scl0067899.2_259-S	54.9186	1.59	2010110K16Rik	NM_026442.2	Mus musculus RIKEN cDNA 2010110K16 gene (2010110K16Rik), mRNA.
scl0056738.1_278-S	54.6935	1.82	Mocs1	NM_020042.1	Mus musculus molybdenum cofactor synthesis 1 (Mocs1), transcript variant 1, mRNA.
scl0098366.1_13-S	54.4521	1.48	Smap1	NM_028534.1	Mus musculus stromal membrane-associated protein 1 (Smap1), mRNA.
scl19927.15.1_16-S	54.274	1.5	Dnttip1	NM_133763.1	Mus musculus deoxynucleotidyltransferase, terminal, interacting protein 1 (Dnttip1), mRNA.
scl25806.10.1_16-S	54.0784	1.74	Zdhhc4	NM_028379.1	Mus musculus zinc finger, DHHC domain containing 4 (Zdhhc4), mRNA.
scl014897.1_33-S	53.9783	1.57	Trip12	NM_133975.2	
scl020715.6_281-S	53.9292	1.48	Serpina3g	XM_354694.1	Mus musculus serine (or cysteine) proteinase inhibitor, clade A, member 3G (Serpina3g), mRNA.
scl24928.17_534-S	53.7091	1.71	Phc2	NM_018774.1	Mus musculus polyhomeotic-like 2 (Drosophila) (Phc2), mRNA.
scl018641.1_29-S	53.5359	1.63	Pfkl	NM_008826.2	Mus musculus phosphofructokinase, liver, B-type (Pfkl), mRNA.
IGHV1S132_AF304552_Ig	53.4313	11.97			
scl019167.11_85-S	53.258	1.45	Psma3	NM_011184.2	Mus musculus proteasome (prosome, macropain) subunit, alpha type 3 (Psma3), mRNA.
scl078325.1_150-S	53.2142	1.8	2700092H06Rik	XM_132529.2	Mus musculus RIKEN cDNA 2700092H06 gene (2700092H06Rik), mRNA.
scl19517.6_242-S	53.0912	1.56	Surf4	NM_011512.2	Mus musculus surfeit gene 4 (Surf4), mRNA.
scl0067184.2_306-S	52.9858	1.61	Grim19	NM_023312.1	Mus musculus genes associated with retinoid-IFN-induced mortality 19 (Grim19), mRNA.
scl37040.9_46-S	52.8404	1.67	Dpagt1	NM_007875.2	Mus musculus dolichyl-phosphate (UDP-N-acetylglucosamine) acetylglucosaminephosphotransferase 1 (GlcNAc-1-P transferase) (Dpagt1), mRNA.
scl23930.5.1_96-S	52.8367	1.55	Atp6v0b	NM_033617.1	Mus musculus ATPase, H+ transporting, V0 subunit B (Atp6v0b), mRNA.
scl0080913.2_173-S	52.709	1.86	Pum2	NM_030723.1	Mus musculus pumilio 2 (Drosophila) (Pum2), mRNA.
scl25096.6.1_229-S	52.4854	1.44	Tal1	NM_011527.1	Mus musculus T-cell acute lymphocytic leukemia 1 (Tal1), mRNA.
scl0078781.2_278-S	52.4528	1.63	Zc3hav1	NM_028864.1	Mus musculus zinc finger CCCH type, antiviral 1 (Zc3hav1), mRNA.
scl43457.5.1_29-S	52.4511	1.51	Mrps30	NM_021556.2	Mus musculus mitochondrial ribosomal protein S30 (Mrps30), mRNA.
scl40141.3_333-S	52.428	1.63	5730466P16Rik	NM_173453.1	Mus musculus RIKEN cDNA 5730466P16 gene (5730466P16Rik), mRNA.
scl39999.14.1_8-S	52.4172	2.71	Alox12	NM_007440.2	Mus musculus arachidonate 12-lipoxygenase (Alox12), mRNA.
scl0017765.2_254-S	52.3742	2.18	Mtf2	NM_013827.1	Mus musculus metal response element binding transcription factor 2 (Mtf2), mRNA.

scl49175.14_151-S	52.0357	2.02	6820449I09Rik	NM_177128.2	
scl0024135.1_295-S	51.9898	1.91	Zfp68	NM_013844.1	Mus musculus zinc finger protein 68 (Zfp68), mRNA.
scl27109.17.1_35-S	51.9055	1.58	Plod3	NM_011962.2	Mus musculus procollagen-lysine, 2-oxoglutarate 5-dioxygenase 3 (Plod3), mRNA.
scl0020661.1_145-S	51.8149	1.65	Sort1	NM_019972	Mus musculus sortilin 1 (Sort1), mRNA.
scl00320332.2_0-S	51.7236	3.28	Hist4h4	NM_175652.1	Mus musculus histone 4, H4 (Hist4h4), mRNA.
scl39617.28.1_49-S	51.5782	1.59	Thrap4	NM_011869.1	Mus musculus thyroid hormone receptor associated protein 4 (Thrap4), mRNA.
scl25126.19.1_29-S	51.4251	1.71	Faf1	NM_007983.2	Mus musculus Fas-associated factor 1 (Faf1), mRNA.
scl23946.8.1_41-S	51.4078	1.43	Urod	NM_009478.1	Mus musculus uroporphyrinogen decarboxylase (Urod), mRNA.
scl53904.6_21-S	51.2267	2.02	Nsbp1	NM_016710.1	Mus musculus nucleosome binding protein 1 (Nsbp1), mRNA.
scl00214290.1_251-S	51.1125	1.47	Zcchc6	NM_153538.1	Mus musculus zinc finger, CCHC domain containing 6 (Zcchc6), mRNA.
scl068152.6_9-S	50.9762	1.65	5830415L20Rik	NM_026583.2	Mus musculus RIKEN cDNA 5830415L20 gene (5830415L20Rik), mRNA.
scl020250.10_235-S	50.9181	1.54	Scd2	NM_009128.1	Mus musculus stearoyl-Coenzyme A desaturase 2 (Scd2), mRNA.
scl072121.12_86-S	50.8009	1.81	2010308M01Rik	XM_131083.2	Mus musculus RIKEN cDNA 2010308M01 gene (2010308M01Rik), mRNA.
scl33743.13.1_0-S	50.677	1.7	Sf4	NM_027481.1	Mus musculus splicing factor 4 (Sf4), mRNA.
scl47734.11_199-S	50.437	1.57	Map3k7ip1	NM_025609.2	Mus musculus mitogen-activated protein kinase kinase kinase 7 interacting protein 1 (Map3k7ip1), mRNA.
scl013000.1_10-S	50.2622	1.66	Csnk2a2	NM_009974.2	Mus musculus casein kinase II, alpha 2, polypeptide (Csnk2a2), mRNA.
scl34795.4.1_9-S	50.1719	1.75	Sap30	NM_021788.1	Mus musculus sin3 associated polypeptide (Sap30), mRNA.
scl20687.14.1_105-S	50.1445	1.49	Slc43a3	NM_021398.1	Mus musculus solute carrier family 43, member 3 (Slc43a3), mRNA.
scl54266.5_48-S	50.1158	1.68	Rap2c	NM_172413.1	Mus musculus RAP2C, member of RAS oncogene family (Rap2c), mRNA.
scl0067452.2_1-S	50.1099	1.6	1200006O19Rik	NM_026164.1	Mus musculus RIKEN cDNA 1200006O19 gene (1200006O19Rik), mRNA.
scl019326.1_315-S	49.7696	1.44	Rab11b	NM_008997.1	Mus musculus RAB11B, member RAS oncogene family (Rab11b), mRNA.
scl49386.9_715-S	49.6218	1.68	Ppm1f	NM_176833.2	Mus musculus protein phosphatase 1F (PP2C domain containing) (Ppm1f), mRNA.
scl31560.6.38_45-S	49.5635	1.95	1110006G06Rik	NM_028661.1	Mus musculus RIKEN cDNA 1110006G06 gene (1110006G06Rik), mRNA.
scl0027407.2_308-S	49.2508	1.51	Abcf2	NM_013853.1	Mus musculus ATP-binding cassette, sub-family F (GCN20), member 2 (Abcf2), mRNA.
scl39908.21.1_34-S	49.2438	2.76	Cpd	NM_007754.1	Mus musculus carboxypeptidase D (Cpd), mRNA.
scl0208715.1_140-S	49.0902	1.71	Hmgcs1	NM_145942.2	Mus musculus 3-hydroxy-3-methylglutaryl-Coenzyme A synthase 1 (Hmgcs1), mRNA.
scl066144.2_329-S	48.9166	1.54	Atp6v1f	NM_025381	Mus musculus ATPase, H+ transporting, V1 subunit F (Atp6v1f), mRNA.
scl54450.17.1_6-S	48.8252	1.55	DXImx40e	NM_138603.2	Mus musculus DNA segment, Chr X, Immunex 40, expressed (DXImx40e), mRNA.
scl073373.8_41-S	48.7895	1.75	1700048E23Rik	NM_028521.1	Mus musculus RIKEN cDNA 1700048E23 gene (1700048E23Rik), mRNA.
scl084652.1_47-S	48.5618	1.5	Drctnnb1a	NM_053090	Mus musculus down-regulated by Ctnnb1, a (Drctnnb1a), mRNA.
scl25992.16.1_1-S	48.3853	2.03	Asl	NM_133768.1	Mus musculus argininosuccinate lyase (Asl), mRNA.
scl24620.16_177-S	48.1916	1.46	BC004012	NM_138671	Mus musculus cDNA sequence BC004012 (BC004012), mRNA.
scl23294.9_553-S	48.1236	1.55	Zfp639	NM_144519	
scl068045.1_243-S	48.107	1.42	2700060E02Rik	NM_026528.1	Mus musculus RIKEN cDNA 2700060E02 gene (2700060E02Rik), mRNA.
scl0075221.1_274-S	47.8128	1.51	Dpp3	NM_133803.1	Mus musculus dipeptidylpeptidase 3 (Dpp3), mRNA.
scl0072278.1_319-S	47.4115	2.17	D9Ertd392e	NM_028181.1	
scl23927.19.1_34-S	47.1329	1.79	Ipo13	NM_146152.2	Mus musculus importin 13 (Ipo13), mRNA.
scl50689.6_424-S	47.0917	1.51	Slc35b1	XM_128634.4	Mus musculus solute carrier family 35, member B1 (Slc35b1), mRNA.
scl00232227.2_246-S	47.0911	2.01	D6Ertd349e	NM_182784.1	Mus musculus DNA segment, Chr 6, ERATO Doi 349, expressed (D6Ertd349e), mRNA.
scl43315.9.1_17-S	47.0673	2.05	Sh3yl1	NM_013709.2	Mus musculus Sh3 domain YSC-like 1 (Sh3yl1), mRNA.
scl0214932.1_204-S	46.9417	2.04	Cecr5	NM_144815.1	Mus musculus cat eye syndrome chromosome region, candidate 5 homolog (human) (Cecr5), mRNA.
scl23219.3_221-S	46.8453	2.13	Rab33b	NM_016858.1	Mus musculus RAB33B, member of RAS oncogene family (Rab33b), mRNA.
scl51768.11.1_9-S	46.4704	1.67	Acaa2	NM_177470.2	Mus musculus acetyl-Coenzyme A acyltransferase 2 (mitochondrial 3-oxoacyl-Coenzyme A thiolase) (Acaa2), mRNA.
scl31412.26.1_74-S	46.3126	1.56	Pold1	NM_011131.2	Mus musculus polymerase (DNA directed), delta 1, catalytic subunit (Pold1), mRNA.
scl0217342.1_24-S	46.2425	1.41	B230113M03Rik	XM_109923.4	Mus musculus RIKEN cDNA B230113M03 gene (B230113M03Rik), mRNA.
scl066390.1_295-S	46.1769	1.57	2310042G06Rik	NM_025531.2	Mus musculus RIKEN cDNA 2310042G06 gene (2310042G06Rik), mRNA.
scl55061.19.1_283-S	46.0423	1.42	Slc38a5	NM_172479.1	
scl40538.6.1_45-S	46.0195	1.51	H2afv	XM_126043.3	
scl33848.6_136-S	45.8163	1.63	4933411K20Rik	NM_025747.2	Mus musculus RIKEN cDNA 4933411K20 gene (4933411K20Rik), mRNA.
scl0328526.4_134-S	45.7135	1.63	LOC328526	XM_283274.2	
scl27603.2.1_3-S	45.637	2.07	Cxcl7	NM_023785.1	Mus musculus chemokine (C-X-C motif) ligand 7 (Cxcl7), mRNA.
scl0075613.2_301-S	45.2768	1.89	2610034E13Rik	NM_029365.1	Mus musculus RIKEN cDNA 2610034E13 gene (2610034E13Rik), mRNA.
scl0072691.1_193-S	45.2508	1.78	2810048G17Rik	NM_133746.2	Mus musculus RIKEN cDNA 2810048G17 gene (2810048G17Rik), mRNA.
scl020239.25_113-S	45.2403	1.52	Sca2	NM_009125.1	Mus musculus spinocerebellar ataxia 2 homolog (human) (Sca2), mRNA.
scl014897.1_29-S	45.1738	1.45	Trip12	NM_133975.2	
scl019298.8_30-S	45.1432	1.6	Pex19	NM_023041.2	Mus musculus peroxisome biogenesis factor 19 (Pex19), mRNA.

scl066725.8_229-S	45.1216	1.84	4921513O20Rik	NM_025730.1	Mus musculus RIKEN cDNA 4921513O20 gene (4921513O20Rik), mRNA.
scl47705.17_95-S	45.1192	2	L3mbtl2	NM_145993.2	Mus musculus l(3)mbt-like 2 (Drosophila) (L3mbtl2), mRNA.
scl35771.8.1_41-S	45.1119	1.71	2410076I21Rik	XM_134948.2	Mus musculus RIKEN cDNA 2410076I21 gene (2410076I21Rik), mRNA.
scl40273.5_591-S	44.9963	1.63	Maml1	NM_175334.2	Mus musculus mastermind like 1 (Drosophila) (Maml1), mRNA.
scl34002.14.1_30-S	44.9938	1.65	2210415M20Rik	NM_027338.1	Mus musculus RIKEN cDNA 2210415M20 gene (2210415M20Rik), mRNA.
scl16186.5.1_30-S	44.9122	2.67	Rgs18	NM_022881.2	Mus musculus regulator of G-protein signaling 18 (Rgs18), mRNA.
scl067248.2_33-S	44.8342	1.78	Rpl39	NM_026055.1	Mus musculus ribosomal protein L39 (Rpl39), mRNA.
scl019935.5_129-S	44.8227	1.47	Mrpl23	NM_011288.1	Mus musculus mitochondrial ribosomal protein L23 (Mrpl23), mRNA.
scl25676.27_663-S	44.7478	2.12	1110037F02Rik	XM_283937.2	Mus musculus RIKEN cDNA 1110037F02 gene (1110037F02Rik), mRNA.
scl0107094.1_210-S	44.7356	1.63	AA408556	NM_199447.2	Mus musculus expressed sequence AA408556 (AA408556), mRNA.
scl00224938.2_37-S	44.7312	1.5	Pja2	NM_144859.1	Mus musculus praja 2, RING-H2 motif containing (Pja2), mRNA.
scl066656.2_23-S	44.7012	1.4	Eef1d	NM_023240.1	Mus musculus eukaryotic translation elongation factor 1 delta (guanine nucleotide exchange protein) (Eef1d), mRNA.
scl26992.46_0-S	44.6946	1.73	AI481500	NM_133901.1	Mus musculus expressed sequence AI481500 (AI481500), mRNA.
scl46000.3_481-S	44.526	2.6	Ndfip2	NM_029561.1	Mus musculus Nedd4 family interacting protein 2 (Ndfip2), mRNA.
scl020926.1_330-S	44.5066	1.57	Supt6h	NM_009297.1	Mus musculus suppressor of Ty 6 homolog (S. cerevisiae) (Supt6h), mRNA.
scl0068977.2_330-S	44.4767	1.9	Haghl	NM_026897.1	Mus musculus hydroxyacylglutathione hydrolase-like (Haghl), mRNA.
scl00108755.1_37-S	44.4195	1.94	2610208E05Rik	NM_175364.2	Mus musculus RIKEN cDNA 2610208E05 gene (2610208E05Rik), mRNA.
scl0108888.1_320-S	44.3299	1.56	Atad3a	NM_179203.1	Mus musculus ATPase family, AAA domain containing 3A (Atad3a), mRNA.
scl0076740.1_108-S	44.288	1.59	C920006C10Rik	NM_133766.1	Mus musculus RIKEN cDNA C920006C10 gene (C920006C10Rik), mRNA.
scl067671.4_8-S	44.1665	1.43	Rpl38	NM_023372.1	Mus musculus ribosomal protein L38 (Rpl38), mRNA.
scl0012045.1_114-S	44.1583	1.41	Bcl2a1b	NM_007534	Mus musculus B-cell leukemia/lymphoma 2 related protein A1b (Bcl2a1b), mRNA.
scl052504.1_184-S	44.0643	1.62	2810429O05Rik	NM_134046.3	Mus musculus RIKEN cDNA 2810429O05 gene (2810429O05Rik), mRNA.
scl40072.13_201-S	44.0264	1.63	Map2k4	NM_009157	Mus musculus mitogen activated protein kinase kinase 4 (Map2k4), mRNA.
scl53489.23_479-S	43.8825	1.56	Pacs1	XM_283545.1	Mus musculus phosphofurin acidic cluster sorting protein 1 (Pacs1), mRNA.
scl0030791.1_194-S	43.8781	1.55	Slc39a1	NM_013901	Mus musculus solute carrier family 39 (zinc transporter), member 1 (Slc39a1), mRNA.
scl074479.1_19-S	43.6519	1.47	Snx11	NM_028965.2	Mus musculus sorting nexin 11 (Snx11), mRNA.
scl15987.1.1_285-S	43.6214	1.43	AI481316	XM_148986.1	Mus musculus expressed sequence AI481316 (AI481316), mRNA.
scl31965.9_618-S	43.5505	1.5	C430003P19Rik	NM_198017.1	Mus musculus RIKEN cDNA C430003P19 gene (C430003P19Rik), mRNA.
scl017776.1_270-S	43.4504	1.46	Mast2	NM_008641.1	Mus musculus microtubule associated serine/threonine kinase 2 (Mast2), mRNA.
scl0019246.2_230-S	43.4474	1.45	Ptpn1	NM_011201.1	Mus musculus protein tyrosine phosphatase, non-receptor type 1 (Ptpn1), mRNA.
scl23523.7.1_20-S	43.4355	1.63	Fbxo6b	NM_015797.1	Mus musculus F-box only protein 6b (Fbxo6b), mRNA.
scl29846.4_459-S	43.3235	1.43	Gcs1	NM_020619.2	Mus musculus glucosidase 1 (Gcs1), mRNA.
scl27262.6_658-S	43.2536	1.78	9130017A15Rik	NM_177242.3	Mus musculus RIKEN cDNA 9130017A15 gene (9130017A15Rik), mRNA.
scl0269582.3_30-S	43.1821	2.1	Clspn	NM_175554.3	
scl066878.10_113-S	43.0551	1.42	Riok3	NM_024182.2	Mus musculus RIO kinase 3 (yeast) (Riok3), mRNA.
scl0003758.1_1237-S	42.977	1.95	Ubqln1	NM_026842.3	Mus musculus ubiquilin 1 (Ubqln1), transcript variant 1, mRNA.
scl017110.1_293-S	42.8407	2.08	Lzp-s	NM_013590.2	Mus musculus P lysozyme structural (Lzp-s), mRNA.
scl017993.1_123-S	42.8134	1.59	Ndufs4	NM_010887.1	Mus musculus NADH dehydrogenase (ubiquinone) Fe-S protein 4 (Ndufs4), mRNA.
scl37776.6_58-S	42.6856	1.59	D10Jhu81e	NM_138601.1	Mus musculus DNA segment, Chr 10, Johns Hopkins University 81 expressed (D10Jhu81e), mRNA.
scl29521.11_18-S	42.6211	1.48	Grcc3f	NM_145130.1	Mus musculus gene rich cluster, C3f gene (Grcc3f), mRNA.
scl0209011.1_312-S	42.5946	1.61	Sirt7	NM_153056.1	Mus musculus sirtuin 7 (silent mating type information regulation 2, homolog) 7 (S. cerevisiae) (Sirt7), mRNA.
scl37695.8.1_48-S	42.5262	2.5	Nfic	NM_008688.2	Mus musculus nuclear factor I/C (Nfic), mRNA.
scl068276.1_0-S	42.2438	1.6	4930584N22Rik	NM_026654.1	Mus musculus RIKEN cDNA 4930584N22 gene (4930584N22Rik), mRNA.
scl0029875.2_286-S	42.143	2.25	Iqgap1	NM_016721.1	Mus musculus IQ motif containing GTPase activating protein 1 (Iqgap1), mRNA.
scl0051788.1_274-S	42.1322	1.39	H2afz	NM_016750.1	Mus musculus H2A histone family, member Z (H2afz), mRNA.
scl23442.8_471-S	42.015	1.67	1110060F11Rik	NM_026395.1	Mus musculus RIKEN cDNA 1110060F11 gene (1110060F11Rik), mRNA.
scl0017274.1_278-S	41.9903	2.2	Rab8a	NM_023126.2	Mus musculus RAB8A, member RAS oncogene family (Rab8a), mRNA.
scl015204.23_217-S	41.8233	1.85	Herc2	NM_010418.1	Mus musculus hect (homologous to the E6-AP (UBE3A) carboxyl terminus) domain and RCC1 (CHC1)-like domain (RLD) 2 (Herc2), mRNA.
scl067246.4_216-S	41.8204	1.48	2810474O19Rik	XM_132966.3	Mus musculus RIKEN cDNA 2810474O19 gene (2810474O19Rik), mRNA.
scl0003527.1_242-S	41.8029	1.87	Ddx6	NM_007841.2	Mus musculus DEAD (Asp-Glu-Ala-Asp) box polypeptide 6 (Ddx6), mRNA.
scl012367.6_5-S	41.6904	1.61	Casp3	NM_009810.1	Mus musculus caspase 3, apoptosis related cysteine protease (Casp3), mRNA.
scl54141.46.3_34-S	41.6218	1.45	Flna	XM_289920.2	Mus musculus filamin, alpha (Flna), mRNA.
scl071175.1_21-S	41.4942	2.09	4933421G18Rik	XM_127929.3	Mus musculus RIKEN cDNA 4933421G18 gene (4933421G18Rik), mRNA.
scl0002625.1_41-S	41.4505	3.48	2210012G02Rik	NM_025617.1	Mus musculus RIKEN cDNA 2210012G02 gene (2210012G02Rik), mRNA.
scl011949.3_3-S	41.4339	1.42	Atp5c1	NM_020615.2	Mus musculus ATP synthase, H+ transporting, mitochondrial F1 complex, gamma polypeptide 1 (Atp5c1), mRNA.
scl32850.30.1_3-S	41.3371	1.73	Map4k1	NM_008279.1	Mus musculus mitogen activated protein kinase kinase kinase kinase 1 (Map4k1), mRNA.

scl0218100.1_36-S	41.3153	1.65	Zfp322a	NM_172586.2	Mus musculus zinc finger protein 322a (Zfp322a), mRNA.
scl52445.7_23-S	41.2803	1.83	Scd1	NM_009127.2	Mus musculus stearoyl-Coenzyme A desaturase 1 (Scd1), mRNA.
scl36067.18_4-S	41.1393	1.59	Aplp2	NM_009691.1	Mus musculus amyloid beta (A4) precursor-like protein 2 (Aplp2), mRNA.
scl0237422.9_211-S	41.0837	2	BC051080	NM_183172.1	
scl0027223.1_124-S	41.0773	1.81	Trp53bp1	NM_013735.2	Mus musculus transformation related protein 53 binding protein 1 (Trp53bp1), mRNA.
scl00102791.2_289-S	41.0401	1.78	Tcta	NM_133986.1	
scl0020747.2_286-S	40.9982	1.48	Spop	NM_025287.1	Mus musculus speckle-type POZ protein (Spop), mRNA.
scl34388.19_15-S	40.9008	1.4	Ranbp10	NM_145824.3	
scl0011308.2_118-S	40.8194	1.4	Abi1	NM_007380.1	Mus musculus abl-interactor 1 (Abi1), mRNA.
scl00319157.1_0-S	40.6269	2.62	Hist1h4f	NM_175655.1	Mus musculus histone 1, H4f (Hist1h4f), mRNA.
scl31534.6.1_6-S	40.561	1.71	Ckap1	NM_025548.1	Mus musculus cytoskeleton-associated protein 1 (Ckap1), mRNA.
scl50694.13.20_68-S	40.466	1.61	Supt3h	NM_178652.1	Mus musculus suppressor of Ty 3 homolog (S. cerevisiae) (Supt3h), mRNA.
scl22059.9_173-S	40.125	1.69	Pdcd10	NM_019745.2	Mus musculus programmed cell death 10 (Pdcd10), mRNA.
scl25784.5.1_54-S	40.1024	1.77	Pdap1	XM_132501.2	Mus musculus PDGFA associated protein 1 (Pdap1), mRNA.
scl0017993.1_168-S	40.0281	1.51	Ndufs4	NM_010887.1	Mus musculus NADH dehydrogenase (ubiquinone) Fe-S protein 4 (Ndufs4), mRNA.
scl0018230.2_224-S	40.007	1.82	Nxn	NM_008750.2	Mus musculus nucleoredoxin (Nxn), mRNA.
scl00104570.2_297-S	39.9736	1.89	AW011752	NM_134034.1	Mus musculus expressed sequence AW011752 (AW011752), mRNA.
scl46303.7_279-S	39.944	1.45	Lrp10	NM_022993.2	Mus musculus low-density lipoprotein receptor-related protein 10 (Lrp10), mRNA.
scl41207.21.1_236-S	39.8768	1.7	Spag5	NM_017407.1	Mus musculus sperm associated antigen 5 (Spag5), mRNA.
scl37112.9_269-S	39.8449	1.59	Esam1	NM_027102.1	Mus musculus endothelial cell-specific adhesion molecule (Esam1), mRNA.
scl020317.1_68-S	39.7666	1.95	Serpinf1	NM_011340.2	Mus musculus serine (or cysteine) proteinase inhibitor, clade F, member 1 (Serpinf1), mRNA.
scl30730.6_511-S	39.7344	1.37	Cdr2	NM_007672.1	Mus musculus cerebellar degeneration-related 2 (Cdr2), mRNA.
scl00217430.2_0-S	39.7165	1.56	E030024M05Rik	NM_172574.1	Mus musculus RIKEN cDNA E030024M05 gene (E030024M05Rik), mRNA.
scl028035.1_139-S	39.6105	1.48	Usp39	NM_138592.1	Mus musculus ubiquitin specific protease 39 (Usp39), mRNA.
scl54760.5_0-S	39.5312	2.26	Efnb1	NM_010110.2	Mus musculus ephrin B1 (Efnb1), mRNA.
scl20502.5_452-S	39.5024	1.85	Lin7c	NM_011699	Mus musculus lin 7 homolog c (C. elegans) (Lin7c), mRNA.
scl23055.15.1_74-S	39.4893	1.4	Plrg1	NM_016784.2	Mus musculus pleiotropic regulator 1, PRL1 homolog (Arabidopsis) (Plrg1), mRNA.
scl30807.22.1_70-S	39.3027	1.9	Mrvi1	NM_194464.1	Mus musculus MRV integration site 1 (Mrvi1), transcript variant 1, mRNA.
scl0002511.1_1272-S	39.2211	1.37	Myh9	NM_022410.1	Mus musculus myosin heavy chain IX (Myh9), mRNA.
scl48225.31_410-S	39.018	1.7	Tiam1	NM_009384.1	Mus musculus T-cell lymphoma invasion and metastasis 1 (Tiam1), mRNA.
scl27010.17_214-S	38.9831	1.5	Pscd3	NM_011182.2	Mus musculus pleckstrin homology, Sec7 and coiled-coil domains 3 (Pscd3), mRNA.
scl022289.28_11-S	38.7206	1.68	Utx	NM_009483.1	Mus musculus ubiquitously transcribed tetratricopeptide repeat gene, X chromosome (Utx), mRNA.
scl017974.6_299-S	38.6845	1.8	Nck2	NM_010879.2	Mus musculus non-catalytic region of tyrosine kinase adaptor protein 2 (Nck2), mRNA.
scl0319191.1_321-S	38.6669	1.58	Hist1h2ai	NM_178182	Mus musculus histone 1, H2ai (Hist1h2ai), mRNA.
scl069583.1_127-S	38.6333	1.67	Tnfsf13	NM_023517.1	Mus musculus tumor necrosis factor (ligand) superfamily, member 13 (Tnfsf13), mRNA.
scl0237898.1_282-S	38.6203	1.57	6430526O11Rik	XM_110937.4	
scl32043.3_256-S	38.5879	1.5	AI467606	NM_178901.2	Mus musculus expressed sequence AI467606 (AI467606), mRNA.
scl027556.1_96-S	38.5816	1.37	Clic4	XM_124389.1	Mus musculus chloride intracellular channel 4 (mitochondrial) (Clic4), mRNA.
scl0003137.1_53-S	38.5421	1.46	Drbp1	NM_178090.2	Mus musculus developmentally regulated RNA binding protein 1 (Drbp1), mRNA.
scl0029809.2_171-S	38.4985	1.68	Rabgap1l	NM_013862.3	
scl00234684.2_208-S	38.2479	3.72	Lrrc29	NM_177449.2	
scl0011983.1_209-S	38.138	1.47	Atpif1	NM_007512.2	
scl019166.8_87-S	38.0017	1.36	Psma2	NM_008944.1	Mus musculus proteasome (prosome, macropain) subunit, alpha type 2 (Psma2), mRNA.
scl28755.11.1_30-S	37.9235	1.8	Dusp11	NM_028099.2	Mus musculus dual specificity phosphatase 11 (RNA/RNP complex 1-interacting) (Dusp11), mRNA.
scl40317.1.1637_8-S	37.8869	4.1	4930527B16Rik	XM_147531.1	Mus musculus RIKEN cDNA 4930527B16 gene (4930527B16Rik), mRNA.
scl0026554.2_150-S	37.8481	1.44	Cul3	NM_016716.2	Mus musculus cullin 3 (Cul3), mRNA.
scl012385.18_225-S	37.8214	1.38	Catna1	NM_009818.1	Mus musculus catenin alpha 1 (Catna1), mRNA.
scl020170.2_257-S	37.6894	1.84	Hps6	NM_176785.1	Mus musculus Hermansky-Pudlak syndrome 6 (Hps6), mRNA.
scl0066884.2_148-S	37.5258	1.46	Appbp2	NM_025825.2	Mus musculus amyloid beta precursor protein (cytoplasmic tail) binding protein 2 (Appbp2), mRNA.
scl21638.11_586-S	37.4305	1.9	B430201A12Rik	XM_283903.2	
scl40985.2.1_86-S	37.4062	3.11	Hoxb4	NM_010459	Mus musculus homeo box B4 (Hoxb4), mRNA.
scl0002699.1_4-S	37.3796	1.71	Nol6	NM_139237.1	Mus musculus nucleolar protein family 6 (RNA-associated) (Nol6), transcript variant alpha, mRNA.
scl0002899.1_69-S	37.3175	1.55	Utx	NM_009483.1	Mus musculus ubiquitously transcribed tetratricopeptide repeat gene, X chromosome (Utx), mRNA.
scl21336.8_145-S	37.2158	1.44	3110001A13Rik	NM_025626.3	Mus musculus RIKEN cDNA 3110001A13 gene (3110001A13Rik), mRNA.
scl37393.11_210-S	37.1961	1.43	Pip5k2c	NM_054097.2	Mus musculus phosphatidylinositol-4-phosphate 5-kinase, type II, gamma (Pip5k2c), mRNA.
scl0237943.1_29-S	37.1517	1.77	5430405G24Rik	XM_152907.3	Mus musculus RIKEN cDNA 5430405G24 gene (5430405G24Rik), mRNA.

scl0021871.2_111-S	36.9407	1.54	Atp6v0a2	NM_011596.1	Mus musculus ATPase, H+ transporting, lysosomal V0 subunit a isoform 2 (Atp6v0a2), mRNA.
scl47044.6.24_75-S	36.9376	1.47	0610041B22Rik	NM_025340.1	Mus musculus RIKEN cDNA 0610041B22 gene (0610041B22Rik), mRNA.
scl0022195.1_278-S	36.8843	1.44	Ube2l3	XM_148568.1	Mus musculus ubiquitin-conjugating enzyme E2L 3 (Ube2l3), mRNA.
scl53403.26.13_76-S	36.8715	1.74	Bscl2	NM_008144.3	Mus musculus Bernardinelli-Seip congenital lipodystrophy 2 homolog (human) (Bscl2), mRNA.
scl00231841.2_319-S	36.7454	1.58	AA881470	NM_181066.1	Mus musculus EST AA881470 (AA881470), mRNA.
scl32464.22.1_107-S	36.6552	1.68	Pde8a	NM_008803.1	Mus musculus phosphodiesterase 8A (Pde8a), mRNA.
scl29492.60.1_120-S	36.6185	2.31	Vwf	NM_011708.2	Mus musculus Von Willebrand factor homolog (Vwf), mRNA.
scl23528.23_0-S	36.5752	1.42	Mfn2	NM_133201.1	Mus musculus mitofusin 2 (Mfn2), mRNA.
scl41519.8_316-S	36.5072	1.48	BC003251	NM_024480.2	Mus musculus cDNA sequence BC003251 (BC003251), mRNA.
scl34568.25_184-S	36.3823	2.17	BC016188	NM_145970.1	Mus musculus cDNA sequence BC016188 (BC016188), mRNA.
scl27950.14.1_172-S	36.3612	1.51	Slc4a1ap	NM_009206.1	Mus musculus solute carrier family 4 (anion exchanger), member 1, adaptor protein (Slc4a1ap), mRNA.
scl067160.10_33-S	36.3587	1.46	Eef1g	NM_026007	Mus musculus eukaryotic translation elongation factor 1 gamma (Eef1g), mRNA.
scl35462.25_609-S	36.339	1.55	Pik3cb	NM_029094.1	Mus musculus phosphatidylinositol 3-kinase, catalytic, beta polypeptide (Pik3cb), mRNA.
scl0320365.12_9-S	36.242	1.93	9330186A19Rik	NM_178781.2	Mus musculus RIKEN cDNA 9330186A19 gene (9330186A19Rik), mRNA.
scl00170459.2_123-S	36.2337	1.67	Stard4	NM_133774.2	Mus musculus StAR-related lipid transfer (START) domain containing 4 (Stard4), mRNA.
scl065111.1_137-S	36.2292	1.38	Dap3	NM_022994.2	Mus musculus death associated protein 3 (Dap3), mRNA.
scl0021453.2_208-S	36.0955	1.76	Tcof1	NM_011552.1	Mus musculus Treacher Collins Franceschetti syndrome 1, homolog (Tcof1), mRNA.
scl071435.1_313-S	36.0872	1.64	Arhgap21	XM_130033.5	
scl20092.24_625-S	35.9915	2.14	Dnmt3b	NM_010068.1	Mus musculus DNA methyltransferase 3B (Dnmt3b), mRNA.
scl26742.12.1_68-S	35.9736	1.44	Eif2b4	NM_010122.1	Mus musculus eukaryotic translation initiation factor 2B, subunit 4 delta (Eif2b4), mRNA.
scl49001.3_229-S	35.8738	1.36	Cggbp1	NM_178647.2	Mus musculus CGG triplet repeat binding protein 1 (Cggbp1), mRNA.
scl0380773.4_2-S	35.8566	1.43	1810035L17Rik	XM_354684.1	
scl022327.6_246-S	35.8128	1.43	Vbp1	NM_011692.1	Mus musculus von Hippel-Lindau binding protein 1 (Vbp1), mRNA.
scl17781.7.1_157-S	35.6325	1.88	D1Ertd161e	NM_026187.3	Mus musculus DNA segment, Chr 1, ERATO Doi 161, expressed (D1Ertd161e), mRNA.
scl072462.6_25-S	35.629	1.89	2600005C20Rik	NM_028244.1	Mus musculus RIKEN cDNA 2600005C20 gene (2600005C20Rik), mRNA.
scl17443.8_550-S	35.6235	1.48	Arl10b	NM_026823.1	
scl32698.6.1_30-S	35.6098	1.54	Irf3	NM_016849.2	Mus musculus interferon regulatory factor 3 (Irf3), mRNA.
scl53380.11_494-S	35.5973	1.39	Fads1	NM_146094.1	Mus musculus fatty acid desaturase 1 (Fads1), mRNA.
scl34564.9_192-S	35.5919	2.26	4930527D15Rik	NM_026350.1	Mus musculus RIKEN cDNA 4930527D15 gene (4930527D15Rik), mRNA.
scl0110253.14_323-S	35.4333	1.62	Triobp	NM_138579.2	Mus musculus TRIO and F-actin binding protein (Triobp), mRNA.
scl27959.12_30-S	35.3917	1.67	Snx17	NM_153680.1	Mus musculus sorting nexin 17 (Snx17), mRNA.
scl059287.1_209-S	35.384	1.46	Ncstn	NM_021607.2	Mus musculus nicastrin (Ncstn), mRNA.
scl00217692.2_267-S	35.348	1.93	Sipa1l1	NM_172579.1	Mus musculus signal-induced proliferation-associated 1 like 1 (Sipa1l1), mRNA.
scl17218.7.1_11-S	35.2882	2.49	Slamf1	NM_013730.2	Mus musculus signaling lymphocytic activation molecule family member 1 (Slamf1), mRNA.
scl50907.6_346-S	35.2475	2.4	Sfrs3	NM_013663.3	Mus musculus splicing factor, arginine/serine-rich 3 (SRp20) (Sfrs3), mRNA.
scl37022.6.1_3-S	35.0882	1.71	Cd3d	NM_013487.1	Mus musculus CD3 antigen, delta polypeptide (Cd3d), mRNA.
scl0002648.1_0-S	35.0834	1.59	Mllt3	NM_027326.2	Mus musculus myeloid/lymphoid or mixed lineage-leukemia translocation to 3 homolog (Drosophila) (Mllt3), mRNA.
scl027883.1_63-S	35.0671	1.84	D16H22S680E	XM_147173.1	Mus musculus DNA segment, Chr 16, human D22S680E, expressed (D16H22S680E), mRNA.
scl072722.1_216-S	35.0625	1.44	2810405J04Rik	NM_133747.1	Mus musculus RIKEN cDNA 2810405J04 gene (2810405J04Rik), mRNA.
scl52858.1.11_122-S	34.9618	1.73	Znhit2	NM_013859.1	
scl53012.17_160-S	34.9524	1.63	AI450540	NM_145505.2	Mus musculus expressed sequence AI450540 (AI450540), mRNA.
scl000567.1_25-S	34.943	1.46	Clcn3	NM_007711.1	Mus musculus chloride channel 3 (Clcn3), transcript variant a, mRNA.
scl068114.7_83-S	34.9218	1.42	Mum1	NM_023431.3	
scl41579.7_169-S	34.864	1.41	Ppp2ca	NM_019411.2	Mus musculus protein phosphatase 2a, catalytic subunit, alpha isoform (Ppp2ca), mRNA.
scl013244.1_23-S	34.7552	1.36	Degs	NM_007853.2	Mus musculus degenerative spermatocyte homolog (Drosophila) (Degs), mRNA.
scl37305.10.1_6-S	34.7092	11.5	Mmp10	NM_019471.1	Mus musculus matrix metalloproteinase 10 (Mmp10), mRNA.
scl27956.4.5_26-S	34.6919	2.49	Krtcap3	NM_027221.1	Mus musculus keratinocyte associated protein 3 (Krtcap3), mRNA.
scl25758.6.1_14-S	34.6703	1.85	1200006F02Rik	NM_027872.1	Mus musculus RIKEN cDNA 1200006F02 gene (1200006F02Rik), mRNA.
scl22088.5.1_88-S	34.6446	2.07	Lxn	NM_016753.2	Mus musculus latexin (Lxn), mRNA.
scl0072313.2_218-S	34.5173	1.39	2510002A14Rik	NM_028194.1	Mus musculus RIKEN cDNA 2510002A14 gene (2510002A14Rik), mRNA.
scl0023821.2_49-S	34.4812	1.72	Bace1	NM_011792.3	Mus musculus beta-site APP cleaving enzyme 1 (Bace1), mRNA.
scl39328.7_317-S	34.4178	1.38	Grb2	NM_008163.2	Mus musculus growth factor receptor bound protein 2 (Grb2), mRNA.
scl30429.2.1_68-S	34.4056	2.22	Gng11	NM_025331.2	Mus musculus guanine nucleotide binding protein (G protein), gamma 11 (Gng11), mRNA.
scl0069080.2_160-S	34.3123	1.55	Gmppa	NM_133708.1	Mus musculus GDP-mannose pyrophosphorylase A (Gmppa), mRNA.
scl54952.8_631-S	34.2076	1.58	1200013B08Rik	NM_028773.2	Mus musculus RIKEN cDNA 1200013B08 gene (1200013B08Rik), mRNA.
scl0215449.1_111-S	34.0702	1.38	Rap1b	NM_024457.1	Mus musculus RAS related protein 1b (Rap1b), mRNA.

scl0170716.1_183-S	34.0682	2.2	Cyp4f13	NM_130882.1	Mus musculus cytochrome P450, family 4, subfamily f, polypeptide 13 (Cyp4f13), mRNA.
scl25693.5.1_159-S	34.0357	3.33	Chd7	XM_149413.3	
scl49743.39.1_15-S	34.0239	1.4	C3	NM_009778.1	Mus musculus complement component 3 (C3), mRNA.
scl0004095.1_33-S	33.9746	4.44	Zcchc8	NM_027494.1	Mus musculus zinc finger, CCHC domain containing 8 (Zcchc8), mRNA.
scl0002578.1_9-S	33.9544	1.34	Eif3s6ip	NM_145139.1	Mus musculus eukaryotic translation initiation factor 3, subunit 6 interacting protein (Eif3s6ip), mRNA.
scl43441.25.1_42-S	33.9522	1.87	Dtnb	NM_007886.1	Mus musculus dystrobrevin, beta (Dtnb), mRNA.
scl0228545.7_132-S	33.9376	1.68	Vps18	NM_172269.2	
scl0057874.1_260-S	33.8098	1.35	AW742319	NM_021345.1	Mus musculus expressed sequence AW742319 (AW742319), mRNA.
scl00234797.2_307-S	33.7611	2.07	6430548M08Rik	NM_172286.2	Mus musculus RIKEN cDNA 6430548M08 gene (6430548M08Rik), mRNA.
scl39652.23.1463_24-S	33.6425	1.36	Kpnb1	NM_008379.2	Mus musculus karyopherin (importin) beta 1 (Kpnb1), mRNA.
scl48709.7_108-S	33.5513	1.37	Ube2l3	XM_148568.1	Mus musculus ubiquitin-conjugating enzyme E2L 3 (Ube2l3), mRNA.
scl0217893.6_312-S	33.5411	1.41	6720425G15Rik	NM_198165.1	Mus musculus RIKEN cDNA 6720425G15 gene (6720425G15Rik), mRNA.
scl46964.12_425-S	33.4695	1.4	4732495E13Rik	XM_128300.4	Mus musculus RIKEN cDNA 4732495E13 gene (4732495E13Rik), mRNA.
scl0072193.1_269-S	33.459	1.61	Sfrs2ip	XM_128178.5	
scl50686.4.1_41-S	33.423	1.62	Mrpl14	NM_026732.2	
scl38701.12.1_200-S	33.412	1.84	Arid3a	NM_007880.1	Mus musculus AT rich interactive domain 3A (Bright like) (Arid3a), mRNA.
scl056292.1_247-S	33.4	1.37	Ard1	NM_019870.1	Mus musculus N-acetyltransferase ARD1 homolog (S. cerevisiae) (Ard1), mRNA.
scl0004165.1_41-S	33.323	8.21	Klhl5	NM_175174.2	Mus musculus kelch-like 5 (Drosophila) (Klhl5), mRNA.
scl17047.9.4_6-S	33.3014	1.93	BC013667	NM_172266.1	Mus musculus cDNA sequence BC013667 (BC013667), mRNA.
scl00252972.1_246-S	33.2927	1.53	Tpcn1	NM_145853.2	Mus musculus two pore channel 1 (Tpcn1), mRNA.
scl0003409.1_16-S	33.2884	1.59	A330080J22Rik	NM_178667.2	Mus musculus RIKEN cDNA A330080J22 gene (A330080J22Rik), mRNA.
scl0067684.1_211-S	33.2744	1.35	3300001P08Rik	NM_026313.1	Mus musculus RIKEN cDNA 3300001P08 gene (3300001P08Rik), mRNA.
scl28480.7_231-S	33.274	1.43	Adipor2	NM_197985.2	
scl55078.9.1_18-S	33.2587	2.64	Lmo6	NM_175097.2	Mus musculus LIM domain only 6 (Lmo6), mRNA.
scl32922.5.1_20-S	33.2557	1.71	BC028440	NM_172148.1	Mus musculus cDNA sequence BC028440 (BC028440), mRNA.
scl22978.7.1_18-S	33.2457	2.08	Krtcap2	NM_025327.1	Mus musculus keratinocyte associated protein 2 (Krtcap2), mRNA.
scl00101502.1_299-S	33.114	1.72	Hsd3b7	NM_133943.1	Mus musculus hydroxy-delta-5-steroid dehydrogenase, 3 beta- and steroid delta-isomerase 7 (Hsd3b7), mRNA.
scl0002028.1_25-S	33.1008	1.36	2310066N05Rik	NM_027371.1	Mus musculus RIKEN cDNA 2310066N05 gene (2310066N05Rik), mRNA.
scl0002675.1_35-S	33.0966	1.59	Nasp	NM_016777.2	Mus musculus nuclear autoantigenic sperm protein (histone-binding) (Nasp), mRNA.
scl0002734.1_23-S	33.0713	1.69	Ssbp3	NM_198438.1	Mus musculus single-stranded DNA binding protein 3 (Ssbp3), transcript variant 2, mRNA.
scl00229211.2_16-S	33.0213	2.6	Acad9	NM_172678.2	Mus musculus acyl-Coenzyme A dehydrogenase family, member 9 (Acad9), mRNA.
scl19579.2_109-S	33.0203	1.45	A830007P12Rik	NM_146115.2	Mus musculus RIKEN cDNA A830007P12 gene (A830007P12Rik), mRNA.
scl0067238.2_233-S	32.9767	1.34	2810453I06Rik	NM_026050.1	Mus musculus RIKEN cDNA 2810453I06 gene (2810453I06Rik), mRNA.
scl0054613.2_126-S	32.94	1.4	Siat10	NM_018784.1	Mus musculus sialyltransferase 10 (alpha-2,3-sialyltransferase VI) (Siat10), mRNA.
scl49488.8_241-S	32.9348	1.62	Zfp263	NM_148924.2	Mus musculus zinc finger protein 263 (Zfp263), mRNA.
scl49959.3.1_23-S	32.9009	1.39	Rpp21	NM_026308.1	Mus musculus ribonuclease P 21kDa subunit (human) (Rpp21), mRNA.
scl18785.23_2-S	32.878	1.42	A930025J12Rik	NM_173734	Mus musculus RIKEN cDNA A930025J12 gene (A930025J12Rik), mRNA.
scl41798.24_462-S	32.8712	1.49	Vps54	NM_139061.2	Mus musculus vacuolar protein sorting 54 (yeast) (Vps54), mRNA.
scl00103850.1_287-S	32.8611	1.68	Nt5m	NM_134029.1	Mus musculus 5,3-nucleotidase, mitochondrial (Nt5m), mRNA.
scl37463.11_46-S	32.8484	1.47	Mdm2	NM_010786.2	Mus musculus transformed mouse 3T3 cell double minute 2 (Mdm2), mRNA.
scl50554.25.1_3-S	32.716	1.85	Ddx11	XM_128714.3	
scl24797.1_325-S	32.6811	1.87	Usp48	NM_028344.1	
scl00224613.2_5-S	32.6023	3.2	E030034P13Rik	NM_153791.1	Mus musculus RIKEN cDNA E030034P13 gene (E030034P13Rik), mRNA.
scl0020452.1_241-S	32.5395	1.48	Siat8d	NM_009183.1	Mus musculus sialyltransferase 8 (alpha-2, 8-sialyltransferase) D (Siat8d), mRNA.
scl0338522.1_21-S	32.5085	2.21	9230115A19Rik	XM_110546.2	
scl27313.11.1_0-S	32.4511	3	Tesc	NM_021344.2	Mus musculus tescalcin (Tesc), mRNA.
scl0240263.2_0-S	32.4414	2.06	Fem1c	NM_173423.1	Mus musculus fem-1 homolog c (C.elegans) (Fem1c), mRNA.
scl49300.12_501-S	32.4173	1.47	St6gal1	NM_145933.2	Mus musculus beta galactoside alpha 2,6 sialyltransferase 1 (St6gal1), mRNA.
scl0056469.2_305-S	32.4155	1.58	Pias1	NM_019663.2	Mus musculus protein inhibitor of activated STAT 1 (Pias1), mRNA.
scl066404.9_27-S	32.4022	1.49	2410001C21Rik	NM_025542.1	Mus musculus RIKEN cDNA 2410001C21 gene (2410001C21Rik), mRNA.
scl056438.5_27-S	32.3511	1.32	Rbx1	NM_019712.2	Mus musculus ring-box 1 (Rbx1), mRNA.
scl067078.2_14-S	32.3321	1.33	1700012G19Rik	NM_025954.2	Mus musculus RIKEN cDNA 1700012G19 gene (1700012G19Rik), mRNA.
scl0012091.2_274-S	32.2767	1.77	Glb1	NM_009752.1	Mus musculus galactosidase, beta 1 (Glb1), mRNA.
scl00214505.2_7-S	32.2551	1.57	AU067744	NM_172529.1	Mus musculus expressed sequence AU067744 (AU067744), mRNA.
scl00236790.1_87-S	32.2108	1.49	6330505F04Rik	NM_172779.1	Mus musculus RIKEN cDNA 6330505F04 gene (6330505F04Rik), mRNA.
scl027418.18_28-S	32.141	1.42	Mkln1	NM_013791.1	Mus musculus muskelin 1, intracellular mediator containing kelch motifs (Mkln1), mRNA.

scl53438.9.1_79-S	32.1245	1.33	Ndufv1	NM_133666.1	Mus musculus NADH dehydrogenase (ubiquinone) flavoprotein 1 (Ndufv1), mRNA.
scl00319161.1_8-S	32.0948	2.74	Hist1h4m	NM_175657	Mus musculus histone 1, H4m (Hist1h4m), mRNA.
scl0067246.1_211-S	32.071	1.42	2810474O19Rik	XM_132966.3	Mus musculus RIKEN cDNA 2810474O19 gene (2810474O19Rik), mRNA.
scl00218294.2_195-S	32.0245	2.08	Cdc14b	NM_172587.2	Mus musculus CDC14 cell division cycle 14 homolog B (S. cerevisiae) (Cdc14b), mRNA.
scl00347722.2_310-S	31.971	1.62	Centg2	NM_178119.2	Mus musculus centaurin, gamma 2 (Centg2), mRNA.
scl074569.1_282-S	31.9656	1.76	Ttc17	NM_183106.1	
scl50670.2.184_270-S	31.9317	2.12	2310039H08Rik	NM_025966.2	Mus musculus RIKEN cDNA 2310039H08 gene (2310039H08Rik), mRNA.
scl0001153.1_10-S	31.9175	12.37	C330016K18Rik	XM_132642.4	Mus musculus RIKEN cDNA C330016K18 gene (C330016K18Rik), mRNA.
scl019720.8_242-S	31.9095	1.34	Trim27	NM_009054	Mus musculus tripartite motif protein 27 (Trim27), mRNA.
scl35960.12_340-S	31.9042	1.45	Tmem24	XM_134795.3	
scl36833.9.1_209-S	31.8809	1.57	Rpl4	NM_024212.2	Mus musculus ribosomal protein L4 (Rpl4), mRNA.
scl40755.5.1_55-S	31.8738	1.78	D11Wsu47e	NM_177777.3	Mus musculus DNA segment, Chr 11, Wayne State University 47, expressed (D11Wsu47e), mRNA.
scl066073.8_269-S	31.8738	1.48	0610040B21Rik	NM_025334.2	Mus musculus RIKEN cDNA 0610040B21 gene (0610040B21Rik), mRNA.
scl37947.10_418-S	31.8661	1.38	Tde2	NM_019760	Mus musculus tumor differentially expressed 2 (Tde2), mRNA.
scl0066706.2_314-S	31.7771	1.4	4733401H18Rik	NM_023247.1	Mus musculus RIKEN cDNA 4733401H18 gene (4733401H18Rik), mRNA.
scl44037.11.1_29-S	31.7663	1.41	Dtnbp1	NM_025772.3	Mus musculus dystrobrevin binding protein 1 (Dtnbp1), mRNA.
scl073341.1_128-S	31.7411	1.59	Arhgef6	NM_152801.1	Mus musculus Rac/Cdc42 guanine nucleotide exchange factor (GEF) 6 (Arhgef6), mRNA.
scl37440.14_650-S	31.7223	1.74	Irak3	NM_028679.2	Mus musculus interleukin-1 receptor-associated kinase 3 (Irak3), mRNA.
scl0076895.2_316-S	31.625	1.84	Bicd2	NM_029791.2	Mus musculus bicaudal D homolog 2 (Drosophila) (Bicd2), mRNA.
scl53173.13_456-S	31.5579	1.5	E430027O22Rik	XM_129248.4	Mus musculus RIKEN cDNA E430027O22 gene (E430027O22Rik), mRNA.
scl28579.2_420-S	31.4804	1.64	Fin14		Mus musculus fibroblast growth factor inducible 14 (Fin14), mRNA.
scl52862.7_705-S	31.4431	1.51	Cdca5	NM_026410.1	
scl0067980.2_203-S	31.3947	1.7	Gnpda2	NM_027681.1	Mus musculus glucosamine-6-phosphate deaminase 2 (Gnpda2), mRNA.
scl0056390.1_292-S	31.234	1.48	Sssca1	NM_020491	Mus musculus Sjogrens syndrome/scleroderma autoantigen 1 homolog (human) (Sssca1), mRNA.
scl059040.12_45-S	31.2234	1.44	Rhot1	NM_021536.5	Mus musculus ras homolog gene family, member T1 (Rhot1), mRNA.
scl39808.15.1_10-S	31.1541	1.7	Aatf	NM_019816.1	Mus musculus apoptosis antagonizing transcription factor (Aatf), mRNA.
scl00212531.2_67-S	31.1082	2.53	Sh3bgrl2	NM_172507.2	
scl000674.1_0-S	31.09	2.4	Itgb1	NM_010578.1	Mus musculus integrin beta 1 (fibronectin receptor beta) (Itgb1), mRNA.
scl47641.14_223-S	31.0687	1.62	D15Ertd781e	NM_145476.2	
scl056529.1_95-S	31.0454	1.32	Spc18	NM_019951.1	Mus musculus signal peptidase complex (Spc18), mRNA.
scl0258771.1_118-S	31.0294	10.9	Olfr473	NM_146775.1	Mus musculus olfactory receptor 473 (Olfr473), mRNA.
scl015441.12_277-S	31.0292	1.32	Hp1bp3	NM_010470.1	Mus musculus heterochromatin protein 1, binding protein 3 (Hp1bp3), mRNA.
scl052397.1_25-S	30.9866	1.63	D5Ertd689e	XM_358356.1	Mus musculus DNA segment, Chr 5, ERATO Doi 689, expressed (D5Ertd689e), mRNA.
scl0002898.1_1-S	30.9643	1.93	Sms	NM_009214	Mus musculus spermine synthase (Sms), mRNA.
scl25152.5.1_78-S	30.9521	1.45	Magoh	NM_010760.1	Mus musculus mago-nashi homolog, proliferation-associated (Drosophila) (Magoh), mRNA.
scl00225358.2_258-S	30.9415	1.7	2610024E20Rik	NM_146084.1	
scl023880.13_4-S	30.9264	1.42	Fyb	NM_011815.1	Mus musculus FYN binding protein (Fyb), mRNA.
scl015384.1_1-S	30.8768	1.3	Hnrpab	NM_010448.2	Mus musculus heterogeneous nuclear ribonucleoprotein A/B (Hnrpab), mRNA.
scl0105841.13_268-S	30.876	1.64	E030003N15Rik	XM_128129.4	Mus musculus RIKEN cDNA E030003N15 gene (E030003N15Rik), mRNA.
scl4899.1.1_252-S	30.8362	11.44	Olfr1188	NM_146919.1	Mus musculus olfactory receptor 1188 (Olfr1188), mRNA.
scl0003304.1_37-S	30.8233	2.29	Xrn2	NM_011917	Mus musculus 5-3 exoribonuclease 2 (Xrn2), mRNA.
scl0066125.2_261-S	30.7812	1.35	Sf3b5	NM_175102.2	
scl30066.5_708-S	30.7511	1.89	6330407D12Rik	NM_175098.2	Mus musculus RIKEN cDNA 6330407D12 gene (6330407D12Rik), mRNA.
scl16332.2_164-S	30.7509	1.38	Cxcr4	NM_009911.2	Mus musculus chemokine (C-X-C motif) receptor 4 (Cxcr4), mRNA.
scl51323.13.1_30-S	30.7304	1.63	Mppe1	NM_172630.1	Mus musculus metallophosphoesterase 1 (Mppe1), mRNA.
scl35560.5_28-S	30.7132	1.64	Cox7a2	NM_009945.2	Mus musculus cytochrome c oxidase, subunit VIIa 2 (Cox7a2), mRNA.
scl0068915.2_300-S	30.6997	2.04	Vars2l	NM_175137.3	Mus musculus valyl-tRNA synthetase 2-like (Vars2l), mRNA.
scl28688.42_395-S	30.4889	1.34	Nup210	NM_018815.1	Mus musculus nucleoporin 210 (Nup210), mRNA.
scl016784.1_5-S	30.4831	1.33	Lamp2	NM_010685.2	Mus musculus lysosomal membrane glycoprotein 2 (Lamp2), mRNA.
scl0231086.16_205-S	30.4698	1.32	Hadhb	NM_145558.1	Mus musculus hydroxyacyl-Coenzyme A dehydrogenase/3-ketoacyl-Coenzyme A thiolase/enoyl-Coenzyme A hydratase (trifunctional protein), beta subunit (Hadhb), mRNA.
scl0023894.2_0-S	30.4657	1.45	Gtf2h2	NM_022011.2	Mus musculus general transcription factor II H, polypeptide 2 (Gtf2h2), mRNA.
scl48556.8.1_18-S	30.4622	1.39	2010319C14Rik	NM_024464.2	Mus musculus RIKEN cDNA 2010319C14 gene (2010319C14Rik), mRNA.
scl52806.22.1_43-S	30.439	1.5	Pitpnm1	NM_008851.1	
scl53451.16.1_293-S	30.3892	3.61	0710001P18Rik	NM_026720.1	Mus musculus RIKEN cDNA 0710001P18 gene (0710001P18Rik), mRNA.
scl0052712.1_238-S	30.3858	1.61	Zfp535	NM_026107.2	Mus musculus zinc finger protein 535 (Zfp535), mRNA.
scl00380608.1_88-S	30.3752	7.08	Tagap	NM_147155	

scl44364.2.1_224-S	30.3199	5.24	C86987	XM_127523.2	Mus musculus expressed sequence C86987 (C86987), mRNA.
scl0225995.2_210-S	30.3167	1.34	D030056L22	NM_177640.2	Mus musculus hypothetical protein D030056L22 (D030056L22), mRNA.
scl40534.12.1_29-S	30.2881	1.55	Tbrg4	NM_134011.1	Mus musculus transforming growth factor beta regulated gene 4 (Tbrg4), mRNA.
scl47060.10.1_13-S	30.2398	1.82	9130210N20Rik	NM_172607.2	Mus musculus RIKEN cDNA 9130210N20 gene (9130210N20Rik), mRNA.
scl0330260.1_61-S	30.1494	1.32	Pon2	NM_183308.1	Mus musculus paraoxonase 2 (Pon2), mRNA.
scl017260.8_51-S	30.0538	1.95	Mef2c	NM_025282.1	Mus musculus myocyte enhancer factor 2C (Mef2c), mRNA.
scl054624.14_263-S	29.9886	1.44	5730511K23Rik	NM_019458.2	Mus musculus RIKEN cDNA 5730511K23 gene (5730511K23Rik), mRNA.
scl32942.2_625-S	29.9763	1.48	Zfp574	NM_175477.2	
scl0016071.1_62-S	29.9544	2.4	Igk-C	XM_132633.4	
scl30665.8.1_14-S	29.927	2.17	1110032O16Rik	XM_133813.4	Mus musculus RIKEN cDNA 1110032O16 gene (1110032O16Rik), mRNA.
scl28535.9.1_27-S	29.9192	1.32	Sec13l1	NM_024206.3	Mus musculus SEC13-like 1 (S. cerevisiae) (Sec13l1), mRNA.
scl44989.1_487-S	29.9185	1.53	Hist1h1c	NM_015786	Mus musculus histone 1, H1c (Hist1h1c), mRNA.
scl37317.9.1_34-S	29.9118	1.37	Casp1	NM_009807.1	Mus musculus caspase 1 (Casp1), mRNA.
scl25760.31.1_106-S	29.8912	1.91	Flt1	NM_010228.2	Mus musculus FMS-like tyrosine kinase 1 (Flt1), mRNA.
scl32898.7.1_80-S	29.8517	1.35	Blvrb	NM_144923	Mus musculus biliverdin reductase B (flavin reductase (NADPH)) (Blvrb), mRNA.
scl0210711.2_41-S	29.7872	1.41	1110007A13Rik	NM_145955.2	Mus musculus RIKEN cDNA 1110007A13 gene (1110007A13Rik), mRNA.
scl056433.4_275-S	29.7847	1.37	Vps29	NM_019780.1	Mus musculus vacuolar protein sorting 29 (S. pombe) (Vps29), mRNA.
scl24870.7.1_13-S	29.7509	2.41	D030015G18Rik	NM_146155.2	Mus musculus RIKEN cDNA D030015G18 gene (D030015G18Rik), mRNA.
scl0019769.2_219-S	29.7307	1.44	Rit1	NM_009069.2	Mus musculus Ras-like without CAAX 1 (Rit1), mRNA.
scl24875.11.1_30-S	29.6102	1.52	Taf12	NM_025579.1	Mus musculus TAF12 RNA polymerase II, TATA box binding protein (TBP)-associated factor (Taf12), mRNA.
scl067596.7_211-S	29.609	2.56	5830405N20Rik	NM_183264.1	Mus musculus RIKEN cDNA 5830405N20 gene (5830405N20Rik), mRNA.
scl39206.7_318-S	29.5892	1.39	BC017643	NM_144832.1	Mus musculus cDNA sequence BC017643 (BC017643), mRNA.
scl026403.1_197-S	29.5541	1.46	Map3k11	NM_022012.2	Mus musculus mitogen activated protein kinase kinase kinase 11 (Map3k11), mRNA.
scl37497.8.1_151-S	29.514	1.42	Glipr1	NM_028608.1	Mus musculus GLI pathogenesis-related 1 (glioma) (Glipr1), mRNA.
scl00320074.1_330-S	29.4872	2.68	Rxrip110	NM_011307.1	
scl0018549.1_176-S	29.3832	9.17	Pcsk2	NM_008792.3	Mus musculus proprotein convertase subtilisin/kexin type 2 (Pcsk2), mRNA.
scl35732.23.1_51-S	29.382	1.42	Kif23	NM_024245	Mus musculus kinesin family member 23 (Kif23), mRNA.
scl50814.22.1_1-S	29.3488	1.36	Crebl1	NM_017406.2	Mus musculus cAMP responsive element binding protein-like 1 (Crebl1), mRNA.
scl21990.8.1_193-S	29.2679	1.42	Prcc	NM_033573.1	Mus musculus papillary renal cell carcinoma (translocation-associated) (Prcc), mRNA.
scl052120.1_108-S	29.2461	1.88	D8Ertd354e	NM_133970	Mus musculus DNA segment, Chr 8, ERATO Doi 354, expressed (D8Ertd354e), mRNA.
scl0067427.2_328-S	29.2078	1.41	Rps20	NM_026147.3	Mus musculus ribosomal protein S20 (Rps20), mRNA.
scl017749.3_11-S	29.1772	1.54	Polr2k	NM_023127	Mus musculus polymerase (RNA) II (DNA directed) polypeptide K (Polr2k), mRNA.
scl0069821.2_297-S	29.1675	1.49	1810059A23Rik	NM_178051.3	
scl014081.20_10-S	29.1665	1.52	Acsl1	NM_007981.2	Mus musculus acyl-CoA synthetase long-chain family member 1 (Acsl1), mRNA.
scl35708.10_49-S	29.1546	2.61	Smad3	NM_016769	
scl50798.28.1_41-S	29.1459	1.57	Vars2	NM_011690.2	Mus musculus valyl-tRNA synthetase 2 (Vars2), mRNA.
scl000076.1_111_REVCO	29.1379	1.52	D11Wsu99e	NM_013581.2	Mus musculus DNA segment, Chr 11, Wayne State University 99, expressed (D11Wsu99e), mRNA.
scl098193.14_109-S	29.1179	1.46	Wdr42a	NM_153555.1	
scl074549.2_261-S	29.0769	1.74	9130404D08Rik	NM_028993.2	Mus musculus RIKEN cDNA 9130404D08 gene (9130404D08Rik), mRNA.
scl013481.4_295-S	29.0001	1.37	Dpm2	NM_010073.2	Mus musculus dolichol-phosphate (beta-D) mannosyltransferase 2 (Dpm2), mRNA.
scl0101095.9_35-S	28.9716	1.58	Zfp282	NM_146175.2	Mus musculus zinc finger protein 282 (Zfp282), mRNA.
scl076071.13_258-S	28.9713	2.14	Gababrbp	NM_178394.2	
scl000348.1_43-S	28.9239	10.56	Rgr	NM_021340.2	Mus musculus retinal G protein coupled receptor (Rgr), mRNA.
scl0215160.4_168-S	28.9195	2.17	Rhbdl7	NM_146002.1	
scl53474.8.1_75-S	28.8759	1.35	Ccs	NM_016892.2	Mus musculus copper chaperone for superoxide dismutase (Ccs), mRNA.
scl019395.3_4-S	28.8611	1.88	Rasgrp2	XM_359299.1	Mus musculus RAS, guanyl releasing protein 2 (Rasgrp2), mRNA.
scl48157.24.1_63-S	28.805	5.78	Wdr9	NM_145125.1	Mus musculus WD repeat domain 9 (Wdr9), mRNA.
scl38001.5.1_73-S	28.7932	1.46	1700021F05Rik	NM_026411.1	Mus musculus RIKEN cDNA 1700021F05 gene (1700021F05Rik), mRNA.
scl0072020.1_140-S	28.7391	2.09	1600021C16Rik	NM_028059.1	Mus musculus RIKEN cDNA 1600021C16 gene (1600021C16Rik), mRNA.
scl53840.18.1_253-S	28.7337	3.56	Sytl4	NM_013757.1	Mus musculus synaptotagmin-like 4 (Sytl4), mRNA.
scl067945.1_102-S	28.7148	1.88	Rpl41	NM_018860.2	Mus musculus ribosomal protein L41 (Rpl41), mRNA.
scl36337.4.1_30-S	28.672	1.31	1500010M16Rik	NM_026892.1	Mus musculus RIKEN cDNA 1500010M16 gene (1500010M16Rik), mRNA.
scl38624.3_492-S	28.6691	2.19	D10Wsu102e	NM_026579.2	Mus musculus DNA segment, Chr 10, Wayne State University 102, expressed (D10Wsu102e), mRNA.
scl28314.7.1_50-S	28.658	1.44	2010012C16Rik	NM_025564.1	Mus musculus RIKEN cDNA 2010012C16 gene (2010012C16Rik), mRNA.
scl00104318.1_298-S	28.6082	1.46	Csnk1d	NM_027874.1	Mus musculus casein kinase 1, delta (Csnk1d), transcript variant 2, mRNA.
scl24019.5_109-S	28.5578	1.39	Cpt2	NM_009949	Mus musculus carnitine palmitoyltransferase 2 (Cpt2), mRNA.

scl41586.4_460-S	28.4947	1.37	D11Ertd497e	NM_029976.2	Mus musculus DNA segment, Chr 11, ERATO Doi 497, expressed (D11Ertd497e), mRNA.
scl072686.1_177-S	28.4487	1.41	Usp24	XM_131566.5	
scl075445.1_91-S	28.3866	5.72	1700008B15Rik	XM_485370	
scl0208117.1_319-S	28.3247	2.41	4632417K02	NM_177583.2	Mus musculus hypothetical protein 4632417K02 (4632417K02), mRNA.
scl54357.9.1_59-S	28.3067	1.95	Pfc	XM_135820.3	Mus musculus properdin factor, complement (Pfc), mRNA.
scl0019699.2_228-S	28.2434	1.81	Reln	NM_011261.1	Mus musculus reelin (Reln), mRNA.
scl072465.7_112-S	28.1599	1.61	Zfp131	NM_028245.1	Mus musculus zinc finger protein 131 (Zfp131), mRNA.
scl020973.4_189-S	28.1414	1.34	Syngr2	NM_009304.1	Mus musculus synaptogyrin 2 (Syngr2), mRNA.
scl40004.19.1_140-S	28.0849	1.4	Acadvl	NM_017366	Mus musculus acyl-Coenzyme A dehydrogenase, very long chain (Acadvl), mRNA.
scl52435.3.1_22-S	28.0848	1.37	Mrpl43	NM_053164.2	Mus musculus mitochondrial ribosomal protein L43 (Mrpl43), nuclear gene encoding mitochondrial protein, mRNA.
scl0001312.1_33-S	28.0461	4.88	Cacna1g	NM_009783.1	Mus musculus calcium channel, voltage-dependent, T type, alpha 1G subunit (Cacna1g), mRNA.
scl0069116.1_159-S	28.0446	2	1810009A16Rik	XM_355528.1	Mus musculus RIKEN cDNA 1810009A16 gene (1810009A16Rik), mRNA.
scl17336.3.1_84-S	28.0306	1.6	2810025M15Rik	NM_027274.2	Mus musculus RIKEN cDNA 2810025M15 gene (2810025M15Rik), mRNA.
scl067414.12_0-S	27.9777	1.5	Mfn1	NM_024200.2	Mus musculus mitofusin 1 (Mfn1), mRNA.
scl33340.4_635-S	27.9075	1.94	Txnl4b	NM_175646.2	
scl0080718.1_88-S	27.9026	3.33	Rab27b	NM_030554.1	Mus musculus RAB27b, member RAS oncogene family (Rab27b), mRNA.
scl44239.8_168-S	27.9017	1.68	Zfp307	NM_023685.2	
scl21186.4.1_111-S	27.8704	1.84	C430004E15Rik	NM_175286.2	Mus musculus RIKEN cDNA C430004E15 gene (C430004E15Rik), mRNA.
scl0107173.1_330-S	27.8704	1.59	AI428855	NM_207220.1	Mus musculus expressed sequence AI428855 (AI428855), mRNA.
scl0068379.1_280-S	27.8634	1.38	Ciz1	NM_028412.1	Mus musculus CDKN1A interacting zinc finger protein 1 (Ciz1), mRNA.
scl19480.4_1-S	27.8178	1.53	Zdhhc12	NM_025428.1	Mus musculus zinc finger, DHHC domain containing 12 (Zdhhc12), mRNA.
scl021858.1_100-S	27.8014	1.45	Timp2	NM_011594.2	Mus musculus tissue inhibitor of metalloproteinase 2 (Timp2), mRNA.
scl39283.5.1_319-S	27.771	2.51	1300017K07Rik	NM_027919.1	Mus musculus RIKEN cDNA 1300017K07 gene (1300017K07Rik), mRNA.
scl00231999.2_275-S	27.752	3.4	BC052360	XM_132552.3	
scl00234023.2_117-S	27.6998	1.32	9430010O03Rik	NM_176849.2	Mus musculus RIKEN cDNA 9430010O03 gene (9430010O03Rik), mRNA.
scl0023845.2_228-S	27.6855	3.09	Clecsf5	NM_021364.1	Mus musculus C-type (calcium dependent, carbohydrate-recognition domain) lectin, superfamily member 5 (Clecsf5), mRNA.
scl50143.3.1_18-S	27.6443	1.56	0610039D01Rik	NM_026307.2	Mus musculus RIKEN cDNA 0610039D01 gene (0610039D01Rik), mRNA.
scl54114.2_230-S	27.6053	5.28	Rab39b	NM_175122.2	Mus musculus RAB39B, member RAS oncogene family (Rab39b), mRNA.
scl0026895.1_34-S	27.6015	1.71	Cops7b	NM_172974.1	Mus musculus COP9 (constitutive photomorphogenic) homolog, subunit 7b (Arabidopsis thaliana) (Cops7b), mRNA.
scl0380791.1_239-S	27.5989	2.13	Igh-VJ558	XM_354700	
scl056772.1_0-S	27.5894	2.24	AI839562	NM_019914.2	Mus musculus expressed sequence AI839562 (AI839562), mRNA.
scl0002306.1_28-S	27.5273	1.61		XM_283061.1	Mus musculus RIKEN cDNA A630086P08 gene (A630086P08Rik), mRNA.
scl0076080.2_277-S	27.4839	1.89	5830472M02Rik	NM_181734.1	Mus musculus RIKEN cDNA 5830472M02 gene (5830472M02Rik), mRNA.
scl00230316.2_241-S	27.444	5.33	Egfl5	NM_172694.1	Mus musculus EGF-like-domain, multiple 5 (Egfl5), mRNA.
scl16458.7.1_28-S	27.4298	1.61	Thap4	NM_025920	Mus musculus THAP domain containing 4 (Thap4), mRNA.
scl40536.24.1_162-S	27.4069	1.36	Myo1g	NM_178440.2	Mus musculus myosin IG (Myo1g), mRNA.
scl51004.4_55-S	27.403	1.74	Sepx1	NM_013759.1	
scl0217558.10_30-S	27.3901	4.92	6030408C04Rik	XM_126912.4	Mus musculus RIKEN cDNA 6030408C04 gene (6030408C04Rik), mRNA.
scl0012725.1_238-S	27.3758	1.44	Clcn3		
scl012464.14_38-S	27.346	1.29	Cct4	NM_009837.1	Mus musculus chaperonin subunit 4 (delta) (Cct4), mRNA.
scl4320.1.1_316-S	27.3385	11.45	Olfr1107	NM_146844.1	Mus musculus olfactory receptor 1107 (Olfr1107), mRNA.
scl00210789.2_87-S	27.3283	6.85	Tbc1d4	NM_173380.1	Mus musculus TBC1 domain family, member 4 (Tbc1d4), mRNA.
scl46811.9.582_26-S	27.3186	1.59	3000003F02Rik	NM_172437.1	Mus musculus RIKEN cDNA 3000003F02 gene (3000003F02Rik), mRNA.
scl0093760.1_64-S	27.3098	1.83	Arid1a	NM_033566.1	Mus musculus AT rich interactive domain 1A (Swi1 like) (Arid1a), mRNA.
scl0056208.2_9-S	27.2681	1.31	Becn1	NM_019584.2	Mus musculus beclin 1 (coiled-coil, myosin-like BCL2-interacting protein) (Becn1), mRNA.
scl28701.8.1_115-S	27.2245	1.49	0710001P09Rik	NM_025351.2	Mus musculus RIKEN cDNA 0710001P09 gene (0710001P09Rik), mRNA.
scl020320.8_193-S	27.1867	1.35	Sdfr1	NM_009145.1	Mus musculus stromal cell derived factor receptor 1 (Sdfr1), mRNA.
scl34424.10_15-S	27.0891	1.71	Tk2	NM_021028.2	Mus musculus thymidine kinase 2, mitochondrial (Tk2), mRNA.
scl066989.6_1-S	27.0059	1.46	2410004N11Rik	NM_025888.2	Mus musculus RIKEN cDNA 2410004N11 gene (2410004N11Rik), mRNA.
scl0002489.1_318-S	26.9846	1.57	4921518A06Rik	NM_198293.1	Mus musculus RIKEN cDNA 4921518A06 gene (4921518A06Rik), mRNA.
scl00226527.1_138-S	26.9528	10.68	BC026585	XM_129546.2	Mus musculus cDNA sequence BC026585 (BC026585), mRNA.
scl19085.2_285-S	26.9438	5.28	Neurod1	NM_010894.1	Mus musculus neurogenic differentiation 1 (Neurod1), mRNA.
scl0223773.9_321-S	26.929	1.45	Zbed4	NM_181412.2	
scl054343.15_6-S	26.9118	1.68	Atf7ip	NM_019426.2	Mus musculus activating transcription factor 7 interacting protein (Atf7ip), mRNA.
scl26735.47.1_66-S	26.8596	1.75	Ift172	NM_026298.4	Mus musculus intraflagellar transport 172 (Ift172), mRNA.
scl022245.1_319-S	26.8503	1.34	Uck1	NM_011675.1	

scl0018010.2_202-S	26.7838	1.42	Neu1	NM_010893.2	Mus musculus neuraminidase 1 (Neu1), mRNA.
scl18004.30_24-S	26.7822	1.47	Tpp2	NM_009418.1	Mus musculus tripeptidyl peptidase II (Tpp2), mRNA.
scl48822.17_669-S	26.764	2.57	D230007K08Rik	NM_175547.2	Mus musculus RIKEN cDNA D230007K08 gene (D230007K08Rik), mRNA.
scl39554.8_358-S	26.7527	1.29	Rab5c	NM_024456	Mus musculus RAB5C, member RAS oncogene family (Rab5c), mRNA.
scl24032.3.148_1-S	26.7308	3.52	2210012G02Rik	NM_025617.1	Mus musculus RIKEN cDNA 2210012G02 gene (2210012G02Rik), mRNA.
scl46589.23.1_15-S	26.7087	1.58	2310021P13Rik	XM_127591.4	
scl0108071.1_257-S	26.6553	10.55	Grm5	XM_149971.3	Mus musculus glutamate receptor, metabotropic 5 (Grm5), mRNA.
scl49695.8_112-S	26.5995	1.34	Vapa	NM_013933.2	Mus musculus vesicle-associated membrane protein, associated protein A (Vapa), mRNA.
scl35398.12.2_77-S	26.5759	2.25	Alas1	NM_020559.1	Mus musculus aminolevulinic acid synthase 1 (Alas1), mRNA.
scl074645.1_117-S	26.569	1.41	4930431B09Rik	XM_207778.3	Mus musculus RIKEN cDNA 4930431B09 gene (4930431B09Rik), mRNA.
scl50582.4.1_9-S	26.5678	1.35	Clpp	NM_017393	Mus musculus caseinolytic protease, ATP-dependent, proteolytic subunit homolog (E. coli) (Clpp), mRNA.
scl0017314.1_97-S	26.5647	1.77	Mgmt	NM_008598.1	Mus musculus O-6-methylguanine-DNA methyltransferase (Mgmt), mRNA.
scl00268996.2_185-S	26.5559	1.34	Ss18	NM_009280.1	Mus musculus synovial sarcoma translocation, Chromosome 18 (Ss18), mRNA.
scl16390.7_3-S	26.5455	1.55	Ralb	NM_022327.3	Mus musculus v-ral simian leukemia viral oncogene homolog B (ras related) (Ralb), mRNA.
scl28793.10_326-S	26.5139	1.63	Stambp	NM_024239.1	Mus musculus Stam binding protein (Stambp), mRNA.
scl49146.12.1_4-S	26.4737	1.54	2610033C09Rik	NM_026407.2	Mus musculus RIKEN cDNA 2610033C09 gene (2610033C09Rik), mRNA.
scl54140.7.1_25-S	26.4585	1.41	Dnase1l1	NM_027109.1	
scl47524.3.1_1-S	26.4522	1.33	2310016M24Rik	NM_183256.1	Mus musculus RIKEN cDNA 2310016M24 gene (2310016M24Rik), mRNA.
scl18972.12.1_41-S	26.4484	1.46	1810020C19Rik	XM_130317.5	Mus musculus RIKEN cDNA 1810020C19 gene (1810020C19Rik), mRNA.
scl00105348.2_131-S	26.3898	1.35	Golph2	NM_027307.1	Mus musculus golgi phosphoprotein 2 (Golph2), mRNA.
scl22690.20.1_0-S	26.3862	4.03	2810453L12Rik	XM_131155.3	Mus musculus RIKEN cDNA 2810453L12 gene (2810453L12Rik), mRNA.
scl050850.17_73-S	26.3763	1.6	Spg4	NM_016962.1	Mus musculus spastic paraplegia 4 homolog (human) (Spg4), mRNA.
scl30706.6.2_3-S	26.3077	10.94	9430065N20Rik	NM_175435.2	Mus musculus RIKEN cDNA 9430065N20 gene (9430065N20Rik), mRNA.
scl27465.5_88-S	26.2784	3.62	6330405H19	XM_358637.1	
scl18013.4_456-S	26.2327	1.52	AI597479	NM_133818.1	Mus musculus expressed sequence AI597479 (AI597479), mRNA.
scl53028.7_552-S	26.2012	1.62	Trim8	NM_053100.1	Mus musculus tripartite motif protein 8 (Trim8), mRNA.
scl24398.4.1_27-S	26.1339	1.68	Hint2	XM_143732.2	
scl36496.9.1_37-S	26.1275	1.5	Tusc4	NM_018879.1	Mus musculus tumor suppressor candidate 4 (Tusc4), mRNA.
scl20535.2.1_122-S	26.0785	8.81	A930018P22Rik	NM_026634.1	Mus musculus RIKEN cDNA A930018P22 gene (A930018P22Rik), mRNA.
scl30062.4.1_1-S	26.0724	2.49	Npy	NM_023456.2	Mus musculus neuropeptide Y (Npy), mRNA.
scl00100169.1_330-S	26.0261	1.43	Phactr4	NM_175306.2	
scl52860.17_315-S	25.9969	1.5	Syvn1	NM_028769.4	
scl36111.9.1_0-S	25.9928	1.37	Rp9h	NM_018739.1	Mus musculus retinitis pigmentosa 9 homolog (human) (Rp9h), mRNA.
scl016568.8_47-S	25.9671	2.07	Kif3a	NM_008443.2	Mus musculus kinesin family member 3A (Kif3a), mRNA.
scl00104681.2_288-S	25.9189	1.43	Slc16a6	NM_134038.1	Mus musculus solute carrier family 16 (monocarboxylic acid transporters), member 6 (Slc16a6), mRNA.
scl0067384.1_276-S	25.909	1.67	Bag4	NM_026121.1	Mus musculus BCL2-associated athanogene 4 (Bag4), mRNA.
scl0075991.1_191-S	25.8735	1.52	5033405K12Rik	NM_153567.1	Mus musculus RIKEN cDNA 5033405K12 gene (5033405K12Rik), mRNA.
scl36214.3_394-S	25.8562	1.76	Gbif	NM_019683.2	Mus musculus globin inducing factor, fetal (Gbif), mRNA.
scl0078655.1_180-S	25.8508	1.37	Eif3s1	NM_144545.1	Mus musculus eukaryotic translation initiation factor 3, subunit 1 alpha (Eif3s1), mRNA.
scl37753.17.1_5-S	25.8166	1.65	Polrmt	NM_172551.1	Mus musculus polymerase (RNA) mitochondrial (DNA directed) (Polrmt), mRNA.
scl28438.14_163-S	25.8082	2.03	Slc2a3	NM_011401.2	Mus musculus solute carrier family 2 (facilitated glucose transporter), member 3 (Slc2a3), mRNA.
scl019068.1_323-S	25.792	1.27	Erh	NM_007951.1	Mus musculus enhancer of rudimentary homolog (Drosophila) (Erh), mRNA.
scl36751.1.1150_295-S	25.7776	6.99	4833444G19Rik	NM_177062.2	Mus musculus RIKEN cDNA 4833444G19 gene (4833444G19Rik), mRNA.
scl46038.17.1_26-S	25.7681	1.47	Tdrd3	NM_172605.2	Mus musculus tudor domain containing 3 (Tdrd3), mRNA.
scl067938.1_193-S	25.6473	1.29	Mylc2b	NM_023402.1	
scl23525.7_662-S	25.623	1.6	Agtrap	NM_009642.3	Mus musculus angiotensin II, type I receptor-associated protein (Agtrap), mRNA.
scl46956.4_124-S	25.6174	1.32	1300006C06Rik	NM_028792.1	Mus musculus RIKEN cDNA 1300006C06 gene (1300006C06Rik), mRNA.
scl17803.10.1_30-S	25.6145	1.27	Arpc2	XM_129773.5	Mus musculus actin related protein 2/3 complex, subunit 2 (Arpc2), mRNA.
scl38366.14_414-S	25.5803	1.39	Gns	NM_029364.1	Mus musculus glucosamine (N-acetyl)-6-sulfatase (Gns), mRNA.
scl00215708.2_187-S	25.5654	4.7	C030011O14Rik	NM_174868.2	Mus musculus RIKEN cDNA C030011O14 gene (C030011O14Rik), mRNA.
scl066151.5_253-S	25.4838	1.27	1110020C13Rik	NM_025385.2	Mus musculus RIKEN cDNA 1110020C13 gene (1110020C13Rik), mRNA.
scl49475.21_477-S	25.4338	1.4	Mgrn1	NM_029657.2	Mus musculus mahogunin, ring finger 1 (Mgrn1), mRNA.
scl0078334.1_222-S	25.4334	1.53	2700084L06Rik	NM_198164.1	Mus musculus RIKEN cDNA 2700084L06 gene (2700084L06Rik), mRNA.
scl0001454.1_1188-S	25.3685	9.61	B230396K10Rik	XM_205737.3	Mus musculus RIKEN cDNA B230396K10 gene (B230396K10Rik), mRNA.
scl00110160.1_313-S	25.3564	1.71	Tcte3	NM_011560	Mus musculus t-complex-associated testis expressed 3 (Tcte3), mRNA.
scl0067500.2_43-S	25.3388	1.37	Ccar1	NM_026201.1	Mus musculus cell division cycle and apoptosis regulator 1 (Ccar1), mRNA.

scl24886.14_14-S	25.2863	1.45	Laptm5	NM_010686.2	Mus musculus lysosomal-associated protein transmembrane 5 (Laptm5), mRNA.
scl36385.5_13-S	25.2803	1.36	Cklfsf6	NM_026036.1	Mus musculus chemokine-like factor super family 6 (Cklfsf6), mRNA.
scl25834.12_533-S	25.2677	1.8	Snx8	NM_172277.1	Mus musculus sorting nexin 8 (Snx8), mRNA.
scl33425.15_229-S	25.2532	1.49	D230025D16Rik	NM_145604.1	
scl26088.14.1_16-S	25.2286	1.47	Aldh2	NM_009656.1	Mus musculus aldehyde dehydrogenase 2, mitochondrial (Aldh2), mRNA.
scl45300.23_484-S	25.2076	1.52	Cog3	NM_177381	Mus musculus component of oligomeric golgi complex 3 (Cog3), mRNA.
scl44903.11.3_96-S	25.2076	1.43	Fars1	NM_024274.1	Mus musculus phenylalanine-tRNA synthetase 1 (mitochondrial) (Fars1), mRNA.
scl066448.4_32-S	25.1857	1.29	Mrpl20	NM_025570.1	Mus musculus mitochondrial ribosomal protein L20 (Mrpl20), mRNA.
scl32577.4.1_30-S	25.1614	1.64	Mcee	XM_133510.4	
scl45706.11_2-S	25.1452	1.31	Ghitm	NM_078478.1	Mus musculus growth hormone inducible transmembrane protein (Ghitm), mRNA.
scl0227929.1_2-S	25.141	1.46	Pscdbp	NM_139200.2	Mus musculus pleckstrin homology, Sec7 and coiled-coil domains, binding protein (Pscdbp), mRNA.
scl0021339.1_180-S	25.0683	2.56	Taf1a	NM_021466.1	Mus musculus TATA box binding protein (Tbp)-associated factor, RNA polymerase I, A (Taf1a), mRNA.
scl066726.1_64-S	25.0645	1.49	4921518A06Rik	NM_198293.1	Mus musculus RIKEN cDNA 4921518A06 gene (4921518A06Rik), mRNA.
scl24219.2.1_21-S	25.0456	1.29	3110001D03Rik	NM_025849.2	Mus musculus RIKEN cDNA 3110001D03 gene (3110001D03Rik), mRNA.
scl50221.17.1_145-S	25.0231	1.42	Pdpk1	NM_011062.1	Mus musculus 3-phosphoinositide dependent protein kinase-1 (Pdpk1), mRNA.
scl0319182.1_4-S	25.0038	2.23	Hist1h2bh	NM_178197.1	Mus musculus histone 1, H2bh (Hist1h2bh), mRNA.
scl23461.27_162-S	24.9766	1.5	Kcnab2	NM_010598.2	Mus musculus potassium voltage-gated channel, shaker-related subfamily, beta member 2 (Kcnab2), mRNA.
scl068735.6_63-S	24.9754	1.32	Mrps18c	XM_194230.3	
scl0019055.2_130-S	24.9327	1.34	Ppp3ca	NM_008913.1	Mus musculus protein phosphatase 3, catalytic subunit, alpha isoform (Ppp3ca), mRNA.
scl0066704.2_280-S	24.9247	1.77	4921506I22Rik	NM_025717.2	Mus musculus RIKEN cDNA 4921506I22 gene (4921506I22Rik), mRNA.
scl0217039.1_54-S	24.909	1.29	Ggnbp2	NM_153144.1	
scl42993.1.28_269-S	24.881	1.36	2410016O06Rik	NM_023633	Mus musculus RIKEN cDNA 2410016O06 gene (2410016O06Rik), mRNA.
scl41169.1_6-S	24.8772	1.98	Cdk5r1	NM_009871.2	
scl0068796.2_313-S	24.8722	1.59	1110039B18Rik	NM_144525.2	Mus musculus RIKEN cDNA 1110039B18 gene (1110039B18Rik), mRNA.
scl0018728.1_38-S	24.8535	1.96	Pira5		
scl0234385.1_330-S	24.8299	1.47	Mast3	NM_199308.1	
scl20945.14_483-S	24.7958	1.41	Epc2	NM_172663.2	
scl0002340.1_98-S	24.7938	1.83	Nin	NM_008697	Mus musculus ninein (Nin), mRNA.
scl39226.2.178_25-S	24.7925	6.12	1110012N22Rik	XM_126634.3	Mus musculus RIKEN cDNA 1110012N22 gene (1110012N22Rik), mRNA.
scl50807.7.1_59-S	24.7832	1.62	Dom3z	NM_033613.1	Mus musculus DOM-3 homolog Z (C. elegans) (Dom3z), mRNA.
scl070302.4_170-S	24.7529	1.93	3110050K21Rik	XM_127780.4	Mus musculus RIKEN cDNA 3110050K21 gene (3110050K21Rik), mRNA.
scl29775.9_427-S	24.7191	1.79	Gata2	NM_008090.3	Mus musculus GATA binding protein 2 (Gata2), mRNA.
scl0099650.1_60-S	24.7172	1.66	4933434E20Rik	NM_025762.1	Mus musculus RIKEN cDNA 4933434E20 gene (4933434E20Rik), mRNA.
scl00214084.2_303-S	24.7118	4.48	Slc18a2	NM_172523.1	Mus musculus solute carrier family 18 (vesicular monoamine), member 2 (Slc18a2), mRNA.
scl39478.12_629-S	24.7036	1.91	Plekhm1	NM_183034.1	Mus musculus pleckstrin homology domain containing, family M (with RUN domain) member 1 (Plekhm1), mRNA.
scl27123.13.129_18-S	24.6818	1.79	Dtx2	NM_023742.1	Mus musculus deltex 2 homolog (Drosophila) (Dtx2), mRNA.
scl015122.2_7-S	24.6435	2.42	Hba-a1	NM_008218.1	Mus musculus hemoglobin alpha, adult chain 1 (Hba-a1), mRNA.
scl47694.7_354-S	24.6344	2.06	2310042L06Rik	NM_172428.1	Mus musculus RIKEN cDNA 2310042L06 gene (2310042L06Rik), mRNA.
scl40178.5_602-S	24.6289	1.77	Zfp39	NM_011758.1	Mus musculus zinc finger protein 39 (Zfp39), mRNA.
scl0230789.1_243-S	24.5818	1.44	BC008163	NM_145553.1	Mus musculus cDNA sequence BC008163 (BC008163), mRNA.
scl066193.1_138-S	24.5732	1.45	1110049F12Rik	NM_025411.2	Mus musculus RIKEN cDNA 1110049F12 gene (1110049F12Rik), mRNA.
scl43280.11_443-S	24.5716	1.47	Ankmy2	NM_146033.2	
scl31056.12.1_57-S	24.5606	1.33	Eed	NM_021876.1	Mus musculus embryonic ectoderm development (Eed), mRNA.
scl0226594.1_266-S	24.5283	1.37	BC025872	NM_178593.2	Mus musculus cDNA sequence BC025872 (BC025872), mRNA.
scl0319156.1_0-S	24.5273	3.17	Hist1h4d	NM_175654.1	Mus musculus histone 1, H4d (Hist1h4d), mRNA.
scl37604.17_345-S	24.5215	1.46	Nedd1	NM_008682	Mus musculus neural precursor cell expressed, developmentally down-regulated gene 1 (Nedd1), mRNA.
scl016822.21_5-S	24.4608	1.32	Lcp2	NM_010696.2	Mus musculus lymphocyte cytosolic protein 2 (Lcp2), mRNA.
scl0104303.6_291-S	24.4573	1.62	Arl1	NM_025859.1	Mus musculus ADP-ribosylation factor-like 1 (Arl1), mRNA.
scl17984.2_572-S	24.4482	1.47	Sdpr	NM_138741.1	Mus musculus serum deprivation response (Sdpr), mRNA.
scl43007.18.1_29-S	24.4334	11.27	Rgs6	NM_015812.1	Mus musculus regulator of G-protein signaling 6 (Rgs6), mRNA.
scl29584.3_468-S	24.4323	1.5	Zfp637	NM_177684.2	
scl40726.5_15-S	24.4119	1.42	Kctd2	NM_183285.1	Mus musculus potassium channel tetramerisation domain containing 2 (Kctd2), mRNA.
scl40616.11_683-S	24.407	1.63	4430402O11Rik	NM_026272.2	Mus musculus RIKEN cDNA 4430402O11 gene (4430402O11Rik), mRNA.
scl30692.5.1_111-S	24.4029	1.85	Lat	NM_010689.2	Mus musculus linker for activation of T cells (Lat), mRNA.
scl0014560.2_59-S	24.3797	1.52	Gdf10	NM_145741.2	Mus musculus growth differentiation factor 10 (Gdf10), mRNA.
scl0231123.1_28-S	24.3669	1.52	BC023882	NM_146159.1	Mus musculus cDNA sequence BC023882 (BC023882), mRNA.

scl00233545.1_305-S	24.3647	1.57	2210018M11Rik		Mus musculus RIKEN cDNA 2210018M11 gene (2210018M11Rik), mRNA.
scl012740.2_183-S	24.3544	9.93	Cldn4	NM_009903.1	Mus musculus claudin 4 (Cldn4), mRNA.
scl39906.6_377-S	24.3529	1.68	AI851076	XM_110955.2	Mus musculus expressed sequence AI851076 (AI851076), mRNA.
scl37199.27.1_42-S	24.3501	1.63	E130103I17Rik	NM_181316.3	Mus musculus RIKEN cDNA E130103I17 gene (E130103I17Rik), mRNA.
scl0022123.1_274-S	24.3274	1.29	Psmd3	NM_009439.1	Mus musculus proteasome (prosome, macropain) 26S subunit, non-ATPase, 3 (Psmd3), mRNA.
scl18413.5.1_0-S	24.3062	1.86	9430008C03Rik	XM_149251.3	Mus musculus RIKEN cDNA 9430008C03 gene (9430008C03Rik), mRNA.
scl0001695.1_1621-S	24.2993	1.31	Ppm1b	NM_011151.1	Mus musculus protein phosphatase 1B, magnesium dependent, beta isoform (Ppm1b), mRNA.
scl34177.5_600-S	24.2826	1.44	2310022B05Rik	NM_175149.3	Mus musculus RIKEN cDNA 2310022B05 gene (2310022B05Rik), mRNA.
scl069035.1_26-S	24.2798	2.29	Zdhhc3	NM_026917.4	Mus musculus zinc finger, DHHC domain containing 3 (Zdhhc3), mRNA.
scl45540.5.1_3-S	24.2308	1.45	1500005A01Rik	NM_033146.1	Mus musculus RIKEN cDNA 1500005A01 gene (1500005A01Rik), mRNA.
scl00108907.2_230-S	24.2167	1.37	Nusap1	NM_133851.1	Mus musculus nucleolar and spindle associated protein 1 (Nusap1), mRNA.
IGKC_V00807_Ig_kappa_	24.1688	2.16	Igk-C	XM_132633.4	
scl15828.17_286-S	24.1531	1.34	Lbr	NM_133815.1	Mus musculus lamin B receptor (Lbr), mRNA.
scl00214572.1_279-S	24.1124	1.36	BC006705	NM_145404.1	Mus musculus cDNA sequence BC006705 (BC006705), mRNA.
scl067912.2_23-S	24.0935	1.53	1600012H06Rik	NM_026451.1	Mus musculus RIKEN cDNA 1600012H06 gene (1600012H06Rik), mRNA.
scl00227743.1_265-S	24.0531	1.43	Mapkap1	NM_177345.2	Mus musculus mitogen-activated protein kinase associated protein 1 (Mapkap1), mRNA.
scl026901.3_41-S	24.0283	1.61	Deb1	NM_026794.2	Mus musculus differentially expressed in B16F10 1 (Deb1), mRNA.
scl0056417.2_229-S	24.0186	1.56	Adar	NM_019655.2	Mus musculus adenosine deaminase, RNA-specific (Adar), mRNA.
scl0100986.1_13-S	23.9969	2.27	Akap9	NM_194462.1	Mus musculus A kinase (PRKA) anchor protein (yotiao) 9 (Akap9), mRNA.
scl0020481.2_24-S	23.9885	5.44	Ski	NM_011385.1	Mus musculus Sloan-Kettering viral oncogene homolog (Ski), mRNA.
scl41127.15.1_23-S	23.9577	1.52	Ddx52	NM_030096.1	Mus musculus DEAD (Asp-Glu-Ala-Asp) box polypeptide 52 (Ddx52), mRNA.
scl0012874.1_259-S	23.9469	2.16	Cpd	NM_007754.1	Mus musculus carboxypeptidase D (Cpd), mRNA.
scl0003218.1_27-S	23.9009	8.38	Ass1	NM_007494.2	Mus musculus argininosuccinate synthetase 1 (Ass1), mRNA.
scl33121.3_245-S	23.8771	2.08	Zfp524	NM_025324.1	
scl22876.3_595-S	23.87	1.37	Mcl1	NM_008562.2	Mus musculus myeloid cell leukemia sequence 1 (Mcl1), mRNA.
scl21684.2.5_0-S	23.8665	1.36	Rbm15	XM_131139.4	
scl0024061.1_292-S	23.8604	1.29	Smc1l1	NM_019710.1	Mus musculus SMC (structural maintenance of chromosomes 1)-like 1 (S. cerevisiae) (Smc1l1), mRNA.
scl0230796.1_23-S	23.8387	3.37	Wdtc1	NM_199306.1	
scl16594.5_443-S	23.8253	1.58	D1Bwg1363e	XM_129886.4	Mus musculus DNA segment, Chr 1, Brigham & Womens Genetics 1363 expressed (D1Bwg1363e), mRNA.
scl0012192.2_207-S	23.822	1.43	Zfp36l1	NM_007564.2	Mus musculus zinc finger protein 36, C3H type-like 1 (Zfp36l1), mRNA.
scl0227570.7_253-S	23.8052	1.54	2810455F06Rik	XM_130011.5	Mus musculus RIKEN cDNA 2810455F06 gene (2810455F06Rik), mRNA.
scl0022329.2_144-S	23.7655	1.56	Vcam1	NM_011693.2	Mus musculus vascular cell adhesion molecule 1 (Vcam1), mRNA.
scl50668.1.78_47-S	23.748	1.31	Tbcc	NM_178385.2	Mus musculus tubulin-specific chaperone c (Tbcc), mRNA.
scl23529.21_298-S	23.7365	2.17	Plod1	NM_011122.1	Mus musculus procollagen-lysine, 2-oxoglutarate 5-dioxygenase 1 (Plod1), mRNA.
scl0319180.1_4-S	23.7137	2.11	Hist1h2bf	NM_178195.1	Mus musculus histone 1, H2bf (Hist1h2bf), mRNA.
scl19617.10_166-S	23.7122	1.47	Pip5k2a	NM_008845.2	Mus musculus phosphatidylinositol-4-phosphate 5-kinase, type II, alpha (Pip5k2a), mRNA.
scl00319262.1_205-S	23.6903	2.5	Fchsd1	NM_175684.3	Mus musculus FCH and double SH3 domains 1 (Fchsd1), mRNA.
scl0016923.1_194-S	23.6743	1.65	Lnk	NM_008507.2	Mus musculus linker of T-cell receptor pathways (Lnk), mRNA.
scl016453.30_17-S	23.6642	1.55	Jak3	NM_010589	Mus musculus Janus kinase 3 (Jak3), mRNA.
scl026874.2_21-S	23.6277	7.29	Abcd2	NM_011994.1	Mus musculus ATP-binding cassette, sub-family D (ALD), member 2 (Abcd2), mRNA.
scl068052.3_30-S	23.627	1.38	Rps13	NM_026533	Mus musculus ribosomal protein S13 (Rps13), mRNA.
scl0001934.1_38-S	23.6135	1.3	Lmna	NM_019390.1	Mus musculus lamin A (Lmna), mRNA.
scl44991.2.1_21-S	23.6112	1.54	Hist1h2bc	NM_023422	Mus musculus histone 1, H2bc (Hist1h2bc), mRNA.
scl41638.9.1_29-S	23.6005	1.55	Timd4	NM_178759.3	
scl074270.26_30-S	23.5741	1.65	Usp20	NM_028846.1	Mus musculus ubiquitin specific protease 20 (Usp20), mRNA.
scl0002692.1_59-S	23.4832	1.59	Galt	NM_016658.1	Mus musculus galactose-1-phosphate uridyl transferase (Galt), mRNA.
scl0070990.1_251-S	23.4799	1.46	4931431L11Rik	XM_485553	
scl23684.11_537-S	23.4454	1.54	Sepn1	NM_029100.1	Mus musculus selenoprotein N, 1 (Sepn1), mRNA.
scl0003840.1_14-S	23.4279	10.18	Ppp1r14c	NM_133485.1	Mus musculus protein phosphatase 1, regulatory (inhibitor) subunit 14c (Ppp1r14c), mRNA.
scl45363.6_489-S	23.4279	1.47	6330407G04Rik	NM_134078.2	Mus musculus RIKEN cDNA 6330407G04 gene (6330407G04Rik), mRNA.
scl0016997.2_276-S	23.4221	1.95	Ltbp2	NM_013589.1	Mus musculus latent transforming growth factor beta binding protein 2 (Ltbp2), mRNA.
scl0192191.2_33-S	23.4046	1.95	BC019367	NM_138675.2	Mus musculus cDNA sequence BC019367 (BC019367), mRNA.
scl38150.2.1_277-S	23.3882	2.3	Slc35d3	XM_125515.3	
scl38416.13.1_1-S	23.3878	2.15	Ptprb	NM_029928.1	Mus musculus protein tyrosine phosphatase, receptor type, B (Ptprb), mRNA.
scl21104.8_445-S	23.3763	2.5	2900073H19Rik	NM_026615.2	Mus musculus RIKEN cDNA 2900073H19 gene (2900073H19Rik), mRNA.
scl000098.1_12-S	23.3632	1.45	0610007P22Rik	NM_026676.1	Mus musculus RIKEN cDNA 0610007P22 gene (0610007P22Rik), mRNA.

scl24913.11_38-S	23.3363	1.3	1110063F24Rik	NM_133889.2	Mus musculus RIKEN cDNA 1110063F24 gene (1110063F24Rik), mRNA.
scl28518.8.1_30-S	23.3302	1.98	Mbd4	NM_010774.1	Mus musculus methyl-CpG binding domain protein 4 (Mbd4), mRNA.
scl0319188.1_136-S	23.2931	1.7	Hist1h2bp	NM_178202	Mus musculus histone 1, H2bp (Hist1h2bp), mRNA.
scl30833.27_114-S	23.2871	1.3	St5	NM_029811.1	Mus musculus suppression of tumorigenicity 5 (St5), mRNA.
scl0109168.12_22-S	23.2767	2.66	5730596K20Rik	NM_146091.2	Mus musculus RIKEN cDNA 5730596K20 gene (5730596K20Rik), mRNA.
scl41425.8_289-S	23.241	2.97	Zfp535	NM_026107.2	Mus musculus zinc finger protein 535 (Zfp535), mRNA.
scl0014082.2_318-S	23.2325	1.78	Fadd	NM_010175.2	Mus musculus Fas (TNFRSF6)-associated via death domain (Fadd), mRNA.
scl0069386.2_29-S	23.2182	2.66	Hist1h4h	NM_153173.1	Mus musculus histone 1, H4h (Hist1h4h), mRNA.
scl077897.11_26-S	23.1873	5.54	Cep1	NM_030000.1	Mus musculus centrosomal protein 1 (Cep1), mRNA.
scl0003280.1_90-S	23.1842	7.07	C330016H24Rik	NM_030155.1	Mus musculus RIKEN cDNA C330016H24 gene (C330016H24Rik), mRNA.
scl34329.24.1_1-S	23.1684	2.64	Fuk	NM_172283.2	Mus musculus fucokinase (Fuk), transcript variant 1, mRNA.
scl31502.9_323-S	23.1672	1.48	Fxyd5	NM_008761.2	Mus musculus FXYD domain-containing ion transport regulator 5 (Fxyd5), mRNA.
scl0011569.2_215-S	23.1229	6.94	Aebp2	NM_009637.1	Mus musculus AE binding protein 2 (Aebp2), mRNA.
scl0066365.1_89-S	23.1132	1.43	2310015N07Rik	NM_025515.1	Mus musculus RIKEN cDNA 2310015N07 gene (2310015N07Rik), mRNA.
scl0109032.3_2-S	23.1056	2.17	5830484A20Rik	XM_284155.2	Mus musculus RIKEN cDNA 5830484A20 gene (5830484A20Rik), mRNA.
scl066292.2_43-S	23.1038	1.35	Mrps21	NM_078479.2	Mus musculus mitochondrial ribosomal protein S21 (Mrps21), mRNA.
scl41017.13_82-S	23.0943	1.35	Ppp1r9b	NM_172261.1	Mus musculus protein phosphatase 1, regulatory subunit 9B (Ppp1r9b), mRNA.
scl0003447.1_0-S	23.0918	2.8	Rbm5	NM_148930.2	Mus musculus RNA binding motif protein 5 (Rbm5), mRNA.
scl059050.2_22-S	23.0338	1.31	5730427N09Rik	NM_021552.1	Mus musculus RIKEN cDNA 5730427N09 gene (5730427N09Rik), mRNA.
scl0023808.2_287-S	23.0305	1.38	Ash2l	NM_011791.1	Mus musculus ash2 (absent, small, or homeotic)-like (Drosophila) (Ash2l), mRNA.
scl45283.8.141_93-S	22.9831	1.62	9030625A04Rik	NM_172488.1	Mus musculus RIKEN cDNA 9030625A04 gene (9030625A04Rik), mRNA.
scl35713.2.1_23-S	22.9441	1.62	2300009A05Rik	XM_204313.2	Mus musculus RIKEN cDNA 2300009A05 gene (2300009A05Rik), mRNA.
scl19453.6.2634_3-S	22.9148	1.51	A130092J06Rik	NM_175511.2	Mus musculus RIKEN cDNA A130092J06 gene (A130092J06Rik), mRNA.
scl23928.4.1_17-S	22.8889	1.58	Dph2l2	NM_026344.2	Mus musculus diptheria toxin resistance protein required for diphthamide biosynthesis (Saccharomyces)-like 2 (Dph2l2), mRNA.
scl19566.6.1_18-S	22.8797	1.83	Dpp7	NM_031843.2	Mus musculus dipeptidylpeptidase 7 (Dpp7), mRNA.
scl18197.5_22-S	22.8676	1.32	Rgs19	NM_026446.2	Mus musculus regulator of G-protein signaling 19 (Rgs19), mRNA.
scl36336.8_440-S	22.8663	1.73	Rpl14	NM_025974.1	Mus musculus ribosomal protein L14 (Rpl14), mRNA.
scl44126.11.1_57-S	22.801	1.97	Gmds	NM_146041.1	Mus musculus GDP-mannose 4, 6-dehydratase (Gmds), mRNA.
scl022666.6_141-S	22.7584	1.37	Zfp161	NM_009547.2	Mus musculus zinc finger protein 161 (Zfp161), mRNA.
scl51993.3_190-S	22.7384	1.39	Eif1a	NM_010120.3	Mus musculus eukaryotic translation initiation factor 1A (Eif1a), mRNA.
scl17152.12_234-S	22.723	1.76	9630058J23Rik	NM_146105.3	Mus musculus RIKEN cDNA 9630058J23 gene (9630058J23Rik), mRNA.
scl30187.2.1_182-S	22.702	1.34	1110001J03Rik	NM_025363.2	Mus musculus RIKEN cDNA 1110001J03 gene (1110001J03Rik), mRNA.
scl023994.4_204-S	22.6769	1.25	Dazap2	NM_011873.2	Mus musculus DAZ associated protein 2 (Dazap2), mRNA.
scl43551.17_116-S	22.648	1.82	Nln	NM_029447.1	Mus musculus neurolysin (metallopeptidase M3 family) (Nln), mRNA.
scl47441.30_193-S	22.6255	1.35	Hemp1	NM_153505.2	
scl069146.11_118-S	22.5675	1.72	Gsdmdc1	NM_026960.1	
scl29977.11.1_22-S	22.5585	2.69	Mmrn1	XM_284198.2	
scl0066432.1_29-S	22.5576	1.63	2010007L18Rik	XM_134478.2	Mus musculus RIKEN cDNA 2010007L18 gene (2010007L18Rik), mRNA.
scl012765.6_0-S	22.5509	1.74	Il8rb	NM_009909.2	Mus musculus interleukin 8 receptor, beta (Il8rb), mRNA.
scl020848.1_5-S	22.5324	1.59	Stat3	NM_011486.2	Mus musculus signal transducer and activator of transcription 3 (Stat3), mRNA.
scl070221.8_1-S	22.5036	3.2	2600011C06Rik	NM_027349.1	Mus musculus RIKEN cDNA 2600011C06 gene (2600011C06Rik), mRNA.
scl066158.1_320-S	22.4925	1.44	1110012O05Rik	NM_024170	Mus musculus RIKEN cDNA 1110012O05 gene (1110012O05Rik), mRNA.
scl00269682.2_253-S	22.4897	1.65	Golga3	NM_008146.3	Mus musculus golgi autoantigen, golgin subfamily a, 3 (Golga3), mRNA.
scl0192976.1_323-S	22.4811	1.74	BC046404	NM_198861.1	Mus musculus cDNA sequence BC046404 (BC046404), mRNA.
scl00209630.2_217-S	22.4601	1.35	Frmd4a	NM_172475.2	
scl018817.10_297-S	22.4346	1.54	Plk1	NM_011121.2	Mus musculus polo-like kinase 1 (Drosophila) (Plk1), mRNA.
scl39338.3_4-S	22.4086	1.28	Nt5c	NM_015807.1	Mus musculus 5,3-nucleotidase, cytosolic (Nt5c), mRNA.
scl0052040.1_253-S	22.385	1.29	Ppp1r10	NM_175934.2	Mus musculus protein phosphatase 1, regulatory subunit 10 (Ppp1r10), mRNA.
scl43508.21.1_5-S	22.3835	1.68	Map3k1	NM_011945.1	Mus musculus mitogen activated protein kinase kinase kinase 1 (Map3k1), mRNA.
scl52415.12_0-S	22.3574	1.29	Ldb1	NM_010697.1	Mus musculus LIM domain binding 1 (Ldb1), mRNA.
scl24033.7.1_11-S	22.3373	1.29	Mrpl37	NM_025500.1	Mus musculus mitochondrial ribosomal protein L37 (Mrpl37), mRNA.
scl0076803.1_194-S	22.3312	11.73	2410141K09Rik	NM_183119	Mus musculus RIKEN cDNA 2410141K09 gene (2410141K09Rik), mRNA.
scl31972.8_47-S	22.3012	1.83	Bub3	NM_009774.2	Mus musculus budding uninhibited by benzimidazoles 3 homolog (S. cerevisiae) (Bub3), mRNA.
scl074132.1_5-S	22.2993	1.41	Rnf6	NM_028774.1	
scl0258666.1_1-S	22.2971	12.06	Olfr822	NM_146671.1	Mus musculus olfactory receptor 822 (Olfr822), mRNA.
scl20781.3.23_109-S	22.297	2.04	1700011J10Rik	NM_183265	Mus musculus RIKEN cDNA 1700011J10 gene (1700011J10Rik), mRNA.

scl0076281.1_330-S	22.2937	1.32	Tax1bp3	NM_029564	
scl30452.18_578-S	22.2919	1.26	Nap1l4	NM_008672	Mus musculus nucleosome assembly protein 1-like 4 (Nap1l4), mRNA.
scl16258.13.1_64-S	22.2842	1.35	Rnpep	NM_145417	Mus musculus arginyl aminopeptidase (aminopeptidase B) (Rnpep), mRNA.
scl24658.9.1_90-S	22.2751	2.63	Tnfrsf25	NM_033042	Mus musculus tumor necrosis factor receptor superfamily, member 25 (Tnfrsf25), mRNA.
scl35535.28_151-S	22.2616	1.64	Ibtk	XM_135030.4	
scl30402.11.1_13-S	22.2279	1.52	BC020002	NM_145374.1	Mus musculus cDNA sequence BC020002 (BC020002), mRNA.
scl50802.2_363-S	22.209	1.92	Bat9	NM_198886.2	Mus musculus HLA-B-associated transcript 9 (Bat9), mRNA.
scl024135.1_208-S	22.2023	1.55	Zfp68	NM_013844.1	Mus musculus zinc finger protein 68 (Zfp68), mRNA.
scl000724.1_245-S	22.2009	4.43	Calr3	NM_029782.2	Mus musculus calreticulin 3 (Calr3), mRNA.
scl32679.14.1_2-S	22.1898	7.39	Tulp2	NM_008807.1	Mus musculus tubby-like protein 2 (Tulp2), mRNA.
scl38985.8_420-S	22.1839	1.32	Cd164	NM_016898.1	Mus musculus CD164 antigen (Cd164), mRNA.
scl078751.8_330-S	22.1523	2.86	Zc3hdc6	NM_178404.2	
scl0225912.8_241-S	22.1421	1.49	BC065078	NM_201351.1	Mus musculus cDNA sequence BC065078 (BC065078), mRNA.
scl0016159.2_68-S	22.1348	2.96	Il12a	NM_008351.1	Mus musculus interleukin 12a (Il12a), mRNA.
scl34464.12_3-S	22.1316	1.69	Dok4	NM_053246.1	Mus musculus downstream of tyrosine kinase 4 (Dok4), mRNA.
scl00103284.2_177-S	22.1314	1.79	AI790326	NM_134003.1	Mus musculus expressed sequence AI790326 (AI790326), mRNA.
scl17783.10_25-S	22.1308	1.31	BC038286	NM_170755.1	Mus musculus cDNA sequence BC038286 (BC038286), mRNA.
scl00320011.1_153-S	22.1203	1.37	Ugcgl1	NM_198899.1	
scl0003659.1_24-S	22.1165	4.47	Mef2c	NM_025282.1	Mus musculus myocyte enhancer factor 2C (Mef2c), mRNA.
scl00116731.1_145-S	22.1111	4.4	Pcdha1	NM_054072	Mus musculus protocadherin alpha 1 (Pcdha1), mRNA.
scl52839.2_29-S	22.1065	1.47	AI837181	NM_134149.1	Mus musculus expressed sequence AI837181 (AI837181), mRNA.
scl0278279.1_174-S	22.0455	11.27	8430438D04Rik	NM_177368.2	Mus musculus RIKEN cDNA 8430438D04 gene (8430438D04Rik), mRNA.
scl0071702.2_200-S	22.0443	1.27	Cdc5l	NM_152810.1	Mus musculus cell division cycle 5-like (S. pombe) (Cdc5l), mRNA.
scl0020848.1_188-S	22.0326	1.34	Stat3	NM_011486.2	Mus musculus signal transducer and activator of transcription 3 (Stat3), mRNA.
scl53499.1.4_164-S	22.0276	4.68	1700020D05Rik	NM_023781.3	Mus musculus RIKEN cDNA 1700020D05 gene (1700020D05Rik), mRNA.
scl0067382.2_271-S	22.0155	1.33	Brd3	NM_023336.2	Mus musculus bromodomain containing 3 (Brd3), mRNA.
scl0011737.2_249-S	22.0033	1.31	Anp32a	NM_009672.2	Mus musculus acidic (leucine-rich) nuclear phosphoprotein 32 family, member A (Anp32a), mRNA.
scl0076367.2_3-S	22.002	7.96	Trp53rk	NM_023815.2	
scl23193.10.1_8-S	21.988	1.76	Alg5	NM_025442.1	Mus musculus asparagine-linked glycosylation 5 homolog (yeast, dolichyl-phosphate beta-glucosyltransferase) (Alg5), mRNA.
scl056398.7_324-S	21.9785	1.27	1500003O03Rik	NM_019769.2	Mus musculus RIKEN cDNA 1500003O03 gene (1500003O03Rik), mRNA.
scl32648.17_169-S	21.9339	1.28	Gtf2h1	NM_008186.2	Mus musculus general transcription factor II H, polypeptide 1 (Gtf2h1), mRNA.
scl0231225.1_255-S	21.9335	1.28	4932414K18Rik	NM_173764	Mus musculus RIKEN cDNA 4932414K18 gene (4932414K18Rik), mRNA.
scl067440.9_18-S	21.926	2.09	Papd1	NM_026157.1	Mus musculus PAP associated domain containing 1 (Papd1), mRNA.
scl54662.1.1_35-S	21.9071	11.25	9330152L17	NM_177917.2	Mus musculus hypothetical protein 9330152L17 (9330152L17), mRNA.
scl54620.5_512-S	21.9059	1.65	Bhlhb9	NM_198161.1	
scl24988.17.1_42-S	21.9004	1.37	Sf3a3	NM_029157.2	Mus musculus splicing factor 3a, subunit 3 (Sf3a3), mRNA.
scl0214505.1_41-S	21.8958	1.67	AU067744	NM_172529.1	Mus musculus expressed sequence AU067744 (AU067744), mRNA.
scl32299.7.1_25-S	21.8949	1.43	Stard10	NM_019990.1	Mus musculus START domain containing 10 (Stard10), mRNA.
scl33668.2_157-S	21.8853	1.86	F2rl3	NM_007975.2	Mus musculus coagulation factor II (thrombin) receptor-like 3 (F2rl3), mRNA.
scl071562.11_5-S	21.8739	1.65	Afmid	NM_027827.2	Mus musculus arylformamidase (Afmid), mRNA.
scl37927.8.1_330-S	21.85	1.68	Slc29a3	NM_023596.2	Mus musculus solute carrier family 29 (nucleoside transporters), member 3 (Slc29a3), mRNA.
scl53116.10_62-S	21.831	1.45	Pi4k2a	NM_145501.1	Mus musculus phosphatidylinositol 4-kinase type 2 alpha (Pi4k2a), mRNA.
scl0107734.5_7-S	21.8057	1.34	Mrpl30	NM_027098.1	Mus musculus mitochondrial ribosomal protein L30 (Mrpl30), mRNA.
scl0067665.1_200-S	21.7918	1.33	Dctn4	NM_026302.2	Mus musculus dynactin 4 (Dctn4), mRNA.
scl0066610.1_317-S	21.7889	1.69	Abi3	NM_025659.1	Mus musculus ABI gene family, member 3 (Abi3), mRNA.
scl0030955.2_290-S	21.7888	1.52	Pik3cg	NM_020272.1	Mus musculus phosphoinositide-3-kinase, catalytic, gamma polypeptide (Pik3cg), mRNA.
scl38655.14_451-S	21.7879	1.56	Pip5k1c	XM_150337.1	Mus musculus phosphatidylinositol-4-phosphate 5-kinase, type 1 gamma (Pip5k1c), mRNA.
scl29212.6_52-S	21.7767	6.49	Opn1sw	NM_007538.2	Mus musculus opsin 1 (cone pigments), short-wave-sensitive (color blindness, tritan) (Opn1sw), mRNA.
scl0026908.1_233-S	21.7635	1.53	Eif2s3y	NM_012011.1	Mus musculus eukaryotic translation initiation factor 2, subunit 3, structural gene Y-linked (Eif2s3y), mRNA.
scl37399.12_263-S	21.7501	1.89	4632413K17Rik	NM_177614.2	Mus musculus RIKEN cDNA 4632413K17 gene (4632413K17Rik), mRNA.
scl21992.22_340-S	21.7265	4.29	3110045G13Rik	NM_028460.1	Mus musculus RIKEN cDNA 3110045G13 gene (3110045G13Rik), mRNA.
scl20314.23.1_217-S	21.7159	6.86	Acoxl	NM_028765.1	
scl0003632.1_1-S	21.7135	10.45	Cast	NM_009817.1	Mus musculus calpastatin (Cast), mRNA.
scl18854.8.1_1-S	21.7018	3.86	Actc1	NM_009608.1	Mus musculus actin, alpha, cardiac (Actc1), mRNA.
scl018521.14_30-S	21.685	1.27	Pcbp2	NM_011042.1	Mus musculus poly(rC) binding protein 2 (Pcbp2), mRNA.
scl25461.9_196-S	21.6839	1.4	Nans	NM_053179.2	Mus musculus N-acetylneuraminic acid synthase (sialic acid synthase) (Nans), mRNA.

scl00319192.1_1-S	21.6771	2.15	Hist2h2aa2	NM_178212	Mus musculus histone 2, H2aa2 (Hist2h2aa2), mRNA.
scl31513.2.1_158-S	21.626	6.96	4930479M11Rik	NM_183108.1	Mus musculus RIKEN cDNA 4930479M11 gene (4930479M11Rik), mRNA.
scl16472.29.1_1-S	21.6228	3.1	4932408F19Rik	NM_207225.1	Mus musculus RIKEN cDNA 4932408F19 gene (4932408F19Rik), mRNA.
scl0002403.1_56-S	21.6221	2.56	Sdccag1	NM_181665.1	Mus musculus serologically defined colon cancer antigen 1 (Sdccag1), transcript variant 1, mRNA.
scl35797.7.1_123-S	21.5911	1.3	Commd4	NM_025417.1	Mus musculus COMM domain containing 4 (Commd4), mRNA.
scl29407.39.1_52-S	21.5596	8.4	Pik3c2g	NM_011084	Mus musculus phosphatidylinositol 3-kinase, C2 domain containing, gamma polypeptide (Pik3c2g), transcript variant 2, mRNA.
scl36433.10_466-S	21.5579	3.16	2610002I17Rik	XM_135177.4	Mus musculus RIKEN cDNA 2610002I17 gene (2610002I17Rik), mRNA.
scl52628.17.1_63-S	21.544	1.7	BC018472	NM_146097.1	Mus musculus cDNA sequence BC018472 (BC018472), mRNA.
scl0210148.13_0-S	21.5427	1.61	Slc30a6	NM_144798.2	Mus musculus solute carrier family 30 (zinc transporter), member 6 (Slc30a6), mRNA.
scl33707.42_67-S	21.5313	1.7	Myo9b	NM_015742.1	Mus musculus myosin IXb (Myo9b), mRNA.
scl0235493.12_117-S	21.5302	1.55	BC031353	NM_153584.1	Mus musculus cDNA sequence BC031353 (BC031353), mRNA.
scl26719.6_100-S	21.5144	1.68	Spon2	NM_133903.2	Mus musculus spondin 2, extracellular matrix protein (Spon2), mRNA.
scl0223672.1_326-S	21.4935	11.1	LOC223672	XM_128064.4	Mus musculus hypothetical protein LOC223672 (LOC223672), mRNA.
scl45393.10_71-S	21.4881	1.24	Bnip3l	NM_009761.2	Mus musculus BCL2/adenovirus E1B 19kDa-interacting protein 3-like (Bnip3l), mRNA.
scl0022379.1_143-S	21.4148	1.58	Fmnl3	NM_011711.1	Mus musculus formin-like 3 (Fmnl3), mRNA.
scl23602.11_383-S	21.4007	1.32	1110005F07Rik	NM_025383	Mus musculus RIKEN cDNA 1110005F07 gene (1110005F07Rik), mRNA.
scl026446.4_108-S	21.3916	1.27	Psmb3	NM_011971.2	Mus musculus proteasome (prosome, macropain) subunit, beta type 3 (Psmb3), mRNA.
scl55015.6.1_13-S	21.3849	2.28	Timp1	NM_011593	Mus musculus tissue inhibitor of metalloproteinase 1 (Timp1), mRNA.
scl0018130.2_77-S	21.3789	1.34	Ddx26	NM_008715.1	Mus musculus DEAD/H (Asp-Glu-Ala-Asp/His) box polypeptide 26 (Ddx26), mRNA.
scl27382.16_83-S	21.3765	1.28	1100001D10Rik	XM_358359.1	Mus musculus RIKEN cDNA 1100001D10 gene (1100001D10Rik), mRNA.
scl39788.24_563-S	21.3231	2.02	Brip1	NM_178309.1	Mus musculus BRCA1 interacting protein C-terminal helicase 1 (Brip1), mRNA.
scl030046.1_110-S	21.3004	1.77	Zfp292	NM_013889.1	Mus musculus zinc finger protein 292 (Zfp292), mRNA.
scl0272322.13_8-S	21.2887	8.26	Arntl2	NM_172309.1	Mus musculus aryl hydrocarbon receptor nuclear translocator-like 2 (Arntl2), mRNA.
scl52693.20_358-S	21.2811	1.58	Tle4	NM_011600.2	Mus musculus transducin-like enhancer of split 4, homolog of Drosophila E(spl) (Tle4), mRNA.
scl0015587.1_282-S	21.2775	1.35	Hyal2	NM_010489.2	Mus musculus hyaluronidase 2 (Hyal2), mRNA.
scl000390.1_159-S	21.2669	6.77	Mtrf1	NM_145960	Mus musculus mitochondrial translational release factor 1 (Mtrf1), mRNA.
scl27224.3.7_29-S	21.2583	1.55	Arl6ip4	NM_144509.1	Mus musculus ADP-ribosylation factor-like 6 interacting protein 4 (Arl6ip4), mRNA.
scl25570.5.1_79-S	21.2464	10.63	Srrp	NM_177774.2	Mus musculus serine-arginine repressor protein (Srrp), mRNA.
scl076281.4_19-S	21.2223	1.32	Tax1bp3	NM_029564	
scl19511.8_175-S	21.2186	1.68	Slc2a6	NM_172659.1	Mus musculus solute carrier family 2 (facilitated glucose transporter), member 6 (Slc2a6), mRNA.
scl0014367.2_144-S	21.2143	6.53	Fzd5	NM_022721.1	Mus musculus frizzled homolog 5 (Drosophila) (Fzd5), mRNA.
scl33219.11.1_14-S	21.2085	3.42	BC021611	NM_144932.2	Mus musculus cDNA sequence BC021611 (BC021611), mRNA.
scl33241.10_186-S	21.1727	1.33	Icsbp1	NM_008320.2	Mus musculus interferon consensus sequence binding protein 1 (Icsbp1), mRNA.
scl30691.10.4_70-S	21.1589	1.61	2210013K02Rik	NM_023712	Mus musculus RIKEN cDNA 2210013K02 gene (2210013K02Rik), mRNA.
scl056444.13_2-S	21.1311	1.29	Actr10	NM_019785.1	Mus musculus ARP10 actin-related protein 10 homolog (S. cerevisiae) (Actr10), mRNA.
scl0020947.1_79-S	21.1267	1.45	Swap70	NM_009302.2	Mus musculus SWAP complex protein (Swap70), mRNA.
scl0239743.2_104-S	21.1114	4.65	Klhl6	NM_183390.1	Mus musculus kelch-like 6 (Drosophila) (Klhl6), mRNA.
scl31127.23.1_1-S	21.0938	1.57	AW538196	NM_133952.1	Mus musculus expressed sequence AW538196 (AW538196), mRNA.
scl0003687.1_76-S	21.0638	3.8	Trim23	NM_030731.1	Mus musculus tripartite motif protein 23 (Trim23), mRNA.
scl44948.10_526-S	21.0614	1.46	Irf4	NM_013674.1	Mus musculus interferon regulatory factor 4 (Irf4), mRNA.
scl070361.1_127-S	21.0465	1.42	Lman1	NM_027400.2	Mus musculus lectin, mannose-binding, 1 (Lman1), mRNA.
scl39143.6.1_8-S	21.0374	2.04	Deadc1	NM_025748.2	Mus musculus deaminase domain containing 1 (Deadc1), mRNA.
scl32924.1_1-S	20.9847	1.88	BC025206	NM_146184.2	
scl020044.5_19-S	20.9749	1.68	Rps14	NM_020600.2	Mus musculus ribosomal protein S14 (Rps14), mRNA.
scl50581.4.1_28-S	20.9584	1.52	2310045B01Rik	NM_025538.1	Mus musculus RIKEN cDNA 2310045B01 gene (2310045B01Rik), mRNA.
scl38024.23.1_253-S	20.9432	1.49	A530089I17Rik	NM_133999.1	Mus musculus RIKEN cDNA A530089I17 gene (A530089I17Rik), mRNA.
scl51843.7.3_62-S	20.9257	1.55	1810029G24Rik	NM_025468.1	Mus musculus RIKEN cDNA 1810029G24 gene (1810029G24Rik), mRNA.
scl48393.15_330-S	20.8912	2.04	Nfkbiz	NM_030612	
scl018120.4_62-S	20.8878	1.35	Mrpl49	NM_026246.1	Mus musculus mitochondrial ribosomal protein L49 (Mrpl49), mRNA.
scl36920.15.1_107-S	20.8872	1.46	Pstpip1	NM_011193.1	Mus musculus proline-serine-threonine phosphatase-interacting protein 1 (Pstpip1), mRNA.
scl074522.7_319-S	20.8859	1.45	Zcwcc1	NM_198162.1	Mus musculus zinc finger, CW-type with coiled-coil domain 1 (Zcwcc1), mRNA.
scl49960.7_412-S	20.8661	1.72	Trim39	NM_024468.1	Mus musculus tripartite motif protein 39 (Trim39), mRNA.
scl38270.7_86-S	20.8468	3.04	Dnajc14	NM_028873.2	
scl0022661.2_277-S	20.8319	1.95	Zfp148	NM_011749.3	Mus musculus zinc finger protein 148 (Zfp148), mRNA.
scl0072333.1_312-S	20.8223	2.14	2410003B16Rik	XM_204283.3	Mus musculus RIKEN cDNA 2410003B16 gene (2410003B16Rik), mRNA.
scl0014682.1_7-S	20.8223	1.41	Gnaq	NM_008139.2	Mus musculus guanine nucleotide binding protein, alpha q polypeptide (Gnaq), mRNA.

scl067604.1_6-S	20.8093	1.3	1110007L15Rik	NM_026269.1	Mus musculus RIKEN cDNA 1110007L15 gene (1110007L15Rik), mRNA.
scl38195.4_424-S	20.8051	1.56	Stx11	XM_203312.2	Mus musculus syntaxin 11 (Stx11), mRNA.
scl0068046.2_0-S	20.7737	1.78	2700062C07Rik	NM_026529.2	Mus musculus RIKEN cDNA 2700062C07 gene (2700062C07Rik), mRNA.
scl0022032.1_225-S	20.7565	3.16	Traf4	NM_009423.2	Mus musculus Tnf receptor associated factor 4 (Traf4), mRNA.
scl0018810.1_283-S	20.7426	1.49	Plec1	NM_011117	Mus musculus plectin 1 (Plec1), transcript variant 1, mRNA.
scl066637.1_226-S	20.7405	1.37	5730449L18Rik	NM_025677.1	Mus musculus RIKEN cDNA 5730449L18 gene (5730449L18Rik), mRNA.
scl00332397.1_279-S	20.7303	3.18	Nanos1	NM_178421.2	Mus musculus nanos homolog 1 (Drosophila) (Nanos1), mRNA.
scl0075731.2_157-S	20.6459	1.45	5133401N09Rik	NM_198004.1	Mus musculus RIKEN cDNA 5133401N09 gene (5133401N09Rik), mRNA.
scl48691.8.1_1-S	20.6457	2.01	Slc25a1	NM_153150.1	Mus musculus solute carrier family 25 (mitochondrial carrier; citrate transporter), member 1 (Slc25a1), mRNA.
scl0011975.2_216-S	20.6099	1.36	Atp6v0a1	NM_016920.1	Mus musculus ATPase, H+ transporting, lysosomal V0 subunit a isoform 1 (Atp6v0a1), mRNA.
scl0054004.2_173-S	20.5642	7.19	Diap2	NM_017398.1	Mus musculus diaphanous homolog 2 (Drosophila) (Diap2), mRNA.
scl0001062.1_67-S	20.5607	1.84	Cnot4	NM_016877.2	Mus musculus CCR4-NOT transcription complex, subunit 4 (Cnot4), mRNA.
scl00224727.2_315-S	20.5583	1.49	Bat3	NM_057171.1	Mus musculus HLA-B-associated transcript 3 (Bat3), mRNA.
scl0067857.2_151-S	20.5425	1.27	Ppp6c	NM_024209.1	Mus musculus protein phosphatase 6, catalytic subunit (Ppp6c), mRNA.
scl48561.1.13_8-S	20.5309	1.5	0610012G03Rik	NM_025320	Mus musculus RIKEN cDNA 0610012G03 gene (0610012G03Rik), mRNA.
scl38757.5.1_81-S	20.5131	1.64	Ndg2	NM_175329.3	
scl012520.7_274-S	20.5126	1.36	Cd81	NM_133655.1	Mus musculus CD 81 antigen (Cd81), mRNA.
scl027397.1_17-S	20.5056	1.37	Mrpl17	NM_025301.1	Mus musculus mitochondrial ribosomal protein L17 (Mrpl17), mRNA.
scl43154.2_481-S	20.4891	1.27	Arf6	NM_007481.1	Mus musculus ADP-ribosylation factor 6 (Arf6), mRNA.
scl19764.11_583-S	20.4832	1.38	Tpd52l2	NM_025482.2	Mus musculus tumor protein D52-like 2 (Tpd52l2), mRNA.
scl47919.3_631-S	20.4787	2.07	D530033C11Rik	NM_030132.2	Mus musculus RIKEN cDNA D530033C11 gene (D530033C11Rik), mRNA.
scl20534.5_360-S	20.4767	1.41	Cd59a	NM_007652.2	Mus musculus CD59a antigen (Cd59a), mRNA.
scl066943.7_225-S	20.3979	1.24	2310009N05Rik	NM_025861.1	Mus musculus RIKEN cDNA 2310009N05 gene (2310009N05Rik), mRNA.
scl40580.5.1_111-S	20.3645	1.23	1110020P15Rik	NM_197979.1	Mus musculus RIKEN cDNA 1110020P15 gene (1110020P15Rik), mRNA.
scl0404314.1_3-S	20.3558	9.49	Olfr257	NM_207554	Mus musculus olfactory receptor 257 (Olfr257), mRNA.
scl142.1.1_252-S	20.3384	10.68	Olfr370	NM_146270.1	Mus musculus olfactory receptor 370 (Olfr370), mRNA.
scl42976.13.1_6-S	20.3104	1.48	Coq6	NM_172582.1	Mus musculus coenzyme Q6 homolog (yeast) (Coq6), mRNA.
scl068911.1_313-S	20.2722	1.42	Pygo2	XM_130932.5	
scl31022.12_350-S	20.2112	1.67	Capn5	NM_007602.2	Mus musculus calpain 5 (Capn5), mRNA.
scl068642.1_282-S	20.2028	2.18	2810441K11Rik	NM_026798.1	Mus musculus RIKEN cDNA 2810441K11 gene (2810441K11Rik), mRNA.
scl015207.2_11-S	20.1973	7.12	Hes3	NM_008237.1	Mus musculus hairy and enhancer of split 3 (Drosophila) (Hes3), mRNA.
scl53405.2_540-S	20.1435	3.55	Zbtb3	NM_133759.1	Mus musculus zinc finger and BTB domain containing 3 (Zbtb3), mRNA.
scl0020271.2_300-S	20.1286	10.98	Scn5a	NM_021544.1	Mus musculus sodium channel, voltage-gated, type V, alpha polypeptide (Scn5a), mRNA.
scl34704.4_282-S	20.1264	1.43	C630013N10Rik	NM_172052.1	Mus musculus RIKEN cDNA C630013N10 gene (C630013N10Rik), mRNA.
scl53371.28_117-S	20.1195	1.27	Ddb1	NM_015735.1	Mus musculus damage specific DNA binding protein 1 (Ddb1), mRNA.
scl45684.12_151-S	20.1024	1.29	D14Ertd226e	NM_145928	Mus musculus DNA segment, Chr 14, ERATO Doi 226, expressed (D14Ertd226e), mRNA.
scl0012334.2_163-S	20.0907	1.39	Capn2	NM_009794.1	Mus musculus calpain 2 (Capn2), mRNA.
scl35324.20.1_127-S	20.0832	1.35	Dhx30	NM_133347.1	Mus musculus DEAH (Asp-Glu-Ala-His) box polypeptide 30 (Dhx30), mRNA.
scl056812.9_277-S	20.0632	1.29	Dnajb10	NM_178055.2	Mus musculus DnaJ (Hsp40) homolog, subfamily B, member 10 (Dnajb10), mRNA.
scl021357.9_262-S	20.0571	1.32	Tarbp2	NM_009319	Mus musculus TAR (HIV) RNA binding protein 2 (Tarbp2), mRNA.
scl00242687.2_177-S	20.0183	1.89	Wasf2	NM_153423.3	Mus musculus WAS protein family, member 2 (Wasf2), mRNA.
**GENES DOWN-REGULATED BY THE ATHEROGENIC DIET IN SPLEENS**					
**TargetID**	**Diff_Score**	**Fold_Change**	**Symbol**	**Accession**	**Definition**
scl24492.20.1_105-S	-371.3336	-67.09	1810074P20Rik	NM_026194.2	Mus musculus RIKEN cDNA 1810074P20 gene (1810074P20Rik), mRNA.
scl32408.23_56-S	-218.2759	-2.96	Picalm	NM_146194	Mus musculus phosphatidylinositol binding clathrin assembly protein (Picalm), mRNA.
scl0015519.1_14-S	-202.1406	-4.45	Hspca	NM_010480	Mus musculus heat shock protein 1, alpha (Hspca), mRNA.
scl0016796.1_242-S	-137.0215	-2.29	Lasp1	NM_010688.2	Mus musculus LIM and SH3 protein 1 (Lasp1), mRNA.
scl29519.10.9_31-S	-135.1647	-3.93	Bcap37	NM_007531.1	Mus musculus B-cell receptor-associated protein 37 (Bcap37), mRNA.
scl011951.2_99-S	-118.3322	-2.05	Atp5g1	NM_007506.2	Mus musculus ATP synthase, H+ transporting, mitochondrial F0 complex, subunit c (subunit 9), isoform 1 (Atp5g1), mRNA.
scl0018813.1_104-S	-116.5303	-2.15	Pa2g4	NM_011119.1	Mus musculus proliferation-associated 2G4 (Pa2g4), mRNA.
scl018643.4_21-S	-109.3146	-3.01	Pfn1	NM_011072.2	Mus musculus profilin 1 (Pfn1), mRNA.
scl26467.3.1_2-S	-102.7471	-2.1	Chic2	NM_028850.1	Mus musculus cysteine-rich hydrophobic domain 2 (Chic2), mRNA.
scl0022144.2_2-S	-101.6286	-3.7	Tuba3	NM_009446.1	Mus musculus tubulin, alpha 3 (Tuba3), mRNA.
scl013681.1_1-S	-100.9534	-1.96	Eif4a1	NM_144958.2	Mus musculus eukaryotic translation initiation factor 4A1 (Eif4a1), mRNA.
scl49148.7.844_25-S	-98.0095	-3.76	Cd80	NM_009855	Mus musculus CD80 antigen (Cd80), mRNA.
scl066475.2_30-S	-97.5206	-2.3	Rps23	NM_024175	Mus musculus ribosomal protein S23 (Rps23), mRNA.

scl0001498.1_3411-S	-95.1501	-2.44	Ankfy1	NM_009671	Mus musculus ankyrin repeat and FYVE domain containing 1 (Ankfy1), mRNA.
scl0003742.1_4-S	-94.5327	-1.92	Tmem14c	NM_025387.1	
scl011867.9_22-S	-93.6991	-1.83	Arpc1b	NM_023142.1	Mus musculus actin related protein 2/3 complex, subunit 1B (Arpc1b), mRNA.
scl0234852.1_50-S	-90.432	-1.89	Pcoln3	NM_145606.1	Mus musculus procollagen (type III) N-endopeptidase (Pcoln3), mRNA.
scl0002580.1_4-S	-89.9325	-1.8	Ptp4a3	NM_008975.2	Mus musculus protein tyrosine phosphatase 4a3 (Ptp4a3), mRNA.
scl26557.25_455-S	-89.7499	-2.78	Recc1	NM_011258.1	Mus musculus replication factor C 1 (Recc1), mRNA.
scl46304.5.1_86-S	-87.8353	-1.89	Mrpl52	NM_026851.1	Mus musculus mitochondrial ribosomal protein L52 (Mrpl52), mRNA.
scl28063.9.402_1-S	-87.4735	-2.32	Psmc2	NM_011188.1	Mus musculus proteasome (prosome, macropain) 26S subunit, ATPase 2 (Psmc2), mRNA.
scl0001433.1_54-S	-87.3486	-2.12	Psmc5	NM_008950.1	Mus musculus protease (prosome, macropain) 26S subunit, ATPase 5 (Psmc5), mRNA.
scl017470.1_35-S	-86.7864	-2.23	Cd200	NM_010818	
scl42037.39_16-S	-85.2865	-4.65	Cdc42bpb	NM_183016.1	Mus musculus Cdc42 binding protein kinase beta (Cdc42bpb), mRNA.
scl45915.9_517-S	-84.9487	-2.16	Dnase1l3	NM_007870.2	Mus musculus deoxyribonuclease 1-like 3 (Dnase1l3), mRNA.
scl011984.1_128-S	-84.6424	-4.77	Atp6v0c	NM_009729.1	Mus musculus ATPase, H+ transporting, V0 subunit C (Atp6v0c), mRNA.
scl00018.1_3-S	-82.292	-2.44	Ptpre	NM_011212.2	Mus musculus protein tyrosine phosphatase, receptor type, E (Ptpre), mRNA.
scl0269261.4_12-S	-82.2104	-1.63	Rpl12	NM_009076	Mus musculus ribosomal protein L12 (Rpl12), mRNA.
scl0002332.1_7-S	-82.2099	-1.91	Dld	NM_007861	Mus musculus dihydrolipoamide dehydrogenase (Dld), mRNA.
scl0015129.1_300-S	-81.4349	-2.65	Hbb-b1	NM_008220.2	Mus musculus hemoglobin, beta adult major chain (Hbb-b1), mRNA.
scl0012301.2_7-S	-76.367	-1.94	Cacybp	NM_009786.1	Mus musculus calcyclin binding protein (Cacybp), mRNA.
scl53421.25_339-S	-74.0469	-1.86	D19Ertd703e	NM_029456.1	Mus musculus DNA segment, Chr 19, ERATO Doi 703, expressed (D19Ertd703e), mRNA.
scl48818.18.1_43-S	-73.8371	-1.75	Trap1	NM_026508.1	
scl022147.3_29-S	-73.6889	-2.77	Tuba7	NM_009449.2	Mus musculus tubulin, alpha 7 (Tuba7), mRNA.
scl066167.1_147-S	-72.6542	-1.98	1110017O22Rik	NM_183250.1	Mus musculus RIKEN cDNA 1110017O22 gene (1110017O22Rik), mRNA.
scl068612.6_90-S	-72.2193	-2.87	Ube2c	NM_026785.1	Mus musculus ubiquitin-conjugating enzyme E2C (Ube2c), mRNA.
scl18338.8_547-S	-69.3786	-1.88	Ncoa5	NM_144892.1	Mus musculus nuclear receptor coactivator 5 (Ncoa5), mRNA.
scl31799.8.1_22-S	-68.8287	-6.28	Ube2s	NM_133777.1	Mus musculus ubiquitin-conjugating enzyme E2S (Ube2s), mRNA.
scl47211.15_135-S	-68.6322	-2.33	Rad21	NM_009009.2	Mus musculus RAD21 homolog (S. pombe) (Rad21), mRNA.
scl020638.2_15-S	-68.5265	-2.25	Snrpb	NM_009225.1	Mus musculus small nuclear ribonucleoprotein B (Snrpb), mRNA.
scl0001631.1_196-S	-68.2634	-1.93	Rpo1-1	NM_009085.1	Mus musculus RNA polymerase 1-1 (Rpo1-1), mRNA.
scl40850.12.1_163-S	-68.1587	-1.63	Nmt1	NM_008707.2	Mus musculus N-myristoyltransferase 1 (Nmt1), mRNA.
scl54158.7.21_44-S	-67.8759	-4.91	Bcap31	NM_012060.2	Mus musculus B-cell receptor-associated protein 31 (Bcap31), mRNA.
scl014998.4_34-S	-65.6074	-2.29	H2-DMa	NM_010386	Mus musculus histocompatibility 2, class II, locus DMa (H2-DMa), mRNA.
scl074410.2_34-S	-65.5389	-2.28	4932702F08Rik	XM_130221.2	Mus musculus RIKEN cDNA 4932702F08 gene (4932702F08Rik), mRNA.
scl0067151.2_31-S	-64.997	-4.65	Psmd9	NM_026000.1	Mus musculus proteasome (prosome, macropain) 26S subunit, non-ATPase, 9 (Psmd9), mRNA.
scl012294.1_10-S	-64.1687	-8.18	Cacna2d3	NM_009785.1	Mus musculus calcium channel, voltage dependent, alpha2/delta subunit 3 (Cacna2d3), mRNA.
scl33602.12.1_74-S	-64.0774	-1.8	Ddx39	NM_197982.2	Mus musculus DEAD (Asp-Glu-Ala-Asp) box polypeptide 39 (Ddx39), mRNA.
scl34935.10.1_29-S	-63.7951	-4.69	Dctn6	NM_011722.1	Mus musculus dynactin 6 (Dctn6), mRNA.
scl40203.12_25-S	-63.3698	-1.67	Sparc	NM_009242	Mus musculus secreted acidic cysteine rich glycoprotein (Sparc), mRNA.
scl35917.28_89-S	-63.2954	-2.07	BC023957	NM_172257.1	
scl056791.4_30-S	-62.7378	-3.26	Ube2l6	NM_019949.1	Mus musculus ubiquitin-conjugating enzyme E2L 6 (Ube2l6), mRNA.
scl00110052.1_94-S	-61.3876	-3.36	Dek	NM_025900.1	Mus musculus DEK oncogene (DNA binding) (Dek), mRNA.
scl021665.1_179-S	-60.9837	-2.23	Tdg	NM_172552.1	Mus musculus thymine DNA glycosylase (Tdg), mRNA.
scl0234388.1_36-S	-60.5798	-3	1810023B24Rik	XM_134222.1	
scl072042.2_8-S	-60.3865	-1.96	Cotl1	XM_150115.1	Mus musculus coactosin-like 1 (Dictyostelium) (Cotl1), mRNA.
scl0026442.1_315-S	-60.3832	-2.29	Psma5	NM_011967.1	Mus musculus proteasome (prosome, macropain) subunit, alpha type 5 (Psma5), mRNA.
scl20390.11.1_18-S	-59.6439	-1.97	Ccndbp1	NM_010761.1	Mus musculus cyclin D-type binding-protein 1 (Ccndbp1), mRNA.
scl013034.10_9-S	-59.5635	-2.1	Ctse	NM_007799	Mus musculus cathepsin E (Ctse), mRNA.
scl0057296.1_256-S	-59.4718	-1.71	Psmd8	NM_026545.1	Mus musculus proteasome (prosome, macropain) 26S subunit, non-ATPase, 8 (Psmd8), mRNA.
scl015382.13_13-S	-59.4152	-1.58	Hnrpa1	NM_010447	Mus musculus heterogeneous nuclear ribonucleoprotein A1 (Hnrpa1), mRNA.
scl015040.2_130-S	-59.2632	-2.11	H2-T23	NM_010398	Mus musculus histocompatibility 2, T region locus 23 (H2-T23), mRNA.
scl19818.14.1_56-S	-59.0092	-2.45	Th1l	NM_020580.1	Mus musculus TH1-like homolog (Drosophila) (Th1l), mRNA.
scl38880.29_102-S	-58.2582	-1.71	Psap	NM_011179	Mus musculus prosaposin (Psap), mRNA.
scl39607.11_182-S	-58.2246	-2.26	Smarce1	NM_020618.3	Mus musculus SWI/SNF related, matrix associated, actin dependent regulator of chromatin, subfamily e, member 1 (Smarce1), mRNA.
scl40689.20_176-S	-58.0881	-22.74	39334	NM_017380.1	Mus musculus septin 9 (Sept9), mRNA.
scl014980.6_15-S	-57.8918	-2.28	H2-L		
scl000176.1_12-S	-57.4369	-2.71	Psma1	NM_011965	Mus musculus proteasome (prosome, macropain) subunit, alpha type 1 (Psma1), mRNA.
scl20055.4.1_10-S	-56.9017	-1.68	Dncl2a	NM_025947.1	Mus musculus dynein, cytoplasmic, light chain 2A (Dncl2a), mRNA.

scl32353.2.4_40-S	-56.1456	-1.91	Ndufc2	NM_024220.1	
scl00229534.2_23-S	-56.1275	-3.76	Pbxip1	NM_146131.1	Mus musculus pre-B-cell leukemia transcription factor interacting protein 1 (Pbxip1), mRNA.
scl0056491.1_59-S	-55.9859	-2.3	Vapb	NM_019806.3	Mus musculus vesicle-associated membrane protein, associated protein B and C (Vapb), mRNA.
scl0066748.2_33-S	-55.9431	-2.02	4933404M02Rik	NM_025744.1	Mus musculus RIKEN cDNA 4933404M02 gene (4933404M02Rik), mRNA.
scl52080.7.1_1-S	-55.5265	-14.13	2410080P20Rik	NM_026464.2	Mus musculus RIKEN cDNA 2410080P20 gene (2410080P20Rik), mRNA.
scl0268449.4_2-S	-55.4769	-1.56	Rpl23a	NM_207523	
scl066506.1_15-S	-55.2828	-6.93	1810042K04Rik	NM_025604.1	Mus musculus RIKEN cDNA 1810042K04 gene (1810042K04Rik), mRNA.
scl0003766.1_1206-S	-54.9477	-3.84	Mknk2	NM_021462	Mus musculus MAP kinase-interacting serine/threonine kinase 2 (Mknk2), mRNA.
scl0077038.2_139-S	-54.7262	-2.1	Zfp289	NM_023854.1	Mus musculus zinc finger protein 289 (Zfp289), mRNA.
scl0021974.1_259-S	-54.6431	-1.69	Top2b	NM_009409.2	Mus musculus topoisomerase (DNA) II beta (Top2b), mRNA.
scl32920.6.1_10-S	-54.1229	-2.33	Tgfb1	NM_011577.1	Mus musculus transforming growth factor, beta 1 (Tgfb1), mRNA.
scl38665.9.1_23-S	-54.0405	-1.56	Map2k2	NM_023138.3	Mus musculus mitogen activated protein kinase kinase 2 (Map2k2), mRNA.
scl024075.4_0-S	-53.9991	-1.58	Taf10	NM_020024.3	Mus musculus TAF10 RNA polymerase II, TATA box binding protein (TBP)-associated factor (Taf10), mRNA.
scl020810.7_29-S	-53.6394	-4.22	Srm	NM_009272.2	Mus musculus spermidine synthase (Srm), mRNA.
scl0018220.1_39-S	-53.2818	-1.8	Nucb1	NM_008749.1	
scl0066377.1_24-S	-53.1874	-1.7	Ndufc1	NM_025523.1	Mus musculus NADH dehydrogenase (ubiquinone) 1, subcomplex unknown, 1 (Ndufc1), mRNA.
scl00233908.1_1058-S	-53.0643	-2.33	Fus	NM_139149.1	Mus musculus fusion, derived from t(12;16) malignant liposarcoma (human) (Fus), mRNA.
scl49191.18.1_6-S	-52.7815	-3.63	Sema5b	XM_147227.1	Mus musculus sema domain, seven thrombospondin repeats (type 1 and type 1-like), transmembrane domain (TM) and short cytoplasmic domain, (semaphorin) 5B (Sema5b), mRNA.
scl0012514.2_91-S	-52.5194	-3.31	Cd68	NM_009853.1	Mus musculus CD68 antigen (Cd68), mRNA.
scl19447.7.1_15-S	-52.1682	-1.57	2900010J23Rik	NM_175190.2	Mus musculus RIKEN cDNA 2900010J23 gene (2900010J23Rik), mRNA.
scl0019087.2_280-S	-52.1606	-2.46	Prkar2a	NM_008924.1	Mus musculus protein kinase, cAMP dependent regulatory, type II alpha (Prkar2a), mRNA.
scl22501.2_29-S	-52.1031	-3.12	39340	NM_053102	Mus musculus selenoprotein (Sep15), mRNA.
scl17471.3_7-S	-51.7663	-1.79	Ppp1r15b	XM_148953.1	Mus musculus protein phosphatase 1, regulatory (inhibitor) subunit 15b (Ppp1r15b), mRNA.
scl29109.11_491-S	-51.7635	-1.56	Mkrn1	NM_018810.1	Mus musculus makorin, ring finger protein, 1 (Mkrn1), mRNA.
scl015528.3_20-S	-51.3776	-1.58	Hspe1	NM_008303.2	Mus musculus heat shock protein 1 (chaperonin 10) (Hspe1), mRNA.
scl000794.1_5-S	-50.6965	-1.61	Tsn	NM_011650.2	Mus musculus translin (Tsn), mRNA.
scl014376.17_49-S	-49.533	-1.55	Ganab	NM_008060.1	
scl0003301.1_436-S	-49.4265	-14.2	Nfs1	NM_010911.1	Mus musculus nitrogen fixation gene 1 (S. cerevisiae) (Nfs1), mRNA.
scl44070.4.1_3-S	-49.3173	-2.01	Eef1e1	NM_025380.1	Mus musculus eukaryotic translation elongation factor 1 epsilon 1 (Eef1e1), mRNA.
scl080876.2_10-S	-48.5893	-1.81	Ifitm2	NM_030694	Mus musculus interferon induced transmembrane protein 2 (Ifitm2), mRNA.
scl27115.7.1_28-S	-48.4197	-1.63	Ttc11	NM_025562.1	Mus musculus tetratricopeptide repeat domain 11 (Ttc11), mRNA.
scl068077.3_0-S	-48.3097	-1.63	Gltscr2	NM_133831.1	Mus musculus glioma tumor suppressor candidate region gene 2 (Gltscr2), mRNA.
scl0218490.3_0-S	-47.9279	-1.88	Btf3	NM_145455.1	Mus musculus basic transcription factor 3 (Btf3), mRNA.
scl020813.1_157-S	-47.5379	-1.52	Srp14	NM_009273.2	Mus musculus signal recognition particle 14 (Srp14), mRNA.
scl012757.9_180-S	-47.4875	-1.76	Clta	NM_016760.1	Mus musculus clathrin, light polypeptide (Lca) (Clta), mRNA.
scl019988.6_33-S	-47.05	-1.57	Rpl6	NM_011290	Mus musculus ribosomal protein L6 (Rpl6), mRNA.
scl0068198.2_271-S	-47.0452	-3.56	Ndufb2	NM_026612.1	Mus musculus NADH dehydrogenase (ubiquinone) 1 beta subcomplex, 2 (Ndufb2), mRNA.
scl000673.1_3-S	-46.6585	-1.48	Prdx2	NM_011563	Mus musculus peroxiredoxin 2 (Prdx2), mRNA.
scl058810.1_30-S	-46.64	-1.55	Akr1a4	NM_021473.2	Mus musculus aldo-keto reductase family 1, member A4 (aldehyde reductase) (Akr1a4), mRNA.
scl029864.1_22-S	-46.5341	-1.52	Rnf11	NM_013876.2	Mus musculus ring finger protein 11 (Rnf11), mRNA.
scl50134.9_8-S	-46.3203	-2.02	Nudt3	NM_019837.1	Mus musculus nudix (nucleotide diphosphate linked moiety X)-type motif 3 (Nudt3), mRNA.
scl48206.19.77_2-S	-46.1897	-1.91	Gart	NM_010256.1	Mus musculus phosphoribosylglycinamide formyltransferase (Gart), mRNA.
scl0192292.18_41-S	-45.8657	-10.55	Nrbp	NM_147201.1	Mus musculus nuclear receptor binding protein (Nrbp), mRNA.
scl49830.2.1_22-S	-45.7423	-2.87	Bzrpl1	NM_027292.1	Mus musculus benzodiazapine receptor, peripheral-like 1 (Bzrpl1), mRNA.
scl40144.28.1_2-S	-45.2545	-2.53	Fliih	NM_022009.1	Mus musculus flightless I homolog (Drosophila) (Fliih), mRNA.
scl38191.11.1_70-S	-45.1359	-3.82	2610020N02Rik	NM_181470.2	Mus musculus RIKEN cDNA 2610020N02 gene (2610020N02Rik), mRNA.
GI_21746160-S	-45.0741	-3.86	2410129E14Rik	NM_023716.1	Mus musculus RIKEN cDNA 2410129E14 gene (2410129E14Rik), mRNA.
scl0017938.1_31-S	-44.9736	-3.26	Naca	NM_013608.2	Mus musculus nascent polypeptide-associated complex alpha polypeptide (Naca), mRNA.
scl30947.3_219-S	-44.7465	-1.8	Rhog	NM_019566.2	Mus musculus ras homolog gene family, member G (Rhog), mRNA.
scl42936.6.9_32-S	-44.4301	-1.71	Ahsa1	NM_146036.1	Mus musculus AHA1, activator of heat shock 90kDa protein ATPase homolog 1 (yeast) (Ahsa1), mRNA.
scl021770.4_4-S	-44.3465	-2.21	Ppp2r5d	NM_009358.2	Mus musculus protein phosphatase 2, regulatory subunit B (B56), delta isoform (Ppp2r5d), mRNA.
scl55019.13_214-S	-43.954	-3.25	Pctk1	NM_011049.2	Mus musculus PCTAIRE-motif protein kinase 1 (Pctk1), mRNA.
scl38604.10.1_9-S	-43.9307	-2.64	Fbxo7	NM_153195.1	Mus musculus F-box only protein 7 (Fbxo7), mRNA.
scl067674.3_12-S	-43.7274	-1.93	0610038D11Rik	NM_026306.1	Mus musculus RIKEN cDNA 0610038D11 gene (0610038D11Rik), mRNA.
scl21701.9.1_3-S	-43.6948	-1.95	Atp5f1	NM_009725.1	Mus musculus ATP synthase, H+ transporting, mitochondrial F0 complex, subunit b, isoform 1 (Atp5f1), mRNA.
scl000330.1_8-S	-43.3607	-1.87	Pdlim2	NM_145978.1	Mus musculus PDZ and LIM domain 2 (Pdlim2), mRNA.

scl50733.5.1_21-S	-42.9037	-2.41	H2-M3	NM_013819.1	Mus musculus histocompatibility 2, M region locus 3 (H2-M3), mRNA.
scl00264064.2_74-S	-42.8729	-5.52	Cdk8	NM_181570.1	Mus musculus cyclin-dependent kinase 8 (Cdk8), transcript variant 1, mRNA.
scl25185.2.323_16-S	-42.8588	-3.29	Ppap2b	NM_080555.1	Mus musculus phosphatidic acid phosphatase type 2B (Ppap2b), mRNA.
scl0002315.1_12-S	-42.7778	-3.14		XM_358429.1	Mus musculus LOC380788 (LOC380788), mRNA.
scl29713.3_34-S	-42.3009	-1.62	Arl6ip5	NM_022992.1	Mus musculus ADP-ribosylation factor-like 6 interacting protein 5 (Arl6ip5), mRNA.
scl019045.1_99-S	-42.2202	-1.49	Ppp1ca	NM_031868.1	Mus musculus protein phosphatase 1, catalytic subunit, alpha isoform (Ppp1ca), mRNA.
scl067278.1_10-S	-42.0556	-2.4	2900092E17Rik	NM_030240.1	Mus musculus RIKEN cDNA 2900092E17 gene (2900092E17Rik), mRNA.
scl36516.9.290_30-S	-41.6761	-1.69	Ptk9l	NM_011876.2	Mus musculus protein tyrosine kinase 9-like (A6-related protein) (Ptk9l), mRNA.
scl43224.11.1_2-S	-41.5214	-2.4	Coch	NM_007728.2	Mus musculus coagulation factor C homolog (Limulus polyphemus) (Coch), mRNA.
scl020443.1_125-S	-41.5042	-3.11	Siat4c	NM_009178.2	Mus musculus sialyltransferase 4C (beta-galactoside alpha-2,3-sialytransferase) (Siat4c), mRNA.
scl0015260.1_245-S	-41.3557	-2.85	Hira	NM_010435.1	Mus musculus histone cell cycle regulation defective homolog A (S. cerevisiae) (Hira), mRNA.
scl0014425.2_292-S	-41.3141	-9.84	Galnt3	NM_015736.1	Mus musculus UDP-N-acetyl-alpha-D-galactosamine:polypeptide N-acetylgalactosaminyltransferase 3 (Galnt3), mRNA.
scl0228608.7_126-S	-41.064	-1.43	Smox	NM_145533.1	Mus musculus spermine oxidase (Smox), mRNA.
scl45439.8_694-S	-41.0446	-10.58	Gata4	NM_008092.2	Mus musculus GATA binding protein 4 (Gata4), mRNA.
scl056348.1_132-S	-40.9127	-1.9	Hsd17b12	NM_019657.2	Mus musculus hydroxysteroid (17-beta) dehydrogenase 12 (Hsd17b12), mRNA.
scl46411.5.1_45-S	-40.8555	-4.25	1810009H17Rik	XM_283205.1	
scl017938.7_45-S	-40.656	-1.6	Naca	NM_013608.2	Mus musculus nascent polypeptide-associated complex alpha polypeptide (Naca), mRNA.
scl022629.4_2-S	-40.5874	-1.84	Ywhah	NM_011738	Mus musculus tyrosine 3-monooxygenase/tryptophan 5-monooxygenase activation protein, eta polypeptide (Ywhah), mRNA.
scl42971.1_9-S	-40.5292	-1.54	0710001C05Rik	XM_203592.1	Mus musculus RIKEN cDNA 0710001C05 gene (0710001C05Rik), mRNA.
scl00223696.2_201-S	-40.5185	-1.88	Tomm22	NM_172609.2	Mus musculus translocase of outer mitochondrial membrane 22 homolog (yeast) (Tomm22), mRNA.
scl00276770.1_2-S	-40.3271	-2.43	Eif5a	NM_181582.2	Mus musculus eukaryotic translation initiation factor 5A (Eif5a), mRNA.
scl52637.16.1_4-S	-40.0973	-2.35	Pip5k1a	NM_008846.1	Mus musculus phosphatidylinositol-4-phosphate 5-kinase, type 1 alpha (Pip5k1a), mRNA.
scl46497.11.10_2-S	-40.0862	-7.74	2510015F01Rik	XM_354801.1	Mus musculus RIKEN cDNA 2510015F01 gene (2510015F01Rik), mRNA.
scl52675.8.401_6-S	-40.063	-1.52	Ostf1	NM_017375.1	Mus musculus osteoclast stimulating factor 1 (Ostf1), mRNA.
scl35222.4_425-S	-40.0099	-2.02	Myd88	NM_010851	Mus musculus myeloid differentiation primary response gene 88 (Myd88), mRNA.
scl26406.12_265-S	-40.0077	-1.76	Grsf1	NM_178700.2	Mus musculus G-rich RNA sequence binding factor 1 (Grsf1), mRNA.
scl015510.1_74-S	-39.9469	-1.55	Hspd1	NM_010477.2	Mus musculus heat shock protein 1 (chaperonin) (Hspd1), mRNA.
scl0003793.1_0-S	-39.6945	-1.93	Metap2	NM_019648.2	Mus musculus methionine aminopeptidase 2 (Metap2), mRNA.
scl071780.11_24-S	-39.634	-8.71	Isyna1	NM_023627.1	Mus musculus myo-inositol 1-phosphate synthase A1 (Isyna1), mRNA.
scl41564.10_42-S	-39.5743	-2.05	39333	NM_033144.1	Mus musculus septin 8 (Sept8), mRNA.
scl53104.2_253-S	-39.5678	-3.24	Nkx2-3	XM_129268.4	Mus musculus NK2 transcription factor related, locus 3 (Drosophila) (Nkx2-3), mRNA.
scl0016195.2_10-S	-39.5187	-7.87	Il6st	NM_010560.2	Mus musculus interleukin 6 signal transducer (Il6st), mRNA.
scl0020788.1_174-S	-39.4169	-1.87	Srebp2	XM_127995.4	
scl000245.1_108-S	-39.0679	-1.96	Irf3	NM_016849.2	Mus musculus interferon regulatory factor 3 (Irf3), mRNA.
scl0004033.1_10-S	-39.0445	-1.85	Arpc1a	NM_019767.1	Mus musculus actin related protein 2/3 complex, subunit 1A (Arpc1a), mRNA.
scl056436.9_4-S	-38.9735	-1.7	Adrm1	NM_019822.2	Mus musculus adhesion regulating molecule 1 (Adrm1), mRNA.
scl35215.8_496-S	-38.6955	-11.66	Axud1	NM_153287.2	Mus musculus AXIN1 up-regulated 1 (Axud1), mRNA.
scl36686.18.1_100-S	-38.6235	-1.88	Gclc	NM_010295.1	Mus musculus glutamate-cysteine ligase, catalytic subunit (Gclc), mRNA.
scl41225.47_412-S	-38.5233	-14.69	Myo18a	NM_011586.1	Mus musculus myosin XVIIIa (Myo18a), mRNA.
scl064656.5_1-S	-38.5087	-1.7	Mrps23	NM_024174.4	Mus musculus mitochondrial ribosomal protein S23 (Mrps23), mRNA.
scl21185.13.1_16-S	-38.4442	-1.53	Anapc2	NM_175300.2	Mus musculus anaphase promoting complex subunit 2 (Anapc2), mRNA.
scl067885.2_20-S	-38.4151	-3.05	1500011K16Rik	NM_175125.2	Mus musculus RIKEN cDNA 1500011K16 gene (1500011K16Rik), mRNA.
scl000533.1_30-S	-38.3962	-2.04	Yif1	NM_026553.2	Mus musculus Yip1 interacting factor homolog (S. cerevisiae) (Yif1), mRNA.
scl21422.13.1_102-S	-38.3601	-13.19	Clca1	NM_009899.2	Mus musculus chloride channel calcium activated 1 (Clca1), mRNA.
scl34048.16.227_13-S	-38.1977	-3.41	Cdc16	NM_027276.1	Mus musculus CDC16 cell division cycle 16 homolog (S. cerevisiae) (Cdc16), mRNA.
scl23453.4_99-S	-37.9259	-1.46	1200015A19Rik	NM_026388.1	Mus musculus RIKEN cDNA 1200015A19 gene (1200015A19Rik), mRNA.
scl45278.7.1_11-S	-37.8869	-10.73	Dnajd1	NM_025384.3	Mus musculus DnaJ (Hsp40) homolog, subfamily D, member 1 (Dnajd1), mRNA.
scl37744.8.1_11-S	-37.6886	-1.55	ORF61	XM_109575.3	Mus musculus open reading frame 61 (ORF61), mRNA.
scl018245.5_1-S	-37.6678	-1.55	Oaz1	NM_008753	Mus musculus ornithine decarboxylase antizyme (Oaz1), mRNA.
scl0012946.2_160-S	-37.6598	-1.54	Crry	NM_013499.1	Mus musculus complement receptor related protein (Crry), mRNA.
scl0001137.1_14-S	-37.4518	-6.74	Mrps25	NM_025578.2	Mus musculus mitochondrial ribosomal protein S25 (Mrps25), mRNA.
scl42112.9.1_0-S	-37.2485	-1.54	Ddx24	NM_020494.2	Mus musculus DEAD (Asp-Glu-Ala-Asp) box polypeptide 24 (Ddx24), mRNA.
scl34884.5.1_26-S	-37.2099	-9.9	Frg1	NM_013522.1	Mus musculus FSHD region gene 1 (Frg1), mRNA.
scl068183.7_27-S	-37.1865	-1.47	Bcas2	NM_026602.1	Mus musculus breast carcinoma amplified sequence 2 (Bcas2), mRNA.
scl18407.1_27-S	-37.0165	-5.8	Mafb	NM_010658.2	Mus musculus v-maf musculoaponeurotic fibrosarcoma oncogene family, protein B (avian) (Mafb), mRNA.
scl0328601.1_103-S	-37.0102	-1.52	Ccnt1	XM_286697.2	Mus musculus cyclin T1 (Ccnt1), mRNA.

scl0114887.6_24-S	-36.9359	-3	Rmcs1	NM_207105.1	Mus musculus response to metastatic cancers 1 (Rmcs1), mRNA.
scl0056045.1_160-S	-36.8985	-1.64	Samhd1	NM_018851.2	Mus musculus SAM domain and HD domain, 1 (Samhd1), mRNA.
scl066483.1_326-S	-36.8122	-1.51	Rpl36al	NM_025589.1	Mus musculus ribosomal protein L36a-like (Rpl36al), mRNA.
scl0013829.2_259-S	-36.7256	-1.65	Epb4.9	NM_013514.2	Mus musculus erythrocyte protein band 4.9 (Epb4.9), mRNA.
scl0001687.1_10-S	-36.4031	-1.92	Mylc2b	NM_023402	
scl0268373.4_12-S	-36.2438	-1.44	Ppia	NM_008907	Mus musculus peptidylprolyl isomerase A (Ppia), mRNA.
scl0059069.1_170-S	-35.4541	-1.36	Tpm3	NM_022314.2	Mus musculus tropomyosin 3, gamma (Tpm3), mRNA.
scl056455.3_23-S	-35.3549	-1.54	Dnclc1	NM_019682	Mus musculus dynein, cytoplasmic, light chain 1 (Dnclc1), mRNA.
IGKV8-26_AJ235945_Ig_	-35.3233	-3.21			
scl20009.15.26_12-S	-35.1062	-1.8	Ctnnbl1	NM_025680.2	Mus musculus catenin, beta like 1 (Ctnnbl1), mRNA.
scl20983.4_177-S	-35.0441	-2.18	Arpc5l	NM_028809.1	Mus musculus actin related protein 2/3 complex, subunit 5-like (Arpc5l), mRNA.
scl0014312.2_62-S	-35.0383	-1.43	Brd2	NM_010238.1	Mus musculus bromodomain containing 2 (Brd2), mRNA.
scl014894.1_227-S	-34.8969	-5.08	Gtl3	NM_008187.1	Mus musculus gene trap locus 3 (Gtl3), mRNA.
scl49476.8.9_25-S	-34.8601	-1.88	Hmox2	NM_010443.1	Mus musculus heme oxygenase (decycling) 2 (Hmox2), mRNA.
scl51506.2_48-S	-34.7338	-2.2	Cd14	NM_009841.2	Mus musculus CD14 antigen (Cd14), mRNA.
scl000196.1_9-S	-34.6243	-3.74	Actn4	NM_021895.2	Mus musculus actinin alpha 4 (Actn4), mRNA.
scl0321022.1_67-S	-34.6157	-1.37	Cdv3	NM_175833.1	Mus musculus carnitine deficiency-associated gene expressed in ventricle 3 (Cdv3), mRNA.
scl32052.6.1_83-S	-34.4859	-3.27	Ypel3	NM_025347.1	
scl38822.17.1_2-S	-34.4391	-8.61	Jmjd1c	NM_207221.1	
scl33416.4.1_0-S	-34.3863	-1.49	Hspc171	NM_025486.1	
scl31568.26.194_22-S	-34.3758	-1.5	Actn4	NM_021895.2	Mus musculus actinin alpha 4 (Actn4), mRNA.
IGKV12-67_AJ235933_Ig	-34.3712	-2.41			
scl42795.16.1_26-S	-34.2984	-1.46	Evl	NM_007965.2	Mus musculus Ena-vasodilator stimulated phosphoprotein (Evl), mRNA.
scl0110557.2_204-S	-34.2792	-1.82	H2-Q6	NM_207648	Mus musculus histocompatibility 2, Q region locus 6 (H2-Q6), mRNA.
scl52692.1_233-S	-34.2707	-1.44	Eif4a1	NM_144958	Mus musculus eukaryotic translation initiation factor 4A1 (Eif4a1), mRNA.
scl38886.15_179-S	-34.2194	-6.09	Cbara1	NM_144822.1	Mus musculus calcium binding atopy-related autoantigen 1 (Cbara1), mRNA.
scl32817.6.1_8-S	-34.1182	-1.45	Tyrobp	NM_011662.2	Mus musculus TYRO protein tyrosine kinase binding protein (Tyrobp), mRNA.
scl26190.16_67-S	-34.0936	-1.41	Coro1c	NM_011779.2	Mus musculus coronin, actin binding protein 1C (Coro1c), mRNA.
scl0001446.1_126-S	-34.0764	-1.75	Gosr2	NM_019650.2	Mus musculus golgi SNAP receptor complex member 2 (Gosr2), mRNA.
scl0053607.2_231-S	-33.9527	-2.08	Snrpa	NM_015782.2	Mus musculus small nuclear ribonucleoprotein polypeptide A (Snrpa), mRNA.
scl50849.26.1_265-S	-33.9199	-1.63	Myo1f	NM_053214.1	Mus musculus myosin IF (Myo1f), mRNA.
scl057437.1_41-S	-33.802	-3.77	Golga7	NM_020585.1	Mus musculus golgi autoantigen, golgin subfamily a, 7 (Golga7), mRNA.
scl25278.23_305-S	-33.7831	-8.47	Tek	NM_013690.1	Mus musculus endothelial-specific receptor tyrosine kinase (Tek), mRNA.
scl41383.7.120_88-S	-33.7022	-1.61	Aurkb	XM_181344.3	Mus musculus aurora kinase B (Aurkb), mRNA.
scl018727.3_44-S	-33.5866	-4.89	Pira4	NM_011091	Mus musculus paired-Ig-like receptor A4 (Pira4), mRNA.
scl056351.11_60-S	-33.5452	-1.76	Tebp	NM_019766.2	Mus musculus telomerase binding protein, p23 (Tebp), mRNA.
scl0002678.1_34-S	-33.5062	-1.71	Magoh	NM_010760.1	Mus musculus mago-nashi homolog, proliferation-associated (Drosophila) (Magoh), mRNA.
scl24944.2_480-S	-33.5008	-1.69	BC003266	NM_030252.1	Mus musculus cDNA sequence BC003266 (BC003266), mRNA.
scl22236.8_4-S	-33.1368	-1.49	Ccna2	NM_009828.1	Mus musculus cyclin A2 (Ccna2), mRNA.
scl077134.2_4-S	-33.0533	-1.96	Hnrpa0		
scl54435.12.1_121-S	-33.0128	-1.38	Was	NM_009515.1	Mus musculus Wiskott-Aldrich syndrome homolog (human) (Was), mRNA.
scl00110842.1_108-S	-32.7732	-1.57	Etfa	NM_145615.2	Mus musculus electron transferring flavoprotein, alpha polypeptide (Etfa), nuclear gene encoding mitochondrial protein, mRNA.
scl50206.42.1_126-S	-32.7507	-2	Tsc2	NM_011647	Mus musculus tuberous sclerosis 2 (Tsc2), mRNA.
scl0002187.1_532-S	-32.6114	-1.55	Rab18	NM_181070.2	Mus musculus RAB18, member RAS oncogene family (Rab18), mRNA.
scl47039.3.1_28-S	-32.5906	-2.7	Fbxl6	NM_013909.1	Mus musculus F-box and leucine-rich repeat protein 6 (Fbxl6), mRNA.
scl071514.8_14-S	-32.5338	-1.39	Sfpq	NM_023603.1	Mus musculus splicing factor proline/glutamine rich (polypyrimidine tract binding protein associated) (Sfpq), mRNA.
scl0116891.1_127-S	-32.4455	-1.78	BC005682	NM_033562.2	Mus musculus cDNA sequence BC005682 (BC005682), mRNA.
scl056620.6_30-S	-32.3942	-1.6	Clecsf10	NM_020001.1	Mus musculus C-type (calcium dependent, carbohydrate recognition domain) lectin, superfamily member 10 (Clecsf10), mRNA.
scl00107250.1_231-S	-32.3759	-2.33	Kazald1	NM_178929.2	
scl011816.1_11-S	-32.2386	-2.03	Apoe	NM_009696.2	Mus musculus apolipoprotein E (Apoe), mRNA.
scl0381974.1_329-S	-32.2379	-9.21	Mrgprg	NM_203492.1	Mus musculus MAS-related GPR, member G (Mrgprg), mRNA.
scl0004139.1_41-S	-32.2261	-2.71	Asrij	NM_023429.2	Mus musculus asrij protein (Asrij), mRNA.
scl50198.3.1_17-S	-32.1762	-1.49	Ndufb10	XM_128594.4	Mus musculus NADH dehydrogenase (ubiquinone) 1 beta subcomplex, 10 (Ndufb10), mRNA.
scl053606.2_17-S	-32.1412	-2.04	G1p2	NM_015783.1	Mus musculus interferon, alpha-inducible protein (G1p2), mRNA.
scl16496.1_30-S	-32.0915	-3.02	Arl7	NM_177305.2	
scl30348.4.1_28-S	-32.0905	-1.52	Lsm8	NM_133939.1	Mus musculus LSM8 homolog, U6 small nuclear RNA associated (S. cerevisiae) (Lsm8), mRNA.

scl34186.14_327-S	-32.0505	-1.72	Abcb10	NM_019552.1	Mus musculus ATP-binding cassette, sub-family B (MDR/TAP), member 10 (Abcb10), nuclear gene encoding mitochondrial protein, mRNA.
scl0001637.1_218-S	-32.0063	-1.81	Luc7l	NM_025881.1	Mus musculus Luc7 homolog (S. cerevisiae)-like (Luc7l), mRNA.
scl41368.8.97_6-S	-31.5463	-1.53	Trp53	NM_011640.1	Mus musculus transformation related protein 53 (Trp53), mRNA.
scl0001821.1_0-S	-31.513	-5.75	Ppp1r2	NM_025800	Mus musculus protein phosphatase 1, regulatory (inhibitor) subunit 2 (Ppp1r2), mRNA.
scl0227292.12_53-S	-31.4678	-1.76	Ctdsp1	NM_153088.1	Mus musculus CTD (carboxy-terminal domain, RNA polymerase II, polypeptide A) small phosphatase 1 (Ctdsp1), mRNA.
scl44304.18_267-S	-31.4666	-1.52	Gtpbp4	NM_027000.2	Mus musculus GTP binding protein 4 (Gtpbp4), mRNA.
scl37876.12_7-S	-31.3934	-2.32	Sirt1	NM_019812.1	Mus musculus sirtuin 1 ((silent mating type information regulation 2, homolog) 1 (S. cerevisiae) (Sirt1), mRNA.
scl0018938.1_81-S	-31.2921	-1.59	Ppp1r14b	NM_008889.1	Mus musculus protein phosphatase 1, regulatory (inhibitor) subunit 14B (Ppp1r14b), mRNA.
scl074255.2_27-S	-31.1574	-1.4	Smu1	NM_021535.2	
scl39693.17_117-S	-31.0403	-1.95	Myst2	NM_177619.2	Mus musculus MYST histone acetyltransferase 2 (Myst2), mRNA.
scl0002638.1_12-S	-31.0365	-8.72	Sdhb	NM_023374.3	Mus musculus succinate dehydrogenase complex, subunit B, iron sulfur (Ip) (Sdhb), mRNA.
scl068020.5_23-S	-30.9953	-1.95	2810002N01Rik	NM_026511.1	Mus musculus RIKEN cDNA 2810002N01 gene (2810002N01Rik), mRNA.
scl020703.2_0-S	-30.9855	-1.57	Serpina1d	NM_009246.1	Mus musculus serine (or cysteine) proteinase inhibitor, clade A, member 1d (Serpina1d), mRNA.
scl0246707.2_7-S	-30.9551	-2.3	Emilin2	NM_145158	Mus musculus elastin microfibril interfacer 2 (Emilin2), mRNA.
scl0170741.3_61-S	-30.8981	-2.21	Pilrb	NM_133209.1	Mus musculus paired immunoglobin-like type 2 receptor beta (Pilrb), mRNA.
scl026941.6_202-S	-30.8039	-1.58	Slc9a3r1	NM_012030.1	Mus musculus solute carrier family 9 (sodium/hydrogen exchanger), isoform 3 regulator 1 (Slc9a3r1), mRNA.
scl066594.3_10-S	-30.7813	-1.53	Uqcr	NM_025650.1	Mus musculus ubiquinol-cytochrome c reductase (6.4kD) subunit (Uqcr), mRNA.
scl0026385.2_311-S	-30.7583	-4.99	Gprk6	NM_011938.1	Mus musculus G protein-coupled receptor kinase 6 (Gprk6), mRNA.
scl37728.2_209-S	-30.7254	-1.88	Klf16	NM_078477	Mus musculus Kruppel-like factor 16 (Klf16), mRNA.
scl066480.2_3-S	-30.6462	-1.47	Rpl15	NM_025586.1	Mus musculus ribosomal protein L15 (Rpl15), mRNA.
scl25881.20.1_16-S	-30.6096	-1.48	Ars2	NM_031405.1	Mus musculus arsenate resistance protein 2 (Ars2), mRNA.
scl054673.4_1-S	-30.607	-1.92	Sh3glb1	NM_019464.1	Mus musculus SH3-domain GRB2-like B1 (endophilin) (Sh3glb1), mRNA.
scl50121.14.1_6-S	-30.3931	-7.95	Tulp1	NM_021478.1	Mus musculus tubby like protein 1 (Tulp1), mRNA.
scl19066.9.1_77-S	-30.3909	-1.75	Serping1	NM_009776	Mus musculus serine (or cysteine) proteinase inhibitor, clade G, member 1 (Serping1), mRNA.
scl50396.10.5_23-S	-30.31	-1.79	Msh6	NM_010830.1	Mus musculus mutS homolog 6 (E. coli) (Msh6), mRNA.
scl43927.8_10-S	-30.2729	-1.72	Lman2	NM_025828.2	Mus musculus lectin, mannose-binding 2 (Lman2), mRNA.
scl022142.1_236-S	-30.222	-1.5	Tuba1	NM_011653	Mus musculus tubulin, alpha 1 (Tuba1), mRNA.
scl0073834.1_147-S	-30.106	-2.64	Atp6v1d	NM_023721.1	Mus musculus ATPase, H+ transporting, V1 subunit D (Atp6v1d), mRNA.
scl0230908.3_49-S	-30.0652	-4.78	Tardbp	NM_145556.2	Mus musculus TAR DNA binding protein (Tardbp), mRNA.
scl32205.4_514-S	-29.9759	-4.8	Rpl27a	NM_011975.2	Mus musculus ribosomal protein L27a (Rpl27a), mRNA.
scl00228359.2_35-S	-29.952	-1.85	Arhgap1	NM_146124.2	Mus musculus Rho GTPase activating protein 1 (Arhgap1), mRNA.
scl0022680.1_25-S	-29.9491	-1.65	Zfp207	NM_011751.1	Mus musculus zinc finger protein 207 (Zfp207), mRNA.
scl23378.7_207-S	-29.8932	-2.21	Car2	NM_009801.3	Mus musculus carbonic anhydrase 2 (Car2), mRNA.
scl068713.2_9-S	-29.8622	-1.52	Ifitm1	NM_026820	Mus musculus interferon induced transmembrane protein 1 (Ifitm1), mRNA.
scl31451.11.1_40-S	-29.7954	-1.92	Ccne1	NM_007633.1	Mus musculus cyclin E1 (Ccne1), mRNA.
scl0237107.1_138-S	-29.7743	-2.04	BC020354	NM_198110.1	Mus musculus cDNA sequence BC020354 (BC020354), mRNA.
scl0026992.2_59-S	-29.7365	-1.54	Brd7	NM_012047.1	Mus musculus bromodomain containing 7 (Brd7), mRNA.
scl20329.42.1_43-S	-29.7065	-1.46	Ascc3l1	NM_177214.3	
scl0001506.1_275-S	-29.6958	-1.81	AA536749	NM_012027.1	Mus musculus expressed sequence AA536749 (AA536749), mRNA.
scl29532.7.1_114-S	-29.6736	-2.52	3110037K17Rik	NM_153197.1	Mus musculus RIKEN cDNA 3110037K17 gene (3110037K17Rik), mRNA.
scl49909.9.1_16-S	-29.6648	-1.5	2610528M18Rik	NM_031863.1	Mus musculus RIKEN cDNA 2610528M18 gene (2610528M18Rik), mRNA.
scl016971.1_277-S	-29.6504	-1.95	Lrp1	NM_008512.1	Mus musculus low density lipoprotein receptor-related protein 1 (Lrp1), mRNA.
scl24360.4_30-S	-29.5717	-1.75	Hemgn	NM_053149.1	Mus musculus hemogen (Hemgn), mRNA.
scl48203.8.22_30-S	-29.5529	-2.84	Donson	NM_021720.1	Mus musculus downstream neighbor of SON (Donson), mRNA.
scl014693.1_109-S	-29.4864	-1.43	Gnb2	NM_010312.3	Mus musculus guanine nucleotide binding protein, beta 2 (Gnb2), mRNA.
scl000275.1_39-S	-29.3752	-1.39	3010033P07Rik	NM_029767.1	Mus musculus RIKEN cDNA 3010033P07 gene (3010033P07Rik), mRNA.
scl0003360.1_0-S	-29.2457	-3.01	Smox	NM_145533.1	Mus musculus spermine oxidase (Smox), mRNA.
scl057808.2_82-S	-29.1598	-1.6	Rpl35a	NM_021338.2	Mus musculus ribosomal protein L35a (Rpl35a), mRNA.
scl0001252.1_55-S	-29.0694	-1.67	Hnrpa2b1	NM_016806	Mus musculus heterogeneous nuclear ribonucleoprotein A2/B1 (Hnrpa2b1), transcript variant 2, mRNA.
scl0001188.1_5-S	-29.0296	-2.42	1810046I24Rik	NM_027218.1	Mus musculus RIKEN cDNA 1810046I24 gene (1810046I24Rik), mRNA.
scl000781.1_68-S	-29.0107	-1.75	Kif21b	NM_019962.2	Mus musculus kinesin family member 21B (Kif21b), mRNA.
scl0003800.1_141-S	-28.9979	-1.58	Tfam	NM_009360.2	Mus musculus transcription factor A, mitochondrial (Tfam), mRNA.
scl50031.6.1_11-S	-28.9742	-3.24	Slc39a7	NM_008202.1	Mus musculus solute carrier family 39 (zinc transporter), member 7 (Slc39a7), mRNA.
scl21129.4_153-S	-28.8754	-6.62	Mrps2	NM_080452.2	Mus musculus mitochondrial ribosomal protein S2 (Mrps2), mRNA.
scl54681.4_59-S	-28.8637	-1.4	Sh3bgrl	NM_019989.2	Mus musculus SH3-binding domain glutamic acid-rich protein like (Sh3bgrl), mRNA.
scl50794.7.1_43-S	-28.8403	-1.35	Clic1	NM_033444.1	Mus musculus chloride intracellular channel 1 (Clic1), mRNA.

scl22797.19_196-S	-28.8275	-1.38	D3Jfr1	NM_144901.2	Mus musculus DNA segment, Chr 3, MJeffers 1 (D3Jfr1), mRNA.
scl0224619.1_310-S	-28.7009	-1.43	Traf7	NM_153792.1	
scl0276770.1_104-S	-28.6391	-1.76	Eif5a	NM_181582.2	Mus musculus eukaryotic translation initiation factor 5A (Eif5a), mRNA.
scl0013211.2_49-S	-28.5963	-2.17	Dhx9	NM_007842.1	Mus musculus DEAH (Asp-Glu-Ala-His) box polypeptide 9 (Dhx9), mRNA.
scl0004133.1_1-S	-28.4513	-2.02	Tyms	NM_021288.2	Mus musculus thymidylate synthase (Tyms), mRNA.
scl0002467.1_77-S	-28.3946	-1.65	Bzrp	NM_009775.2	Mus musculus benzodiazepine receptor, peripheral (Bzrp), mRNA.
scl0080912.2_313-S	-28.34	-4.07	Pum1	NM_030722.1	Mus musculus pumilio 1 (Drosophila) (Pum1), mRNA.
scl020655.4_35-S	-28.3255	-1.49	Sod1	NM_011434.1	Mus musculus superoxide dismutase 1, soluble (Sod1), mRNA.
scl41510.8.35_73-S	-28.2744	-1.49	Butr1	NM_138678.1	Mus musculus butyrophilin related 1 (Butr1), mRNA.
scl0070396.1_30-S	-28.1729	-2.52	2210409M21Rik	NM_133728.2	Mus musculus RIKEN cDNA 2210409M21 gene (2210409M21Rik), mRNA.
scl43644.9.1_5-S	-28.1656	-2.1	Hexb	NM_010422.1	Mus musculus hexosaminidase B (Hexb), mRNA.
scl0016069.1_4-S	-28.0362	-2.69	Igj	NM_152839.1	Mus musculus immunoglobulin joining chain (Igj), mRNA.
scl00231464.2_19-S	-27.9636	-1.79	Cnot6l	NM_144910.1	
scl28749.2.254_68-S	-27.9602	-1.58	Pcbp1	NM_011865.2	Mus musculus poly(rC) binding protein 1 (Pcbp1), mRNA.
scl26219.29_122-S	-27.9308	-1.91	Ulk1	NM_009469.3	Mus musculus Unc-51 like kinase 1 (C. elegans) (Ulk1), mRNA.
scl30883.9.1_56-S	-27.925	-10.42	Arfip2	NM_029802.2	Mus musculus ADP-ribosylation factor interacting protein 2 (Arfip2), mRNA.
scl0068045.1_0-S	-27.8331	-9.86	2700060E02Rik	NM_026528.1	Mus musculus RIKEN cDNA 2700060E02 gene (2700060E02Rik), mRNA.
scl0066881.2_144-S	-27.8292	-1.78	Pcyox1	NM_025823.3	Mus musculus prenylcysteine oxidase 1 (Pcyox1), mRNA.
scl46932.19_335-S	-27.8085	-1.31	Rangap1	NM_011241	Mus musculus RAN GTPase activating protein 1 (Rangap1), mRNA.
scl0210106.1_119-S	-27.8	-2.24	Pols	NM_198600.1	Mus musculus polymerase (DNA directed) sigma (Pols), mRNA.
scl29171.13.1_11-S	-27.7409	-6.3	Chchd3	NM_025336.1	Mus musculus coiled-coil-helix-coiled-coil-helix domain containing 3 (Chchd3), mRNA.
scl012260.3_14-S	-27.738	-1.79	C1qb	NM_009777.1	Mus musculus complement component 1, q subcomponent, beta polypeptide (C1qb), mRNA.
scl0054632.2_248-S	-27.6468	-3.13	Ftsj	NM_133991.2	
scl067201.1_106-S	-27.64	-2.74	2700085E05Rik	NM_026029.1	Mus musculus RIKEN cDNA 2700085E05 gene (2700085E05Rik), mRNA.
scl20876.12.1_41-S	-27.5365	-2.17	Tank	NM_011529.1	Mus musculus TRAF family member-associated Nf-kappa B activator (Tank), mRNA.
scl0072544.1_107-S	-27.3815	-1.89	Exosc6	NM_028274.1	Mus musculus exosome component 6 (Exosc6), mRNA.
scl44866.7.1_13-S	-27.3359	-9.39	Pak1ip1	NM_026550.1	Mus musculus PAK1 interacting protein 1 (Pak1ip1), mRNA.
scl32110.5.1_11-S	-27.3088	-13.51	0610012D09Rik	NM_021554.2	Mus musculus RIKEN cDNA 0610012D09 gene (0610012D09Rik), mRNA.
scl20194.20.1_79-S	-27.2948	-2.18	Sec23b	NM_019787.2	Mus musculus SEC23B (S. cerevisiae) (Sec23b), mRNA.
scl19560.8.1_32-S	-27.2895	-10.54	1700013L23Rik	XM_130127.3	Mus musculus RIKEN cDNA 1700013L23 gene (1700013L23Rik), mRNA.
scl017283.2_24-S	-27.2169	-2.24	Men1	NM_008583.1	Mus musculus multiple endocrine neoplasia 1 (Men1), mRNA.
scl071846.4_6-S	-27.2111	-7.96	1700013H19Rik	NM_027954.1	Mus musculus RIKEN cDNA 1700013H19 gene (1700013H19Rik), mRNA.
scl012988.1_164-S	-27.1934	-1.33	Csk	NM_007783.2	Mus musculus c-src tyrosine kinase (Csk), mRNA.
scl30629.5.1_26-S	-27.1289	-1.88	Vkorc1	NM_178600.2	Mus musculus vitamin K epoxide reductase complex, subunit 1 (Vkorc1), mRNA.
scl00224133.1_32-S	-27.0649	-3.5	Parp14	NM_145481.1	
scl38737.14.1_60-S	-27.055	-1.57	Itgb2	NM_008404.1	Mus musculus integrin beta 2 (Itgb2), mRNA.
scl0002609.1_52-S	-27.0337	-1.94	Psmb2	NM_011970.2	Mus musculus proteasome (prosome, macropain) subunit, beta type 2 (Psmb2), mRNA.
scl50308.28_413-S	-27.0123	-3.35	Map3k4	NM_011948.1	Mus musculus mitogen activated protein kinase kinase kinase 4 (Map3k4), mRNA.
scl056330.3_4-S	-26.9969	-1.38	Pdcd5	NM_019746.2	Mus musculus programmed cell death 5 (Pdcd5), mRNA.
scl24829.3_156-S	-26.9927	-1.99	Cnr2	NM_009924	Mus musculus cannabinoid receptor 2 (macrophage) (Cnr2), mRNA.
scl070356.1_235-S	-26.9666	-1.6	St13	NM_133726.1	Mus musculus suppression of tumorigenicity 13 (St13), mRNA.
scl46751.13_99-S	-26.944	-1.54	Prkag1	NM_016781	Mus musculus protein kinase, AMP-activated, gamma 1 non-catalytic subunit (Prkag1), mRNA.
scl41258.12_129-S	-26.8686	-1.61	Pitpn	NM_008850.1	Mus musculus phosphatidylinositol transfer protein (Pitpn), mRNA.
scl34300.16.8_59-S	-26.868	-1.8	Kars	NM_053092.1	Mus musculus lysyl-tRNA synthetase (Kars), mRNA.
scl023833.2_2-S	-26.7944	-1.73	Cd52	NM_013706	Mus musculus CD52 antigen (Cd52), mRNA.
scl0018458.1_6-S	-26.7722	-2.87	Pabpc1	NM_008774.2	Mus musculus poly A binding protein, cytoplasmic 1 (Pabpc1), mRNA.
scl41542.4_321-S	-26.7428	-1.43	Gm2a	NM_010299.2	Mus musculus GM2 ganglioside activator protein (Gm2a), mRNA.
scl48097.14_64-S	-26.5269	-13.23	1110020G09Rik	XM_127934.4	Mus musculus RIKEN cDNA 1110020G09 gene (1110020G09Rik), mRNA.
scl43929.4.1_16-S	-26.4334	-8.12	Mxd3	NM_016662.2	
scl47395.9.8_1-S	-26.3691	-1.43	1110064N10Rik	NM_026396.2	Mus musculus RIKEN cDNA 1110064N10 gene (1110064N10Rik), mRNA.
scl36472.2.242_20-S	-26.3647	-1.38	Gpx1	NM_008160.1	Mus musculus glutathione peroxidase 1 (Gpx1), mRNA.
scl41758.26.1_24-S	-26.3626	-1.85	Pnpt1	NM_027869.1	Mus musculus polyribonucleotide nucleotidyltransferase 1 (Pnpt1), mRNA.
scl24791.10_1-S	-26.2892	-1.4	Ddost	NM_007838.2	Mus musculus dolichyl-di-phosphooligosaccharide-protein glycotransferase (Ddost), mRNA.
scl53624.9_65-S	-26.2464	-6.62	Syap1	NM_025932.1	Mus musculus synapse associated protein 1 (Syap1), mRNA.
scl0004022.1_70-S	-26.2381	-1.4	Arpc1b	NM_023142.1	Mus musculus actin related protein 2/3 complex, subunit 1B (Arpc1b), mRNA.
scl0055946.2_3-S	-26.1844	-8.84	Ap3m1	NM_018829.2	Mus musculus adaptor-related protein complex 3, mu 1 subunit (Ap3m1), mRNA.

scl0002144.1_169-S	-26.1505	-2.58	0610031J06Rik	NM_020003.1	Mus musculus RIKEN cDNA 0610031J06 gene (0610031J06Rik), mRNA.
scl27616.6_187-S	-26.0997	-1.85	Dck	NM_007832.3	Mus musculus deoxycytidine kinase (Dck), mRNA.
scl012491.1_51-S	-25.9922	-2.24	Cd36	NM_007643.2	Mus musculus CD36 antigen (Cd36), mRNA.
scl50784.3.81_39-S	-25.97	-1.75	Ltb	NM_008518.1	Mus musculus lymphotoxin B (Ltb), mRNA.
scl25953.10_428-S	-25.9584	-2.87	Wbscr16	NM_033572.1	Mus musculus Williams-Beuren syndrome chromosome region 16 homolog (human) (Wbscr16), mRNA.
scl42750.13_363-S	-25.9437	-1.76	Tnfaip2	NM_009396.1	Mus musculus tumor necrosis factor, alpha-induced protein 2 (Tnfaip2), mRNA.
scl49241.6_310-S	-25.9025	-3.34	3110001H15Rik	NM_027355.1	Mus musculus RIKEN cDNA 3110001H15 gene (3110001H15Rik), mRNA.
scl38867.11_23-S	-25.861	-1.44	Sara1	NM_009120.1	Mus musculus SAR1a gene homolog 1 (S. cerevisiae) (Sara1), mRNA.
scl0192170.3_20-S	-25.8327	-1.42	Ddx48	NM_138669.1	Mus musculus DEAD (Asp-Glu-Ala-Asp) box polypeptide 48 (Ddx48), mRNA.
scl39350.4.1_193-S	-25.7567	-3.53	Igsf7	XM_126696.1	Mus musculus immunoglobulin superfamily, member 7 (Igsf7), mRNA.
scl0022185.2_118-S	-25.7391	-1.51	U2af2	NM_133671.1	Mus musculus U2 small nuclear ribonucleoprotein auxiliary factor (U2AF) 2 (U2af2), mRNA.
scl41083.21_226-S	-25.7273	-6.56	Mtmr4	NM_133215.1	Mus musculus myotubularin related protein 4 (Mtmr4), mRNA.
scl21793.3_27-S	-25.6398	-2.01	Bcl9	XM_358351.1	Mus musculus B-cell CLL/lymphoma 9 (Bcl9), mRNA.
scl072195.1_127-S	-25.6109	-3.84	2610524B01Rik	XM_355594	Mus musculus RIKEN cDNA 2610524B01 gene (2610524B01Rik), mRNA.
scl000538.1_65-S	-25.6082	-1.56	Banf1	NM_011793.2	Mus musculus barrier to autointegration factor 1 (Banf1), mRNA.
scl068544.3_98-S	-25.5596	-1.36	2310036O22Rik	NM_026760.1	
scl067219.1_81-S	-25.5307	-1.85	2810046C01Rik	NM_026039.2	Mus musculus RIKEN cDNA 2810046C01 gene (2810046C01Rik), mRNA.
scl53554.5.129_30-S	-25.495	-1.8	Bad	NM_007522.1	Mus musculus Bcl-associated death promoter (Bad), mRNA.
scl0001292.1_30-S	-25.4033	-1.52	Snrp116	NM_011431.2	Mus musculus U5 small nuclear ribonucleoprotein (Snrp116), mRNA.
scl050931.2_70-S	-25.3978	-2.94	Il27ra	NM_016671.2	Mus musculus interleukin 27 receptor, alpha (Il27ra), mRNA.
scl35812.9_1-S	-25.3903	-11.14	Tm4sf8	NM_019793.2	Mus musculus transmembrane 4 superfamily member 8 (Tm4sf8), mRNA.
scl0001554.1_4-S	-25.3867	-1.77	Tk1	NM_009387	Mus musculus thymidine kinase 1 (Tk1), mRNA.
scl36473.24.9_6-S	-25.294	-1.44	Usp4	NM_011678.1	Mus musculus ubiquitin specific protease 4 (proto-oncogene) (Usp4), mRNA.
scl0023881.1_86-S	-25.2746	-1.31	E430034L04Rik	NM_011816.2	Mus musculus RIKEN cDNA E430034L04 gene (E430034L04Rik), mRNA.
scl25783.3.1_76-S	-25.2484	-1.71	Zfp99	NM_023322.1	Mus musculus zinc finger protein 99 (Zfp99), mRNA.
scl0056284.1_41-S	-25.2358	-2.09	Mrpl19	NM_026490.1	Mus musculus mitochondrial ribosomal protein L19 (Mrpl19), mRNA.
scl011545.25_15-S	-25.2334	-1.42	Adprt1	NM_007415.2	Mus musculus ADP-ribosyltransferase (NAD+; poly (ADP-ribose) polymerase) 1 (Adprt1), mRNA.
scl22983.9.9_77-S	-25.1694	-2.31	Scamp3	NM_011886.1	Mus musculus secretory carrier membrane protein 3 (Scamp3), mRNA.
scl16516.6.1_29-S	-25.1498	-1.87	Pde6d	NM_008801.2	Mus musculus phosphodiesterase 6D, cGMP-specific, rod, delta (Pde6d), mRNA.
scl0026900.1_130-S	-25.091	-4.23	Ddx3y	NM_012008.1	Mus musculus DEAD (Asp-Glu-Ala-Asp) box polypeptide 3, Y-linked (Ddx3y), mRNA.
scl44726.4.1_4-S	-25.0436	-4.91	Caml	NM_007596.1	Mus musculus calcium modulating ligand (Caml), mRNA.
scl018293.8_37-S	-24.9078	-1.36	Ogdh	NM_010956.1	Mus musculus oxoglutarate dehydrogenase (lipoamide) (Ogdh), mRNA.
scl50760.8.1_58-S	-24.8615	-3.16	Dhx16	NM_026987.1	Mus musculus DEAH (Asp-Glu-Ala-His) box polypeptide 16 (Dhx16), mRNA.
scl27971.8.1_8-S	-24.8523	-1.8	Emilin1	NM_133918.1	Mus musculus elastin microfibril interfacer 1 (Emilin1), mRNA.
scl0076789.1_86-S	-24.8168	-1.92	2410129H14Rik	NM_175245.2	Mus musculus RIKEN cDNA 2410129H14 gene (2410129H14Rik), mRNA.
scl012521.1_26-S	-24.7693	-1.28	Kai1	NM_007656.1	Mus musculus kangai 1 (suppression of tumorigenicity 6, prostate) (Kai1), mRNA.
scl0066494.2_134-S	-24.7585	-1.44	2610524G07Rik	NM_025596.4	Mus musculus RIKEN cDNA 2610524G07 gene (2610524G07Rik), mRNA.
scl23903.13_130-S	-24.7469	-1.46	Ermap	NM_013848.1	Mus musculus erythroblast membrane-associated protein (Ermap), mRNA.
scl0056347.2_235-S	-24.6361	-1.47	Eif3s8	NM_019646.1	Mus musculus eukaryotic translation initiation factor 3, subunit 8 (Eif3s8), mRNA.
scl40889.7.141_9-S	-24.6015	-1.74	D11Wsu68e	NM_026776.3	Mus musculus DNA segment, Chr 11, Wayne State University 68, expressed (D11Wsu68e), mRNA.
scl0068080.2_0-S	-24.3212	-3.8	D5Ertd708e	NM_024216.1	
scl21820.14.1_60-S	-24.2886	-8.96	Vps45	NM_013841.1	Mus musculus vacuolar protein sorting 45 (yeast) (Vps45), mRNA.
scl0240514.1_161-S	-24.278	-2.22	AI842788	NM_198616.1	
scl066916.1_19-S	-24.244	-4.23	Ndufb7	NM_025843.1	Mus musculus NADH dehydrogenase (ubiquinone) 1 beta subcomplex, 7 (Ndufb7), mRNA.
scl017527.1_149-S	-24.2335	-2.77	Mpv17	NM_008622.1	Mus musculus Mpv17 transgene, kidney disease mutant (Mpv17), mRNA.
scl0015598.1_13-S	-24.1856	-1.55	Iap	NM_010490	Mus musculus intracisternal A particles (Iap), mRNA.
scl36846.9.2_23-S	-24.1819	-1.74	Anp32a	NM_009672.2	Mus musculus acidic (leucine-rich) nuclear phosphoprotein 32 family, member A (Anp32a), mRNA.
scl47032.21.1_6-S	-24.1174	-2.17	Recql4	NM_058214.1	Mus musculus RecQ protein-like 4 (Recql4), mRNA.
scl48261.13.240_30-S	-24.0912	-1.27	Cct8	NM_009840.2	Mus musculus chaperonin subunit 8 (theta) (Cct8), mRNA.
scl020701.2_3-S	-24.0819	-1.47	Serpina1b	NM_009244.2	Mus musculus serine (or cysteine) proteinase inhibitor, clade A, member 1b (Serpina1b), mRNA.
scl24926.6.1_18-S	-24.0309	-2.04	Ak2	NM_016895.2	Mus musculus adenylate kinase 2 (Ak2), mRNA.
scl0027374.2_29-S	-23.9055	-1.75	Skb1	NM_013768.1	Mus musculus SKB1 homolog (S. pombe) (Skb1), mRNA.
scl0014980.1_39-S	-23.8868	-1.47	H2-L		
scl0224703.1_41-S	-23.7603	-1.33	9530046H09Rik	NM_145486.2	Mus musculus RIKEN cDNA 9530046H09 gene (9530046H09Rik), mRNA.
scl0018484.1_248-S	-23.7461	-3.86	Pam	NM_013626.1	Mus musculus peptidylglycine alpha-amidating monooxygenase (Pam), mRNA.
GI_23592945-S	-23.7212	-1.38	Eef1a1	XM_134967.2	Mus musculus eukaryotic translation elongation factor 1 alpha 1 (Eef1a1), mRNA.

scl0002486.1_42-S	-23.6826	-8.56	Myg1	NM_021713.1	Mus musculus melanocyte prolifeating gene 1 (Myg1), mRNA.
scl00329958.1_84-S	-23.634	-1.52	E2f2	NM_183301.1	Mus musculus E2F transcription factor 2 (E2f2), mRNA.
scl52793.9.1_8-S	-23.6098	-3.17	Unc93b	NM_019449.1	Mus musculus unc-93 homolog B (C. elegans) (Unc93b), mRNA.
scl069072.8_1-S	-23.5618	-1.44	Ebna1bp2	NM_026932.2	Mus musculus EBNA1 binding protein 2 (Ebna1bp2), mRNA.
scl000251.1_1-S	-23.5004	-4.87	Mrpl48	NM_026971.2	Mus musculus mitochondrial ribosomal protein L48 (Mrpl48), transcript variant 2, mRNA.
scl35096.10_154-S	-23.4872	-1.52	Ankrd10	NM_133971.1	Mus musculus ankyrin repeat domain 10 (Ankrd10), mRNA.
scl0002081.1_46-S	-23.4502	-4.84	2500003M10Rik	NM_023215.3	Mus musculus RIKEN cDNA 2500003M10 gene (2500003M10Rik), mRNA.
scl29628.36_98-S	-23.4079	-4.75	Fancd2	XM_132796.4	Mus musculus Fanconi anemia, complementation group D2 (Fancd2), mRNA.
scl33713.6.1_0-S	-23.3895	-1.56	Lsm4	NM_015816.1	Mus musculus LSM4 homolog, U6 small nuclear RNA associated (S. cerevisiae) (Lsm4), mRNA.
scl54227.4_188-S	-23.3761	-2.59	Tmem32	NM_146234.2	
scl0016423.1_78-S	-23.3587	-1.28	Cd47	NM_010581.3	Mus musculus CD47 antigen (Rh-related antigen, integrin-associated signal transducer) (Cd47), mRNA.
scl37797.29_0-S	-23.3151	-3.57	Col6a2	NM_146007.1	Mus musculus procollagen, type VI, alpha 2 (Col6a2), mRNA.
scl013690.1_50-S	-23.2848	-1.39	Eif4g2	NM_013507.2	Mus musculus eukaryotic translation initiation factor 4, gamma 2 (Eif4g2), mRNA.
scl38285.22_33-S	-23.2782	-8.78	Stat2	NM_019963.1	Mus musculus signal transducer and activator of transcription 2 (Stat2), mRNA.
scl0319168.1_1-S	-23.167	-1.76	Hist1h2ah	NM_175659.1	Mus musculus histone 1, H2ah (Hist1h2ah), mRNA.
scl41340.2.1_55-S	-23.1398	-2.41	1110030J09Rik	NM_025397.2	Mus musculus RIKEN cDNA 1110030J09 gene (1110030J09Rik), mRNA.
scl0054645.1_120-S	-23.1095	-1.59	DXImx47e	NM_207670.1	Mus musculus DNA segment, Chr X, Immunex 47, expressed (DXImx47e), mRNA.
scl29514.8.22_6-S	-23.0589	-1.48	Mlf2	NM_145385.1	Mus musculus myeloid leukemia factor 2 (Mlf2), mRNA.
scl51350.23.1_4-S	-23.0081	-1.73	Fbxo38	NM_134136.1	
scl0001713.1_7-S	-22.9974	-1.48	Trim10	NM_011280.1	Mus musculus tripartite motif protein 10 (Trim10), mRNA.
scl0016179.2_78-S	-22.969	-2.52	Irak1	NM_008363.1	Mus musculus interleukin-1 receptor-associated kinase 1 (Irak1), mRNA.
scl0001607.1_37-S	-22.8989	-1.51	2810410M20Rik	NM_024428	Mus musculus RIKEN cDNA 2810410M20 gene (2810410M20Rik), mRNA.
scl0020135.2_120-S	-22.8985	-1.69	Rrm2	NM_009104.1	Mus musculus ribonucleotide reductase M2 (Rrm2), mRNA.
scl45489.6.1_24-S	-22.8805	-10.72	2510005D08Rik	NM_026526.1	Mus musculus RIKEN cDNA 2510005D08 gene (2510005D08Rik), mRNA.
scl0012443.2_42-S	-22.8493	-3.22	Ccnd1	NM_007631.1	Mus musculus cyclin D1 (Ccnd1), mRNA.
scl016541.8_1-S	-22.8083	-1.47	Napsa	NM_008437.1	
scl0003366.1_7-S	-22.7207	-2.57	Nusap1	NM_133851.1	Mus musculus nucleolar and spindle associated protein 1 (Nusap1), mRNA.
scl066923.1_197-S	-22.7187	-1.52	2610016F04Rik	NM_025847.1	Mus musculus RIKEN cDNA 2610016F04 gene (2610016F04Rik), mRNA.
scl051792.12_26-S	-22.6802	-1.55	Ppp2r1a	NM_016891.2	Mus musculus protein phosphatase 2 (formerly 2A), regulatory subunit A (PR 65), alpha isoform (Ppp2r1a), mRNA.
scl34187.26.1_95-S	-22.6624	-6.21	Nup133	NM_172288.1	Mus musculus nucleoporin 133 (Nup133), mRNA.
scl0003171.1_33-S	-22.6166	-1.41	Psmb7	NM_011187.1	Mus musculus proteasome (prosome, macropain) subunit, beta type 7 (Psmb7), mRNA.
scl0001042.1_14-S	-22.614	-1.83	Mrps25	NM_025578.2	Mus musculus mitochondrial ribosomal protein S25 (Mrps25), mRNA.
scl38167.5_407-S	-22.5588	-2.09	3110003A17Rik	XM_125510.2	Mus musculus RIKEN cDNA 3110003A17 gene (3110003A17Rik), mRNA.
scl017330.5_48-S	-22.5523	-1.4	Minpp1	NM_010799.1	Mus musculus multiple inositol polyphosphate histidine phosphatase 1 (Minpp1), mRNA.
scl34674.6.1_40-S	-22.495	-1.32	Plvap	NM_032398.1	Mus musculus plasmalemma vesicle associated protein (Plvap), mRNA.
scl028019.8_226-S	-22.4636	-8.78	Ing4	NM_133345.1	Mus musculus inhibitor of growth family, member 4 (Ing4), mRNA.
scl072201.1_290-S	-22.4359	-1.43	2600013N14Rik	NM_152812.1	Mus musculus RIKEN cDNA 2600013N14 gene (2600013N14Rik), mRNA.
scl40113.4_264-S	-22.4218	-1.77	Akap10	NM_019921.2	Mus musculus A kinase (PRKA) anchor protein 10 (Akap10), mRNA.
scl32275.19.1_102-S	-22.4077	-1.66	Rrm1	NM_009103	Mus musculus ribonucleotide reductase M1 (Rrm1), mRNA.
scl50703.12.1_8-S	-22.4025	-1.72	Pla2g7	NM_013737.2	Mus musculus phospholipase A2, group VII (platelet-activating factor acetylhydrolase, plasma) (Pla2g7), mRNA.
scl55020.28_14-S	-22.3748	-1.32	Ube1x	NM_009457.2	Mus musculus ubiquitin-activating enzyme E1, Chr X (Ube1x), mRNA.
scl37474.12.36_46-S	-22.3704	-1.56	Cct2	NM_007636.1	Mus musculus chaperonin subunit 2 (beta) (Cct2), mRNA.
scl0003453.1_49-S	-22.3214	-6.95	Snx19	NM_028874.1	Mus musculus sorting nexin 19 (Snx19), mRNA.
scl31959.7.1_44-S	-22.2861	-1.3	Bccip	NM_025392.1	Mus musculus BRCA2 and CDKN1A interacting protein (Bccip), mRNA.
scl0066142.1_255-S	-22.2663	-1.56	Cox7b	NM_025379.1	Mus musculus cytochrome c oxidase subunit VIIb (Cox7b), mRNA.
scl0056304.1_278-S	-22.2169	-1.89	LOC56304	NM_019633.1	Mus musculus recombinant antineuraminidase single chain Ig VH and VL domains (LOC56304), mRNA.
scl0020842.1_84-S	-22.1982	-1.53	Stag1	NM_009282.2	Mus musculus stromal antigen 1 (Stag1), mRNA.
scl0015531.1_129-S	-22.1813	-1.82	Ndst1	NM_008306.2	Mus musculus N-deacetylase/N-sulfotransferase (heparan glucosaminyl) 1 (Ndst1), mRNA.
scl0070885.2_95-S	-22.1363	-1.54	4921521J11Rik	NM_027590.1	Mus musculus RIKEN cDNA 4921521J11 gene (4921521J11Rik), mRNA.
scl0004155.1_21-S	-22.1201	-3.53	Hip2	NM_016786.2	Mus musculus huntingtin interacting protein 2 (Hip2), mRNA.
scl093684.5_156-S	-22.0988	-1.52	39340	NM_053102.1	Mus musculus selenoprotein (Sep15), mRNA.
scl0014105.1_218-S	-22.0521	-1.55	Fusip1	NM_010178.1	Mus musculus FUS interacting protein (serine-arginine rich) 1 (Fusip1), mRNA.
scl067530.3_0-S	-21.9942	-1.4	Uqcrb	NM_026219.1	Mus musculus ubiquinol-cytochrome c reductase binding protein (Uqcrb), mRNA.
scl0002832.1_59-S	-21.9882	-2.18	Ptp4a2	NM_008974.2	Mus musculus protein tyrosine phosphatase 4a2 (Ptp4a2), mRNA.
scl51923.8.5_28-S	-21.9679	-2.02	Lmnb1	NM_010721.1	Mus musculus lamin B1 (Lmnb1), mRNA.
scl019684.7_10-S	-21.9612	-1.48	Rdx	NM_009041	Mus musculus radixin (Rdx), mRNA.

scl19292.6_70-S	-21.9317	-1.33	0610033I05Rik	NM_030243.2	Mus musculus RIKEN cDNA 0610033I05 gene (0610033I05Rik), mRNA.
scl0003917.1_104-S	-21.931	-1.32	Nap1l1	NM_015781.2	Mus musculus nucleosome assembly protein 1-like 1 (Nap1l1), mRNA.
scl51339.12_2-S	-21.9302	-1.31	Fech	NM_007998.3	Mus musculus ferrochelatase (Fech), mRNA.
scl39022.13.1_2-S	-21.923	-1.32	Hdac2	NM_008229.1	Mus musculus histone deacetylase 2 (Hdac2), mRNA.
scl36290.7_92-S	-21.9027	-1.44	Limd1	NM_013860.1	Mus musculus LIM domains containing 1 (Limd1), mRNA.
scl46105.6.1_21-S	-21.8991	-1.6	Vdrip	NM_026119.2	Mus musculus vitamin D receptor interacting protein (Vdrip), mRNA.
scl50915.17_55-S	-21.8509	-1.31	Mapk14	NM_011951	Mus musculus mitogen activated protein kinase 14 (Mapk14), mRNA.
scl0004148.1_17-S	-21.8079	-1.49	Lias	NM_024471.2	Mus musculus lipoic acid synthetase (Lias), mRNA.
scl33261.5_41-S	-21.8052	-1.41	Hsbp1	NM_024219.1	Mus musculus heat shock factor binding protein 1 (Hsbp1), mRNA.
scl54976.5.1_9-S	-21.7905	-1.41	Mcts1	NM_026902.1	Mus musculus malignant T cell amplified sequence 1 (Mcts1), mRNA.
scl0067239.2_94-S	-21.7851	-1.81	Bxdc1	NM_023323.2	Mus musculus brix domain containing 1 (Bxdc1), mRNA.
scl50638.6.1_30-S	-21.764	-1.77	Treml4	NM_172623.1	
scl0014423.1_138-S	-21.7609	-1.32	Galnt1	NM_013814.2	Mus musculus UDP-N-acetyl-alpha-D-galactosamine:polypeptide N-acetylgalactosaminyltransferase 1 (Galnt1), mRNA.
scl24426.8_231-S	-21.7272	-1.42	Wdr40a	NM_026893.2	
scl0072133.2_35-S	-21.7259	-8.21	2610009I02Rik	NM_028115.2	Mus musculus RIKEN cDNA 2610009I02 gene (2610009I02Rik), mRNA.
scl28407.7.1_0-S	-21.7139	-2.06	LOC213233	NM_145391.1	Mus musculus similar to hypothetical protein FLJ10143 (LOC213233), mRNA.
scl00229227.1_231-S	-21.6709	-3.55	D630029K19Rik	NM_172680.2	Mus musculus RIKEN cDNA D630029K19 gene (D630029K19Rik), mRNA.
scl00394432.2_137-S	-21.6133	-2.12	Ugt1a10	NM_201642	
scl00229541.2_280-S	-21.5726	-5.45	BC026996	NM_201407.1	Mus musculus cDNA sequence BC026996 (BC026996), mRNA.
scl050917.1_181-S	-21.4537	-7.06	Galns	NM_016722	Mus musculus galactosamine (N-acetyl)-6-sulfate sulfatase (Galns), mRNA.
scl00117198.2_233-S	-21.4307	-2.57	Ivns1abp	NM_028582.2	Mus musculus influenza virus NS1A binding protein (Ivns1abp), mRNA.
scl0004120.1_356-S	-21.394	-1.95	Orc5l	NM_011959.1	Mus musculus origin recognition complex, subunit 5-like (S. cerevisiae) (Orc5l), mRNA.
scl00231327.1_147-S	-21.3731	-1.73	Ppat	NM_172146.1	Mus musculus phosphoribosyl pyrophosphate amidotransferase (Ppat), mRNA.
scl25228.16_571-S	-21.3347	-8.46	A430091O22Rik	NM_183024.1	Mus musculus RIKEN cDNA A430091O22 gene (A430091O22Rik), mRNA.
scl0002780.1_2-S	-21.3272	-1.89	Exosc10	NM_016699.1	
scl0020438.2_182-S	-21.2947	-2.28	Siah1b	NM_009173.1	Mus musculus seven in absentia 1B (Siah1b), mRNA.
scl0002777.1_339-S	-21.2524	-1.48	Ak2	NM_016895.2	Mus musculus adenylate kinase 2 (Ak2), mRNA.
scl0071078.2_199-S	-21.2124	-6.01	Adam30	NM_027665.1	Mus musculus a disintegrin and metalloproteinase domain 30 (Adam30), mRNA.
scl52691.13_82-S	-21.2092	-1.54	Psat1	XM_129211.2	Mus musculus phosphoserine aminotransferase 1 (Psat1), mRNA.
scl17725.6_585-S	-21.2065	-1.37	Itm2c	NM_022417.1	Mus musculus integral membrane protein 2C (Itm2c), mRNA.
scl40751.1_9-S	-21.1835	-1.37	Rpl38	NM_023372.1	Mus musculus ribosomal protein L38 (Rpl38), mRNA.
scl066496.2_57-S	-21.176	-8.88	2700038C09Rik	NM_025598.1	Mus musculus RIKEN cDNA 2700038C09 gene (2700038C09Rik), mRNA.
scl0001589.1_43-S	-21.1603	-6.22	AI415282	NM_134021.1	Mus musculus expressed sequence AI415282 (AI415282), mRNA.
scl49610.9.216_11-S	-21.0517	-1.68	Fez2	NM_199448.1	Mus musculus fasciculation and elongation protein zeta 2 (zygin II) (Fez2), mRNA.
scl24617.16.29_1-S	-21.0316	-2.65	Cdc2l2	NM_007661.2	
scl39991.5_43-S	-21.0184	-7.56	Spag7	NM_172561	Mus musculus sperm associated antigen 7 (Spag7), mRNA.
scl0003429.1_37-S	-20.9623	-1.49	Hmbs	NM_013551	Mus musculus hydroxymethylbilane synthase (Hmbs), mRNA.
scl056378.6_20-S	-20.9435	-1.37	Arpc3	NM_019824.2	Mus musculus actin related protein 2/3 complex, subunit 3 (Arpc3), mRNA.
scl30825.22_142-S	-20.8977	-1.43	Rab6ip1	NM_021494.1	Mus musculus Rab6 interacting protein 1 (Rab6ip1), mRNA.
scl42032.3_211-S	-20.8475	-3.33	Bag5	XM_127149.3	Mus musculus BCL2-associated athanogene 5 (Bag5), mRNA.
scl41463.6_233-S	-20.785	-4.96	Grap	NM_027817.1	Mus musculus GRB2-related adaptor protein (Grap), mRNA.
scl32695.9.13_0-S	-20.784	-5.68	Nosip	NM_025533.1	Mus musculus nitric oxide synthase interacting protein (Nosip), mRNA.
scl000319.1_7-S	-20.774	-9.15	Acin1	NM_019567.1	Mus musculus apoptotic chromatin condensation inducer 1 (Acin1), mRNA.
scl012417.4_32-S	-20.7504	-1.38	Cbx3	NM_007624	Mus musculus chromobox homolog 3 (Drosophila HP1 gamma) (Cbx3), mRNA.
scl068299.1_68-S	-20.7177	-6.45	3100002B05Rik	NM_026664.2	Mus musculus RIKEN cDNA 3100002B05 gene (3100002B05Rik), mRNA.
scl53398.18.1_7-S	-20.7111	-5.55	Ganab	NM_008060.1	
scl36121.3_28-S	-20.6917	-1.34	1110011K10Rik	NM_170777.3	Mus musculus RIKEN cDNA 1110011K10 gene (1110011K10Rik), mRNA.
scl0020658.1_193-S	-20.6127	-3.03	Son	NM_178880.3	Mus musculus Son cell proliferation protein (Son), transcript variant 1, mRNA.
scl18851.3_427-S	-20.5761	-5.59	6430601A21Rik	NM_175466.2	Mus musculus RIKEN cDNA 6430601A21 gene (6430601A21Rik), mRNA.
scl31638.15.1_106-S	-20.5659	-3.23	Pou2f2	NM_011138.1	Mus musculus POU domain, class 2, transcription factor 2 (Pou2f2), mRNA.
scl39287.25.1_2-S	-20.5623	-1.43	Tmc6	NM_145439.1	Mus musculus transmembrane channel-like gene family 6 (Tmc6), mRNA.
scl0052477.2_74-S	-20.5327	-1.54	D1Ertd396e	NM_021421.2	Mus musculus DNA segment, Chr 1, ERATO Doi 396, expressed (D1Ertd396e), mRNA.
scl33660.5_397-S	-20.5248	-1.54	Hmox1	NM_010442.1	Mus musculus heme oxygenase (decycling) 1 (Hmox1), mRNA.
scl023825.3_146-S	-20.4432	-1.39	Banf1	NM_011793.2	Mus musculus barrier to autointegration factor 1 (Banf1), mRNA.
scl000884.1_198-S	-20.3947	-6.16	Kist	NM_010633.3	Mus musculus kinase interacting with leukemia-associated gene (stathmin) (Kist), mRNA.
scl38461.7.1_1-S	-20.349	-5.14	Pawr	XM_125814.2	Mus musculus PRKC, apoptosis, WT1, regulator (Pawr), mRNA.

scl0003661.1_1612-S	-20.3185	-1.75	Nedd9	NM_017464.2	Mus musculus neural precursor cell expressed, developmentally down-regulated gene 9 (Nedd9), mRNA.
scl068089.7_12-S	-20.3061	-1.32	Arpc4	NM_026552	Mus musculus actin related protein 2/3 complex, subunit 4 (Arpc4), mRNA.
scl40921.6_29-S	-20.2773	-1.44	Igfbp4	NM_010517.2	Mus musculus insulin-like growth factor binding protein 4 (Igfbp4), mRNA.
scl0018173.2_296-S	-20.2249	-1.39	Slc11a1	NM_013612.1	Mus musculus solute carrier family 11 (proton-coupled divalent metal ion transporters), member 1 (Slc11a1), mRNA.
scl00216760.1_116-S	-20.1964	-1.65	Mfap3	NM_145426.1	
scl48911.16.3_18-S	-20.196	-2.39	Usp16	NM_024258.1	Mus musculus ubiquitin specific protease 16 (Usp16), mRNA.
scl0022214.2_142-S	-20.1429	-1.27	Ube2h	XM_133034.1	Mus musculus ubiquitin-conjugating enzyme E2H (Ube2h), mRNA.
scl000622.1_13-S	-20.0981	-1.76	Brd7	NM_012047.1	Mus musculus bromodomain containing 7 (Brd7), mRNA.
scl0266692.1_279-S	-20.0777	-1.57	Cpne1	NM_170590.1	Mus musculus copine I (Cpne1), transcript variant 1, mRNA.
scl46987.7.1_26-S	-20.0762	-1.29	Rac2	NM_009008.2	Mus musculus RAS-related C3 botulinum substrate 2 (Rac2), mRNA.
scl068377.10_23-S	-20.0647	-5.12	Acta2	NM_007392.2	Mus musculus RIKEN cDNA 0610041G09 gene (0610041G09Rik), mRNA.
scl00106840.2_67-S	-20.0451	-1.73	AA407659	NM_175352.2	Mus musculus expressed sequence AA407659 (AA407659), mRNA.
scl0002138.1_36-S	-20.034	-2.74	4833424P18Rik	NM_029017	Mus musculus RIKEN cDNA 4833424P18 gene (4833424P18Rik), mRNA.
scl000184.1_1-S	-20.0325	-5.32	Sec23ip	XM_133737.4	Mus musculus Sec23 interacting protein (Sec23ip), mRNA.
scl020541.2_143-S	-20.0294	-2.2	Slc8a1	NM_011406.1	Mus musculus solute carrier family 8 (sodium/calcium exchanger), member 1 (Slc8a1), mRNA.
scl23887.17.1_103-S	-20.0239	-1.56	Ctps	NM_016748.1	Mus musculus cytidine 5-triphosphate synthase (Ctps), mRNA.
scl51737.13.1_28-S	-20.0167	-1.24	Atp5a1	NM_007505.1	Mus musculus ATP synthase, H+ transporting, mitochondrial F1 complex, alpha subunit, isoform 1 (Atp5a1), mRNA.

TABLE A4

GENMAPPS AND GENE ONTOLOGIES SIGNIFICANTLY CHANGED BY THE ATHEROGENIC DIET IN SPLEENS

UP-REGULATED - GENMAPP

No.	MAPP Name	Number Changed	Number Measured	Number on MAPP	Percent Changed	Percent Present	Z Score	Permuted P Value
1	Mm_Apoptosis	13	83	83	15.6627	100.0000	3.5500	0.0030
2	Mm_TNF-alpha-NF-kB_NetPath_9	19	166	176	11.4458	94.3182	2.7880	0.0050
3	Mm_B_Cell_Receptor_NetPath_12	17	147	150	11.5646	98.0000	2.6780	0.0080

UP-REGULATED - GENE ONTOLOGY

No.	GO Name	GO Type	Number Changed	Number Measured	Number in GO	Percent Changed	Percent Present	Z Score	Permuted P Value
1	nucleosome assembly	P	14	66	128	21.2121	51.5625	5.5780	0.0000
2	organelle organization and biogenesis	P	79	842	1000	9.3824	84.2000	5.0190	0.0000
3	RNA processing	P	38	329	371	11.5502	88.6792	4.8190	0.0000
4	protein biosynthesis	P	58	588	801	9.8639	73.4082	4.6770	0.0000
5	cell organization and biogenesis	P	132	1659	1897	7.9566	87.4539	4.5670	0.0000
6	DNA packaging	P	27	215	291	12.5581	73.8832	4.5310	0.0000
7	cellular physiological process	P	553	8916	10403	6.2023	85.7061	4.3510	0.0000
8	protein metabolism	P	202	2842	3423	7.1077	83.0266	4.0660	0.0000
9	apoptotic program	P	10	56	61	17.8571	91.8033	4.0370	0.0000
10	chromosome organization and biogenesis (sensu Eukaryota)	P	28	251	332	11.1554	75.6024	3.9220	0.0000
11	metabolism	P	424	6705	7938	6.3236	84.4671	3.8000	0.0000
12	RNA splicing	P	18	144	167	12.5000	86.2276	3.6690	0.0010
13	nucleobase\, nucleoside\, nucleotide and nucleic acid metabolism	P	195	2803	3355	6.9568	83.5469	3.6440	0.0010
14	mitosis	P	16	136	146	11.7647	93.1507	3.1880	0.0010
15	pyrimidine deoxyribonucleotide catabolism	P	2	2	2	100.0000	100.0000	5.8400	0.0020
16	biosynthesis	P	89	1154	1428	7.7123	80.8123	3.3600	0.0020
17	protein transport	P	49	589	654	8.3192	90.0612	3.0090	0.0020
18	protein modification	P	106	1476	1665	7.1816	88.6487	2.9060	0.0020
19	imprinting	P	3	6	6	50.0000	100.0000	4.7610	0.0030
20	intra-Golgi vesicle-mediated transport	P	4	11	12	36.3636	91.6667	4.4700	0.0030
21	protein catabolism	P	20	190	215	10.5263	88.3721	3.0240	0.0040
22	valyl-tRNA aminoacylation	P	2	2	2	100.0000	100.0000	5.8400	0.0050
23	small GTPase mediated signal transduction	P	29	305	344	9.5082	88.6628	3.0610	0.0050
24	protein processing	P	9	64	72	14.0625	88.8889	2.9870	0.0050
25	endosome organization and biogenesis	P	3	7	8	42.8571	87.5000	4.3170	0.0060
26	mitotic chromosome condensation	P	4	14	16	28.5714	87.5000	3.7690	0.0060
27	fatty acid oxidation	P	6	32	32	18.7500	100.0000	3.2700	0.0060
28	ubiquitin-dependent protein catabolism	P	15	132	149	11.3636	88.5906	2.9380	0.0060
29	superoxide metabolism	P	5	21	22	23.8095	95.4545	3.6620	0.0070
30	ubiquitin cycle	P	37	437	511	8.4668	85.5186	2.7150	0.0070
31	ribosomal protein import into nucleus	P	2	3	3	66.6667	100.0000	4.6280	0.0090
32	ribosome biogenesis and assembly	P	11	86	105	12.7907	81.9048	2.9480	0.0090
33	CTD phosphatase activity	F	4	6	6	66.6667	100.0000	6.5460	0.0000
34	protein phosphatase type 2C activity	F	4	9	10	44.4444	90.0000	5.1030	0.0000
35	binding	F	567	9101	10565	6.2301	86.1429	4.6550	0.0000
36	protein binding	F	310	4715	5185	6.5748	90.9354	3.7660	0.0000
37	oxidoreductase activity\, acting on paired donors\, with oxidation of a pair of donors	F	4	9	10	44.4444	90.0000	5.1030	0.0010
38	inositol or phosphatidylinositol kinase activity	F	7	29	33	24.1379	87.8788	4.3820	0.0010
39	enzyme activator activity	F	20	180	199	11.1111	90.4523	3.2870	0.0010
40	translation release factor activity\, codon specific	F	2	3	3	66.6667	100.0000	4.6280	0.0020
41	translation initiation factor activity	F	10	66	86	15.1515	76.7442	3.4210	0.0020
42	RNA binding	F	41	466	548	8.7983	85.0365	3.1240	0.0020
43	myosin phosphatase activity	F	3	5	5	60.0000	100.0000	5.3240	0.0030
44	metalloendopeptidase inhibitor activity	F	3	7	7	42.8571	100.0000	4.3170	0.0030
45	nucleic acid binding	F	182	2709	3327	6.7183	81.4247	2.9670	0.0030
46	protein phosphatase type 2A activity	F	3	6	6	50.0000	100.0000	4.7610	0.0040
47	enzyme binding	F	19	179	198	10.6145	90.4040	2.9860	0.0040
48	valine-tRNA ligase activity	F	2	2	2	100.0000	100.0000	5.8400	0.0050
49	procollagen-lysine 5-dioxygenase activity	F	2	3	3	66.6667	100.0000	4.6280	0.0050
50	phosphatidylinositol-4\,5-bisphosphate 3-kinase activity	F	2	3	3	66.6667	100.0000	4.6280	0.0070
51	copper\, zinc superoxide dismutase activity	F	3	7	7	42.8571	100.0000	4.3170	0.0070
52	acetyl-CoA C-acyltransferase activity	F	2	3	3	66.6667	100.0000	4.6280	0.0080
53	1-phosphatidylinositol-4-phosphate 5-kinase activity	F	3	8	8	37.5000	100.0000	3.9520	0.0080
54	casein kinase I activity	F	2	3	3	66.6667	100.0000	4.6280	0.0090
55	intracellular	C	492	6933	8118	7.0965	85.4028	7.7860	0.0000
56	nucleosome	C	14	56	115	25.0000	48.6957	6.3780	0.0000
57	cytoplasm	C	266	3484	3969	7.6349	87.7803	6.1860	0.0000
58	protein complex	C	150	1750	2202	8.5714	79.4732	5.9050	0.0000
59	nucleus	C	239	3274	3798	7.2999	86.2033	4.9910	0.0000
60	mitochondrion	C	65	730	822	8.9041	88.8078	4.0750	0.0000
61	chromatin	C	18	140	217	12.8571	64.5161	3.8030	0.0000
62	nuclear lamina	C	4	9	9	44.4444	100.0000	5.1030	0.0020
63	dynactin complex	C	3	6	7	50.0000	85.7143	4.7610	0.0020
64	nuclear inner membrane	C	4	11	13	36.3636	84.6154	4.4700	0.0020
65	ribosome	C	21	176	345	11.9318	51.0145	3.7290	0.0030
66	chromosome	C	27	279	364	9.6774	76.6484	3.0500	0.0030
67	mitochondrial envelope	C	27	280	329	9.6429	85.1064	3.0300	0.0040
68	eukaryotic translation elongation factor 1 complex	C	3	6	6	50.0000	100.0000	4.7610	0.0050
69	ATP-binding cassette (ABC) transporter complex	C	3	8	8	37.5000	100.0000	3.9520	0.0070
70	nucleoplasm	C	31	343	376	9.0379	91.2234	2.8650	0.0070
71	endosome	C	12	96	104	12.5000	92.3077	2.9910	0.0090
72	ribonucleoprotein complex	C	32	355	550	9.0141	64.5455	2.8960	0.0090

DOWN-REGULATED - GENMAPP

No.	MAPP Name	Number Changed	Number Measured	Number on MAPP	Percent Changed	Percent Present	Z Score	Permuted P Value
1	Mm_Proteasome_Degradation	13	73	81	17.8082	90.1235	5.3850	0.0000
2	Mm_Porphyrin_and_chlorophyll_metabolism	5	15	58	33.3333	25.8621	5.2850	0.0000
3	Mm_mRNA_processing_binding_Reactome	37	426	551	8.6854	77.3140	4.1660	0.0000

4	Mm_Pyrimidine_metabolism		7	51	87	13.7255	58.6207	3.0960	0.0080

DOWN-REGULATED - GENE ONTOLOGY

No.	GO Name	GO Type	Number Changed	Number Measured	Number in GO	Percent Changed	Percent Present	Z Score	Permuted P Value
1	heme oxidation	P	2	2	2	100.0000	100.0000	7.7730	0.0000
2	metabolism	P	288	6706	7938	4.2947	84.4797	6.8700	0.0000
3	regulation of progression through cell cycle	P	34	375	410	9.0667	91.4634	6.5300	0.0000
4	mRNA metabolism	P	22	196	223	11.2245	87.8924	6.4180	0.0000
5	RNA processing	P	30	329	371	9.1185	88.6792	6.1610	0.0000
6	RNA splicing	P	17	144	167	11.8056	86.2276	5.8900	0.0000
7	mRNA processing	P	19	173	198	10.9827	87.3737	5.8440	0.0000
8	cell cycle	P	45	629	681	7.1542	92.3642	5.7490	0.0000
9	DNA replication	P	16	145	160	11.0345	90.6250	5.3800	0.0000
10	protein biosynthesis	P	41	588	801	6.9728	73.4082	5.2960	0.0000
11	actin cytoskeleton organization and biogenesis	P	17	163	171	10.4295	95.3216	5.2670	0.0000
12	actin polymerization and/or depolymerization	P	8	52	54	15.3846	96.2963	4.9960	0.0000
13	protein polymerization	P	7	43	50	16.2791	86.0000	4.8750	0.0000
14	DNA metabolism	P	37	544	682	6.8015	79.7654	4.8550	0.0000
15	nuclear mRNA splicing\, via spliceosome	P	13	118	135	11.0170	87.4074	4.8380	0.0000
16	nucleobase\, nucleoside\, nucleotide and nucleic acid metabolism	P	128	2804	3355	4.5649	83.5768	4.5470	0.0000
17	ubiquitin-dependent protein catabolism	P	13	132	149	9.8485	88.5906	4.3540	0.0000
18	protein catabolism	P	16	190	215	8.4211	88.3721	4.1100	0.0000
19	protein modification	P	73	1476	1665	4.9458	88.6487	4.0050	0.0000
20	cell death	P	35	603	650	5.8043	92.7692	3.7020	0.0000
21	response to biotic stimulus	P	45	879	969	5.1195	90.7121	3.3250	0.0000
22	oligosaccharide catabolism	P	2	2	2	100.0000	100.0000	7.7730	0.0010
23	pyridoxine biosynthesis	P	2	2	2	100.0000	100.0000	7.7730	0.0010
24	cyclin catabolism	P	2	3	3	66.6667	100.0000	6.2420	0.0010
25	antigen processing\, exogenous antigen via MHC class II	P	3	9	13	33.3333	69.2308	5.1340	0.0010
26	actin filament polymerization	P	5	25	27	20.0000	92.5926	4.7720	0.0010
27	cytoskeleton organization and biogenesis	P	25	385	431	6.4935	89.3272	3.7140	0.0010
28	protein folding	P	15	200	227	7.5000	88.1057	3.4730	0.0010
29	positive regulation of T cell differentiation	P	4	17	17	23.5294	100.0000	4.7610	0.0020
30	chromosome segregation	P	6	41	46	14.6342	89.1304	4.1610	0.0020
31	cell division	P	14	168	178	8.3333	94.3820	3.7970	0.0020
32	ubiquitin cycle	P	26	437	511	5.9497	85.5186	3.3080	0.0020
33	mRNA export from nucleus	P	5	26	32	19.2308	81.2500	4.6440	0.0030
34	DNA-dependent DNA replication	P	8	69	73	11.5942	94.5206	3.9670	0.0030
35	mitosis	P	12	136	146	8.8235	93.1507	3.7380	0.0030
36	apoptosis	P	32	572	617	5.5944	92.7066	3.3110	0.0030
37	purine base biosynthesis	P	2	4	4	50.0000	100.0000	5.3150	0.0040
38	deoxyribonucleotide metabolism	P	3	13	27	23.0769	48.1482	4.0700	0.0060
39	mitotic chromosome condensation	P	3	14	16	21.4286	87.5000	3.8740	0.0060
40	antigen presentation	P	6	52	63	11.5385	82.5397	3.4190	0.0060
41	defense response	P	40	828	915	4.8309	90.4918	2.7360	0.0060
42	ATP synthesis coupled proton transport	P	5	45	60	11.1111	75.0000	3.0160	0.0070
43	ribosome biogenesis	P	7	79	84	8.8608	94.0476	2.8620	0.0070
44	response to stress	P	47	1014	1107	4.6351	91.5989	2.6810	0.0070
45	mitochondrial electron transport\, NADH to ubiquinone	P	3	14	25	21.4286	56.0000	3.8740	0.0080
46	response to reactive oxygen species	P	3	16	16	18.7500	100.0000	3.5330	0.0080
47	actin filament organization	P	5	45	47	11.1111	95.7447	3.0160	0.0080
48	antigen presentation\, exogenous peptide antigen	P	2	5	6	40.0000	83.3333	4.6730	0.0090
49	germ cell migration	P	2	6	6	33.3333	100.0000	4.1910	0.0090
50	regulation of mitosis	P	5	42	45	11.9048	93.3333	3.2060	0.0090
51	heme oxygenase (decyclizing) activity	F	2	2	2	100.0000	100.0000	7.7730	0.0000
52	RNA binding	F	40	466	548	8.5837	85.0365	6.7010	0.0000
53	ribonucleoside-diphosphate reductase activity	F	2	3	3	66.6667	100.0000	6.2420	0.0000
54	nucleotide binding	F	95	1750	1959	5.4286	89.3313	5.6290	0.0000
55	protein binding	F	207	4716	5185	4.3893	90.9547	5.6050	0.0000
56	structural constituent of ribosome	F	18	183	354	9.8361	51.6949	5.1260	0.0000
57	unfolded protein binding	F	15	146	159	10.2740	91.8239	4.8750	0.0000
58	antigen binding	F	6	34	63	17.6471	53.9683	4.7870	0.0000
59	ATP binding	F	59	1180	1331	5.0000	88.6551	3.6520	0.0000
60	binding	F	326	9103	10565	3.5812	86.1619	3.3050	0.0000
61	NADH dehydrogenase (ubiquinone) activity	F	5	27	39	18.5185	69.2308	4.5230	0.0010
62	NADH dehydrogenase activity	F	5	31	43	16.1290	72.0930	4.0900	0.0020
63	3 iron\, 4 sulfur cluster binding	F	2	2	2	100.0000	100.0000	7.7730	0.0030
64	extracellular matrix constituent conferring elasticity	F	2	4	4	50.0000	100.0000	5.3150	0.0030
65	threonine endopeptidase activity	F	4	18	21	22.2222	85.7143	4.5840	0.0030
66	identical protein binding	F	16	223	237	7.1749	94.0928	3.3930	0.0030
67	hydrogen-transporting ATP synthase activity\, rotational mechanism	F	5	43	56	11.6279	76.7857	3.1410	0.0050
68	hydrogen-transporting ATPase activity\, rotational mechanism	F	5	45	58	11.1111	77.5862	3.0160	0.0050
69	structural molecule activity	F	32	632	893	5.0633	70.7727	2.7130	0.0050
70	intracellular calcium activated chloride channel activity	F	2	3	3	66.6667	100.0000	6.2420	0.0060
71	ligase activity	F	21	382	449	5.4974	85.0780	2.5790	0.0070
72	beta-N-acetylhexosaminidase activity	F	2	4	5	50.0000	80.0000	5.3150	0.0080
73	ATP-dependent helicase activity	F	9	98	109	9.1837	89.9083	3.3720	0.0080
74	methionyl aminopeptidase activity	F	2	5	5	40.0000	100.0000	4.6730	0.0090
75	pepsin A activity	F	2	7	9	28.5714	77.7778	3.8120	0.0090
76	ATPase activity	F	19	312	360	6.0897	86.6667	2.9250	0.0090
77	intracellular	C	325	6934	8118	4.6870	85.4151	9.6380	0.0000
78	cytoplasm	C	191	3484	3969	5.4822	87.7803	8.7380	0.0000
79	Arp2/3 protein complex	C	4	8	8	50.0000	100.0000	7.5170	0.0000
80	protein complex	C	106	1751	2202	6.0537	79.5186	7.2130	0.0000
81	ribonucleoprotein complex	C	33	355	550	9.2958	64.5455	6.5970	0.0000
82	proteasome complex (sensu Eukaryota)	C	8	37	42	21.6216	88.0952	6.3690	0.0000
83	mitochondrion	C	50	730	822	6.8493	88.8078	5.7360	0.0000
84	nucleus	C	149	3275	3798	4.5496	86.2296	4.9580	0.0000
85	spliceosome complex	C	9	71	78	12.6761	91.0256	4.5430	0.0000
86	ribosome	C	16	176	345	9.0909	51.0145	4.4610	0.0000

87	mitochondrial inner membrane	C	19	232	275	8.1897	84.3636	4.3460	0.0000
88	cytosol	C	23	344	383	6.6860	89.8172	3.7100	0.0000
89	cyclin-dependent protein kinase holoenzyme complex	C	3	7	7	42.8571	100.0000	5.9580	0.0020
90	proteasome core complex (sensu Eukaryota)	C	4	18	21	22.2222	85.7143	4.5840	0.0020
91	actin cytoskeleton	C	16	206	229	7.7670	89.9563	3.7450	0.0020
92	MHC class I protein complex	C	4	25	32	16.0000	78.1250	3.6360	0.0080
93	proton-transporting two-sector ATPase complex	C	5	47	61	10.6383	77.0492	2.8990	0.0080
94	nucleolus	C	10	133	147	7.5188	90.4762	2.8380	0.0090

TABLE A5					
GENES UP-REGULATED BY THE ATHEROGENIC DIET IN HEARTS					
TargetID	Diff_Score	Fold_Change	Symbol	Accession	Definition
scl013728.1_329-S	225.3751	2.31	Mark2	NM_007928.1	Mus musculus MAP/microtubule affinity-regulating kinase 2 (Mark2), mRNA.
scl00227682.2_166-S	206.0585	2.24	G430055L02Rik	NM_145520.2	
scl19105.6_353-S	203.7144	2.1	Ttn	XM_130312.3	Mus musculus titin (Ttn), mRNA.
scl23219.3_221-S	195.1325	2.3	Rab33b	NM_016858.1	Mus musculus RAB33B, member of RAS oncogene family (Rab33b), mRNA.
scl020320.8_193-S	180.409	2.11	Sdfr1	NM_009145.1	Mus musculus stromal cell derived factor receptor 1 (Sdfr1), mRNA.
scl000674.1_0-S	167.4774	3.47	Itgb1	NM_010578.1	Mus musculus integrin beta 1 (fibronectin receptor beta) (Itgb1), mRNA.
scl35003.22_120-S	166.5555	2.11	Adam9	NM_007404.1	Mus musculus a disintegrin and metalloproteinase domain 9 (meltrin gamma) (Adam9), mRNA.
scl0066885.2_329-S	158.1225	1.78	Acadsb	NM_025826.1	Mus musculus acyl-Coenzyme A dehydrogenase, short/branched chain (Acadsb), mRNA.
scl0326622.12_322-S	150.4993	1.92	Upf2	XM_140801.3	Mus musculus UPF2 regulator of nonsense transcripts homolog (yeast) (Upf2), mRNA.
scl38952.4.1_0-S	149.1629	2.2	Popdc3	NM_024286.1	Mus musculus popeye domain containing 3 (Popdc3), mRNA.
scl0019246.2_230-S	148.3342	2.4	Ptpn1	NM_011201.1	Mus musculus protein tyrosine phosphatase, non-receptor type 1 (Ptpn1), mRNA.
scl071955.1_257-S	145.442	2.11	2400003C14Rik	NM_028018.1	Mus musculus RIKEN cDNA 2400003C14 gene (2400003C14Rik), mRNA.
scl0002448.1_81-S	143.3259	1.77	Cpt1b	NM_009948.1	Mus musculus carnitine palmitoyltransferase 1b, muscle (Cpt1b), mRNA.
scl28579.2_420-S	142.4768	2.08	Fin14		Mus musculus fibroblast growth factor inducible 14 (Fin14), mRNA.
scl023849.4_8-S	141.4361	1.78	Copeb	NM_011803.1	Mus musculus core promoter element binding protein (Copeb), mRNA.
scl19523.39_64-S	141.3836	2.2	Notch1	NM_008714.2	Mus musculus Notch gene homolog 1 (Drosophila) (Notch1), mRNA.
scl37402.3_73-S	140.2336	1.75	Sas	NM_025982	Mus musculus sarcoma amplified sequence (Sas), mRNA.
scl21429.6_290-S	138.9363	1.76	Lmo4	NM_010723.2	Mus musculus LIM domain only 4 (Lmo4), mRNA.
scl067248.2_33-S	136.3396	1.67	Rpl39	NM_026055.1	Mus musculus ribosomal protein L39 (Rpl39), mRNA.
scl40333.9_365-S	132.7923	4.67	Mat2b	NM_134017.1	Mus musculus methionine adenosyltransferase II, beta (Mat2b), mRNA.
scl00320487.1_23-S	132.4043	2.59	D930036F22Rik	NM_177171.3	Mus musculus RIKEN cDNA D930036F22 gene (D930036F22Rik), mRNA.
scl0012848.2_316-S	132.2569	1.8	Cops2	NM_009939.1	Mus musculus COP9 (constitutive photomorphogenic) homolog, subunit 2 (Arabidopsis thaliana) (Cops2), mRNA.
scl0075613.2_301-S	126.1417	1.78	2610034E13Rik	NM_029365.1	Mus musculus RIKEN cDNA 2610034E13 gene (2610034E13Rik), mRNA.
scl52911.9_189-S	125.2484	1.79	Esrra	NM_007953.1	Mus musculus estrogen related receptor, alpha (Esrra), mRNA.
scl38222.21_67-S	122.9735	2.17	Sash1	NM_175155.3	
scl0026442.1_315-S	119.57	3.08	Psma5	NM_011967.1	Mus musculus proteasome (prosome, macropain) subunit, alpha type 5 (Psma5), mRNA.
scl39226.2.178_25-S	118.4697	1.72	1110012N22Rik	XM_126634.3	Mus musculus RIKEN cDNA 1110012N22 gene (1110012N22Rik), mRNA.
scl29852.1_47-S	116.9425	1.63	Mrpl53	NM_026744.1	Mus musculus mitochondrial ribosomal protein L53 (Mrpl53), mRNA.
scl45459.17_81-S	116.6657	1.67	Kpna3	NM_008466.2	Mus musculus karyopherin (importin) alpha 3 (Kpna3), mRNA.
scl25574.8.1_15-S	116.1775	2.37	Ube2j1	NM_019586.2	Mus musculus ubiquitin-conjugating enzyme E2, J1 (Ube2j1), mRNA.
scl0070231.1_327-S	115.9849	1.78	Gorasp2	NM_027352.2	Mus musculus golgi reassembly stacking protein 2 (Gorasp2), mRNA.
scl26735.47.1_66-S	112.1964	1.81	Ift172	NM_026298.4	Mus musculus intraflagellar transport 172 (Ift172), mRNA.
scl45408.20.1_29-S	112.1871	1.81	Ephx2	NM_007940.2	Mus musculus epoxide hydrolase 2, cytoplasmic (Ephx2), mRNA.
scl28480.7_231-S	111.8619	1.74	Adipor2	NM_197985.2	
scl0003758.1_1237-S	110.8698	1.61	Ubqln1	NM_026842.3	Mus musculus ubiquilin 1 (Ubqln1), transcript variant 1, mRNA.
scl24770.15_172-S	110.3379	1.76	Aldh4a1	NM_175438	Mus musculus aldehyde dehydrogenase 4 family, member A1 (Aldh4a1), mRNA.
scl27267.9.1_12-S	110.0567	2.3	Myl2	NM_010861	Mus musculus myosin, light polypeptide 2, regulatory, cardiac, slow (Myl2), mRNA.
scl0015530.1_184-S	108.2422	1.67	Hspg2	NM_008305.2	Mus musculus perlecan (heparan sulfate proteoglycan 2) (Hspg2), mRNA.
scl015204.23_217-S	107.9656	1.8	Herc2	NM_010418.1	Mus musculus hect (homologous to the E6-AP (UBE3A) carboxyl terminus) domain and RCC1 (CHC1)-like domain (RLD) 2 (Herc2), mRNA.
scl17431.17.1_113-S	107.3953	2.74	Tnnt2	NM_011619.1	Mus musculus troponin T2, cardiac (Tnnt2), mRNA.
scl42463.7.1_1-S	107.0418	2.51	1810011O16Rik	NM_025456.2	Mus musculus RIKEN cDNA 1810011O16 gene (1810011O16Rik), mRNA.
scl0069386.2_29-S	106.7067	1.8	Hist1h4h	NM_153173.1	Mus musculus histone 1, H4h (Hist1h4h), mRNA.
scl53453.19_390-S	106.5871	2.2	Adrbk1	NM_130863	Mus musculus adrenergic receptor kinase, beta 1 (Adrbk1), mRNA.
scl022190.1_154-S	105.8834	2.08	Ubc	XM_147315.1	Mus musculus ubiquitin C (Ubc), mRNA.
scl020818.1_29-S	103.5134	3.04	Srprb	NM_009275.2	Mus musculus signal recognition particle receptor, B subunit (Srprb), mRNA.
scl30402.11.1_13-S	102.3947	2.01	BC020002	NM_145374.1	Mus musculus cDNA sequence BC020002 (BC020002), mRNA.
scl40890.2_6-S	102.1184	1.56	Ramp2	NM_019444.1	Mus musculus receptor (calcitonin) activity modifying protein 2 (Ramp2), mRNA.
scl018132.31_78-S	101.1458	1.8	Notch4	NM_010929	Mus musculus Notch gene homolog 4 (Drosophila) (Notch4), mRNA.
scl24427.29_125-S	101.059	1.61	Ubap2	NM_026872.1	Mus musculus ubiquitin-associated protein 2 (Ubap2), mRNA.
scl0017859.1_306-S	100.7395	1.8	Mxi1	NM_010847.1	Mus musculus Max interacting protein 1 (Mxi1), mRNA.
scl17336.3.1_84-S	100.7229	1.61	2810025M15Rik	NM_027274.2	Mus musculus RIKEN cDNA 2810025M15 gene (2810025M15Rik), mRNA.
scl52119.18_599-S	100.0919	3.25	Jmjd1b	NM_029518.1	Mus musculus jumonji domain containing 1B (Jmjd1b), mRNA.
scl084652.1_47-S	98.7797	1.78	Drctnnb1a	NM_053090	Mus musculus down-regulated by Ctnnb1, a (Drctnnb1a), mRNA.

scl0076890.1_96-S	98.3278	2.06	0610016J10Rik	NM_133771.1	Mus musculus RIKEN cDNA 0610016J10 gene (0610016J10Rik), mRNA.
scl54140.7.1_25-S	97.5282	1.71	Dnase1l1	NM_027109.1	
scl27703.26_20-S	97.2142	1.67	Pdgfra	NM_011058.1	Mus musculus platelet derived growth factor receptor, alpha polypeptide (Pdgfra), mRNA.
scl019244.5_2-S	97.1468	1.52	Ptp4a2	NM_008974.2	Mus musculus protein tyrosine phosphatase 4a2 (Ptp4a2), mRNA.
scl0067949.2_13-S	96.6472	2.08	Mki67ip	NM_026472.2	Mus musculus Mki67 (FHA domain) interacting nucleolar phosphoprotein (Mki67ip), mRNA.
scl39478.12_629-S	96.4748	1.72	Plekhm1	NM_183034.1	Mus musculus pleckstrin homology domain containing, family M (with RUN domain) member 1 (Plekhm1), mRNA.
scl0022070.1_31-S	95.528	2.02	Tpt1	NM_009429	Mus musculus tumor protein, translationally-controlled 1 (Tpt1), mRNA.
scl069257.1_70-S	95.0704	1.88	Elf2	NM_023502.1	Mus musculus E74-like factor 2 (Elf2), mRNA.
scl46010.5_526-S	94.9227	1.6	Cln5	XM_127882.3	Mus musculus ceroid-lipofuscinosis, neuronal 5 (Cln5), mRNA.
scl00101476.1_284-S	94.8918	1.57	AA960558	NM_133942.1	Mus musculus expressed sequence AA960558 (AA960558), mRNA.
scl014897.1_29-S	94.3564	1.58	Trip12	NM_133975.2	
scl066576.4_1-S	94.0626	1.53	Uqcrh	NM_025641.2	
scl49765.36_30-S	93.8535	1.7	Ptprs	NM_011218.1	Mus musculus protein tyrosine phosphatase, receptor type, S (Ptprs), mRNA.
scl056433.4_275-S	93.2447	1.56	Vps29	NM_019780.1	Mus musculus vacuolar protein sorting 29 (S. pombe) (Vps29), mRNA.
scl40141.3_333-S	92.8065	1.57	5730466P16Rik	NM_173453.1	Mus musculus RIKEN cDNA 5730466P16 gene (5730466P16Rik), mRNA.
scl31534.6.1_6-S	92.0252	1.75	Ckap1	NM_025548.1	Mus musculus cytoskeleton-associated protein 1 (Ckap1), mRNA.
scl42444.40_226-S	91.3322	2.03	Garnl1	NM_019994.3	Mus musculus GTPase activating RANGAP domain-like 1 (Garnl1), mRNA.
scl21235.24_380-S	91.2097	1.58	BC026657	NM_029895.3	
scl24919.4.1_260-S	90.5221	2.06	Sync	NM_023485.2	Mus musculus syncoilin (Sync), mRNA.
scl20180.20_84-S	90.2175	1.73	Rin2	NM_028724.2	Mus musculus Ras and Rab interactor 2 (Rin2), mRNA.
scl17047.9.4_6-S	90.1592	1.72	BC013667	NM_172266.1	Mus musculus cDNA sequence BC013667 (BC013667), mRNA.
scl073122.1_292-S	90.1279	1.66	3110018K12Rik	XM_129857.4	Mus musculus RIKEN cDNA 3110018K12 gene (3110018K12Rik), mRNA.
scl18838.17_213-S	89.9051	1.78	Mrg1	NM_010825	Mus musculus myeloid ecotropic viral integration site-related gene 1 (Mrg1), mRNA.
scl018412.1_16-S	89.8835	1.61	Sqstm1	NM_011018.1	Mus musculus sequestosome 1 (Sqstm1), mRNA.
scl0072333.1_312-S	89.1298	1.55	2410003B16Rik	XM_204283.3	Mus musculus RIKEN cDNA 2410003B16 gene (2410003B16Rik), mRNA.
scl49695.8_112-S	88.1561	1.55	Vapa	NM_013933.2	Mus musculus vesicle-associated membrane protein, associated protein A (Vapa), mRNA.
scl42239.4_289-S	87.6963	1.59	Npc2	NM_023409.3	Mus musculus Niemann Pick type C2 (Npc2), mRNA.
scl022218.1_29-S	87.6662	1.49	Sumo1	NM_009460.1	
scl0020019.1_252-S	87.5783	1.89	Rpo1-4	NM_009088.2	Mus musculus RNA polymerase 1-4 (Rpo1-4), mRNA.
scl013424.9_0-S	87.194	1.91	Dnchc1	NM_030238.1	Mus musculus dynein, cytoplasmic, heavy chain 1 (Dnchc1), mRNA.
scl16460.33_2-S	87.1561	3.54	Hdlbp	NM_133808.2	Mus musculus high density lipoprotein (HDL) binding protein (Hdlbp), mRNA.
scl16146.28_80-S	86.6554	1.74	Lamc1	NM_010683	Mus musculus laminin, gamma 1 (Lamc1), mRNA.
scl32857.8.1_60-S	85.6905	1.96	Lgals4	NM_010706.1	Mus musculus lectin, galactose binding, soluble 4 (Lgals4), mRNA.
scl20687.14.1_105-S	85.3676	2.65	Slc43a3	NM_021398.1	Mus musculus solute carrier family 43, member 3 (Slc43a3), mRNA.
scl0232087.1_5-S	84.6148	1.99	Mat2a	NM_145569	Mus musculus methionine adenosyltransferase II, alpha (Mat2a), mRNA.
scl0017765.2_254-S	84.4411	2.15	Mtf2	NM_013827.1	Mus musculus metal response element binding transcription factor 2 (Mtf2), mRNA.
scl19394.13_183-S	84.2157	1.73	Ggta1	NM_010283.1	Mus musculus glycoprotein galactosyltransferase alpha 1, 3 (Ggta1), mRNA.
scl24880.7_98-S	84.1182	1.52	Sfrs4	NM_020587.1	Mus musculus splicing factor, arginine/serine-rich 4 (SRp75) (Sfrs4), mRNA.
scl21980.4.1_109-S	83.9432	1.75	1700021C14Rik	XM_355444.1	Mus musculus RIKEN cDNA 1700021C14 gene (1700021C14Rik), mRNA.
scl0003002.1_1949-S	83.687	3.1	Zfp106	NM_011743.1	Mus musculus zinc finger protein 106 (Zfp106), mRNA.
scl0012846.1_253-S	83.658	1.82	Comt	XM_147265.1	Mus musculus catechol-O-methyltransferase (Comt), mRNA.
scl069920.4_5-S	83.165	1.67	Polr2i	XM_133304.4	Mus musculus polymerase (RNA) II (DNA directed) polypeptide I (Polr2i), mRNA.
scl00224938.2_37-S	83.014	1.56	Pja2	NM_144859.1	Mus musculus praja 2, RING-H2 motif containing (Pja2), mRNA.
scl069178.1_5-S	82.6794	2.02	Snx5	NM_024225.2	Mus musculus sorting nexin 5 (Snx5), mRNA.
scl37112.9_269-S	82.6682	1.49	Esam1	NM_027102.1	Mus musculus endothelial cell-specific adhesion molecule (Esam1), mRNA.
scl074150.16_288-S	82.5657	1.82	Slc35f5	NM_028787.2	Mus musculus solute carrier family 35, member F5 (Slc35f5), mRNA.
scl067440.9_18-S	82.2604	2.29	Papd1	NM_026157.1	Mus musculus PAP associated domain containing 1 (Papd1), mRNA.
scl0072046.1_240-S	81.9215	2.98	2010005J08Rik	NM_178623.2	Mus musculus RIKEN cDNA 2010005J08 gene (2010005J08Rik), mRNA.
scl31626.10.1_112-S	81.6969	1.5	Bckdha	NM_007533.2	Mus musculus branched chain ketoacid dehydrogenase E1, alpha polypeptide (Bckdha), mRNA.
scl066958.2_91-S	81.1071	1.74	2310042M24Rik	NM_025868.1	Mus musculus RIKEN cDNA 2310042M24 gene (2310042M24Rik), mRNA.
scl29537.12_355-S	81.0835	1.76	Foxj2	NM_021899.2	Mus musculus forkhead box J2 (Foxj2), mRNA.
scl0076055.1_48-S	80.9698	4.59	Mgea5	NM_023799.2	Mus musculus meningioma expressed antigen 5 (hyaluronidase) (Mgea5), mRNA.
scl067414.12_0-S	80.4662	1.49	Mfn1	NM_024200.2	Mus musculus mitofusin 1 (Mfn1), mRNA.
scl00170791.1_106-S	79.6319	1.56	Rnpc2	NM_133242.1	Mus musculus RNA-binding region (RNP1, RRM) containing 2 (Rnpc2), mRNA.
scl30059.16.1_6-S	79.1378	1.98	Mpp6	NM_019939.1	Mus musculus membrane protein, palmitoylated 6 (MAGUK p55 subfamily member 6) (Mpp6), mRNA.
scl17443.8_550-S	79.0117	1.49	Arl10b	NM_026823.1	

scl068337.8_322-S	78.7831	1.53	Crip2	NM_024223.1	Mus musculus cysteine rich protein 2 (Crip2), mRNA.
scl54521.7.509_3-S	78.6403	2.1	Smpx	NM_025357.1	Mus musculus small muscle protein, X-linked (Smpx), mRNA.
scl0065111.1_63-S	78.5063	1.64	Dap3	NM_022994.2	Mus musculus death associated protein 3 (Dap3), mRNA.
scl013480.1_38-S	78.3417	3.25	Dpm1	NM_010072.2	Mus musculus dolichol-phosphate (beta-D) mannosyltransferase 1 (Dpm1), mRNA.
scl020826.1_68-S	77.7439	2.48	Ssfa1	XM_196564.2	Mus musculus sperm specific antigen 1 (Ssfa1), mRNA.
scl0020928.2_272-S	77.3593	1.55	Abcc9	NM_011511.1	Mus musculus ATP-binding cassette, sub-family C (CFTR/MRP), member 9 (Abcc9), transcript variant 1, mRNA.
scl17939.35.1_294-S	76.7563	1.64	Aox1	NM_009676.1	Mus musculus aldehyde oxidase 1 (Aox1), mRNA.
scl0073197.1_297-S	76.0097	1.58	D19Ertd703e	NM_029456.1	
scl22246.19.1_4-S	75.8665	1.85	Mccc1	NM_023644.2	Mus musculus methylcrotonoyl-Coenzyme A carboxylase 1 (alpha) (Mccc1), mRNA.
scl24819.10.1_28-S	75.5987	1.58	Tcea3	NM_011542	Mus musculus transcription elongation factor A (SII), 3 (Tcea3), mRNA.
scl35194.5_5-S	75.3312	1.56	Hig1	NM_019814.2	Mus musculus hypoxia induced gene 1 (Hig1), mRNA.
scl000011.1_73-S	74.6768	1.49	Strn3	NM_052973	Mus musculus striatin, calmodulin binding protein 3 (Strn3), mRNA.
scl23946.8.1_41-S	74.6415	1.44	Urod	NM_009478.1	Mus musculus uroporphyrinogen decarboxylase (Urod), mRNA.
scl0072278.1_319-S	74.1941	2.07	D9Ertd392e	NM_028181.1	
scl39652.23.1463_24-S	73.732	1.52	Kpnb1	NM_008379.2	Mus musculus karyopherin (importin) beta 1 (Kpnb1), mRNA.
scl51536.11_7-S	73.7081	1.75	Etf1	NM_144866.2	Mus musculus eukaryotic translation termination factor 1 (Etf1), mRNA.
scl0021761.2_320-S	73.5191	2.57	Morf4l1	NM_024431	Mus musculus mortality factor 4 like 1 (Morf4l1), mRNA.
scl019166.8_87-S	73.4733	1.66	Psma2	NM_008944.1	Mus musculus proteasome (prosome, macropain) subunit, alpha type 2 (Psma2), mRNA.
scl011958.2_29-S	73.1739	2.16	Atp5k	NM_007507	Mus musculus ATP synthase, H+ transporting, mitochondrial F1F0 complex, subunit e (Atp5k), mRNA.
scl54871.18_531-S	73.0805	1.86	Mtmr1	NM_016985.1	Mus musculus myotubularin related protein 1 (Mtmr1), mRNA.
scl54450.17.1_6-S	73.0591	1.66	DXImx40e	NM_138603.2	Mus musculus DNA segment, Chr X, Immunex 40, expressed (DXImx40e), mRNA.
scl22658.17.1_65-S	72.8446	6.12	Sec24d	NM_027135.2	Mus musculus SEC24 related gene family, member D (S. cerevisiae) (Sec24d), mRNA.
scl27109.17.1_35-S	72.728	1.76	Plod3	NM_011962.2	Mus musculus procollagen-lysine, 2-oxoglutarate 5-dioxygenase 3 (Plod3), mRNA.
scl067374.11_26-S	72.5981	1.49	Jam2	NM_023844.2	Mus musculus junction adhesion molecule 2 (Jam2), mRNA.
scl35560.5_28-S	72.3851	1.59	Cox7a2	NM_009945.2	Mus musculus cytochrome c oxidase, subunit VIIa 2 (Cox7a2), mRNA.
scl42464.2_123-S	72.2149	1.46	1110002B05Rik	NM_134054.1	Mus musculus RIKEN cDNA 1110002B05 gene (1110002B05Rik), mRNA.
scl25450.6_639-S	71.7949	1.72	5730528L13Rik	NM_028137.1	Mus musculus RIKEN cDNA 5730528L13 gene (5730528L13Rik), mRNA.
scl0064934.1_269-S	71.6239	2.55	Pes1	NM_022889.2	Mus musculus pescadillo homolog 1, containing BRCT domain (zebrafish) (Pes1), mRNA.
scl25834.12_533-S	71.6202	1.64	Snx8	NM_172277.1	Mus musculus sorting nexin 8 (Snx8), mRNA.
scl0012651.2_322-S	71.5604	1.55	Chkb	NM_007692.3	Mus musculus choline kinase beta (Chkb), mRNA.
scl30665.8.1_14-S	71.489	2.76	1110032O16Rik	XM_133813.4	Mus musculus RIKEN cDNA 1110032O16 gene (1110032O16Rik), mRNA.
scl0017977.2_208-S	71.1215	2.02	Ncoa1	NM_010881.1	Mus musculus nuclear receptor coactivator 1 (Ncoa1), mRNA.
scl0027407.2_308-S	70.9962	1.47	Abcf2	NM_013853.1	Mus musculus ATP-binding cassette, sub-family F (GCN20), member 2 (Abcf2), mRNA.
scl00230249.2_159-S	70.986	1.43	AI314180	NM_172381.2	Mus musculus expressed sequence AI314180 (AI314180), mRNA.
scl53474.8.1_75-S	70.8739	1.52	Ccs	NM_016892.2	Mus musculus copper chaperone for superoxide dismutase (Ccs), mRNA.
scl0027223.1_124-S	70.7521	1.69	Trp53bp1	NM_013735.2	Mus musculus transformation related protein 53 binding protein 1 (Trp53bp1), mRNA.
scl098193.14_109-S	70.69	1.9	Wdr42a	NM_153555.1	
scl027883.1_63-S	70.3744	1.72	D16H22S680E	XM_147173.1	Mus musculus DNA segment, Chr 16, human D22S680E, expressed (D16H22S680E), mRNA.
scl0110829.10_232-S	70.2414	2.52	Lims1	NM_026148.1	Mus musculus LIM and senescent cell antigen-like domains 1 (Lims1), mRNA.
scl018786.2_1-S	70.1267	1.44	Plaa	NM_172695.1	Mus musculus phospholipase A2, activating protein (Plaa), mRNA.
scl24399.13_176-S	70.1045	1.8	Gba2	NM_172692.1	Mus musculus glucosidase beta 2 (Gba2), mRNA.
scl32537.2.2118_88-S	69.7762	2.02	Rgma	NM_177740.2	Mus musculus RGM domain family, member A (Rgma), mRNA.
scl41579.7_169-S	69.6541	1.6	Ppp2ca	NM_019411.2	Mus musculus protein phosphatase 2a, catalytic subunit, alpha isoform (Ppp2ca), mRNA.
scl00229211.2_16-S	69.4127	2.27	Acad9	NM_172678.2	Mus musculus acyl-Coenzyme A dehydrogenase family, member 9 (Acad9), mRNA.
scl39445.1.46_133-S	69.4031	1.69	Tex2	XM_203370.3	Mus musculus testis expressed gene 2 (Tex2), mRNA.
scl000086.1_135-S	69.3765	1.53	AA959742	NM_133807.1	Mus musculus expressed sequence AA959742 (AA959742), mRNA.
scl0268470.1_0-S	69.3266	1.71	D11Moh35	NM_172300	Mus musculus DNA segment, Chr 11, KL Mohlke 35 (D11Moh35), mRNA.
scl0241431.7_330-S	69.3112	5.75	Cmya3	XM_141283.2	
scl014897.1_33-S	69.1069	1.57	Trip12	NM_133975.2	
GI_21070949-S	68.4962	1.91	Ubc	NM_019639.2	Mus musculus ubiquitin C (Ubc), mRNA.
scl068051.5_64-S	68.3951	1.54	Nutf2	NM_026532.2	Mus musculus nuclear transport factor 2 (Nutf2), mRNA.
scl24787.5_386-S	68.0804	1.51	0610009K11Rik	NM_026689.3	Mus musculus RIKEN cDNA 0610009K11 gene (0610009K11Rik), mRNA.
scl020170.2_257-S	67.804	1.91	Hps6	NM_176785.1	Mus musculus Hermansky-Pudlak syndrome 6 (Hps6), mRNA.
scl22078.18_184-S	67.7254	2.5	Kpna4	NM_008467.2	Mus musculus karyopherin (importin) alpha 4 (Kpna4), mRNA.
scl50763.13.1_199-S	67.7102	1.44	Flot1	NM_008027.1	Mus musculus flotillin 1 (Flot1), mRNA.
scl00218442.1_227-S	67.7027	2.45	A130038L21Rik	NM_172588.2	Mus musculus RIKEN cDNA A130038L21 gene (A130038L21Rik), mRNA.

scl018521.14_30-S	67.6242	1.4	Pcbp2	NM_011042.1	Mus musculus poly(rC) binding protein 2 (Pcbp2), mRNA.
scl014077.4_160-S	67.5145	2.17	Fabp3	NM_010174.1	Mus musculus fatty acid binding protein 3, muscle and heart (Fabp3), mRNA.
gi_21070949_ref_NM_01!	67.3619	2	Ubc	XM_147315.1	Mus musculus ubiquitin C (Ubc), mRNA.
scl32464.22.1_107-S	67.1591	1.67	Pde8a	NM_008803.1	Mus musculus phosphodiesterase 8A (Pde8a), mRNA.
scl39118.3_388-S	67.0302	3.09	Perp	NM_022032.1	Mus musculus PERP, TP53 apoptosis effector (Perp), mRNA.
scl0002881.1_47-S	66.8993	2.77	Pdcd8	NM_012019.2	Mus musculus programmed cell death 8 (Pdcd8), mRNA.
scl022335.1_61-S	66.5786	1.95	Vdac3	NM_011696.1	Mus musculus voltage-dependent anion channel 3 (Vdac3), mRNA.
scl39983.12.1_110-S	66.2556	1.5	Nup88	NM_172394.1	Mus musculus nucleoporin 88 (Nup88), mRNA.
scl012385.18_225-S	66.1646	1.4	Catna1	NM_009818.1	Mus musculus catenin alpha 1 (Catna1), mRNA.
scl44846.7_0-S	66.1242	2.07	Nol7	NM_023554.1	Mus musculus nucleolar protein 7 (Nol7), mRNA.
scl46983.8_85-S	66.0701	1.79	Mfng	NM_008595.1	Mus musculus manic fringe homolog (Drosophila) (Mfng), mRNA.
scl27730.23_48-S	65.8121	1.63	Atp10d	NM_153389.2	Mus musculus ATPase, Class V, type 10D (Atp10d), mRNA.
scl073024.7_20-S	65.7376	1.6	2900064A13Rik	NM_133749.1	Mus musculus RIKEN cDNA 2900064A13 gene (2900064A13Rik), mRNA.
scl0067916.1_299-S	65.612	1.41	Ppap2b	NM_080555.1	Mus musculus phosphatidic acid phosphatase type 2B (Ppap2b), mRNA.
scl0077480.1_264-S	65.4847	1.52	C330002I19Rik	XM_126866.5	
scl0003079.1_4-S	65.3952	1.49	Grb14	NM_016719.1	Mus musculus growth factor receptor bound protein 14 (Grb14), mRNA.
scl052690.1_86-S	65.3661	3.28	D12Ertd771e	NM_028262.1	Mus musculus DNA segment, Chr 12, ERATO Doi 771, expressed (D12Ertd771e), mRNA.
scl41578.9_407-S	65.3087	1.6	Skp1a	NM_011543.2	Mus musculus S-phase kinase-associated protein 1A (Skp1a), mRNA.
scl0003259.1_330-S	65.279	3.27	Sh2d3c	NM_013781.2	Mus musculus SH2 domain containing 3C (Sh2d3c), mRNA.
scl0239126.1_315-S	65.0925	1.53	9130217G22Rik	NM_183175.1	
scl32855.14.1_3-S	65.041	1.54	Hnrpl	NM_177301.3	Mus musculus heterogeneous nuclear ribonucleoprotein L (Hnrpl), mRNA.
scl070425.3_227-S	65.0279	1.61	Csnk1g3	NM_152809.1	Mus musculus casein kinase 1, gamma 3 (Csnk1g3), mRNA.
scl0067590.2_244-S	64.2646	1.71	4930521E07Rik	NM_026260.2	Mus musculus RIKEN cDNA 4930521E07 gene (4930521E07Rik), mRNA.
scl44775.16.1_15-S	64.0729	1.93	Secisbp2	XM_127336.2	
scl017749.3_11-S	63.9446	1.43	Polr2k	NM_023127	Mus musculus polymerase (RNA) II (DNA directed) polypeptide K (Polr2k), mRNA.
scl066073.8_269-S	63.8043	1.49	0610040B21Rik	NM_025334.2	Mus musculus RIKEN cDNA 0610040B21 gene (0610040B21Rik), mRNA.
scl0067096.2_233-S	63.7345	1.49	1810037K07Rik	NM_025962.1	Mus musculus RIKEN cDNA 1810037K07 gene (1810037K07Rik), mRNA.
scl46775.6_475-S	63.524	1.4	Asb8	NM_030121.2	Mus musculus ankyrin repeat and SOCS box-containing protein 8 (Asb8), mRNA.
scl26992.46_0-S	63.3344	1.8	AI481500	NM_133901.1	Mus musculus expressed sequence AI481500 (AI481500), mRNA.
scl022327.6_246-S	63.1732	1.46	Vbp1	NM_011692.1	Mus musculus von Hippel-Lindau binding protein 1 (Vbp1), mRNA.
scl38900.29.1_38-S	62.8757	2.15	Ranbp2	NM_011240.2	Mus musculus RAN binding protein 2 (Ranbp2), mRNA.
scl074569.1_282-S	62.7754	1.56	Ttc17	NM_183106.1	
gi_30794511_ref_NM_01	62.7326	1.65	Hmbs	NM_013551.1	Mus musculus hydroxymethylbilane synthase (Hmbs), mRNA.
scl39283.5.1_319-S	62.5259	1.66	1300017K07Rik	NM_027919.1	Mus musculus RIKEN cDNA 1300017K07 gene (1300017K07Rik), mRNA.
scl39318.8_348-S	62.502	1.84	Wbp2	NM_016852.1	Mus musculus WW domain binding protein 2 (Wbp2), mRNA.
scl0016956.1_234-S	62.443	1.37	Lpl	NM_008509.1	Mus musculus lipoprotein lipase (Lpl), mRNA.
scl49626.40.1_3-S	62.3802	2.5	Xdh	NM_011723	Mus musculus xanthine dehydrogenase (Xdh), mRNA.
scl37880.6.1_25-S	62.2145	1.51	1110056A04Rik	NM_182992.1	Mus musculus RIKEN cDNA 1110056A04 gene (1110056A04Rik), mRNA.
scl50686.4.1_41-S	62.2096	1.39	Mrpl14	NM_026732.2	
scl0109077.2_328-S	62.0219	1.79	1110055N21Rik	NM_176843	Mus musculus RIKEN cDNA 1110055N21 gene (1110055N21Rik), mRNA.
scl0012865.1_86-S	61.8452	1.39	Cox7a1	NM_009944.2	Mus musculus cytochrome c oxidase, subunit VIIa 1 (Cox7a1), mRNA.
scl49035.10.1_1-S	61.6738	9.42	Cblb	XM_358863.1	Mus musculus Casitas B-lineage lymphoma b (Cblb), mRNA.
scl022224.14_3-S	61.5248	1.63	Usp10	NM_009462.1	Mus musculus ubiquitin specific protease 10 (Usp10), mRNA.
scl0014897.1_159-S	61.4799	1.43	Trip12	NM_133975.2	
scl0075914.1_304-S	61.3949	1.55	Sec15l2	XM_355790.1	
scl056724.5_33-S	61.2746	1.66	1200020A08Rik	NM_019936.1	Mus musculus RIKEN cDNA 1200020A08 gene (1200020A08Rik), mRNA.
scl077634.9_5-S	61.0429	2.84	Snapc3	NM_029949.1	Mus musculus small nuclear RNA activating complex, polypeptide 3 (Snapc3), mRNA.
scl21601.6.1_41-S	60.9378	1.48	Palmd	NM_023245.2	Mus musculus palmdelphin (Palmd), mRNA.
scl50602.12.1_148-S	60.8811	1.57	Hdgfrp2	NM_008233.1	Mus musculus hepatoma-derived growth factor, related protein 2 (Hdgfrp2), mRNA.
scl29233.13_53-S	60.8653	1.61	Wasl	XM_133026.3	Mus musculus Wiskott-Aldrich syndrome-like (human) (Wasl), mRNA.
scl26162.14.72_6-S	60.7904	1.49	Rnf10	NM_016698	Mus musculus ring finger protein 10 (Rnf10), mRNA.
scl23222.4_222-S	60.6056	2.24	Ccrn4l	NM_009834.1	Mus musculus CCR4 carbon catabolite repression 4-like (S. cerevisiae) (Ccrn4l), mRNA.
scl20945.14_483-S	60.5886	1.51	Epc2	NM_172663.2	
scl16475.25_424-S	60.3258	1.8	Per2	NM_011066.1	Mus musculus period homolog 2 (Drosophila) (Per2), mRNA.
scl00219189.1_160-S	60.0944	1.59	1300010F03Rik	XM_127785.5	Mus musculus RIKEN cDNA 1300010F03 gene (1300010F03Rik), mRNA.
scl070257.1_12-S	59.9968	1.43	2010107E04Rik	NM_027360.1	Mus musculus RIKEN cDNA 2010107E04 gene (2010107E04Rik), mRNA.

scl056275.3_18-S	59.9741	2.1	Rbm14	NM_019869.1	Mus musculus RNA binding motif protein 14 (Rbm14), mRNA.
scl29854.5.1_26-S	59.9667	1.79	Aup1	NM_007517.1	Mus musculus ancient ubiquitous protein (Aup1), mRNA.
scl52870.7_104-S	59.8541	1.88	Ehd1	NM_010119.3	Mus musculus EH-domain containing 1 (Ehd1), mRNA.
scl0019043.2_303-S	59.8267	1.39	Ppm1b	NM_011151.1	Mus musculus protein phosphatase 1B, magnesium dependent, beta isoform (Ppm1b), mRNA.
scl054381.6_56-S	59.8264	1.69	Pgcp	NM_176073.2	Mus musculus plasma glutamate carboxypeptidase (Pgcp), mRNA.
scl51004.4_55-S	59.5655	1.52	Sepx1	NM_013759.1	
scl44723.2_201-S	59.5043	1.41	B230219D22Rik	NM_181278.2	Mus musculus RIKEN cDNA B230219D22 gene (B230219D22Rik), mRNA.
scl075705.2_53-S	59.4992	2.1	Eif4b	NM_145625.1	Mus musculus eukaryotic translation initiation factor 4B (Eif4b), mRNA.
scl0230866.24_27-S	59.4961	3.2	C230096C10Rik	NM_146157.2	Mus musculus RIKEN cDNA C230096C10 gene (C230096C10Rik), mRNA.
scl49145.9_59-S	59.4087	2.53	B4galt4	NM_019804.1	Mus musculus UDP-Gal:betaGlcNAc beta 1,4-galactosyltransferase, polypeptide 4 (B4galt4), mRNA.
scl000724.1_245-S	59.2955	2.23	Calr3	NM_029782.2	Mus musculus calreticulin 3 (Calr3), mRNA.
scl33996.15_314-S	59.2234	1.45	Slc20a2	NM_011394.1	Mus musculus solute carrier family 20, member 2 (Slc20a2), mRNA.
scl0051810.1_209-S	58.832	2.56	Hnrpu	NM_016805.1	Mus musculus heterogeneous nuclear ribonucleoprotein U (Hnrpu), mRNA.
scl30789.23.1_30-S	58.7886	2.19	Copb1	NM_033370	Mus musculus coatomer protein complex, subunit beta 1 (Copb1), mRNA.
scl28744.16_312-S	58.6418	1.88	Anxa4	NM_013471	Mus musculus annexin A4 (Anxa4), mRNA.
scl0004113.1_27-S	58.6064	1.36	Mrpl33	NM_025796.1	Mus musculus mitochondrial ribosomal protein L33 (Mrpl33), mRNA.
scl0012908.2_4-S	58.5935	1.46	Crat	NM_007760.1	Mus musculus carnitine acetyltransferase (Crat), mRNA.
scl30654.9_73-S	58.541	2.15	1110003P22Rik	NM_144522	Mus musculus RIKEN cDNA 1110003P22 gene (1110003P22Rik), mRNA.
scl021981.1_87-S	58.4956	1.98	Ppp1r13b	NM_011625.1	Mus musculus protein phosphatase 1, regulatory (inhibitor) subunit 13B (Ppp1r13b), mRNA.
scl0017289.2_279-S	58.326	2.8	Mertk	NM_008587.1	Mus musculus c-mer proto-oncogene tyrosine kinase (Mertk), mRNA.
scl43457.5.1_29-S	58.3238	1.37	Mrps30	NM_021556.2	Mus musculus mitochondrial ribosomal protein S30 (Mrps30), mRNA.
scl098766.1_292-S	58.3065	1.41	Ubadc1	NM_133835.1	Mus musculus ubiquitin associated domain containing 1 (Ubadc1), mRNA.
scl077781.2_11-S	58.0195	2.34	Epm2aip1	NM_175266.2	Mus musculus EPM2A (laforin) interacting protein 1 (Epm2aip1), mRNA.
scl0019769.2_219-S	57.9868	1.45	Rit1	NM_009069.2	Mus musculus Ras-like without CAAX 1 (Rit1), mRNA.
scl066869.1_197-S	57.2674	2.14	1200003I07Rik	XM_134220.1	Mus musculus RIKEN cDNA 1200003I07 gene (1200003I07Rik), mRNA.
scl0078655.1_180-S	57.1289	1.52	Eif3s1	NM_144545.1	Mus musculus eukaryotic translation initiation factor 3, subunit 1 alpha (Eif3s1), mRNA.
scl35945.10_223-S	56.6996	1.83	Arcn1	NM_145985.2	Mus musculus archain 1 (Arcn1), mRNA.
scl072085.4_41-S	56.6825	1.44	Osgepl1	NM_028091.2	Mus musculus O-sialoglycoprotein endopeptidase-like 1 (Osgepl1), mRNA.
scl066144.2_329-S	56.5774	1.44	Atp6v1f	NM_025381	Mus musculus ATPase, H+ transporting, V1 subunit F (Atp6v1f), mRNA.
scl018538.1_192-S	56.531	1.73	Pcna	NM_011045.1	Mus musculus proliferating cell nuclear antigen (Pcna), mRNA.
scl072284.5_21-S	56.4565	2.63	Oraov1	NM_028184.2	Mus musculus oral cancer overexpressed 1 (Oraov1), mRNA.
scl34490.14.1_30-S	56.4225	1.98	Ces3	NM_053200.1	Mus musculus carboxylesterase 3 (Ces3), mRNA.
scl24019.5_109-S	56.41	1.44	Cpt2	NM_009949	Mus musculus carnitine palmitoyltransferase 2 (Cpt2), mRNA.
scl015122.2_7-S	56.0896	2.13	Hba-a1	NM_008218.1	Mus musculus hemoglobin alpha, adult chain 1 (Hba-a1), mRNA.
scl066151.5_253-S	56.0681	1.38	1110020C13Rik	NM_025385.2	Mus musculus RIKEN cDNA 1110020C13 gene (1110020C13Rik), mRNA.
scl072686.1_177-S	56.0613	1.45	Usp24	XM_131566.5	
scl18579.23.1_24-S	55.9594	1.53	Rrbp1	XM_130532.1	Mus musculus ribosome binding protein 1 (Rrbp1), mRNA.
scl0015078.1_248-S	55.892	1.35	H3f3a	NM_008210.2	Mus musculus H3 histone, family 3A (H3f3a), mRNA.
scl030057.2_1-S	55.7951	1.36	Timm8b	NM_013897.1	Mus musculus translocase of inner mitochondrial membrane 8 homolog b (yeast) (Timm8b), mRNA.
scl0237898.1_282-S	55.7599	1.76	6430526O11Rik	XM_110937.4	
scl0067673.1_322-S	55.6282	1.38	Tceb2	NM_026305.1	Mus musculus transcription elongation factor B (SIII), polypeptide 2 (Tceb2), mRNA.
scl40755.5.1_55-S	55.4836	1.65	D11Wsu47e	NM_177777.3	Mus musculus DNA segment, Chr 11, Wayne State University 47, expressed (D11Wsu47e), mRNA.
scl015107.1_301-S	55.0458	2.51	Hadhsc	NM_008212.1	Mus musculus L-3-hydroxyacyl-Coenzyme A dehydrogenase, short chain (Hadhsc), mRNA.
scl0003902.1_2-S	55.0145	2.56	Snx3	NM_017472.2	Mus musculus sorting nexin 3 (Snx3), mRNA.
scl093739.4_24-S	54.9479	2.33	Gabarapl2	NM_026693.2	Mus musculus GABA(A) receptor-associated protein like 2 (Gabarapl2), mRNA.
scl0067618.2_236-S	54.4488	2.08	Aasdhppt	NM_026276.1	Mus musculus aminoadipate-semialdehyde dehydrogenase-phosphopantetheinyl transferase (Aasdhppt), mRNA.
scl0074144.2_313-S	54.3203	1.5	Robo4	NM_028783.2	Mus musculus roundabout homolog 4 (Drosophila) (Robo4), mRNA.
scl50134.9_8-S	54.1864	1.57	Nudt3	NM_019837.1	Mus musculus nudix (nucleotide diphosphate linked moiety X)-type motif 3 (Nudt3), mRNA.
scl071704.9_277-S	54.0878	1.38	Arhgef3	NM_027871.1	Mus musculus Rho guanine nucleotide exchange factor (GEF) 3 (Arhgef3), mRNA.
scl34266.7_421-S	53.9277	1.71	2700067E09Rik	NM_175185.2	Mus musculus RIKEN cDNA 2700067E09 gene (2700067E09Rik), mRNA.
scl0338522.1_21-S	53.886	2.44	9230115A19Rik	XM_110546.2	
scl48128.6_4-S	53.88	1.48	2900001O04Rik	NM_026213.2	Mus musculus RIKEN cDNA 2900001O04 gene (2900001O04Rik), mRNA.
scl0003659.1_24-S	53.7811	2.57	Mef2c	NM_025282.1	Mus musculus myocyte enhancer factor 2C (Mef2c), mRNA.
scl0013640.1_290-S	53.778	1.46	Efna5	NM_207654.1	Mus musculus ephrin A5 (Efna5), transcript variant 2, mRNA.
scl00232227.2_246-S	53.7742	1.45	D6Ertd349e	NM_182784.1	Mus musculus DNA segment, Chr 6, ERATO Doi 349, expressed (D6Ertd349e), mRNA.
scl00109113.1_250-S	53.6581	1.5	Uhrf2	NM_144873.1	Mus musculus ubiquitin-like, containing PHD and RING finger domains 2 (Uhrf2), mRNA.

scl0067452.2_1-S	53.648	1.35	1200006O19Rik	NM_026164.1	Mus musculus RIKEN cDNA 1200006O19 gene (1200006O19Rik), mRNA.
scl0056430.2_105-S	53.5577	1.49	Rsn	NM_019765.2	Mus musculus restin (Reed-Steinberg cell-expressed intermediate filament-associated protein) (Rsn), mRNA.
scl29044.3_57-S	53.3508	1.83	Ian6	NM_153175.2	Mus musculus immune associated nucleotide 6 (Ian6), mRNA.
scl28963.13.1_27-S	53.2282	1.41	Nt5c3	NM_026004.1	Mus musculus 5-nucleotidase, cytosolic III (Nt5c3), mRNA.
scl29636.20.1_18-S	53.1752	1.73	1110061O04Rik	NM_026849	Mus musculus RIKEN cDNA 1110061O04 gene (1110061O04Rik), mRNA.
scl25801.8_88-S	53.1287	1.72	Rac1	NM_009007.1	Mus musculus RAS-related C3 botulinum substrate 1 (Rac1), mRNA.
scl16995.8.6_15-S	52.8353	1.56	Cops5	NM_013715.1	Mus musculus COP9 (constitutive photomorphogenic) homolog, subunit 5 (Arabidopsis thaliana) (Cops5), mRNA.
scl32687.7.1_13-S	52.8259	1.42	Hrc	NM_010473.1	Mus musculus histidine rich calcium binding protein (Hrc), mRNA.
scl0003853.1_11-S	52.7947	1.84	Lta4h	NM_008517.1	Mus musculus leukotriene A4 hydrolase (Lta4h), mRNA.
scl48796.17_240-S	52.7099	1.77	2700067D09Rik	NM_028301.1	Mus musculus RIKEN cDNA 2700067D09 gene (2700067D09Rik), mRNA.
scl00268996.2_185-S	52.5167	1.41	Ss18	NM_009280.1	Mus musculus synovial sarcoma translocation, Chromosome 18 (Ss18), mRNA.
scl19885.7_41-S	52.4475	1.42	Zfp313	NM_030743.3	Mus musculus zinc finger protein 313 (Zfp313), mRNA.
scl45764.20_72-S	52.4451	1.34	Nisch	NM_022656.1	Mus musculus nischarin (Nisch), mRNA.
scl071228.1_292-S	52.1073	1.7	Dlg5	XM_127605.4	Mus musculus discs, large homolog 5 (Drosophila) (Dlg5), mRNA.
scl36423.7.11_132-S	51.9702	1.79	Myl3	NM_010859.1	Mus musculus myosin, light polypeptide 3, alkali; ventricular, skeletal, slow (Myl3), mRNA.
scl067281.3_0-S	51.9427	1.44	Rpl37	NM_026069.1	Mus musculus ribosomal protein L37 (Rpl37), mRNA.
scl099650.7_29-S	51.8261	1.53	4933434E20Rik	NM_025762.1	Mus musculus RIKEN cDNA 4933434E20 gene (4933434E20Rik), mRNA.
scl0056347.2_235-S	51.7718	2.04	Eif3s8	NM_019646.1	Mus musculus eukaryotic translation initiation factor 3, subunit 8 (Eif3s8), mRNA.
scl23442.8_471-S	51.6614	1.5	1110060F11Rik	NM_026395.1	Mus musculus RIKEN cDNA 1110060F11 gene (1110060F11Rik), mRNA.
scl27262.6_658-S	51.5691	1.35	9130017A15Rik	NM_177242.3	Mus musculus RIKEN cDNA 9130017A15 gene (9130017A15Rik), mRNA.
scl53360.9.1_13-S	51.5456	4.2	Ms4a6b	NM_027209.2	Mus musculus membrane-spanning 4-domains, subfamily A, member 6B (Ms4a6b), mRNA.
scl0003527.1_242-S	51.5347	1.4	Ddx6	NM_007841.2	Mus musculus DEAD (Asp-Glu-Ala-Asp) box polypeptide 6 (Ddx6), mRNA.
scl067581.1_259-S	51.476	1.56	4930451A13Rik	NM_026254.2	Mus musculus RIKEN cDNA 4930451A13 gene (4930451A13Rik), mRNA.
scl0099650.1_60-S	51.3261	1.56	4933434E20Rik	NM_025762.1	Mus musculus RIKEN cDNA 4933434E20 gene (4933434E20Rik), mRNA.
scl50670.2.184_270-S	51.1817	1.49	2310039H08Rik	NM_025966.2	Mus musculus RIKEN cDNA 2310039H08 gene (2310039H08Rik), mRNA.
scl34388.19_15-S	51.1341	1.53	Ranbp10	NM_145824.3	
scl47805.7_5-S	51.1127	1.53	Grina	NM_023168.2	Mus musculus glutamate receptor, ionotropic, N-methyl D-asparate-associated protein 1 (glutamate binding) (Grina), mRNA.
scl48815.9.1_11-S	51.0109	7.15	Crebbp	XM_148699.3	Mus musculus CREB binding protein (Crebbp), mRNA.
scl0015587.1_282-S	50.8726	1.4	Hyal2	NM_010489.2	Mus musculus hyaluronidase 2 (Hyal2), mRNA.
scl55045.9_124-S	50.743	1.4	Tm4sf2	NM_019634.1	Mus musculus transmembrane 4 superfamily member 2 (Tm4sf2), mRNA.
scl22978.7.1_18-S	50.4696	1.34	Krtcap2	NM_025327.1	Mus musculus keratinocyte associated protein 2 (Krtcap2), mRNA.
scl49200.44.268_9-S	50.3771	1.96	Mylk	NM_139300	Mus musculus myosin, light polypeptide kinase (Mylk), mRNA.
scl074383.1_23-S	50.3408	1.9	Ubap2l	NM_028475.1	
scl38628.18.8_108-S	50.3191	2.53	Txnrd1	NM_015762.1	Mus musculus thioredoxin reductase 1 (Txnrd1), mRNA.
scl42971.1_9-S	50.2789	1.64	0710001C05Rik	XM_203592.1	Mus musculus RIKEN cDNA 0710001C05 gene (0710001C05Rik), mRNA.
scl093836.1_167-S	50.0768	3.62	Rnf111	NM_033604.1	Mus musculus ring finger 111 (Rnf111), mRNA.
scl52858.1.11_122-S	50.035	2.23	Znhit2	NM_013859.1	
scl011544.1_20-S	50.0215	1.34	Adprh	NM_007414.2	Mus musculus ADP-ribosylarginine hydrolase (Adprh), mRNA.
scl53173.13_456-S	49.9947	1.55	E430027O22Rik	XM_129248.4	Mus musculus RIKEN cDNA E430027O22 gene (E430027O22Rik), mRNA.
scl072193.1_10-S	49.9048	1.69	Sfrs2ip	XM_128178.5	
scl00214137.2_222-S	49.8937	1.41	B130017I01Rik	NM_172525.1	Mus musculus RIKEN cDNA B130017I01 gene (B130017I01Rik), mRNA.
scl16458.7.1_28-S	49.6925	1.65	Thap4	NM_025920	Mus musculus THAP domain containing 4 (Thap4), mRNA.
scl35462.25_609-S	49.6635	1.59	Pik3cb	NM_029094.1	Mus musculus phosphatidylinositol 3-kinase, catalytic, beta polypeptide (Pik3cb), mRNA.
scl21707.11_464-S	49.6222	1.68	Ddx20	NM_017397.2	Mus musculus DEAD (Asp-Glu-Ala-Asp) box polypeptide 20 (Ddx20), mRNA.
scl18420.2_47-S	49.5796	1.55	Blcap	NM_016916.2	Mus musculus bladder cancer associated protein homolog (human) (Blcap), mRNA.
scl40289.5_205-S	49.5731	1.64	Trim41	NM_145377.1	Mus musculus tripartite motif-containing 41 (Trim41), mRNA.
scl30301.28.1_43-S	49.5514	2.29	AL033314	NM_019776	Mus musculus expressed sequence AL033314 (AL033314), mRNA.
scl50963.5.1_38-S	49.5468	1.6	Mrpl28	NM_024227.2	Mus musculus mitochondrial ribosomal protein L28 (Mrpl28), nuclear gene encoding mitochondrial protein, mRNA.
scl056771.4_27-S	49.4891	1.64	Usp49	NM_020048.2	Mus musculus ubiquitin specific protease 49 (Usp49), mRNA.
scl41114.9.1_30-S	49.4129	2.04	Car4	NM_007607.1	Mus musculus carbonic anhydrase 4 (Car4), mRNA.
scl018139.26_3-S	49.2378	1.52	Zfml	NM_008717.1	
scl31560.6.38_45-S	49.0572	1.53	1110006G06Rik	NM_028661.1	Mus musculus RIKEN cDNA 1110006G06 gene (1110006G06Rik), mRNA.
scl000063.1_0-S	49.0359	1.44	Nit1	NM_012049.1	Mus musculus nitrilase 1 (Nit1), mRNA.
scl50495.21.3_143-S	49.0079	1.95	2610511O17Rik	NM_152817.2	Mus musculus RIKEN cDNA 2610511O17 gene (2610511O17Rik), mRNA.
gi_31981889_ref_NM_00	48.8991	1.48	B2m	NM_009735.2	Mus musculus beta-2 microglobulin (B2m), mRNA.
scl0229488.1_136-S	48.8716	2.65	9930021J17Rik	NM_172682.1	Mus musculus RIKEN cDNA 9930021J17 gene (9930021J17Rik), mRNA.

scl37183.6.1_31-S	48.8064	1.61	Thy28	NM_144543.1	Mus musculus thymocyte protein thy28 (Thy28), mRNA.
scl066836.1_32-S	48.7753	1.84	0610006I08Rik	NM_025791.1	Mus musculus RIKEN cDNA 0610006I08 gene (0610006I08Rik), mRNA.
scl47911.5_100-S	48.7155	2.98	Nov	NM_010930.3	Mus musculus nephroblastoma overexpressed gene (Nov), mRNA.
scl073373.8_41-S	48.6326	1.43	1700048E23Rik	NM_028521.1	Mus musculus RIKEN cDNA 1700048E23 gene (1700048E23Rik), mRNA.
scl0000100.1_15-S	48.4716	1.75	Tnxb	NM_031176.1	Mus musculus tenascin XB (Tnxb), mRNA.
scl0022661.2_277-S	48.4255	1.68	Zfp148	NM_011749.3	Mus musculus zinc finger protein 148 (Zfp148), mRNA.
scl012757.9_180-S	48.2625	1.91	Clta	NM_016760.1	Mus musculus clathrin, light polypeptide (Lca) (Clta), mRNA.
scl0105445.1_143-S	48.1631	1.8	Dock9	XM_358315.1	
scl24842.10.1_114-S	48.1326	2.45	Rhced	NM_011270.2	Mus musculus Rhesus blood group CE and D (Rhced), mRNA.
scl28291.1_56-S	48.124	1.38	Tctex1	NM_009342	Mus musculus t-complex testis expressed 1 (Tctex1), mRNA.
scl19927.15.1_16-S	48.1171	1.44	Dnttip1	NM_133763.1	Mus musculus deoxynucleotidyltransferase, terminal, interacting protein 1 (Dnttip1), mRNA.
scl18972.12.1_41-S	48.0692	1.46	1810020C19Rik	XM_130317.5	Mus musculus RIKEN cDNA 1810020C19 gene (1810020C19Rik), mRNA.
scl27294.16.1_239-S	48.0001	1.59	Slc24a6	NM_133221.1	Mus musculus solute carrier family 24 (sodium/potassium/calcium exchanger), member 6 (Slc24a6), mRNA.
scl53773.1.1793_9-S	47.9908	2.19	Acsl4	NM_207625.1	Mus musculus acyl-CoA synthetase long-chain family member 4 (Acsl4), transcript variant 2, mRNA.
scl23055.15.1_74-S	47.9886	1.35	Plrg1	NM_016784.2	Mus musculus pleiotropic regulator 1, PRL1 homolog (Arabidopsis) (Plrg1), mRNA.
scl0076895.2_316-S	47.8528	2.03	Bicd2	NM_029791.2	Mus musculus bicaudal D homolog 2 (Drosophila) (Bicd2), mRNA.
scl23588.20_436-S	47.8425	1.39	Plekhm2	XM_204109.2	
scl49667.33.1_65-S	47.8194	1.44	Ptprm	NM_008984	Mus musculus protein tyrosine phosphatase, receptor type, M (Ptprm), mRNA.
scl36630.14.1_50-S	47.8017	1.65	Ctsh	NM_007801	Mus musculus cathepsin H (Ctsh), mRNA.
scl018641.1_29-S	47.7334	1.33	Pfkl	NM_008826.2	Mus musculus phosphofructokinase, liver, B-type (Pfkl), mRNA.
scl44186.8.1_13-S	47.6618	1.65	Gmnn	NM_020567.1	Mus musculus geminin (Gmnn), mRNA.
scl0105841.13_268-S	47.6262	1.79	E030003N15Rik	XM_128129.4	Mus musculus RIKEN cDNA E030003N15 gene (E030003N15Rik), mRNA.
scl0003984.1_492-S	47.5785	1.38	Atp2a2	NM_009722.1	Mus musculus ATPase, Ca++ transporting, cardiac muscle, slow twitch 2 (Atp2a2), mRNA.
scl51735.42.647_134-S	47.4972	1.87	5430411K18Rik	XM_140491.4	Mus musculus RIKEN cDNA 5430411K18 gene (5430411K18Rik), mRNA.
scl24797.1_325-S	47.4102	1.56	Usp48	NM_028344.1	
scl33848.6_136-S	47.4095	1.44	4933411K20Rik	NM_025747.2	Mus musculus RIKEN cDNA 4933411K20 gene (4933411K20Rik), mRNA.
scl36105.10.25_13-S	47.3989	1.83	Tbx20	NM_194263.1	Mus musculus T-box 20 (Tbx20), transcript variant 2, mRNA.
scl016418.1_114-S	47.3935	1.51	Itgb4bp	NM_010579.1	Mus musculus integrin beta 4 binding protein (Itgb4bp), mRNA.
scl0019699.2_228-S	47.3549	2.57	Reln	NM_011261.1	Mus musculus reelin (Reln), mRNA.
scl23536.7_579-S	47.3277	1.62	2610305D13Rik	NM_145078	Mus musculus RIKEN cDNA 2610305D13 gene (2610305D13Rik), mRNA.
scl069077.10_34-S	47.1041	2.41	Psmd11	NM_178616.2	Mus musculus proteasome (prosome, macropain) 26S subunit, non-ATPase, 11 (Psmd11), mRNA.
scl0026367.1_126-S	47.099	1.69	Ceacam2	NM_007543.2	Mus musculus CEA-related cell adhesion molecule 2 (Ceacam2), mRNA.
scl29310.11_209-S	47.0957	2.75	Pdk4	NM_013743.1	Mus musculus pyruvate dehydrogenase kinase, isoenzyme 4 (Pdk4), mRNA.
scl38525.3.714_1-S	47.0039	1.59	Ube2n	NM_080560.2	Mus musculus ubiquitin-conjugating enzyme E2N (Ube2n), mRNA.
scl015289.1_26-S	46.974	1.52	Hmgb1	NM_010439.2	Mus musculus high mobility group box 1 (Hmgb1), mRNA.
scl0012934.1_152-S	46.8969	1.32	Dpysl2	NM_009955.2	Mus musculus dihydropyrimidinase-like 2 (Dpysl2), mRNA.
scl0057261.1_145-S	46.8449	1.38	Brd4	NM_020508.2	Mus musculus bromodomain containing 4 (Brd4), mRNA.
scl018639.4_213-S	46.831	8.98	Pfkfb1	XM_284750.2	Mus musculus 6-phosphofructo-2-kinase/fructose-2,6-biphosphatase 1 (Pfkfb1), mRNA.
scl52515.14.1_211-S	46.7833	1.46	5730455O13Rik	XM_129261.5	Mus musculus RIKEN cDNA 5730455O13 gene (5730455O13Rik), mRNA.
scl022333.10_87-S	46.7729	1.37	Vdac1	NM_011694	Mus musculus voltage-dependent anion channel 1 (Vdac1), mRNA.
scl49983.22_274-S	46.7648	1.56	Ddr1	NM_007584.1	Mus musculus discoidin domain receptor family, member 1 (Ddr1), mRNA.
scl069612.1_312-S	46.7135	2.93	2310037I24Rik	NM_133714.2	Mus musculus RIKEN cDNA 2310037I24 gene (2310037I24Rik), mRNA.
scl46496.6.1_3-S	46.6645	1.44	Tnnc1	NM_009393.1	
scl0227634.1_319-S	46.6513	1.85	Camsap1	XM_129375.4	
scl0230126.1_281-S	46.6187	1.58	Shb	XM_131399.4	Mus musculus src homology 2 domain-containing transforming protein B (Shb), mRNA.
scl25126.19.1_29-S	46.6179	1.63	Faf1	NM_007983.2	Mus musculus Fas-associated factor 1 (Faf1), mRNA.
scl40876.7.1_108-S	46.5317	1.37	Rad52b	NM_025654.1	Mus musculus RAD52 homolog B (S. cerevisiae) (Rad52b), mRNA.
scl49542.15_45-S	46.4742	2	D030028O16Rik	NM_145984.2	Mus musculus RIKEN cDNA D030028O16 gene (D030028O16Rik), mRNA.
scl068024.2_210-S	46.2806	1.89	Hist1h2bc	NM_023422.1	Mus musculus histone 1, H2bc (Hist1h2bc), mRNA.
scl18935.13_58-S	46.0453	4.01	Cat	NM_009804	Mus musculus catalase (Cat), mRNA.
scl20689.4.1_4-S	45.9934	1.46	Timm13a	NM_013899.1	Mus musculus translocase of inner mitochondrial membrane 13 homolog a (yeast) (Timm13a), mRNA.
scl0021339.1_180-S	45.9842	1.69	Taf1a	NM_021466.1	Mus musculus TATA box binding protein (Tbp)-associated factor, RNA polymerase I, A (Taf1a), mRNA.
scl014009.13_0-S	45.9462	1.45	Etv1	NM_007960.1	Mus musculus ets variant gene 1 (Etv1), mRNA.
scl25510.19.1_73-S	45.8103	1.37	Npr2	NM_173788.2	Mus musculus natriuretic peptide receptor 2 (Npr2), mRNA.
scl052609.1_148-S	45.8091	1.53	Cbx7	NM_144811.2	
scl47641.14_223-S	45.6683	1.5	D15Ertd781e	NM_145476.2	

scl0067958.1_275-S	45.571	1.35	2610101N10Rik	NM_026476.1	Mus musculus RIKEN cDNA 2610101N10 gene (2610101N10Rik), mRNA.
scl0217893.6_312-S	45.5247	1.6	6720425G15Rik	NM_198165.1	Mus musculus RIKEN cDNA 6720425G15 gene (6720425G15Rik), mRNA.
scl0003447.1_0-S	45.4148	1.77	Rbm5	NM_148930.2	Mus musculus RNA binding motif protein 5 (Rbm5), mRNA.
scl0026554.2_150-S	45.3534	1.32	Cul3	NM_016716.2	Mus musculus cullin 3 (Cul3), mRNA.
scl0066691.1_167-S	45.3385	1.41	4432404J10Rik	NM_025709.2	Mus musculus RIKEN cDNA 4432404J10 gene (4432404J10Rik), mRNA.
scl016971.1_277-S	45.2741	1.94	Lrp1	NM_008512.1	Mus musculus low density lipoprotein receptor-related protein 1 (Lrp1), mRNA.
scl36067.18_4-S	45.2041	1.98	Aplp2	NM_009691.1	Mus musculus amyloid beta (A4) precursor-like protein 2 (Aplp2), mRNA.
scl0002734.1_23-S	45.1344	1.5	Ssbp3	NM_198438.1	Mus musculus single-stranded DNA binding protein 3 (Ssbp3), transcript variant 2, mRNA.
scl25758.6.1_14-S	45.0787	1.71	1200006F02Rik	NM_027872.1	Mus musculus RIKEN cDNA 1200006F02 gene (1200006F02Rik), mRNA.
scl20781.3.23_109-S	45.0031	2.25	1700011J10Rik	NM_183265	Mus musculus RIKEN cDNA 1700011J10 gene (1700011J10Rik), mRNA.
scl0071766.1_324-S	44.9829	2.23	1300006N24Rik	NM_027911.2	Mus musculus RIKEN cDNA 1300006N24 gene (1300006N24Rik), mRNA.
scl34359.7_33-S	44.9125	1.31	Psmd7	NM_010817.1	Mus musculus proteasome (prosome, macropain) 26S subunit, non-ATPase, 7 (Psmd7), mRNA.
scl013628.1_321-S	44.8774	1.61	Eef1a2	NM_007906.2	Mus musculus eukaryotic translation elongation factor 1 alpha 2 (Eef1a2), mRNA.
scl34595.13_308-S	44.874	1.4	Gab1	NM_021356.2	Mus musculus growth factor receptor bound protein 2-associated protein 1 (Gab1), mRNA.
scl23510.49_38-S	44.7945	6.09	Kif1b	NM_207682.1	Mus musculus kinesin family member 1B (Kif1b), transcript variant 1, mRNA.
scl17781.7.1_157-S	44.7917	1.57	D1Ertd161e	NM_026187.3	Mus musculus DNA segment, Chr 1, ERATO Doi 161, expressed (D1Ertd161e), mRNA.
scl52780.10_0-S	44.7754	2.24	Slc3a2	NM_008577.2	Mus musculus solute carrier family 3 (activators of dibasic and neutral amino acid transport), member 2 (Slc3a2), mRNA.
scl50103.16_203-S	44.6555	1.58	Mtch1	NM_019880.2	Mus musculus mitochondrial carrier homolog 1 (C. elegans) (Mtch1), mRNA.
scl30063.24.1_0-S	44.5546	8.06	Stk31	NM_029916.1	Mus musculus serine threonine kinase 31 (Stk31), mRNA.
scl067345.15_210-S	44.514	1.82	1700056O17Rik	NM_026101.2	Mus musculus RIKEN cDNA 1700056O17 gene (1700056O17Rik), mRNA.
scl20807.21.1_18-S	44.51	1.53	Dncic2	NM_010064	Mus musculus dynein, cytoplasmic, intermediate chain 2 (Dncic2), mRNA.
scl0004082.1_371-S	44.4987	2.56		XM_355695.1	Mus musculus similar to protocadherin 7 isoform c precursor; BH-pcdh; protocadherin 7; brain-heart protocadherin (LOC381720), mRNA.
scl013030.16_220-S	44.3933	1.29	Ctsb	NM_007798.1	Mus musculus cathepsin B (Ctsb), mRNA.
scl0015040.1_90-S	44.1812	1.49	H2-T23	NM_010398.1	Mus musculus histocompatibility 2, T region locus 23 (H2-T23), mRNA.
scl0068915.2_300-S	44.0664	1.7	Vars2l	NM_175137.3	Mus musculus valyl-tRNA synthetase 2-like (Vars2l), mRNA.
scl28548.7_53-S	43.9882	3	Camk1	NM_133926.1	Mus musculus calcium/calmodulin-dependent protein kinase I (Camk1), mRNA.
scl0001170.1_113-S	43.948	1.96	Crbn	NM_175357.1	
scl29467.5.1_148-S	43.946	1.52	Ocil	NM_053109.1	Mus musculus osteoclast inhibitory lectin (Ocil), mRNA.
scl40004.19.1_140-S	43.8797	1.5	Acadvl	NM_017366	Mus musculus acyl-Coenzyme A dehydrogenase, very long chain (Acadvl), mRNA.
scl0022359.2_126-S	43.8763	1.48	Vldlr	NM_013703.1	Mus musculus very low density lipoprotein receptor (Vldlr), mRNA.
scl0093762.1_285-S	43.8537	1.64	Smarca5	NM_053124.2	Mus musculus SWI/SNF related, matrix associated, actin dependent regulator of chromatin, subfamily a, member 5 (Smarca5), mRNA.
scl49960.7_412-S	43.6746	1.66	Trim39	NM_024468.1	Mus musculus tripartite motif protein 39 (Trim39), mRNA.
scl028036.1_230-S	43.6677	5.63	D3Wsu161e	NM_138593	Mus musculus DNA segment, Chr 3, Wayne State University 161, expressed (D3Wsu161e), mRNA.
scl0052708.2_28-S	43.6505	2.54	D12Ertd748e	NM_144833.1	Mus musculus DNA segment, Chr 12, ERATO Doi 748, expressed (D12Ertd748e), mRNA.
scl50800.2_484-S	43.5597	2.01	Hspa1l	NM_013558.1	Mus musculus heat shock protein 1-like (Hspa1l), mRNA.
scl056351.11_60-S	43.5196	3.16	Tebp	NM_019766.2	Mus musculus telomerase binding protein, p23 (Tebp), mRNA.
scl41798.24_462-S	43.5069	1.41	Vps54	NM_139061.2	Mus musculus vacuolar protein sorting 54 (yeast) (Vps54), mRNA.
scl34532.9_187-S	43.4295	1.34	Dnaja2	NM_019794.1	Mus musculus DnaJ (Hsp40) homolog, subfamily A, member 2 (Dnaja2), mRNA.
scl0003979.1_54-S	43.3174	2.16	Fastk	NM_023229.1	Mus musculus Fas-activated serine/threonine kinase (Fastk), mRNA.
scl0056422.1_70-S	43.2816	1.43	Hbs1l	NM_019702.1	Mus musculus Hbs1-like (S. cerevisiae) (Hbs1l), mRNA.
scl26088.14.1_16-S	43.1547	1.61	Aldh2	NM_009656.1	Mus musculus aldehyde dehydrogenase 2, mitochondrial (Aldh2), mRNA.
scl0011737.2_249-S	43.1168	1.37	Anp32a	NM_009672.2	Mus musculus acidic (leucine-rich) nuclear phosphoprotein 32 family, member A (Anp32a), mRNA.
scl016779.32_71-S	43.0674	1.68	Lamb2	NM_008483.2	Mus musculus laminin, beta 2 (Lamb2), mRNA.
scl00319713.1_52-S	43.0642	2.5	D930036B08Rik	NM_198649.1	Mus musculus RIKEN cDNA D930036B08 gene (D930036B08Rik), mRNA.
scl074270.26_30-S	42.9933	1.34	Usp20	NM_028846.1	Mus musculus ubiquitin specific protease 20 (Usp20), mRNA.
scl47705.17_95-S	42.8057	1.94	L3mbtl2	NM_145993.2	Mus musculus l(3)mbt-like 2 (Drosophila) (L3mbtl2), mRNA.
scl074549.2_261-S	42.7943	1.82	9130404D08Rik	NM_028993.2	Mus musculus RIKEN cDNA 9130404D08 gene (9130404D08Rik), mRNA.
scl011668.12_94-S	42.735	1.4	Aldh1a1	NM_013467	Mus musculus aldehyde dehydrogenase family 1, subfamily A1 (Aldh1a1), mRNA.
scl056529.1_95-S	42.6526	1.41	Spc18	NM_019951.1	Mus musculus signal peptidase complex (Spc18), mRNA.
scl50064.18_425-S	42.605	2.27	Wiz	NM_212438.1	Mus musculus widely-interspaced zinc finger motifs (Wiz), transcript variant 3, mRNA.
scl073316.1_23-S	42.5176	1.48	Calr3	NM_028500.1	Mus musculus calreticulin 3 (Calr3), mRNA.
scl0027406.2_22-S	42.5078	1.45	Abcf3	NM_013852.1	Mus musculus ATP-binding cassette, sub-family F (GCN20), member 3 (Abcf3), mRNA.
scl51323.13.1_30-S	42.4783	1.64	Mppe1	NM_172630.1	Mus musculus metallophosphoesterase 1 (Mppe1), mRNA.
scl013006.15_44-S	42.4551	1.65	Cspg6	NM_007790.2	Mus musculus chondroitin sulfate proteoglycan 6 (Cspg6), mRNA.
scl0319182.1_4-S	42.3615	1.62	Hist1h2bh	NM_178197.1	Mus musculus histone 1, H2bh (Hist1h2bh), mRNA.
scl0277333.1_280-S	42.2353	1.71	MGC68323	NM_199472.1	Mus musculus similar to glyceraldehyde-3-phosphate dehydrogenase (phosphorylating) (EC 1.2.1.12) - mouse (MGC68323), mRNA.

scl20502.5_452-S	42.1345	2	Lin7c	NM_011699	Mus musculus lin 7 homolog c (C. elegans) (Lin7c), mRNA.
scl22876.3_595-S	42.1284	1.31	Mcl1	NM_008562.2	Mus musculus myeloid cell leukemia sequence 1 (Mcl1), mRNA.
scl072656.1_14-S	42.1264	1.78	2810013E07Rik	NM_178112.3	Mus musculus RIKEN cDNA 2810013E07 gene (2810013E07Rik), mRNA.
scl012867.3_17-S	42.1166	1.39	Cox7c	NM_007749.1	Mus musculus cytochrome c oxidase, subunit VIIc (Cox7c), mRNA.
scl078325.1_150-S	42.1002	1.45	2700092H06Rik	XM_132529.2	Mus musculus RIKEN cDNA 2700092H06 gene (2700092H06Rik), mRNA.
scl0066660.1_237-S	42.0539	1.39	5730555F13Rik	NM_025690.1	Mus musculus RIKEN cDNA 5730555F13 gene (5730555F13Rik), mRNA.
scl31352.29.1_249-S	42.0242	1.76	Ush1c	NM_023649.1	Mus musculus Usher syndrome 1C homolog (human) (Ush1c), transcript variant a1, mRNA.
scl0026895.1_34-S	41.9523	1.74	Cops7b	NM_172974.1	Mus musculus COP9 (constitutive photomorphogenic) homolog, subunit 7b (Arabidopsis thaliana) (Cops7b), mRNA.
scl016865.11_15-S	41.9042	1.73	Lgtn	NM_010709.1	Mus musculus ligatin (Lgtn), mRNA.
scl36130.5_341-S	41.8983	1.6	Rab3d	NM_031874.3	Mus musculus RAB3D, member RAS oncogene family (Rab3d), mRNA.
scl054610.1_1-S	41.8693	2.39	Tbc1d8	NM_018775.1	Mus musculus TBC1 domain family, member 8 (Tbc1d8), mRNA.
scl33920.14_231-S	41.8662	1.43	Gsr	NM_010344.3	Mus musculus glutathione reductase 1 (Gsr), mRNA.
scl27178.12.102_2-S	41.8107	1.32	Cct6a	NM_009838	Mus musculus chaperonin subunit 6a (zeta) (Cct6a), mRNA.
scl0078785.2_44-S	41.7745	1.31	1700024K14Rik	NM_030179.2	Mus musculus RIKEN cDNA 1700024K14 gene (1700024K14Rik), mRNA.
scl0016969.1_242-S	41.758	2.98	Zbtb7	NM_010731.1	Mus musculus zinc finger and BTB domain containing 7 (Zbtb7), mRNA.
scl37776.6_58-S	41.7111	1.37	D10Jhu81e	NM_138601.1	Mus musculus DNA segment, Chr 10, Johns Hopkins University 81 expressed (D10Jhu81e), mRNA.
scl34649.4_549-S	41.665	1.97	Crsp7	NM_027485.1	Mus musculus cofactor required for Sp1 transcriptional activation, subunit 7 (Crsp7), mRNA.
scl015024.2_236-S	41.6284	2.15	H2-T10	NM_010395.2	Mus musculus histocompatibility 2, T region locus 10 (H2-T10), mRNA.
scl0239706.7_17-S	41.5751	1.49	BC024814	NM_146247	Mus musculus cDNA sequence BC024814 (BC024814), mRNA.
scl066725.8_229-S	41.5521	1.86	4921513O20Rik	NM_025730.1	Mus musculus RIKEN cDNA 4921513O20 gene (4921513O20Rik), mRNA.
scl34704.4_282-S	41.5099	1.47	C630013N10Rik	NM_172052.1	Mus musculus RIKEN cDNA C630013N10 gene (C630013N10Rik), mRNA.
scl40985.2.1_86-S	41.461	2.74	Hoxb4	NM_010459	Mus musculus homeo box B4 (Hoxb4), mRNA.
scl000488.1_1423-S	41.4572	5.86	Hectd2	NM_172637.1	
scl0099237.1_73-S	41.4011	1.99	Tm9sf4	NM_133847.2	
scl017169.9_120-S	41.3394	1.69	Mark3	NM_021516.1	Mus musculus MAP/microtubule affinity-regulating kinase 3 (Mark3), mRNA.
scl050850.17_73-S	41.1766	1.52	Spg4	NM_016962.1	Mus musculus spastic paraplegia 4 homolog (human) (Spg4), mRNA.
scl0272428.22_84-S	41.1606	3.82	C730027J19Rik	NM_178758.2	Mus musculus RIKEN cDNA C730027J19 gene (C730027J19Rik), mRNA.
scl012520.7_274-S	41.1309	1.38	Cd81	NM_133655.1	Mus musculus CD 81 antigen (Cd81), mRNA.
scl32299.7.1_25-S	41.0927	1.69	Stard10	NM_019990.1	Mus musculus START domain containing 10 (Stard10), mRNA.
scl012389.5_102-S	41.0776	1.28	Cav1	NM_007616.2	
scl0231225.1_255-S	41.0727	1.46	4932414K18Rik	NM_173764	Mus musculus RIKEN cDNA 4932414K18 gene (4932414K18Rik), mRNA.
scl000076.1_111_REVCO	41.0475	1.55	D11Wsu99e	NM_013581.2	Mus musculus DNA segment, Chr 11, Wayne State University 99, expressed (D11Wsu99e), mRNA.
scl18647.1_62-S	40.9424	1.33	Cenpb	NM_007682.2	Mus musculus centromere autoantigen B (Cenpb), mRNA.
scl54847.11_478-S	40.9401	1.33	Slc6a8	NM_133987.1	Mus musculus solute carrier family 6 (neurotransmitter transporter, creatine), member 8 (Slc6a8), mRNA.
scl0068965.1_205-S	40.888	2.37	1500010G04Rik	NM_173366.1	Mus musculus RIKEN cDNA 1500010G04 gene (1500010G04Rik), mRNA.
scl0106042.1_246-S	40.8718	1.37	Prickle1	XM_147935.1	Mus musculus prickle like 1 (Drosophila) (Prickle1), mRNA.
scl33340.4_635-S	40.8449	2.06	Txnl4b	NM_175646.2	
scl35708.10_49-S	40.8387	1.57	Smad3	NM_016769	
scl0001100.1_114-S	40.8268	1.59	Aqp1	NM_007472.1	Mus musculus aquaporin 1 (Aqp1), mRNA.
scl066361.1_72-S	40.7606	2.11	2310008M20Rik	NM_025512.2	Mus musculus RIKEN cDNA 2310008M20 gene (2310008M20Rik), mRNA.
scl0014567.2_263-S	40.7322	1.36	Gdi1	NM_010273.1	Mus musculus guanosine diphosphate (GDP) dissociation inhibitor 1 (Gdi1), mRNA.
scl50887.7_31-S	40.6955	1.29	Tex27	NM_148926.1	Mus musculus testis expressed gene 27 (Tex27), mRNA.
scl012153.1_23-S	40.6111	2.09	Bmp1	NM_009755.2	Mus musculus bone morphogenetic protein 1 (Bmp1), mRNA.
scl30418.7_5-S	40.5607	2.93	Asb4	NM_023048.4	Mus musculus ankyrin repeat and SOCS box-containing protein 4 (Asb4), mRNA.
scl33012.5.1_34-S	40.5471	1.38	Dmwd	NM_010058.1	Mus musculus dystrophia myotonica-containing WD repeat motif (Dmwd), mRNA.
scl065019.1_21-S	40.5162	1.27	Rpl23	NM_022891.1	Mus musculus ribosomal protein L23 (Rpl23), mRNA.
scl0002109.1_724-S	40.4556	1.39	Dnajb4	NM_025926.1	Mus musculus DnaJ (Hsp40) homolog, subfamily B, member 4 (Dnajb4), mRNA.
scl34424.10_15-S	40.3359	1.54	Tk2	NM_021028.2	Mus musculus thymidine kinase 2, mitochondrial (Tk2), mRNA.
scl011758.1_35-S	40.2604	1.47	Prdx6	NM_007453.2	Mus musculus peroxiredoxin 6 (Prdx6), mRNA.
scl0066704.2_280-S	40.2225	1.79	4921506I22Rik	NM_025717.2	Mus musculus RIKEN cDNA 4921506I22 gene (4921506I22Rik), mRNA.
scl27811.23.1_37-S	40.2043	1.45	2810453K03Rik	NM_144517.2	Mus musculus RIKEN cDNA 2810453K03 gene (2810453K03Rik), mRNA.
scl16978.3_2-S	40.1817	2.4	Msc	NM_010827	Mus musculus musculin (Msc), mRNA.
scl28573.11_3-S	40.1466	1.52	Crbn	NM_175357.1	
scl070652.1_1-S	40.1281	1.93	5730537D05Rik	NM_027495.2	Mus musculus RIKEN cDNA 5730537D05 gene (5730537D05Rik), mRNA.
scl45667.15_40-S	40.0914	1.31	Plekhc1	NM_146054.1	Mus musculus pleckstrin homology domain containing, family C (with FERM domain) member 1 (Plekhc1), mRNA.
scl0016599.2_295-S	40.0811	1.46	Klf3	NM_008453.2	Mus musculus Kruppel-like factor 3 (basic) (Klf3), mRNA.

scl056372.5_328-S	39.9773	3.67	1110004F10Rik	NM_019772.1	Mus musculus RIKEN cDNA 1110004F10 gene (1110004F10Rik), mRNA.
scl075786.2_11-S	39.9581	5.88	4930432B04Rik	XM_130287.5	Mus musculus RIKEN cDNA 4930432B04 gene (4930432B04Rik), mRNA.
scl052856.8_265-S	39.8619	1.72	Gtpbp5	NM_181424.2	Mus musculus GTP binding protein 5 (Gtpbp5), mRNA.
scl30362.8.2_277-S	39.8456	1.46	Kdt1	NM_175088.2	Mus musculus kidney cell line derived transcript 1 (Kdt1), mRNA.
scl066448.4_32-S	39.8177	1.28	Mrpl20	NM_025570.1	Mus musculus mitochondrial ribosomal protein L20 (Mrpl20), mRNA.
scl071779.8_36-S	39.7897	1.71	Mir	NM_027920.3	
scl28198.16.9_0-S	39.7625	2.22	4933424B01Rik	NM_138757.1	Mus musculus RIKEN cDNA 4933424B01 gene (4933424B01Rik), mRNA.
scl35530.18_2-S	39.6905	1.29	Mod1	NM_008615	Mus musculus malic enzyme, supernatant (Mod1), mRNA.
scl011981.2_30-S	39.6237	1.68	Atp9a	NM_015731.2	Mus musculus ATPase, class II, type 9A (Atp9a), mRNA.
scl0001999.1_48-S	39.5401	1.4	4933434E20Rik	NM_025762.1	Mus musculus RIKEN cDNA 4933434E20 gene (4933434E20Rik), mRNA.
scl0069740.2_2-S	39.5395	2.4	2410012M04Rik	NM_027193.2	Mus musculus RIKEN cDNA 2410012M04 gene (2410012M04Rik), mRNA.
scl40072.13_201-S	39.5068	1.4	Map2k4	NM_009157	Mus musculus mitogen activated protein kinase kinase 4 (Map2k4), mRNA.
scl066860.1_6-S	39.4994	1.42	1200003E16Rik	XM_130249.3	Mus musculus RIKEN cDNA 1200003E16 gene (1200003E16Rik), mRNA.
scl20100.20_8-S	39.3951	2.39	Tm9sf4	NM_133847.2	
scl20254.4.1_23-S	39.3742	1.66	1110034G24Rik	XM_130388.3	Mus musculus RIKEN cDNA 1110034G24 gene (1110034G24Rik), mRNA.
scl30620.3.1_5-S	39.3737	1.41	Cox6a2	NM_009943.1	Mus musculus cytochrome c oxidase, subunit VI a, polypeptide 2 (Cox6a2), mRNA.
scl24433.7.1_53-S	39.3319	1.56	Bag1	NM_009736.1	Mus musculus Bcl2-associated athanogene 1 (Bag1), mRNA.
scl017776.1_270-S	39.28	2.01	Mast2	NM_008641.1	Mus musculus microtubule associated serine/threonine kinase 2 (Mast2), mRNA.
scl43301.1.2269_3-S	39.2581	3.44	Gdap10	NM_010268.1	Mus musculus ganglioside-induced differentiation-associated-protein 10 (Gdap10), mRNA.
scl33219.11.1_14-S	39.187	1.55	BC021611	NM_144932.2	Mus musculus cDNA sequence BC021611 (BC021611), mRNA.
scl26322.8.1_52-S	39.1578	3.72	2310002F18Rik	XM_132218.3	Mus musculus RIKEN cDNA 2310002F18 gene (2310002F18Rik), mRNA.
scl23193.10.1_8-S	39.111	1.37	Alg5	NM_025442.1	Mus musculus asparagine-linked glycosylation 5 homolog (yeast, dolichyl-phosphate beta-glucosyltransferase) (Alg5), mRNA.
scl015191.7_7-S	39.0876	1.63	Hdgf	NM_008231.2	Mus musculus hepatoma-derived growth factor (Hdgf), mRNA.
scl22997.10_102-S	39.0798	1.42	AI663987	NM_033526.1	Mus musculus expressed sequence AI663987 (AI663987), mRNA.
scl24928.17_534-S	39.0554	1.83	Phc2	NM_018774.1	Mus musculus polyhomeotic-like 2 (Drosophila) (Phc2), mRNA.
scl0022608.1_233-S	39.005	1.27	Nsep1	NM_011732.1	Mus musculus nuclease sensitive element binding protein 1 (Nsep1), mRNA.
scl31410.39.1_13-S	38.9261	2.06	Myh14	NM_028021.1	
scl0057874.1_260-S	38.9175	1.32	AW742319	NM_021345.1	Mus musculus expressed sequence AW742319 (AW742319), mRNA.
scl016857.8_15-S	38.8584	2.08	Lgals4	NM_010706.1	Mus musculus lectin, galactose binding, soluble 6 (Lgals6), mRNA.
scl19291.9.1_25-S	38.8546	3.05	Nmi	NM_019401.1	Mus musculus N-myc (and STAT) interactor (Nmi), mRNA.
scl25917.6_37-S	38.7355	1.5	Ywhag	NM_018871.2	Mus musculus 3-monooxgenase/tryptophan 5-monooxgenase activation protein, gamma polypeptide (Ywhag), mRNA.
scl21772.5_341-S	38.7164	8.95	Hsd3b1	NM_008293.1	Mus musculus hydroxysteroid dehydrogenase-1, delta<5>-3-beta (Hsd3b1), mRNA.
scl54377.16.1_8-S	38.651	1.42	Maob	NM_172778.1	Mus musculus monoamine oxidase B (Maob), mRNA.
scl057905.1_53-S	38.6473	1.79	5830446M03Rik	NM_133934.2	Mus musculus RIKEN cDNA 5830446M03 gene (5830446M03Rik), mRNA.
scl23528.23_0-S	38.6268	1.28	Mfn2	NM_133201.1	Mus musculus mitofusin 2 (Mfn2), mRNA.
scl33420.4_697-S	38.61	1.57	Nol3	NM_030152.2	Mus musculus nucleolar protein 3 (apoptosis repressor with CARD domain) (Nol3), mRNA.
scl31074.15.1_32-S	38.5691	1.98	Fah	NM_010176.1	Mus musculus fumarylacetoacetate hydrolase (Fah), mRNA.
scl16317.12_198-S	38.5161	1.48	Mapkapk2	NM_008551.1	Mus musculus MAP kinase-activated protein kinase 2 (Mapkapk2), mRNA.
scl40024.13.1_29-S	38.4862	1.57	Senp3	NM_030702.2	Mus musculus SUMO/sentrin specific protease 3 (Senp3), mRNA.
scl0019046.2_75-S	38.4632	3.86	Ppp1cb	NM_172707.1	Mus musculus protein phosphatase 1, catalytic subunit, beta isoform (Ppp1cb), mRNA.
scl45413.15_171-S	38.4438	1.47	Elp3	NM_028811.1	Mus musculus elongation protein 3 homolog (S. cerevisiae) (Elp3), mRNA.
scl23489.7_25-S	38.4118	1.74	4930422J18Rik	NM_029035.2	Mus musculus RIKEN cDNA 4930422J18 gene (4930422J18Rik), mRNA.
scl23509.31_82-S	38.4	1.36	Ube4b	NM_022022.1	Mus musculus ubiquitination factor E4B, UFD2 homolog (S. cerevisiae) (Ube4b), mRNA.
scl020239.25_113-S	38.3891	1.3	Sca2	NM_009125.1	Mus musculus spinocerebellar ataxia 2 homolog (human) (Sca2), mRNA.
scl45160.32_9-S	38.345	1.83	Abcc4	XM_139262.2	Mus musculus ATP-binding cassette, sub-family C (CFTR/MRP), member 4 (Abcc4), mRNA.
scl0071720.1_33-S	38.344	2.1	Osbpl3	NM_027881.1	Mus musculus oxysterol binding protein-like 3 (Osbpl3), mRNA.
scl41017.13_82-S	38.2669	1.49	Ppp1r9b	NM_172261.1	Mus musculus protein phosphatase 1, regulatory subunit 9B (Ppp1r9b), mRNA.
scl00100986.1_185-S	38.247	1.47	Akap9	NM_194462.1	Mus musculus A kinase (PRKA) anchor protein (yotiao) 9 (Akap9), mRNA.
scl32196.12_2-S	38.2211	7.62	Wee1	NM_009516.2	Mus musculus wee 1 homolog (S. pombe) (Wee1), mRNA.
scl00319604.2_16-S	38.2018	1.87	B930006L02Rik	NM_178764.2	Mus musculus RIKEN cDNA B930006L02 gene (B930006L02Rik), mRNA.
gi_21070949_ref_NM_01	38.183	1.72	Ubc	XM_147315.1	Mus musculus ubiquitin C (Ubc), mRNA.
scl20734.8_508-S	38.1584	1.48	Plekha3	NM_031256.2	Mus musculus pleckstrin homology domain-containing, family A (phosphoinositide binding specific) member 3 (Plekha3), mRNA.
scl19758.9.1_11-S	38.11	1.78	Tcea2	NM_009326	Mus musculus transcription elongation factor A (SII), 2 (Tcea2), mRNA.
scl012314.1_140-S	38.1091	1.29	Calm2	NM_007589	Mus musculus calmodulin 2 (Calm2), mRNA.
scl47044.6.24_75-S	38.0915	1.35	0610041B22Rik	NM_025340.1	Mus musculus RIKEN cDNA 0610041B22 gene (0610041B22Rik), mRNA.
scl43966.3_250-S	38.0703	2.33	Nfil3	NM_017373.2	Mus musculus nuclear factor, interleukin 3, regulated (Nfil3), mRNA.

scl29631.10_10-S	37.9904	1.43	Creld1	NM_133930.1	Mus musculus cysteine-rich with EGF-like domains 1 (Creld1), mRNA.
scl0003894.1_174-S	37.9889	1.35	Amid	NM_153779.1	Mus musculus apoptosis-inducing factor (AIF)-like mitochondrion-associated inducer of death (Amid), mRNA.
scl31746.2.26_98-S	37.887	1.52	1500016L11Rik	NM_026885.1	Mus musculus RIKEN cDNA 1500016L11 gene (1500016L11Rik), mRNA.
scl34858.4_85-S	37.7698	1.8	Slc25a4	XM_134169.4	Mus musculus solute carrier family 25 (mitochondrial carrier; adenine nucleotide translocator), member 4 (Slc25a4), mRNA.
scl44140.2_304-S	37.7418	1.98	Uqcrfs1	NM_025710.1	Mus musculus ubiquinol-cytochrome c reductase, Rieske iron-sulfur polypeptide 1 (Uqcrfs1), mRNA.
scl0002744.1_1-S	37.7412	1.39	Zfyve9	XM_131643.4	
scl33121.3_245-S	37.7372	1.84	Zfp524	NM_025324.1	
scl42532.6_0-S	37.7208	1.99	Tm4sf13	NM_025359.2	Mus musculus transmembrane 4 superfamily member 13 (Tm4sf13), mRNA.
scl0106393.1_61-S	37.718	1.45	9830004M20Rik	NM_175347.3	
scl022110.2_300-S	37.557	3.02	Tspyl1	NM_009433.2	
scl26990.11.129_69-S	37.5414	1.4	Arpc1a	NM_019767.1	Mus musculus actin related protein 2/3 complex, subunit 1A (Arpc1a), mRNA.
scl29521.11_18-S	37.52	1.37	Grcc3f	NM_145130.1	Mus musculus gene rich cluster, C3f gene (Grcc3f), mRNA.
scl015207.2_11-S	37.4322	8.39	Hes3	NM_008237.1	Mus musculus hairy and enhancer of split 3 (Drosophila) (Hes3), mRNA.
scl0320949.2_42-S	37.3911	2.53	D830039M14Rik	NM_177297.2	Mus musculus RIKEN cDNA D830039M14 gene (D830039M14Rik), mRNA.
scl0021843.2_5-S	37.35	1.58	Tial1	NM_009383.1	Mus musculus Tial1 cytotoxic granule-associated RNA binding protein-like 1 (Tial1), mRNA.
scl24063.27.1_21-S	37.2842	1.57	Jak1	NM_146145.1	Mus musculus Janus kinase 1 (Jak1), mRNA.
scl48140.10_39-S	37.2533	1.92	Sepp1	NM_009155.2	Mus musculus selenoprotein P, plasma, 1 (Sepp1), mRNA.
scl30948.10_348-S	37.2442	1.78	AI849286	XM_194372.3	Mus musculus expressed sequence AI849286 (AI849286), mRNA.
scl0053413.2_143-S	37.2385	1.38	Exoc7	NM_016857.1	Mus musculus exocyst complex component 7 (Exoc7), mRNA.
scl0012192.2_207-S	37.2296	1.47	Zfp36l1	NM_007564.2	Mus musculus zinc finger protein 36, C3H type-like 1 (Zfp36l1), mRNA.
scl094112.1_23-S	37.1848	1.86	Pcqap	NM_033609.1	Mus musculus positive cofactor 2, multiprotein complex, glutamine/Q-rich-associated protein (Pcqap), mRNA.
scl41521.1.2_20-S	37.1689	7.9	4930438A08Rik	XM_126175.2	Mus musculus RIKEN cDNA 4930438A08 gene (4930438A08Rik), mRNA.
scl17286.15.1_2-S	37.1332	5.17	Selp	NM_011347.1	Mus musculus selectin, platelet (Selp), mRNA.
scl42127.12.1_16-S	37.1256	1.42	Lgmn	NM_011175.1	Mus musculus legumain (Lgmn), mRNA.
scl18417.10.1_104-S	37.1114	2.3	2610036D13Rik	NM_029282.1	Mus musculus RIKEN cDNA 2610036D13 gene (2610036D13Rik), mRNA.
scl24532.13_158-S	37.1014	2.75	Decr1	NM_026172.3	Mus musculus 2,4-dienoyl CoA reductase 1, mitochondrial (Decr1), mRNA.
scl013864.1_5-S	36.9608	1.44	Nr2f6	NM_010150	Mus musculus nuclear receptor subfamily 2, group F, member 6 (Nr2f6), mRNA.
scl0054216.2_72-S	36.9529	2.85	Pcdh7	NM_018764.1	Mus musculus protocadherin 7 (Pcdh7), mRNA.
scl52593.10.1_23-S	36.9421	1.34	5033414D02Rik	NM_026362.1	Mus musculus RIKEN cDNA 5033414D02 gene (5033414D02Rik), mRNA.
scl50203.3_209-S	36.9341	1.71	Gfer	NM_023040.2	Mus musculus growth factor, erv1 (S. cerevisiae)-like (augmenter of liver regeneration) (Gfer), transcript variant 2, mRNA.
scl00217692.2_267-S	36.9066	1.82	Sipa1l1	NM_172579.1	Mus musculus signal-induced proliferation-associated 1 like 1 (Sipa1l1), mRNA.
scl0229622.3_21-S	36.8881	1.74	9430063L05Rik	NM_178080.3	Mus musculus RIKEN cDNA 9430063L05 gene (9430063L05Rik), mRNA.
scl0081877.2_114-S	36.8879	1.69	Tnxb	NM_031176.1	Mus musculus tenascin XB (Tnxb), mRNA.
scl00107932.1_52-S	36.886	1.41	Chd4	NM_145979.1	Mus musculus chromodomain helicase DNA binding protein 4 (Chd4), mRNA.
scl068655.1_199-S	36.8845	4.5	1110027O12Rik	XM_354975.1	
scl53590.23.1_11-S	36.836	2.76	Ofd1	NM_177429.2	Mus musculus oral-facial-digital syndrome 1 gene homolog (human) (Ofd1), mRNA.
scl00234839.1_51-S	36.7976	1.59	2310061F22Rik	XM_134537.3	Mus musculus RIKEN cDNA 2310061F22 gene (2310061F22Rik), mRNA.
scl24261.12.1_29-S	36.7952	1.79	Alad	NM_008525.3	Mus musculus aminolevulinate, delta-, dehydratase (Alad), mRNA.
scl056278.9_3-S	36.7872	1.34	Gkap1	NM_019832.2	
scl0014082.2_318-S	36.6838	1.49	Fadd	NM_010175.2	Mus musculus Fas (TNFRSF6)-associated via death domain (Fadd), mRNA.
scl075956.6_20-S	36.5954	1.5	Srrm2	NM_175229.2	Mus musculus serine/arginine repetitive matrix 2 (Srrm2), mRNA.
scl50069.32.1454_21-S	36.5947	1.83	Notch3	NM_008716.1	Mus musculus Notch gene homolog 3 (Drosophila) (Notch3), mRNA.
scl23433.18.1_222-S	36.5898	1.35	2210008I11Rik	NM_145124	Mus musculus RIKEN cDNA 2210008I11 gene (2210008I11Rik), mRNA.
scl40603.7.49_6-S	36.5305	1.35	Drg1	NM_007879.1	Mus musculus developmentally regulated GTP binding protein 1 (Drg1), mRNA.
GI_23346428-S	36.4172	7.65	Myt1	NM_008665.2	Mus musculus myelin transcription factor 1 (Myt1), mRNA.
scl27246.12.1_228-S	36.3951	1.42	BC035291	NM_177581.2	Mus musculus cDNA sequence BC035291 (BC035291), mRNA.
scl0003163.1_0-S	36.3859	1.41	Mmrp19	NM_019735.2	Mus musculus monocyte macrophage 19 (Mmrp19), mRNA.
scl0001572.1_0-S	36.3684	1.36	Rai12	NM_018740.1	Mus musculus retinoic acid induced 12 (Rai12), mRNA.
scl0002068.1_1-S	36.3068	4	Casp6	NM_009811.2	Mus musculus caspase 6 (Casp6), mRNA.
scl36431.25.1_92-S	36.1953	1.75	Scap	XM_135190.4	Mus musculus Sreb cleavage-activating protein (Scap), mRNA.
scl014057.6_27-S	36.1724	1.47	Sfxn1	NM_027324.2	Mus musculus sideroflexin 1 (Sfxn1), mRNA.
scl000601.1_1063-S	36.164	1.51	Siat5	NM_178048.2	Mus musculus sialyltransferase 5 (Siat5), mRNA.
scl39906.6_377-S	36.0951	1.67	AI851076	XM_110955.2	Mus musculus expressed sequence AI851076 (AI851076), mRNA.
scl31978.3.3_6-S	36.0904	1.38	5730458D16Rik	NM_177609	Mus musculus RIKEN cDNA 5730458D16 gene (5730458D16Rik), mRNA.
scl0266692.1_279-S	36.0866	2.24	Cpne1	NM_170590.1	Mus musculus copine I (Cpne1), transcript variant 1, mRNA.
scl0230700.13_329-S	36.0642	1.32	C330039G02Rik	NM_172699.2	Mus musculus RIKEN cDNA C330039G02 gene (C330039G02Rik), mRNA.

scl33425.15_229-S	36.0435	1.47	D230025D16Rik	NM_145604.1	
scl00038.1_12-S	36.0383	1.5	Mrpl48	NM_026971.2	Mus musculus mitochondrial ribosomal protein L48 (Mrpl48), transcript variant 2, mRNA.
scl079555.6_1-S	36.0064	1.37	BC005537	NM_024473.2	Mus musculus cDNA sequence BC005537 (BC005537), mRNA.
scl17693.18_476-S	35.966	1.38	Apg16l	NM_029846.2	Mus musculus APG16 autophagy 16-like (S. cerevisiae) (Apg16l), transcript variant b, mRNA.
scl056772.1_0-S	35.9251	1.56	AI839562	NM_019914.2	Mus musculus expressed sequence AI839562 (AI839562), mRNA.
scl30452.18_578-S	35.8937	1.36	Nap1l4	NM_008672	Mus musculus nucleosome assembly protein 1-like 4 (Nap1l4), mRNA.
scl47166.29.1_41-S	35.8242	1.86	E430025E21Rik	NM_153548.2	Mus musculus RIKEN cDNA E430025E21 gene (E430025E21Rik), mRNA.
scl00100169.1_330-S	35.809	1.32	Phactr4	NM_175306.2	
scl26374.22_257-S	35.7211	2.48	Sdad1	NM_172713.1	Mus musculus SDA1 domain containing 1 (Sdad1), mRNA.
scl35445.15.1_13-S	35.6558	1.44	Pccb	NM_025835.1	Mus musculus propionyl Coenzyme A carboxylase, beta polypeptide (Pccb), mRNA.
scl023986.1_240-S	35.5801	2.64	Peci	NM_011868.1	Mus musculus peroxisomal delta3, delta2-enoyl-Coenzyme A isomerase (Peci), mRNA.
scl0002028.1_25-S	35.5799	1.3	2310066N05Rik	NM_027371.1	Mus musculus RIKEN cDNA 2310066N05 gene (2310066N05Rik), mRNA.
scl47755.2_19-S	35.5516	1.75	H1f0	NM_008197.2	Mus musculus H1 histone family, member 0 (H1f0), mRNA.
scl32101.7.1_5-S	35.4798	1.74	D7Wsu128e	NM_138589	Mus musculus DNA segment, Chr 7, Wayne State University 128, expressed (D7Wsu128e), mRNA.
scl46038.17.1_26-S	35.4707	1.4	Tdrd3	NM_172605.2	Mus musculus tudor domain containing 3 (Tdrd3), mRNA.
scl19566.6.1_18-S	35.4495	1.95	Dpp7	NM_031843.2	Mus musculus dipeptidylpeptidase 7 (Dpp7), mRNA.
scl068581.2_286-S	35.3826	1.27	1110014C03Rik	NM_026775.2	Mus musculus RIKEN cDNA 1110014C03 gene (1110014C03Rik), mRNA.
scl31465.20_298-S	35.368	7.57	9330164H19Rik	NM_178704.2	Mus musculus RIKEN cDNA 9330164H19 gene (9330164H19Rik), mRNA.
scl51774.11.3_64-S	35.3363	1.3	Cxxc1	NM_028868.1	Mus musculus CXXC finger 1 (PHD domain) (Cxxc1), mRNA.
scl066656.2_23-S	35.2994	1.25	Eef1d	NM_023240.1	Mus musculus eukaryotic translation elongation factor 1 delta (guanine nucleotide exchange protein) (Eef1d), mRNA.
scl25446.2_385-S	35.2982	1.37	2310039E09Rik	NM_026509.1	Mus musculus RIKEN cDNA 2310039E09 gene (2310039E09Rik), mRNA.
scl23602.11_383-S	35.2585	1.39	1110005F07Rik	NM_025383	Mus musculus RIKEN cDNA 1110005F07 gene (1110005F07Rik), mRNA.
scl42993.1.28_269-S	35.139	1.57	2410016O06Rik	NM_023633	Mus musculus RIKEN cDNA 2410016O06 gene (2410016O06Rik), mRNA.
scl0001996.1_45-S	35.1117	1.57	1110001A05Rik	NM_022554.1	Mus musculus RIKEN cDNA 1110001A05 gene (1110001A05Rik), mRNA.
scl0066125.2_261-S	34.9938	1.31	Sf3b5	NM_175102.2	
scl0056208.2_9-S	34.8441	1.29	Becn1	NM_019584.2	Mus musculus beclin 1 (coiled-coil, myosin-like BCL2-interacting protein) (Becn1), mRNA.
scl22729.4.1_31-S	34.8344	1.6	Amigo	NM_146137.1	Mus musculus amphoterin induced gene and ORF (Amigo), mRNA.
scl0320365.12_9-S	34.8079	1.65	9330186A19Rik	NM_178781.2	Mus musculus RIKEN cDNA 9330186A19 gene (9330186A19Rik), mRNA.
scl36580.22.1_188-S	34.7816	1.26	Copb2	NM_015827.1	Mus musculus coatomer protein complex, subunit beta 2 (beta prime) (Copb2), mRNA.
scl16174.14.1_64-S	34.7692	1.31	BC003331	NM_145511.1	Mus musculus cDNA sequence BC003331 (BC003331), mRNA.
scl52460.11.1_30-S	34.7081	1.61	Got1	NM_010324.1	Mus musculus glutamate oxaloacetate transaminase 1, soluble (Got1), mRNA.
scl42244.15_187-S	34.6543	1.26	Aldh6a1	NM_134042.1	Mus musculus aldehyde dehydrogenase family 6, subfamily A1 (Aldh6a1), mRNA.
scl012491.1_51-S	34.6496	2.42	Cd36	NM_007643.2	Mus musculus CD36 antigen (Cd36), mRNA.
scl0075089.2_74-S	34.558	1.46	E030041M21Rik	NM_029166.1	Mus musculus RIKEN cDNA E030041M21 gene (E030041M21Rik), mRNA.
scl16803.9_203-S	34.4578	1.59	Slc40a1	NM_016917.1	Mus musculus solute carrier family 40 (iron-regulated transporter), member 1 (Slc40a1), mRNA.
scl0016158.2_239-S	34.4276	1.54	Il11ra2	NM_010550	Mus musculus interleukin 11 receptor, alpha chain 2 (Il11ra2), mRNA.
scl0107094.1_210-S	34.4163	1.48	AA408556	NM_199447.2	Mus musculus expressed sequence AA408556 (AA408556), mRNA.
scl23010.9_157-S	34.2961	1.46	2610029K21Rik	NM_025663.2	Mus musculus RIKEN cDNA 2610029K21 gene (2610029K21Rik), mRNA.
scl26218.7.1_61-S	34.2812	2.42	AW049829	NM_153571.1	Mus musculus expressed sequence AW049829 (AW049829), mRNA.
scl074522.7_319-S	34.2256	1.51	Zcwcc1	NM_198162.1	Mus musculus zinc finger, CW-type with coiled-coil domain 1 (Zcwcc1), mRNA.
scl0004118.1_555-S	34.2006	2.82	Rpo1-3	NM_181730.2	Mus musculus RNA polymerase 1-3 (Rpo1-3), mRNA.
scl20124.13_167-S	34.1902	2.19	Csnk2a1	NM_007788.2	Mus musculus casein kinase II, alpha 1 polypeptide (Csnk2a1), mRNA.
scl0001148.1_50-S	34.1398	1.42	Ing4	NM_133345.1	Mus musculus inhibitor of growth family, member 4 (Ing4), mRNA.
scl29632.2_358-S	34.1332	1.64	5830427H10Rik	NM_026365.1	Mus musculus RIKEN cDNA 5830427H10 gene (5830427H10Rik), mRNA.
scl024004.2_257-S	34.0807	1.73	Rai2	NM_198409.1	Mus musculus retinoic acid induced 2 (Rai2), mRNA.
scl38284.14_73-S	34.0705	1.26	Cs	NM_026444.2	Mus musculus citrate synthase (Cs), mRNA.
scl21098.22.1_27-S	34.0548	1.99	Pkn3	NM_153805.1	Mus musculus protein kinase N3 (Pkn3), mRNA.
scl25133.32.1_60-S	34.0526	1.34	Nrd1	NM_146150.2	Mus musculus nardilysin, N-arginine dibasic convertase, NRD convertase 1 (Nrd1), mRNA.
scl27950.14.1_172-S	34.0195	1.35	Slc4a1ap	NM_009206.1	Mus musculus solute carrier family 4 (anion exchanger), member 1, adaptor protein (Slc4a1ap), mRNA.
scl36833.9.1_209-S	33.9736	1.76	Rpl4	NM_024212.2	Mus musculus ribosomal protein L4 (Rpl4), mRNA.
scl29872.10.1_133-S	33.973	2.26	Suclg1	NM_019879.1	Mus musculus succinate-CoA ligase, GDP-forming, alpha subunit (Suclg1), mRNA.
scl000031.1_6-S	33.953	2.66	Eif3s1	NM_144545	Mus musculus eukaryotic translation initiation factor 3, subunit 1 alpha (Eif3s1), mRNA.
scl46589.23.1_15-S	33.878	2.11	2310021P13Rik	XM_127591.4	
scl0067238.2_233-S	33.8678	1.45	2810453I06Rik	NM_026050.1	Mus musculus RIKEN cDNA 2810453I06 gene (2810453I06Rik), mRNA.
scl0020747.2_286-S	33.8139	1.25	Spop	NM_025287.1	Mus musculus speckle-type POZ protein (Spop), mRNA.
scl38694.11.1_0-S	33.7703	1.32	Stk11	NM_011492.1	Mus musculus serine/threonine kinase 11 (Stk11), mRNA.

scl066407.8_34-S	33.7496	1.45	Mrps15		
scl30657.16.1_159-S	33.7343	1.38	Mvp	NM_080638.1	Mus musculus major vault protein (Mvp), mRNA.
scl0100732.7_1-S	33.7203	2.62	Mapre3	NM_133350.1	Mus musculus microtubule-associated protein, RP/EB family, member 3 (Mapre3), mRNA.
scl066114.1_221-S	33.708	1.55	Wbscr18	NM_025362.2	Mus musculus Williams-Beuren syndrome chromosome region 18 homolog (human) (Wbscr18), mRNA.
scl067267.4_0-S	33.6756	1.29	2900010M23Rik	NM_026063.1	Mus musculus RIKEN cDNA 2900010M23 gene (2900010M23Rik), mRNA.
scl31893.12.1_41-S	33.6632	4.41	5730427C23Rik	NM_153777.1	Mus musculus RIKEN cDNA 5730427C23 gene (5730427C23Rik), mRNA.
scl0217069.13_16-S	33.6226	1.4	Trim25	XM_126545.2	Mus musculus tripartite motif protein 25 (Trim25), mRNA.
scl0216705.9_38-S	33.4461	1.87	AI642036	XM_109767.2	Mus musculus expressed sequence AI642036 (AI642036), mRNA.
scl0235493.12_117-S	33.3558	1.75	BC031353	NM_153584.1	Mus musculus cDNA sequence BC031353 (BC031353), mRNA.
scl16472.29.1_1-S	33.3428	2.03	4932408F19Rik	NM_207225.1	Mus musculus RIKEN cDNA 4932408F19 gene (4932408F19Rik), mRNA.
scl0223773.9_321-S	33.3342	1.64	Zbed4	NM_181412.2	
scl017936.1_8-S	33.2559	5.01	Nab1	NM_008667.2	Mus musculus Ngfi-A binding protein 1 (Nab1), mRNA.
scl011702.3_204-S	33.2516	1.75	Amd1	NM_009665.2	Mus musculus S-adenosylmethionine decarboxylase 1 (Amd1), mRNA.
scl45529.12.1_2-S	33.2296	2.13	Rabggta	NM_019519.1	Mus musculus Rab geranylgeranyl transferase, a subunit (Rabggta), mRNA.
scl0056516.1_106-S	33.1885	1.69	Rbms2	NM_019711.1	Mus musculus RNA binding motif, single stranded interacting protein 2 (Rbms2), mRNA.
scl0237082.4_163-S	33.1498	1.4	Nxt2	NM_172782.1	
scl0001502.1_0-S	33.0622	1.97	Hspa4	NM_008300.2	Mus musculus heat shock protein 4 (Hspa4), mRNA.
scl014862.2_242-S	33.0541	1.33	Gstm1	NM_010358.2	Mus musculus glutathione S-transferase, mu 1 (Gstm1), mRNA.
scl067905.1_18-S	32.9998	1.37	2810423O19Rik	NM_026447	Mus musculus RIKEN cDNA 2810423O19 gene (2810423O19Rik), transcript variant 1, mRNA.
scl00192657.1_320-S	32.8981	3.7	Ell2	NM_138953.1	Mus musculus elongation factor RNA polymerase II 2 (Ell2), mRNA.
scl23969.12.1_3-S	32.8933	1.56	Cyp4b1	NM_007823.1	Mus musculus cytochrome P450, family 4, subfamily b, polypeptide 1 (Cyp4b1), mRNA.
scl53966.33.1_30-S	32.8714	2.71	Phka1	NM_008832.1	Mus musculus phosphorylase kinase alpha 1 (Phka1), mRNA.
scl0209497.2_5-S	32.8502	2.28	AW547186	NM_177592.2	Mus musculus expressed sequence AW547186 (AW547186), mRNA.
scl0002552.1_594-S	32.8309	1.59	Triobp	NM_138579.2	Mus musculus TRIO and F-actin binding protein (Triobp), mRNA.
scl32033.10.26_108-S	32.822	1.44	BC006909	NM_145589.2	Mus musculus cDNA sequence BC006909 (BC006909), mRNA.
scl068114.7_83-S	32.8201	1.3	Mum1	NM_023431.3	
scl066943.7_225-S	32.7933	1.29	2310009N05Rik	NM_025861.1	Mus musculus RIKEN cDNA 2310009N05 gene (2310009N05Rik), mRNA.
scl0050908.1_300-S	32.75	3.44	C1s	NM_144938.1	Mus musculus complement component 1, s subcomponent (C1s), mRNA.
scl22111.1_41-S	32.727	7.52	4631416L12Rik	NM_175365.2	Mus musculus RIKEN cDNA 4631416L12 gene (4631416L12Rik), mRNA.
scl018023.1_196-S	32.7231	1.25	Nfe2l1	NM_008686.2	Mus musculus nuclear factor, erythroid derived 2,-like 1 (Nfe2l1), mRNA.
scl00214290.1_251-S	32.6806	1.31	Zcchc6	NM_153538.1	Mus musculus zinc finger, CCHC domain containing 6 (Zcchc6), mRNA.
scl0069654.1_290-S	32.6581	1.53	Dctn2	NM_027151.1	Mus musculus dynactin 2 (Dctn2), mRNA.
scl00231841.2_319-S	32.6374	1.45	AA881470	NM_181066.1	Mus musculus EST AA881470 (AA881470), mRNA.
scl053610.12_290-S	32.6096	1.64	Nono	NM_023144.1	Mus musculus non-POU-domain-containing, octamer binding protein (Nono), mRNA.
scl011787.1_19-S	32.5821	1.48	Apbb2	NM_009686.1	Mus musculus amyloid beta (A4) precursor protein-binding, family B, member 2 (Apbb2), mRNA.
scl15987.1.1_285-S	32.5739	1.33	AI481316	XM_148986.1	Mus musculus expressed sequence AI481316 (AI481316), mRNA.
scl070221.8_1-S	32.5375	7.07	2600011C06Rik	NM_027349.1	Mus musculus RIKEN cDNA 2600011C06 gene (2600011C06Rik), mRNA.
scl056708.1_17-S	32.5185	7.3	Bsf3	NM_019952.1	Mus musculus B-cell stimulating factor 3 (Bsf3), mRNA.
scl26445.6.1_1-S	32.4872	1.28	Igfbp7	NM_008048.1	Mus musculus insulin-like growth factor binding protein 7 (Igfbp7), mRNA.
scl54486.8.1_241-S	32.4191	1.79	Asb11	NM_026853.1	Mus musculus ankyrin repeat and SOCS box-containing protein 11 (Asb11), mRNA.
scl0002359.1_0-S	32.4082	2.18	Pbef1	NM_021524.1	Mus musculus pre-B-cell colony-enhancing factor 1 (Pbef1), mRNA.
scl38416.13.1_1-S	32.3255	1.68	Ptprb	NM_029928.1	Mus musculus protein tyrosine phosphatase, receptor type, B (Ptprb), mRNA.
scl0020923.1_202-S	32.2869	1.29	Supt4h2	NM_011509	Mus musculus suppressor of Ty 4 homolog 2 (S. cerevisiae) (Supt4h2), mRNA.
scl0269523.2_3-S	32.2778	1.24	Vcp	NM_009503.2	Mus musculus valosin containing protein (Vcp), mRNA.
scl0012453.1_227-S	32.2266	3.11	Ccni	NM_017367.2	Mus musculus cyclin I (Ccni), mRNA.
scl0054613.2_126-S	32.2192	1.33	Siat10	NM_018784.1	Mus musculus sialyltransferase 10 (alpha-2,3-sialyltransferase VI) (Siat10), mRNA.
scl0237422.9_211-S	32.218	1.28	BC051080	NM_183172.1	
scl34554.11.1_42-S	32.1752	2.95	Gcdh	NM_008097.1	Mus musculus glutaryl-Coenzyme A dehydrogenase (Gcdh), mRNA.
scl00217430.2_0-S	32.1742	1.54	E030024M05Rik	NM_172574.1	Mus musculus RIKEN cDNA E030024M05 gene (E030024M05Rik), mRNA.
scl18197.5_22-S	32.1519	1.81	Rgs19	NM_026446.2	Mus musculus regulator of G-protein signaling 19 (Rgs19), mRNA.
scl0116904.18_144-S	32.1502	1.31	AW319487	NM_054085.1	Mus musculus expressed sequence AW319487 (AW319487), mRNA.
scl026895.7_143-S	32.1439	2.74	Cops7b	NM_172974.1	Mus musculus COP9 (constitutive photomorphogenic) homolog, subunit 7b (Arabidopsis thaliana) (Cops7b), mRNA.
scl0029809.2_171-S	32.0603	1.7	Rabgap1l	NM_013862.3	
scl012457.3_248-S	32.0107	2	Ccrn4l	NM_009834.1	Mus musculus CCR4 carbon catabolite repression 4-like (S. cerevisiae) (Ccrn4l), mRNA.
scl0015183.2_163-S	32.0058	1.28	Hdac3	NM_010411.1	Mus musculus histone deacetylase 3 (Hdac3), mRNA.
scl16121.8.1_163-S	31.9836	1.36	BC034090	XM_148974.3	Mus musculus cDNA sequence BC034090 (BC034090), mRNA.

scl0387340.1_274-S	31.9712	6.97	Tas2r104	NM_207011.1	Mus musculus taste receptor, type 2, member 104 (Tas2r104), mRNA.
scl37604.17_345-S	31.9704	1.53	Nedd1	NM_008682	Mus musculus neural precursor cell expressed, developmentally down-regulated gene 1 (Nedd1), mRNA.
scl51006.12.1_70-S	31.9545	1.28	Rpl3l	NM_025425.1	Mus musculus ribosomal protein L3-like (Rpl3l), mRNA.
scl0001099.1_185-S	31.9202	2.37	St7	NM_022332.1	Mus musculus suppression of tumorigenicity 7 (St7), mRNA.
scl40718.3_48-S	31.9194	2.7	2210020M01Rik	NM_183259.1	Mus musculus RIKEN cDNA 2210020M01 gene (2210020M01Rik), mRNA.
scl0017755.1_176-S	31.9083	7.07	Mtap1b	NM_008634.1	Mus musculus microtubule-associated protein 1 B (Mtap1b), mRNA.
scl0023821.2_49-S	31.892	1.31	Bace1	NM_011792.3	Mus musculus beta-site APP cleaving enzyme 1 (Bace1), mRNA.
scl068730.1_55-S	31.8834	1.39	1110032N12Rik	NM_026824.2	Mus musculus RIKEN cDNA 1110032N12 gene (1110032N12Rik), mRNA.
scl33707.42_67-S	31.8828	1.52	Myo9b	NM_015742.1	Mus musculus myosin IXb (Myo9b), mRNA.
scl0029869.2_146-S	31.8742	1.41	Ulk2	NM_013881.3	Mus musculus Unc-51 like kinase 2 (C. elegans) (Ulk2), mRNA.
scl18742.7_10-S	31.8533	1.39	Slc30a4	NM_011774	Mus musculus solute carrier family 30 (zinc transporter), member 4 (Slc30a4), mRNA.
scl33602.12.1_74-S	31.8518	2.21	Ddx39	NM_197982.2	Mus musculus DEAD (Asp-Glu-Ala-Asp) box polypeptide 39 (Ddx39), mRNA.
scl068735.6_63-S	31.8078	1.25	Mrps18c	XM_194230.3	
scl011430.3_98-S	31.8011	2.48	Acox1	NM_015729	Mus musculus acyl-Coenzyme A oxidase 1, palmitoyl (Acox1), mRNA.
scl070478.15_14-S	31.772	1.39	Mipep	NM_027436.1	Mus musculus mitochondrial intermediate peptidase (Mipep), mRNA.
scl33398.3_321-S	31.7719	1.54	AW539964	NM_173432.2	Mus musculus expressed sequence AW539964 (AW539964), mRNA.
scl38585.9.1_9-S	31.756	1.94	2900091E11Rik	NM_026070.1	Mus musculus RIKEN cDNA 2900091E11 gene (2900091E11Rik), mRNA.
scl019946.1_13-S	31.7534	1.53	Rpl30	NM_009083.2	Mus musculus ribosomal protein L30 (Rpl30), mRNA.
scl0001538.1_0-S	31.7486	5.34	Cacnb1	NM_031173.1	Mus musculus calcium channel, voltage-dependent, beta 1 subunit (Cacnb1), mRNA.
scl0020947.1_79-S	31.711	1.43	Swap70	NM_009302.2	Mus musculus SWAP complex protein (Swap70), mRNA.
scl42839.5.1_76-S	31.7008	7.11	Serpina5	NM_172953.1	Mus musculus serine (or cysteine) proteinase inhibitor, clade A, member 5 (Serpina5), mRNA.
scl38270.7_86-S	31.6468	3.79	Dnajc14	NM_028873.2	
scl018458.3_17-S	31.6402	1.4	Pabpc1	NM_008774.2	Mus musculus poly A binding protein, cytoplasmic 1 (Pabpc1), mRNA.
scl21064.31_2-S	31.614	1.3	5830434P21Rik	NM_172661.2	Mus musculus RIKEN cDNA 5830434P21 gene (5830434P21Rik), mRNA.
scl072795.9_17-S	31.5939	1.26	Ttc19	NM_028360.1	
scl31512.7.1_16-S	31.5471	1.49	5033425B17Rik	NM_027215	Mus musculus RIKEN cDNA 5033425B17 gene (5033425B17Rik), mRNA.
scl0227570.7_253-S	31.4709	1.49	2810455F06Rik	XM_130011.5	Mus musculus RIKEN cDNA 2810455F06 gene (2810455F06Rik), mRNA.
scl49475.21_477-S	31.4624	1.35	Mgrn1	NM_029657.2	Mus musculus mahogunin, ring finger 1 (Mgrn1), mRNA.
scl35001.15_103-S	31.4505	1.28	Plekha2	NM_031257.2	Mus musculus pleckstrin homology domain-containing, family A (phosphoinositide binding specific) member 2 (Plekha2), mRNA.
scl26595.14.1_90-S	31.4295	2.04	Ppargc1a	NM_008904.1	Mus musculus peroxisome proliferative activated receptor, gamma, coactivator 1 alpha (Ppargc1a), mRNA.
scl017449.1_1-S	31.3768	2.42	Mdh1	NM_008618.2	Mus musculus malate dehydrogenase 1, NAD (soluble) (Mdh1), mRNA.
scl0215193.1_66-S	31.3554	2.51	AA408296	NM_145415.1	Mus musculus expressed sequence AA408296 (AA408296), mRNA.
scl27876.6.1_53-S	31.2349	1.39	2810021O14Rik	NM_025480.2	Mus musculus RIKEN cDNA 2810021O14 gene (2810021O14Rik), mRNA.
scl0216622.2_35-S	31.2114	1.7	4931440F15Rik	NM_176829.1	Mus musculus RIKEN cDNA 4931440F15 gene (4931440F15Rik), mRNA.
scl37393.11_210-S	31.1946	1.32	Pip5k2c	NM_054097.2	Mus musculus phosphatidylinositol-4-phosphate 5-kinase, type II, gamma (Pip5k2c), mRNA.
scl0080708.1_141-S	31.1924	1.25	Pacsin3	NM_028733.1	Mus musculus protein kinase C and casein kinase substrate in neurons 3 (Pacsin3), mRNA.
scl42729.13.1_74-S	31.1536	1.35	Adssl1	NM_007421.1	
scl0211446.1_21-S	31.1218	1.37	Sec6l1	NM_177333	Mus musculus SEC6-like 1 (S. cerevisiae) (Sec6l1), mRNA.
scl00235534.1_198-S	31.0959	1.47	C130099A20Rik	NM_153420.1	
scl50778.12.1_32-S	31.0688	1.26	Bat1a	NM_019693.2	Mus musculus HLA-B-associated transcript 1A (Bat1a), mRNA.
scl51773.17.1_8-S	31.0517	2.95	Mbd1	NM_013594.1	Mus musculus methyl-CpG binding domain protein 1 (Mbd1), mRNA.
scl21832.19.384_11-S	30.9645	1.41	Tsrc1	NM_144899.2	Mus musculus thrombospondin repeat containing 1 (Tsrc1), mRNA.
scl026901.3_41-S	30.9497	1.35	Deb1	NM_026794.2	Mus musculus differentially expressed in B16F10 1 (Deb1), mRNA.
scl0058909.2_289-S	30.9482	1.69	D430015B01Rik	NM_153574.1	Mus musculus RIKEN cDNA D430015B01 gene (D430015B01Rik), mRNA.
scl00229949.2_260-S	30.9117	7.01	Ak5	NM_153066.1	Mus musculus adenylate kinase 5 (Ak5), mRNA.
scl066369.15_25-S	30.9043	2.85	2310016K04Rik	XM_134309.1	Mus musculus RIKEN cDNA 2310016K04 gene (2310016K04Rik), mRNA.
scl0102143.1_151-S	30.8846	1.3	D130072O21Rik	NM_175322.2	Mus musculus RIKEN cDNA D130072O21 gene (D130072O21Rik), mRNA.
scl44745.15.1_5-S	30.8685	6.9	Unc5a	NM_153131.1	Mus musculus unc-5 homolog A (C. elegans) (Unc5a), mRNA.
scl0001814.1_15-S	30.8669	1.28	Magmas	NM_025571.1	Mus musculus mitochondria-associated protein involved in granulocyte-macrophage colony-stimulating factor signal transduction (Magmas), mRNA.
scl33380.18.1_13-S	30.8558	1.55	Tex292	NM_011574.1	Mus musculus testis expressed gene 292 (Tex292), mRNA.
scl49774.8.1_3-S	30.8155	1.62	2310076L09Rik	NM_025874.2	Mus musculus RIKEN cDNA 2310076L09 gene (2310076L09Rik), mRNA.
scl0067184.2_306-S	30.771	1.41	Grim19	NM_023312.1	Mus musculus genes associated with retinoid-IFN-induced mortality 19 (Grim19), mRNA.
scl44342.6.5_4-S	30.7706	1.37	Mocs2	NM_013826.1	Mus musculus molybdenum cofactor synthesis 2 (Mocs2), mRNA.
scl28496.23.1_78-S	30.7556	1.82	Bms1l	NM_194339.1	Mus musculus BMS1-like, ribosome assembly protein (yeast) (Bms1l), mRNA.
scl012608.3_48-S	30.7532	2.82	Cebpb	NM_009883.1	Mus musculus CCAAT/enhancer binding protein (C/EBP), beta (Cebpb), mRNA.
scl25760.31.1_106-S	30.707	1.46	Flt1	NM_010228.2	Mus musculus FMS-like tyrosine kinase 1 (Flt1), mRNA.

scl0240263.2_0-S	30.6953	2	Fem1c	NM_173423.1	Mus musculus fem-1 homolog c (C.elegans) (Fem1c), mRNA.
scl0072472.2_14-S	30.667	1.83	Slc16a10	NM_028247.1	Mus musculus solute carrier family 16 (monocarboxylic acid transporters), member 10 (Slc16a10), mRNA.
scl32661.6_250-S	30.6491	2.71	Kdelr1	NM_133950	Mus musculus KDEL (Lys-Asp-Glu-Leu) endoplasmic reticulum protein retention receptor 1 (Kdelr1), mRNA.
scl0215015.1_6-S	30.6426	1.54	C530043G21Rik	NM_145413.2	Mus musculus RIKEN cDNA C530043G21 gene (C530043G21Rik), mRNA.
scl34621.8_577-S	30.632	1.34	Ednra	NM_010332.1	Mus musculus endothelin receptor type A (Ednra), mRNA.
scl45372.3_35-S	30.5826	2.73	Mscp	NM_030054.2	Mus musculus mitochondrial solute carrier protein (Mscp), mRNA.
scl0003299.1_11-S	30.5806	1.98	2810410A08Rik	XM_130324.4	Mus musculus RIKEN cDNA 2810410A08 gene (2810410A08Rik), mRNA.
scl056258.4_7-S	30.5796	2.86	Hnrph2	NM_019868.2	Mus musculus heterogeneous nuclear ribonucleoprotein H2 (Hnrph2), mRNA.
scl0108888.1_320-S	30.5743	1.42	Atad3a	NM_179203.1	Mus musculus ATPase family, AAA domain containing 3A (Atad3a), mRNA.
scl41644.4_98-S	30.5648	1.36	Crsp9	NM_025426.2	Mus musculus cofactor required for Sp1 transcriptional activation, subunit 9 (Crsp9), mRNA.
scl36808.5_96-S	30.5646	1.54	Pdcd7	NM_016688.1	Mus musculus programmed cell death protein 7 (Pdcd7), mRNA.
scl26220.57_399-S	30.5478	1.57	Ep400	NM_029337.1	Mus musculus E1A binding protein p400 (Ep400), mRNA.
scl40878.26_32-S	30.5467	1.42	Nbr1	NM_008676.1	Mus musculus neighbor of Brca1 gene 1 (Nbr1), mRNA.
scl00140740.1_195-S	30.5451	1.39	Sec63	NM_153055.2	Mus musculus SEC63-like (S. cerevisiae) (Sec63), mRNA.
scl00192197.1_323-S	30.5223	1.62	Bcas3	NM_138681.2	Mus musculus breast carcinoma amplified sequence 3 (Bcas3), mRNA.
scl093684.5_156-S	30.4843	1.74	39340	NM_053102.1	Mus musculus selenoprotein (Sep15), mRNA.
scl0001327.1_163-S	30.4824	2.12	Trim41	NM_145377	Mus musculus tripartite motif-containing 41 (Trim41), mRNA.
scl068058.2_29-S	30.4735	1.54	Chd1l	NM_026539.1	Mus musculus chromodomain helicase DNA binding protein 1-like (Chd1l), mRNA.
scl41617.26_170-S	30.4166	2.15	2700008N14Rik	XM_109880.4	Mus musculus RIKEN cDNA 2700008N14 gene (2700008N14Rik), mRNA.
scl0067464.2_263-S	30.4154	1.36	Entpd4	NM_026174.1	
scl54760.5_0-S	30.4085	2.28	Efnb1	NM_010110.2	Mus musculus ephrin B1 (Efnb1), mRNA.
scl45065.22.7_66-S	30.4059	1.46	B130016L12Rik	NM_144835.3	
scl0076367.2_3-S	30.3998	5.55	Trp53rk	NM_023815.2	
scl20678.2.192_93-S	30.3694	6.37	Olfr996	NM_146437.1	Mus musculus olfactory receptor 996 (Olfr996), mRNA.
scl37933.6_401-S	30.3606	1.5	D10Ertd641e	NM_025514.1	Mus musculus DNA segment, Chr 10, ERATO Doi 641, expressed (D10Ertd641e), mRNA.
scl019130.1_4-S	30.3428	1.57	Prox1	NM_008937.2	Mus musculus prospero-related homeobox 1 (Prox1), mRNA.
scl0083964.2_193-S	30.3358	1.47	Jam3	NM_023277.1	Mus musculus junction adhesion molecule 3 (Jam3), mRNA.
scl018744.1_4-S	30.2537	1.3	Pja1	NM_008853.1	Mus musculus praja1, RING-H2 motif containing (Pja1), mRNA.
scl40219.8.1_51-S	30.2506	1.34	Pdlim4	NM_019417.1	Mus musculus PDZ and LIM domain 4 (Pdlim4), mRNA.
scl36930.15.1_0-S	30.2387	1.49	3010021M21Rik	NM_180600.1	Mus musculus RIKEN cDNA 3010021M21 gene (3010021M21Rik), mRNA.
scl011363.1_35-S	30.2021	2.49	Acadl	NM_007381.2	Mus musculus acetyl-Coenzyme A dehydrogenase, long-chain (Acadl), mRNA.
scl31989.25.1_124-S	30.2014	1.84	Tacc2	NM_206856.1	Mus musculus transforming, acidic coiled-coil containing protein 2 (Tacc2), transcript variant 1, mRNA.
scl026446.4_108-S	30.1713	1.23	Psmb3	NM_011971.2	Mus musculus proteasome (prosome, macropain) subunit, beta type 3 (Psmb3), mRNA.
scl33146.4.49_0-S	30.115	1.35	Ndufa3	NM_025348	Mus musculus NADH dehydrogenase (ubiquinone) 1 alpha subcomplex, 3 (Ndufa3), mRNA.
scl18978.15.15_4-S	30.0755	3.09	Ext2	NM_010163	Mus musculus exostoses (multiple) 2 (Ext2), mRNA.
scl0001672.1_92-S	30.0654	3.91	Phf1	NM_009343.1	Mus musculus PHD finger protein 1 (Phf1), mRNA.
scl00107371.2_186-S	29.9771	1.39	Sec15l1	NM_175353.1	
scl18763.35.1_291-S	29.97	1.61	B430315C20Rik	NM_178795.3	Mus musculus RIKEN cDNA B430315C20 gene (B430315C20Rik), mRNA.
scl073998.24_21-S	29.9692	1.33	Herc3	NM_028705.2	Mus musculus hect domain and RLD 3 (Herc3), mRNA.
scl49330.22.4_270-S	29.9427	3.02	Chrd	NM_009893.1	Mus musculus chordin (Chrd), mRNA.
scl33725.12_115-S	29.9388	1.39	Fkbp8	NM_010223.1	Mus musculus FK506 binding protein 8 (Fkbp8), mRNA.
scl0003137.1_53-S	29.9128	1.32	Drbp1	NM_178090.2	Mus musculus developmentally regulated RNA binding protein 1 (Drbp1), mRNA.
scl34841.3_211-S	29.8673	2.57	4921511I16Rik	NM_172407.1	Mus musculus RIKEN cDNA 4921511I16 gene (4921511I16Rik), mRNA.
scl30532.13.1_156-S	29.7172	4.25	Stk32c	NM_021302.1	
scl0002044.1_26-S	29.713	1.89	Alg5	NM_025442.1	Mus musculus asparagine-linked glycosylation 5 homolog (yeast, dolichyl-phosphate beta-glucosyltransferase) (Alg5), mRNA.
scl23666.9_53-S	29.6899	1.25	Lypla2	NM_011942.1	Mus musculus lysophospholipase 2 (Lypla2), mRNA.
scl37440.14_650-S	29.628	1.77	Irak3	NM_028679.2	Mus musculus interleukin-1 receptor-associated kinase 3 (Irak3), mRNA.
scl00104318.1_298-S	29.5963	1.56	Csnk1d	NM_027874.1	Mus musculus casein kinase 1, delta (Csnk1d), transcript variant 2, mRNA.
scl38337.8_34-S	29.5845	4.06	Ctdsp2	NM_146012.1	Mus musculus CTD (carboxy-terminal domain, RNA polymerase II, polypeptide A) small phosphatase 2 (Ctdsp2), mRNA.
scl40305.32_46-S	29.5625	1.82	Cyfip2	XM_147526.1	Mus musculus cytoplasmic FMR1 interacting protein 2 (Cyfip2), mRNA.
scl0013669.1_3-S	29.555	1.42	Eif3s10	NM_010123.2	Mus musculus eukaryotic translation initiation factor 3, subunit 10 (theta) (Eif3s10), mRNA.
scl00224727.2_315-S	29.5399	1.33	Bat3	NM_057171.1	Mus musculus HLA-B-associated transcript 3 (Bat3), mRNA.
scl078090.2_4-S	29.4702	4.04	Golgb1	XM_148244.4	
scl057435.1_222-S	29.4516	1.9	S3-12	NM_020568.1	Mus musculus plasma membrane associated protein, S3-12 (S3-12), mRNA.
scl47016.7_165-S	29.4188	6.13	9130022K13Rik	XM_203652.2	Mus musculus RIKEN cDNA 9130022K13 gene (9130022K13Rik), mRNA.
scl011532.9_123-S	29.403	1.57	Adh5	NM_007410.2	Mus musculus alcohol dehydrogenase 5 (class III), chi polypeptide (Adh5), mRNA.

scl28314.7.1_50-S	29.395	1.4	2010012C16Rik	NM_025564.1	Mus musculus RIKEN cDNA 2010012C16 gene (2010012C16Rik), mRNA.
scl41618.6.1_22-S	29.3891	1.3	Rnf130	NM_021540.2	Mus musculus ring finger protein 130 (Rnf130), mRNA.
scl080751.6_5-S	29.3852	1.26	Rnf34	NM_030564.1	Mus musculus ring finger protein 34 (Rnf34), mRNA.
scl00234733.1_149-S	29.3758	4.06	2810457M08Rik	NM_172284.1	Mus musculus RIKEN cDNA 2810457M08 gene (2810457M08Rik), mRNA.
GI_31982339-S	29.3532	5.21	Gip	NM_008119.2	Mus musculus gastric inhibitory polypeptide (Gip), mRNA.
scl018550.2_67-S	29.3481	3.93	Furin	NM_011046.1	Mus musculus furin (paired basic amino acid cleaving enzyme) (Furin), mRNA.
scl25081.14.1_17-S	29.3474	1.53	D630045E04Rik	NM_153521.1	Mus musculus RIKEN cDNA D630045E04 gene (D630045E04Rik), mRNA.
scl20653.2_227-S	29.3098	1.8	C1qtnf4	NM_026161.1	Mus musculus C1q and tumor necrosis factor related protein 4 (C1qtnf4), mRNA.
scl20297.9_280-S	29.2823	1.28	Ptpns1	XM_149178.1	Mus musculus protein tyrosine phosphatase, non-receptor type substrate 1 (Ptpns1), mRNA.
scl067455.1_261-S	29.2756	1.55	Klhl13	NM_026167.1	Mus musculus kelch-like 13 (Drosophila) (Klhl13), mRNA.
scl24307.19_191-S	29.2687	1.66	Catnal1	NM_018761.2	Mus musculus catenin alpha-like 1 (Catnal1), mRNA.
scl0002099.1_352-S	29.2647	1.6	Pias3	NM_146135.1	Mus musculus protein inhibitor of activated STAT 3 (Pias3), transcript variant 1, mRNA.
scl0020479.2_290-S	29.2579	1.64	Vps4b	NM_009190.1	Mus musculus vacuolar protein sorting 4b (yeast) (Vps4b), mRNA.
scl078653.2_11-S	29.2418	1.23	1810056O20Rik	NM_175277.2	Mus musculus RIKEN cDNA 1810056O20 gene (1810056O20Rik), mRNA.
scl41358.1_245-S	29.2103	1.37	1810027O10Rik	XM_109683.4	Mus musculus RIKEN cDNA 1810027O10 gene (1810027O10Rik), mRNA.
scl47390.18.104_38-S	29.2068	1.82	Tars	NM_033074.2	Mus musculus threonyl-tRNA synthetase (Tars), mRNA.
scl22117.3_138-S	29.1556	5.5	Igsf10	XM_143254.3	
scl0068310.2_45-S	29.1246	2.5	Zmym1	NM_026670.2	Mus musculus zinc finger, MYM domain containing 1 (Zmym1), mRNA.
scl22773.20.1_35-S	29.109	2.27	Phtf1	XM_356222.1	Mus musculus putative homeodomain transcription factor 1 (Phtf1), mRNA.
scl47045.25.1_21-S	29.0404	1.64	Oplah	NM_153122.1	Mus musculus 5-oxoprolinase (ATP-hydrolysing) (Oplah), mRNA.
scl31414.28.1_4-S	29.0287	1.82	Mybpc2	NM_146189.1	Mus musculus myosin binding protein C, fast-type (Mybpc2), mRNA.
scl0020491.2_142-S	29.0242	4.71	Sla	NM_009192.1	Mus musculus src-like adaptor (Sla), mRNA.
scl22977.3.91_193-S	29.0092	1.38	Dpm3	XM_130951.1	Mus musculus dolichyl-phosphate mannosyltransferase polypeptide 3 (Dpm3), mRNA.
scl31487.7.1_48-S	28.9859	1.3	6030457N17Rik	XM_133378.3	Mus musculus RIKEN cDNA 6030457N17 gene (6030457N17Rik), mRNA.
scl0001346.1_38-S	28.9703	8.05	Sphk1	NM_011451.1	Mus musculus sphingosine kinase 1 (Sphk1), mRNA.
scl00215751.1_299-S	28.9131	1.25	BC013529	NM_145418.1	Mus musculus cDNA sequence BC013529 (BC013529), mRNA.
scl54755.10_245-S	28.8883	4.01	Eda	NM_010099.1	Mus musculus ectodysplasin-A (Eda), mRNA.
scl026912.9_26-S	28.879	1.59	Gcat	NM_013847	Mus musculus glycine C-acetyltransferase (2-amino-3-ketobutyrate-coenzyme A ligase) (Gcat), mRNA.
scl0002340.1_98-S	28.8461	1.82	Nin	NM_008697	Mus musculus ninein (Nin), mRNA.
scl35165.21_648-S	28.8405	1.23	Fyco1	NM_148925.1	Mus musculus FYVE and coiled-coil domain containing 1 (Fyco1), mRNA.
scl25868.6.1_19-S	28.8333	1.63	D5Wsu46e	NM_144913.2	Mus musculus DNA segment, Chr 5, Wayne State University 46, expressed (D5Wsu46e), mRNA.
scl0003483.1_26-S	28.7804	7.17	2210413P10Rik	NM_020518.1	Mus musculus RIKEN cDNA 2210413P10 gene (2210413P10Rik), mRNA.
scl0216792.1_314-S	28.7794	1.91	A230051G13Rik	NM_173785.2	
scl34640.17_455-S	28.7763	1.26	Large	NM_010687.1	Mus musculus like-glycosyltransferase (Large), mRNA.
scl0234385.1_330-S	28.7734	1.88	Mast3	NM_199308.1	
scl41592.12.1_36-S	28.7445	1.4	2900006B13Rik	NM_028398.1	Mus musculus RIKEN cDNA 2900006B13 gene (2900006B13Rik), mRNA.
scl55017.16.1_2-S	28.7013	1.7	Araf1	NM_009703.1	
scl42976.13.1_6-S	28.6977	1.37	Coq6	NM_172582.1	Mus musculus coenzyme Q6 homolog (yeast) (Coq6), mRNA.
scl31001.17.1_88-S	28.6188	1.47	Slco2b1	NM_175316.2	Mus musculus solute carrier organic anion transporter family, member 2b1 (Slco2b1), mRNA.
scl39534.12.3_104-S	28.6154	1.38	2310044P18Rik	NM_144829.1	Mus musculus RIKEN cDNA 2310044P18 gene (2310044P18Rik), mRNA.
scl38024.23.1_253-S	28.5615	1.42	A530089I17Rik	NM_133999.1	Mus musculus RIKEN cDNA A530089I17 gene (A530089I17Rik), mRNA.
scl0056550.1_264-S	28.5545	1.33	Ube2d2	NM_019912.1	Mus musculus ubiquitin-conjugating enzyme E2D 2 (Ube2d2), mRNA.
scl0011518.2_215-S	28.5215	1.85	Add1	NM_013457.1	Mus musculus adducin 1 (alpha) (Add1), mRNA.
scl38291.1_10-S	28.5179	4.34	D10Ucla2	NM_133996.2	Mus musculus DNA segment, Chr 10, University of California at Los Angeles 2 (D10Ucla2), mRNA.
scl23470.4_177-S	28.4789	2.85	Phf13	NM_172705.1	Mus musculus PHD finger protein 13 (Phf13), mRNA.
scl44054.10_313-S	28.4591	1.4	Nedd9	NM_017464.2	Mus musculus neural precursor cell expressed, developmentally down-regulated gene 9 (Nedd9), mRNA.
scl40538.6.1_45-S	28.4519	1.27	H2afv	XM_126043.3	
scl0116701.6_71-S	28.4152	1.86	Fgfrl1	NM_054071.1	Mus musculus fibroblast growth factor receptor-like 1 (Fgfrl1), mRNA.
scl28444.18.1_1-S	28.4151	1.33	Phc1	NM_007905.1	Mus musculus polyhomeotic-like 1 (Drosophila) (Phc1), mRNA.
scl24373.4.1_116-S	28.3919	6.01	Exosc3	NM_025513.1	Mus musculus exosome component 3 (Exosc3), mRNA.
scl54116.7.1_56-S	28.3784	1.63	Mtcp1	NM_010839.2	Mus musculus mature T-cell proliferation 1 (Mtcp1), mRNA.
scl25927.13_1-S	28.3726	1.47	Pom121	NM_148932.1	Mus musculus nuclear pore membrane protein 121 (Pom121), mRNA.
scl45364.8.1_112-S	28.3706	1.44	1700020M16Rik	XM_127811.4	Mus musculus RIKEN cDNA 1700020M16 gene (1700020M16Rik), mRNA.
scl074319.4_0-S	28.3356	1.61	1110005A03Rik	NM_028865	Mus musculus RIKEN cDNA 1110005A03 gene (1110005A03Rik), mRNA.
scl00102103.1_84-S	28.2976	1.45	Mtus1	NM_178902.3	Mus musculus mitochondrial tumor suppressor 1 (Mtus1), mRNA.
scl41378.4.1_23-S	28.2262	7.08	Hes7	NM_033041	Mus musculus hairy and enhancer of split 7 (Drosophila) (Hes7), mRNA.

scl014007.1_13-S	28.2006	1.25	Cugbp2	NM_010160.1	Mus musculus CUG triplet repeat,RNA binding protein 2 (Cugbp2), mRNA.
scl0268859.12_21-S	28.1968	1.26	A2bp1	NM_021477	Mus musculus ataxin 2 binding protein 1 (A2bp1), transcript variant 2, mRNA.
scl38714.10.10_60-S	28.1705	1.58	Hcn2	NM_008226.1	Mus musculus hyperpolarization-activated, cyclic nucleotide-gated K+ 2 (Hcn2), mRNA.
scl056384.3_31-S	28.1607	1.68	Letm1	NM_019694.1	Mus musculus leucine zipper-EF-hand containing transmembrane protein 1 (Letm1), mRNA.
scl0026430.2_0-S	28.1418	1.56	Parg	NM_011960.1	Mus musculus poly (ADP-ribose) glycohydrolase (Parg), mRNA.
scl46303.7_279-S	28.1372	1.34	Lrp10	NM_022993.2	Mus musculus low-density lipoprotein receptor-related protein 10 (Lrp10), mRNA.
scl48545.9.1_240-S	28.1332	4.26	D630035O19Rik	NM_145932	Mus musculus RIKEN cDNA D630035O19 gene (D630035O19Rik), mRNA.
scl068066.1_115-S	28.1301	1.3	D11Ertd333e	NM_026542.1	
scl54562.12.1_64-S	28.119	2.43	Alas2	NM_009653.1	Mus musculus aminolevulinic acid synthase 2, erythroid (Alas2), mRNA.
scl056438.5_27-S	28.1058	1.22	Rbx1	NM_019712.2	Mus musculus ring-box 1 (Rbx1), mRNA.
scl45906.4.1_41-S	28.0882	2.64	Fhit	NM_010210.1	Mus musculus fragile histidine triad gene (Fhit), mRNA.
scl000098.1_12-S	27.991	1.38	0610007P22Rik	NM_026676.1	Mus musculus RIKEN cDNA 0610007P22 gene (0610007P22Rik), mRNA.
scl46082.11_140-S	27.9824	1.33	Tgfb1i4	NM_009366.1	Mus musculus transforming growth factor beta 1 induced transcript 4 (Tgfb1i4), mRNA.
scl0017168.2_280-S	27.9818	4.73	Mare	NM_181569.1	Mus musculus alpha globin regulatory element containing gene (Mare), mRNA.
scl0319721.1_96-S	27.9658	4.32	C030002J06Rik	NM_176988.1	Mus musculus RIKEN cDNA C030002J06 gene (C030002J06Rik), mRNA.
scl22134.5_174-S	27.9643	1.25	Pfn2	NM_019410.2	Mus musculus profilin 2 (Pfn2), mRNA.
scl35088.19_67-S	27.9514	7.46	Tubgcp3	NM_198031.1	Mus musculus tubulin, gamma complex associated protein 3 (Tubgcp3), mRNA.
scl0002157.1_164-S	27.922	5.61	Kcnn2	NM_080465.1	Mus musculus potassium intermediate/small conductance calcium-activated channel, subfamily N, member 2 (Kcnn2), mRNA.
scl070767.1_256-S	27.907	1.94	Prpf3	NM_027541.2	Mus musculus PRP3 pre-mRNA processing factor 3 homolog (yeast) (Prpf3), mRNA.
scl33986.7.1_21-S	27.8997	1.3	Ank1	NM_031158.1	Mus musculus ankyrin 1, erythroid (Ank1), mRNA.
scl067869.4_264-S	27.8945	1.46	Paip2	NM_026420.1	Mus musculus polyadenylate-binding protein-interacting protein 2 (Paip2), mRNA.
scl49386.9_715-S	27.871	1.26	Ppm1f	NM_176833.2	Mus musculus protein phosphatase 1F (PP2C domain containing) (Ppm1f), mRNA.
scl36473.24.9_6-S	27.8605	1.39	Usp4	NM_011678.1	Mus musculus ubiquitin specific protease 4 (proto-oncogene) (Usp4), mRNA.
scl0110323.3_24-S	27.8469	1.26	Cox6b	NM_025628.1	Mus musculus cytochrome c oxidase, subunit VIb (Cox6b), mRNA.
scl47173.4.1_30-S	27.842	1.63	8230402K04Rik	NM_177755.2	
scl00231999.2_275-S	27.82	1.93	BC052360	XM_132552.3	
scl17473.2_640-S	27.8186	2.02	Lrrn2	XM_136224.2	Mus musculus leucine rich repeat protein 2, neuronal (Lrrn2), mRNA.
scl0021969.2_46-S	27.8043	1.93	Top1	NM_009408.1	Mus musculus topoisomerase (DNA) I (Top1), mRNA.
scl33040.15.1_23-S	27.7898	1.71	AI325941	NM_178900.2	Mus musculus expressed sequence AI325941 (AI325941), mRNA.
scl43073.2_375-S	27.7633	1.37	Hspa2	NM_008301.2	Mus musculus heat shock protein 2 (Hspa2), mRNA.
scl0217333.1_84-S	27.7371	1.37	Trim47	NM_172570.2	Mus musculus tripartite motif protein 47 (Trim47), mRNA.
scl23719.3_55-S	27.732	3.82	Gpr3	NM_008154.1	Mus musculus G-protein coupled receptor 3 (Gpr3), mRNA.
scl17355.9_0-S	27.6874	2.5	Glul	NM_008131.2	Mus musculus glutamate-ammonia ligase (glutamine synthase) (Glul), mRNA.
scl53400.8.1_51-S	27.6705	1.23	D19Ertd721e	NM_146093.1	Mus musculus DNA segment, Chr 19, ERATO Doi 721, expressed (D19Ertd721e), mRNA.
scl34524.3_290-S	27.6619	1.48	Siah1a	NM_009172.1	Mus musculus seven in absentia 1A (Siah1a), mRNA.
scl54248.9.17_2-S	27.6562	1.96	Gpc3	NM_016697.2	Mus musculus glypican 3 (Gpc3), mRNA.
scl47095.40_145-S	27.6541	1.32	Ptk2	NM_007982.1	Mus musculus PTK2 protein tyrosine kinase 2 (Ptk2), mRNA.
scl0066775.2_265-S	27.6479	2.25	4933428I03Rik	NM_025760.2	Mus musculus RIKEN cDNA 4933428I03 gene (4933428I03Rik), mRNA.
scl40126.18_106-S	27.601	1.47	Epn2	NM_010148.1	Mus musculus epsin 2 (Epn2), mRNA.
scl0011977.1_52-S	27.5805	2.31	Atp7a	NM_009726.2	Mus musculus ATPase, Cu++ transporting, alpha polypeptide (Atp7a), mRNA.
scl072554.6_30-S	27.5689	2.74	Utp14a	XM_135857.4	
scl24344.15.1_2-S	27.5623	1.48	Txndc4	NM_029572.1	Mus musculus thioredoxin domain containing 4 (endoplasmic reticulum) (Txndc4), mRNA.
scl35449.1.1_268-S	27.5479	3.42	B830007D08Rik	XM_150222.1	Mus musculus RIKEN cDNA B830007D08 gene (B830007D08Rik), mRNA.
scl21259.14_115-S	27.5383	5.11	Plxdc2	NM_026162.2	Mus musculus plexin domain containing 2 (Plxdc2), mRNA.
scl35976.44_48-S	27.5273	2.78	Arhgef12	NM_027144.1	Mus musculus Rho guanine nucleotide exchange factor (GEF) 12 (Arhgef12), mRNA.
scl31785.1.4_192-S	27.5158	1.53	Rasl2-9	NM_009028.1	Mus musculus RAS-like, family 2, locus 9 (Rasl2-9), mRNA.
scl0055943.1_284-S	27.4687	1.46	Stx8	NM_018768.1	Mus musculus syntaxin 8 (Stx8), mRNA.
scl0024074.2_26-S	27.4559	4.59	Taf7		
scl17938.36.1_1-S	27.4146	2.5	Aox3	NM_023617.1	
scl00218214.2_131-S	27.4044	1.91	Aof1	NM_172262.1	Mus musculus amine oxidase, flavin containing 1 (Aof1), mRNA.
scl47392.22_290-S	27.3583	1.91	Rai14	NM_030690.2	Mus musculus retinoic acid induced 14 (Rai14), mRNA.
scl0317757.1_85-S	27.3575	4.97	E230026N22Rik	NM_175035.3	Mus musculus RIKEN cDNA E230026N22 gene (E230026N22Rik), mRNA.
scl0001695.1_1621-S	27.3495	1.26	Ppm1b	NM_011151.1	Mus musculus protein phosphatase 1B, magnesium dependent, beta isoform (Ppm1b), mRNA.
scl0017159.2_303-S	27.3139	1.28	Man2b1	NM_010764	Mus musculus mannosidase 2, alpha B1 (Man2b1), mRNA.
scl0218333.1_228-S	27.2662	1.32	BC018507	XM_358313.1	Mus musculus cDNA sequence BC018507 (BC018507), mRNA.
scl47700.4_662-S	27.2505	2.74	AI481750	NM_145473.1	Mus musculus expressed sequence AI481750 (AI481750), mRNA.

IGKV4-75_AJ231227_Ig_	27.2439	6.31			
scl0234988.3_288-S	27.2326	5.25	Mbd3l2	NM_144934.2	Mus musculus methyl-CpG binding domain protein 3-like 2 (Mbd3l2), mRNA.
scl070302.4_170-S	27.2306	1.8	3110050K21Rik	XM_127780.4	Mus musculus RIKEN cDNA 3110050K21 gene (3110050K21Rik), mRNA.
scl30676.4_510-S	27.2295	2.83	2410170E21Rik	NM_029420.1	Mus musculus RIKEN cDNA 2410170E21 gene (2410170E21Rik), mRNA.
scl39638.4.1_92-S	27.2012	1.39	Rpl23	NM_022891.1	Mus musculus ribosomal protein L23 (Rpl23), mRNA.
scl34795.4.1_9-S	27.1828	1.26	Sap30	NM_021788.1	Mus musculus sin3 associated polypeptide (Sap30), mRNA.
scl24875.11.1_30-S	27.1286	1.49	Taf12	NM_025579.1	Mus musculus TAF12 RNA polymerase II, TATA box binding protein (TBP)-associated factor (Taf12), mRNA.
scl36724.29_71-S	27.0258	1.58	Nedd4	NM_010890.2	Mus musculus neural precursor cell expressed, developmentally down-regulted gene 4 (Nedd4), mRNA.
scl0078757.1_96-S	27.0007	1.35	4921505C17Rik	NM_030168.2	Mus musculus RIKEN cDNA 4921505C17 gene (4921505C17Rik), mRNA.
scl0023808.2_287-S	26.9903	1.47	Ash2l	NM_011791.1	Mus musculus ash2 (absent, small, or homeotic)-like (Drosophila) (Ash2l), mRNA.
scl00320165.1_190-S	26.972	1.28	B230378H13Rik	NM_177089.3	
scl34385.6.1_0-S	26.9105	6.11	Lcat	NM_008490.1	Mus musculus lecithin cholesterol acyltransferase (Lcat), mRNA.
scl019395.3_4-S	26.8891	4.91	Rasgrp2	XM_359299.1	Mus musculus RAS, guanyl releasing protein 2 (Rasgrp2), mRNA.
scl43398.10.1_4-S	26.8791	1.24	Laptm4a	NM_008640.1	Mus musculus lysosomal-associated protein transmembrane 4A (Laptm4a), mRNA.
scl32679.14.1_2-S	26.7532	4.62	Tulp2	NM_008807.1	Mus musculus tubby-like protein 2 (Tulp2), mRNA.
scl0026932.2_213-S	26.7294	1.39	Ppp2r5e	NM_012024.1	Mus musculus protein phosphatase 2, regulatory subunit B (B56), epsilon isoform (Ppp2r5e), mRNA.
scl00103850.1_287-S	26.7237	1.37	Nt5m	NM_134029.1	Mus musculus 5,3-nucleotidase, mitochondrial (Nt5m), mRNA.
scl069038.2_4-S	26.6712	1.22	1810006K21Rik	XM_207074	Mus musculus RIKEN cDNA 1810006K21 gene (1810006K21Rik), mRNA.
scl0024061.1_292-S	26.6039	1.34	Smc1l1	NM_019710.1	Mus musculus SMC (structural maintenance of chromosomes 1)-like 1 (S. cerevisiae) (Smc1l1), mRNA.
scl23497.11_62-S	26.5942	1.4	Pex14	NM_019781.1	Mus musculus peroxisomal biogenesis factor 14 (Pex14), mRNA.
scl18413.5.1_0-S	26.5777	2.2	9430008C03Rik	XM_149251.3	Mus musculus RIKEN cDNA 9430008C03 gene (9430008C03Rik), mRNA.
scl00104479.1_248-S	26.5123	1.59	BC018601	NM_134033.1	Mus musculus cDNA sequence BC018601 (BC018601), mRNA.
scl33446.12_65-S	26.4873	1.35	Cdh5	NM_009868	Mus musculus cadherin 5 (Cdh5), mRNA.
scl31483.18_189-S	26.47	1.4	4931406P16Rik	NM_172741.1	Mus musculus RIKEN cDNA 4931406P16 gene (4931406P16Rik), mRNA.
scl0216760.3_93-S	26.4637	1.74	Mfap3	NM_145426.1	
scl0213499.8_8-S	26.4523	5.16	Fbxo42	NM_172518.2	
scl019326.1_315-S	26.4444	1.32	Rab11b	NM_008997.1	Mus musculus RAB11B, member RAS oncogene family (Rab11b), mRNA.
scl50305.48_81-S	26.4342	1.27	Igf2r	NM_010515.1	Mus musculus insulin-like growth factor 2 receptor (Igf2r), mRNA.
scl0068682.1_300-S	26.4265	1.27	1110028E10Rik	NM_152808.1	Mus musculus RIKEN cDNA 1110028E10 gene (1110028E10Rik), mRNA.
scl41711.15_185-S	26.4233	2.96	Fbxw11	NM_134015.1	
scl0110265.1_302-S	26.4207	3.03	Msra	NM_026322.2	Mus musculus methionine sulfoxide reductase A (Msra), mRNA.
scl29286.18.1_125-S	26.4072	1.74	Ica1	NM_010492.2	Mus musculus islet cell autoantigen 1 (Ica1), mRNA.
scl45913.15.1_122-S	26.3724	3.6	Acox2	NM_053115.1	Mus musculus acyl-Coenzyme A oxidase 2, branched chain (Acox2), mRNA.
scl29671.63_470-S	26.3672	1.37	Itpr1	NM_010585.2	Mus musculus inositol 1,4,5-triphosphate receptor 1 (Itpr1), mRNA.
scl074302.1_207-S	26.3667	3.15	Mtmr3	NM_028860.1	Mus musculus myotubularin related protein 3 (Mtmr3), mRNA.
scl45854.5_74-S	26.3611	1.87	Usp54	NM_030180.1	Mus musculus ubiquitin specific protease 54 (Usp54), mRNA.
scl23632.8_120-S	26.3031	2.99	Ubxd3	NM_178671.2	Mus musculus UBX domain containing 3 (Ubxd3), mRNA.
scl00224023.1_141-S	26.2924	1.46	2610318I18Rik	NM_145479	Mus musculus RIKEN cDNA 2610318I18 gene (2610318I18Rik), mRNA.
scl0104871.12_180-S	26.2819	1.57	AI661438	NM_178914.3	
scl31965.9_618-S	26.2673	1.25	C430003P19Rik	NM_198017.1	Mus musculus RIKEN cDNA C430003P19 gene (C430003P19Rik), mRNA.
scl013000.1_10-S	26.2607	1.43	Csnk2a2	NM_009974.2	Mus musculus casein kinase II, alpha 2, polypeptide (Csnk2a2), mRNA.
scl45419.1.422_138-S	26.2462	1.3	Extl3	NM_018788.2	Mus musculus exostoses (multiple)-like 3 (Extl3), mRNA.
scl067116.1_6-S	26.2453	1.32	Cuedc2	NM_024192.1	Mus musculus CUE domain containing 2 (Cuedc2), mRNA.
scl38236.13.428_30-S	26.241	2.6	Magi1	NM_172546.1	Mus musculus membrane associated guanylate kinase interacting protein-like 1 (Magi1), mRNA.
scl26903.5_40-S	26.239	2.49	AI481214	NM_054098.2	
scl00102791.2_289-S	26.237	1.69	Tcta	NM_133986.1	
scl16734.10.1_6-S	26.2322	1.76	Clk1	NM_009905.1	
scl46638.4_82-S	26.1981	1.62	Kctd6	NM_027782.1	Mus musculus potassium channel tetramerisation domain containing 6 (Kctd6), mRNA.
scl068708.1_138-S	26.1941	1.69	Rabl2a	NM_026817.1	Mus musculus RAB, member of RAS oncogene family-like 2A (Rabl2a), mRNA.
scl26971.4.541_0-S	26.1658	1.24	Rpo1-3	NM_009087.1	Mus musculus RNA polymerase 1-3 (Rpo1-3), mRNA.
scl0018845.2_170-S	26.1546	1.36	Plxna2	NM_008882.1	Mus musculus plexin A2 (Plxna2), mRNA.
scl29584.3_468-S	26.1529	1.27	Zfp637	NM_177684.2	
scl47734.11_199-S	26.1422	1.35	Map3k7ip1	NM_025609.2	Mus musculus mitogen-activated protein kinase kinase kinase 7 interacting protein 1 (Map3k7ip1), mRNA.
scl000501.1_12-S	26.1366	2.64	Uhrf2	NM_144873.1	Mus musculus ubiquitin-like, containing PHD and RING finger domains 2 (Uhrf2), mRNA.
scl47579.6.1_16-S	26.134	1.39	B930062P21Rik	NM_178789.2	Mus musculus RIKEN cDNA B930062P21 gene (B930062P21Rik), mRNA.
scl41150.22.1_20-S	26.1231	1.24	Cmya4	NM_178680.2	Mus musculus cardiomyopathy associated 4 (Cmya4), mRNA.

scl0002727.1_932-S	26.119	3.13	Thea	NM_025590.3	Mus musculus thioesterase, adipose associated (Thea), mRNA.
scl53499.1.4_164-S	26.1027	5.55	1700020D05Rik	NM_023781.3	Mus musculus RIKEN cDNA 1700020D05 gene (1700020D05Rik), mRNA.
scl027967.3_2-S	26.0921	1.5	Cherp	NM_138585.2	Mus musculus calcium homeostasis endoplasmic reticulum protein (Cherp), mRNA.
scl0067665.1_200-S	26.0752	1.24	Dctn4	NM_026302.2	Mus musculus dynactin 4 (Dctn4), mRNA.
scl0016997.2_276-S	26.0667	1.72	Ltbp2	NM_013589.1	Mus musculus latent transforming growth factor beta binding protein 2 (Ltbp2), mRNA.
scl00216233.1_1004-S	26.0567	1.94	Socs2	NM_007706.1	Mus musculus suppressor of cytokine signaling 2 (Socs2), mRNA.
scl22226.9.1_81-S	26.0371	1.25	Nudt6	NM_153561.1	Mus musculus nudix (nucleoside diphosphate linked moiety X)-type motif 6 (Nudt6), mRNA.
scl0208715.1_140-S	26.0362	1.37	Hmgcs1	NM_145942.2	Mus musculus 3-hydroxy-3-methylglutaryl-Coenzyme A synthase 1 (Hmgcs1), mRNA.
scl097820.1_227-S	26.015	1.21	4833439L19Rik	NM_133797	Mus musculus RIKEN cDNA 4833439L19 gene (4833439L19Rik), mRNA.
scl00347722.2_310-S	26.0079	1.46	Centg2	NM_178119.2	Mus musculus centaurin, gamma 2 (Centg2), mRNA.
scl0077590.2_319-S	26.0041	1.95	4631426J05Rik	NM_029935.2	Mus musculus RIKEN cDNA 4631426J05 gene (4631426J05Rik), mRNA.
scl00213484.2_217-S	25.9848	1.43	BC036718	NM_153136.1	Mus musculus cDNA sequence BC036718 (BC036718), mRNA.
scl29492.60.1_120-S	25.9375	1.37	Vwf	NM_011708.2	Mus musculus Von Willebrand factor homolog (Vwf), mRNA.
scl22890.10.1_51-S	25.9293	1.8	Tmod4	NM_016712.1	Mus musculus tropomodulin 4 (Tmod4), mRNA.
scl29880.12.1_23-S	25.926	2.46	0610039N19Rik	NM_026159.2	Mus musculus RIKEN cDNA 0610039N19 gene (0610039N19Rik), mRNA.
scl0011569.2_215-S	25.896	2.98	Aebp2	NM_009637.1	Mus musculus AE binding protein 2 (Aebp2), mRNA.
scl015483.1_7-S	25.8933	1.5	Hsd11b1	NM_008288	Mus musculus hydroxysteroid 11-beta dehydrogenase 1 (Hsd11b1), mRNA.
scl075267.1_5-S	25.8468	1.92	4930555L03Rik	XM_204030.1	
scl34568.25_184-S	25.8427	1.71	BC016188	NM_145970.1	Mus musculus cDNA sequence BC016188 (BC016188), mRNA.
scl0001585.1_28-S	25.8142	2.27	Hdac5	NM_010412.2	Mus musculus histone deacetylase 5 (Hdac5), mRNA.
scl019358.2_23-S	25.7729	1.76	Rad23a	NM_009010.2	Mus musculus RAD23a homolog (S. cerevisiae) (Rad23a), mRNA.
scl44477.7_25-S	25.7627	3.64	Ankra2	NM_023472.1	Mus musculus ankyrin repeat, family A (RFXANK-like), 2 (Ankra2), mRNA.
scl48261.13.240_30-S	25.7534	1.22	Cct8	NM_009840.2	Mus musculus chaperonin subunit 8 (theta) (Cct8), mRNA.
scl26699.18.1_4-S	25.7483	1.6	2610033H07Rik	NM_029278.1	Mus musculus RIKEN cDNA 2610033H07 gene (2610033H07Rik), mRNA.
scl014630.7_323-S	25.7418	2.57	Gclm	NM_008129.2	Mus musculus glutamate-cysteine ligase , modifier subunit (Gclm), mRNA.
scl020916.11_281-S	25.7138	1.6	Sucla2	XM_127733.2	Mus musculus succinate-Coenzyme A ligase, ADP-forming, beta subunit (Sucla2), mRNA.
scl011722.3_4-S	25.7129	2.18	Amy1	NM_007446.1	Mus musculus amylase 1, salivary (Amy1), mRNA.
scl00234725.2_22-S	25.7114	2.12	Zfp612	NM_175480.3	Mus musculus zinc finger protein 612 (Zfp612), mRNA.
scl43387.27.1_23-S	25.6962	1.45	Smc6l1	NM_025695.2	Mus musculus SMC6 structural maintenance of chromosomes 6-like 1 (yeast) (Smc6l1), mRNA.
scl065111.1_137-S	25.6886	1.49	Dap3	NM_022994.2	Mus musculus death associated protein 3 (Dap3), mRNA.
scl00226539.2_30-S	25.6425	1.78	5830468K18Rik	NM_172644.1	Mus musculus RIKEN cDNA 5830468K18 gene (5830468K18Rik), mRNA.
scl0021681.1_61-S	25.6416	1.32	Thoc4	NM_011568.1	
scl38420.17.1_82-S	25.6224	3.48	Ptprr	NM_011217.1	Mus musculus protein tyrosine phosphatase, receptor type, R (Ptprr), mRNA.
scl0014858.1_22-S	25.6206	3.53	Gsta2	NM_008182.1	Mus musculus glutathione S-transferase, alpha 2 (Yc2) (Gsta2), mRNA.
scl23964.7.1_102-S	25.6034	1.22	2810405F18Rik	NM_028142.1	Mus musculus RIKEN cDNA 2810405F18 gene (2810405F18Rik), mRNA.
scl0056351.1_126-S	25.5974	1.25	Tebp	NM_019766.2	Mus musculus telomerase binding protein, p23 (Tebp), mRNA.
scl00023.1_1-S	25.5895	3.1	Cyp2a5	NM_007812.1	Mus musculus cytochrome P450, family 2, subfamily a, polypeptide 5 (Cyp2a5), mRNA.
scl0002264.1_586-S	25.5855	1.37	Epc1	NM_007935.1	Mus musculus enhancer of polycomb homolog 1 (Drosophila) (Epc1), transcript variant 2, mRNA.
scl46990.11.1_92-S	25.5841	4.72	Il2rb	NM_008368.3	Mus musculus interleukin 2 receptor, beta chain (Il2rb), mRNA.
scl068738.1_270-S	25.5797	1.33	Acas2l	NM_080575.1	Mus musculus acetyl-Coenzyme A synthetase 2 (AMP forming)-like (Acas2l), mRNA.
scl29346.9.1_7-S	25.5256	1.27	Mrps35	NM_145573.1	Mus musculus mitochondrial ribosomal protein S35 (Mrps35), mRNA.
scl41264.42.1_0-S	25.5174	1.27	Prpf8	NM_138659.1	Mus musculus pre-mRNA processing factor 8 (Prpf8), mRNA.
scl0227290.1_240-S	25.5112	1.35	Aamp	NM_146110.1	Mus musculus angio-associated migratory protein (Aamp), mRNA.
scl022152.4_13-S	25.509	2.76	Tubb3	NM_023279.2	Mus musculus tubulin, beta 3 (Tubb3), mRNA.
scl0231503.1_74-S	25.4947	3.92	BC062109	NM_182841.1	
scl0027029.2_174-S	25.4917	1.57	Sgsh	NM_018822.2	Mus musculus N-sulfoglucosamine sulfohydrolase (sulfamidase) (Sgsh), mRNA.
scl0068501.1_28-S	25.4867	4.04	1110014D18Rik	NM_026746.1	Mus musculus RIKEN cDNA 1110014D18 gene (1110014D18Rik), mRNA.
scl28523.18.1_12-S	25.4441	1.34	Raf1	NM_029780.1	Mus musculus v-raf-1 leukemia viral oncogene 1 (Raf1), mRNA.
scl32669.8.1_3-S	25.4239	1.76	Car11	NM_009800.2	Mus musculus carbonic anhydrase 11 (Car11), mRNA.
scl35535.28_151-S	25.4188	1.31	Ibtk	XM_135030.4	
scl0015950.1_6-S	25.4151	1.69	Ifi203	NM_008328.1	Mus musculus interferon activated gene 203 (Ifi203), mRNA.
scl00238247.1_16-S	25.4103	3.77	Arid4a	XM_354675.1	
scl0104771.9_30-S	25.4007	1.27	1200003C05Rik	NM_024205.1	Mus musculus RIKEN cDNA 1200003C05 gene (1200003C05Rik), mRNA.
scl54930.9.1_1-S	25.3966	1.23	Hprt	NM_013556	
scl020926.1_330-S	25.3769	1.54	Supt6h	NM_009297.1	Mus musculus suppressor of Ty 6 homolog (S. cerevisiae) (Supt6h), mRNA.
scl29041.8.1_24-S	25.3452	1.56	1810009M01Rik	NM_023056.2	Mus musculus RIKEN cDNA 1810009M01 gene (1810009M01Rik), mRNA.

scl00268490.1_147-S	25.3372	1.46	2600001B17Rik	NM_172947.1	
scl36548.17_86-S	25.3261	1.25	Ryk	NM_013649.2	Mus musculus receptor-like tyrosine kinase (Ryk), mRNA.
scl018655.12_25-S	25.3219	1.21	Pgk1	NM_008828	Mus musculus phosphoglycerate kinase 1 (Pgk1), mRNA.
scl067665.1_0-S	25.3114	3.43	Dctn4	NM_026302.2	Mus musculus dynactin 4 (Dctn4), mRNA.
scl24593.4_239-S	25.3054	1.32	2310042D19Rik	NM_172417.1	Mus musculus RIKEN cDNA 2310042D19 gene (2310042D19Rik), mRNA.
scl0004042.1_234-S	25.304	2.49	Fln29	NM_172275.1	Mus musculus FLN29 gene product (Fln29), mRNA.
scl0217837.1_325-S	25.2808	1.52	Itpk1	NM_172584.1	Mus musculus inositol 1,3,4-triphosphate 5/6 kinase (Itpk1), mRNA.
scl058809.2_14-S	25.2673	1.5	Rnase4	NM_021472.1	Mus musculus ribonuclease, RNase A family 4 (Rnase4), mRNA.
scl0230796.1_23-S	25.245	3.4	Wdtc1	NM_199306.1	
scl55039.15_103-S	25.2077	1.4	Ddx3x	NM_010028	Mus musculus DEAD/H (Asp-Glu-Ala-Asp/His) box polypeptide 3, X-linked (Ddx3x), mRNA.
scl49875.3_8-S	25.138	1.86	Mad2l1bp	NM_025649.1	Mus musculus MAD2L1 binding protein (Mad2l1bp), mRNA.
scl34623.10.1_30-S	25.1378	1.83	Tmem34	NM_145599.2	
scl0235072.13_139-S	25.1344	1.63	39332	NM_009859.2	Mus musculus septin 7 (Sept7), mRNA.
scl018771.13_29-S	25.1205	1.98	Pknox1	NM_016670.2	Mus musculus Pbx/knotted 1 homeobox (Pknox1), mRNA.
scl53380.11_494-S	25.1181	1.23	Fads1	NM_146094.1	Mus musculus fatty acid desaturase 1 (Fads1), mRNA.
scl38713.11.10_6-S	25.0905	1.28	Bsg	NM_009768.1	Mus musculus basigin (Bsg), mRNA.
scl53438.9.1_79-S	25.0888	1.36	Ndufv1	NM_133666.1	Mus musculus NADH dehydrogenase (ubiquinone) flavoprotein 1 (Ndufv1), mRNA.
scl0001050.1_29-S	25.0547	1.31	Grcc2f	NM_013536.1	Mus musculus gene rich cluster, C2f gene (Grcc2f), mRNA.
scl056399.1_113-S	25.0133	1.4	Akap8	NM_019774.2	Mus musculus A kinase (PRKA) anchor protein 8 (Akap8), mRNA.
scl00001.1_0-S	25.0003	1.48	2400010D15Rik	NM_029623.1	Mus musculus RIKEN cDNA 2400010D15 gene (2400010D15Rik), mRNA.
scl54295.26.1_21-S	24.9879	2.38	Smarca1	NM_053123.3	Mus musculus SWI/SNF related, matrix associated, actin dependent regulator of chromatin, subfamily a, member 1 (Smarca1), mRNA.
scl0066870.1_13-S	24.9752	1.31	1200009K13Rik	NM_025814.1	Mus musculus RIKEN cDNA 1200009K13 gene (1200009K13Rik), mRNA.
scl0067123.2_83-S	24.9685	1.57	Ubap1	NM_023305.2	Mus musculus ubiquitin-associated protein 1 (Ubap1), mRNA.
scl0011677.2_223-S	24.967	1.42	Akr1b3	NM_009658.2	Mus musculus aldo-keto reductase family 1, member B3 (aldose reductase) (Akr1b3), mRNA.
scl0219158.1_104-S	24.9665	1.67	2610301G19Rik	XM_358314.1	Mus musculus RIKEN cDNA 2610301G19 gene (2610301G19Rik), mRNA.
scl19667.9.1_67-S	24.9462	1.29	Rsu1	NM_009105	Mus musculus Ras suppressor protein 1 (Rsu1), mRNA.
scl23237.22_584-S	24.9452	1.52	3110057O12Rik	NM_026622.1	Mus musculus RIKEN cDNA 3110057O12 gene (3110057O12Rik), mRNA.
scl0016157.2_292-S	24.9268	1.36	Il11ra1	NM_010549.1	Mus musculus interleukin 11 receptor, alpha chain 1 (Il11ra1), mRNA.
scl31386.5.1_16-S	24.8975	6.4	AI414495	NM_015789.2	
scl50992.26_310-S	24.8927	1.76	Clcn7	NM_011930.2	Mus musculus chloride channel 7 (Clcn7), mRNA.
scl31589.11.1_58-S	24.8901	1.21	Psmc4	NM_011874.1	Mus musculus proteasome (prosome, macropain) 26S subunit, ATPase, 4 (Psmc4), mRNA.
scl38298.13.28_46-S	24.8669	1.38	Atp5b	NM_016774.2	Mus musculus ATP synthase, H+ transporting mitochondrial F1 complex, beta subunit (Atp5b), mRNA.
scl000022.1_12-S	24.8306	1.25	Cpt2	NM_009949	Mus musculus carnitine palmitoyltransferase 2 (Cpt2), mRNA.
scl0029875.2_286-S	24.8239	1.43	Iqgap1	NM_016721.1	Mus musculus IQ motif containing GTPase activating protein 1 (Iqgap1), mRNA.
scl012909.6_94-S	24.8029	1.45	Crcp	NM_007761.1	Mus musculus calcitonin gene-related peptide-receptor component protein (Crcp), mRNA.
GI_7106304-S	24.7995	7.24	En1	NM_010133.1	Mus musculus engrailed 1 (En1), mRNA.
scl0378466.6_0-S	24.7994	3.78	9330175B10Rik	NM_194351.1	Mus musculus RIKEN cDNA 9330175B10 gene (9330175B10Rik), mRNA.
scl42456.11_22-S	24.7942	3.25	BC065123	NM_172576.1	
scl33443.4.1_10-S	24.7887	7.14	Cklfsf2b	NM_028524.1	Mus musculus chemokine-like factor super family 2B (Cklfsf2b), mRNA.
scl0075751.2_140-S	24.7605	1.74	Ipo4	NM_024267.4	Mus musculus importin 4 (Ipo4), mRNA.
scl0225341.10_229-S	24.7444	1.52	Lims2	NM_144862.1	Mus musculus LIM and senescent cell antigen like domains 2 (Lims2), mRNA.
scl011906.16_208-S	24.6988	1.42	Atbf1	NM_007496.1	Mus musculus AT motif binding factor 1 (Atbf1), mRNA.
scl37463.11_46-S	24.6928	1.23	Mdm2	NM_010786.2	Mus musculus transformed mouse 3T3 cell double minute 2 (Mdm2), mRNA.
scl39882.5_436-S	24.6566	1.37	AI316787	NM_199199.1	Mus musculus expressed sequence AI316787 (AI316787), mRNA.
scl0001516.1_2-S	24.6458	2.46	Sgcd	NM_011891.2	Mus musculus sarcoglycan, delta (dystrophin-associated glycoprotein) (Sgcd), mRNA.
scl45165.9.4_29-S	24.6318	1.59	Dct	NM_010024	Mus musculus dopachrome tautomerase (Dct), mRNA.
scl45770.11.1_1-S	24.6111	1.23	C77032	NM_153547.2	Mus musculus EST C77032 (C77032), transcript variant 1, mRNA.
scl00239652.2_53-S	24.5984	3.47	9930016F01Rik	NM_173769.2	Mus musculus RIKEN cDNA 9930016F01 gene (9930016F01Rik), mRNA.
scl00353208.1_64-S	24.579	1.53	2810021G02Rik		
scl46722.5_604-S	24.5786	1.84	BC035295	NM_153407.1	Mus musculus cDNA sequence BC035295 (BC035295), mRNA.
scl23508.1.1_311-S	24.5613	1.4	Kif1b	NM_207682.1	Mus musculus kinesin family member 1B (Kif1b), transcript variant 1, mRNA.
scl0209039.30_312-S	24.5597	2.19	Tenc1	NM_153533.1	Mus musculus tensin like C1 domain-containing phosphatase (Tenc1), mRNA.
scl50307.11.1_44-S	24.5594	1.69	Slc22a3	NM_011395.1	Mus musculus solute carrier family 22 (organic cation transporter), member 3 (Slc22a3), mRNA.
scl0052231.1_82-S	24.5437	1.93	D1Ertd161e	NM_026187.3	Mus musculus DNA segment, Chr 1, ERATO Doi 161, expressed (D1Ertd161e), mRNA.
scl28403.9.1_10-S	24.5194	3.44	Ltbr	NM_010736.1	Mus musculus lymphotoxin B receptor (Ltbr), mRNA.
scl00394433.1_10-S	24.5136	2.48	Ugt1a2	NM_013701.1	

scl27525.13.1_50-S	24.5135	1.28	Arhgap24	NM_146161.1	Mus musculus Rho GTPase activating protein 24 (Arhgap24), mRNA.
scl012864.3_58-S	24.5035	1.3	Cox6c	NM_053071.1	Mus musculus cytochrome c oxidase, subunit VIc (Cox6c), mRNA.
scl21824.4.1_15-S	24.4868	1.57	L259	NM_153513.1	
scl059287.1_209-S	24.4849	1.24	Ncstn	NM_021607.2	Mus musculus nicastrin (Ncstn), mRNA.
scl054324.3_167-S	24.479	1.43	Arhgef5	XM_133067.3	Mus musculus Rho guanine nucleotide exchange factor (GEF) 5 (Arhgef5), mRNA.
scl25795.24.1_9-S	24.477	2.57	2210010N04Rik	XM_149712.5	Mus musculus RIKEN cDNA 2210010N04 gene (2210010N04Rik), mRNA.
scl35713.2.1_23-S	24.4705	1.27	2300009A05Rik	XM_204313.2	Mus musculus RIKEN cDNA 2300009A05 gene (2300009A05Rik), mRNA.
scl33851.12.1_1-S	24.4626	1.33	1810047C23Rik	NM_138668.1	Mus musculus RIKEN cDNA 1810047C23 gene (1810047C23Rik), mRNA.
scl0070834.1_254-S	24.372	1.55	Spag9	NM_027569.1	Mus musculus sperm associated antigen 9 (Spag9), mRNA.
scl0110796.1_34-S	24.3717	1.27	Sdccag33	XM_129060.3	Mus musculus serologically defined colon cancer antigen 33 (Sdccag33), mRNA.
scl000985.1_28-S	24.2903	4.29	Tsga10	NM_207228	Mus musculus testis specific 10 (Tsga10), mRNA.
scl0229473.1_11-S	24.2847	1.4	D930015E06Rik	NM_172681.2	Mus musculus RIKEN cDNA D930015E06 gene (D930015E06Rik), mRNA.
scl026874.2_21-S	24.2811	3.85	Abcd2	NM_011994.1	Mus musculus ATP-binding cassette, sub-family D (ALD), member 2 (Abcd2), mRNA.
scl077574.1_0-S	24.2227	2.28	3321401G04Rik	XM_133096.5	
scl0228983.14_82-S	24.2021	1.23	Osbpl2	NM_144500.1	Mus musculus oxysterol binding protein-like 2 (Osbpl2), mRNA.
scl0056542.1_233-S	24.1929	1.51	Ick	NM_019987.1	Mus musculus intestinal cell kinase (Ick), mRNA.
scl00328468.1_58-S	24.1449	2.2	C330046E03		
scl21165.1.12_85-S	24.137	2.2	Bmyc	NM_023326	Mus musculus brain expressed myelocytomatosis oncogene (Bmyc), mRNA.
scl30591.7_70-S	24.1299	4.69	Zfpn1a5	NM_175115.4	Mus musculus zinc finger protein, subfamily 1A, 5 (Zfpn1a5), mRNA.
scl0002307.1_1088-S	24.1245	2.13	Rps6ka5	NM_153587	Mus musculus ribosomal protein S6 kinase, polypeptide 5 (Rps6ka5), mRNA.
scl18363.8_35-S	24.1212	1.89	Sdc4	NM_011521.1	Mus musculus syndecan 4 (Sdc4), mRNA.
scl014252.10_327-S	24.1027	1.53	Flot2	NM_008028.1	Mus musculus flotillin 2 (Flot2), mRNA.
scl00234797.2_307-S	24.0659	2.04	6430548M08Rik	NM_172286.2	Mus musculus RIKEN cDNA 6430548M08 gene (6430548M08Rik), mRNA.
scl0194404.1_81-S	24.0583	1.48	BC030863	XM_359324.1	Mus musculus cDNA sequence BC030863 (BC030863), mRNA.
scl29342.18_151-S	24.0264	1.48	1810060J02Rik	NM_025911.1	Mus musculus RIKEN cDNA 1810060J02 gene (1810060J02Rik), mRNA.
scl42553.13_222-S	24.0242	1.31	Hbp1	NM_153198	Mus musculus high mobility group box transcription factor 1 (Hbp1), mRNA.
scl23164.14.1_82-S	24.018	1.22	D3Ertd194e	XM_130881.3	Mus musculus DNA segment, Chr 3, ERATO Doi 194, expressed (D3Ertd194e), mRNA.
scl53489.23_479-S	24.0088	1.8	Pacs1	XM_283545.1	Mus musculus phosphofurin acidic cluster sorting protein 1 (Pacs1), mRNA.
scl38548.13.242_0-S	24.0052	3.11	Lta4h	NM_008517.1	Mus musculus leukotriene A4 hydrolase (Lta4h), mRNA.
scl43495.3.1_6-S	23.9831	1.26	2310016C16Rik	NM_027127.1	Mus musculus RIKEN cDNA 2310016C16 gene (2310016C16Rik), mRNA.
scl31426.8.1_131-S	23.9746	3.46	2610041B18Rik	NM_027264.1	Mus musculus RIKEN cDNA 2610041B18 gene (2610041B18Rik), mRNA.
scl23522.8_437-S	23.9623	1.81	Fbxo44	NM_173401.1	
scl0023802.2_62-S	23.9297	1.2	Amfr	NM_011787.1	Mus musculus autocrine motility factor receptor (Amfr), mRNA.
scl46617.10.1_4-S	23.8915	1.81	Il3ra	NM_008369.1	Mus musculus interleukin 3 receptor, alpha chain (Il3ra), mRNA.
scl000764.1_2-S	23.8891	1.3	Sumo1	NM_009460.1	
scl00243813.1_281-S	23.8599	1.86	Leng9	NM_175529.2	
scl066335.13_14-S	23.8432	1.36	Atp6v1c1	NM_025494.1	Mus musculus ATPase, H+ transporting, V1 subunit C, isoform 1 (Atp6v1c1), mRNA.
scl17140.18.1_11-S	23.7962	2.88	Adprt1	NM_007415.2	Mus musculus ADP-ribosyltransferase (NAD+; poly (ADP-ribose) polymerase) 1 (Adprt1), mRNA.
scl00241308.2_305-S	23.7876	3.79	Ralgps1	NM_175211.2	
scl069601.7_240-S	23.7823	1.24	Dab2ip	NM_001001602	Mus musculus disabled homolog 2 (Drosophila) interacting protein (Dab2ip), mRNA.
scl0017993.1_168-S	23.7623	1.25	Ndufs4	NM_010887.1	Mus musculus NADH dehydrogenase (ubiquinone) Fe-S protein 4 (Ndufs4), mRNA.
scl0019684.1_297-S	23.7239	1.9	Rdx	NM_009041.1	Mus musculus radixin (Rdx), mRNA.
scl0003687.1_76-S	23.69	3.07	Trim23	NM_030731.1	Mus musculus tripartite motif protein 23 (Trim23), mRNA.
scl43593.17_243-S	23.6593	1.31	Rad17	NM_011233.1	Mus musculus RAD17 homolog (S. pombe) (Rad17), mRNA.
scl54877.7_76-S	23.641	1.5	BC023829	XM_135913.3	Mus musculus cDNA sequence BC023829 (BC023829), mRNA.
scl068453.4_14-S	23.5828	1.7	Gpihbp1	XM_128001.2	
scl014569.11_60-S	23.5725	1.49	Gdi3	NM_008112.2	Mus musculus guanosine diphosphate (GDP) dissociation inhibitor 3 (Gdi3), mRNA.
scl46501.15.1_0-S	23.5578	3.26	Nek4	NM_011849.1	Mus musculus NIMA (never in mitosis gene a)-related expressed kinase 4 (Nek4), mRNA.
scl0002207.1_183-S	23.5516	1.25	Dtna	NM_010087.2	Mus musculus dystrobrevin alpha (Dtna), transcript variant 2, mRNA.
scl20221.14.1_27-S	23.5308	1.44	2310003L22Rik	NM_027093.1	Mus musculus RIKEN cDNA 2310003L22 gene (2310003L22Rik), mRNA.
scl0228714.3_276-S	23.5246	1.26	Csrp2bp	NM_181417.2	Mus musculus cysteine and glycine-rich protein 2 binding protein (Csrp2bp), mRNA.
scl17777.9.1_24-S	23.5199	1.8	Des	NM_010043.1	Mus musculus desmin (Des), mRNA.
scl40181.5.1_1-S	23.5126	2.62	2210407C18Rik	NM_144544.1	Mus musculus RIKEN cDNA 2210407C18 gene (2210407C18Rik), mRNA.
scl0069821.2_297-S	23.5065	1.27	1810059A23Rik	NM_178051.3	
scl36820.13_122-S	23.4997	1.29	2010321M09Rik	NM_175153.2	Mus musculus RIKEN cDNA 2010321M09 gene (2010321M09Rik), mRNA.
scl48494.5_274-S	23.4785	1.44	Gtf2e1	NM_028812.1	Mus musculus general transcription factor II E, polypeptide 1 (alpha subunit) (Gtf2e1), mRNA.

scl0011797.2_322-S	23.4618	1.5	Birc2	NM_007465.1	Mus musculus baculoviral IAP repeat-containing 2 (Birc2), mRNA.
scl30008.5_1-S	23.4317	1.87	Aqp1	NM_007472.1	Mus musculus aquaporin 1 (Aqp1), mRNA.
scl54141.46.3_34-S	23.4155	1.47	Flna	XM_289920.2	Mus musculus filamin, alpha (Flna), mRNA.
scl40580.5.1_111-S	23.4115	1.26	1110020P15Rik	NM_197979.1	Mus musculus RIKEN cDNA 1110020P15 gene (1110020P15Rik), mRNA.
scl33181.7.1_94-S	23.3762	3.47	1110067L22Rik	NM_026855	Mus musculus RIKEN cDNA 1110067L22 gene (1110067L22Rik), mRNA.
scl21104.8_445-S	23.3523	2.24	2900073H19Rik	NM_026615.2	Mus musculus RIKEN cDNA 2900073H19 gene (2900073H19Rik), mRNA.
scl0054683.2_242-S	23.3461	1.21	Prdx5	NM_012021.1	Mus musculus peroxiredoxin 5 (Prdx5), mRNA.
scl000341.1_0-S	23.2911	1.73	Adprtl2	NM_009632.2	Mus musculus ADP-ribosyltransferase (NAD+; poly(ADP-ribose) polymerase)-like 2 (Adprtl2), mRNA.
scl53034.9_68-S	23.2864	1.94	Casp7	NM_007611.1	Mus musculus caspase 7 (Casp7), mRNA.
scl48651.6_179-S	23.2678	2.2	Ehhadh	NM_023737.2	Mus musculus enoyl-Coenzyme A, hydratase/3-hydroxyacyl Coenzyme A dehydrogenase (Ehhadh), mRNA.
scl00235574.1_8-S	23.2579	2.02	Atp2c1	NM_175025.2	Mus musculus ATPase, Ca++-sequestering (Atp2c1), mRNA.
scl0018642.1_78-S	23.2483	1.25	Pfkm	NM_021514.2	Mus musculus phosphofructokinase, muscle (Pfkm), mRNA.
scl46602.21_8-S	23.2365	1.41	Nid2	NM_008695.1	Mus musculus nidogen 2 (Nid2), mRNA.
scl0050790.1_147-S	23.2251	1.34	Acsl4	NM_207625.1	Mus musculus acyl-CoA synthetase long-chain family member 4 (Acsl4), transcript variant 2, mRNA.
scl20206.6_190-S	23.198	1.87	Dstn	NM_019771.1	Mus musculus destrin (Dstn), mRNA.
scl18043.8_79-S	23.1622	2.15	D1Bwg0212e	NM_028043.2	Mus musculus DNA segment, Chr 1, Brigham & Womens Genetics 0212 expressed (D1Bwg0212e), mRNA.
scl066568.3_30-S	23.1619	3.94	2510027J23Rik	NM_025637	Mus musculus RIKEN cDNA 2510027J23 gene (2510027J23Rik), mRNA.
scl0016564.2_223-S	23.1328	1.82	Kif21a	NM_016705.1	Mus musculus kinesin family member 21A (Kif21a), mRNA.
scl00107358.2_164-S	23.1263	2.15	Smbp	NM_133352.1	Mus musculus SM-11044 binding protein (Smbp), mRNA.
scl017347.2_7-S	23.0893	1.55	Mknk2	NM_021462.2	Mus musculus MAP kinase-interacting serine/threonine kinase 2 (Mknk2), mRNA.
scl51993.3_190-S	23.0688	1.36	Eif1a	NM_010120.3	Mus musculus eukaryotic translation initiation factor 1A (Eif1a), mRNA.
scl31053.2.1_38-S	23.0626	1.2	1810020E01Rik	NM_025460.1	Mus musculus RIKEN cDNA 1810020E01 gene (1810020E01Rik), mRNA.
scl24032.3.148_1-S	23.0619	2.04	2210012G02Rik	NM_025617.1	Mus musculus RIKEN cDNA 2210012G02 gene (2210012G02Rik), mRNA.
scl41298.15_342-S	23.0487	6.02	Camkk1	NM_018883.1	Mus musculus calcium/calmodulin-dependent protein kinase kinase 1, alpha (Camkk1), mRNA.
scl0233744.9_15-S	23.0288	2.9	Spon1	NM_145584.1	Mus musculus spondin 1, (f-spondin) extracellular matrix protein (Spon1), mRNA.
scl000108.1_22-S	22.9977	3.81	Cttn	NM_007803.1	Mus musculus cortactin (Cttn), mRNA.
scl21963.5_3-S	22.9859	1.32	Syt11	NM_018804.2	Mus musculus synaptotagmin 11 (Syt11), mRNA.
scl19239.6_43-S	22.9473	7.11	1110055L24Rik	NM_025422.1	Mus musculus RIKEN cDNA 1110055L24 gene (1110055L24Rik), mRNA.
scl0067443.1_283-S	22.9376	1.2	Map1lc3b	NM_026160	Mus musculus microtubule-associated protein 1 light chain 3 beta (Map1lc3b), mRNA.
scl0106583.1_2-S	22.8948	1.48	Rbm16	NM_134123.2	Mus musculus RNA binding motif protein 16 (Rbm16), mRNA.
scl0013499.1_2-S	22.8834	1.21	Drr1		
scl0140792.13_120-S	22.8702	1.75	Colec12	NM_130449.1	Mus musculus collectin sub-family member 12 (Colec12), mRNA.
scl39016.40.1_145-S	22.8653	1.56	Lama4	NM_010681.1	Mus musculus laminin, alpha 4 (Lama4), mRNA.
scl53447.10_104-S	22.8613	2.95	1810062O14Rik	NM_178650.2	Mus musculus RIKEN cDNA 1810062O14 gene (1810062O14Rik), mRNA.
scl20092.24_625-S	22.8523	2.24	Dnmt3b	NM_010068.1	Mus musculus DNA methyltransferase 3B (Dnmt3b), mRNA.
scl25078.23.1_39-S	22.8358	1.42	4930467B06Rik	NM_029786.1	Mus musculus RIKEN cDNA 4930467B06 gene (4930467B06Rik), mRNA.
scl054214.31_157-S	22.8227	6.25	Golga4	NM_018748.2	Mus musculus golgi autoantigen, golgin subfamily a, 4 (Golga4), mRNA.
scl067582.10_107-S	22.8177	1.53	Slc25a26	NM_026255.2	Mus musculus solute carrier family 25 (mitochondrial carrier; phosphate carrier), member 26 (Slc25a26), mRNA.
scl00053.1_244-S	22.8017	2.26	C030034J04Rik	NM_175023.1	Mus musculus RIKEN cDNA C030034J04 gene (C030034J04Rik), mRNA.
scl45096.3_115-S	22.7902	1.53	Copeb	NM_011803	Mus musculus core promoter element binding protein (Copeb), mRNA.
scl44989.1_487-S	22.7709	1.36	Hist1h1c	NM_015786	Mus musculus histone 1, H1c (Hist1h1c), mRNA.
scl32577.4.1_30-S	22.7533	1.24	Mcee	XM_133510.4	
scl0320827.6_21-S	22.7418	3.08	C530008M17Rik	XM_287460.2	Mus musculus RIKEN cDNA C530008M17 gene (C530008M17Rik), mRNA.
scl0002105.1_331-S	22.6629	3.77	Kcnn3	XM_147305.1	Mus musculus potassium intermediate/small conductance calcium-activated channel, subfamily N, member 3 (Kcnn3), mRNA.
scl52427.9.154_111-S	22.66	1.51	Poll	NM_020032.1	Mus musculus polymerase (DNA directed), lambda (Poll), mRNA.
scl35703.13_436-S	22.6534	1.43	Map2k1	NM_008927.1	Mus musculus mitogen activated protein kinase kinase 1 (Map2k1), mRNA.
scl0024135.1_295-S	22.6493	1.51	Zfp68	NM_013844.1	Mus musculus zinc finger protein 68 (Zfp68), mRNA.
scl00219150.2_85-S	22.6487	4.09	F830020C16Rik	NM_177338.3	Mus musculus RIKEN cDNA F830020C16 gene (F830020C16Rik), mRNA.
scl41342.11.1_81-S	22.6154	1.8	Mgl1	NM_010796.1	Mus musculus macrophage galactose N-acetyl-galactosamine specific lectin 1 (Mgl1), mRNA.
scl066079.4_60-S	22.6137	1.32	0610027O18Rik	NM_025339.2	Mus musculus RIKEN cDNA 0610027O18 gene (0610027O18Rik), mRNA.
scl072722.1_216-S	22.6079	1.38	2810405J04Rik	NM_133747.1	Mus musculus RIKEN cDNA 2810405J04 gene (2810405J04Rik), mRNA.
scl28677.12_376-S	22.5885	1.66	Zfyve20	NM_030081.2	Mus musculus zinc finger, FYVE domain containing 20 (Zfyve20), mRNA.
scl0227800.12_249-S	22.5526	1.2	Rabgap1	NM_146121.1	Mus musculus RAB GTPase activating protein 1 (Rabgap1), mRNA.
scl20535.2.1_122-S	22.5467	3.73	A930018P22Rik	NM_026634.1	Mus musculus RIKEN cDNA A930018P22 gene (A930018P22Rik), mRNA.
scl44666.7_30-S	22.5373	1.77	BC028265	NM_172392.2	
scl0012861.1_173-S	22.5327	1.21	Cox6a1	NM_007748.2	Mus musculus cytochrome c oxidase, subunit VI a, polypeptide 1 (Cox6a1), mRNA.

scl072043.2_38-S	22.5078	1.3	Sulf2	XM_358343.1	
scl28755.11.1_30-S	22.5045	1.35	Dusp11	NM_028099.2	Mus musculus dual specificity phosphatase 11 (RNA/RNP complex 1-interacting) (Dusp11), mRNA.
scl48822.17_669-S	22.4758	2.29	D230007K08Rik	NM_175547.2	Mus musculus RIKEN cDNA D230007K08 gene (D230007K08Rik), mRNA.
scl0052712.1_238-S	22.4702	1.41	Zfp535	NM_026107.2	Mus musculus zinc finger protein 535 (Zfp535), mRNA.
scl0231871.16_33-S	22.4549	1.48	E330036I19Rik	NM_144915.2	Mus musculus RIKEN cDNA E330036I19 gene (E330036I19Rik), mRNA.
scl0218885.8_148-S	22.4451	1.22	BC019806	NM_145460	Mus musculus cDNA sequence BC019806 (BC019806), mRNA.
scl0208117.1_319-S	22.4124	1.5	4632417K02	NM_177583.2	Mus musculus hypothetical protein 4632417K02 (4632417K02), mRNA.
scl0065973.1_101-S	22.4119	1.4	Asph	NM_023066.1	Mus musculus aspartate-beta-hydroxylase (Asph), mRNA.
scl21992.22_340-S	22.4067	2.92	3110045G13Rik	NM_028460.1	Mus musculus RIKEN cDNA 3110045G13 gene (3110045G13Rik), mRNA.
scl0067500.2_43-S	22.3747	1.33	Ccar1	NM_026201.1	Mus musculus cell division cycle and apoptosis regulator 1 (Ccar1), mRNA.
scl24033.7.1_11-S	22.3616	1.21	Mrpl37	NM_025500.1	Mus musculus mitochondrial ribosomal protein L37 (Mrpl37), mRNA.
scl24094.1_31-S	22.3402	1.67	Jun	NM_010591.1	Mus musculus Jun oncogene (Jun), mRNA.
scl47786.6.1_30-S	22.338	1.6	2310010G13Rik	NM_027122.2	Mus musculus RIKEN cDNA 2310010G13 gene (2310010G13Rik), mRNA.
scl40257.8.9_25-S	22.3257	1.97	0610039K22Rik	NM_025346.1	Mus musculus RIKEN cDNA 0610039K22 gene (0610039K22Rik), mRNA.
scl00214063.1_272-S	22.3126	1.39	4732437J24Rik	NM_172338.1	Mus musculus RIKEN cDNA 4732437J24 gene (4732437J24Rik), mRNA.
scl26291.11.1_15-S	22.312	1.26	Sparcl1	NM_010097.2	Mus musculus SPARC-like 1 (mast9, hevin) (Sparcl1), mRNA.
scl43315.9.1_17-S	22.3089	2.15	Sh3yl1	NM_013709.2	Mus musculus Sh3 domain YSC-like 1 (Sh3yl1), mRNA.
scl068642.1_282-S	22.2855	1.82	2810441K11Rik	NM_026798.1	Mus musculus RIKEN cDNA 2810441K11 gene (2810441K11Rik), mRNA.
scl0054673.1_296-S	22.2788	1.92	Sh3glb1	NM_019464.1	Mus musculus SH3-domain GRB2-like B1 (endophilin) (Sh3glb1), mRNA.
scl0230088.1_170-S	22.2666	1.45	B230312A22Rik	NM_172691.1	Mus musculus RIKEN cDNA B230312A22 gene (B230312A22Rik), mRNA.
scl54915.11_540-S	22.2603	1.2	Htatsf1	NM_028242.1	
scl0018472.1_99-S	22.257	1.51	Pafah1b1	NM_013625.1	Mus musculus platelet-activating factor acetylhydrolase, isoform 1b, beta1 subunit (Pafah1b1), mRNA.
scl46811.9.582_26-S	22.2565	1.81	3000003F02Rik	NM_172437.1	Mus musculus RIKEN cDNA 3000003F02 gene (3000003F02Rik), mRNA.
scl23523.7.1_20-S	22.2486	1.35	Fbxo6b	NM_015797.1	Mus musculus F-box only protein 6b (Fbxo6b), mRNA.
scl41758.26.1_24-S	22.2463	1.55	Pnpt1	NM_027869.1	Mus musculus polyribonucleotide nucleotidyltransferase 1 (Pnpt1), mRNA.
scl33577.2_419-S	22.2327	1.82	Gadd45gip1	NM_183358.2	Mus musculus growth arrest and DNA-damage-inducible, gamma interacting protein 1 (Gadd45gip1), mRNA.
scl0003256.1_3-S	22.2302	1.34	Cdk5rap1	NM_025876.1	Mus musculus CDK5 regulatory subunit associated protein 1 (Cdk5rap1), mRNA.
scl24978.4_154-S	22.2294	2.06	Snip1	NM_175246.2	Mus musculus Smad nuclear interacting protein 1 (Snip1), mRNA.
scl0020404.1_267-S	22.1851	2.49	Sh3gl2	NM_019535.2	Mus musculus SH3-domain GRB2-like 2 (Sh3gl2), mRNA.
scl18127.10.1_92-S	22.1781	1.86	Gsta3	NM_010356.2	Mus musculus glutathione S-transferase, alpha 3 (Gsta3), mRNA.
scl42964.22.1_29-S	22.1746	1.42	Ylpm1	NM_178363.2	
scl32676.9.1_1-S	22.1716	2.3	Plekha4	NM_148927.1	Mus musculus pleckstrin homology domain containing, family A (phosphoinositide binding specific) member 4 (Plekha4), mRNA.
scl25806.10.1_16-S	22.1667	1.28	Zdhhc4	NM_028379.1	Mus musculus zinc finger, DHHC domain containing 4 (Zdhhc4), mRNA.
scl30655.6_70-S	22.1601	1.24	Cd2bp2	NM_027353.2	Mus musculus CD2 antigen (cytoplasmic tail) binding protein 2 (Cd2bp2), mRNA.
scl066935.3_28-S	22.1561	1.83	1700023B02Rik	NM_025854.1	Mus musculus RIKEN cDNA 1700023B02 gene (1700023B02Rik), mRNA.
scl51588.6_379-S	22.1524	1.2	D030070L09Rik	NM_172625.1	Mus musculus RIKEN cDNA D030070L09 gene (D030070L09Rik), mRNA.
scl36433.10_466-S	22.1521	2.85	2610002I17Rik	XM_135177.4	Mus musculus RIKEN cDNA 2610002I17 gene (2610002I17Rik), mRNA.
scl39390.10.1_10-S	22.1366	2.06	BC029169	NM_153782.1	Mus musculus cDNA sequence BC029169 (BC029169), mRNA.
scl0012398.2_38-S	22.1267	1.39	Cbfa2t3h	NM_009824.1	Mus musculus core-binding factor, runt domain, alpha subunit 2; translocated to, 3 homolog (human) (Cbfa2t3h), mRNA.
scl22110.22.1_30-S	22.1166	1.27	Dhx36	NM_028136.1	Mus musculus DEAH (Asp-Glu-Ala-His) box polypeptide 36 (Dhx36), mRNA.
scl0105148.34_198-S	22.1045	4.21	Iars	NM_172015.1	Mus musculus isoleucine-tRNA synthetase (Iars), mRNA.
scl00026.1_111-S	22.0925	1.32	H47	NM_024439.2	Mus musculus histocompatibility 47 (H47), mRNA.
scl070938.1_239-S	22.0904	2.48	Vti1a	NM_183291.1	Mus musculus vesicle transport through interaction with t-SNAREs homolog 1A (yeast) (Vti1a), mRNA.
scl0012747.1_67-S	22.0809	1.56	Clk1	NM_009905.1	
scl00224143.2_195-S	22.0733	1.42	9630046K23Rik	NM_172380.1	Mus musculus RIKEN cDNA 9630046K23 gene (9630046K23Rik), mRNA.
scl0022195.1_278-S	22.064	1.32	Ube2l3	XM_148568.1	Mus musculus ubiquitin-conjugating enzyme E2L 3 (Ube2l3), mRNA.
scl0002518.1_2-S	22.0623	1.63	Mrpl13	NM_026759.2	Mus musculus mitochondrial ribosomal protein L13 (Mrpl13), mRNA.
scl18545.1_131-S	22.0436	1.28	Thbd	NM_009378.1	Mus musculus thrombomodulin (Thbd), mRNA.
scl0380773.4_2-S	22.0426	1.2	1810035L17Rik	XM_354684.1	
scl38302.14.1_13-S	21.9998	2.53	Prim1	NM_008921.1	Mus musculus DNA primase, p49 subunit (Prim1), mRNA.
scl29623.5_373-S	21.9818	1.49	Vhlh	NM_009507	Mus musculus von Hippel-Lindau syndrome homolog (Vhlh), mRNA.
scl34044.10_124-S	21.943	1.36	Fbxo25	NM_025785.1	Mus musculus F-box only protein 25 (Fbxo25), mRNA.
scl00234023.2_117-S	21.9046	1.21	9430010O03Rik	NM_176849.2	Mus musculus RIKEN cDNA 9430010O03 gene (9430010O03Rik), mRNA.
scl8888.1.1_269-S	21.9024	3.04	Olfr575	NM_147114.1	Mus musculus olfactory receptor 575 (Olfr575), mRNA.
scl18004.30_24-S	21.8921	1.27	Tpp2	NM_009418.1	Mus musculus tripeptidyl peptidase II (Tpp2), mRNA.
scl43584.17.1_2-S	21.8899	1.28	Slc30a5	NM_022885.1	Mus musculus solute carrier family 30 (zinc transporter), member 5 (Slc30a5), mRNA.

scl23200.12_402-S	21.8768	1.8	2810046L04Rik	NM_173382	Mus musculus RIKEN cDNA 2810046L04 gene (2810046L04Rik), mRNA.
scl50598.25_178-S	21.866	2.17	Jmjd2b	NM_172132.1	Mus musculus jumonji domain containing 2B (Jmjd2b), mRNA.
scl46229.3_225-S	21.8649	2.91	9430059P22Rik	NM_145463.3	Mus musculus RIKEN cDNA 9430059P22 gene (9430059P22Rik), mRNA.
scl53403.26.13_76-S	21.8646	1.32	Bscl2	NM_008144.3	Mus musculus Bernardinelli-Seip congenital lipodystrophy 2 homolog (human) (Bscl2), mRNA.
scl30499.14.1_7-S	21.8507	1.27	Rnh1	NM_145135.2	Mus musculus ribonuclease/angiogenin inhibitor 1 (Rnh1), mRNA.
scl0066408.1_304-S	21.8448	1.4	Aptx	NM_025545.2	Mus musculus aprataxin (Aptx), mRNA.
scl022793.9_329-S	21.8394	1.5	Zyx	NM_011777.1	Mus musculus zyxin (Zyx), mRNA.
scl068098.1_43-S	21.8359	1.23	Rchy1	NM_026557.2	Mus musculus ring finger and CHY zinc finger domain containing 1 (Rchy1), mRNA.
scl072465.7_112-S	21.8339	1.29	Zfp131	NM_028245.1	Mus musculus zinc finger protein 131 (Zfp131), mRNA.
scl074104.1_75-S	21.8299	1.33	Abcb6	NM_023732.2	Mus musculus ATP-binding cassette, sub-family B (MDR/TAP), member 6 (Abcb6), mRNA.
scl00210530.2_241-S	21.8158	2.79	Leprel1	NM_173379.1	
scl0078781.2_278-S	21.8066	1.7	Zc3hav1	NM_028864.1	Mus musculus zinc finger CCCH type, antiviral 1 (Zc3hav1), mRNA.
scl46056.12.1_60-S	21.7928	1.59	Mtrf1	NM_145960	Mus musculus mitochondrial translational release factor 1 (Mtrf1), mRNA.
scl32924.1_1-S	21.7711	1.63	BC025206	NM_146184.2	
scl0101095.9_35-S	21.7577	1.53	Zfp282	NM_146175.2	Mus musculus zinc finger protein 282 (Zfp282), mRNA.
scl073170.1_63-S	21.7473	3.36	Rwdd3	NM_028456	Mus musculus RWD domain containing 3 (Rwdd3), mRNA.
scl24881.12.1_60-S	21.7375	1.59	Nrbf1	NM_025297.1	Mus musculus nuclear receptor binding factor 1 (Nrbf1), mRNA.
scl18462.14.1_6-S	21.7354	2.91	Ncoa6	NM_019825.1	Mus musculus nuclear receptor coactivator 6 (Ncoa6), mRNA.
scl074610.15_28-S	21.7341	1.53	4833412N02Rik	NM_029020.2	Mus musculus RIKEN cDNA 4833412N02 gene (4833412N02Rik), mRNA.
scl51769.42.1_6-S	21.7091	1.55	Myo5b	NM_201600.1	Mus musculus myosin Vb (Myo5b), transcript variant 1, mRNA.
scl55015.6.1_13-S	21.6988	1.54	Timp1	NM_011593	Mus musculus tissue inhibitor of metalloproteinase 1 (Timp1), mRNA.
scl022629.4_2-S	21.6403	1.56	Ywhah	NM_011738	Mus musculus tyrosine 3-monooxygenase/tryptophan 5-monooxygenase activation protein, eta polypeptide (Ywhah), mRNA.
scl44090.4_104-S	21.5832	1.97	Nrn1	NM_153529.1	Mus musculus neuritin 1 (Nrn1), mRNA.
scl33085.7_52-S	21.5609	1.95	Zfp606	NM_026112.2	Mus musculus zinc finger protein 606 (Zfp606), mRNA.
scl38673.9_126-S	21.5323	3.42	Sf3a2	NM_013651.3	Mus musculus splicing factor 3a, subunit 2 (Sf3a2), mRNA.
scl0072020.1_140-S	21.522	1.87	1600021C16Rik	NM_028059.1	Mus musculus RIKEN cDNA 1600021C16 gene (1600021C16Rik), mRNA.
scl000592.1_8-S	21.4898	3.23	Hp	NM_017370.1	Mus musculus haptoglobin (Hp), mRNA.
scl42736.10.1_35-S	21.4892	3.83	Tdrd9	XM_127120.2	
scl48157.24.1_63-S	21.4867	2.91	Wdr9	NM_145125.1	Mus musculus WD repeat domain 9 (Wdr9), mRNA.
scl37396.13.1_16-S	21.48	1.33	D10Ertd610e	NM_028027.1	Mus musculus DNA segment, Chr 10, ERATO Doi 610, expressed (D10Ertd610e), mRNA.
scl0002306.1_28-S	21.4572	1.73		XM_283061.1	Mus musculus RIKEN cDNA A630086P08 gene (A630086P08Rik), mRNA.
scl51888.24_434-S	21.4429	1.3	Pdgfrb	NM_008809.1	Mus musculus platelet derived growth factor receptor, beta polypeptide (Pdgfrb), mRNA.
scl00217265.2_148-S	21.4425	3.27	Abca5	NM_147219.1	Mus musculus ATP-binding cassette, sub-family A (ABC1), member 5 (Abca5), mRNA.
scl20269.13_663-S	21.4373	1.32	Hspa12b	NM_028306.2	Mus musculus heat shock protein 12B (Hspa12b), mRNA.
GI_6671508-S	21.4365	1.36	Actb	NM_007393.1	Mus musculus actin, beta, cytoplasmic (Actb), mRNA.
scl44868.8_25-S	21.4344	1.59	Gcnt2	NM_008105.2	Mus musculus glucosaminyl (N-acetyl) transferase 2, I-branching enzyme (Gcnt2), transcript variant 3, mRNA.
scl23294.9_553-S	21.427	1.33	Zfp639	NM_144519	
scl31056.12.1_57-S	21.3968	1.25	Eed	NM_021876.1	Mus musculus embryonic ectoderm development (Eed), mRNA.
scl069938.2_27-S	21.3749	3.03	Scrn1	NM_027268.1	Mus musculus secernin 1 (Scrn1), mRNA.
scl022187.2_0-S	21.3694	1.46	Ubb	NM_011664.1	Mus musculus ubiquitin B (Ubb), mRNA.
scl011737.7_8-S	21.3649	1.33	Anp32a	NM_009672.2	Mus musculus acidic (leucine-rich) nuclear phosphoprotein 32 family, member A (Anp32a), mRNA.
scl016582.1_68-S	21.3498	1.49	Kifc3	NM_010631.1	Mus musculus kinesin family member C3 (Kifc3), mRNA.
scl50143.3.1_18-S	21.3484	1.62	0610039D01Rik	NM_026307.2	Mus musculus RIKEN cDNA 0610039D01 gene (0610039D01Rik), mRNA.
scl020536.5_65-S	21.3314	3.96	Slc4a3	NM_009208.1	Mus musculus solute carrier family 4 (anion exchanger), member 3 (Slc4a3), mRNA.
scl0231510.14_8-S	21.3287	1.66	4933408F15	NM_172715.1	Mus musculus hypothetical protein 4933408F15 (4933408F15), mRNA.
scl022724.2_70-S	21.3189	4.86	Zfp67	NM_009565.1	Mus musculus zinc finger protein 67 (Zfp67), mRNA.
scl23635.5.2_0-S	21.3176	1.34	Pink1	NM_026880.1	Mus musculus PTEN induced putative kinase 1 (Pink1), mRNA.
scl073247.26_162-S	21.315	2.95	1600027N09Rik	XM_130743.3	Mus musculus RIKEN cDNA 1600027N09 gene (1600027N09Rik), mRNA.
scl42747.5_440-S	21.2896	1.54	6720458F09Rik	NM_177374.2	Mus musculus RIKEN cDNA 6720458F09 gene (6720458F09Rik), mRNA.
scl00107029.2_8-S	21.2721	2.38	Me2	NM_145494.1	Mus musculus malic enzyme 2, NAD(+)-dependent, mitochondrial (Me2), mRNA.
scl49488.8_241-S	21.2649	1.26	Zfp263	NM_148924.2	Mus musculus zinc finger protein 263 (Zfp263), mRNA.
scl52628.17.1_63-S	21.2605	1.4	BC018472	NM_146097.1	Mus musculus cDNA sequence BC018472 (BC018472), mRNA.
scl066989.6_1-S	21.2558	1.52	2410004N11Rik	NM_025888.2	Mus musculus RIKEN cDNA 2410004N11 gene (2410004N11Rik), mRNA.
scl22167.22_124-S	21.2461	1.2	Cog6	NM_026225.1	Mus musculus component of oligomeric golgi complex 6 (Cog6), mRNA.
scl0217708.6_214-S	21.2442	4.36	5830457H20Rik	NM_173756.2	
scl067604.1_6-S	21.2407	1.36	1110007L15Rik	NM_026269.1	Mus musculus RIKEN cDNA 1110007L15 gene (1110007L15Rik), mRNA.

scl26137.12_266-S	21.2063	1.6	2400003N08Rik	NM_178622.3	Mus musculus RIKEN cDNA 2400003N08 gene (2400003N08Rik), mRNA.
scl28793.10_326-S	21.1988	1.35	Stambp	NM_024239.1	Mus musculus Stam binding protein (Stambp), mRNA.
scl059050.2_22-S	21.1924	1.18	5730427N09Rik	NM_021552.1	Mus musculus RIKEN cDNA 5730427N09 gene (5730427N09Rik), mRNA.
scl38879.8_20-S	21.1917	1.44	4632428N05Rik	NM_028732.2	Mus musculus RIKEN cDNA 4632428N05 gene (4632428N05Rik), mRNA.
scl056398.7_324-S	21.1687	1.18	1500003O03Rik	NM_019769.2	Mus musculus RIKEN cDNA 1500003O03 gene (1500003O03Rik), mRNA.
scl27967.7.1_149-S	21.1631	1.71	Abhd1	NM_021304.2	Mus musculus abhydrolase domain containing 1 (Abhd1), mRNA.
scl0117599.1_293-S	21.1529	1.32	Helb	NM_080446.1	Mus musculus helicase (DNA) B (Helb), mRNA.
scl42451.6.1_16-S	21.1427	2.8	Nfkbia	NM_010907	Mus musculus nuclear factor of kappa light chain gene enhancer in B-cells inhibitor, alpha (Nfkbia), mRNA.
scl53028.7_552-S	21.1062	1.21	Trim8	NM_053100.1	Mus musculus tripartite motif protein 8 (Trim8), mRNA.
scl41127.15.1_23-S	21.0855	1.35	Ddx52	NM_030096.1	Mus musculus DEAD (Asp-Glu-Ala-Asp) box polypeptide 52 (Ddx52), mRNA.
scl27567.9_262-S	21.0491	1.36	Ccng2	NM_007635.2	Mus musculus cyclin G2 (Ccng2), mRNA.
scl52081.14_14-S	21.0217	1.21	Ik	NM_011879.1	Mus musculus IK cytokine (Ik), mRNA.
scl016396.2_0-S	21.0052	2.95	Itch	XM_192925.3	Mus musculus itchy (Itch), mRNA.
scl24120.2_589-S	21.0037	1.94	Cdkn2b	NM_007670.2	Mus musculus cyclin-dependent kinase inhibitor 2B (p15, inhibits CDK4) (Cdkn2b), mRNA.
scl0066853.2_236-S	20.9822	1.96	Pnpla2	NM_025802.1	
scl0068299.1_136-S	20.9632	1.35	3100002B05Rik	NM_026664.2	Mus musculus RIKEN cDNA 3100002B05 gene (3100002B05Rik), mRNA.
scl066302.10_6-S	20.9593	2.69	2410005O16Rik	NM_025476.3	Mus musculus RIKEN cDNA 2410005O16 gene (2410005O16Rik), mRNA.
scl29590.2_145-S	20.9227	2.26	8430408G22Rik	NM_145980.1	Mus musculus RIKEN cDNA 8430408G22 gene (8430408G22Rik), mRNA.
scl020091.3_53-S	20.9185	1.21	Rps3a	NM_016959.2	Mus musculus ribosomal protein S3a (Rps3a), mRNA.
scl50284.10.368_11-S	20.8938	3.68	Dll1	NM_007865.2	Mus musculus delta-like 1 (Drosophila) (Dll1), mRNA.
scl49343.16.24_43-S	20.8915	1.2	Eif2b5	NM_172265.1	Mus musculus eukaryotic translation initiation factor 2B, subunit 5 epsilon (Eif2b5), mRNA.
scl25513.7.1_27-S	20.8846	1.23	Creb3	NM_013497.1	Mus musculus cAMP responsive element binding protein 3 (Creb3), mRNA.
scl21986.3.1_30-S	20.8808	1.55	Apoa1bp	NM_144897	Mus musculus apolipoprotein A-I binding protein (Apoa1bp), mRNA.
scl28959.7_65-S	20.8794	1.52	AW146242	NM_146168.1	Mus musculus expressed sequence AW146242 (AW146242), mRNA.
scl0020598.1_244-S	20.8738	1.32	Smpd2	NM_009213.1	Mus musculus sphingomyelin phosphodiesterase 2, neutral (Smpd2), mRNA.
scl29983.21.1_98-S	20.853	7.59	Abcg2	NM_011920.1	Mus musculus ATP-binding cassette, sub-family G (WHITE), member 2 (Abcg2), mRNA.
scl0003409.1_16-S	20.8436	3.29	A330080J22Rik	NM_178667.2	Mus musculus RIKEN cDNA A330080J22 gene (A330080J22Rik), mRNA.
scl46440.4.1_161-S	20.8316	2.45	Lrrc21	NM_146245.1	Mus musculus leucine rich repeat containing 21 (Lrrc21), mRNA.
scl27382.16_83-S	20.8274	1.34	1100001D10Rik	XM_358359.1	Mus musculus RIKEN cDNA 1100001D10 gene (1100001D10Rik), mRNA.
scl071435.1_313-S	20.8141	1.2	Arhgap21	XM_130033.5	
scl38001.5.1_73-S	20.7788	1.18	1700021F05Rik	NM_026411.1	Mus musculus RIKEN cDNA 1700021F05 gene (1700021F05Rik), mRNA.
scl43318.5_59-S	20.7564	3.19	Tmem18	NM_172049.1	
scl0002419.1_3-S	20.7397	1.26	Trappc6b	BC031464	Mus musculus trafficking protein particle complex 6B (Trappc6b), mRNA.
scl42858.13_56-S	20.7058	2.04	Rin3	NM_177620.2	Mus musculus Ras and Rab interactor 3 (Rin3), mRNA.
scl36309.11_147-S	20.6717	1.27	Abhd5	NM_026179.1	Mus musculus abhydrolase domain containing 5 (Abhd5), mRNA.
scl44629.24_90-S	20.6702	1.31	Slc12a7	NM_011390.1	Mus musculus solute carrier family 12, member 7 (Slc12a7), mRNA.
scl020973.4_189-S	20.6675	1.25	Syngr2	NM_009304.1	Mus musculus synaptogyrin 2 (Syngr2), mRNA.
scl0019727.2_202-S	20.6577	2.66	Rfxank	NM_011266.1	Mus musculus regulatory factor X-associated ankyrin-containing protein (Rfxank), mRNA.
scl021783.10_20-S	20.6403	2	A330080J22Rik	NM_178667.2	
scl066390.1_295-S	20.6056	1.19	2310042G06Rik	NM_025531.2	Mus musculus RIKEN cDNA 2310042G06 gene (2310042G06Rik), mRNA.
scl25676.27_663-S	20.6039	1.69	1110037F02Rik	XM_283937.2	Mus musculus RIKEN cDNA 1110037F02 gene (1110037F02Rik), mRNA.
scl17135.9_190-S	20.6003	1.25	Acbd3	NM_133225.1	Mus musculus acyl-Coenzyme A binding domain containing 3 (Acbd3), mRNA.
scl0067225.1_69-S	20.5927	2.04	2810441O16Rik	NM_026043.2	Mus musculus RIKEN cDNA 2810441O16 gene (2810441O16Rik), mRNA.
scl0067899.2_259-S	20.5898	1.41	2010110K16Rik	NM_026442.2	Mus musculus RIKEN cDNA 2010110K16 gene (2010110K16Rik), mRNA.
scl31521.10_99-S	20.5804	4.27	1810054G18Rik	NM_029377.1	Mus musculus RIKEN cDNA 1810054G18 gene (1810054G18Rik), mRNA.
scl0271457.7_14-S	20.5767	4.48	Rab5a	NM_025887.2	Mus musculus RAB5A, member RAS oncogene family (Rab5a), mRNA.
scl29313.9.1_4-S	20.5763	1.36	Pon3	NM_173006.1	Mus musculus paraoxonase 3 (Pon3), mRNA.
scl46659.10_50-S	20.5692	2.43	Zfp385	NM_013866.1	Mus musculus zinc finger protein 385 (Zfp385), mRNA.
scl25143.20.1_12-S	20.5649	1.98	A830039B04Rik	NM_177045.2	Mus musculus RIKEN cDNA A830039B04 gene (A830039B04Rik), mRNA.
scl49720.1.1_123-S	20.5604	3.07	BC016608	NM_146080.1	Mus musculus cDNA sequence BC016608 (BC016608), mRNA.
scl30523.17.39_21-S	20.5481	1.33	Tubgcp2	NM_133755.1	Mus musculus tubulin, gamma complex associated protein 2 (Tubgcp2), mRNA.
scl0067398.1_126-S	20.5436	1.24	Srpr	NM_026130.1	Mus musculus signal recognition particle receptor (docking protein) (Srpr), mRNA.
scl0002625.1_41-S	20.5175	2.05	2210012G02Rik	NM_025617.1	Mus musculus RIKEN cDNA 2210012G02 gene (2210012G02Rik), mRNA.
scl0030934.2_247-S	20.5135	1.75	Tor1b	NM_133673.2	Mus musculus torsin family 1, member B (Tor1b), mRNA.
scl32610.6.1_5-S	20.51	4.22	6430529G09Rik	NM_178706.2	Mus musculus RIKEN cDNA 6430529G09 gene (6430529G09Rik), mRNA.
scl46254.30.2_18-S	20.5019	2.17	Adprtl1	XM_283217.2	

scl43911.4.1_38-S	20.4932	3.27	Cxcl14	NM_019568.1	Mus musculus chemokine (C-X-C motif) ligand 14 (Cxcl14), mRNA.
scl0229543.1_170-S	20.4658	1.42	C77668	NM_145540.2	Mus musculus expressed sequence C77668 (C77668), transcript variant 1, mRNA.
scl0076080.2_277-S	20.4605	3.35	5830472M02Rik	NM_181734.1	Mus musculus RIKEN cDNA 5830472M02 gene (5830472M02Rik), mRNA.
scl066177.1_13-S	20.4495	1.18	Ubl5	NM_025401.1	Mus musculus ubiquitin-like 5 (Ubl5), mRNA.
scl0236732.21_8-S	20.4228	1.5	Rbm10	NM_145627	Mus musculus RNA binding motif protein 10 (Rbm10), mRNA.
scl022259.1_112-S	20.4079	1.49	Nr1h3	NM_013839.1	Mus musculus nuclear receptor subfamily 1, group H, member 3 (Nr1h3), mRNA.
scl26090.11.1_214-S	20.4033	1.64	9330129D05Rik	NM_178799.2	Mus musculus RIKEN cDNA 9330129D05 gene (9330129D05Rik), mRNA.
scl46570.12.1_20-S	20.3854	1.24	Vdac2	NM_011695.1	Mus musculus voltage-dependent anion channel 2 (Vdac2), mRNA.
scl23736.8.1_149-S	20.3774	1.32	1110007F05Rik	XM_355561.1	Mus musculus RIKEN cDNA 1110007F05 gene (1110007F05Rik), mRNA.
scl53358.2.1_26-S	20.3753	2.17	Ms4a6c	NM_028595	Mus musculus membrane-spanning 4-domains, subfamily A, member 6C (Ms4a6c), mRNA.
scl0234267.8_19-S	20.3753	1.46	Gpm6a	NM_153581.2	Mus musculus glycoprotein m6a (Gpm6a), mRNA.
scl00232784.2_34-S	20.3413	1.89	Zfp212	NM_145576.1	Mus musculus Zinc finger protein 212 (Zfp212), mRNA.
scl22860.4.705_3-S	20.3401	1.21	Rgmc	NM_027126.2	Mus musculus RGM domain family, member C (Rgmc), mRNA.
scl34845.7_126-S	20.306	2.87	4933409N07Rik	NM_175162.2	Mus musculus RIKEN cDNA 4933409N07 gene (4933409N07Rik), mRNA.
scl000057.1_28-S	20.3041	10.66	Nme7	NM_138314.1	Mus musculus non-metastatic cells 7, protein expressed in (Nme7), mRNA.
scl0071911.2_148-S	20.2991	1.49	Bdh	NM_175177.3	Mus musculus 3-hydroxybutyrate dehydrogenase (heart, mitochondrial) (Bdh), mRNA.
scl46562.6.1_9-S	20.2795	3.72	1700112E06Rik	XM_127589.2	Mus musculus RIKEN cDNA 1700112E06 gene (1700112E06Rik), mRNA.
scl000091.1_88-S	20.2709	1.23	Phf10	NM_024250.3	Mus musculus RIKEN cDNA 1600012H06 gene (1600012H06Rik), mRNA.
scl45062.20_217-S	20.2526	1.3	Nid1	NM_010917.1	Mus musculus nidogen 1 (Nid1), mRNA.
scl000916.1_126-S	20.2446	1.98	Pou2f1	NM_011137.1	Mus musculus POU domain, class 2, transcription factor 1 (Pou2f1), transcript variant 1, mRNA.
scl42857.13.1_22-S	20.1913	1.35	Golga5	NM_013747.2	Mus musculus golgi autoantigen, golgin subfamily a, 5 (Golga5), mRNA.
scl44991.2.1_21-S	20.164	1.25	Hist1h2bc	NM_023422	Mus musculus histone 1, H2bc (Hist1h2bc), mRNA.
scl0011832.2_50-S	20.1586	4.11	Aqp7	NM_007473.3	Mus musculus aquaporin 7 (Aqp7), mRNA.
scl055980.1_58-S	20.1428	1.44	Impa1	NM_018864.3	Mus musculus inositol (myo)-1(or 4)-monophosphatase 1 (Impa1), mRNA.
scl18714.45_17-S	20.1401	2.2	Trpm7	NM_021450.1	Mus musculus transient receptor potential cation channel, subfamily M, member 7 (Trpm7), mRNA.
scl36214.3_394-S	20.1372	1.49	Gbif	NM_019683.2	Mus musculus globin inducing factor, fetal (Gbif), mRNA.
scl52614.18.1_13-S	20.1325	1.51	D19Bwg1357e	NM_177474.2	Mus musculus DNA segment, Chr 19, Brigham & Womens Genetics 1357 expressed (D19Bwg1357e), mRNA.
scl48733.40.1_84-S	20.102	1.55	Myh11	NM_013607.1	Mus musculus myosin heavy chain 11, smooth muscle (Myh11), mRNA.
scl0003482.1_2198-S	20.0988	1.62	Tbx20	NM_194263.1	Mus musculus T-box 20 (Tbx20), transcript variant 2, mRNA.
scl36336.8_440-S	20.0975	2.86	Rpl14	NM_025974.1	Mus musculus ribosomal protein L14 (Rpl14), mRNA.
scl30534.8_189-S	20.0823	5.58	Bnip3	NM_009760.2	Mus musculus BCL2/adenovirus E1B 19kDa-interacting protein 1, NIP3 (Bnip3), mRNA.
scl0018130.2_77-S	20.0761	1.23	Ddx26	NM_008715.1	Mus musculus DEAD/H (Asp-Glu-Ala-Asp/His) box polypeptide 26 (Ddx26), mRNA.
scl0237943.1_29-S	20.0713	1.55	5430405G24Rik	XM_152907.3	Mus musculus RIKEN cDNA 5430405G24 gene (5430405G24Rik), mRNA.
scl53441.6.1_144-S	20.0514	1.18	Aip	NM_016666	Mus musculus aryl-hydrocarbon receptor-interacting protein (Aip), mRNA.
scl26742.12.1_68-S	20.0477	1.2	Eif2b4	NM_010122.1	Mus musculus eukaryotic translation initiation factor 2B, subunit 4 delta (Eif2b4), mRNA.
scl41573.28.1_3-S	20.0443	2.24	B230374F23Rik	NM_177059.2	Mus musculus RIKEN cDNA B230374F23 gene (B230374F23Rik), mRNA.
scl0056738.1_278-S	20.0432	1.65	Mocs1	NM_020042.1	Mus musculus molybdenum cofactor synthesis 1 (Mocs1), transcript variant 1, mRNA.
scl0381626.4_101-S	20.0424	1.34	6430512A10Rik	XM_355585.1	
scl20654.12_238-S	20.0414	1.32	Mtch2	NM_019758.2	Mus musculus mitochondrial carrier homolog 2 (C. elegans) (Mtch2), mRNA.
scl25518.12_3-S	20.0338	1.49	Rusc2	XM_131380.3	
scl28799.5.1_30-S	20.0279	1.37	Dguok	NM_013764.1	Mus musculus deoxyguanosine kinase (Dguok), mRNA.
scl056045.1_19-S	20.0226	2.97	Samhd1	NM_018851.2	Mus musculus SAM domain and HD domain, 1 (Samhd1), mRNA.
scl000781.1_68-S	20.0063	1.97	Kif21b	NM_019962.2	Mus musculus kinesin family member 21B (Kif21b), mRNA.
scl38403.1.6_135-S	20.0044	2.06	Lrrc10	NM_146242.1	Mus musculus leucine-rich repeat-containing 10 (Lrrc10), mRNA.
**GENES DOWN-REGULATED BY THE ATHEROGENIC DIET IN HEARTS**					
**TargetID**	**Diff_Score**	**Fold_Change**	**Symbol**	**Accession**	**Definition**
scl49148.7.844_25-S	-231.1983	-2.83	Cd80	NM_009855	Mus musculus CD80 antigen (Cd80), mRNA.
18S_rRNA_X00686_523-	-212.7893	-2.48	Pigt	NM_133779.1	Mus musculus phosphatidylinositol glycan, class T (Pigt), mRNA.
scl31105.14.1_82-S	-210.0654	-2.16	9830160G03Rik	NM_172904.1	Mus musculus RIKEN cDNA 9830160G03 gene (9830160G03Rik), mRNA.
scl0056284.1_41-S	-209.6915	-2.94	Mrpl19	NM_026490.1	Mus musculus mitochondrial ribosomal protein L19 (Mrpl19), mRNA.
scl00276770.1_2-S	-208.9187	-3.03	Eif5a	NM_181582.2	Mus musculus eukaryotic translation initiation factor 5A (Eif5a), mRNA.
scl017527.1_149-S	-204.72	-2.04	Mpv17	NM_008622.1	Mus musculus Mpv17 transgene, kidney disease mutant (Mpv17), mRNA.
scl0003580.1_5-S	-204.6237	-2.69	Tm4sf8	NM_019793.2	Mus musculus transmembrane 4 superfamily member 8 (Tm4sf8), mRNA.
scl018642.4_28-S	-200.6638	-4.27	Pfkm	NM_021514.2	Mus musculus phosphofructokinase, muscle (Pfkm), mRNA.
scl000319.1_7-S	-192.3135	-2.26	Acin1	NM_019567.1	Mus musculus apoptotic chromatin condensation inducer 1 (Acin1), mRNA.
scl020813.1_157-S	-191.9405	-1.7	Srp14	NM_009273.2	Mus musculus signal recognition particle 14 (Srp14), mRNA.

scl014470.4_28-S	-187.262	-2.01	Rabac1	NM_010261.1	Mus musculus Rab acceptor 1 (prenylated) (Rabac1), mRNA.
scl0014751.2_19-S	-177.7448	-3.13	Gpi1	NM_008155.1	Mus musculus glucose phosphate isomerase 1 (Gpi1), mRNA.
scl22501.2_29-S	-175.3556	-3.49	39340	NM_053102	Mus musculus selenoprotein (Sep15), mRNA.
scl018643.4_21-S	-175.0392	-3.89	Pfn1	NM_011072.2	Mus musculus profilin 1 (Pfn1), mRNA.
scl019173.2_9-S	-171.7794	-2.16	Psmb5	NM_011186.1	Mus musculus proteasome (prosome, macropain) subunit, beta type 5 (Psmb5), mRNA.
scl000533.1_30-S	-168.095	-1.93	Yif1	NM_026553.2	Mus musculus Yip1 interacting factor homolog (S. cerevisiae) (Yif1), mRNA.
scl34935.10.1_29-S	-166.153	-2.41	Dctn6	NM_011722.1	Mus musculus dynactin 6 (Dctn6), mRNA.
scl26802.3_29-S	-164.6109	-2.16	Fastk	NM_023229.1	Mus musculus Fas-activated serine/threonine kinase (Fastk), mRNA.
scl066480.2_3-S	-163.0832	-1.82	Rpl15	NM_025586.1	Mus musculus ribosomal protein L15 (Rpl15), mRNA.
scl24944.2_480-S	-161.9106	-1.78	BC003266	NM_030252.1	Mus musculus cDNA sequence BC003266 (BC003266), mRNA.
scl0001433.1_54-S	-161.1855	-1.99	Psmc5	NM_008950.1	Mus musculus protease (prosome, macropain) 26S subunit, ATPase 5 (Psmc5), mRNA.
scl0003742.1_4-S	-160.0903	-1.72	Tmem14c	NM_025387.1	
scl0003011.1_11-S	-159.3461	-2.84	Agpat2	NM_026212.1	Mus musculus 1-acylglycerol-3-phosphate O-acyltransferase 2 (lysophosphatidic acid acyltransferase, beta) (Agpat2), mRNA.
scl022147.3_29-S	-159.2025	-1.95	Tuba7	NM_009449.2	Mus musculus tubulin, alpha 7 (Tuba7), mRNA.
scl065973.2_37-S	-156.4666	-2.24	Asph	NM_023066.1	Mus musculus aspartate-beta-hydroxylase (Asph), mRNA.
scl26557.25_455-S	-155.8362	-3.03	Recc1	NM_011258.1	Mus musculus replication factor C 1 (Recc1), mRNA.
scl0014751.2_296-S	-155.1344	-1.58	Gpi1	NM_008155.1	Mus musculus glucose phosphate isomerase 1 (Gpi1), mRNA.
scl26467.3.1_2-S	-154.5631	-2.18	Chic2	NM_028850.1	Mus musculus cysteine-rich hydrophobic domain 2 (Chic2), mRNA.
scl00018.1_3-S	-152.9511	-2.11	Ptpre	NM_011212.2	Mus musculus protein tyrosine phosphatase, receptor type, E (Ptpre), mRNA.
scl39988.9.1_13-S	-152.8662	-1.69	Slc25a11	NM_024211.2	Mus musculus solute carrier family 25 (mitochondrial carrier; oxoglutarate carrier), member 11 (Slc25a11), mRNA.
scl40009.3.137_12-S	-151.6259	-2.24	Eif5a		
scl000176.1_12-S	-150.0554	-1.94	Psma1	NM_011965	Mus musculus proteasome (prosome, macropain) subunit, alpha type 1 (Psma1), mRNA.
scl029864.1_22-S	-149.2443	-1.81	Rnf11	NM_013876.2	Mus musculus ring finger protein 11 (Rnf11), mRNA.
scl26406.12_265-S	-144.0603	-1.74	Grsf1	NM_178700.2	Mus musculus G-rich RNA sequence binding factor 1 (Grsf1), mRNA.
scl36846.9.2_23-S	-140.3461	-2.14	Anp32a	NM_009672.2	Mus musculus acidic (leucine-rich) nuclear phosphoprotein 32 family, member A (Anp32a), mRNA.
scl40177.4_172-S	-138.491	-2.05	2410016F01Rik	NM_022423.1	
scl27366.3_38-S	-138.1552	-2.12	D5Ertd33e	NM_026504.1	Mus musculus DNA segment, Chr 5, ERATO Doi 33, expressed (D5Ertd33e), mRNA.
scl30629.5.1_26-S	-137.5654	-1.99	Vkorc1	NM_178600.2	Mus musculus vitamin K epoxide reductase complex, subunit 1 (Vkorc1), mRNA.
scl0240514.1_161-S	-137.0703	-2.3	AI842788	NM_198616.1	
scl29519.10.9_31-S	-136.7177	-2.18	Bcap37	NM_007531.1	Mus musculus B-cell receptor-associated protein 37 (Bcap37), mRNA.
scl23678.5.1_21-S	-136.2405	-1.62	3200001F09Rik	NM_027935.1	Mus musculus RIKEN cDNA 3200001F09 gene (3200001F09Rik), mRNA.
scl24492.20.1_105-S	-132.8549	-18.68	1810074P20Rik	NM_026194.2	Mus musculus RIKEN cDNA 1810074P20 gene (1810074P20Rik), mRNA.
scl17649.8_241-S	-132.0722	-1.89	4631423F02Rik	XM_129928.2	Mus musculus RIKEN cDNA 4631423F02 gene (4631423F02Rik), mRNA.
scl25185.2.323_16-S	-131.5121	-5.78	Ppap2b	NM_080555.1	Mus musculus phosphatidic acid phosphatase type 2B (Ppap2b), mRNA.
scl011867.9_22-S	-131.4458	-1.95	Arpc1b	NM_023142.1	Mus musculus actin related protein 2/3 complex, subunit 1B (Arpc1b), mRNA.
scl013179.1_84-S	-131.1051	-3.01	Dcn	NM_007833.1	Mus musculus decorin (Dcn), mRNA.
scl054673.4_1-S	-127.9836	-2.11	Sh3glb1	NM_019464.1	Mus musculus SH3-domain GRB2-like B1 (endophilin) (Sh3glb1), mRNA.
scl42936.6.9_32-S	-127.9241	-1.64	Ahsa1	NM_146036.1	Mus musculus AHA1, activator of heat shock 90kDa protein ATPase homolog 1 (yeast) (Ahsa1), mRNA.
scl00029.1_14-S	-127.4967	-1.65	9430029K10Rik	XM_284281.2	Mus musculus RIKEN cDNA 9430029K10 gene (9430029K10Rik), mRNA.
scl066916.1_19-S	-125.0458	-1.96	Ndufb7	NM_025843.1	Mus musculus NADH dehydrogenase (ubiquinone) 1 beta subcomplex, 7 (Ndufb7), mRNA.
scl47431.10_195-S	-124.7647	-2	AW549877	NM_145930	Mus musculus expressed sequence AW549877 (AW549877), mRNA.
scl068544.3_98-S	-122.3541	-1.9	2310036O22Rik	NM_026760.1	
scl0003159.1_1-S	-121.2068	-3.64	Gsn	NM_146120.2	Mus musculus gelsolin (Gsn), mRNA.
scl0017938.1_31-S	-121.0419	-2.49	Naca	NM_013608.2	Mus musculus nascent polypeptide-associated complex alpha polypeptide (Naca), mRNA.
scl0056808.2_258-S	-120.442	-6.86	Cacna2d2	NM_020263.2	Mus musculus calcium channel, voltage-dependent, alpha 2/delta subunit 2 (Cacna2d2), mRNA.
scl000646.1_33-S	-120.1567	-1.8	2310061C15Rik	NM_026844.2	
scl0268449.4_2-S	-119.8986	-1.51	Rpl23a	NM_207523	
scl000906.1_50-S	-119.1866	-2.04	Acadl	NM_007381.2	Mus musculus acetyl-Coenzyme A dehydrogenase, long-chain (Acadl), mRNA.
gi_6671508_ref_NM_007	-115.139	-1.49	Actb	NM_007393.1	Mus musculus actin, beta, cytoplasmic (Actb), mRNA.
scl0003351.1_17-S	-114.0424	-1.65	Mkks	NM_021527.1	Mus musculus McKusick-Kaufman syndrome protein (Mkks), mRNA.
scl36516.9.290_30-S	-113.6603	-1.91	Ptk9l	NM_011876.2	Mus musculus protein tyrosine kinase 9-like (A6-related protein) (Ptk9l), mRNA.
scl0016796.1_242-S	-112.9152	-2.56	Lasp1	NM_010688.2	Mus musculus LIM and SH3 protein 1 (Lasp1), mRNA.
scl44826.11.1_30-S	-112.8093	-1.59	Gmpr	NM_025508.1	Mus musculus guanosine monophosphate reductase (Gmpr), mRNA.
scl20055.4.1_10-S	-112.6293	-1.52	Dncl2a	NM_025947.1	Mus musculus dynein, cytoplasmic, light chain 2A (Dncl2a), mRNA.
scl0002832.1_59-S	-111.6119	-2.97	Ptp4a2	NM_008974.2	Mus musculus protein tyrosine phosphatase 4a2 (Ptp4a2), mRNA.
scl050795.3_8-S	-109.8487	-3.91	Sh3bgr	XM_147272.1	Mus musculus SH3-binding domain glutamic acid-rich protein (Sh3bgr), mRNA.

scl067942.2_64-S	-109.7261	-1.47	Atp5g2	NM_026468.1	Mus musculus ATP synthase, H+ transporting, mitochondrial F0 complex, subunit c (subunit 9), isoform 2 (Atp5g2), mRNA.
scl32570.6.1_25-S	-109.368	-1.64	H47	NM_024439.2	Mus musculus histocompatibility 47 (H47), mRNA.
scl011951.2_99-S	-108.7208	-1.81	Atp5g1	NM_007506.2	Mus musculus ATP synthase, H+ transporting, mitochondrial F0 complex, subunit c (subunit 9), isoform 1 (Atp5g1), mRNA.
scl29713.3_34-S	-108.5177	-1.59	Arl6ip5	NM_022992.1	Mus musculus ADP-ribosylation factor-like 6 interacting protein 5 (Arl6ip5), mRNA.
scl23453.4_99-S	-108.1653	-1.5	1200015A19Rik	NM_026388.1	Mus musculus RIKEN cDNA 1200015A19 gene (1200015A19Rik), mRNA.
scl35873.2.103_19-S	-107.6336	-1.73	Hspb2	NM_024441.1	Mus musculus heat shock protein 2 (Hspb2), mRNA.
scl35195.14.1_5-S	-106.877	-2.05	1110011D13Rik	NM_029095.1	Mus musculus RIKEN cDNA 1110011D13 gene (1110011D13Rik), mRNA.
scl068097.1_32-S	-106.1103	-3.51	6720463E02Rik	NM_026556.2	Mus musculus RIKEN cDNA 6720463E02 gene (6720463E02Rik), mRNA.
scl0002315.1_12-S	-106.0995	-10.73		XM_358429.1	Mus musculus LOC380788 (LOC380788), mRNA.
scl40850.12.1_163-S	-106.0766	-1.5	Nmt1	NM_008707.2	Mus musculus N-myristoyltransferase 1 (Nmt1), mRNA.
scl000134.1_57-S	-104.6817	-1.76	Csrp3	NM_013808.3	Mus musculus cysteine and glycine-rich protein 3 (Csrp3), mRNA.
scl00233908.1_1058-S	-104.1162	-3.13	Fus	NM_139149.1	Mus musculus fusion, derived from t(12;16) malignant liposarcoma (human) (Fus), mRNA.
scl000673.1_3-S	-102.9866	-1.5	Prdx2	NM_011563	Mus musculus peroxiredoxin 2 (Prdx2), mRNA.
scl19361.2.11_20-S	-101.6333	-1.67	Psmb7	NM_011187.1	Mus musculus proteasome (prosome, macropain) subunit, beta type 7 (Psmb7), mRNA.
scl0068349.1_0-S	-100.548	-1.49	Ndufs3	XM_130347.1	Mus musculus NADH dehydrogenase (ubiquinone) Fe-S protein 3 (Ndufs3), mRNA.
scl014683.1_3-S	-99.8804	-1.6	Gnas	NM_010309.1	Mus musculus GNAS (guanine nucleotide binding protein, alpha stimulating) complex locus (Gnas), mRNA.
scl056334.4_3-S	-99.711	-2.19	1110032D12Rik	NM_019770	Mus musculus RIKEN cDNA 1110032D12 gene (1110032D12Rik), mRNA.
scl0170826.1_122-S	-99.4383	-1.78	Ppargc1b	NM_133249	Mus musculus peroxisome proliferative activated receptor, gamma, coactivator 1 beta (Ppargc1b), mRNA.
scl21241.7.1_29-S	-98.5845	-1.75	Msrb	NM_029619.2	
scl000532.1_17-S	-96.6803	-1.6	Yif1	NM_026553.2	Mus musculus Yip1 interacting factor homolog (S. cerevisiae) (Yif1), mRNA.
scl32119.15.11_22-S	-96.3639	-1.42	Uqcrc2	NM_025899.1	Mus musculus RIKubiquinol cytochrome c reductase core protein 2 (Uqcrc2), mRNA.
scl0018220.1_39-S	-96.082	-1.98	Nucb1	NM_008749.1	
scl26807.16.1_176-S	-95.7166	-3.07	Kcnh2	NM_013569.1	Mus musculus potassium voltage-gated channel, subfamily H (eag-related), member 2 (Kcnh2), mRNA.
GI_23592945-S	-95.325	-1.44	Eef1a1	XM_134967.2	Mus musculus eukaryotic translation elongation factor 1 alpha 1 (Eef1a1), mRNA.
scl0011733.2_121-S	-95.2653	-1.84	Ank1	NM_031158.1	Mus musculus ankyrin 1, erythroid (Ank1), mRNA.
scl011816.1_11-S	-95.1942	-1.64	Apoe	NM_009696.2	Mus musculus apolipoprotein E (Apoe), mRNA.
scl30345.6_391-S	-94.5832	-1.7	Kcnd2	XM_132981.1	Mus musculus potassium voltage-gated channel, Shal-related family, member 2 (Kcnd2), mRNA.
scl46304.5.1_86-S	-93.6629	-1.73	Mrpl52	NM_026851.1	Mus musculus mitochondrial ribosomal protein L52 (Mrpl52), mRNA.
scl22956.4.1_1-S	-93.5445	-1.47	Jtb	NM_206924.1	Mus musculus jumping translocation breakpoint (Jtb), mRNA.
scl50699.8.1_77-S	-92.833	-1.54	Dscr1l1	NM_207649.1	Mus musculus Down syndrome critical region gene 1-like 1 (Dscr1l1), transcript variant 2, mRNA.
scl0013135.2_58-S	-91.9565	-1.49	Dad1	NM_010015.1	Mus musculus defender against cell death 1 (Dad1), mRNA.
scl0023881.1_86-S	-91.6782	-1.81	E430034L04Rik	NM_011816.2	Mus musculus RIKEN cDNA E430034L04 gene (E430034L04Rik), mRNA.
scl33301.5.15_0-S	-90.0233	-1.54	Nudt7	NM_024437	Mus musculus nudix (nucleoside diphosphate linked moiety X)-type motif 7 (Nudt7), mRNA.
scl070083.1_37-S	-88.7248	-1.95	Metrn	NM_133719	
scl38287.7.1_16-S	-88.6384	-1.69	Tmem4	NM_019953.1	Mus musculus transmembrane protein 4 (Tmem4), mRNA.
scl0024030.2_121-S	-88.374	-1.51	Mrps12	NM_011885.2	Mus musculus mitochondrial ribosomal protein S12 (Mrps12), mRNA.
scl019045.1_99-S	-87.9898	-1.51	Ppp1ca	NM_031868.1	Mus musculus protein phosphatase 1, catalytic subunit, alpha isoform (Ppp1ca), mRNA.
scl0014423.1_138-S	-87.6428	-1.81	Galnt1	NM_013814.2	Mus musculus UDP-N-acetyl-alpha-D-galactosamine:polypeptide N-acetylgalactosaminyltransferase 1 (Galnt1), mRNA.
scl22543.4.4_13-S	-87.0786	-1.89	Adh5	NM_007410.2	Mus musculus alcohol dehydrogenase 5 (class III), chi polypeptide (Adh5), mRNA.
scl54976.5.1_9-S	-87.0686	-1.71	Mcts1	NM_026902.1	Mus musculus malignant T cell amplified sequence 1 (Mcts1), mRNA.
scl052898.2_0-S	-86.6151	-1.45	D11Bwg0434e	NM_173742.1	Mus musculus DNA segment, Chr 11, Brigham & Womens Genetics 0434 expressed (D11Bwg0434e), mRNA.
scl058194.8_43-S	-85.9173	-1.76	Sh3kbp1	NM_021389.3	Mus musculus SH3-domain kinase binding protein 1 (Sh3kbp1), mRNA.
scl0029876.1_51-S	-85.8115	-3.92	Clic4	XM_124389.1	Mus musculus chloride intracellular channel 4 (mitochondrial) (Clic4), mRNA.
scl50151.16_294-S	-85.6291	-1.59	AI429612	XM_128606.4	Mus musculus expressed sequence AI429612 (AI429612), mRNA.
scl0066494.2_134-S	-85.3182	-1.59	2610524G07Rik	NM_025596.4	Mus musculus RIKEN cDNA 2610524G07 gene (2610524G07Rik), mRNA.
scl0002080.1_55-S	-85.0837	-2.25	39340	NM_053102.1	
scl25188.13.1_0-S	-85.0518	-2.79	C8b	NM_133882.1	Mus musculus complement component 8, beta subunit (C8b), mRNA.
scl0212111.7_156-S	-84.3487	-1.41	Inpp5a	NM_183144.1	Mus musculus inositol polyphosphate-5-phosphatase A (Inpp5a), mRNA.
scl22902.7.1_30-S	-83.8264	-1.73	Mrpl9	NM_030116.1	Mus musculus mitochondrial ribosomal protein L9 (Mrpl9), mRNA.
scl31882.2.42_7-S	-83.8049	-9.34	Cd151	NM_009842.1	Mus musculus CD151 antigen (Cd151), mRNA.
scl32994.8.1_63-S	-83.5894	-1.72	Ckm	NM_007710.1	Mus musculus creatine kinase, muscle (Ckm), mRNA.
scl24839.6.1_26-S	-83.3923	-1.6	Gcipip	NM_026780.1	Mus musculus GCIP-interacting protein p29 (Gcipip), mRNA.
scl020719.1_146-S	-83.069	-1.44	Serpinb6a	NM_009254.2	Mus musculus serine (or cysteine) proteinase inhibitor, clade B, member 6a (Serpinb6a), mRNA.
scl47204.11.1_33-S	-81.9668	-1.82	Ext1	NM_010162.1	Mus musculus exostoses (multiple) 1 (Ext1), mRNA.
scl012417.4_32-S	-81.67	-3.94	Cbx3	NM_007624	Mus musculus chromobox homolog 3 (Drosophila HP1 gamma) (Cbx3), mRNA.
scl18213.3.5_8-S	-81.266	-3.2	Eef1a2	NM_007906.2	Mus musculus eukaryotic translation elongation factor 1 alpha 2 (Eef1a2), mRNA.

scl27115.7.1_28-S	-80.7554	-1.65	Ttc11	NM_025562.1	Mus musculus tetratricopeptide repeat domain 11 (Ttc11), mRNA.
scl0001439.1_101-S	-80.1617	-2.1	Ube2g1	NM_025985.2	Mus musculus ubiquitin-conjugating enzyme E2G 1 (UBC7 homolog, C. elegans) (Ube2g1), mRNA.
scl53528.4.1_29-S	-79.8929	-1.74	Arl2	NM_019722.3	Mus musculus ADP-ribosylation factor-like 2 (Arl2), mRNA.
scl075265.1_98-S	-79.2991	-6.11	Sucla2	XM_127733.2	
scl014980.6_15-S	-79.2496	-1.52	H2-L		
scl55000.11_69-S	-78.8039	-2.02	Il13ra1	NM_133990.3	Mus musculus interleukin 13 receptor, alpha 1 (Il13ra1), mRNA.
scl0268373.4_12-S	-78.7803	-1.4	Ppia	NM_008907	Mus musculus peptidylprolyl isomerase A (Ppia), mRNA.
scl24926.6.1_18-S	-78.0072	-1.54	Ak2	NM_016895.2	Mus musculus adenylate kinase 2 (Ak2), mRNA.
scl19818.14.1_56-S	-77.7162	-5.13	Th1l	NM_020580.1	Mus musculus TH1-like homolog (Drosophila) (Th1l), mRNA.
scl0230514.5_86-S	-77.4872	-1.57	Obrgrp	NM_175036	Mus musculus leptin receptor gene-related protein (Obrgrp), mRNA.
scl017313.2_5-S	-77.4799	-1.38	Mglap	NM_008597.2	Mus musculus matrix gamma-carboxyglutamate (gla) protein (Mglap), mRNA.
scl0076789.1_86-S	-77.337	-2.36	2410129H14Rik	NM_175245.2	Mus musculus RIKEN cDNA 2410129H14 gene (2410129H14Rik), mRNA.
scl0001621.1_13-S	-76.7492	-2.15	Tcp1	NM_013686	Mus musculus t-complex protein 1 (Tcp1), mRNA.
scl056378.6_20-S	-76.731	-1.41	Arpc3	NM_019824.2	Mus musculus actin related protein 2/3 complex, subunit 3 (Arpc3), mRNA.
scl53656.13_28-S	-76.5463	-1.37	Pdha1	NM_008810.2	Mus musculus pyruvate dehydrogenase E1 alpha 1 (Pdha1), mRNA.
scl000502.1_59-S	-76.2879	-1.73	Prdx5	NM_012021.1	Mus musculus peroxiredoxin 5 (Prdx5), mRNA.
scl0234388.1_36-S	-75.8211	-1.69	1810023B24Rik	XM_134222.1	
scl018769.1_15-S	-75.6802	-1.5	Pkig	NM_011106.1	Mus musculus protein kinase inhibitor, gamma (Pkig), mRNA.
scl056348.1_132-S	-75.1873	-1.95	Hsd17b12	NM_019657.2	Mus musculus hydroxysteroid (17-beta) dehydrogenase 12 (Hsd17b12), mRNA.
scl011461.1_64-S	-74.3439	-1.65	Actb	NM_007393.1	Mus musculus actin, beta, cytoplasmic (Actb), mRNA.
scl0017105.1_34-S	-74.3065	-1.93	Lyzs	NM_017372.2	Mus musculus lysozyme (Lyzs), mRNA.
scl15816.17.1_28-S	-74.103	-1.92	Capn2	NM_009794.1	Mus musculus calpain 2 (Capn2), mRNA.
scl0002758.1_802-S	-74.0474	-4.36	Asph	NM_133723	Mus musculus aspartate-beta-hydroxylase (Asph), mRNA.
scl16816.7.1_32-S	-73.5346	-1.47	Fhl2	NM_010212	Mus musculus four and a half LIM domains 2 (Fhl2), mRNA.
scl017826.2_113-S	-73.2661	-1.62	Mtvr2	NM_023166	Mus musculus mammary tumor virus receptor 2 (Mtvr2), mRNA.
scl0002954.1_36-S	-73.0991	-1.96	Rbm3	NM_016809	Mus musculus RNA binding motif protein 3 (Rbm3), mRNA.
scl0002680.1_6-S	-72.9921	-1.48	Nsep1	NM_011732.1	Mus musculus nuclease sensitive element binding protein 1 (Nsep1), mRNA.
scl067681.3_8-S	-72.8315	-1.57	Mrpl18	NM_026310.1	Mus musculus mitochondrial ribosomal protein L18 (Mrpl18), mRNA.
scl22769.5.2_16-S	-72.8064	-2.34	Rhoc	NM_007484.1	Mus musculus ras homolog gene family, member C (Rhoc), mRNA.
scl0014700.2_203-S	-72.5874	-1.79	Gng10	NM_025277.1	Mus musculus guanine nucleotide binding protein (G protein), gamma 10 (Gng10), mRNA.
scl49150.5.1_75-S	-72.2887	-1.46	Popdc2	NM_022318.1	Mus musculus popeye domain containing 2 (Popdc2), mRNA.
scl53624.9_65-S	-72.1858	-9.19	Syap1	NM_025932.1	Mus musculus synapse associated protein 1 (Syap1), mRNA.
scl0019328.2_52-S	-72.1115	-1.94	Rab12	NM_024448.1	Mus musculus RAB12, member RAS oncogene family (Rab12), mRNA.
scl0056491.1_59-S	-72.0222	-1.47	Vapb	NM_019806.3	Mus musculus vesicle-associated membrane protein, associated protein B and C (Vapb), mRNA.
scl40772.15_67-S	-71.4067	-1.46	Prkar1a	NM_021880.1	Mus musculus protein kinase, cAMP dependent regulatory, type I, alpha (Prkar1a), mRNA.
scl33416.4.1_0-S	-70.5976	-1.7	Hspc171	NM_025486.1	
scl019988.6_33-S	-70.3694	-1.36	Rpl6	NM_011290	Mus musculus ribosomal protein L6 (Rpl6), mRNA.
scl0002841.1_13-S	-70.1321	-8.06	1200015A19Rik	NM_026388.1	Mus musculus RIKEN cDNA 1200015A19 gene (1200015A19Rik), mRNA.
scl32110.5.1_11-S	-69.1538	-1.94	0610012D09Rik	NM_021554.2	Mus musculus RIKEN cDNA 0610012D09 gene (0610012D09Rik), mRNA.
scl0002191.1_543-S	-69.0286	-1.38	Svil	NM_153153	Mus musculus supervillin (Svil), mRNA.
scl069072.8_1-S	-68.5113	-1.65	Ebna1bp2	NM_026932.2	Mus musculus EBNA1 binding protein 2 (Ebna1bp2), mRNA.
scl32129.5.1_10-S	-68.0687	-2.73	8430420C20Rik	NM_145586.1	Mus musculus RIKEN cDNA 8430420C20 gene (8430420C20Rik), mRNA.
scl19251.13.1_62-S	-67.8924	-12.12	2610014F08Rik	XM_130321.4	Mus musculus RIKEN cDNA 2610014F08 gene (2610014F08Rik), mRNA.
scl029876.1_59-S	-67.5714	-1.4	Clic4	XM_124389.1	Mus musculus chloride intracellular channel 4 (mitochondrial) (Clic4), mRNA.
scl000923.1_16-S	-67.5563	-1.7	Insig2	NM_178082.2	Mus musculus insulin induced gene 2 (Insig2), mRNA.
scl41544.8_269-S	-67.4463	-1.66	Gpx3	NM_008161.1	Mus musculus glutathione peroxidase 3 (Gpx3), mRNA.
scl0004124.1_11-S	-67.226	-4.46	Anapc5	NM_021505.1	Mus musculus anaphase-promoting complex subunit 5 (Anapc5), mRNA.
scl34300.16.8_59-S	-66.8909	-1.47	Kars	NM_053092.1	Mus musculus lysyl-tRNA synthetase (Kars), mRNA.
scl27066.10_346-S	-66.8703	-1.48	1110007L15Rik	NM_026269.1	Mus musculus RIKEN cDNA 1110007L15 gene (1110007L15Rik), mRNA.
scl017992.3_16-S	-66.798	-1.34	Ndufa4	NM_010886.1	Mus musculus NADH dehydrogenase (ubiquinone) 1 alpha subcomplex, 4 (Ndufa4), mRNA.
scl000657.1_20-S	-66.6849	-1.71	Mrps31	NM_020560.1	Mus musculus mitochondrial ribosomal protein S31 (Mrps31), mRNA.
scl0234664.3_26-S	-66.6438	-1.46	Appbp1	NM_144931.1	Mus musculus amyloid beta precursor protein binding protein 1 (Appbp1), mRNA.
scl28771.4_14-S	-66.0732	-1.72	Spr	NM_011467.1	Mus musculus sepiapterin reductase (Spr), mRNA.
scl0001294.1_4-S	-66.0203	-1.99	G6pc3	NM_175935.2	Mus musculus glucose 6 phosphatase, catalytic, 3 (G6pc3), mRNA.
scl44884.17.178_34-S	-65.5869	-5.4	Riok1	NM_024242.2	Mus musculus RIO kinase 1 (yeast) (Riok1), mRNA.
scl011740.4_112-S	-65.4192	-1.34	Slc25a5	NM_007451.2	Mus musculus solute carrier family 25 (mitochondrial carrier; adenine nucleotide translocator), member 5 (Slc25a5), mRNA.

scl022240.3_34-S	-65.1238	-2.06	Dpysl3	NM_009468.1	Mus musculus dihydropyrimidinase-like 3 (Dpysl3), mRNA.
scl49191.18.1_6-S	-64.9112	-2.16	Sema5b	XM_147227.1	Mus musculus sema domain, seven thrombospondin repeats (type 1 and type 1-like), transmembrane domain (TM) and short cytoplasmic domain, (semaphorin) 5B (Sema5b), mRNA.
scl35271.8.1_43-S	-64.756	-8.23	Cklfsf7	NM_133978.1	Mus musculus chemokine-like factor super family 7 (Cklfsf7), mRNA.
scl40859.13.1_14-S	-63.8883	-1.4	Grn	NM_008175.2	Mus musculus granulin (Grn), mRNA.
scl40144.28.1_2-S	-63.6716	-1.79	Fliih	NM_022009.1	Mus musculus flightless I homolog (Drosophila) (Fliih), mRNA.
scl0015275.2_184-S	-63.2289	-2.41	Hk1	NM_010438.1	Mus musculus hexokinase 1 (Hk1), mRNA.
scl0001745.1_1-S	-62.9916	-3.73	Flot1	NM_008027.1	Mus musculus flotillin 1 (Flot1), mRNA.
scl0073046.2_136-S	-62.9458	-1.46	2900070E19Rik	NM_028419.1	Mus musculus RIKEN cDNA 2900070E19 gene (2900070E19Rik), mRNA.
scl41029.2_494-S	-62.7939	-2.01	Tob1	NM_009427.1	Mus musculus transducer of ErbB-2.1 (Tob1), mRNA.
scl34224.8.1_50-S	-62.5045	-1.41	Cyba	NM_007806.1	Mus musculus cytochrome b-245, alpha polypeptide (Cyba), mRNA.
scl29414.11.4_112-S	-62.4456	-1.43	Strap	NM_011499.1	Mus musculus serine/threonine kinase receptor associated protein (Strap), mRNA.
scl0001607.1_37-S	-62.4294	-1.52	2810410M20Rik	NM_024428	Mus musculus RIKEN cDNA 2810410M20 gene (2810410M20Rik), mRNA.
scl019223.1_1-S	-62.3463	-1.43	Ptgis	NM_008968.2	Mus musculus prostaglandin I2 (prostacyclin) synthase (Ptgis), mRNA.
scl21503.2.1_16-S	-62.2208	-1.45	Hadhsc	NM_008212.1	Mus musculus L-3-hydroxyacyl-Coenzyme A dehydrogenase, short chain (Hadhsc), mRNA.
scl52675.8.401_6-S	-62.1572	-1.54	Ostf1	NM_017375.1	Mus musculus osteoclast stimulating factor 1 (Ostf1), mRNA.
scl012512.6_24-S	-61.2829	-1.44	Cd63	NM_007653.1	Mus musculus Cd63 antigen (Cd63), mRNA.
scl20327.6_25-S	-61.1906	-1.42	Stard7	NM_139308.1	Mus musculus START domain containing 7 (Stard7), mRNA.
scl28399.6.24_1-S	-61.0764	-1.53	Cd9	NM_007657.2	Mus musculus CD9 antigen (Cd9), mRNA.
scl00232087.1_95-S	-60.984	-2.77	Mat2a	NM_145569	Mus musculus methionine adenosyltransferase II, alpha (Mat2a), mRNA.
scl36904.3.10_19-S	-60.9475	-1.32	Cox5a	NM_007747	Mus musculus cytochrome c oxidase, subunit Va (Cox5a), mRNA.
scl0003537.1_10-S	-60.8858	-2.5	Pcolce2	NM_029620.1	Mus musculus procollagen C-endopeptidase enhancer 2 (Pcolce2), mRNA.
scl0001450.1_42-S	-60.7921	-1.76	1700113I22Rik	NM_026865	Mus musculus RIKEN cDNA 1700113I22 gene (1700113I22Rik), mRNA.
scl19155.19_403-S	-60.2787	-1.43	Slc25a12	NM_172436.2	Mus musculus solute carrier family 25 (mitochondrial carrier, Aralar), member 12 (Slc25a12), mRNA.
scl17636.2.1_21-S	-60.2765	-1.91	0710001B24Rik	NM_175118	Mus musculus RIKEN cDNA 0710001B24 gene (0710001B24Rik), mRNA.
scl054616.1_2-S	-60.044	-1.63	Extl3	NM_018788.2	Mus musculus exostoses (multiple)-like 3 (Extl3), mRNA.
scl019042.1_95-S	-60.0315	-1.86	Ppm1a	NM_008910.2	Mus musculus protein phosphatase 1A, magnesium dependent, alpha isoform (Ppm1a), mRNA.
scl0074026.1_154-S	-60.0088	-1.48	4121402D02Rik	NM_028722.1	Mus musculus RIKEN cDNA 4121402D02 gene (4121402D02Rik), mRNA.
scl34655.25.1_28-S	-59.913	-1.43	Eps15-rs	NM_007944.1	Mus musculus epidermal growth factor receptor pathway substrate 15, related sequence (Eps15-rs), mRNA.
scl37045.17.1_33-S	-59.8917	-1.52	Usp2	NM_198091.1	Mus musculus ubiquitin specific protease 2 (Usp2), transcript variant 2, mRNA.
scl0019663.2_128-S	-59.8699	-1.47	Rbpms	NM_019733.1	Mus musculus RNA binding protein gene with multiple splicing (Rbpms), mRNA.
scl0058239.1_184-S	-59.79	-2.61	Dexi	NM_021428.3	Mus musculus dexamethasone-induced transcript (Dexi), mRNA.
scl0002222.1_6-S	-59.6781	-1.39	Lims2	NM_144862.1	Mus musculus LIM and senescent cell antigen like domains 2 (Lims2), mRNA.
scl0001801.1_3-S	-59.5225	-1.37	Dscr5	NM_019543.2	Mus musculus Down syndrome critical region homolog 5 (human) (Dscr5), mRNA.
scl0328601.1_103-S	-59.5225	-1.5	Ccnt1	XM_286697.2	Mus musculus cyclin T1 (Ccnt1), mRNA.
scl0023807.1_130-S	-59.5194	-1.42	Arih2	NM_011790.2	Mus musculus ariadne homolog 2 (Drosophila) (Arih2), mRNA.
scl026441.8_13-S	-59.5017	-1.43	Psma4	NM_011966	Mus musculus proteasome (prosome, macropain) subunit, alpha type 4 (Psma4), mRNA.
scl41225.47_412-S	-59.3369	-1.57	Myo18a	NM_011586.1	Mus musculus myosin XVIIIa (Myo18a), mRNA.
scl45884.3_67-S	-59.2528	-2.16	Lrrc3b	NM_146052.2	
scl0001631.1_196-S	-59.2396	-1.92	Rpo1-1	NM_009085.1	Mus musculus RNA polymerase 1-1 (Rpo1-1), mRNA.
scl0003623.1_49-S	-59.1614	-2.95	Pfkp	NM_019703.2	Mus musculus phosphofructokinase, platelet (Pfkp), mRNA.
scl43914.11.4_21-S	-58.9848	-2.72	H2afy	NM_012015.1	Mus musculus H2A histone family, member Y (H2afy), mRNA.
scl25288.8_80-S	-58.9794	-2.92	Mtap	NM_024433.1	Mus musculus methylthioadenosine phosphorylase (Mtap), mRNA.
scl0053378.2_54-S	-58.9359	-2.44	Sdcbp	NM_016807.1	Mus musculus syndecan binding protein (Sdcbp), mRNA.
scl066589.4_10-S	-58.4174	-2.43	Ube2v1	NM_023230.1	Mus musculus ubiquitin-conjugating enzyme E2 variant 1 (Ube2v1), mRNA.
scl012700.3_170-S	-58.3809	-1.78	Cish	NM_009895.2	Mus musculus cytokine inducible SH2-containing protein (Cish), mRNA.
scl0098878.2_247-S	-58.3486	-1.37	Ehd4	NM_133838.2	Mus musculus EH-domain containing 4 (Ehd4), mRNA.
scl015040.2_130-S	-58.345	-1.56	H2-T23	NM_010398	Mus musculus histocompatibility 2, T region locus 23 (H2-T23), mRNA.
scl0012450.2_17-S	-58.1918	-1.42	Ccng1	NM_009831.1	Mus musculus cyclin G1 (Ccng1), mRNA.
scl0078920.2_103-S	-57.9661	-1.36	Dlst	NM_030225.3	Mus musculus dihydrolipoamide S-succinyltransferase (E2 component of 2-oxo-glutarate complex) (Dlst), mRNA.
scl020638.2_15-S	-57.8901	-1.96	Snrpb	NM_009225.1	Mus musculus small nuclear ribonucleoprotein B (Snrpb), mRNA.
scl0022213.2_69-S	-57.6849	-8.11	Ube2g2	NM_019803.2	Mus musculus ubiquitin-conjugating enzyme E2G 2 (Ube2g2), mRNA.
scl0001344.1_14-S	-57.4981	-6.63	4930579A11Rik	NM_029478.2	Mus musculus RIKEN cDNA 4930579A11 gene (4930579A11Rik), mRNA.
scl49476.8.9_25-S	-57.2789	-1.51	Hmox2	NM_010443.1	Mus musculus heme oxygenase (decycling) 2 (Hmox2), mRNA.
scl54227.4_188-S	-57.2465	-1.5	Tmem32	NM_146234.2	
scl48911.16.3_18-S	-56.7814	-2.67	Usp16	NM_024258.1	Mus musculus ubiquitin specific protease 16 (Usp16), mRNA.
scl0002187.1_532-S	-56.6275	-1.41	Rab18	NM_181070.2	Mus musculus RAB18, member RAS oncogene family (Rab18), mRNA.

scl0245688.1_74-S	-56.5537	-9.65	Rbbp7	NM_009031.2	Mus musculus retinoblastoma binding protein 7 (Rbbp7), mRNA.
gi_6679936_ref_NM_008(	-56.5098	-1.52			
scl0001137.1_14-S	-56.4962	-1.8	Mrps25	NM_025578.2	Mus musculus mitochondrial ribosomal protein S25 (Mrps25), mRNA.
scl51225.7.1_1-S	-56.0459	-2.26	1110032A13Rik	NM_199197.1	Mus musculus RIKEN cDNA 1110032A13 gene (1110032A13Rik), mRNA.
scl15941.6.1_27-S	-56.0314	-1.6	Ndufs2	NM_153064.3	Mus musculus NADH dehydrogenase (ubiquinone) Fe-S protein 2 (Ndufs2), mRNA.
scl50948.6.850_26-S	-55.9666	-1.39	Atp6v0e	NM_025272.1	Mus musculus ATPase, H+ transporting, V0 subunit (Atp6v0e), mRNA.
scl24605.2.1_63-S	-55.8384	-1.38	0610033H09Rik	NM_025338.1	Mus musculus RIKEN cDNA 0610033H09 gene (0610033H09Rik), mRNA.
scl000333.1_26-S	-55.1878	-7.26	Tgfb1i4	NM_009366.1	Mus musculus transforming growth factor beta 1 induced transcript 4 (Tgfb1i4), mRNA.
scl00211378.1_156-S	-55.015	-9.86	6720489N17Rik	XM_142448	Mus musculus RIKEN cDNA 6720489N17 gene (6720489N17Rik), mRNA.
scl000245.1_108-S	-55.0086	-2.46	Irf3	NM_016849.2	Mus musculus interferon regulatory factor 3 (Irf3), mRNA.
scl0319184.1_311-S	-54.7894	-1.58	Hist1h2bk	NM_175665.1	Mus musculus histone 1, H2bk (Hist1h2bk), mRNA.
scl059042.1_5-S	-54.5164	-1.61	Cope	NM_021538.1	Mus musculus coatomer protein complex, subunit epsilon (Cope), mRNA.
scl057785.2_9-S	-54.511	-1.57	Rangnrf	NM_021329.1	Mus musculus RAN guanine nucleotide release factor (Rangnrf), mRNA.
scl0001591.1_7-S	-54.3187	-1.62	Hnrph1	NM_021510.1	Mus musculus heterogeneous nuclear ribonucleoprotein H1 (Hnrph1), mRNA.
scl18232.3.7_30-S	-54.2221	-1.33	Psma7	NM_011969.1	Mus musculus proteasome (prosome, macropain) subunit, alpha type 7 (Psma7), mRNA.
scl40837.7_339-S	-54.092	-1.39	Arf2	NM_007477.2	Mus musculus ADP-ribosylation factor 2 (Arf2), mRNA.
scl46751.13_99-S	-53.8089	-1.38	Prkag1	NM_016781	Mus musculus protein kinase, AMP-activated, gamma 1 non-catalytic subunit (Prkag1), mRNA.
scl00071.1_7-S	-53.7464	-1.48	Psmd13	NM_011875	Mus musculus proteasome (prosome, macropain) 26S subunit, non-ATPase, 13 (Psmd13), mRNA.
scl39081.11_80-S	-53.7194	-1.5	Stx7	NM_016797.2	Mus musculus syntaxin 7 (Stx7), mRNA.
scl0056307.2_142-S	-53.4806	-1.41	Metap2	NM_019648.2	Mus musculus methionine aminopeptidase 2 (Metap2), mRNA.
scl18853.7.1_146-S	-53.2249	-1.38	Actc1	NM_009608.1	Mus musculus actin, alpha, cardiac (Actc1), mRNA.
scl53350.20_138-S	-53.1554	-2.4	AV312086	NM_172635.1	Mus musculus expressed sequence AV312086 (AV312086), mRNA.
scl46489.21.1_17-S	-53.1278	-1.76	Capn7	NM_009796.1	Mus musculus calpain 7 (Capn7), mRNA.
scl34581.9.1_48-S	-53.0869	-1.32	Gpsn2	NM_134118.1	Mus musculus glycoprotein, synaptic 2 (Gpsn2), mRNA.
scl0215494.1_125-S	-52.6969	-1.64	C85492	NM_153540.2	Mus musculus expressed sequence C85492 (C85492), mRNA.
scl52314.6.1_5-S	-52.6631	-1.31	Prdx3	NM_007452.1	Mus musculus peroxiredoxin 3 (Prdx3), mRNA.
scl0068395.1_77-S	-52.6163	-2.98	0610037M15Rik		
scl0004061.1_14-S	-52.5791	-1.98	Mpv17	NM_008622.1	Mus musculus Mpv17 transgene, kidney disease mutant (Mpv17), mRNA.
scl39607.11_182-S	-52.5736	-2.29	Smarce1	NM_020618.3	Mus musculus SWI/SNF related, matrix associated, actin dependent regulator of chromatin, subfamily e, member 1 (Smarce1), mRNA.
scl0012443.2_42-S	-52.5056	-2.61	Ccnd1	NM_007631.1	Mus musculus cyclin D1 (Ccnd1), mRNA.
scl40889.7.141_9-S	-52.4878	-1.4	D11Wsu68e	NM_026776.3	Mus musculus DNA segment, Chr 11, Wayne State University 68, expressed (D11Wsu68e), mRNA.
scl0016195.2_10-S	-52.4664	-3.82	Il6st	NM_010560.2	Mus musculus interleukin 6 signal transducer (Il6st), mRNA.
scl38444.3.1_70-S	-52.371	-1.89	Phlda1	NM_009344.1	Mus musculus pleckstrin homology-like domain, family A, member 1 (Phlda1), mRNA.
scl0003838.1_10-S	-52.3352	-1.85	Pcmt1	NM_008786.1	Mus musculus protein-L-isoaspartate (D-aspartate) O-methyltransferase 1 (Pcmt1), mRNA.
scl36121.3_28-S	-52.3165	-1.6	1110011K10Rik	NM_170777.3	Mus musculus RIKEN cDNA 1110011K10 gene (1110011K10Rik), mRNA.
scl0004181.1_42-S	-52.1847	-1.56	Vps29	NM_019780.1	Mus musculus vacuolar protein sorting 29 (S. pombe) (Vps29), mRNA.
scl00100273.2_58-S	-52.1464	-1.31	Osbpl9	NM_133885.1	Mus musculus oxysterol binding protein-like 9 (Osbpl9), mRNA.
scl066108.3_20-S	-52.0677	-1.42	Ndufa9	NM_025358.1	Mus musculus NADH dehydrogenase (ubiquinone) 1 alpha subcomplex, 9 (Ndufa9), mRNA.
scl0192292.18_41-S	-52.04	-2.53	Nrbp	NM_147201.1	Mus musculus nuclear receptor binding protein (Nrbp), mRNA.
scl067959.1_232-S	-51.8701	-1.33	2410104I19Rik	NM_133691.1	Mus musculus RIKEN cDNA 2410104I19 gene (2410104I19Rik), mRNA.
scl28784.6_160-S	-51.7306	-1.33	Tex261	NM_009357.1	Mus musculus testis expressed gene 261 (Tex261), mRNA.
scl34280.9.1_28-S	-51.6307	-1.48	2310061C15Rik	NM_026844.2	
scl0004033.1_10-S	-51.4937	-1.63	Arpc1a	NM_019767.1	Mus musculus actin related protein 2/3 complex, subunit 1A (Arpc1a), mRNA.
scl071452.5_105-S	-51.3615	-1.31	5530600A18Rik	NM_027799.1	Mus musculus RIKEN cDNA 5530600A18 gene (5530600A18Rik), mRNA.
scl40361.13.1_2-S	-50.9818	-1.79	Rars	NM_025936.1	Mus musculus arginyl-tRNA synthetase (Rars), mRNA.
scl0073834.1_147-S	-50.8948	-1.69	Atp6v1d	NM_023721.1	Mus musculus ATPase, H+ transporting, V1 subunit D (Atp6v1d), mRNA.
scl024075.4_0-S	-50.8492	-1.4	Taf10	NM_020024.3	Mus musculus TAF10 RNA polymerase II, TATA box binding protein (TBP)-associated factor (Taf10), mRNA.
scl0272396.21_80-S	-50.7526	-2.09	A530046H20Rik	NM_172310.1	Mus musculus RIKEN cDNA A530046H20 gene (A530046H20Rik), mRNA.
scl000534.1_21-S	-50.6741	-1.5	Ppp1ca	NM_031868.1	Mus musculus protein phosphatase 1, catalytic subunit, alpha isoform (Ppp1ca), mRNA.
scl0001044.1_79-S	-50.385	-1.72	Arl6ip5	NM_022992.1	Mus musculus ADP-ribosylation factor-like 6 interacting protein 5 (Arl6ip5), mRNA.
scl0059013.2_121-S	-50.3736	-3.07	Hnrph1	NM_021510.1	Mus musculus heterogeneous nuclear ribonucleoprotein H1 (Hnrph1), mRNA.
scl017938.7_45-S	-50.1689	-1.48	Naca	NM_013608.2	Mus musculus nascent polypeptide-associated complex alpha polypeptide (Naca), mRNA.
scl51485.9_146-S	-50.0442	-1.48	Fgf1	NM_010197.2	Mus musculus fibroblast growth factor 1 (Fgf1), mRNA.
scl00114663.2_25-S	-50.0374	-7.54	Impa2	NM_053261.1	Mus musculus inositol (myo)-1(or 4)-monophosphatase 2 (Impa2), mRNA.
scl019921.5_131-S	-49.9083	-1.51	Rpl19	NM_009078	Mus musculus ribosomal protein L19 (Rpl19), mRNA.
scl054170.29_181-S	-49.8139	-1.35	Rragc	NM_017475	Mus musculus Ras-related GTP binding C (Rragc), mRNA.

scl0003291.1_0-S	-49.6173	-1.57	Arfrp1	NM_029702.2	Mus musculus ADP-ribosylation factor related protein 1 (Arfrp1), mRNA.
scl36048.5_11-S	-49.613	-2.18	BC024806	NM_172291.1	Mus musculus cDNA sequence BC024806 (BC024806), mRNA.
scl0003638.1_65-S	-49.2536	-1.48	2310016C16Rik	NM_027127.1	Mus musculus RIKEN cDNA 2310016C16 gene (2310016C16Rik), mRNA.
scl32057.1.564_6-S	-49.2458	-1.5	1110025L05Rik	NM_175103.2	Mus musculus RIKEN cDNA 1110025L05 gene (1110025L05Rik), mRNA.
scl35242.18_241-S	-49.2385	-1.63	Rbms3	NM_178660.2	
scl066475.2_30-S	-49.238	-1.5	Rps23	NM_024175	Mus musculus ribosomal protein S23 (Rps23), mRNA.
scl52799.1_8-S	-49.0462	-1.8	Doc2g	NM_021791.2	Mus musculus double C2, gamma (Doc2g), mRNA.
scl0019171.2_204-S	-48.8397	-1.54	Psmb10	NM_013640.1	Mus musculus proteasome (prosome, macropain) subunit, beta type 10 (Psmb10), mRNA.
scl30230.21.1_91-S	-48.4274	-2.07	Sec8l1	NM_009148.1	Mus musculus SEC8-like 1 (S. cerevisiae) (Sec8l1), mRNA.
scl000639.1_71-S	-48.3555	-2.17	1110017C15Rik	NM_025391.1	Mus musculus RIKEN cDNA 1110017C15 gene (1110017C15Rik), mRNA.
scl0002710.1_8-S	-48.3448	-9.37	Mfn2	NM_133201.1	Mus musculus mitofusin 2 (Mfn2), mRNA.
scl016909.6_211-S	-48.2955	-1.56	Lmo2	NM_008505.3	Mus musculus LIM domain only 2 (Lmo2), mRNA.
scl0229279.5_20-S	-48.2557	-2.85	2610510D13Rik	NM_146130	
scl32819.7.1_18-S	-48.1846	-6.52	AI839550	NM_198027.2	Mus musculus expressed sequence AI839550 (AI839550), mRNA.
scl46932.19_335-S	-48.084	-1.42	Rangap1	NM_011241	Mus musculus RAN GTPase activating protein 1 (Rangap1), mRNA.
scl30702.10.1_59-S	-48.052	-1.44	2510027N19Rik	NM_026330.2	Mus musculus RIKEN cDNA 2510027N19 gene (2510027N19Rik), mRNA.
scl016828.6_330-S	-47.6926	-1.65	Ldh1	NM_010699.1	Mus musculus lactate dehydrogenase 1, A chain (Ldh1), mRNA.
scl072139.1_117-S	-47.5263	-3.79	2610044O15Rik	NM_153780.1	Mus musculus RIKEN cDNA 2610044O15 gene (2610044O15Rik), mRNA.
scl51719.2_118-S	-47.4774	-1.5	Zadh2	NM_146090.2	
scl15964.10_1-S	-47.3423	-1.42	Hsd17b7	NM_010476.2	Mus musculus hydroxysteroid (17-beta) dehydrogenase 7 (Hsd17b7), mRNA.
scl0002112.1_25-S	-47.251	-1.62	1110001A05Rik	NM_019809	Mus musculus RIKEN cDNA 1110001A05 gene (1110001A05Rik), mRNA.
scl017762.10_59-S	-47.1859	-1.61	Mapt	NM_010838.2	Mus musculus microtubule-associated protein tau (Mapt), mRNA.
scl0002467.1_77-S	-47.0292	-1.48	Bzrp	NM_009775.2	Mus musculus benzodiazepine receptor, peripheral (Bzrp), mRNA.
scl37659.18.259_11-S	-46.8877	-1.28	Tra1	NM_011631.1	Mus musculus tumor rejection antigen gp96 (Tra1), mRNA.
scl020655.4_35-S	-46.6165	-1.3	Sod1	NM_011434.1	Mus musculus superoxide dismutase 1, soluble (Sod1), mRNA.
scl0218490.3_0-S	-46.6021	-1.35	Btf3	NM_145455.1	Mus musculus basic transcription factor 3 (Btf3), mRNA.
scl019942.4_1-S	-46.5943	-1.33	Rpl27	NM_011289	Mus musculus ribosomal protein L27 (Rpl27), mRNA.
scl0001335.1_10-S	-46.5879	-1.51	BC005682	NM_033562.2	Mus musculus cDNA sequence BC005682 (BC005682), mRNA.
scl27180.9.1_11-S	-46.5628	-1.54	Gbas	NM_008095.1	Mus musculus glioblastoma amplified sequence (Gbas), mRNA.
scl0019056.2_142-S	-46.5578	-1.3	Ppp3cb	NM_008914.1	Mus musculus protein phosphatase 3, catalytic subunit, beta isoform (Ppp3cb), mRNA.
scl0012793.2_38-S	-46.5169	-1.42	Cnih	NM_009919.1	Mus musculus cornichon homolog (Drosophila) (Cnih), mRNA.
scl39231.4_382-S	-46.3833	-1.31	BC003940	XM_203523.2	Mus musculus cDNA sequence BC003940 (BC003940), mRNA.
scl36684.10_99-S	-45.9181	-1.44	Elovl5	NM_134255.2	Mus musculus ELOVL family member 5, elongation of long chain fatty acids (yeast) (Elovl5), mRNA.
scl36793.6.1_16-S	-45.6704	-1.47	Ppib	NM_011149.1	Mus musculus peptidylprolyl isomerase B (Ppib), mRNA.
scl21088.14_336-S	-45.6031	-1.68	Ppp2r4	NM_138748.2	Mus musculus protein phosphatase 2A, regulatory subunit B (PR 53) (Ppp2r4), mRNA.
scl0018590.2_128-S	-45.593	-1.62	Pdgfa	NM_008808.2	Mus musculus platelet derived growth factor, alpha (Pdgfa), mRNA.
scl33257.7.1_7-S	-45.562	-2.55	Wfdc1	NM_023395.1	Mus musculus WAP four-disulfide core domain 1 (Wfdc1), mRNA.
scl45839.23_390-S	-45.5368	-1.61	Camk2g	NM_178597.2	Mus musculus calcium/calmodulin -dependent protein kinase II gamma (Camk2g), mRNA.
scl00015.1_21-S	-45.4878	-1.28	Rabac1	NM_010261.1	Mus musculus Rab acceptor 1 (prenylated) (Rabac1), mRNA.
scl053607.7_2-S	-45.4437	-2.49	Snrpa	NM_015782.2	Mus musculus small nuclear ribonucleoprotein polypeptide A (Snrpa), mRNA.
scl37745.17_21-S	-45.3859	-1.48	Thrap5	NM_198107.1	Mus musculus thyroid hormone receptor associated protein 5 (Thrap5), mRNA.
scl020810.7_29-S	-45.0393	-4.44	Srm	NM_009272.2	Mus musculus spermidine synthase (Srm), mRNA.
scl0057296.1_256-S	-44.9858	-1.54	Psmd8	NM_026545.1	Mus musculus proteasome (prosome, macropain) 26S subunit, non-ATPase, 8 (Psmd8), mRNA.
scl000386.1_25-S	-44.6064	-3.01	1110008L20Rik	NM_028643.1	Mus musculus RIKEN cDNA 1110008L20 gene (1110008L20Rik), mRNA.
scl020280.7_0-S	-44.5067	-2.05	Scp2	NM_011327.1	Mus musculus sterol carrier protein 2, liver (Scp2), mRNA.
scl45706.11_2-S	-44.4254	-1.32	Ghitm	NM_078478.1	Mus musculus growth hormone inducible transmembrane protein (Ghitm), mRNA.
scl31031.8.1_28-S	-44.3319	-1.46	1810020D17Rik	NM_183251.2	Mus musculus RIKEN cDNA 1810020D17 gene (1810020D17Rik), mRNA.
scl094091.6_116-S	-44.3067	-1.94	Trim11	NM_053168.1	Mus musculus tripartite motif protein 11 (Trim11), mRNA.
scl0053379.2_22-S	-44.2233	-2.02	Hnrpa2b1	NM_182650.2	Mus musculus heterogeneous nuclear ribonucleoprotein A2/B1 (Hnrpa2b1), transcript variant 2, mRNA.
scl23697.3.1_58-S	-44.2005	-2.17	Sh3bgrl3	NM_080559.1	Mus musculus SH3 domain binding glutamic acid-rich protein-like 3 (Sh3bgrl3), mRNA.
scl000708.1_100-S	-44.1369	-1.98	Atp6v0d1	NM_013477.2	Mus musculus ATPase, H+ transporting, V0 subunit D isoform 1 (Atp6v0d1), mRNA.
scl32956.7.1_6-S	-44.0614	-1.6	Ethe1	NM_023154.2	Mus musculus ethylmalonic encephalopathy 1 (Ethe1), mRNA.
scl28804.3_1-S	-43.9814	-2.75	Wbp1	NM_016757.1	Mus musculus WW domain binding protein 1 (Wbp1), mRNA.
scl0104457.3_1-S	-43.4985	-2.88	0610010K14Rik	NM_026757.1	Mus musculus RIKEN cDNA 0610010K14 gene (0610010K14Rik), mRNA.
scl31668.16.1_1-S	-43.4449	-5.59	Lu	NM_020486.1	Mus musculus Lutheran blood group (Auberger b antigen included) (Lu), mRNA.
scl30720.15_54-S	-43.1602	-1.39	Gga2	XM_133801.3	Mus musculus golgi associated, gamma adaptin ear containing, ARF binding protein 2 (Gga2), mRNA.

scl0071949.2_8-S	-43.104	-1.68	Lass5	NM_028015.2	Mus musculus longevity assurance homolog 5 (S. cerevisiae) (Lass5), mRNA.
scl0003622.1_15-S	-42.7568	-2.01	Nqo2	NM_020282.2	Mus musculus NAD(P)H dehydrogenase, quinone 2 (Nqo2), mRNA.
scl055949.3_17-S	-42.6355	-1.26	Eef1b2	NM_018796.2	Mus musculus eukaryotic translation elongation factor 1 beta 2 (Eef1b2), mRNA.
scl068377.10_23-S	-42.5747	-2.05	Acta2	NM_007392.2	Mus musculus RIKEN cDNA 0610041G09 gene (0610041G09Rik), mRNA.
scl0068045.1_0-S	-42.4888	-2.48	2700060E02Rik	NM_026528.1	Mus musculus RIKEN cDNA 2700060E02 gene (2700060E02Rik), mRNA.
scl0002269.1_18-S	-42.4271	-6.92	Matr3	NM_010771.3	Mus musculus matrin 3 (Matr3), mRNA.
scl020218.6_1-S	-42.3334	-5.41	Khdrbs1	NM_011317.2	Mus musculus KH domain containing, RNA binding, signal transduction associated 1 (Khdrbs1), mRNA.
scl0014670.2_304-S	-42.313	-1.62	Gna-rs1	NM_008136.1	Mus musculus guanine nucleotide binding protein, related sequence 1 (Gna-rs1), mRNA.
scl38956.10_80-S	-42.2991	-1.55	Rtn4ip1	NM_130892.2	Mus musculus reticulon 4 interacting protein 1 (Rtn4ip1), mRNA.
scl0068272.1_99-S	-42.2905	-1.65	Rbm28	NM_133925.1	
scl000468.1_25-S	-42.2891	-1.42	Mrpl43	NM_053164.2	Mus musculus mitochondrial ribosomal protein L43 (Mrpl43), nuclear gene encoding mitochondrial protein, mRNA.
scl0002320.1_23-S	-42.2292	-3.04	Adssl1	NM_007421.1	
scl012462.9_0-S	-42.2179	-1.57	Cct3	NM_009836.1	Mus musculus chaperonin subunit 3 (gamma) (Cct3), mRNA.
scl40689.20_176-S	-42.1985	-4.49	39334	NM_017380.1	Mus musculus septin 9 (Sept9), mRNA.
scl19720.5_23-S	-42.1338	-1.95	Echdc3	NM_024208.3	Mus musculus enoyl Coenzyme A hydratase domain containing 3 (Echdc3), mRNA.
scl014693.1_109-S	-42.1151	-1.39	Gnb2	NM_010312.3	Mus musculus guanine nucleotide binding protein, beta 2 (Gnb2), mRNA.
scl42385.21_636-S	-42.1053	-2.54	Sos2	XM_127051.3	Mus musculus Son of sevenless homolog 2 (Drosophila) (Sos2), mRNA.
scl0001329.1_110-S	-42.0705	-1.44	0610009H04Rik	NM_018854.3	Mus musculus RIKEN cDNA 0610009H04 gene (0610009H04Rik), mRNA.
scl000679.1_109-S	-42.0523	-2.04	1810045K07Rik	NM_026432.2	Mus musculus RIKEN cDNA 1810045K07 gene (1810045K07Rik), mRNA.
scl068077.3_0-S	-42.002	-1.5	Gltscr2	NM_133831.1	Mus musculus glioma tumor suppressor candidate region gene 2 (Gltscr2), mRNA.
scl52796.6.1_4-S	-41.953	-1.55	Acy3	NM_027857.2	
scl0003098.1_1-S	-41.9396	-1.41	Snrpb	NM_009225.1	Mus musculus small nuclear ribonucleoprotein B (Snrpb), mRNA.
scl23887.17.1_103-S	-41.7931	-1.33	Ctps	NM_016748.1	Mus musculus cytidine 5-triphosphate synthase (Ctps), mRNA.
scl0053607.2_0-S	-41.771	-2.4	Snrpa	NM_015782.2	Mus musculus small nuclear ribonucleoprotein polypeptide A (Snrpa), mRNA.
scl026893.9_4-S	-41.5601	-1.27	Cops6	NM_012002.1	Mus musculus COP9 (constitutive photomorphogenic) homolog, subunit 6 (Arabidopsis thaliana) (Cops6), mRNA.
scl0001574.1_5-S	-41.1872	-4.64	G3bp	NM_013716.1	Mus musculus Ras-GTPase-activating protein SH3-domain binding protein (G3bp), mRNA.
scl31033.2.1_0-S	-41.1378	-3.68	Thrsp	NM_009381.2	Mus musculus thyroid hormone responsive SPOT14 homolog (Rattus) (Thrsp), mRNA.
scl014790.2_94-S	-41.1153	-1.28	Grcc10	NM_013535.1	Mus musculus gene rich cluster, C10 gene (Grcc10), mRNA.
scl27505.17_595-S	-41.0973	-1.35	Pkd2	NM_008861.2	Mus musculus polycystic kidney disease 2 (Pkd2), mRNA.
scl33423.6_118-S	-41.0652	-1.4	Cbfb	NM_022309.2	Mus musculus core binding factor beta (Cbfb), mRNA.
scl0001255.1_2-S	-40.9315	-6.15	2500002L14Rik	NM_025607.2	Mus musculus RIKEN cDNA 2500002L14 gene (2500002L14Rik), mRNA.
scl0234852.1_50-S	-40.9038	-1.32	Pcoln3	NM_145606.1	Mus musculus procollagen (type III) N-endopeptidase (Pcoln3), mRNA.
scl25011.1.118_77-S	-40.8935	-1.56	Cited4	NM_019563	Mus musculus Cbp/p300-interacting transactivator, with Glu/Asp-rich carboxy-terminal domain, 4 (Cited4), mRNA.
scl0018938.1_81-S	-40.8915	-1.44	Ppp1r14b	NM_008889.1	Mus musculus protein phosphatase 1, regulatory (inhibitor) subunit 14B (Ppp1r14b), mRNA.
scl000986.1_622-S	-40.8297	-1.35	Insig2	NM_133748.1	Mus musculus insulin induced gene 2 (Insig2), mRNA.
scl0056304.1_278-S	-40.7637	-2.1	LOC56304	NM_019633.1	Mus musculus recombinant antineuraminidase single chain Ig VH and VL domains (LOC56304), mRNA.
scl067105.6_6-S	-40.6689	-1.96	1700034H14Rik	NM_025969.1	Mus musculus RIKEN cDNA 1700034H14 gene (1700034H14Rik), mRNA.
scl00223455.2_26-S	-40.6484	-2.04	F830029L24Rik	NM_172606.1	Mus musculus RIKEN cDNA F830029L24 gene (F830029L24Rik), mRNA.
scl0070396.1_30-S	-40.6097	-1.67	2210409M21Rik	NM_133728.2	Mus musculus RIKEN cDNA 2210409M21 gene (2210409M21Rik), mRNA.
scl016012.5_14-S	-40.5851	-2.06	Igfbp6	NM_008344.1	Mus musculus insulin-like growth factor binding protein 6 (Igfbp6), mRNA.
scl34048.16.227_13-S	-40.5664	-1.68	Cdc16	NM_027276.1	Mus musculus CDC16 cell division cycle 16 homolog (S. cerevisiae) (Cdc16), mRNA.
scl015510.1_74-S	-40.4878	-1.25	Hspd1	NM_010477.2	Mus musculus heat shock protein 1 (chaperonin) (Hspd1), mRNA.
scl020443.1_125-S	-40.4709	-3.02	Siat4c	NM_009178.2	Mus musculus sialyltransferase 4C (beta-galactoside alpha-2,3-sialytransferase) (Siat4c), mRNA.
scl46411.5.1_45-S	-40.4695	-1.8	1810009H17Rik	XM_283205.1	
scl0077975.2_18-S	-40.4384	-1.48	B230114J08Rik	NM_030018.2	Mus musculus RIKEN cDNA B230114J08 gene (B230114J08Rik), mRNA.
scl50509.4_211-S	-40.419	-2.07	Lbh	NM_029999.3	Mus musculus limb-bud and heart (Lbh), mRNA.
scl0319710.13_85-S	-40.3884	-1.64	4930488L10Rik	NM_028127.3	Mus musculus RIKEN cDNA 4930488L10 gene (4930488L10Rik), mRNA.
scl0002942.1_1346-S	-40.2196	-2.09	Mic2l1	NM_138309.1	Mus musculus MIC2 (monoclonal Imperial Cancer Research Fund 2)-like 1 (Mic2l1), mRNA.
scl47780.3.9_1-S	-40.205	-1.25	Rpl8	NM_012053.1	Mus musculus ribosomal protein L8 (Rpl8), mRNA.
scl39110.7.1_195-S	-40.1028	-3.12	E230031K19	NM_172786.1	Mus musculus hypothetical protein E230031K19 (E230031K19), mRNA.
scl0077038.2_139-S	-40.0938	-1.46	Zfp289	NM_023854.1	Mus musculus zinc finger protein 289 (Zfp289), mRNA.
scl068436.2_19-S	-40.05	-1.36	1100001I22Rik	XM_485311	
scl20983.4_177-S	-39.9983	-1.44	Arpc5l	NM_028809.1	Mus musculus actin related protein 2/3 complex, subunit 5-like (Arpc5l), mRNA.
scl014870.5_30-S	-39.998	-1.26	Gstp1	NM_013541.1	Mus musculus glutathione S-transferase, pi 1 (Gstp1), mRNA.
scl000645.1_1-S	-39.9413	-1.61	2010315L10Rik	NM_025917.1	Mus musculus RIKEN cDNA 2010315L10 gene (2010315L10Rik), mRNA.
scl30088.4_382-S	-39.8586	-1.4	0610006O14Rik	NM_133764.1	Mus musculus RIKEN cDNA 0610006O14 gene (0610006O14Rik), mRNA.

scl0001320.1_85-S	-39.7139	-1.28	Mdh1	NM_008618.2	Mus musculus malate dehydrogenase 1, NAD (soluble) (Mdh1), mRNA.
scl0002964.1_2-S	-39.6952	-7.54	Sh3kbp1	NM_021389.3	Mus musculus SH3-domain kinase binding protein 1 (Sh3kbp1), mRNA.
scl071941.3_29-S	-39.6042	-1.38	2310051N18Rik	XM_134026.4	Mus musculus RIKEN cDNA 2310051N18 gene (2310051N18Rik), mRNA.
scl0012180.2_196-S	-39.5672	-1.97	Smyd1	NM_009762.1	Mus musculus SET and MYND domain containing 1 (Smyd1), mRNA.
scl19211.5_285-S	-39.5174	-2.9	Fign	NM_021716.1	Mus musculus fidgetin (Fign), mRNA.
scl48193.7_228-S	-39.4861	-1.42	Dscr1	NM_019466.2	Mus musculus Down syndrome critical region homolog 1 (human) (Dscr1), mRNA.
scl0270106.4_75-S	-39.4718	-1.24	Rpl13	NM_016738	Mus musculus ribosomal protein L13 (Rpl13), mRNA.
scl066306.4_122-S	-39.2913	-3.02	2810012G03Rik	NM_175104.3	Mus musculus RIKEN cDNA 2810012G03 gene (2810012G03Rik), mRNA.
scl000287.1_1-S	-39.2033	-1.29	AI043088	NM_198613.1	Mus musculus expressed sequence AI043088 (AI043088), mRNA.
scl058810.1_30-S	-39.2022	-1.26	Akr1a4	NM_021473.2	Mus musculus aldo-keto reductase family 1, member A4 (aldehyde reductase) (Akr1a4), mRNA.
scl0050875.1_33-S	-39.008	-1.45	Tmod3	NM_016963.1	Mus musculus tropomodulin 3 (Tmod3), mRNA.
scl000395.1_15-S	-38.9967	-1.47	Adk	NM_134079	Mus musculus adenosine kinase (Adk), mRNA.
scl0003800.1_141-S	-38.9635	-1.4	Tfam	NM_009360.2	Mus musculus transcription factor A, mitochondrial (Tfam), mRNA.
scl50660.8.1_1-S	-38.9586	-1.43	Mrps10	NM_183086.1	Mus musculus mitochondrial ribosomal protein S10 (Mrps10), mRNA.
scl072195.1_127-S	-38.9031	-2.28	2610524B01Rik	XM_355594	Mus musculus RIKEN cDNA 2610524B01 gene (2610524B01Rik), mRNA.
scl39065.17_337-S	-38.8694	-2.66	Arhgap18	NM_176837.1	Mus musculus Rho GTPase activating protein 18 (Arhgap18), mRNA.
scl31391.7.1_2-S	-38.7886	-2.07	Rcn3	NM_026555.1	Mus musculus reticulocalbin 3, EF-hand calcium binding domain (Rcn3), mRNA.
scl0003727.1_2-S	-38.7861	-3.91	Hnrpk	NM_025279.1	Mus musculus heterogeneous nuclear ribonucleoprotein K (Hnrpk), mRNA.
scl00223696.2_201-S	-38.7623	-1.35	Tomm22	NM_172609.2	Mus musculus translocase of outer mitochondrial membrane 22 homolog (yeast) (Tomm22), mRNA.
scl0116891.1_127-S	-38.6883	-1.48	BC005682	NM_033562.2	Mus musculus cDNA sequence BC005682 (BC005682), mRNA.
scl0002190.1_16-S	-38.6764	-3.11	Fech	NM_007998.3	Mus musculus ferrochelatase (Fech), mRNA.
scl41467.7_13-S	-38.6092	-1.9	Mfap4	NM_029568.1	Mus musculus microfibrillar-associated protein 4 (Mfap4), mRNA.
scl012558.4_4-S	-38.5127	-1.76	Cdh2	NM_007664.1	Mus musculus cadherin 2 (Cdh2), mRNA.
scl00217869.1_91-S	-38.508	-1.51	Eif5	NM_173363.2	Mus musculus eukaryotic translation initiation factor 5 (Eif5), mRNA.
scl0012540.1_20-S	-38.4876	-1.3	Cdc42	NM_009861.1	Mus musculus cell division cycle 42 homolog (S. cerevisiae) (Cdc42), mRNA.
scl00229534.2_23-S	-38.4199	-1.66	Pbxip1	NM_146131.1	Mus musculus pre-B-cell leukemia transcription factor interacting protein 1 (Pbxip1), mRNA.
scl50733.5.1_21-S	-38.3277	-3.86	H2-M3	NM_013819.1	Mus musculus histocompatibility 2, M region locus 3 (H2-M3), mRNA.
scl019182.11_5-S	-38.1145	-1.25	Psmc3	NM_008948.1	Mus musculus proteasome (prosome, macropain) 26S subunit, ATPase 3 (Psmc3), mRNA.
scl018102.1_15-S	-38.0768	-1.51	Nme1	NM_008704.2	Mus musculus expressed in non-metastatic cells 1, protein (Nme1), mRNA.
scl0192170.3_20-S	-38.0053	-1.43	Ddx48	NM_138669.1	Mus musculus DEAD (Asp-Glu-Ala-Asp) box polypeptide 48 (Ddx48), mRNA.
scl50768.5_1-S	-37.8964	-1.66	2310014H01Rik	XM_355011.1	Mus musculus RIKEN cDNA 2310014H01 gene (2310014H01Rik), mRNA.
scl18851.3_427-S	-37.7183	-3.04	6430601A21Rik	NM_175466.2	Mus musculus RIKEN cDNA 6430601A21 gene (6430601A21Rik), mRNA.
scl0018537.2_170-S	-37.6933	-1.36	Pcmt1	NM_008786.1	Mus musculus protein-L-isoaspartate (D-aspartate) O-methyltransferase 1 (Pcmt1), mRNA.
scl000715.1_17-S	-37.6722	-1.99	Got2	NM_010325.1	Mus musculus glutamate oxaloacetate transaminase 2, mitochondrial (Got2), mRNA.
scl000737.1_18-S	-37.5445	-2.43	Atp6v0d1	NM_013477.2	Mus musculus ATPase, H+ transporting, V0 subunit D isoform 1 (Atp6v0d1), mRNA.
scl33551.36_1-S	-37.4246	-2	Phkb	NM_199446.1	Mus musculus phosphorylase kinase beta (Phkb), mRNA.
scl38623.34_689-S	-37.4084	-1.46	A230046K03Rik	XM_193573.3	Mus musculus RIKEN cDNA A230046K03 gene (A230046K03Rik), mRNA.
scl49235.18_0-S	-37.3634	-2.68	Tfrc	NM_011638.3	Mus musculus transferrin receptor (Tfrc), mRNA.
scl0002163.1_25-S	-37.3403	-1.43	Acaa2	NM_177470	Mus musculus acetyl-Coenzyme A acyltransferase 2 (mitochondrial 3-oxoacyl-Coenzyme A thiolase) (Acaa2), mRNA.
scl38595.2_598-S	-37.3355	-1.31	C630002B14Rik	NM_175331.2	Mus musculus RIKEN cDNA C630002B14 gene (C630002B14Rik), mRNA.
scl068460.2_9-S	-37.1957	-1.44	1110001P11Rik	XM_126127.3	Mus musculus RIKEN cDNA 1110001P11 gene (1110001P11Rik), mRNA.
scl23892.3.1_1-S	-37.1732	-1.6	6330579B17Rik	NM_026494.1	Mus musculus RIKEN cDNA 6330579B17 gene (6330579B17Rik), mRNA.
scl37031.1_4-S	-37.1597	-3.02	Bcl9l	NM_030256.1	Mus musculus B-cell CLL/lymphoma 9-like (Bcl9l), mRNA.
scl0002631.1_50-S	-37.1282	-1.41	Eif3s2	NM_018799.1	Mus musculus eukaryotic translation initiation factor 3, subunit 2 (beta) (Eif3s2), mRNA.
scl44070.4.1_3-S	-37.1265	-2.11	Eef1e1	NM_025380.1	Mus musculus eukaryotic translation elongation factor 1 epsilon 1 (Eef1e1), mRNA.
scl066506.1_15-S	-37.0836	-7.43	1810042K04Rik	NM_025604.1	Mus musculus RIKEN cDNA 1810042K04 gene (1810042K04Rik), mRNA.
scl20640.7.7_31-S	-36.7613	-8.47	Pacsin3	NM_028733.1	Mus musculus protein kinase C and casein kinase substrate in neurons 3 (Pacsin3), mRNA.
scl15745.2.1_5-S	-36.6492	-2.1	G0s2	NM_008059.1	Mus musculus G0/G1 switch gene 2 (G0s2), mRNA.
scl066310.2_4-S	-36.6369	-1.3	2810410M20Rik	NM_024428	Mus musculus RIKEN cDNA 2810410M20 gene (2810410M20Rik), mRNA.
scl47844.7.1_12-S	-36.5514	-5.98	Khdrbs3	NM_010158.1	Mus musculus KH domain containing, RNA binding, signal transduction associated 3 (Khdrbs3), mRNA.
scl38665.9.1_23-S	-36.5305	-1.38	Map2k2	NM_023138.3	Mus musculus mitogen activated protein kinase kinase 2 (Map2k2), mRNA.
scl022088.2_10-S	-36.48	-1.44	Tsg101	NM_021884.1	Mus musculus tumor susceptibility gene 101 (Tsg101), mRNA.
scl48922.5.43_8-S	-36.4558	-1.29	Jam2	NM_023844.2	Mus musculus junction adhesion molecule 2 (Jam2), mRNA.
scl19661.4.1_12-S	-36.273	-1.45	Ptpla	NM_013935.1	Mus musculus protein tyrosine phosphatase-like (proline instead of catalytic arginine), member a (Ptpla), mRNA.
scl46892.6_503-S	-36.2556	-1.4	BC025519	XM_110101.3	Mus musculus cDNA sequence BC025519 (BC025519), mRNA.
scl0002846.1_27-S	-36.1337	-1.82	Akr7a5	NM_025337.2	Mus musculus aldo-keto reductase family 7, member A5 (aflatoxin aldehyde reductase) (Akr7a5), mRNA.

scl029876.1_93-S	-36.0654	-1.6	Clic4	XM_124389.1	Mus musculus chloride intracellular channel 4 (mitochondrial) (Clic4), mRNA.
scl33074.8.142_21-S	-36.0639	-1.35	Rps5	NM_009095	Mus musculus ribosomal protein S5 (Rps5), mRNA.
scl022021.6_75-S	-36.0273	-1.48	Tpst1	NM_013837.1	Mus musculus protein-tyrosine sulfotransferase 1 (Tpst1), mRNA.
scl0001807.1_51-S	-35.9929	-2.25	Bfar	NM_025976	Mus musculus bifunctional apoptosis regulator (Bfar), mRNA.
scl0353156.8_65-S	-35.9685	-1.41	Egfl7	NM_198724.1	Mus musculus EGF-like domain 7 (Egfl7), transcript variant b, mRNA.
scl014998.4_34-S	-35.962	-7.57	H2-DMa	NM_010386	Mus musculus histocompatibility 2, class II, locus DMa (H2-DMa), mRNA.
GI_21746160-S	-35.9618	-10.76	2410129E14Rik	NM_023716.1	Mus musculus RIKEN cDNA 2410129E14 gene (2410129E14Rik), mRNA.
scl0001289.1_85-S	-35.958	-1.54	Sgca	NM_009161.1	Mus musculus sarcoglycan, alpha (dystrophin-associated glycoprotein) (Sgca), mRNA.
scl0056428.2_17-S	-35.8873	-1.36	Mtch2	NM_019758.2	Mus musculus mitochondrial carrier homolog 2 (C. elegans) (Mtch2), mRNA.
scl0022350.2_262-S	-35.7052	-1.56	Vil2	NM_009510.1	Mus musculus villin 2 (Vil2), mRNA.
scl39238.2.1_49-S	-35.6695	-1.29	1810049H13Rik	NM_025560.1	Mus musculus RIKEN cDNA 1810049H13 gene (1810049H13Rik), mRNA.
scl027407.6_4-S	-35.6651	-1.69	Abcf2	NM_013853.1	Mus musculus ATP-binding cassette, sub-family F (GCN20), member 2 (Abcf2), mRNA.
scl071784.1_62-S	-35.6449	-2.34	1110007C02Rik	NM_027923.1	Mus musculus RIKEN cDNA 1110007C02 gene (1110007C02Rik), mRNA.
scl35348.11.1_65-S	-35.6445	-6.26	4933406E20Rik	NM_028944.2	Mus musculus RIKEN cDNA 4933406E20 gene (4933406E20Rik), mRNA.
scl25663.5_353-S	-35.6343	-2.09	C130086A10	NM_173746.2	Mus musculus hypothetical protein C130086A10 (C130086A10), mRNA.
scl067941.3_4-S	-35.6162	-1.23	Rps27l	NM_026467.1	Mus musculus ribosomal protein S27-like (Rps27l), mRNA.
scl51379.4_394-S	-35.562	-2.06	2010002N04Rik	NM_134133.1	
scl056541.13_2-S	-35.5031	-1.64	Habp4	NM_019986	Mus musculus hyaluronic acid binding protein 4 (Habp4), mRNA.
scl25278.23_305-S	-35.4718	-1.61	Tek	NM_013690.1	Mus musculus endothelial-specific receptor tyrosine kinase (Tek), mRNA.
scl0022792.2_35-S	-35.4669	-1.6	Zrf2	NM_009584	Mus musculus zuotin related factor 2 (Zrf2), mRNA.
scl067097.5_20-S	-35.4657	-1.24	Rps10	NM_025963	Mus musculus ribosomal protein S10 (Rps10), mRNA.
scl0012859.2_116-S	-35.3753	-1.24	Cox5b	NM_009942.1	Mus musculus cytochrome c oxidase, subunit Vb (Cox5b), mRNA.
scl49065.9.1_50-S	-35.3317	-1.52	Apg3l	NM_026402.1	
scl067278.1_10-S	-35.2217	-1.85	2900092E17Rik	NM_030240.1	Mus musculus RIKEN cDNA 2900092E17 gene (2900092E17Rik), mRNA.
scl49975.4.297_19-S	-35.2181	-2.4	Tubb5	NM_011655.2	Mus musculus tubulin, beta 5 (Tubb5), mRNA.
scl18180.9.1_47-S	-35.1764	-2.32	A030012M09Rik	NM_183028.1	Mus musculus RIKEN cDNA A030012M09 gene (A030012M09Rik), mRNA.
scl45535.5_307-S	-35.1404	-1.48	1810034K20Rik	NM_023397.3	Mus musculus RIKEN cDNA 1810034K20 gene (1810034K20Rik), mRNA.
scl0014719.1_330-S	-35.1281	-1.27	Got2	NM_010325.1	Mus musculus glutamate oxaloacetate transaminase 2, mitochondrial (Got2), mRNA.
scl00320711.2_57-S	-35.0764	-1.52	9830147P19Rik	NM_177238.2	Mus musculus RIKEN cDNA 9830147P19 gene (9830147P19Rik), mRNA.
scl012260.3_14-S	-35.0393	-1.82	C1qb	NM_009777.1	Mus musculus complement component 1, q subcomponent, beta polypeptide (C1qb), mRNA.
scl000628.1_299-S	-35.0391	-1.43	Arl2bp	NM_024191.1	Mus musculus ADP-ribosylation factor-like 2 binding protein (Arl2bp), mRNA.
scl067939.1_2-S	-35.034	-1.41	2010316F05Rik	NM_026465.1	Mus musculus RIKEN cDNA 2010316F05 gene (2010316F05Rik), mRNA.
scl51415.5.1_5-S	-35.0076	-1.89	Ppic	NM_008908.1	Mus musculus peptidylprolyl isomerase C (Ppic), mRNA.
scl0060365.1_251-S	-34.9936	-1.98	Rbm8	NM_025875.1	Mus musculus RNA binding motif protein 8 (Rbm8), mRNA.
scl000210.1_44-S	-34.9798	-7.96	Eif4g2	NM_013507.2	Mus musculus eukaryotic translation initiation factor 4, gamma 2 (Eif4g2), mRNA.
scl35042.9.1_7-S	-34.8792	-5.68	Agpt2	NM_007426.2	Mus musculus angiopoietin 2 (Agpt2), mRNA.
scl019070.8_188-S	-34.8378	-3.71	Prei3	NM_025283.2	Mus musculus preimplantation protein 3 (Prei3), mRNA.
scl27589.4_81-S	-34.8318	-1.57	9130213B05Rik	NM_145562.1	Mus musculus RIKEN cDNA 9130213B05 gene (9130213B05Rik), mRNA.
scl028019.8_226-S	-34.7276	-3.17	Ing4	NM_133345.1	Mus musculus inhibitor of growth family, member 4 (Ing4), mRNA.
scl42591.3.1_5-S	-34.6891	-1.67	Idb2	NM_010496.2	Mus musculus inhibitor of DNA binding 2 (Idb2), mRNA.
scl46497.11.10_2-S	-34.5808	-2.24	2510015F01Rik	XM_354801.1	Mus musculus RIKEN cDNA 2510015F01 gene (2510015F01Rik), mRNA.
scl38368.11_94-S	-34.5449	-1.3	Wif1	NM_011915.1	Mus musculus Wnt inhibitory factor 1 (Wif1), mRNA.
scl016210.11_31-S	-34.5383	-1.5	Impact	NM_008378.1	Mus musculus imprinted and ancient (Impact), mRNA.
scl0227613.1_83-S	-34.5073	-1.51	4930542G03Rik	NM_146116.1	Mus musculus RIKEN cDNA 4930542G03 gene (4930542G03Rik), mRNA.
scl24545.4.1_12-S	-34.5067	-1.41	6720467C03Rik	NM_026558.2	Mus musculus RIKEN cDNA 6720467C03 gene (6720467C03Rik), mRNA.
scl00142682.1_213-S	-34.4787	-1.73	Zcchc14	NM_080855.1	
scl0004026.1_20-S	-34.4176	-1.77	Hadhb	NM_145558	Mus musculus hydroxyacyl-Coenzyme A dehydrogenase/3-ketoacyl-Coenzyme A thiolase/enoyl-Coenzyme A hydratase (trifunctional protein), beta subunit (Hadhb), mRNA.
scl000615.1_57-S	-34.4102	-5.02	Cdh13	NM_019707.1	Mus musculus cadherin 13 (Cdh13), mRNA.
scl54739.8.5_29-S	-34.3762	-1.31	Itgb1bp2	NM_013712.1	Mus musculus integrin beta 1 binding protein 2 (Itgb1bp2), mRNA.
scl29626.4_8-S	-34.3553	-1.27	6720456B07Rik	NM_133937.1	Mus musculus RIKEN cDNA 6720456B07 gene (6720456B07Rik), mRNA.
scl0058249.2_204-S	-34.2762	-1.39	Fibp	NM_021438.1	Mus musculus fibroblast growth factor (acidic) intracellular binding protein (Fibp), mRNA.
scl0068585.2_67-S	-34.1956	-1.25	Rtn4	NM_194054.1	Mus musculus reticulon 4 (Rtn4), transcript variant 5, mRNA.
scl0012837.1_129-S	-34.1277	-1.91	Col8a1	NM_007739.1	Mus musculus procollagen, type VIII, alpha 1 (Col8a1), mRNA.
scl35489.7_147-S	-34.1181	-1.24	Atp1b3	NM_007502.1	Mus musculus ATPase, Na+/K+ transporting, beta 3 polypeptide (Atp1b3), mRNA.
scl00048.1_13-S	-34.1104	-1.42	Sirt3	NM_022433	Mus musculus sirtuin 3 (silent mating type information regulation 2, homolog) 3 (S. cerevisiae) (Sirt3), mRNA.
scl067674.3_12-S	-34.045	-1.81	0610038D11Rik	NM_026306.1	Mus musculus RIKEN cDNA 0610038D11 gene (0610038D11Rik), mRNA.

scl0068493.2_155-S	-33.9207	-1.54	1110007M04Rik	NM_026742.1	Mus musculus RIKEN cDNA 1110007M04 gene (1110007M04Rik), mRNA.
scl0067264.2_318-S	-33.9121	-1.31	Ndufb8	NM_026061.1	Mus musculus NADH dehydrogenase (ubiquinone) 1 beta subcomplex 8 (Ndufb8), mRNA.
scl42555.3_391-S	-33.8307	-2.43	Gpr22	NM_175191.2	Mus musculus G protein-coupled receptor 22 (Gpr22), mRNA.
scl0076438.2_4-S	-33.6407	-1.47	2310015N21Rik	NM_181397.1	Mus musculus RIKEN cDNA 2310015N21 gene (2310015N21Rik), mRNA.
scl32695.9.13_0-S	-33.6235	-1.66	Nosip	NM_025533.1	Mus musculus nitric oxide synthase interacting protein (Nosip), mRNA.
scl000993.1_5-S	-33.6008	-1.48	Eef1b2	NM_018796.2	Mus musculus eukaryotic translation elongation factor 1 beta 2 (Eef1b2), mRNA.
scl00224133.1_32-S	-33.4919	-6.92	Parp14	NM_145481.1	
scl0076142.2_55-S	-33.4783	-1.88	Ppp1r14c	NM_133485.1	Mus musculus protein phosphatase 1, regulatory (inhibitor) subunit 14c (Ppp1r14c), mRNA.
scl015387.16_11-S	-33.4783	-4.69	Hnrpk	NM_025279.1	Mus musculus heterogeneous nuclear ribonucleoprotein K (Hnrpk), mRNA.
scl0002144.1_169-S	-33.4482	-2.3	0610031J06Rik	NM_020003.1	Mus musculus RIKEN cDNA 0610031J06 gene (0610031J06Rik), mRNA.
scl0003000.1_3-S	-33.4446	-1.56	Myl9	XM_283793.2	Mus musculus myosin, light polypeptide 9, regulatory (Myl9), mRNA.
scl0271005.13_174-S	-33.3764	-1.51	Klhdc1	NM_178253.2	Mus musculus kelch domain containing 1 (Klhdc1), mRNA.
scl27818.30.1_7-S	-33.351	-1.36	Anapc4	NM_024213.1	Mus musculus anaphase promoting complex subunit 4 (Anapc4), mRNA.
scl058239.1_71-S	-33.3498	-1.36	Dexi	NM_021428.3	Mus musculus dexamethasone-induced transcript (Dexi), mRNA.
scl31806.5.1_30-S	-33.3194	-1.72	1500019G21Rik	NM_024172.2	Mus musculus RIKEN cDNA 1500019G21 gene (1500019G21Rik), mRNA.
scl059069.6_0-S	-33.2278	-3.2	Tpm3	NM_022314	Mus musculus tropomyosin 3, gamma (Tpm3), mRNA.
scl0066999.2_129-S	-33.2133	-1.24	1500003D12Rik	NM_025895.2	Mus musculus RIKEN cDNA 1500003D12 gene (1500003D12Rik), mRNA.
scl070356.1_235-S	-33.1962	-1.46	St13	NM_133726.1	Mus musculus suppression of tumorigenicity 13 (St13), mRNA.
scl0014980.1_39-S	-33.1572	-1.38	H2-L		
scl31535.10.6_2-S	-33.1473	-1.35	Capns1	NM_009795	Mus musculus calpain, small subunit 1 (Capns1), mRNA.
scl21752.24.8_3-S	-33.1306	-1.24	Atp1a1	NM_144900.1	Mus musculus ATPase, Na+/K+ transporting, alpha 1 polypeptide (Atp1a1), mRNA.
scl39991.5_43-S	-33.0725	-1.71	Spag7	NM_172561	Mus musculus sperm associated antigen 7 (Spag7), mRNA.
scl056282.5_222-S	-33.0351	-1.25	Mrpl12	NM_027204.2	Mus musculus mitochondrial ribosomal protein L12 (Mrpl12), mRNA.
scl030932.2_33-S	-33.0268	-1.75	Zfp330	NM_145600.1	Mus musculus zinc finger protein 330 (Zfp330), mRNA.
scl23686.4_28-S	-32.9906	-1.84	2410166I05Rik	NM_029759.2	Mus musculus RIKEN cDNA 2410166I05 gene (2410166I05Rik), mRNA.
scl0214572.8_7-S	-32.8772	-1.87	BC006705	NM_145404.1	Mus musculus cDNA sequence BC006705 (BC006705), mRNA.
scl0018813.1_104-S	-32.7612	-1.89	Pa2g4	NM_011119.1	Mus musculus proliferation-associated 2G4 (Pa2g4), mRNA.
scl0004139.1_41-S	-32.6702	-1.48	Asrij	NM_023429.2	Mus musculus asrij protein (Asrij), mRNA.
scl37470.5.1_23-S	-32.6692	-1.88	Yeats4	NM_026570.1	
scl0245841.4_4-S	-32.6572	-2.14	Polr2h	NM_145632	Mus musculus polymerase (RNA) II (DNA directed) polypeptide H (Polr2h), mRNA.
scl014109.3_67-S	-32.6553	-1.31	Fau	NM_007990	Mus musculus Finkel-Biskis-Reilly murine sarcoma virus (FBR-MuSV) ubiquitously expressed (fox derived) (Fau), mRNA.
scl020068.3_1-S	-32.5713	-1.26	Rps17	NM_009092.2	Mus musculus ribosomal protein S17 (Rps17), mRNA.
scl020462.9_41-S	-32.4718	-2.77	Sfrs10	NM_009186	Mus musculus splicing factor, arginine/serine-rich 10 (transformer 2 homolog, Drosophila) (Sfrs10), mRNA.
scl0050496.2_2-S	-32.4251	-4.41	E2f6	NM_033270.1	Mus musculus E2F transcription factor 6 (E2f6), mRNA.
scl0003212.1_0-S	-32.3238	-3.57	2410003P15Rik	NM_018888.1	Mus musculus RIKEN cDNA 2410003P15 gene (2410003P15Rik), mRNA.
scl0227292.12_53-S	-32.306	-1.36	Ctdsp1	NM_153088.1	Mus musculus CTD (carboxy-terminal domain, RNA polymerase II, polypeptide A) small phosphatase 1 (Ctdsp1), mRNA.
scl28746.7_64-S	-32.1761	-1.27	2610209M04Rik	NM_025665.1	Mus musculus RIKEN cDNA 2610209M04 gene (2610209M04Rik), mRNA.
scl0003459.1_490-S	-32.0496	-2.31	Lrrfip2	XM_284541.1	Mus musculus leucine rich repeat (in FLII) interacting protein 2 (Lrrfip2), mRNA.
scl32844.9.1_17-S	-32.0055	-1.34	9430029K10Rik	XM_284281.2	Mus musculus RIKEN cDNA 9430029K10 gene (9430029K10Rik), mRNA.
scl068349.3_1-S	-31.9666	-1.23	Ndufs3	XM_130347.1	Mus musculus NADH dehydrogenase (ubiquinone) Fe-S protein 3 (Ndufs3), mRNA.
scl075608.7_106-S	-31.9231	-1.35	2010012F05Rik	NM_029362	Mus musculus RIKEN cDNA 2010012F05 gene (2010012F05Rik), mRNA.
scl26327.26.63_55-S	-31.9076	-2.98	Sec31l1	XM_132230.3	
scl0002293.1_7-S	-31.9065	-3.29	Sfrs5	NM_009159	Mus musculus splicing factor, arginine/serine-rich 5 (SRp40, HRS) (Sfrs5), mRNA.
scl057808.2_82-S	-31.8889	-1.34	Rpl35a	NM_021338.2	Mus musculus ribosomal protein L35a (Rpl35a), mRNA.
scl0002298.1_37-S	-31.884	-2.79	Coq6	NM_172582.1	Mus musculus coenzyme Q6 homolog (yeast) (Coq6), mRNA.
scl0002171.1_4-S	-31.8533	-1.61	Sra1	NM_025291.1	Mus musculus steroid receptor RNA activator 1 (Sra1), mRNA.
scl17782.7.1_158-S	-31.7666	-3.06	1110060O18Rik	NM_026846.1	Mus musculus RIKEN cDNA 1110060O18 gene (1110060O18Rik), mRNA.
scl49028.27_379-S	-31.7302	-3.31	2810413I22Rik	NM_025483.2	Mus musculus RIKEN cDNA 2810413I22 gene (2810413I22Rik), mRNA.
scl18868.1.64_1-S	-31.6926	-5.46	2610318K02Rik	NM_026519.1	Mus musculus RIKEN cDNA 2610318K02 gene (2610318K02Rik), mRNA.
scl0002678.1_34-S	-31.5804	-1.41	Magoh	NM_010760.1	Mus musculus mago-nashi homolog, proliferation-associated (Drosophila) (Magoh), mRNA.
scl39347.7.18_70-S	-31.4705	-5.61	1110028N05Rik	NM_025400.2	Mus musculus RIKEN cDNA 1110028N05 gene (1110028N05Rik), mRNA.
scl00228410.1_260-S	-31.4418	-1.94	Cstf3	NM_145529.1	Mus musculus cleavage stimulation factor, 3 pre-RNA, subunit 3 (Cstf3), mRNA.
scl33261.5_41-S	-31.4183	-1.25	Hsbp1	NM_024219.1	Mus musculus heat shock factor binding protein 1 (Hsbp1), mRNA.
scl0002404.1_3-S	-31.3392	-2.85	Cfl2	NM_007688.1	Mus musculus cofilin 2, muscle (Cfl2), mRNA.
scl000908.1_4-S	-31.3374	-2.92	Hes6	NM_019479.2	Mus musculus hairy and enhancer of split 6 (Drosophila) (Hes6), mRNA.
scl083456.10_6-S	-31.303	-1.37	Mov10l1	NM_031260.1	Mus musculus Moloney leukemia virus 10-like 1 (Mov10l1), mRNA.

scl0003746.1_25-S	-31.2813	-3.79	Gdi3	NM_008112.2	Mus musculus guanosine diphosphate (GDP) dissociation inhibitor 3 (Gdi3), mRNA.
scl44625.4_223-S	-31.2677	-7.64	2900041A09Rik	NM_182839.1	Mus musculus RIKEN cDNA 2900041A09 gene (2900041A09Rik), mRNA.
scl0053607.2_231-S	-31.2633	-2.08	Snrpa	NM_015782.2	Mus musculus small nuclear ribonucleoprotein polypeptide A (Snrpa), mRNA.
scl0012476.2_68-S	-31.2112	-1.23	Cd151	NM_009842.1	Mus musculus CD151 antigen (Cd151), mRNA.
scl0001595.1_53-S	-31.2008	-1.31	9530058B02Rik	NM_026633.1	Mus musculus RIKEN cDNA 9530058B02 gene (9530058B02Rik), mRNA.
scl00209630.2_217-S	-31.1998	-1.39	Frmd4a	NM_172475.2	
scl25931.11.1_56-S	-31.1757	-1.48	Wbscr22	NM_025375.1	Mus musculus Williams Beuren syndrome chromosome region 22 (Wbscr22), mRNA.
scl013806.12_49-S	-31.1469	-1.63	Eno1	NM_023119	Mus musculus enolase 1, alpha non-neuron (Eno1), mRNA.
scl0002924.1_11-S	-31.0389	-3	Psmd10	NM_016883.3	Mus musculus proteasome (prosome, macropain) 26S subunit, non-ATPase, 10 (Psmd10), mRNA.
scl0110557.2_204-S	-31.012	-1.74	H2-Q6	NM_207648	Mus musculus histocompatibility 2, Q region locus 6 (H2-Q6), mRNA.
scl53686.6.11_16-S	-30.9648	-1.27	Prdx4	NM_016764.1	Mus musculus peroxiredoxin 4 (Prdx4), mRNA.
scl47965.12.3_6-S	-30.9623	-1.31	Atp6v1c1	NM_025494	Mus musculus ATPase, H+ transporting, V1 subunit C, isoform 1 (Atp6v1c1), mRNA.
scl0066511.2_242-S	-30.9356	-1.23	2500003M10Rik	NM_023215.3	Mus musculus RIKEN cDNA 2500003M10 gene (2500003M10Rik), mRNA.
scl0003288.1_26-S	-30.9108	-1.87	2410003P15Rik	NM_018888	Mus musculus RIKEN cDNA 2410003P15 gene (2410003P15Rik), mRNA.
scl0001537.1_132-S	-30.8847	-5.42	Rtn4	NM_194054.1	Mus musculus reticulon 4 (Rtn4), transcript variant 5, mRNA.
scl076073.9_113-S	-30.8574	-1.31	0610009F02Rik	NM_029508.1	
scl47039.3.1_28-S	-30.8202	-2.66	Fbxl6	NM_013909.1	Mus musculus F-box and leucine-rich repeat protein 6 (Fbxl6), mRNA.
scl000481.1_29-S	-30.808	-1.51	5330431N19Rik	NM_172639.1	Mus musculus RIKEN cDNA 5330431N19 gene (5330431N19Rik), mRNA.
scl0074006.2_261-S	-30.7741	-2.92	Dnm1l	NM_152816.1	Mus musculus dynamin 1-like (Dnm1l), mRNA.
scl44632.16.1_30-S	-30.7696	-1.26	C130052I12Rik	NM_146047.1	Mus musculus RIKEN cDNA C130052I12 gene (C130052I12Rik), mRNA.
scl53554.5.129_30-S	-30.5857	-1.76	Bad	NM_007522.1	Mus musculus Bcl-associated death promoter (Bad), mRNA.
scl020463.2_41-S	-30.5523	-1.3	Cox7a2l	XM_123188.1	Mus musculus cytochrome c oxidase subunit VIIa polypeptide 2-like (Cox7a2l), mRNA.
scl34412.1_2-S	-30.5377	-6.51	C76566	NM_178879.2	Mus musculus expressed sequence C76566 (C76566), mRNA.
scl070544.3_53-S	-30.5236	-1.31	5730437N04Rik	NM_027457.2	Mus musculus RIKEN cDNA 5730437N04 gene (5730437N04Rik), mRNA.
scl31678.22.1_32-S	-30.4513	-1.72	Sfrs16	NM_016680	Mus musculus splicing factor, arginine/serine-rich 16 (suppressor-of-white-apricot homolog, Drosophila) (Sfrs16), transcript variant L, mRNA.
scl42371.18.1_30-S	-30.4108	-1.71	Pygl	NM_133198.1	Mus musculus liver glycogen phosphorylase (Pygl), mRNA.
scl52252.1.58_3-S	-30.341	-1.45	Snrpd1	NM_009226.2	Mus musculus small nuclear ribonucleoprotein D1 (Snrpd1), mRNA.
scl41883.7.1_5-S	-30.2258	-1.99	2610510L01Rik	NM_026015.1	Mus musculus RIKEN cDNA 2610510L01 gene (2610510L01Rik), mRNA.
scl0019087.2_280-S	-30.2203	-1.42	Prkar2a	NM_008924.1	Mus musculus protein kinase, cAMP dependent regulatory, type II alpha (Prkar2a), mRNA.
scl52826.3.1_11-S	-30.0898	-1.4	Mrpl11	NM_025553.1	Mus musculus mitochondrial ribosomal protein L11 (Mrpl11), mRNA.
scl066212.2_13-S	-30.0486	-2.23	Sec61b	NM_024171	Mus musculus Sec61 beta subunit (Sec61b), mRNA.
scl21970.4.1_6-S	-30.0288	-1.27	Mapbpip	NM_031248.3	Mus musculus mitogen activated protein binding protein interacting protein (Mapbpip), mRNA.
scl42999.15.1_85-S	-29.9403	-1.95	Wdr21	NM_030246.1	Mus musculus WD repeat domain 21 (Wdr21), mRNA.
scl0054473.2_162-S	-29.9387	-1.27	Tollip	NM_023764.2	Mus musculus toll interacting protein (Tollip), mRNA.
scl38698.8_214-S	-29.9047	-1.4	Cnn2	NM_007725.1	Mus musculus calponin 2 (Cnn2), mRNA.
scl018032.3_14-S	-29.8982	-1.24	Nfix	NM_010906.1	Mus musculus nuclear factor I/X (Nfix), mRNA.
scl0105083.1_137-S	-29.8804	-1.66	Pelo	NM_134058.1	Mus musculus pelota homolog (Drosophila) (Pelo), mRNA.
scl000538.1_65-S	-29.8799	-1.44	Banf1	NM_011793.2	Mus musculus barrier to autointegration factor 1 (Banf1), mRNA.
scl068240.2_29-S	-29.8022	-1.56	Rpa3	NM_026632.1	Mus musculus replication protein A3 (Rpa3), mRNA.
scl0021417.2_96-S	-29.7692	-1.34	Zfhx1a	NM_011546.1	Mus musculus zinc finger homeobox 1a (Zfhx1a), mRNA.
scl0110196.3_64-S	-29.7293	-1.35	Fdps	NM_134469.2	Mus musculus farnesyl diphosphate synthetase (Fdps), mRNA.
scl19160.22_222-S	-29.6984	-1.32	Tlk1	NM_172664.2	Mus musculus tousled-like kinase 1 (Tlk1), mRNA.
scl0001446.1_126-S	-29.6373	-1.82	Gosr2	NM_019650.2	Mus musculus golgi SNAP receptor complex member 2 (Gosr2), mRNA.
scl43941.6_46-S	-29.6035	-1.68	Cltb	NM_028870.1	Mus musculus clathrin, light polypeptide (Lcb) (Cltb), mRNA.
scl021770.4_4-S	-29.5972	-1.57	Ppp2r5d	NM_009358.2	Mus musculus protein phosphatase 2, regulatory subunit B (B56), delta isoform (Ppp2r5d), mRNA.
scl27205.18_385-S	-29.5963	-2.76	Aacs	NM_030210.1	Mus musculus acetoacetyl-CoA synthetase (Aacs), mRNA.
scl0001479.1_77-S	-29.5316	-1.27	Ccng1	NM_009831.1	Mus musculus cyclin G1 (Ccng1), mRNA.
scl41258.12_129-S	-29.5294	-1.49	Pitpn	NM_008850.1	Mus musculus phosphatidylinositol transfer protein (Pitpn), mRNA.
scl015526.3_30-S	-29.5087	-1.26	Hspa9a	NM_010481.1	Mus musculus heat shock protein, A (Hspa9a), mRNA.
scl16516.6.1_29-S	-29.4965	-1.45	Pde6d	NM_008801.2	Mus musculus phosphodiesterase 6D, cGMP-specific, rod, delta (Pde6d), mRNA.
scl0002784.1_2-S	-29.4611	-5.57	Dnajc11	NM_172704.1	
scl0066226.1_62-S	-29.431	-1.72	Trappc2	NM_025432	Mus musculus trafficking protein particle complex 2 (Trappc2), mRNA.
scl48818.18.1_43-S	-29.4168	-1.29	Trap1	NM_026508.1	
scl0066878.2_2-S	-29.3229	-1.27	Riok3	NM_024182.2	Mus musculus RIO kinase 3 (yeast) (Riok3), mRNA.
scl000898.1_12-S	-29.3105	-1.55	Ivns1abp	NM_054102.1	Mus musculus influenza virus NS1A binding protein (Ivns1abp), mRNA.
scl00223254.1_50-S	-29.2756	-5.11	Farp1	XM_127877.4	Mus musculus FERM, RhoGEF (Arhgef) and pleckstrin domain protein 1 (chondrocyte-derived) (Farp1), mRNA.

scl050916.6_54-S	-29.2196	-6.51	Irx4	NM_018885.1	Mus musculus Iroquois related homeobox 4 (Drosophila) (Irx4), mRNA.
scl20894.20.1_21-S	-29.1965	-2.41	Pkp4	XM_130277.3	Mus musculus plakophilin 4 (Pkp4), mRNA.
scl011773.12_241-S	-29.1897	-1.3	Ap2m1	NM_009679.1	Mus musculus adaptor protein complex AP-2, mu1 (Ap2m1), mRNA.
scl0001229.1_5-S	-29.1792	-1.47	Camk1	NM_133926.1	Mus musculus calcium/calmodulin-dependent protein kinase I (Camk1), mRNA.
scl066231.1_52-S	-29.1371	-1.26	1500006O09Rik	XM_147163.1	Mus musculus RIKEN cDNA 1500006O09 gene (1500006O09Rik), mRNA.
scl0001763.1_85-S	-29.0305	-2.79	Slc35b1	XM_128634.4	Mus musculus solute carrier family 35, member B1 (Slc35b1), mRNA.
scl0003647.1_892-S	-29.0043	-1.39	Sptlc1	NM_009269.2	Mus musculus serine palmitoyltransferase, long chain base subunit 1 (Sptlc1), mRNA.
scl0002486.1_42-S	-28.9377	-5.53	Myg1	NM_021713.1	Mus musculus melanocyte prolifeating gene 1 (Myg1), mRNA.
scl0067374.1_279-S	-28.881	-1.95	Jam2	NM_023844.2	Mus musculus junction adhesion molecule 2 (Jam2), mRNA.
scl0001563.1_89-S	-28.8396	-2.09	Spnb2	NM_175836.1	Mus musculus spectrin beta 2 (Spnb2), transcript variant 1, mRNA.
scl46277.2.1_30-S	-28.775	-2.13	1110028A07Rik	NM_026808.1	Mus musculus RIKEN cDNA 1110028A07 gene (1110028A07Rik), mRNA.
scl40175.7.1_79-S	-28.7358	-1.24	Obscn	XM_126461.3	
scl0072098.1_29-S	-28.7353	-1.48	2010300G19Rik	NM_028097.2	Mus musculus RIKEN cDNA 2010300G19 gene (2010300G19Rik), mRNA.
scl066366.11_67-S	-28.7338	-1.4	Sdbcag84	NM_025516.2	Mus musculus serologically defined breast cancer antigen 84 (Sdbcag84), mRNA.
scl000125.1_16-S	-28.6864	-6.26	Snrp70	NM_009224.2	Mus musculus U1 small nuclear ribonucleoprotein polypeptide A (Snrp70), mRNA.
scl18899.2.2_47-S	-28.6137	-2.45	1700030A21Rik	NM_026992.1	Mus musculus RIKEN cDNA 1700030A21 gene (1700030A21Rik), mRNA.
scl021922.2_0-S	-28.5928	-2.87	Tna	XM_147338.1	Mus musculus tetranectin (plasminogen binding protein) (Tna), mRNA.
scl38191.11.1_70-S	-28.5737	-2.14	2610020N02Rik	NM_181470.2	Mus musculus RIKEN cDNA 2610020N02 gene (2610020N02Rik), mRNA.
scl34173.5_8-S	-28.5588	-1.54	Egln1	NM_053207.1	Mus musculus EGL nine homolog 1 (C. elegans) (Egln1), mRNA.
scl000928.1_86-S	-28.5088	-2.97	Hspd1	NM_010477.2	Mus musculus heat shock protein 1 (chaperonin) (Hspd1), mRNA.
scl15839.11.1_64-S	-28.5078	-1.28	Ephx1	NM_010145.2	Mus musculus epoxide hydrolase 1, microsomal (Ephx1), mRNA.
scl37735.5.1_7-S	-28.4925	-1.79	Mbd3	NM_013595.1	Mus musculus methyl-CpG binding domain protein 3 (Mbd3), mRNA.
scl0017257.1_80-S	-28.4213	-2.22	Mecp2	NM_010788.1	Mus musculus methyl CpG binding protein 2 (Mecp2), mRNA.
scl068020.5_23-S	-28.36	-1.46	2810002N01Rik	NM_026511.1	Mus musculus RIKEN cDNA 2810002N01 gene (2810002N01Rik), mRNA.
scl056550.9_27-S	-28.2736	-1.25	Ube2d2	NM_019912.1	Mus musculus ubiquitin-conjugating enzyme E2D 2 (Ube2d2), mRNA.
scl32012.1.1_330-S	-28.2276	-5.92	B230325K18Rik	NM_176936.2	Mus musculus RIKEN cDNA B230325K18 gene (B230325K18Rik), mRNA.
scl39939.4_200-S	-28.2074	-2.07	Hic1	NM_010430.1	Mus musculus hypermethylated in cancer 1 (Hic1), mRNA.
scl0066827.2_11-S	-28.1324	-1.27	Ttc1	NM_133795.1	Mus musculus tetratricopeptide repeat domain 1 (Ttc1), mRNA.
gi_32129296_ref_NM_00	-28.1251	-1.39	Rpl13a	NM_009438.3	Mus musculus ribosomal protein L13a (Rpl13a), mRNA.
scl0012514.2_91-S	-28.1143	-4.46	Cd68	NM_009853.1	Mus musculus CD68 antigen (Cd68), mRNA.
scl021859.7_4-S	-28.0962	-2.26	Timp3	NM_011595.1	Mus musculus tissue inhibitor of metalloproteinase 3 (Timp3), mRNA.
scl20390.11.1_18-S	-28.0251	-1.75	Ccndbp1	NM_010761.1	Mus musculus cyclin D-type binding-protein 1 (Ccndbp1), mRNA.
scl21129.4_153-S	-28.0067	-2	Mrps2	NM_080452.2	Mus musculus mitochondrial ribosomal protein S2 (Mrps2), mRNA.
scl0011564.2_163-S	-27.9387	-1.29	Adsl	NM_009634.3	Mus musculus adenylosuccinate lyase (Adsl), mRNA.
scl000238.1_112-S	-27.9226	-3.95	Stard10	NM_019990.1	Mus musculus START domain containing 10 (Stard10), mRNA.
scl52418.9.1_3-S	-27.9146	-1.93	Kcnip2	NM_145703.1	Mus musculus Kv channel-interacting protein 2 (Kcnip2), mRNA.
scl0003604.1_30-S	-27.847	-1.27	Etfa	NM_145615.2	Mus musculus electron transferring flavoprotein, alpha polypeptide (Etfa), nuclear gene encoding mitochondrial protein, mRNA.
scl23689.13.5_3-S	-27.8164	-1.44	Extl1	NM_019578	Mus musculus exostoses (multiple)-like 1 (Extl1), mRNA.
scl48378.7.7_6-S	-27.8138	-1.64	Nit2	NM_023175.1	
scl000719.1_1-S	-27.7769	-2	5033428A16Rik	XM_356102.1	
scl35682.17.1_6-S	-27.7188	-1.33	Snx1	NM_019727.1	Mus musculus sorting nexin 1 (Snx1), mRNA.
scl16334.7.1_2-S	-27.5796	-3.37	Dars	NM_145507.1	Mus musculus aspartyl-tRNA synthetase (Dars), mRNA.
scl0001304.1_8-S	-27.5312	-3.22	4930579A11Rik	NM_029478.2	Mus musculus RIKEN cDNA 4930579A11 gene (4930579A11Rik), mRNA.
scl0016905.1_7-S	-27.5118	-1.32	Lmna	NM_019390.1	Mus musculus lamin A (Lmna), mRNA.
scl45193.8_18-S	-27.4653	-1.34	Ednrb	NM_007904.2	Mus musculus endothelin receptor type B (Ednrb), mRNA.
scl000989.1_15-S	-27.4585	-4.13	sty	U21209	
scl50794.7.1_43-S	-27.397	-1.34	Clic1	NM_033444.1	Mus musculus chloride intracellular channel 1 (Clic1), mRNA.
scl33440.8_211-S	-27.3793	-1.93	Cklfsf3	NM_024217.2	Mus musculus chemokine-like factor super family 3 (Cklfsf3), mRNA.
scl47677.6.2_17-S	-27.3465	-1.31	Bzrp	NM_009775.2	Mus musculus benzodiazepine receptor, peripheral (Bzrp), mRNA.
scl17433.3.1_30-S	-27.3259	-1.54	Phlda3	NM_013750.1	Mus musculus pleckstrin homology-like domain, family A, member 3 (Phlda3), mRNA.
scl15945.4.1_0-S	-27.2995	-1.25	1110021H02Rik	NM_025388.1	Mus musculus RIKEN cDNA 1110021H02 gene (1110021H02Rik), mRNA.
scl0001825.1_141-S	-27.2911	-4	A2bp1	NM_183188	Mus musculus ataxin 2 binding protein 1 (A2bp1), transcript variant 2, mRNA.
scl013629.6_24-S	-27.2716	-2.42	Eef2	NM_007907.1	Mus musculus eukaryotic translation elongation factor 2 (Eef2), mRNA.
scl066495.2_23-S	-27.2696	-1.2	Ndufb3	NM_025597.1	Mus musculus NADH dehydrogenase (ubiquinone) 1 beta subcomplex 3 (Ndufb3), mRNA.
scl33620.26.1_275-S	-27.2513	-2.76	Inpp4b	XM_134427.3	
scl22797.19_196-S	-27.246	-1.2	D3Jfr1	NM_144901.2	Mus musculus DNA segment, Chr 3, MJeffers 1 (D3Jfr1), mRNA.

scl44709.8_227-S	-27.2416	-1.31	Smad5	NM_008541.2	
scl00319263.2_23-S	-27.2208	-1.27	A030012M09Rik	NM_183028.1	Mus musculus RIKEN cDNA A030012M09 gene (A030012M09Rik), mRNA.
scl0020677.2_120-S	-27.2156	-2.01	Sox4	NM_009238.1	Mus musculus SRY-box containing gene 4 (Sox4), mRNA.
scl0003185.1_157-S	-26.9813	-5.04	Crat	NM_007760	Mus musculus carnitine acetyltransferase (Crat), mRNA.
scl31469.3.28_7-S	-26.9579	-1.31	D7Rp2e	NM_033080.1	Mus musculus DNA segment, Chr 7, Roswell Park 2 complex, expressed (D7Rp2e), mRNA.
scl23113.7.1_10-S	-26.9257	-1.54	Mlf1	NM_010801.1	Mus musculus myeloid leukemia factor 1 (Mlf1), mRNA.
scl0326618.9_77-S	-26.9087	-1.5	Tpm4	XM_134274.3	
scl099683.1_9-S	-26.8777	-1.48	Sec24b	XM_131192.5	
scl51823.8.1_71-S	-26.8414	-1.4	Tnfsf5ip1	NM_134138.1	Mus musculus tumor necrosis factor superfamily, member 5-induced protein 1 (Tnfsf5ip1), mRNA.
scl0105245.1_201-S	-26.8181	-1.43	Txndc5	NM_145367.2	Mus musculus thioredoxin domain containing 5 (Txndc5), mRNA.
scl013063.3_11-S	-26.7983	-1.19	Cycs	NM_007808.2	Mus musculus cytochrome c, somatic (Cycs), mRNA.
scl052469.1_143-S	-26.7931	-1.24	D11Ertd99e	NM_026618.1	Mus musculus DNA segment, Chr 11, ERATO Doi 99, expressed (D11Ertd99e), mRNA.
scl0071448.2_44-S	-26.7628	-2.69	5530601I19Rik	NM_027797.1	Mus musculus RIKEN cDNA 5530601I19 gene (5530601I19Rik), mRNA.
scl068035.2_9-S	-26.7301	-1.8	3100004P22Rik	NM_133693.1	Mus musculus RIKEN cDNA 3100004P22 gene (3100004P22Rik), mRNA.
scl000768.1_18-S	-26.7131	-1.61	5230400G24Rik	NM_029409.2	Mus musculus RIKEN cDNA 5230400G24 gene (5230400G24Rik), mRNA.
scl17725.6_585-S	-26.6535	-1.3	Itm2c	NM_022417.1	Mus musculus integral membrane protein 2C (Itm2c), mRNA.
scl0065970.2_260-S	-26.653	-1.43	D15Ertd366e	NM_023063.1	Mus musculus DNA segment, Chr 15, ERATO Doi 366, expressed (D15Ertd366e), mRNA.
scl35076.15.1_19-S	-26.6338	-1.3	Gas6	NM_019521.1	Mus musculus growth arrest specific 6 (Gas6), mRNA.
scl0016179.2_78-S	-26.5191	-1.59	Irak1	NM_008363.1	Mus musculus interleukin-1 receptor-associated kinase 1 (Irak1), mRNA.
scl31908.3.1_72-S	-26.4554	-1.61	Ryd5	XM_355976.1	
scl0072981.1_191-S	-26.4449	-1.42	Prkrir	NM_028410.1	Mus musculus protein-kinase, interferon-inducible double stranded RNA dependent inhibitor, repressor of (P58 repressor) (Prkrir), mRNA.
scl0011307.2_72-S	-26.4299	-2.27	Abcg1	NM_009593.1	Mus musculus ATP-binding cassette, sub-family G (WHITE), member 1 (Abcg1), mRNA.
scl0013822.2_330-S	-26.4229	-2.23	Epb4.1l2	NM_013511.1	Mus musculus erythrocyte protein band 4.1-like 2 (Epb4.1l2), mRNA.
scl020382.2_66-S	-26.4088	-1.26	Sfrs2	NM_011358.1	Mus musculus splicing factor, arginine/serine-rich 2 (SC-35) (Sfrs2), mRNA.
scl000794.1_5-S	-26.4079	-1.32	Tsn	NM_011650.2	Mus musculus translin (Tsn), mRNA.
scl35096.10_154-S	-26.4014	-1.45	Ankrd10	NM_133971.1	Mus musculus ankyrin repeat domain 10 (Ankrd10), mRNA.
scl0067433.1_155-S	-26.392	-1.36	0610011N22Rik	NM_024201.2	Mus musculus RIKEN cDNA 0610011N22 gene (0610011N22Rik), mRNA.
scl48775.5_265-S	-26.264	-1.31	Emp2	NM_007929.1	Mus musculus epithelial membrane protein 2 (Emp2), mRNA.
scl057321.4_17-S	-26.2153	-1.51	Terf2ip	NM_020584.1	Mus musculus telomeric repeat binding factor 2, interacting protein (Terf2ip), mRNA.
scl53255.10.1_6-S	-26.2121	-1.29	D330024H06Rik	NM_181404.3	Mus musculus RIKEN cDNA D330024H06 gene (D330024H06Rik), mRNA.
scl42057.12_19-S	-26.1605	-1.33	Wars	NM_011710.2	Mus musculus tryptophanyl-tRNA synthetase (Wars), mRNA.
scl0077683.1_104-S	-26.1211	-3.21	Ehmt1	NM_172545.1	
scl0003607.1_28-S	-26.1175	-1.38	Il1rl1l	NM_010744.1	Mus musculus interleukin 1 receptor-like 1 ligand (Il1rl1l), mRNA.
scl012443.1_0-S	-26.1066	-2.96	Ccnd1	NM_007631.1	Mus musculus cyclin D1 (Ccnd1), mRNA.
scl49852.7_79-S	-26.0186	-2.39	1600025D17Rik	NM_028065	
scl49273.6_155-S	-25.9767	-2.38	Hrasls	NM_013751.3	Mus musculus HRAS-like suppressor (Hrasls), mRNA.
scl49970.18.121_17-S	-25.9473	-1.61	Abcf1	NM_013854.1	Mus musculus ATP-binding cassette, sub-family F (GCN20), member 1 (Abcf1), mRNA.
scl39557.17.1_8-S	-25.9014	-1.28	Dnajc7	NM_019795.3	Mus musculus DnaJ (Hsp40) homolog, subfamily C, member 7 (Dnajc7), mRNA.
scl0002267.1_458-S	-25.8235	-5.12	Osbpl1a	NM_020573.1	Mus musculus oxysterol binding protein-like 1A (Osbpl1a), mRNA.
scl0012226.2_9-S	-25.8196	-2.21	Btg1	NM_007569.1	Mus musculus B-cell translocation gene 1, anti-proliferative (Btg1), mRNA.
scl068794.13_69-S	-25.7897	-1.21	1110055E19Rik	XM_284175.2	Mus musculus RIKEN cDNA 1110055E19 gene (1110055E19Rik), mRNA.
scl0001102.1_1-S	-25.7771	-1.31	Suclg1	NM_019879.1	Mus musculus succinate-CoA ligase, GDP-forming, alpha subunit (Suclg1), mRNA.
scl0003102.1_1-S	-25.7678	-1.37	Map1lc3a	NM_025735.1	Mus musculus microtubule-associated protein 1 light chain 3 alpha (Map1lc3a), mRNA.
scl21185.13.1_16-S	-25.7362	-1.29	Anapc2	NM_175300.2	Mus musculus anaphase promoting complex subunit 2 (Anapc2), mRNA.
scl000184.1_1-S	-25.7137	-6.01	Sec23ip	XM_133737.4	Mus musculus Sec23 interacting protein (Sec23ip), mRNA.
scl0023828.2_1-S	-25.7008	-1.25	Bves	NM_024285.1	Mus musculus blood vessel epicardial substance (Bves), mRNA.
scl0013685.1_134-S	-25.6871	-3.49	Eif4ebp1	NM_007918.2	Mus musculus eukaryotic translation initiation factor 4E binding protein 1 (Eif4ebp1), mRNA.
scl24435.7.6_19-S	-25.6625	-1.68	Smu1	NM_021535.2	
scl23202.2_5-S	-25.6601	-4.29	2810489O06Rik	NM_175386.3	Mus musculus RIKEN cDNA 2810489O06 gene (2810489O06Rik), mRNA.
scl39392.13.1_3-S	-25.6398	-1.22	D11Ertd498e	NM_145940.1	Mus musculus DNA segment, Chr 11, ERATO Doi 498, expressed (D11Ertd498e), mRNA.
scl0003895.1_38-S	-25.5927	-1.27	Myl6	NM_010860	Mus musculus myosin, light polypeptide 6, alkali, smooth muscle and non-muscle (Myl6), mRNA.
scl54430.4.1_35-S	-25.5916	-1.39	Ebp	NM_007898.2	Mus musculus phenylalkylamine Ca2+ antagonist (emopamil) binding protein (Ebp), mRNA.
scl0001036.1_14-S	-25.5835	-4.92	0610006O14Rik	NM_133764.1	Mus musculus RIKEN cDNA 0610006O14 gene (0610006O14Rik), mRNA.
scl014773.13_0-S	-25.5786	-1.8	Gprk5	NM_018869.2	Mus musculus G protein-coupled receptor kinase 5 (Gprk5), mRNA.
scl0002729.1_104-S	-25.5671	-2.49	Rgs3	NM_134257.1	Mus musculus regulator of G-protein signaling 3 (Rgs3), mRNA.
scl42676.6_93-S	-25.4971	-1.41	Rab10	NM_016676.2	Mus musculus RAB10, member RAS oncogene family (Rab10), mRNA.

scl42274.8.1_35-S	-25.4905	-1.51	Med6	NM_027213.2	Mus musculus mediator of RNA polymerase II transcription, subunit 6 homolog (yeast) (Med6), mRNA.
scl013831.1_46-S	-25.4236	-2.19	Epc1	NM_027497.1	Mus musculus enhancer of polycomb homolog 1 (Drosophila) (Epc1), transcript variant 2, mRNA.
scl50105.6_4-S	-25.4167	-1.62	Ppil1	NM_026845.1	Mus musculus peptidylprolyl isomerase (cyclophilin)-like 1 (Ppil1), mRNA.
scl32052.6.1_83-S	-25.3986	-2.35	Ypel3	NM_025347.1	
scl19472.10_43-S	-25.3919	-1.28	Sh3glb2	NM_139302.1	Mus musculus SH3-domain GRB2-like endophilin B2 (Sh3glb2), mRNA.
scl22402.6.1_12-S	-25.3298	-1.26	Mrps28	NM_025434.2	Mus musculus mitochondrial ribosomal protein S28 (Mrps28), mRNA.
scl0004044.1_9-S	-25.3113	-1.6	Ppp1cb	NM_172707.1	Mus musculus protein phosphatase 1, catalytic subunit, beta isoform (Ppp1cb), mRNA.
scl30562.12.1_13-S	-25.2937	-1.32	Uros	NM_009479.1	Mus musculus uroporphyrinogen III synthase (Uros), mRNA.
scl00242291.2_165-S	-25.2834	-2.26	1110001C20Rik	NM_177730.2	Mus musculus RIKEN cDNA 1110001C20 gene (1110001C20Rik), mRNA.
scl0097159.2_118-S	-25.2594	-1.45	A430005L14Rik	NM_175287.2	Mus musculus RIKEN cDNA A430005L14 gene (A430005L14Rik), mRNA.
scl45489.6.1_24-S	-25.1733	-1.75	2510005D08Rik	NM_026526.1	Mus musculus RIKEN cDNA 2510005D08 gene (2510005D08Rik), mRNA.
scl40109.5.1_10-S	-25.1699	-1.56	2410012H22Rik	XM_126343.1	Mus musculus RIKEN cDNA 2410012H22 gene (2410012H22Rik), mRNA.
scl35716.24.1_20-S	-25.139	-1.71	Map2k5	NM_011840.1	Mus musculus mitogen activated protein kinase kinase 5 (Map2k5), mRNA.
scl0403187.2_39-S	-25.1217	-1.3	D630048P19Rik	NM_207525.1	Mus musculus RIKEN cDNA D630048P19 gene (D630048P19Rik), mRNA.
scl00243382.2_254-S	-25.1182	-1.47	A930026L03Rik	NM_175523.2	Mus musculus RIKEN cDNA A930026L03 gene (A930026L03Rik), mRNA.
scl25783.3.1_76-S	-25.0472	-1.53	Zfp99	NM_023322.1	Mus musculus zinc finger protein 99 (Zfp99), mRNA.
scl0076373.1_321-S	-25.0252	-4.99	2810409K11Rik	XM_133164	Mus musculus RIKEN cDNA 2810409K11 gene (2810409K11Rik), mRNA.
scl54833.10_21-S	-24.9864	-1.19	Atp6ap1	NM_018794.2	Mus musculus ATPase, H+ transporting, lysosomal accessory protein 1 (Atp6ap1), mRNA.
scl0381974.1_329-S	-24.966	-5.07	Mrgprg	NM_203492.1	Mus musculus MAS-related GPR, member G (Mrgprg), mRNA.
scl000251.1_1-S	-24.9576	-1.46	Mrpl48	NM_026971.2	Mus musculus mitochondrial ribosomal protein L48 (Mrpl48), transcript variant 2, mRNA.
scl068552.1_11-S	-24.9285	-1.2	1110003E01Rik	NM_133697.1	Mus musculus RIKEN cDNA 1110003E01 gene (1110003E01Rik), mRNA.
scl016905.3_15-S	-24.9253	-1.26	Lmna	NM_019390.1	Mus musculus lamin A (Lmna), mRNA.
scl0001498.1_3411-S	-24.8972	-1.61	Ankfy1	NM_009671	Mus musculus ankyrin repeat and FYVE domain containing 1 (Ankfy1), mRNA.
scl19139.14_223-S	-24.8323	-1.96	Waspip	NM_153138.2	Mus musculus Wiskott-Aldrich syndrome protein interacting protein (Waspip), mRNA.
scl022121.1_118-S	-24.7791	-1.33	Rpl13a	NM_009438	Mus musculus ribosomal protein L13a (Rpl13a), mRNA.
scl073737.1_220-S	-24.7503	-1.28	1110008P14Rik	NM_198001.1	Mus musculus RIKEN cDNA 1110008P14 gene (1110008P14Rik), mRNA.
scl0003562.1_115-S	-24.6859	-1.37	Mrpl3	NM_053159.2	Mus musculus mitochondrial ribosomal protein L3 (Mrpl3), mRNA.
scl30601.15_186-S	-24.6753	-1.26	Ate1	NM_013799.2	Mus musculus arginine-tRNA-protein transferase 1 (Ate1), mRNA.
scl29767.12_42-S	-24.6445	-1.23	Mgll	NM_011844.3	Mus musculus monoglyceride lipase (Mgll), mRNA.
scl31481.11_594-S	-24.6354	-1.32	2700023B17Rik	NM_025948.1	Mus musculus RIKEN cDNA 2700023B17 gene (2700023B17Rik), mRNA.
scl43949.6.1_8-S	-24.5996	-1.39	Thoc3	NM_028597.1	Mus musculus THO complex 3 (Thoc3), mRNA.
scl54811.82_56-S	-24.5712	-1.27	Dmd	NM_007868.1	Mus musculus dystrophin, muscular dystrophy (Dmd), mRNA.
scl0018117.1_185-S	-24.564	-1.74	Noc4	NM_010926.1	Mus musculus neighbor of Cox4 (Noc4), mRNA.
scl00264064.2_74-S	-24.5163	-5.56	Cdk8	NM_181570.1	Mus musculus cyclin-dependent kinase 8 (Cdk8), transcript variant 1, mRNA.
scl066359.2_17-S	-24.4785	-1.21	2310005N03Rik	NM_025511.1	Mus musculus RIKEN cDNA 2310005N03 gene (2310005N03Rik), mRNA.
scl0066190.1_159-S	-24.4662	-2.03	Phca	NM_025408.1	Mus musculus phytoceramidase, alkaline (Phca), mRNA.
scl026429.1_36-S	-24.4416	-3.64	Orc5l	NM_011959.1	Mus musculus origin recognition complex, subunit 5-like (S. cerevisiae) (Orc5l), mRNA.
scl0268490.8_24-S	-24.404	-1.64	2600001B17Rik	NM_172947.1	
scl28384.6_282-S	-24.3658	-1.83	Ccnd2	NM_009829.2	Mus musculus cyclin D2 (Ccnd2), mRNA.
scl26325.14_6-S	-24.3515	-6	AI461788	NM_172714.1	Mus musculus expressed sequence AI461788 (AI461788), mRNA.
scl011545.25_15-S	-24.3408	-1.46	Adprt1	NM_007415.2	Mus musculus ADP-ribosyltransferase (NAD+; poly (ADP-ribose) polymerase) 1 (Adprt1), mRNA.
scl0108121.3_5-S	-24.3394	-1.54	U2af1	NM_024187	Mus musculus U2 small nuclear ribonucleoprotein auxiliary factor (U2AF) 1 (U2af1), mRNA.
scl31901.9_22-S	-24.3159	-1.25	Psmd13	NM_011875.2	Mus musculus proteasome (prosome, macropain) 26S subunit, non-ATPase, 13 (Psmd13), mRNA.
scl0003277.1_55-S	-24.2798	-2.22	H13	NM_010376.2	Mus musculus histocompatibility 13 (H13), mRNA.
scl0012050.1_82-S	-24.2672	-1.22	Bcl2l2	NM_007537.1	Mus musculus Bcl2-like 2 (Bcl2l2), mRNA.
scl45926.20.1_29-S	-24.2088	-1.37	Pcca	NM_144844.1	Mus musculus propionyl-Coenzyme A carboxylase, alpha polypeptide (Pcca), mRNA.
scl53699.1.245_256-S	-24.1738	-4.3	Kctd12b	NM_175429.3	Mus musculus potassium channel tetramerisation domain containing 12b (Kctd12b), mRNA.
scl0066377.1_24-S	-24.1692	-1.21	Ndufc1	NM_025523.1	Mus musculus NADH dehydrogenase (ubiquinone) 1, subcomplex unknown, 1 (Ndufc1), mRNA.
scl056436.9_4-S	-24.1685	-1.33	Adrm1	NM_019822.2	Mus musculus adhesion regulating molecule 1 (Adrm1), mRNA.
scl0067326.2_320-S	-24.1634	-1.51	1700037H04Rik	NM_026091.1	Mus musculus RIKEN cDNA 1700037H04 gene (1700037H04Rik), mRNA.
scl18457.19.17_30-S	-24.1495	-1.7	Trpc4ap	NM_019828.1	Mus musculus transient receptor potential cation channel, subfamily C, member 4 associated protein (Trpc4ap), mRNA.
scl0003913.1_65-S	-24.0648	-1.75	Cdk4	NM_009870.2	Mus musculus cyclin-dependent kinase 4 (Cdk4), mRNA.
scl24262.3_152-S	-24.0144	-2.9	Hdhd3	NM_024257.1	
scl0019157.1_43-S	-23.9856	-1.48	Pscd1	NM_011180.2	Mus musculus pleckstrin homology, Sec7 and coiled-coil domains 1 (Pscd1), mRNA.
scl0003075.1_15-S	-23.9723	-2.33	2310042M24Rik	NM_025868.1	Mus musculus RIKEN cDNA 2310042M24 gene (2310042M24Rik), mRNA.
scl25753.9_50-S	-23.9577	-1.19	Ubl3	NM_011908.1	Mus musculus ubiquitin-like 3 (Ubl3), mRNA.

scl37899.10_160-S	-23.9569	-1.24	Vps26	NM_133672	Mus musculus vacuolar protein sorting 26 (yeast) (Vps26), mRNA.
scl066154.6_169-S	-23.9425	-1.25	Tmem14c	NM_025387.1	
scl31799.8.1_22-S	-23.9182	-5.82	Ube2s	NM_133777.1	Mus musculus ubiquitin-conjugating enzyme E2S (Ube2s), mRNA.
scl42728.4.1_1-S	-23.9163	-1.25	Siva	NM_013929	Mus musculus Cd27 binding protein (Hindu God of destruction) (Siva), mRNA.
scl072330.2_29-S	-23.9032	-2.06	Kbtbd5	XM_147132.2	
scl32920.6.1_10-S	-23.8823	-6.65	Tgfb1	NM_011577.1	Mus musculus transforming growth factor, beta 1 (Tgfb1), mRNA.
scl098710.3_111-S	-23.8741	-2.13	Rabif	NM_145510.1	
scl40402.14.1_6-S	-23.8455	-1.3	4933407N01Rik	NM_025745.2	Mus musculus RIKEN cDNA 4933407N01 gene (4933407N01Rik), mRNA.
scl39222.8.1_55-S	-23.8214	-1.41	Cbr2	NM_007621.1	Mus musculus carbonyl reductase 2 (Cbr2), mRNA.
scl069029.1_30-S	-23.8163	-1.18	1500032L24Rik	XM_193800.2	Mus musculus RIKEN cDNA 1500032L24 gene (1500032L24Rik), mRNA.
scl19835.1_73-S	-23.7634	-4.39	Rnpc1	NM_019547.1	Mus musculus RNA-binding region (RNP1, RRM) containing 1 (Rnpc1), mRNA.
scl0004155.1_21-S	-23.731	-1.75	Hip2	NM_016786.2	Mus musculus huntingtin interacting protein 2 (Hip2), mRNA.
scl019989.6_6-S	-23.7178	-1.19	Rpl7	NM_011291	Mus musculus ribosomal protein L7 (Rpl7), mRNA.
scl0066801.1_114-S	-23.7175	-2.91	Prkrip1	NM_025774.1	Mus musculus Prkr interacting protein 1 (IL11 inducible) (Prkrip1), mRNA.
scl33204.17_108-S	-23.6845	-1.23	D8Ertd325e	NM_025804	Mus musculus DNA segment, Chr 8, ERATO Doi 325, expressed (D8Ertd325e), mRNA.
scl29544.11_278-S	-23.6703	-1.89	Mfap5	NM_015776	Mus musculus microfibrillar associated protein 5 (Mfap5), mRNA.
scl21737.4_335-S	-23.662	-1.37	Olfml3	NM_133859.1	
scl0056376.2_179-S	-23.656	-2.8	1110001A05Rik	NM_019808.1	Mus musculus RIKEN cDNA 1110001A05 gene (1110001A05Rik), mRNA.
scl0067236.2_173-S	-23.6461	-1.34	2810452K22Rik	NM_026048.1	Mus musculus RIKEN cDNA 2810452K22 gene (2810452K22Rik), mRNA.
scl0114863.5_23-S	-23.5978	-1.95	Prosc	NM_054057.1	Mus musculus proline synthetase co-transcribed (Prosc), mRNA.
scl0001492.1_866-S	-23.5567	-5.46	Myocd	NM_146386.1	Mus musculus myocardin (Myocd), mRNA.
scl0018008.1_3-S	-23.5468	-4.8	Nes	NM_016701.2	Mus musculus nestin (Nes), mRNA.
scl39475.6_48-S	-23.5224	-1.84	Gosr2	NM_019650.2	Mus musculus golgi SNAP receptor complex member 2 (Gosr2), mRNA.
scl36834.4.1_4-S	-23.514	-3.16	Snapc5	NM_183316.1	Mus musculus small nuclear RNA activating complex, polypeptide 5 (Snapc5), mRNA.
scl16965.6_134-S	-23.4874	-1.39	Tceb1	NM_026456.2	Mus musculus transcription elongation factor B (SIII), polypeptide 1 (Tceb1), mRNA.
scl48855.3.1_17-S	-23.4735	-1.33	Cbr1	NM_007620.1	Mus musculus carbonyl reductase 1 (Cbr1), mRNA.
scl27602.3.1_69-S	-23.4581	-1.42	Cxcl4	NM_019932.1	Mus musculus chemokine (C-X-C motif) ligand 4 (Cxcl4), mRNA.
scl25380.5_310-S	-23.4527	-1.51	Slc31a1	NM_175090.2	Mus musculus solute carrier family 31, member 1 (Slc31a1), mRNA.
scl37876.12_7-S	-23.4017	-2.03	Sirt1	NM_019812.1	Mus musculus sirtuin 1 ((silent mating type information regulation 2, homolog) 1 (S. cerevisiae) (Sirt1), mRNA.
scl000669.1_31-S	-23.2945	-1.3	Gpsn2	NM_134118.1	Mus musculus glycoprotein, synaptic 2 (Gpsn2), mRNA.
scl0001548.1_1-S	-23.2756	-4.48	Abca9	NM_147220.1	Mus musculus ATP-binding cassette transporter sub-family A member 9 (Abca9), mRNA.
scl013808.8_24-S	-23.2426	-1.24	Eno3	NM_007933.2	Mus musculus enolase 3, beta muscle (Eno3), mRNA.
scl21370.9_11-S	-23.1916	-1.19	Acadm	NM_007382	Mus musculus acetyl-Coenzyme A dehydrogenase, medium chain (Acadm), mRNA.
scl18631.16_116-S	-23.1717	-1.62	Slc23a2	NM_018824.2	Mus musculus solute carrier family 23 (nucleobase transporters), member 2 (Slc23a2), mRNA.
scl0002598.1_1-S	-23.1148	-4.77	Prnpip1	NM_080469.2	Mus musculus prion protein interacting protein 1 (Prnpip1), mRNA.
scl0002929.1_32-S	-23.104	-1.71	2610029G23Rik	NM_026312.2	Mus musculus RIKEN cDNA 2610029G23 gene (2610029G23Rik), mRNA.
scl068955.1_4-S	-23.0997	-3.1	1500001A10Rik	NM_026886	Mus musculus RIKEN cDNA 1500001A10 gene (1500001A10Rik), mRNA.
scl21068.9_287-S	-23.0867	-2.27	2810003C17Rik	NM_145144.1	Mus musculus RIKEN cDNA 2810003C17 gene (2810003C17Rik), mRNA.
scl0076846.1_142-S	-23.0804	-1.21	3010033P07Rik	NM_029767.1	
scl0020638.1_79-S	-23.0711	-1.88	Snrpb	NM_009225.1	Mus musculus small nuclear ribonucleoprotein B (Snrpb), mRNA.
scl40464.11_0-S	-23.0534	-1.21	Ugp2	NM_139297.2	Mus musculus UDP-glucose pyrophosphorylase 2 (Ugp2), mRNA.
scl0001990.1_3-S	-23.0459	-1.92	4933434E20Rik	NM_025762	Mus musculus RIKEN cDNA 4933434E20 gene (4933434E20Rik), mRNA.
scl020541.2_143-S	-23.0298	-1.33	Slc8a1	NM_011406.1	Mus musculus solute carrier family 8 (sodium/calcium exchanger), member 1 (Slc8a1), mRNA.
scl068089.7_12-S	-23.0276	-1.43	Arpc4	NM_026552	Mus musculus actin related protein 2/3 complex, subunit 4 (Arpc4), mRNA.
scl35515.3_6-S	-22.9388	-1.95	1200002G13Rik	NM_025360.1	Mus musculus RIKEN cDNA 1200002G13 gene (1200002G13Rik), mRNA.
scl000968.1_12-S	-22.9228	-1.75	Uchl5	NM_019562.1	Mus musculus ubiquitin carboxyl-terminal esterase L5 (Uchl5), mRNA.
scl0001010.1_64-S	-22.8802	-3.53	Ppil3	NM_027351	Mus musculus peptidylprolyl isomerase (cyclophilin)-like 3 (Ppil3), mRNA.
scl41505.7.1_14-S	-22.865	-5.42	2310033P09Rik	NM_024210.1	Mus musculus RIKEN cDNA 2310033P09 gene (2310033P09Rik), mRNA.
scl066522.1_109-S	-22.8442	-1.6	Pgpep1	NM_023217.2	
scl21820.14.1_60-S	-22.7694	-5.19	Vps45	NM_013841.1	Mus musculus vacuolar protein sorting 45 (yeast) (Vps45), mRNA.
scl44251.7.1_3-S	-22.766	-2.06	Stard3nl	NM_024270.1	Mus musculus STARD3 N-terminal like (Stard3nl), mRNA.
scl023825.3_146-S	-22.7348	-1.22	Banf1	NM_011793.2	Mus musculus barrier to autointegration factor 1 (Banf1), mRNA.
scl21665.7.1_0-S	-22.729	-1.52	0610005A07Rik	XM_359308.1	Mus musculus RIKEN cDNA 0610005A07 gene (0610005A07Rik), mRNA.
scl019684.7_10-S	-22.7245	-2.31	Rdx	NM_009041	Mus musculus radixin (Rdx), mRNA.
scl39660.7_333-S	-22.7186	-1.6	AI415282	NM_134021.1	Mus musculus expressed sequence AI415282 (AI415282), mRNA.
scl00109019.2_207-S	-22.7143	-3.26	5830411E10Rik	NM_028696.1	Mus musculus RIKEN cDNA 5830411E10 gene (5830411E10Rik), mRNA.

scl48137.14.1_1-S	-22.6957	-1.24	Oxct1	NM_024188.3	
scl056043.1_7-S	-22.6802	-1.37	Akr1e1	NM_018859.1	Mus musculus aldo-keto reductase family 1, member E1 (Akr1e1), mRNA.
scl52793.9.1_8-S	-22.6702	-4.03	Unc93b	NM_019449.1	Mus musculus unc-93 homolog B (C. elegans) (Unc93b), mRNA.
scl068713.2_9-S	-22.6672	-1.42	Ifitm1	NM_026820	Mus musculus interferon induced transmembrane protein 1 (Ifitm1), mRNA.
scl00226251.1_194-S	-22.6635	-6.4	Ablim1	NM_178688.2	Mus musculus actin-binding LIM protein 1 (Ablim1), mRNA.
scl54681.4_59-S	-22.6071	-1.31	Sh3bgrl	NM_019989.2	Mus musculus SH3-binding domain glutamic acid-rich protein like (Sh3bgrl), mRNA.
scl0070316.1_255-S	-22.5864	-3.18	Ndufab1	NM_028177.2	Mus musculus NADH dehydrogenase (ubiquinone) 1, alpha/beta subcomplex, 1 (Ndufab1), mRNA.
scl42524.16_317-S	-22.5573	-2.33	Scin	NM_009132.1	Mus musculus scinderin (Scin), mRNA.
scl0001436.1_98-S	-22.5072	-1.2	Commd1	NM_144514.1	Mus musculus COMM domain containing 1 (Commd1), mRNA.
scl071780.11_24-S	-22.4805	-2.74	Isyna1	NM_023627.1	Mus musculus myo-inositol 1-phosphate synthase A1 (Isyna1), mRNA.
scl32044.6_195-S	-22.4218	-1.32	Cdipt	NM_138754.1	Mus musculus CDP-diacylglycerol--inositol 3-phosphatidyltransferase (phosphatidylinositol synthase) (Cdipt), mRNA.
scl069890.1_204-S	-22.4171	-1.58	Zfp219	NM_027248	Mus musculus zinc finger protein 219 (Zfp219), mRNA.
scl47406.11_619-S	-22.4022	-3.28	Slc1a3	NM_148938.2	Mus musculus solute carrier family 1 (glial high affinity glutamate transporter), member 3 (Slc1a3), mRNA.
scl0003429.1_37-S	-22.3979	-1.33	Hmbs	NM_013551	Mus musculus hydroxymethylbilane synthase (Hmbs), mRNA.
scl000935.1_50-S	-22.3943	-1.8	Tfb2m	XM_122407.1	Mus musculus transcription factor B2, mitochondrial (Tfb2m), mRNA.
scl17197.4_15-S	-22.3887	-1.65	Tagln2	NM_178598.1	Mus musculus transgelin 2 (Tagln2), mRNA.
scl074194.1_11-S	-22.3689	-3.76	Rhoe	NM_028810.1	Mus musculus ras homolog gene family, member E (Rhoe), mRNA.
scl00101206.2_281-S	-22.3673	-1.37	Tada3l	NM_133932.1	Mus musculus transcriptional adaptor 3 (NGG1 homolog, yeast)-like (Tada3l), mRNA.
scl018247.9_19-S	-22.3509	-1.27	Oaz2	NM_010952.2	Mus musculus ornithine decarboxylase antizyme 2 (Oaz2), mRNA.
scl053606.2_17-S	-22.3284	-2.26	G1p2	NM_015783.1	Mus musculus interferon, alpha-inducible protein (G1p2), mRNA.
scl098496.1_28-S	-22.2809	-1.65	AL024069	XM_129912.3	Mus musculus expressed sequence AL024069 (AL024069), mRNA.
scl54611.2_16-S	-22.2519	-1.9	Tceal1	NM_146236.1	Mus musculus transcription elongation factor A (SII)-like 1 (Tceal1), mRNA.
scl30749.5_442-S	-22.2168	-1.51	Gprc5b	NM_022420.1	Mus musculus G protein-coupled receptor, family C, group 5, member B (Gprc5b), mRNA.
scl000472.1_13-S	-22.2086	-1.62	1110011F09Rik	NM_029576.1	Mus musculus RIKEN cDNA 1110011F09 gene (1110011F09Rik), mRNA.
scl0003116.1_14-S	-22.1744	-1.64	Gnas	NM_010309.1	Mus musculus GNAS (guanine nucleotide binding protein, alpha stimulating) complex locus (Gnas), mRNA.
scl30960.6.1_9-S	-22.1586	-2.12	Folr2	NM_008035	Mus musculus folate receptor 2 (fetal) (Folr2), mRNA.
scl0001167.1_16-S	-22.1534	-3.35	Lmcd1	NM_144799.1	Mus musculus LIM and cysteine-rich domains 1 (Lmcd1), mRNA.
scl41340.2.1_55-S	-22.1422	-3.65	1110030J09Rik	NM_025397.2	Mus musculus RIKEN cDNA 1110030J09 gene (1110030J09Rik), mRNA.
scl34186.14_327-S	-22.1379	-1.37	Abcb10	NM_019552.1	Mus musculus ATP-binding cassette, sub-family B (MDR/TAP), member 10 (Abcb10), nuclear gene encoding mitochondrial protein, mRNA.
scl012988.1_164-S	-22.1295	-1.76	Csk	NM_007783.2	Mus musculus c-src tyrosine kinase (Csk), mRNA.
scl23400.7_310-S	-22.0975	-3.53	Stmn2	NM_025285.1	Mus musculus stathmin-like 2 (Stmn2), mRNA.
scl0001675.1_34-S	-22.0584	-5.13	Tbp	NM_013684.1	Mus musculus TATA box binding protein (Tbp), mRNA.
scl32923.6.1_33-S	-22.0452	-2.26	Exosc5	NM_138586.1	Mus musculus exosome component 5 (Exosc5), mRNA.
scl54629.5_192-S	-21.9922	-1.68	Armcx1	NM_030066.2	
scl38265.24.1_4-S	-21.975	-1.64	Itga7	NM_008398.1	Mus musculus integrin alpha 7 (Itga7), mRNA.
scl0210106.1_119-S	-21.9653	-1.54	Pols	NM_198600.1	Mus musculus polymerase (DNA directed) sigma (Pols), mRNA.
scl0002324.1_12-S	-21.9604	-1.73	Synj2bp	NM_025292.2	Mus musculus synaptojanin 2 binding protein (Synj2bp), mRNA.
scl067956.13_1-S	-21.9537	-1.32	2410195B05Rik	NM_030241.2	Mus musculus RIKEN cDNA 2410195B05 gene (2410195B05Rik), mRNA.
scl30477.6_12-S	-21.9445	-1.78	2700078K21Rik	NM_028308.1	
scl071966.3_0-S	-21.88	-1.28	Nkiras2	NM_028024.1	Mus musculus NFKB inhibitor interacting Ras-like protein 2 (Nkiras2), mRNA.
scl067678.4_130-S	-21.8752	-1.31	Lsm3	NM_026309.1	Mus musculus LSM3 homolog, U6 small nuclear RNA associated (S. cerevisiae) (Lsm3), mRNA.
scl0004066.1_8-S	-21.8581	-2.93	Asl	NM_133768.1	Mus musculus argininosuccinate lyase (Asl), mRNA.
scl0016998.2_125-S	-21.8349	-1.38	Ltbp3	NM_008520.1	Mus musculus latent transforming growth factor beta binding protein 3 (Ltbp3), mRNA.
scl022381.3_136-S	-21.809	-1.3	Wbp5	NM_011712.1	Mus musculus WW domain binding protein 5 (Wbp5), mRNA.
scl37205.3.1_21-S	-21.7647	-1.37	1810008A14Rik	NM_025457.1	Mus musculus RIKEN cDNA 1810008A14 gene (1810008A14Rik), mRNA.
scl53527.2.1_48-S	-21.7573	-2.94	2410004C24Rik	NM_133678.2	Mus musculus RIKEN cDNA 2410004C24 gene (2410004C24Rik), mRNA.
scl29898.5_140-S	-21.7184	-1.31	Vps24	NM_025783.2	Mus musculus vacuolar protein sorting 24 (yeast) (Vps24), mRNA.
scl067163.2_42-S	-21.7	-2.62	2610204L23Rik	NM_026009.1	Mus musculus RIKEN cDNA 2610204L23 gene (2610204L23Rik), mRNA.
scl33370.7_212-S	-21.6862	-1.31	1810044O22Rik	NM_025558.2	Mus musculus RIKEN cDNA 1810044O22 gene (1810044O22Rik), mRNA.
gi_7305154_ref_NM_013	-21.6612	-1.25	Hprt	NM_013556.1	Mus musculus hypoxanthine guanine phosphoribosyl transferase (Hprt), mRNA.
scl014745.1_6-S	-21.6555	-1.45	Edg2	NM_010336	Mus musculus endothelial differentiation, lysophosphatidic acid G-protein-coupled receptor, 2 (Edg2), mRNA.
scl0105239.1_62-S	-21.6218	-1.2	Rnf44	NM_134064.1	Mus musculus ring finger protein 44 (Rnf44), mRNA.
scl0072133.2_35-S	-21.608	-5.02	2610009I02Rik	NM_028115.2	Mus musculus RIKEN cDNA 2610009I02 gene (2610009I02Rik), mRNA.
scl0059069.1_170-S	-21.6062	-1.24	Tpm3	NM_022314.2	Mus musculus tropomyosin 3, gamma (Tpm3), mRNA.
scl017256.3_8-S	-21.5952	-2.08	Mea1	NM_010787.1	Mus musculus male enhanced antigen 1 (Mea1), mRNA.
scl0112405.12_70-S	-21.5809	-1.37	Egln1	NM_053207.1	Mus musculus EGL nine homolog 1 (C. elegans) (Egln1), mRNA.

scl15925.5_61-S	-21.5648	-1.37	Pea15	NM_008556.1	Mus musculus phosphoprotein enriched in astrocytes 15 (Pea15), mRNA.
scl0059091.1_151-S	-21.5553	-2.44	Jph2	NM_021566.1	Mus musculus junctophilin 2 (Jph2), mRNA.
scl0001589.1_43-S	-21.5324	-4.49	AI415282	NM_134021.1	Mus musculus expressed sequence AI415282 (AI415282), mRNA.
scl0001950.1_6-S	-21.5286	-1.65	Tpm3	NM_022314	Mus musculus tropomyosin 3, gamma (Tpm3), mRNA.
scl00244416.2_151-S	-21.4923	-2.94	Ppp1r3b	NM_177741.2	Mus musculus protein phosphatase 1, regulatory (inhibitor) subunit 3B (Ppp1r3b), mRNA.
scl0026992.2_59-S	-21.4771	-1.33	Brd7	NM_012047.1	Mus musculus bromodomain containing 7 (Brd7), mRNA.
scl45163.12.1_28-S	-21.4032	-1.87	2610025M23Rik	NM_029578	Mus musculus RIKEN cDNA 2610025M23 gene (2610025M23Rik), mRNA.
scl071810.6_21-S	-21.395	-4.74	2610024N24Rik	XM_128698.5	Mus musculus RIKEN cDNA 2610024N24 gene (2610024N24Rik), mRNA.
scl0059043.2_22-S	-21.39	-1.19	Wsb2	NM_021539.3	Mus musculus WD repeat and SOCS box-containing 2 (Wsb2), mRNA.
scl19453.6.2634_3-S	-21.3497	-1.79	A130092J06Rik	NM_175511.2	Mus musculus RIKEN cDNA A130092J06 gene (A130092J06Rik), mRNA.
scl000524.1_1771-S	-21.3419	-2.22	Gcnt1	NM_173442.1	Mus musculus glucosaminyl (N-acetyl) transferase 1, core 2 (Gcnt1), mRNA.
scl000275.1_39-S	-21.2939	-1.24	3010033P07Rik	NM_029767.1	Mus musculus RIKEN cDNA 3010033P07 gene (3010033P07Rik), mRNA.
scl022041.3_13-S	-21.2018	-1.51	Trf	NM_133977.1	Mus musculus transferrin (Trf), mRNA.
scl40969.4.1_13-S	-21.1637	-1.98	Mrpl10	NM_026154.1	Mus musculus mitochondrial ribosomal protein L10 (Mrpl10), mRNA.
scl0001081.1_37-S	-21.1491	-1.76	Camk1	NM_133926.1	Mus musculus calcium/calmodulin-dependent protein kinase I (Camk1), mRNA.
scl47631.9.1_4-S	-21.0988	-1.48	5730592L21Rik	NM_029720.1	Mus musculus RIKEN cDNA 5730592L21 gene (5730592L21Rik), mRNA.
scl017470.1_35-S	-21.0961	-1.38	Cd200	NM_010818	
scl0001816.1_0-S	-21.0959	-1.63	Eif4a2	XM_147230.1	Mus musculus eukaryotic translation initiation factor 4A2 (Eif4a2), mRNA.
scl31415.7_3-S	-21.047	-1.19	2310044H10Rik	NM_197991	Mus musculus RIKEN cDNA 2310044H10 gene (2310044H10Rik), mRNA.
scl0055989.2_71-S	-21.0296	-1.58	Nol5	NM_018868.1	Mus musculus nucleolar protein 5 (Nol5), mRNA.
scl0003640.1_4-S	-20.9941	-1.38	Ptcd2	XM_127497.2	
scl21140.12.1_50-S	-20.9808	-3.31	Dbh	NM_138942.2	Mus musculus dopamine beta hydroxylase (Dbh), mRNA.
scl0023950.1_557-S	-20.9769	-1.75	Dnajb6	NM_011847.1	Mus musculus DnaJ (Hsp40) homolog, subfamily B, member 6 (Dnajb6), mRNA.
scl38324.4.1_0-S	-20.9673	-1.56	Ddit3	NM_007837.2	Mus musculus DNA-damage inducible transcript 3 (Ddit3), mRNA.
scl0003223.1_29-S	-20.9646	-1.45	Mrrf	NM_026422.1	Mus musculus mitochondrial ribosome recycling factor (Mrrf), mRNA.
scl057265.1_17-S	-20.9627	-4.53	Fzd2	NM_020510.1	Mus musculus frizzled homolog 2 (Drosophila) (Fzd2), mRNA.
scl000082.1_54-S	-20.9327	-3.14	Nr1d1	NM_145434.1	Mus musculus nuclear receptor subfamily 1, group D, member 1 (Nr1d1), mRNA.
scl49266.7.1_79-S	-20.9174	-1.37	Hes1	NM_008235.2	Mus musculus hairy and enhancer of split 1 (Drosophila) (Hes1), mRNA.
scl0320067.2_10-S	-20.9051	-3.32	Tnni3k	NM_177066.2	
scl00242297.2_152-S	-20.9038	-1.6	1700012H17Rik	NM_173426.1	Mus musculus RIKEN cDNA 1700012H17 gene (1700012H17Rik), mRNA.
scl020055.2_9-S	-20.8921	-1.22	Rps16	NM_013647.1	Mus musculus ribosomal protein S16 (Rps16), mRNA.
scl0067495.2_241-S	-20.8633	-1.41	2010200O16Rik	NM_026198.1	Mus musculus RIKEN cDNA 2010200O16 gene (2010200O16Rik), mRNA.
scl53053.7.1_7-S	-20.8481	-1.75	As3mt	NM_020577.1	
scl026557.1_7-S	-20.8187	-1.28	Homer2	XM_133550.4	Mus musculus homer homolog 2 (Drosophila) (Homer2), mRNA.
scl37690.5.564_26-S	-20.8169	-1.27	Gna11	NM_010301.1	Mus musculus guanine nucleotide binding protein, alpha 11 (Gna11), mRNA.
scl0015531.1_129-S	-20.779	-1.62	Ndst1	NM_008306.2	Mus musculus N-deacetylase/N-sulfotransferase (heparan glucosaminyl) 1 (Ndst1), mRNA.
scl017283.2_24-S	-20.7643	-2.45	Men1	NM_008583.1	Mus musculus multiple endocrine neoplasia 1 (Men1), mRNA.
scl0066421.2_54-S	-20.7517	-1.36	2410004B18Rik	NM_025555.1	Mus musculus RIKEN cDNA 2410004B18 gene (2410004B18Rik), mRNA.
scl23476.5_13-S	-20.7299	-1.25	Vamp3	NM_009498.3	Mus musculus vesicle-associated membrane protein 3 (Vamp3), mRNA.
scl40865.6.1_13-S	-20.71	-1.55	G6pc3	NM_175935.2	Mus musculus glucose 6 phosphatase, catalytic, 3 (G6pc3), mRNA.
scl066163.7_0-S	-20.7027	-1.3	Mrpl4	NM_023167.1	Mus musculus mitochondrial ribosomal protein L4 (Mrpl4), mRNA.
scl0058194.2_124-S	-20.652	-1.23	Sh3kbp1	NM_021389.3	Mus musculus SH3-domain kinase binding protein 1 (Sh3kbp1), mRNA.
scl0228608.7_126-S	-20.6338	-2.06	Smox	NM_145533.1	Mus musculus spermine oxidase (Smox), mRNA.
scl099003.2_10-S	-20.6041	-1.76	4732486I23Rik	XM_130565.3	Mus musculus RIKEN cDNA 4732486I23 gene (4732486I23Rik), mRNA.
scl24746.3_22-S	-20.5995	-1.21	Hspb7	NM_013868.2	Mus musculus heat shock protein family, member 7 (cardiovascular) (Hspb7), mRNA.
scl41542.4_321-S	-20.5942	-1.3	Gm2a	NM_010299.2	Mus musculus GM2 ganglioside activator protein (Gm2a), mRNA.
scl25892.5.96_2-S	-20.5877	-1.5	Ap1s1	NM_007457.1	Mus musculus adaptor protein complex AP-1, sigma 1 (Ap1s1), mRNA.
scl066496.2_57-S	-20.5863	-3.12	2700038C09Rik	NM_025598.1	Mus musculus RIKEN cDNA 2700038C09 gene (2700038C09Rik), mRNA.
scl40146.10.1_50-S	-20.5637	-1.4	Atpaf2	NM_145427.1	Mus musculus ATP synthase mitochondrial F1 complex assembly factor 2 (Atpaf2), mRNA.
scl0002139.1_4-S	-20.5581	-5.14	5730537D05Rik	NM_027495.2	Mus musculus RIKEN cDNA 5730537D05 gene (5730537D05Rik), mRNA.
scl0003295.1_37-S	-20.5493	-4.73	Crat	NM_007760.1	Mus musculus carnitine acetyltransferase (Crat), mRNA.
scl00208922.2_306-S	-20.5355	-4.33	Cpeb3	NM_198300.1	Mus musculus cytoplasmic polyadenylation element binding protein 3 (Cpeb3), mRNA.
scl0018829.1_65-S	-20.5097	-1.71	Ccl21b	NM_011124.3	Mus musculus chemokine (C-C motif) ligand 21b (serine) (Ccl21b), mRNA.
scl0217779.3_307-S	-20.4931	-1.66	2610022K04Rik	NM_153121.1	Mus musculus RIKEN cDNA 2610022K04 gene (2610022K04Rik), mRNA.
scl0071078.2_199-S	-20.4669	-2.71	Adam30	NM_027665.1	Mus musculus a disintegrin and metalloproteinase domain 30 (Adam30), mRNA.
scl39937.19_289-S	-20.4662	-1.18	Rpa1	NM_026653.1	Mus musculus replication protein A1 (Rpa1), mRNA.

scl0014312.2_62-S	-20.4626	-1.38	Brd2	NM_010238.1	Mus musculus bromodomain containing 2 (Brd2), mRNA.
scl42309.7.1_14-S	-20.4562	-1.17	Vti1b	NM_016800.2	Mus musculus vesicle transport through interaction with t-SNAREs 1B homolog (Vti1b), mRNA.
scl0075423.2_59-S	-20.4534	-2.59	2810410P22Rik	NM_182994.1	
scl00101739.1_35-S	-20.4466	-1.5	Psip1	NM_133948.2	Mus musculus PC4 and SFRS1 interacting protein 1 (Psip1), mRNA.
scl020116.3_11-S	-20.4459	-1.26	Rps8	NM_009098.2	Mus musculus ribosomal protein S8 (Rps8), mRNA.
scl072265.1_70-S	-20.422	-1.27	Tram1	NM_028173.1	Mus musculus translocating chain-associating membrane protein 1 (Tram1), mRNA.
scl46998.15.1_4-S	-20.3832	-1.41	Eif3s7	NM_018749	Mus musculus eukaryotic translation initiation factor 3, subunit 7 (zeta) (Eif3s7), mRNA.
scl000941.1_0-S	-20.3706	-1.2	Ndufs2	NM_153064.3	Mus musculus NADH dehydrogenase (ubiquinone) Fe-S protein 2 (Ndufs2), mRNA.
scl0051812.2_78-S	-20.3424	-1.41	Mcrs1	NM_016766.2	Mus musculus microspherule protein 1 (Mcrs1), mRNA.
scl39384.39.1_1-S	-20.3123	-1.46	Abca8a	NM_153145.1	Mus musculus ATP-binding cassette, sub-family A (ABC1), member 8a (Abca8a), mRNA.
scl0064138.2_328-S	-20.3026	-1.33	Ctsz	NM_022325.3	Mus musculus cathepsin Z (Ctsz), mRNA.
scl55019.13_214-S	-20.2434	-1.48	Pctk1	NM_011049.2	Mus musculus PCTAIRE-motif protein kinase 1 (Pctk1), mRNA.
scl00072.1_32-S	-20.2209	-2.1	Rab6	NM_024287.2	Mus musculus RAB6, member RAS oncogene family (Rab6), mRNA.
scl24711.3.1_0-S	-20.2068	-1.74	Nppb	NM_008726.2	Mus musculus natriuretic peptide precursor type B (Nppb), mRNA.
scl0003006.1_310-S	-20.1897	-1.36	Fbxo3	NM_212433.1	Mus musculus F-box only protein 3 (Fbxo3), transcript variant 2, mRNA.
scl21150.5.1_23-S	-20.1848	-1.7	B230317C12Rik	NM_019833.1	Mus musculus RIKEN cDNA B230317C12 gene (B230317C12Rik), mRNA.
scl33662.13_427-S	-20.1526	-1.61	Hmgb2l1	NM_178017.1	Mus musculus high mobility group box 2-like 1 (Hmgb2l1), mRNA.
scl0001977.1_31-S	-20.124	-1.53	Dap3	NM_022994.2	Mus musculus death associated protein 3 (Dap3), mRNA.
scl50952.14_117-S	-20.1069	-1.31	1200007D18Rik	NM_026170.2	Mus musculus RIKEN cDNA 1200007D18 gene (1200007D18Rik), mRNA.
scl49447.9_394-S	-20.1057	-1.67	Pmm2	XM_147219.1	Mus musculus phosphomannomutase 2 (Pmm2), mRNA.
scl000496.1_7-S	-20.1008	-3.61	Tpt1h	NM_153597.1	Mus musculus tRNA splicing 2 phosphotransferase 1 homolog (S. cerevisiae) (Tpt1h), mRNA.
scl33059.9.1_102-S	-20.0825	-1.44	Kptn	NM_133727.1	Mus musculus kaptin (Kptn), mRNA.
scl18523.10_225-S	-20.0703	-1.46	2310001A20Rik	NM_027977.1	Mus musculus RIKEN cDNA 2310001A20 gene (2310001A20Rik), mRNA.
scl00192287.1_146-S	-20.0526	-3.6	C330005L02Rik	NM_138756.2	Mus musculus RIKEN cDNA C330005L02 gene (C330005L02Rik), mRNA.
scl50681.9.1_22-S	-20.0414	-1.2	D17Wsu94e	NM_145353.1	Mus musculus DNA segment, Chr 17, Wayne State University 94, expressed (D17Wsu94e), mRNA.
scl069668.1_14-S	-20.0397	-1.27	2310061I09Rik	XM_129811.2	Mus musculus RIKEN cDNA 2310061I09 gene (2310061I09Rik), mRNA.
scl27078.4.1_81-S	-20.03	-1.22	0910001L09Rik	XM_132434.2	Mus musculus RIKEN cDNA 0910001L09 gene (0910001L09Rik), mRNA.
scl016202.8_23-S	-20.0293	-1.44	Ilk	NM_010562.1	Mus musculus integrin linked kinase (Ilk), mRNA.
scl00319263.1_175-S	-20.0292	-1.67	A030012M09Rik	NM_183028.1	Mus musculus RIKEN cDNA A030012M09 gene (A030012M09Rik), mRNA.

TABLE A6

GENMAPPS AND GENE ONTOLOGIES SIGNIFICANTLY CHANGED BY THE ATHEROGENIC DIET IN HEARTS

UP-REGULATED - GENMAPP

No.	MAPP Name	Number Changed	Number Measured	Number on MAPP	Percent Changed	Percent Present	Z Score	Permuted P Value
1	Mm_Fatty_Acid_Beta_Oxidation_1_BiGCaT	10	26	27	38.4615	96.2963	4.8100	0.0000
2	Mm_Fatty_Acid_Beta_Oxidation_Meta_BiGCaT	11	31	32	35.4839	96.8750	4.7030	0.0000
3	Mm_Delta-Notch_NetPath_3	19	77	80	24.6753	96.2500	4.2780	0.0000
4	Mm_Fatty_acid_metabolism	14	50	66	28.0000	75.7576	4.2210	0.0000
5	Mm_TNF-alpha-NF-kB_NetPath_9	31	166	176	18.6747	94.3182	3.7330	0.0000
6	Mm_Valine_leucine_and_isoleucine_degradation	9	26	44	34.6154	59.0909	4.1570	0.0020
7	Mm_Glutamate_metabolism	5	13	40	38.4615	32.5000	3.3960	0.0020
8	Mm_Mitochondrial_fatty_acid_betaoxidation	6	16	16	37.5000	100.0000	3.6410	0.0040
9	Mm_Translation_Factors	12	49	50	24.4898	98.0000	3.3580	0.0050
10	Mm_Heme_Biosynthesis	4	9	9	44.4444	100.0000	3.4200	0.0090
11	Mm_mRNA_processing_binding_Reactome	59	426	551	13.8498	77.3140	2.6980	0.0090

UP-REGULATED - GENE ONTOLOGY

No.	GO Name	GO Type	Number Changed	Number Measured	Number in GO	Percent Changed	Percent Present	Z Score	Permuted P Value
1	cellular physiological process	P	871	8916	10403	9.7690	85.7061	7.5060	0.0000
2	metabolism	P	682	6705	7938	10.1715	84.4671	7.1510	0.0000
3	ubiquitin cycle	P	69	437	511	15.7895	85.5186	5.6690	0.0000
4	protein modification	P	177	1476	1665	11.9919	88.6487	5.2710	0.0000
5	protein metabolism	P	308	2842	3423	10.8374	83.0266	5.2510	0.0000
6	NLS-bearing substrate import into nucleus	P	4	7	7	57.1429	100.0000	4.6550	0.0000
7	fatty acid metabolism	P	28	156	164	17.9487	95.1220	4.3330	0.0000
8	chromosome organization and biogenesis	P	41	272	354	15.0735	76.8362	4.0170	0.0000
9	chromatin modification	P	22	125	133	17.6000	93.9850	3.7330	0.0000
10	chromosome organization and biogenesis (sensu Eukaryota)	P	37	251	332	14.7410	75.6024	3.6650	0.0000
11	protein ubiquitination	P	24	147	173	16.3265	84.9711	3.4910	0.0000
12	protein catabolism	P	29	190	215	15.2632	88.3721	3.4430	0.0000
13	RNA processing	P	44	329	371	13.3739	88.6792	3.3020	0.0000
14	RNA splicing	P	23	144	167	15.9722	86.2276	3.3010	0.0000
15	protein transport	P	70	589	654	11.8846	90.0612	3.1280	0.0000
16	heme biosynthesis	P	5	11	11	45.4545	100.0000	4.4370	0.0010
17	proteasomal ubiquitin-dependent protein catabolism	P	7	20	22	35.0000	90.9091	4.2970	0.0010
18	regulation of fatty acid metabolism	P	4	8	8	50.0000	100.0000	4.2480	0.0010
19	ER-associated protein catabolism	P	5	12	12	41.6667	100.0000	4.1610	0.0010
20	translational elongation	P	7	23	28	30.4348	82.1429	3.8180	0.0010
21	transport	P	255	2540	2884	10.0394	88.0721	3.3070	0.0010
22	protein localization	P	74	644	714	11.4907	90.1961	2.9080	0.0010
23	glucocorticoid receptor signaling pathway	P	3	4	4	75.0000	100.0000	4.8070	0.0020
24	electron transport	P	51	386	461	13.2124	83.7310	3.4680	0.0020
25	generation of precursor metabolites and energy	P	73	598	722	12.2074	82.8255	3.4430	0.0020
26	mRNA metabolism	P	28	196	223	14.2857	87.8924	3.0010	0.0020
27	lipid metabolism	P	68	581	616	11.7040	94.3182	2.9450	0.0020
28	protein import into nucleus	P	13	65	72	20.0000	90.2778	3.3860	0.0030
29	Notch signaling pathway	P	9	39	43	23.0769	90.6977	3.3150	0.0030
30	establishment of protein localization	P	70	617	684	11.3452	90.2047	2.7100	0.0030
31	nuclear mRNA splicing\, via spliceosome	P	18	118	135	15.2542	87.4074	2.7030	0.0030
32	protein amino acid C-linked glycosylation via 2'-alpha-mannosyl-L-tryptophan	P	2	2	2	100.0000	100.0000	4.6750	0.0040
33	protein amino acid O-linked mannosylation	P	2	2	2	100.0000	100.0000	4.6750	0.0040
34	Golgi organization and biogenesis	P	4	9	9	44.4444	100.0000	3.9040	0.0040
35	fatty acid beta-oxidation	P	6	20	20	30.0000	100.0000	3.4900	0.0040
36	ubiquitin-dependent protein catabolism	P	22	132	149	16.6667	88.5906	3.4480	0.0040
37	fatty acid oxidation	P	8	32	32	25.0000	100.0000	3.3950	0.0040
38	nucleobase\, nucleoside\, nucleotide and nucleic acid metabolism	P	273	2803	3355	9.7396	83.5469	2.8760	0.0050
39	RNA elongation	P	4	10	10	40.0000	100.0000	3.6080	0.0070
40	malate metabolism	P	3	6	6	50.0000	100.0000	3.6780	0.0090
41	translational initiation	P	11	58	72	18.9655	80.5556	2.9130	0.0090
42	binding	F	864	9101	10565	9.4935	86.1429	6.1730	0.0000
43	nucleotide binding	F	212	1750	1959	12.1143	89.3313	5.9960	0.0000
44	catalytic activity	F	485	4686	5420	10.3500	86.4576	5.8780	0.0000
45	translation regulator activity	F	28	127	151	22.0473	84.1060	5.5790	0.0000
46	acyl-CoA dehydrogenase activity	F	7	16	16	43.7500	100.0000	5.1070	0.0000
47	ubiquitin-protein ligase activity	F	39	219	259	17.8082	84.5560	5.0690	0.0000
48	molybdenum ion binding	F	3	4	4	75.0000	100.0000	4.8070	0.0000
49	ligase activity	F	56	382	449	14.6597	85.0780	4.4830	0.0000
50	translation elongation factor activity	F	10	34	37	29.4118	91.8919	4.4290	0.0000
51	ATP binding	F	137	1180	1331	11.6102	88.6551	4.1680	0.0000
52	protein binding	F	457	4715	5185	9.6925	90.9354	3.9290	0.0000
53	ATPase activity	F	45	312	360	14.4231	86.6667	3.8890	0.0000
54	RNA binding	F	61	466	548	13.0901	85.0365	3.7240	0.0000
55	oxidoreductase activity	F	81	662	775	12.2357	85.4194	3.6580	0.0000
56	enzyme binding	F	28	179	198	15.6425	90.4040	3.5250	0.0000
57	transcription factor binding	F	42	325	350	12.9231	92.8571	2.9850	0.0000
58	cytochrome-c oxidase activity	F	7	21	27	33.3333	77.7778	4.1280	0.0010
59	malic enzyme activity	F	3	4	4	75.0000	100.0000	4.8070	0.0020
60	magnesium ion binding	F	37	253	273	14.6245	92.6740	3.6120	0.0020
61	nucleic acid binding	F	268	2709	3327	9.8929	81.4247	3.1350	0.0020
62	FAD binding	F	7	26	28	26.9231	92.8571	3.4130	0.0030
63	metal ion binding	F	285	2924	3379	9.7469	86.5345	2.9680	0.0030
64	zinc ion binding	F	161	1556	1866	10.3470	83.3869	2.9530	0.0040
65	voltage-gated ion-selective channel activity	F	3	4	5	75.0000	80.0000	4.8070	0.0050
66	electron transporter activity	F	25	175	195	14.2857	89.7436	2.8330	0.0050
67	long-chain-acyl-CoA dehydrogenase activity	F	2	2	2	100.0000	100.0000	4.6750	0.0060
68	ATPase activity\, coupled	F	35	266	301	13.1579	88.3721	2.8340	0.0060
69	myosin light chain kinase activity	F	2	2	3	100.0000	66.6667	4.6750	0.0070
70	Rab GDP-dissociation inhibitor activity	F	2	2	2	100.0000	100.0000	4.6750	0.0070

71	vascular endothelial growth factor receptor activity	F	5	13	13	38.4615	100.0000	3.9140	0.0080
72	2 iron\, 2 sulfur cluster binding	F	4	10	11	40.0000	90.9091	3.6080	0.0080
73	translation initiation factor activity	F	12	66	86	18.1818	76.7442	2.8780	0.0080
74	helicase activity	F	18	121	136	14.8760	88.9706	2.5870	0.0080
75	aldehyde oxidase activity	F	2	2	2	100.0000	100.0000	4.6750	0.0090
76	intracellular	C	740	6933	8118	10.6736	85.4028	9.4510	0.0000
77	cytoplasm	C	427	3484	3969	12.2560	87.7803	9.4350	0.0000
78	mitochondrion	C	118	730	822	16.1644	88.8078	7.7790	0.0000
79	mitochondrial envelope	C	52	280	329	18.5714	85.1064	6.2090	0.0000
80	protein complex	C	212	1750	2202	12.1143	79.4732	5.9960	0.0000
81	mitochondrial membrane	C	46	262	308	17.5573	85.0649	5.4050	0.0000
82	cytosol	C	53	344	383	15.4070	89.8172	4.7550	0.0000
83	mitochondrial inner membrane	C	38	232	275	16.3793	84.3636	4.4280	0.0000
84	mitochondrial electron transport chain	C	9	30	40	30.0000	75.0000	4.2760	0.0000
85	nucleus	C	333	3274	3798	10.1710	86.2033	4.1810	0.0000
86	ATP-binding cassette (ABC) transporter complex	C	4	8	8	50.0000	100.0000	4.2480	0.0010
87	Golgi apparatus	C	54	435	471	12.4138	92.3567	3.0780	0.0010
88	eukaryotic translation initiation factor 3 complex	C	3	5	5	60.0000	100.0000	4.1650	0.0020
89	endoplasmic reticulum	C	62	528	561	11.7424	94.1177	2.8350	0.0020
90	respiratory chain complex IV (sensu Eukaryota)	C	3	4	4	75.0000	100.0000	4.8070	0.0030
91	mannosyltransferase complex	C	2	2	2	100.0000	100.0000	4.6750	0.0040
92	transcription elongation factor complex	C	3	5	5	60.0000	100.0000	4.1650	0.0040
93	mitochondrial outer membrane	C	8	30	33	26.6667	90.9091	3.6160	0.0040
94	cellular component unknown	C	46	368	404	12.5000	91.0891	2.8840	0.0040
95	endoplasmic reticulum membrane	C	18	104	109	17.3077	95.4128	3.2950	0.0060
96	nuclear envelope	C	16	97	111	16.4948	87.3874	2.8910	0.0060
97	exocyst	C	3	6	6	50.0000	100.0000	3.6780	0.0070
98	flotillin complex	C	2	2	2	100.0000	100.0000	4.6750	0.0080
99	eukaryotic translation elongation factor 1 complex	C	3	6	6	50.0000	100.0000	3.6780	0.0080
100	DNA-directed RNA polymerase I complex	C	3	5	5	60.0000	100.0000	4.1650	0.0090
101	mitochondrial ribosome	C	8	35	37	22.8571	94.5946	3.0930	0.0090
102	mitochondrial matrix	C	13	74	76	17.5676	97.3684	2.8570	0.0090
103	microtubule	C	21	143	166	14.6853	86.1446	2.7320	0.0090

DOWN-REGULATED - GENMAPP

No.	MAPP Name	Number Changed	Number Measured	Number on MAPP	Percent Changed	Percent Present	Z Score	Permuted P Value
1	Mm_Ribosomal_Proteins	18	78	80	23.0769	97.5000	4.8800	0.0000
2	Mm_Proteasome_Degradation	16	73	81	21.9178	90.1235	4.3530	0.0000
3	Mm_Glycolysis_and_Gluconeogenesis	11	42	42	26.1905	100.0000	4.3080	0.0000
4	Mm_Circadian_Exercise	10	48	49	20.8333	97.9592	3.2430	0.0020
5	Mm_Translation_Factors	11	49	50	22.4490	98.0000	3.6930	0.0030
6	Mm_Glycolysis_Gluconeogenesis	10	42	61	23.8095	68.8525	3.7410	0.0040
7	Mm_Tetrachloroethene_degradation	3	5	12	60.0000	41.6667	4.2490	0.0050
8	Mm_Unsaturated_Fatty_Acid_Beta_Oxidation_BiGCaT	3	6	6	50.0000	100.0000	3.7580	0.0060
9	Mm_Alanine_and_aspartate_metabolism	5	13	42	38.4615	30.9524	4.0110	0.0070
10	Mm_mRNA_processing_binding_Reactome	49	426	551	11.5024	77.3140	2.6920	0.0090

DOWN-REGULATED - GENE ONTOLOGY

No.	GO Name	GO Type	Number Changed	Number Measured	Number in GO	Percent Changed	Percent Present	Z Score	Permuted P Value
1	metabolism	P	486	6706	7938	7.2472	84.4797	7.3550	0.0000
2	biosynthesis	P	121	1154	1428	10.4853	80.8123	7.2840	0.0000
3	cellular physiological process	P	598	8917	10403	6.7063	85.7157	6.4600	0.0000
4	protein metabolism	P	234	2843	3423	8.2307	83.0558	6.4510	0.0000
5	protein biosynthesis	P	69	588	801	11.7347	73.4082	6.4270	0.0000
6	proteasomal ubiquitin-dependent protein catabolism	P	7	20	22	35.0000	90.9091	5.6500	0.0000
7	ubiquitin-dependent protein catabolism	P	21	132	149	15.9091	88.5906	5.0740	0.0000
8	intracellular transport	P	66	648	740	10.1852	87.5676	5.0230	0.0000
9	protein polymerization	P	10	43	50	23.2558	86.0000	4.9660	0.0000
10	purine nucleotide biosynthesis	P	13	75	96	17.3333	78.1250	4.3500	0.0000
11	protein folding	P	25	200	227	12.5000	88.1057	4.1680	0.0000
12	generation of precursor metabolites and energy	P	57	598	722	9.5318	82.8255	4.1130	0.0000
13	ubiquitin cycle	P	42	437	511	9.6110	85.5186	3.5690	0.0000
14	actin filament severing	P	3	5	5	60.0000	100.0000	5.2330	0.0010
15	glycolysis	P	10	48	78	20.8333	61.5385	4.5230	0.0010
16	regulation of translational initiation	P	7	30	30	23.3333	100.0000	4.1650	0.0010
17	ATP synthesis coupled proton transport	P	9	45	60	20.0000	75.0000	4.1380	0.0010
18	protein transport	P	56	589	654	9.5076	90.0612	4.0550	0.0010
19	translation	P	21	168	201	12.5000	83.5821	3.8150	0.0010
20	protein catabolism	P	22	190	215	11.5790	88.3721	3.5100	0.0010
21	actin cytoskeleton organization and biogenesis	P	20	163	171	12.2699	95.3216	3.6300	0.0020
22	ER to Golgi vesicle-mediated transport	P	9	53	59	16.9811	89.8305	3.5430	0.0020
23	RNA splicing	P	18	144	167	12.5000	86.2276	3.5300	0.0020
24	muscle development	P	18	149	161	12.0805	92.5466	3.3690	0.0020
25	succinyl-CoA metabolism	P	2	2	2	100.0000	100.0000	5.7480	0.0030
26	intracellular protein transport	P	40	415	463	9.6386	89.6328	3.5000	0.0030
27	glucose metabolism	P	13	100	135	13.0000	74.0741	3.1530	0.0030
28	protein modification	P	109	1476	1665	7.3848	88.6487	2.9250	0.0030
29	barbed-end actin filament capping	P	5	21	21	23.8095	100.0000	3.5770	0.0040
30	mRNA processing	P	19	173	198	10.9827	87.3737	3.0070	0.0040
31	regulation of progression through cell cycle	P	35	375	410	9.3333	91.4634	3.0640	0.0050
32	purine ribonucleoside salvage	P	2	3	3	66.6667	100.0000	4.5510	0.0080
33	actin filament polymerization	P	5	25	27	20.0000	92.5926	3.0820	0.0080
34	proton transport	P	10	74	92	13.5135	80.4348	2.9010	0.0080
35	cell growth	P	15	135	143	11.1111	94.4056	2.7180	0.0080
36	mitotic spindle assembly	P	2	3	4	66.6667	75.0000	4.5510	0.0090
37	SMAD protein nuclear translocation	P	2	3	3	66.6667	100.0000	4.5510	0.0090
38	mitochondrial electron transport\, NADH to ubiquinone	P	4	14	25	28.5714	56.0000	3.6890	0.0090
39	unfolded protein response	P	3	9	9	33.3333	100.0000	3.5730	0.0090
40	protein amino acid methylation	P	5	23	25	21.7391	92.0000	3.3160	0.0090
41	structural constituent of ribosome	F	36	183	354	19.6721	51.6949	8.1920	0.0000

42	structural molecule activity	F	65	632	893	10.2848	70.7727	5.0680	0.0000
43	RNA binding	F	51	466	548	10.9442	85.0365	4.9500	0.0000
44	NADH dehydrogenase activity	F	8	31	43	25.8065	72.0930	4.8280	0.0000
45	protein binding	F	326	4716	5185	6.9126	90.9547	4.3200	0.0000
46	unfolded protein binding	F	20	146	159	13.6986	91.8239	4.1810	0.0000
47	actin binding	F	24	207	230	11.5942	90.0000	3.6750	0.0000
48	nucleotide binding	F	133	1750	1959	7.6000	89.3313	3.6320	0.0000
49	catalytic activity	F	313	4686	5420	6.6795	86.4576	3.4670	0.0000
50	cytoskeletal protein binding	F	32	315	344	10.1587	91.5698	3.4410	0.0000
51	threonine endopeptidase activity	F	6	18	21	33.3333	85.7143	5.0540	0.0010
52	NADH dehydrogenase (ubiquinone) activity	F	7	27	39	25.9259	69.2308	4.5320	0.0010
53	hydrogen-transporting ATPase activity\, rotational mechanism	F	9	45	58	20.0000	77.5862	4.1380	0.0010
54	translation regulator activity	F	16	127	151	12.5984	84.1060	3.3610	0.0010
55	electron carrier activity	F	10	67	80	14.9254	83.7500	3.2590	0.0010
56	thioredoxin peroxidase activity	F	2	2	2	100.0000	100.0000	5.7480	0.0020
57	antioxidant activity	F	9	51	56	17.6471	91.0714	3.6810	0.0030
58	hydrogen-transporting ATP synthase activity\, rotational mechanism	F	8	43	56	18.6047	76.7857	3.6500	0.0030
59	GTP binding	F	31	301	335	10.2990	89.8508	3.4680	0.0040
60	oxidoreductase activity	F	56	662	775	8.4592	85.4194	3.1210	0.0040
61	ligase activity	F	35	382	449	9.1623	85.0780	2.9470	0.0040
62	glucuronosyl-N-acetylglucosaminyl-proteoglycan 4-alpha-N-acetylglucosaminyltransferase activity	F	2	2	2	100.0000	100.0000	5.7480	0.0050
63	structural constituent of cytoskeleton	F	11	84	98	13.0952	85.7143	2.9260	0.0050
64	6-phosphofructokinase activity	F	2	3	3	66.6667	100.0000	4.5510	0.0060
65	protein serine/threonine phosphatase activity	F	6	32	36	18.7500	88.8889	3.1830	0.0060
66	ATPase activity\, coupled to transmembrane movement of substances	F	16	134	158	11.9403	84.8101	3.1230	0.0060
67	hydrolase activity\, acting on acid anhydrides\, catalyzing transmembrane movement of substances	F	16	136	161	11.7647	84.4721	3.0580	0.0070
68	molecular function unknown	F	37	404	455	9.1584	88.7912	3.0300	0.0070
69	carbonyl reductase (NADPH) activity	F	2	3	4	66.6667	75.0000	4.5510	0.0080
70	protein-L-isoaspartate (D-aspartate) O-methyltransferase activity	F	2	3	3	66.6667	100.0000	4.5510	0.0090
71	nucleoside-triphosphatase activity	F	42	498	565	8.4337	88.1416	2.6660	0.0090
72	cytoplasm	C	351	3484	3969	10.0746	87.7803	12.7100	0.0000
73	intracellular	C	554	6934	8118	7.9896	85.4151	11.2500	0.0000
74	mitochondrion	C	103	730	822	14.1096	88.8078	10.0340	0.0000
75	protein complex	C	182	1751	2202	10.3941	79.5186	9.0010	0.0000
76	ribosome	C	35	176	345	19.8864	51.0145	8.1550	0.0000
77	ribonucleoprotein complex	C	54	355	550	15.2113	64.5455	7.8110	0.0000
78	tubulin	C	3	3	3	100.0000	100.0000	7.0400	0.0000
79	proteasome complex (sensu Eukaryota)	C	11	37	42	29.7297	88.0952	6.3050	0.0000
80	mitochondrial ribosome	C	10	35	37	28.5714	94.5946	5.8360	0.0000
81	small ribosomal subunit	C	9	32	45	28.1250	71.1111	5.4710	0.0000
82	mitochondrial envelope	C	35	280	329	12.5000	85.1064	4.9450	0.0000
83	actin cytoskeleton	C	28	206	229	13.5922	89.9563	4.9110	0.0000
84	mitochondrial matrix	C	14	74	76	18.9189	97.3684	4.9100	0.0000
85	cytosol	C	40	344	383	11.6279	89.8172	4.7880	0.0000
86	mitochondrial inner membrane	C	30	232	275	12.9310	84.3636	4.7790	0.0000
87	Arp2/3 protein complex	C	4	8	8	50.0000	100.0000	5.4010	0.0010
88	proteasome core complex (sensu Eukaryota)	C	6	18	21	33.3333	85.7143	5.0540	0.0010
89	proton-transporting two-sector ATPase complex	C	9	47	61	19.1489	77.0492	3.9770	0.0010
90	proteasome regulatory particle (sensu Eukaryota)	C	3	6	7	50.0000	85.7143	4.6770	0.0020
91	large ribosomal subunit	C	7	29	42	24.1379	69.0476	4.2810	0.0020
92	cytoskeleton	C	63	742	859	8.4906	86.3795	3.3510	0.0020
93	endoplasmic reticulum lumen	C	4	14	14	28.5714	100.0000	3.6890	0.0040
94	endoplasmic reticulum	C	45	528	561	8.5227	94.1177	2.8380	0.0040
95	6-phosphofructokinase complex	C	2	3	3	66.6667	100.0000	4.5510	0.0060
96	interleukin-1 receptor complex	C	2	3	3	66.6667	100.0000	4.5510	0.0080
97	microvillus	C	3	9	10	33.3333	90.0000	3.5730	0.0090
