# Supplementary material for: Oil palm phenolics attenuate changes caused by an atherogenic diet in mice
Source: Eur J Nutr. 2012 Apr 11;52(2):443–56. doi: 10.1007/s00394-012-0346-0 (PMC3573186; doi:10.1007/s00394-012-0346-0)
Supplement: Supplementary file 3 — Supplementary material 3 (PDF 219 kb) [file 394_2012_346_MOESM3_ESM.pdf]

Article Title: Oil palm phenolics attenuate changes caused by an atherogenic diet in mice.

Journal Name: European Journal of Nutrition.

Author Names: Soon-Sen Leow, Shamala Devi Sekaran, Kalyana Sundram, YewAi Tan, Ravigadevi Sambanthamurthi.

Corresponding Author: Ravigadevi Sambanthamurthi; Malaysian Palm Oil Board, No. 6, Persiaran Institusi, Bandar Baru Bangi, 43000 Kajang, Selangor, Malaysia; E-mail: raviga@mpob.gov.my.

Supplementary Material 3

This file contains six tables which show lists of genes, as well as GenMAPPs and gene ontologies significantly changed by OPP in livers, spleens and hearts of mice. The |Differential Score| for all genes is more than 20, equivalent to a P Value of less than 0.01, with the exception of genes in the heart, which were selected using a |Differential Score| of more than 13, equivalent to a P Value of less than 0.05. All GenMAPPs and gene ontologies had Permuted P Values of less than 0.01, Numbers of Genes Changed of more than or equal to 2 and Z Scores of more than 2, except for those in the heart, which were selected using Permuted P Values of less than 0.05.

Table A7 Genes significantly changed by OPP in livers.

Table A8 GenMAPPs and gene ontologies significantly changed by OPP in livers.

Table A9 Genes significantly changed by OPP in spleens.

Table A10 GenMAPPs and gene ontologies significantly changed by OPP in spleens.

Table A11 Genes significantly changed by OPP in hearts.

Table A12 GenMAPPs and gene ontologies significantly changed by OPP in hearts.

TABLE A7					
GENES UP-REGULATED BY OPP IN LIVERS					
TargetID	Diff_Score	Fold_Change	Symbol	Accession	Definition
scl29051.11.1_27-S	62.1574	1.62	Cai	NM_009787.1	Mus musculus calcium binding protein, intestinal (Cai), mRNA.
scl0227613.1_83-S	45.7174	1.73	4930542G03Rik	NM_146116.1	Mus musculus RIKEN cDNA 4930542G03 gene (4930542G03Rik), mRNA.
scl020567.3_14-S	38.7127	1.42	Slp	NM_011413	Mus musculus sex-limited protein (Slp), mRNA.
scl40797.10.1_7-S	34.6073	1.35	Psmc5	NM_008950.1	Mus musculus protease (prosome, macropain) 26S subunit, ATPase 5 (Psmc5), mRNA.
scl49146.12.1_4-S	34.3051	1.7	2610033C09Rik	NM_026407.2	Mus musculus RIKEN cDNA 2610033C09 gene (2610033C09Rik), mRNA.
scl41025.7_232-S	33.1084	1.47	AA959742	NM_133807.1	Mus musculus expressed sequence AA959742 (AA959742), mRNA.
scl020382.2_66-S	32.0853	1.49	Sfrs2	NM_011358.1	Mus musculus splicing factor, arginine/serine-rich 2 (SC-35) (Sfrs2), mRNA.
scl49316.10.1_12-S	29.0872	1.89	Dnajb11	XM_148071.1	Mus musculus DnaJ (Hsp40) homolog, subfamily B, member 11 (Dnajb11), mRNA.
scl0080708.1_141-S	28.7693	1.42	Pacsin3	NM_028733.1	Mus musculus protein kinase C and casein kinase substrate in neurons 3 (Pacsin3), mRNA.
scl38328.12.1_41-S	28.2092	1.75	Galgt1	NM_008080.3	Mus musculus UDP-N-acetyl-alpha-D-galactosamine:(N-acetylneuraminyl)- galactosylglucosylceramide-beta-1, 4-N-acetylgalactosaminyltransferase (Galgt1), mRNA.
scl37681.14_50-S	27.7446	1.97	Slc41a2	NM_177388.2	
scl47392.22_290-S	26.8987	1.43	Rai14	NM_030690.2	Mus musculus retinoic acid induced 14 (Rai14), mRNA.
scl33485.7_10-S	26.7333	1.51	Herpud1	NM_022331.1	Mus musculus homocysteine-inducible, endoplasmic reticulum stress-inducible, ubiquitin-like domain member 1 (Herpud1), mRNA.
scl38868.13.1_10-S	26.0727	1.4	Pyp	NM_026438.2	Mus musculus pyrophosphatase (Pyp), mRNA.
scl37659.18.259_11-S	25.9447	1.35	Tra1	NM_011631.1	Mus musculus tumor rejection antigen gp96 (Tra1), mRNA.
scl41012.9.1_84-S	25.56	1.28	Ugalt2	NM_016752.1	Mus musculus UDP-galactose translocator 2 (Ugalt2), mRNA.
scl00224938.2_37-S	25.3174	1.33	Pja2	NM_144859.1	Mus musculus praja 2, RING-H2 motif containing (Pja2), mRNA.
scl47810.5_374-S	25.0248	1.45	1500031N24Rik	XM_128010.4	Mus musculus RIKEN cDNA 1500031N24 gene (1500031N24Rik), mRNA.
scl0003984.1_492-S	24.6608	1.55	Atp2a2	NM_009722.1	Mus musculus ATPase, Ca++ transporting, cardiac muscle, slow twitch 2 (Atp2a2), mRNA.
scl26742.12.1_68-S	24.6243	1.31	Eif2b4	NM_010122.1	Mus musculus eukaryotic translation initiation factor 2B, subunit 4 delta (Eif2b4), mRNA.
scl000086.1_135-S	24.2991	1.28	AA959742	NM_133807.1	Mus musculus expressed sequence AA959742 (AA959742), mRNA.
scl27100.19.1_2-S	23.9718	1.31	Trfr2	NM_015799.2	Mus musculus transferrin receptor 2 (Trfr2), mRNA.
scl20056.4.1_63-S	23.8768	1.34	Map1lc3a	NM_025735.1	Mus musculus microtubule-associated protein 1 light chain 3 alpha (Map1lc3a), mRNA.
scl37037.27_257-S	22.4045	2.04	Hyou1	NM_021395.2	Mus musculus hypoxia up-regulated 1 (Hyou1), mRNA.
scl24607.15_131-S	22.2705	1.45	Dvl1	NM_010091.2	Mus musculus dishevelled, dsh homolog 1 (Drosophila) (Dvl1), mRNA.
scl018816.1_117-S	21.5879	1.34	Serpinf2	NM_008878.1	Mus musculus serine (or cysteine) proteinase inhibitor, clade F, member 2 (Serpinf2), mRNA.
scl15961.15.1_3-S	21.4268	1.31	Uap1	NM_133806.2	Mus musculus UDP-N-acetylglucosamine pyrophosphorylase 1 (Uap1), mRNA.
scl020338.1_17-S	20.9782	1.29	Sel1h	NM_011344.1	Mus musculus Sel1 (suppressor of lin-12) 1 homolog (C. elegans) (Sel1h), mRNA.
scl53392.18_161-S	20.8154	1.47	Mta2	NM_011842.2	Mus musculus metastais-associated gene family, member 2 (Mta2), mRNA.
scl0012055.2_161-S	20.6824	1.35	Bcl7c	NM_009746	Mus musculus B-cell CLL/lymphoma 7C (Bcl7c), mRNA.
scl0269523.2_3-S	20.5116	1.23	Vcp	NM_009503.2	Mus musculus valosin containing protein (Vcp), mRNA.
scl0023881.1_86-S	20.2451	1.31	E430034L04Rik	NM_011816.2	Mus musculus RIKEN cDNA E430034L04 gene (E430034L04Rik), mRNA.
scl55073.12_278-S	20.1097	1.74	Tcfe3	NM_172472.1	Mus musculus transcription factor E3 (Tcfe3), mRNA.
scl52839.2_29-S	20.087	1.35	AI837181	NM_134149.1	Mus musculus expressed sequence AI837181 (AI837181), mRNA.
scl0003591.1_99-S	20.0477	1.48	Keap1	NM_016679.2	Mus musculus kelch-like ECH-associated protein 1 (Keap1), mRNA.
GENES DOWN-REGULATED BY OPP IN LIVERS					
TargetID	Diff_Score	Fold_Change	Symbol	Accession	Definition
scl37827.3_399-S	-68.574	-1.54	D10Ertd214e	NM_134007.2	Mus musculus DNA segment, Chr 10, ERATO Doi 214, expressed (D10Ertd214e), mRNA.
scl051800.6_308-S	-60.7516	-1.74	Bok	NM_016778	Mus musculus Bcl-2-related ovarian killer protein (Bok), mRNA.
scl017836.18_18-S	-59.0568	-1.87	Mug1	NM_008645.2	Mus musculus murinoglobulin 1 (Mug1), mRNA.
scl0277333.1_280-S	-53.49	-1.59	MGC68323	NM_199472.1	Mus musculus similar to glyceraldehyde-3-phosphate dehydrogenase (phosphorylating) (EC 1.2.1.12) - mouse (MGC68323), mRNA.
scl30665.8.1_14-S	-52.5647	-1.49	1110032O16Rik	XM_133813.4	Mus musculus RIKEN cDNA 1110032O16 gene (1110032O16Rik), mRNA.
scl20534.5_360-S	-47.3892	-1.4	Cd59a	NM_007652.2	Mus musculus CD59a antigen (Cd59a), mRNA.
scl42840.6.80_202-S	-41.3808	-1.41	Serpina4-ps1	XM_127122.2	
scl093739.4_24-S	-41.1992	-1.65	Gabarapl2	NM_026693.2	Mus musculus GABA(A) receptor-associated protein like 2 (Gabarapl2), mRNA.
scl060525.18_152-S	-38.7756	-1.41	Acas2	NM_019811.2	Mus musculus acetyl-Coenzyme A synthetase 2 (ADP forming) (Acas2), mRNA.
scl27606.14.1_65-S	-38.692	-1.33	Afm	NM_145146.1	Mus musculus afamin (Afm), mRNA.
scl068195.1_129-S	-38.5412	-1.38	Rnaset2	NM_026611.1	Mus musculus ribonuclease T2 (Rnaset2), mRNA.
scl42858.13_56-S	-38.4163	-1.75	Rin3	NM_177620.2	Mus musculus Ras and Rab interactor 3 (Rin3), mRNA.
scl23142.2.172_159-S	-36.0157	-2.08	P2ry1	NM_008772.2	Mus musculus purinergic receptor P2Y, G-protein coupled 1 (P2ry1), mRNA.
scl36156.3_7-S	-35.13	-1.79	Edg5	NM_010333	Mus musculus endothelial differentiation, sphingolipid G-protein-coupled receptor, 5 (Edg5), mRNA.
scl020317.1_68-S	-34.928	-1.3	Serpinf1	NM_011340.2	Mus musculus serine (or cysteine) proteinase inhibitor, clade F, member 1 (Serpinf1), mRNA.
scl0015040.1_90-S	-33.9706	-1.33	H2-T23	NM_010398.1	Mus musculus histocompatibility 2, T region locus 23 (H2-T23), mRNA.

scl0076654.1_225-S	-33.5381	-1.46	Upp2	NM_029692.1	Mus musculus uridine phosphorylase 2 (Upp2), mRNA.
scl51764.24.1_222-S	-33.2678	-1.6	4933427L07Rik	NM_027727.1	Mus musculus RIKEN cDNA 4933427L07 gene (4933427L07Rik), mRNA.
scl36510.4_431-S	-33.1002	-1.37	1810013B01Rik	NM_029631	Mus musculus RIKEN cDNA 1810013B01 gene (1810013B01Rik), mRNA.
scl0013809.2_54-S	-32.0077	-1.46	Enpep	NM_007934.1	Mus musculus glutamyl aminopeptidase (Enpep), mRNA.
scl38453.7.1_27-S	-31.8387	-1.35	Csrp2	NM_007792.2	Mus musculus cysteine and glycine-rich protein 2 (Csrp2), mRNA.
scl072043.2_38-S	-29.581	-1.48	Sulf2	XM_358343.1	
scl0231871.16_33-S	-28.0465	-1.46	E330036I19Rik	NM_144915.2	Mus musculus RIKEN cDNA E330036I19 gene (E330036I19Rik), mRNA.
scl0069354.1_68-S	-28.015	-1.37	Slc38a4	NM_027052.2	Mus musculus solute carrier family 38, member 4 (Slc38a4), mRNA.
scl31028.4.1_18-S	-27.8178	-2.09	Aqp11	NM_175105.2	Mus musculus aquaporin 11 (Aqp11), mRNA.
scl076654.7_194-S	-27.4625	-1.4	Upp2	NM_029692.1	Mus musculus uridine phosphorylase 2 (Upp2), mRNA.
scl15766.4.1_27-S	-27.0369	-1.44	1110060M21Rik	NM_025424.1	Mus musculus RIKEN cDNA 1110060M21 gene (1110060M21Rik), mRNA.
scl068028.3_20-S	-26.6605	-1.46	3110001N18Rik	NM_026517.1	Mus musculus RIKEN cDNA 3110001N18 gene (3110001N18Rik), mRNA.
scl00353208.1_64-S	-26.6454	-1.46	2810021G02Rik		
scl013885.9_29-S	-26.3108	-1.27	Esd	NM_016903	
scl50689.6_424-S	-26.0925	-1.36	Slc35b1	XM_128634.4	Mus musculus solute carrier family 35, member B1 (Slc35b1), mRNA.
scl24019.5_109-S	-26.0389	-1.25	Cpt2	NM_009949	Mus musculus carnitine palmitoyltransferase 2 (Cpt2), mRNA.
scl48561.1.13_8-S	-25.5364	-1.32	0610012G03Rik	NM_025320	Mus musculus RIKEN cDNA 0610012G03 gene (0610012G03Rik), mRNA.
scl0056473.2_148-S	-25.5211	-1.62	Fads2	NM_019699.1	Mus musculus fatty acid desaturase 2 (Fads2), mRNA.
scl24738.7.1_167-S	-25.3922	-1.59	Agmat	XM_131722.2	Mus musculus agmatine ureohydrolase (agmatinase) (Agmat), mRNA.
scl0012091.2_274-S	-25.3493	-1.41	Glb1	NM_009752.1	Mus musculus galactosidase, beta 1 (Glb1), mRNA.
scl0001121.1_1-S	-25.2118	-1.37	Ndufa5	NM_026614.1	Mus musculus NADH dehydrogenase (ubiquinone) 1 alpha subcomplex, 5 (Ndufa5), mRNA.
scl0019299.2_231-S	-24.9867	-1.42	Abcd3	NM_008991.1	Mus musculus ATP-binding cassette, sub-family D (ALD), member 3 (Abcd3), mRNA.
scl013010.2_9-S	-24.7589	-1.24	Cst3	NM_009976.2	Mus musculus cystatin C (Cst3), mRNA.
scl27904.14.1_131-S	-24.6539	-1.41	Hgfac	NM_019447.1	Mus musculus hepatocyte growth factor activator (Hgfac), mRNA.
scl0066881.2_144-S	-24.5497	-1.52	Pcyox1	NM_025823.3	Mus musculus prenylcysteine oxidase 1 (Pcyox1), mRNA.
scl00232449.2_167-S	-24.4302	-1.4	2500002K03Rik	NM_172733.1	Mus musculus RIKEN cDNA 2500002K03 gene (2500002K03Rik), mRNA.
scl012615.5_26-S	-24.4192	-1.83	Cenpa	NM_007681.1	Mus musculus centromere autoantigen A (Cenpa), mRNA.
scl011958.2_29-S	-24.2201	-1.67	Atp5k	NM_007507	Mus musculus ATP synthase, H+ transporting, mitochondrial F1F0 complex, subunit e (Atp5k), mRNA.
scl016005.2_201-S	-24.1536	-1.43	Igfals	NM_008340.2	Mus musculus insulin-like growth factor binding protein, acid labile subunit (Igfals), mRNA.
scl0065111.1_63-S	-24.0089	-1.25	Dap3	NM_022994.2	Mus musculus death associated protein 3 (Dap3), mRNA.
scl0071755.2_316-S	-23.6636	-1.39	1300018L09Rik	NM_027903.1	
scl0245867.4_1-S	-23.1996	-1.57	5330414D10Rik	NM_153594.2	Mus musculus RIKEN cDNA 5330414D10 gene (5330414D10Rik), mRNA.
scl47189.8.1_128-S	-23.0352	-1.26	Mrpl13	NM_026759.2	Mus musculus mitochondrial ribosomal protein L13 (Mrpl13), mRNA.
scl069786.2_30-S	-23.0343	-1.9	1810034M08Rik	NM_176842.2	Mus musculus RIKEN cDNA 1810034M08 gene (1810034M08Rik), mRNA.
scl030931.1_245-S	-22.9104	-1.29	Dyt1	NM_144884.1	Mus musculus dystonia 1 (Dyt1), mRNA.
scl0015587.1_282-S	-22.7617	-1.3	Hyal2	NM_010489.2	Mus musculus hyaluronidase 2 (Hyal2), mRNA.
scl27967.7.1_149-S	-22.7011	-1.25	Abhd1	NM_021304.2	Mus musculus abhydrolase domain containing 1 (Abhd1), mRNA.
scl0017993.1_168-S	-22.4263	-1.23	Ndufs4	NM_010887.1	Mus musculus NADH dehydrogenase (ubiquinone) Fe-S protein 4 (Ndufs4), mRNA.
scl015024.2_236-S	-22.3217	-1.5	H2-T10	NM_010395.2	Mus musculus histocompatibility 2, T region locus 10 (H2-T10), mRNA.
scl071435.1_313-S	-22.1672	-1.29	Arhgap21	XM_130033.5	
scl28963.13.1_27-S	-22.1273	-1.38	Nt5c3	NM_026004.1	Mus musculus 5-nucleotidase, cytosolic III (Nt5c3), mRNA.
scl46659.10_50-S	-22.0509	-1.27	Zfp385	NM_013866.1	Mus musculus zinc finger protein 385 (Zfp385), mRNA.
scl056043.1_7-S	-22.0415	-1.52	Akr1e1	NM_018859.1	Mus musculus aldo-keto reductase family 1, member E1 (Akr1e1), mRNA.
scl35702.23.1_49-S	-21.9961	-1.66	AV340375	NM_172519.1	Mus musculus expressed sequence AV340375 (AV340375), mRNA.
scl017449.1_1-S	-21.9579	-1.49	Mdh1	NM_008618.2	Mus musculus malate dehydrogenase 1, NAD (soluble) (Mdh1), mRNA.
scl23150.4.1_11-S	-21.9494	-1.59	Aadac	NM_023383.1	Mus musculus arylacetamide deacetylase (esterase) (Aadac), mRNA.
scl53954.7_10-S	-21.8459	-1.54	Slc16a2	NM_009197.1	Mus musculus solute carrier family 16 (monocarboxylic acid transporters), member 2 (Slc16a2), mRNA.
scl52495.17.1_252-S	-21.8017	-9.16	Blnk	NM_008528.3	Mus musculus B-cell linker (Blnk), mRNA.
scl023986.1_240-S	-21.7845	-1.6	Peci	NM_011868.1	Mus musculus peroxisomal delta3, delta2-enoyl-Coenzyme A isomerase (Peci), mRNA.
scl28480.7_231-S	-21.7816	-1.23	Adipor2	NM_197985.2	
gi_30794511_ref_NM_01	-21.7606	-1.41	Hmbs	NM_013551.1	Mus musculus hydroxymethylbilane synthase (Hmbs), mRNA.
scl29306.21.1_2-S	-21.7278	-1.24	Slc25a13	NM_015829.1	Mus musculus solute carrier family 25 (mitochondrial carrier; adenine nucleotide translocator), member 13 (Slc25a13), mRNA.
scl0016923.1_194-S	-21.5186	-1.43	Lnk	NM_008507.2	Mus musculus linker of T-cell receptor pathways (Lnk), mRNA.
scl31053.2.1_38-S	-21.4446	-1.3	1810020E01Rik	NM_025460.1	Mus musculus RIKEN cDNA 1810020E01 gene (1810020E01Rik), mRNA.
scl0015129.1_300-S	-21.4001	-2.21	Hbb-b1	NM_008220.2	Mus musculus hemoglobin, beta adult major chain (Hbb-b1), mRNA.
scl44903.11.3_96-S	-21.2726	-1.42	Fars1	NM_024274.1	Mus musculus phenylalanine-tRNA synthetase 1 (mitochondrial) (Fars1), mRNA.

scl22246.19.1_4-S	-21.26	-1.33	Mccc1	NM_023644.2	Mus musculus methylcrotonoyl-Coenzyme A carboxylase 1 (alpha) (Mccc1), mRNA.
scl21666.4.43_5-S	-21.1897	-2.7	Gstm6	NM_008184.1	Mus musculus glutathione S-transferase, mu 6 (Gstm6), mRNA.
scl0014733.2_286-S	-21.12	-2.19	Gpc1	NM_016696.1	Mus musculus glypican 1 (Gpc1), mRNA.
scl16633.10.1_180-S	-20.9497	-1.36	Pecr	NM_023523.3	Mus musculus peroxisomal trans-2-enoyl-CoA reductase (Pecr), mRNA.
scl29045.3.1_38-S	-20.8041	-1.34	Rarres2	NM_027852.1	Mus musculus retinoic acid receptor responder (tazarotene induced) 2 (Rarres2), mRNA.
scl29983.21.1_98-S	-20.7876	-2.43	Abcg2	NM_011920.1	Mus musculus ATP-binding cassette, sub-family G (WHITE), member 2 (Abcg2), mRNA.
scl019122.2_199-S	-20.6556	-1.43	Prnp	NM_011170.1	Mus musculus prion protein (Prnp), mRNA.
scl24777.7.1_203-S	-20.5607	-1.35	Akr7a5	NM_025337.2	Mus musculus aldo-keto reductase family 7, member A5 (aflatoxin aldehyde reductase) (Akr7a5), mRNA.
scl35398.12.2_77-S	-20.5418	-1.36	Alas1	NM_020559.1	Mus musculus aminolevulinic acid synthase 1 (Alas1), mRNA.
scl0011883.2_180-S	-20.4325	-1.66	Arsa	NM_009713.1	Mus musculus arylsulfatase A (Arsa), mRNA.
scl065111.1_137-S	-20.3498	-1.24	Dap3	NM_022994.2	Mus musculus death associated protein 3 (Dap3), mRNA.
scl000022.1_12-S	-20.021	-1.28	Cpt2	NM_009949	Mus musculus carnitine palmitoyltransferase 2 (Cpt2), mRNA.

TABLE A8

GENMAPPS AND GENE ONTOLOGIES SIGNIFICANTLY CHANGED BY OPP IN LIVERS

UP-REGULATED - GENMAPP

No.	MAPP Name	Number Changed	Number Measured	Number on MAPP	Percent Changed	Percent Present	Z Score	Permuted P Value
1	Mm_IL-9_NetPath_20	2	23	24	8.6957	95.8333	6.8320	0.0000

UP-REGULATED - GENE ONTOLOGY

No.	GO Name	GO Type	Number Changed	Number Measured	Number in GO	Percent Changed	Percent Present	Z Score	Permuted P Value
1	response to unfolded protein	P	3	46	55	6.5217	83.6364	9.2090	0.0000
2	endoplasmic reticulum	C	8	529	561	1.5123	94.2959	6.5250	0.0000
3	cytoplasm	C	19	3488	3969	0.5447	87.8811	4.7660	0.0000
4	microsome	C	3	138	148	2.1739	93.2432	4.9670	0.0020
5	intracellular	C	23	6945	8118	0.3312	85.5506	2.8170	0.0050
6	metabolism	P	22	6721	7938	0.3273	84.6687	2.6410	0.0060

DOWN-REGULATED - GENMAPP

No.	MAPP Name	Number Changed	Number Measured	Number on MAPP	Percent Changed	Percent Present	Z Score	Permuted P Value
1	Mm_Reductive_carboxylate_cycle_CO2_fixation_	2	5	15	40.0000	33.3333	9.7920	0.0000
2	Mm_Heme_Biosynthesis	2	9	9	22.2222	100.0000	7.1810	0.0010
3	Mm_Galactose_metabolism	2	19	46	10.5263	41.3044	4.7410	0.0030
4	Mm_Mitochondrial_fatty_acid_betaoxidation	2	16	16	12.5000	100.0000	5.2320	0.0040

DOWN-REGULATED - GENE ONTOLOGY

No.	GO Name	GO Type	Number Changed	Number Measured	Number in GO	Percent Changed	Percent Present	Z Score	Permuted P Value
1	mitochondrion	C	15	730	822	2.0548	88.8078	5.8980	0.0000
2	cytoplasm	C	33	3488	3969	0.9461	87.8811	3.9820	0.0000
3	oxidoreductase activity	F	10	663	775	1.5083	85.5484	3.6090	0.0000
4	catalytic activity	F	39	4696	5420	0.8305	86.6421	3.5580	0.0000
5	arylsulfatase activity	F	2	6	7	33.3333	85.7143	11.1590	0.0010
6	heme biosynthesis	P	2	11	11	18.1818	100.0000	8.1340	0.0010
7	antigen processing\, endogenous antigen via MHC class I	P	2	20	23	10.0000	86.9565	5.8880	0.0010
8	mitochondrial inner membrane	C	6	232	275	2.5862	84.3636	4.4000	0.0010
9	MHC class I protein complex	C	2	25	32	8.0000	78.1250	5.1950	0.0020
10	MHC class I receptor activity	F	2	28	35	7.1429	80.0000	4.8680	0.0020
11	sulfuric ester hydrolase activity	F	2	11	12	18.1818	91.6667	8.1340	0.0030
12	oxygen transporter activity	F	2	17	21	11.7647	80.9524	6.4390	0.0030
13	lysosome	C	4	123	129	3.2520	95.3488	4.2210	0.0030
14	oxygen transport	P	2	19	25	10.5263	76.0000	6.0580	0.0040
15	antigen presentation\, endogenous antigen	P	2	22	25	9.0909	88.0000	5.5840	0.0040
16	NADH dehydrogenase (ubiquinone) activity	F	2	27	39	7.4074	69.2308	4.9710	0.0070
17	carbohydrate metabolism	P	7	442	506	1.5837	87.3518	3.1470	0.0070

TABLE A9					
GENES UP-REGULATED BY OPP IN SPLEENS					
TargetID	Diff_Score	Fold_Change	Symbol	Accession	Definition
scl38701.12.1_200-S	177.7388	2.66	Arid3a	NM_007880.1	Mus musculus AT rich interactive domain 3A (Bright like) (Arid3a), mRNA.
scl35398.12.2_77-S	161.9746	2.75	Alas1	NM_020559.1	Mus musculus aminolevulinic acid synthase 1 (Alas1), mRNA.
scl21972.20_222-S	160.1009	3	Sema4a	NM_013658.2	Mus musculus sema domain, immunoglobulin domain (Ig), transmembrane domain (TM) and short cytoplasmic domain, (semaphorin) 4A (Sema4a), mRNA.
scl0270198.14_257-S	150.8033	2.82	C230090D14	NM_173019.2	Mus musculus hypothetical protein C230090D14 (C230090D14), mRNA.
scl49773.2_28-S	148.7503	4.16	Lrg1	NM_029796.2	Mus musculus leucine-rich alpha-2-glycoprotein 1 (Lrg1), mRNA.
scl021881.14_30-S	143.4326	1.85	Tkt	NM_009388.2	Mus musculus transketolase (Tkt), mRNA.
scl012655.1_87-S	138.0005	5	Chi3l3	NM_009892	Mus musculus chitinase 3-like 3 (Chi3l3), mRNA.
scl30503.2.1_167-S	133.1051	4.44	Ifitm6	XM_133956.3	
scl0110208.1_282-S	132.4158	2.82	Pgd	NM_025801.1	Mus musculus phosphogluconate dehydrogenase (Pgd), mRNA.
scl36760.13.1_118-S	132.1993	2.03	Anxa2	NM_007585.2	Mus musculus annexin A2 (Anxa2), mRNA.
scl022793.9_329-S	130.0814	1.8	Zyx	NM_011777.1	Mus musculus zyxin (Zyx), mRNA.
scl0014751.2_296-S	129.6172	2.38	Gpi1	NM_008155.1	Mus musculus glucose phosphate isomerase 1 (Gpi1), mRNA.
scl33920.14_231-S	129.181	2.49	Gsr	NM_010344.3	Mus musculus glutathione reductase 1 (Gsr), mRNA.
scl45515.5.1_34-S	123.3775	4.55	Ctsg	NM_007800.1	Mus musculus cathepsin G (Ctsg), mRNA.
scl50898.5.1_60-S	123.2693	4.75	Pi16	NM_023734.2	
scl22942.3.1_64-S	122.1666	2.74	S100a6	NM_011313.1	Mus musculus S100 calcium binding protein A6 (calcyclin) (S100a6), mRNA.
scl0019126.2_253-S	122.0294	5.3	Prom1	NM_008935.1	Mus musculus prominin 1 (Prom1), mRNA.
scl28438.14_163-S	121.5518	2.39	Slc2a3	NM_011401.2	Mus musculus solute carrier family 2 (facilitated glucose transporter), member 3 (Slc2a3), mRNA.
scl018824.1_278-S	120.214	2.12	Plp2	NM_019755.2	Mus musculus proteolipid protein 2 (Plp2), mRNA.
scl012608.3_48-S	119.9969	2.36	Cebpb	NM_009883.1	Mus musculus CCAAT/enhancer binding protein (C/EBP), beta (Cebpb), mRNA.
scl34345.7_19-S	115.677	4.99	Hp	NM_017370.1	Mus musculus haptoglobin (Hp), mRNA.
scl36717.10_331-S	115.5086	2.97	Rab27a	NM_023635.2	Mus musculus RAB27A, member RAS oncogene family (Rab27a), mRNA.
scl41074.16.1_14-S	113.2969	3.54	Mpo	NM_010824	Mus musculus myeloperoxidase (Mpo), mRNA.
scl33924.6.9_11-S	109.588	1.9	Ppp2cb	NM_017374.2	Mus musculus protein phosphatase 2a, catalytic subunit, beta isoform (Ppp2cb), mRNA.
scl0245195.2_0-S	105.9338	4.93	Retnlg	NM_181596.2	Mus musculus resistin like gamma (Retnlg), mRNA.
scl00235534.1_198-S	104.1644	2.81	C130099A20Rik	NM_153420.1	
scl0016796.1_242-S	103.3243	2.05	Lasp1	NM_010688.2	Mus musculus LIM and SH3 protein 1 (Lasp1), mRNA.
scl20002.16.7_10-S	102.794	4.56	Lbp	NM_008489	Mus musculus lipopolysaccharide binding protein (Lbp), mRNA.
scl25450.6_639-S	101.9543	3.78	5730528L13Rik	NM_028137.1	Mus musculus RIKEN cDNA 5730528L13 gene (5730528L13Rik), mRNA.
scl31130.14_110-S	101.9276	2.49	Fes	NM_010194.1	Mus musculus feline sarcoma oncogene (Fes), mRNA.
scl21429.6_290-S	99.8299	1.71	Lmo4	NM_010723.2	Mus musculus LIM domain only 4 (Lmo4), mRNA.
scl36920.15.1_107-S	96.1643	2	Pstpip1	NM_011193.1	Mus musculus proline-serine-threonine phosphatase-interacting protein 1 (Pstpip1), mRNA.
scl47772.10.1_9-S	94.7984	2.81	Ncf4	NM_008677.1	Mus musculus neutrophil cytosolic factor 4 (Ncf4), mRNA.
scl016952.2_2-S	94.4381	3.41	Anxa1	NM_010730.1	Mus musculus annexin A1 (Anxa1), mRNA.
scl49691.9_66-S	92.3655	1.91	1700093E07Rik	NM_133685.1	Mus musculus RIKEN cDNA 1700093E07 gene (1700093E07Rik), mRNA.
scl32869.6.1_0-S	89.7918	1.84	Gmfg	NM_022024.1	Mus musculus glia maturation factor, gamma (Gmfg), mRNA.
scl27561.16.206_36-S	89.5431	3.46	Anxa3	NM_013470.1	Mus musculus annexin A3 (Anxa3), mRNA.
scl050701.4_101-S	88.8819	4.07	NE	NM_015779	Mus musculus neutrophil elastase (NE), mRNA.
scl0074349.2_256-S	88.0091	2.48	4632419K20Rik	NM_199009.1	Mus musculus RIKEN cDNA 4632419K20 gene (4632419K20Rik), mRNA.
scl067689.9_195-S	87.8247	2.45	Aldh3b1	NM_026316.2	Mus musculus aldehyde dehydrogenase 3 family, member B1 (Aldh3b1), mRNA.
scl0003853.1_11-S	87.643	2.51	Lta4h	NM_008517.1	Mus musculus leukotriene A4 hydrolase (Lta4h), mRNA.
scl50639.3.1_165-S	86.9091	6.17	Trem3	NM_021407.1	Mus musculus triggering receptor expressed on myeloid cells 3 (Trem3), mRNA.
scl066375.2_40-S	86.6477	2.17	Dhrs7	NM_025522.1	Mus musculus dehydrogenase/reductase (SDR family) member 7 (Dhrs7), mRNA.
scl51494.33_412-S	84.2179	2.05	E030006K04Rik	NM_139206.1	Mus musculus RIKEN cDNA E030006K04 gene (E030006K04Rik), mRNA.
scl41137.3_142-S	84.0799	5.38	1700020L24Rik	NM_025492.1	Mus musculus RIKEN cDNA 1700020L24 gene (1700020L24Rik), mRNA.
scl34127.7.1_0-S	83.1342	4.52	1810033B17Rik	XM_110690.2	Mus musculus RIKEN cDNA 1810033B17 gene (1810033B17Rik), mRNA.
scl017970.15_30-S	82.707	1.98	Ncf2	NM_010877	Mus musculus neutrophil cytosolic factor 2 (Ncf2), mRNA.
scl34224.8.1_50-S	80.4056	1.56	Cyba	NM_007806.1	Mus musculus cytochrome b-245, alpha polypeptide (Cyba), mRNA.
scl068713.2_9-S	79.4977	1.56	Ifitm1	NM_026820	Mus musculus interferon induced transmembrane protein 1 (Ifitm1), mRNA.
scl27078.4.1_81-S	78.783	2.04	0910001L09Rik	XM_132434.2	Mus musculus RIKEN cDNA 0910001L09 gene (0910001L09Rik), mRNA.
scl0320078.9_68-S	78.6236	3.58	Olfml2b	NM_177068.3	
scl0026367.1_126-S	78.1041	2.33	Ceacam2	NM_007543.2	Mus musculus CEA-related cell adhesion molecule 2 (Ceacam2), mRNA.
scl42729.13.1_74-S	77.728	2.32	Adssl1	NM_007421.1	

scl20865.8_33-S	76.7349	2.83	Gca	NM_145523.2	Mus musculus grancalcin (Gca), mRNA.
scl014252.10_327-S	75.7065	2.25	Flot2	NM_008028.1	Mus musculus flotillin 2 (Flot2), mRNA.
scl37304.10_29-S	75.1493	5.39	Mmp8	NM_008611.2	Mus musculus matrix metalloproteinase 8 (Mmp8), mRNA.
scl33021.4.288_87-S	74.8791	4.41	Pglyrp1	NM_009402.1	Mus musculus peptidoglycan recognition protein 1 (Pglyrp1), mRNA.
scl41339.16.1_19-S	73.9063	2.46	Arrb2	NM_145429.1	Mus musculus arrestin, beta 2 (Arrb2), mRNA.
gi_6679936_ref_NM_008(	73.7644	1.56			
scl014289.2_177-S	72.9112	3.22	Fpr-rs2	NM_008039.1	Mus musculus formyl peptide receptor, related sequence 2 (Fpr-rs2), mRNA.
scl46402.6.1_29-S	72.2488	1.68	Lgals3	NM_010705.1	Mus musculus lectin, galactose binding, soluble 3 (Lgals3), mRNA.
GI_6679936-S	72.1757	1.8	Gapd	NM_008084.1	Mus musculus glyceraldehyde-3-phosphate dehydrogenase (Gapd), mRNA.
scl52720.7.1_78-S	71.2929	3.96	Ms4a3	NM_133246.2	Mus musculus membrane-spanning 4-domains, subfamily A, member 3 (Ms4a3), mRNA.
scl014433.2_76-S	70.4827	1.86	Gapd	NM_008084	Mus musculus glyceraldehyde-3-phosphate dehydrogenase (Gapd), mRNA.
scl074145.1_2-S	70.3988	3.29	F13a1	NM_028784	
scl23525.7_662-S	70.0965	1.82	Agtrap	NM_009642.3	Mus musculus angiotensin II, type I receptor-associated protein (Agtrap), mRNA.
scl0018230.2_224-S	69.9751	1.86	Nxn	NM_008750.2	Mus musculus nucleoredoxin (Nxn), mRNA.
scl46215.1.1_293-S	68.6139	3.4	Arl11	NM_177337.3	
scl0053857.2_245-S	67.3285	4.17	Tuba8	NM_017379.1	Mus musculus tubulin, alpha 8 (Tuba8), mRNA.
scl35378.12_17-S	67.1574	3.04	Mapkapk3	NM_178907	
scl013806.12_49-S	66.5394	2.11	Eno1	NM_023119	Mus musculus enolase 1, alpha non-neuron (Eno1), mRNA.
scl43966.3_250-S	66.4341	2.65	Nfil3	NM_017373.2	Mus musculus nuclear factor, interleukin 3, regulated (Nfil3), mRNA.
scl23305.6_263-S	66.2011	3.21	Gpr160	XM_130823.3	
scl0002552.1_594-S	65.9619	1.52	Triobp	NM_138579.2	Mus musculus TRIO and F-actin binding protein (Triobp), mRNA.
scl35271.8.1_43-S	65.8349	2.91	Cklfsf7	NM_133978.1	Mus musculus chemokine-like factor super family 7 (Cklfsf7), mRNA.
scl19917.13_586-S	65.56	4.3	Mmp9	NM_013599.2	Mus musculus matrix metalloproteinase 9 (Mmp9), mRNA.
scl35154.1.15_20-S	65.5458	2.97	2400009B08Rik	XM_358687.1	Mus musculus RIKEN cDNA 2400009B08 gene (2400009B08Rik), mRNA.
scl011821.1_236-S	64.4542	2.14	Aprt	NM_009698.1	Mus musculus adenine phosphoribosyl transferase (Aprt), mRNA.
scl27109.17.1_35-S	64.0687	1.57	Plod3	NM_011962.2	Mus musculus procollagen-lysine, 2-oxoglutarate 5-dioxygenase 3 (Plod3), mRNA.
scl39250.15_28-S	63.3882	2.17	Aatk	NM_007377.1	Mus musculus apoptosis-associated tyrosine kinase (Aatk), mRNA.
scl067905.1_18-S	63.2065	1.54	2810423O19Rik	NM_026447	Mus musculus RIKEN cDNA 2810423O19 gene (2810423O19Rik), transcript variant 1, mRNA.
scl42194.2_0-S	62.8876	4.54	Dio2	NM_010050	Mus musculus deiodinase, iodothyronine, type II (Dio2), mRNA.
scl28812.19_190-S	62.355	2.11	Hk2	NM_013820.1	Mus musculus hexokinase 2 (Hk2), mRNA.
scl34064.10_3-S	61.627	2.63	F10	NM_007972.2	Mus musculus coagulation factor X (F10), mRNA.
scl0020661.1_145-S	61.3907	1.89	Sort1	NM_019972	Mus musculus sortilin 1 (Sort1), mRNA.
scl43488.6_364-S	60.9435	1.61	Snag1	NM_130796.1	Mus musculus sorting nexin associated golgi protein 1 (Snag1), mRNA.
scl0017523.2_246-S	60.7962	3.47	Mpo	NM_010824.1	Mus musculus myeloperoxidase (Mpo), mRNA.
scl018655.12_25-S	60.5693	1.83	Pgk1	NM_008828	Mus musculus phosphoglycerate kinase 1 (Pgk1), mRNA.
scl50849.26.1_265-S	60.3933	2.04	Myo1f	NM_053214.1	Mus musculus myosin IF (Myo1f), mRNA.
scl52308.5.1_95-S	59.4846	2.17	Csf2ra	NM_009970.1	Mus musculus colony stimulating factor 2 receptor, alpha, low-affinity (granulocyte-macrophage) (Csf2ra), mRNA.
scl23461.27_162-S	59.4831	1.64	Kcnab2	NM_010598.2	Mus musculus potassium voltage-gated channel, shaker-related subfamily, beta member 2 (Kcnab2), mRNA.
scl32703.15.1_0-S	58.8422	2.25	Pnkp	NM_021549.1	Mus musculus polynucleotide kinase 3- phosphatase (Pnkp), mRNA.
scl0023845.2_228-S	58.6269	2.29	Clecsf5	NM_021364.1	Mus musculus C-type (calcium dependent, carbohydrate-recognition domain) lectin, superfamily member 5 (Clecsf5), mRNA.
scl0207777.9_233-S	58.5508	4.03	Bzrap1	NM_172449.1	Mus musculus benzodiazapine receptor associated protein 1 (Bzrap1), mRNA.
scl0022591.2_289-S	58.3405	2.85	Xpc	NM_009531.1	Mus musculus xeroderma pigmentosum, complementation group C (Xpc), mRNA.
scl46266.2_361-S	58.1673	4.18	Ltb4r1	NM_008519.1	Mus musculus leukotriene B4 receptor 1 (Ltb4r1), mRNA.
scl011777.6_49-S	58.0383	2.02	Ap3s1	NM_009681.2	Mus musculus adaptor-related protein complex 3, sigma 1 subunit (Ap3s1), mRNA.
scl011674.2_46-S	57.3537	2.06	Aldoa	NM_007438.2	
scl21336.8_145-S	56.8663	1.52	3110001A13Rik	NM_025626.3	Mus musculus RIKEN cDNA 3110001A13 gene (3110001A13Rik), mRNA.
scl018405.4_2-S	55.7594	4.37	Orm1	NM_008768.1	Mus musculus orosomucoid 1 (Orm1), mRNA.
scl34132.19.1_1-S	54.5609	1.5	Stxbp2	NM_011503.2	Mus musculus syntaxin binding protein 2 (Stxbp2), mRNA.
scl22689.16.1_37-S	54.5119	1.68	Sdfr2	NM_009146	Mus musculus stromal cell derived factor receptor 2 (Sdfr2), mRNA.
scl45684.12_151-S	54.4907	1.52	D14Ertd226e	NM_145928	Mus musculus DNA segment, Chr 14, ERATO Doi 226, expressed (D14Ertd226e), mRNA.
scl16317.12_198-S	53.8349	1.96	Mapkapk2	NM_008551.1	Mus musculus MAP kinase-activated protein kinase 2 (Mapkapk2), mRNA.
scl0004022.1_70-S	53.7937	1.69	Arpc1b	NM_023142.1	Mus musculus actin related protein 2/3 complex, subunit 1B (Arpc1b), mRNA.
scl30727.6.1_169-S	53.2772	2.13	Igsf6	NM_030691.1	Mus musculus immunoglobulin superfamily, member 6 (Igsf6), mRNA.
scl0378460.6_10-S	52.8484	8.23	4632424B03Rik	XM_128537.3	Mus musculus RIKEN cDNA 4632424B03 gene (4632424B03Rik), mRNA.
scl012986.17_26-S	52.6157	2.73	Csf3r	NM_007782.1	Mus musculus colony stimulating factor 3 receptor (granulocyte) (Csf3r), mRNA.
scl30153.32_359-S	52.6145	19.4	Mgam	XM_133071.2	

scl18037.10.1_70-S	52.6026	18.68	Il1r2	NM_010555.2	Mus musculus interleukin 1 receptor, type II (Il1r2), mRNA.
scl38707.5.30_13-S	52.1451	4.09	Prtn3	NM_011178.2	Mus musculus proteinase 3 (Prtn3), mRNA.
scl25159.19.1_9-S	51.8008	1.85	2810475A17Rik	NM_028355.1	Mus musculus RIKEN cDNA 2810475A17 gene (2810475A17Rik), mRNA.
scl50768.5_1-S	51.7844	1.44	2310014H01Rik	XM_355011.1	Mus musculus RIKEN cDNA 2310014H01 gene (2310014H01Rik), mRNA.
scl16390.7_3-S	51.5634	1.7	Ralb	NM_022327.3	Mus musculus v-ral simian leukemia viral oncogene homolog B (ras related) (Ralb), mRNA.
scl32949.5.149_25-S	51.4288	3.82	Ceacam10	NM_007675.2	Mus musculus CEA-related cell adhesion molecule 10 (Ceacam10), mRNA.
scl33481.17.1_142-S	51.1403	6.47	Cpne2	NM_153507.2	Mus musculus copine II (Cpne2), mRNA.
scl0112407.1_41-S	50.8773	4.63	Egln3	NM_028133.1	Mus musculus EGL nine homolog 3 (C. elegans) (Egln3), mRNA.
scl018746.1_82-S	50.7045	2.61	Pkm2	NM_011099.2	Mus musculus pyruvate kinase, muscle (Pkm2), mRNA.
scl50763.13.1_199-S	50.4259	1.7	Flot1	NM_008027.1	Mus musculus flotillin 1 (Flot1), mRNA.
scl34795.4.1_9-S	50.3475	1.67	Sap30	NM_021788.1	Mus musculus sin3 associated polypeptide (Sap30), mRNA.
scl49245.4_458-S	50.3267	5.2	1500031L02Rik	NM_025892.1	Mus musculus RIKEN cDNA 1500031L02 gene (1500031L02Rik), mRNA.
scl054672.8_79-S	49.6359	2.87	Gpr97	NM_173036.2	Mus musculus G protein-coupled receptor 97 (Gpr97), mRNA.
scl30062.4.1_1-S	49.5887	1.96	Npy	NM_023456.2	Mus musculus neuropeptide Y (Npy), mRNA.
scl33753.14_134-S	49.2227	1.44	Atp6v1b2	NM_007509.2	Mus musculus ATPase, H+ transporting, V1 subunit B, isoform 2 (Atp6v1b2), mRNA.
scl00230709.1_4-S	48.6561	1.8	Zmpste24	NM_172700.1	Mus musculus zinc metalloproteinase, STE24 homolog (S. cerevisiae) (Zmpste24), mRNA.
scl26700.8.1_2-S	48.4696	1.62	0610009O03Rik	NM_026660.2	Mus musculus RIKEN cDNA 0610009O03 gene (0610009O03Rik), mRNA.
scl0012874.1_259-S	47.8375	2.1	Cpd	NM_007754.1	Mus musculus carboxypeptidase D (Cpd), mRNA.
scl42371.18.1_30-S	47.7175	4.15	Pygl	NM_133198.1	Mus musculus liver glycogen phosphorylase (Pygl), mRNA.
scl36309.11_147-S	47.536	1.8	Abhd5	NM_026179.1	Mus musculus abhydrolase domain containing 5 (Abhd5), mRNA.
scl0014733.2_286-S	47.3074	2.17	Gpc1	NM_016696.1	Mus musculus glypican 1 (Gpc1), mRNA.
scl0067742.1_215-S	47.2492	1.59	Samsn1	NM_023380.1	Mus musculus SAM domain, SH3 domain and nuclear localisation signals, 1 (Samsn1), mRNA.
scl011867.9_22-S	47.0802	1.65	Arpc1b	NM_023142.1	Mus musculus actin related protein 2/3 complex, subunit 1B (Arpc1b), mRNA.
scl44121.8_285-S	47.0159	3.07	Serpinb1a	NM_025429.1	Mus musculus serine (or cysteine) proteinase inhibitor, clade B, member 1a (Serpinb1a), mRNA.
scl36385.5_13-S	46.4355	1.54	Cklfsf6	NM_026036.1	Mus musculus chemokine-like factor super family 6 (Cklfsf6), mRNA.
scl45160.32_9-S	46.1822	2.09	Abcc4	XM_139262.2	Mus musculus ATP-binding cassette, sub-family C (CFTR/MRP), member 4 (Abcc4), mRNA.
scl0072141.1_1-S	46.0449	3.02	Adpgk	NM_028121.1	
scl37440.14_650-S	45.8546	1.68	Irak3	NM_028679.2	Mus musculus interleukin-1 receptor-associated kinase 3 (Irak3), mRNA.
scl0020861.2_171-S	45.7838	6.09	Stfa1	NM_001001332	Mus musculus stefin A1 (Stfa1), mRNA.
scl46955.19_187-S	45.7413	2.18	B230369L08Rik	NM_194342	
scl48225.31_410-S	44.755	1.74	Tiam1	NM_009384.1	Mus musculus T-cell lymphoma invasion and metastasis 1 (Tiam1), mRNA.
scl43914.11.4_21-S	44.668	1.57	H2afy	NM_012015.1	Mus musculus H2A histone family, member Y (H2afy), mRNA.
scl34521.8.1_20-S	44.5067	1.76	BC004022	XM_134557.1	Mus musculus cDNA sequence BC004022 (BC004022), mRNA.
scl25240.12_194-S	44.3524	1.55	Pgm2	NM_028132	Mus musculus phosphoglucomutase 2 (Pgm2), mRNA.
scl28328.8.1_45-S	43.9772	9.87	Klra17	NM_133203	Mus musculus killer cell lectin-like receptor, subfamily A, member 17 (Klra17), mRNA.
scl0014191.2_145-S	43.4806	1.64	Fgr	NM_010208.2	Mus musculus Gardner-Rasheed feline sarcoma viral (Fgr) oncogene homolog (Fgr), mRNA.
scl054722.1_159-S	43.2271	8.63	Dfna5h	XM_147318.1	Mus musculus deafness, autosomal dominant 5 homolog (human) (Dfna5h), mRNA.
scl44868.8_25-S	43.1492	1.95	Gcnt2	NM_008105.2	Mus musculus glucosaminyl (N-acetyl) transferase 2, I-branching enzyme (Gcnt2), transcript variant 3, mRNA.
scl069583.1_127-S	42.9846	1.67	Tnfsf13	NM_023517.1	Mus musculus tumor necrosis factor (ligand) superfamily, member 13 (Tnfsf13), mRNA.
scl53023.9_185-S	42.6139	6.68	4930538D17Rik	NM_029186.1	Mus musculus RIKEN cDNA 4930538D17 gene (4930538D17Rik), mRNA.
scl50873.6.1_76-S	42.4855	1.64	1500032D16Rik	NM_030087.1	Mus musculus RIKEN cDNA 1500032D16 gene (1500032D16Rik), mRNA.
scl013723.9_229-S	42.247	1.98	Emb	NM_010330.2	Mus musculus embigin (Emb), mRNA.
scl53436.18.1_9-S	41.4501	1.57	Tcirg1	NM_016921.2	Mus musculus T-cell, immune regulator 1 (Tcirg1), mRNA.
scl17197.4_15-S	41.3415	1.74	Tagln2	NM_178598.1	Mus musculus transgelin 2 (Tagln2), mRNA.
scl016828.6_330-S	41.3184	1.5	Ldh1	NM_010699.1	Mus musculus lactate dehydrogenase 1, A chain (Ldh1), mRNA.
scl49589.13.8_8-S	41.1247	1.61	2810036L13Rik	NM_144802.2	Mus musculus RIKEN cDNA 2810036L13 gene (2810036L13Rik), mRNA.
scl0083921.1_123-S	41.1206	2.15	Tmem2	NM_031997.2	Mus musculus transmembrane protein 2 (Tmem2), mRNA.
scl54435.12.1_121-S	40.9588	1.43	Was	NM_009515.1	Mus musculus Wiskott-Aldrich syndrome homolog (human) (Was), mRNA.
scl23214.8.1_66-S	40.6213	3.08	Mgst2	NM_174995.1	Mus musculus microsomal glutathione S-transferase 2 (Mgst2), mRNA.
scl054610.1_1-S	40.6127	1.6	Tbc1d8	NM_018775.1	Mus musculus TBC1 domain family, member 8 (Tbc1d8), mRNA.
scl011964.1_274-S	40.6024	1.55	Atp6v1a1	NM_007508.2	Mus musculus ATPase, H+ transporting, V1 subunit A, isoform 1 (Atp6v1a1), mRNA.
scl012765.6_0-S	40.2226	2.05	Il8rb	NM_009909.2	Mus musculus interleukin 8 receptor, beta (Il8rb), mRNA.
scl00100017.2_199-S	40.1967	1.55	AA691260	NM_145554.1	Mus musculus expressed sequence AA691260 (AA691260), mRNA.
scl16186.5.1_30-S	39.971	1.62	Rgs18	NM_022881.2	Mus musculus regulator of G-protein signaling 18 (Rgs18), mRNA.
scl40534.12.1_29-S	39.9435	1.54	Tbrg4	NM_134011.1	Mus musculus transforming growth factor beta regulated gene 4 (Tbrg4), mRNA.
scl0277333.1_280-S	39.8186	1.67	MGC68323	NM_199472.1	Mus musculus similar to glyceraldehyde-3-phosphate dehydrogenase (phosphorylating) (EC 1.2.1.12) - mouse (MGC68323), mRNA.

scl00234797.2_307-S	38.8116	1.73	6430548M08Rik	NM_172286.2	Mus musculus RIKEN cDNA 6430548M08 gene (6430548M08Rik), mRNA.
scl30672.9.1_29-S	38.7339	1.5	Ppp4c	NM_019674.2	Mus musculus protein phosphatase 4, catalytic subunit (Ppp4c), mRNA.
scl31356.7.1_27-S	38.4871	1.44	Emp3	NM_010129	Mus musculus epithelial membrane protein 3 (Emp3), mRNA.
scl0002191.1_543-S	38.3957	2.06	Svil	NM_153153	Mus musculus supervillin (Svil), mRNA.
scl51004.4_55-S	38.2238	1.71	Sepx1	NM_013759.1	
scl20642.5.1_21-S	38.0252	3.01	Sfpi1	NM_011355.1	Mus musculus SFFV proviral integration 1 (Sfpi1), mRNA.
scl38135.15_73-S	38.0106	2.43	Myb	NM_010848.2	Mus musculus myeloblastosis oncogene (Myb), mRNA.
scl000172.1_1-S	37.8641	2.26	Gmfg	NM_022024.1	Mus musculus glia maturation factor, gamma (Gmfg), mRNA.
scl000527.1_11-S	37.761	4.88	Ms4a3	NM_133246.2	Mus musculus membrane-spanning 4-domains, subfamily A, member 3 (Ms4a3), mRNA.
scl29264.8.1_214-S	37.7023	2.34	Tcfec	NM_031198.1	Mus musculus transcription factor EC (Tcfec), mRNA.
scl012512.6_24-S	37.5595	1.87	Cd63	NM_007653.1	Mus musculus Cd63 antigen (Cd63), mRNA.
scl017474.5_5-S	36.9744	2.81	Clecsf8	NM_010819.1	Mus musculus C-type (calcium dependent, carbohydrate recognition domain) lectin, superfamily member 8 (Clecsf8), mRNA.
scl41300.23_511-S	36.9048	1.42	Atp2a3	NM_016745.2	Mus musculus ATPase, Ca++ transporting, ubiquitous (Atp2a3), mRNA.
scl44463.11.22_177-S	36.7738	1.63	Smn1	NM_011420.1	
scl0056722.2_11-S	36.6369	1.35	Litaf	NM_019980.1	Mus musculus LPS-induced TN factor (Litaf), mRNA.
scl26663.16_246-S	36.2871	1.37	Wdr1	NM_011715.1	Mus musculus WD repeat domain 1 (Wdr1), mRNA.
scl50917.9.3_9-S	36.2421	4.42	Mapk13	NM_011950.1	Mus musculus mitogen activated protein kinase 13 (Mapk13), mRNA.
scl0012659.1_5-S	36.1239	3.61	Ovgp1	NM_007696.2	Mus musculus oviductal glycoprotein 1 (Ovgp1), mRNA.
scl000135.1_51-S	35.5586	2.37	Nkg7	NM_024253.3	Mus musculus natural killer cell group 7 sequence (Nkg7), mRNA.
scl26949.8.1_14-S	35.4348	4.07	Alox5ap	NM_009663	Mus musculus arachidonate 5-lipoxygenase activating protein (Alox5ap), mRNA.
scl0001732.1_22-S	35.0146	2.45	Sepx1	NM_013759.1	
scl30807.22.1_70-S	34.7359	1.47	Mrvi1	NM_194464.1	Mus musculus MRV integration site 1 (Mrvi1), transcript variant 1, mRNA.
scl0242700.9_212-S	34.6494	3.85	Il28ra	NM_174851.2	Mus musculus interleukin 28 receptor alpha (Il28ra), mRNA.
scl0003321.1_53-S	34.5316	4.09	Fcnb	XM_130120.1	Mus musculus ficolin B (Fcnb), mRNA.
scl41147.2_666-S	34.4353	1.88	Slfn1	NM_011407.1	Mus musculus schlafen 1 (Slfn1), mRNA.
scl36902.11_284-S	34.332	1.43	Scamp2	NM_022813.2	Mus musculus secretory carrier membrane protein 2 (Scamp2), mRNA.
scl0014571.1_161-S	34.3199	1.61	Gpd2	NM_010274.2	Mus musculus glycerol phosphate dehydrogenase 2, mitochondrial (Gpd2), mRNA.
scl068089.7_12-S	34.2431	1.57	Arpc4	NM_026552	Mus musculus actin related protein 2/3 complex, subunit 4 (Arpc4), mRNA.
scl0019336.1_63-S	33.9742	1.68	Rab24	NM_009000.2	Mus musculus RAB24, member RAS oncogene family (Rab24), mRNA.
scl28522.14.1_1-S	33.7133	5.12	9030407H20Rik	NM_144805.1	Mus musculus RIKEN cDNA 9030407H20 gene (9030407H20Rik), mRNA.
scl16258.13.1_64-S	33.6867	1.65	Rnpep	NM_145417	Mus musculus arginyl aminopeptidase (aminopeptidase B) (Rnpep), mRNA.
scl19446.4_4-S	33.6048	3.7	Lcn2	NM_008491.1	Mus musculus lipocalin 2 (Lcn2), mRNA.
scl50794.7.1_43-S	33.3363	1.5	Clic1	NM_033444.1	Mus musculus chloride intracellular channel 1 (Clic1), mRNA.
scl018950.7_181-S	33.2694	1.46	Pnp	NM_013632.2	Mus musculus purine-nucleoside phosphorylase (Pnp), mRNA.
scl0110253.14_323-S	33.2441	1.4	Triobp	NM_138579.2	Mus musculus TRIO and F-actin binding protein (Triobp), mRNA.
scl48582.2_713-S	33.1814	1.55	Gp5	NM_008148.2	Mus musculus glycoprotein 5 (platelet) (Gp5), mRNA.
scl33039.2.1_179-S	33.0056	2.94	Ptgir	NM_008967.1	Mus musculus prostaglandin I receptor (IP) (Ptgir), mRNA.
scl021991.1_200-S	32.8239	1.65	Tpi1	NM_009415.1	
scl24620.16_177-S	32.6204	1.32	BC004012	NM_138671	Mus musculus cDNA sequence BC004012 (BC004012), mRNA.
scl0059069.1_170-S	32.6013	1.32	Tpm3	NM_022314.2	Mus musculus tropomyosin 3, gamma (Tpm3), mRNA.
scl066151.5_253-S	32.5835	1.31	1110020C13Rik	NM_025385.2	Mus musculus RIKEN cDNA 1110020C13 gene (1110020C13Rik), mRNA.
scl0110196.3_64-S	32.3891	1.88	Fdps	NM_134469.2	Mus musculus farnesyl diphosphate synthetase (Fdps), mRNA.
scl017002.17_74-S	32.21	3.06	Ltf	NM_008522.2	Mus musculus lactotransferrin (Ltf), mRNA.
scl17239.5.1_240-S	32.1646	1.69	Fcrl3	NM_144559.1	Mus musculus Fc receptor-like 3 (Fcrl3), mRNA.
scl36089.6_17-S	31.9133	1.49	2310075A12Rik	NM_178027.3	
scl41130.3.1_26-S	31.8848	2.17	1100001G20Rik	NM_183249.1	Mus musculus RIKEN cDNA 1100001G20 gene (1100001G20Rik), mRNA.
scl27250.2_266-S	31.5112	1.95	D730049H07Rik	NM_175423	Mus musculus RIKEN cDNA D730049H07 gene (D730049H07Rik), mRNA.
scl0002340.1_98-S	31.2171	1.67	Nin	NM_008697	Mus musculus ninein (Nin), mRNA.
scl017063.13_302-S	31.2045	2.2	Ly64	NM_010739.1	Mus musculus lymphocyte antigen 64 (Ly64), mRNA.
scl41521.1.2_20-S	31.0848	2.22	4930438A08Rik	XM_126175.2	Mus musculus RIKEN cDNA 4930438A08 gene (4930438A08Rik), mRNA.
scl0022608.1_233-S	31.0426	1.28	Nsep1	NM_011732.1	Mus musculus nuclease sensitive element binding protein 1 (Nsep1), mRNA.
scl30675.13.1_1-S	30.986	1.3	Coro1a	NM_009898.2	Mus musculus coronin, actin binding protein 1A (Coro1a), mRNA.
scl016784.1_5-S	30.9448	1.34	Lamp2	NM_010685.2	Mus musculus lysosomal membrane glycoprotein 2 (Lamp2), mRNA.
scl51416.9_257-S	30.8775	5.55	Lox	NM_010728.1	Mus musculus lysyl oxidase (Lox), mRNA.
scl000502.1_59-S	30.8698	2.44	Prdx5	NM_012021.1	Mus musculus peroxiredoxin 5 (Prdx5), mRNA.
scl26323.5.1_40-S	30.5963	1.72	Plac8	NM_139198.1	Mus musculus placenta-specific 8 (Plac8), mRNA.

scl35598.8_175-S	30.5218	1.7	Mapk6	NM_015806.2	Mus musculus mitogen-activated protein kinase 6 (Mapk6), mRNA.
scl0003450.1_9-S	30.1398	2.12	Cklfsf7	NM_133978.1	Mus musculus chemokine-like factor super family 7 (Cklfsf7), mRNA.
scl020861.1_5-S	29.9964	10.38	Stfa1	NM_001001332	Mus musculus stefin A1 (Stfa1), mRNA.
scl52691.13_82-S	29.9437	1.71	Psat1	XM_129211.2	Mus musculus phosphoserine aminotransferase 1 (Psat1), mRNA.
scl35222.4_425-S	29.9389	2.1	Myd88	NM_010851	Mus musculus myeloid differentiation primary response gene 88 (Myd88), mRNA.
scl30947.3_219-S	29.8038	1.6	Rhog	NM_019566.2	Mus musculus ras homolog gene family, member G (Rhog), mRNA.
scl18197.5_22-S	29.7097	1.4	Rgs19	NM_026446.2	Mus musculus regulator of G-protein signaling 19 (Rgs19), mRNA.
scl00234825.2_277-S	29.1477	1.4	Klhdc4	NM_145605.1	
scl52908.26.6_30-S	28.8941	1.44	2610041P08Rik	NM_198008.1	Mus musculus RIKEN cDNA 2610041P08 gene (2610041P08Rik), mRNA.
scl33694.4.1_117-S	28.8729	1.33	Pgls	NM_025396.1	Mus musculus 6-phosphogluconolactonase (Pgls), mRNA.
scl0213673.3_37-S	28.7827	1.45	9530068E07Rik	XM_203329.2	Mus musculus RIKEN cDNA 9530068E07 gene (9530068E07Rik), mRNA.
scl0276770.1_104-S	28.7242	1.41	Eif5a	NM_181582.2	Mus musculus eukaryotic translation initiation factor 5A (Eif5a), mRNA.
scl0020862.1_160-S	28.3977	10.46	Stfa2	XM_148531.3	Mus musculus stefin A2 (Stfa2), mRNA.
scl0078334.1_222-S	28.2837	1.47	2700084L06Rik	NM_198164.1	Mus musculus RIKEN cDNA 2700084L06 gene (2700084L06Rik), mRNA.
scl012530.13_135-S	28.2018	1.89	Cdc25a	NM_007658	Mus musculus cell division cycle 25 homolog A (S. cerevisiae) (Cdc25a), mRNA.
scl32756.4.1_7-S	28.0063	2.49	Nkg7	NM_024253	Mus musculus natural killer cell group 7 sequence (Nkg7), mRNA.
scl47133.29_179-S	27.8524	1.63	Ddef1	NM_010026.1	Mus musculus development and differentiation enhancing (Ddef1), mRNA.
scl19517.6_242-S	27.8296	1.31	Surf4	NM_011512.2	Mus musculus surfeit gene 4 (Surf4), mRNA.
scl45774.17.1_29-S	27.8216	1.44	Prkcd	NM_011103.1	Mus musculus protein kinase C, delta (Prkcd), mRNA.
scl072042.2_8-S	27.8143	1.86	Cotl1	XM_150115.1	Mus musculus coactosin-like 1 (Dictyostelium) (Cotl1), mRNA.
scl012331.2_166-S	27.7137	1.32	Cap1	NM_007598.2	Mus musculus CAP, adenylate cyclase-associated protein 1 (yeast) (Cap1), mRNA.
scl29744.12_13-S	27.6622	1.72	1200015A22Rik	NM_028766.1	Mus musculus RIKEN cDNA 1200015A22 gene (1200015A22Rik), mRNA.
scl54134.13_56-S	27.5631	3.59	G6pdx	NM_008062.1	Mus musculus glucose-6-phosphate dehydrogenase X-linked (G6pdx), mRNA.
scl20145.4.1_7-S	27.318	4.16	Cst7	NM_009977.1	Mus musculus cystatin F (leukocystatin) (Cst7), mRNA.
scl48556.8.1_18-S	27.188	1.33	2010319C14Rik	NM_024464.2	Mus musculus RIKEN cDNA 2010319C14 gene (2010319C14Rik), mRNA.
scl37715.13_93-S	27.1217	3.51	Lmnb2	NM_010722.2	Mus musculus lamin B2 (Lmnb2), mRNA.
scl38698.8_214-S	26.8665	1.41	Cnn2	NM_007725.1	Mus musculus calponin 2 (Cnn2), mRNA.
scl28709.9_141-S	26.49	1.29	Abtb1	NM_030251.1	Mus musculus ankyrin repeat and BTB (POZ) domain containing 1 (Abtb1), mRNA.
scl0023821.2_49-S	26.4696	1.45	Bace1	NM_011792.3	Mus musculus beta-site APP cleaving enzyme 1 (Bace1), mRNA.
scl00394432.2_137-S	26.2339	2.48	Ugt1a10	NM_201642	
scl42858.13_56-S	26.1544	1.48	Rin3	NM_177620.2	Mus musculus Ras and Rab interactor 3 (Rin3), mRNA.
scl36146.3_205-S	26.0953	1.35	Cdkn2d	NM_009878.2	Mus musculus cyclin-dependent kinase inhibitor 2D (p19, inhibits CDK4) (Cdkn2d), mRNA.
scl020195.2_6-S	26.0905	1.81	S100a11	NM_016740	Mus musculus S100 calcium binding protein A11 (calizzarin) (S100a11), mRNA.
scl071784.1_62-S	26.0782	2.93	1110007C02Rik	NM_027923.1	Mus musculus RIKEN cDNA 1110007C02 gene (1110007C02Rik), mRNA.
scl0030955.2_290-S	26.0576	1.41	Pik3cg	NM_020272.1	Mus musculus phosphoinositide-3-kinase, catalytic, gamma polypeptide (Pik3cg), mRNA.
scl0072685.1_143-S	25.8438	1.55	Dnajc6	NM_198412.1	Mus musculus DnaJ (Hsp40) homolog, subfamily C, member 6 (Dnajc6), mRNA.
scl16996.9.1_203-S	25.8007	13	4930418G15Rik	NM_145692.1	
scl31009.10_67-S	25.6067	2.33	Dgat2	NM_026384.2	Mus musculus diacylglycerol O-acyltransferase 2 (Dgat2), mRNA.
scl54141.46.3_34-S	25.5567	1.37	Flna	XM_289920.2	Mus musculus filamin, alpha (Flna), mRNA.
scl000111.1_99-S	25.2804	1.31	Coro1a	NM_009898.2	Mus musculus coronin, actin binding protein 1A (Coro1a), mRNA.
scl50576.26.1_29-S	25.1698	1.46	Vav1	NM_011691.2	Mus musculus vav 1 oncogene (Vav1), mRNA.
scl33196.2.1635_134-S	25.141	2.13	Rhou	NM_133955.1	Mus musculus ras homolog gene family, member U (Rhou), mRNA.
scl0054683.2_242-S	25.1015	1.98	Prdx5	NM_012021.1	Mus musculus peroxiredoxin 5 (Prdx5), mRNA.
scl41842.10.1_70-S	24.8912	4.16	Upp1	NM_009477.1	Mus musculus uridine phosphorylase 1 (Upp1), mRNA.
scl056378.6_20-S	24.5889	1.38	Arpc3	NM_019824.2	Mus musculus actin related protein 2/3 complex, subunit 3 (Arpc3), mRNA.
scl39908.21.1_34-S	24.5384	1.77	Cpd	NM_007754.1	Mus musculus carboxypeptidase D (Cpd), mRNA.
scl064382.1_42-S	24.3121	1.56	Ms4a6d	NM_026835.1	Mus musculus membrane-spanning 4-domains, subfamily A, member 11 (Ms4a11), mRNA.
scl50935.5_66-S	24.2356	1.83	Hmga1	NM_016660.1	Mus musculus high mobility group AT-hook 1 (Hmga1), mRNA.
scl18985.4_291-S	23.9705	1.43	Slc35c1	NM_145832.2	
scl0231807.1_240-S	23.8969	1.61	BC037034	NM_153161.1	Mus musculus cDNA sequence BC037034 (BC037034), mRNA.
scl00102103.1_84-S	23.8366	3.79	Mtus1	NM_178902.3	Mus musculus mitochondrial tumor suppressor 1 (Mtus1), mRNA.
scl080876.2_10-S	23.8155	1.77	Ifitm2	NM_030694	Mus musculus interferon induced transmembrane protein 2 (Ifitm2), mRNA.
scl54485.6_175-S	23.4874	1.53	Piga	NM_011081.1	Mus musculus phosphatidylinositol glycan, class A (Piga), mRNA.
scl29411.4.6_15-S	23.3167	1.79	Mgst1	NM_019946.3	Mus musculus microsomal glutathione S-transferase 1 (Mgst1), mRNA.
scl016541.8_1-S	23.2856	1.43	Napsa	NM_008437.1	
scl021937.10_167-S	23.1485	1.38	Tnfrsf1a	NM_011609.2	Mus musculus tumor necrosis factor receptor superfamily, member 1a (Tnfrsf1a), mRNA.

scl50208.9_12-S	23.1179	1.67	Gbl	NM_019988.2	Mus musculus G protein beta subunit-like (Gbl), mRNA.
scl37649.7_307-S	23.0994	1.56	1200002N14Rik	NM_027878.1	Mus musculus RIKEN cDNA 1200002N14 gene (1200002N14Rik), mRNA.
scl27205.18_385-S	22.9503	1.79	Aacs	NM_030210.1	Mus musculus acetoacetyl-CoA synthetase (Aacs), mRNA.
scl41405.25_9-S	22.8023	2.36	Gas7	NM_008088	Mus musculus growth arrest specific 7 (Gas7), mRNA.
scl0107934.22_249-S	22.7214	6.19	Celsr3	NM_080437.1	Mus musculus cadherin EGF LAG seven-pass G-type receptor 3 (Celsr3), mRNA.
scl47670.15_189-S	22.6491	1.69	Parvg	NM_022321.2	Mus musculus parvin, gamma (Parvg), mRNA.
scl013244.1_23-S	22.59	1.29	Degs	NM_007853.2	Mus musculus degenerative spermatocyte homolog (Drosophila) (Degs), mRNA.
scl37316.9.1_41-S	22.5853	1.85	Casp4	NM_007609.1	Mus musculus caspase 4, apoptosis-related cysteine protease (Casp4), mRNA.
scl46585.22_265-S	22.557	1.34	Vcl	NM_009502	Mus musculus vinculin (Vcl), mRNA.
scl52675.8.401_6-S	22.4657	1.44	Ostf1	NM_017375.1	Mus musculus osteoclast stimulating factor 1 (Ostf1), mRNA.
scl0230709.2_6-S	22.423	2.1	Zmpste24	NM_172700.1	Mus musculus zinc metalloproteinase, STE24 homolog (S. cerevisiae) (Zmpste24), mRNA.
scl18632.11_218-S	22.3331	1.42	Rassf2	NM_175445.3	Mus musculus Ras association (RalGDS/AF-6) domain family 2 (Rassf2), mRNA.
scl39819.5_328-S	22.1603	3.06	Ccl9	NM_011338	Mus musculus chemokine (C-C motif) ligand 9 (Ccl9), mRNA.
scl37751.5.62_171-S	22.1444	10.55	Prssl1	XM_196763.2	
scl52790.5_512-S	21.9702	1.8	1810055G02Rik	NM_028077.1	Mus musculus RIKEN cDNA 1810055G02 gene (1810055G02Rik), mRNA.
scl19499.9.1_0-S	21.9338	3.44	Fcnb	XM_130120.1	Mus musculus ficolin B (Fcnb), mRNA.
scl35462.25_609-S	21.8188	1.32	Pik3cb	NM_029094.1	Mus musculus phosphatidylinositol 3-kinase, catalytic, beta polypeptide (Pik3cb), mRNA.
scl0009.1_32-S	21.7075	2.86	Pnkp	NM_021549.1	Mus musculus polynucleotide kinase 3- phosphatase (Pnkp), mRNA.
scl53163.10.1_0-S	21.6956	6.12	1200008O12Rik	NM_028760.1	Mus musculus RIKEN cDNA 1200008O12 gene (1200008O12Rik), mRNA.
scl069101.5_23-S	21.6653	1.87	1810015A11Rik	XM_148046.3	Mus musculus RIKEN cDNA 1810015A11 gene (1810015A11Rik), mRNA.
scl34254.8_613-S	21.6284	1.34	Zdhhc7	NM_133967.2	Mus musculus zinc finger, DHHC domain containing 7 (Zdhhc7), mRNA.
scl28929.1_28-S	21.6036	2.38	Tacstd2	NM_020047.2	Mus musculus tumor-associated calcium signal transducer 2 (Tacstd2), mRNA.
scl26088.14.1_16-S	21.5141	1.34	Aldh2	NM_009656.1	Mus musculus aldehyde dehydrogenase 2, mitochondrial (Aldh2), mRNA.
scl022038.3_2-S	21.446	3.08	Plscr1	NM_011636.1	Mus musculus phospholipid scramblase 1 (Plscr1), mRNA.
scl0020660.2_10-S	21.4444	1.45	Sorl1	NM_011436	Mus musculus sortilin-related receptor, LDLR class A repeats-containing (Sorl1), mRNA.
scl00230316.2_241-S	21.4425	2.4	Egfl5	NM_172694.1	Mus musculus EGF-like-domain, multiple 5 (Egfl5), mRNA.
scl36516.9.290_30-S	21.4408	1.7	Ptk9l	NM_011876.2	Mus musculus protein tyrosine kinase 9-like (A6-related protein) (Ptk9l), mRNA.
scl00216971.1_119-S	21.3847	1.49	BC017647	NM_145430.1	Mus musculus cDNA sequence BC017647 (BC017647), mRNA.
scl40050.12_410-S	21.3841	1.39	Ndel1	NM_023668.1	Mus musculus nuclear distribution gene E-like homolog 1 (A. nidulans) (Ndel1), mRNA.
scl50927.13.1_12-S	21.2682	1.95	Def6	NM_027185.1	Mus musculus differentially expressed in FDCP 6 (Def6), mRNA.
scl030931.1_245-S	21.207	1.37	Dyt1	NM_144884.1	Mus musculus dystonia 1 (Dyt1), mRNA.
scl00230257.2_191-S	21.115	1.35	Rod1	NM_144904.1	Mus musculus ROD1 regulator of differentiation 1 (S. pombe) (Rod1), mRNA.
scl23697.3.1_58-S	21.0586	1.53	Sh3bgrl3	NM_080559.1	Mus musculus SH3 domain binding glutamic acid-rich protein-like 3 (Sh3bgrl3), mRNA.
scl00233046.2_82-S	21.0346	7.85	Rasgrp4	NM_145149.1	Mus musculus RAS guanyl releasing protein 4 (Rasgrp4), mRNA.
scl00218581.2_84-S	21.028	1.36	Depdc1b	NM_178683.2	
scl014862.2_242-S	20.9678	1.89	Gstm1	NM_010358.2	Mus musculus glutathione S-transferase, mu 1 (Gstm1), mRNA.
scl33129.9_499-S	20.9638	1.33	Suv420h2	NM_146177.1	
scl000075.1_18_REVCOM	20.9358	1.38	AA959742	NM_133807.1	Mus musculus expressed sequence AA959742 (AA959742), mRNA.
scl20444.23.1_130-S	20.8933	1.48	Bub1b	NM_009773	Mus musculus budding uninhibited by benzimidazoles 1 homolog, beta (S. cerevisiae) (Bub1b), mRNA.
scl015331.1_246-S	20.6317	1.46	Hmgn2	NM_016957.2	Mus musculus high mobility group nucleosomal binding domain 2 (Hmgn2), mRNA.
scl22994.6_49-S	20.6004	1.37	Ssr2	NM_025448.2	Mus musculus signal sequence receptor, beta (Ssr2), mRNA.
scl53421.25_339-S	20.5607	1.39	D19Ertd703e	NM_029456.1	Mus musculus DNA segment, Chr 19, ERATO Doi 703, expressed (D19Ertd703e), mRNA.
scl030963.1_56-S	20.5155	1.44	Ptpla	NM_013935.1	Mus musculus protein tyrosine phosphatase-like (proline instead of catalytic arginine), member a (Ptpla), mRNA.
scl39392.13.1_3-S	20.4994	1.62	D11Ertd498e	NM_145940.1	Mus musculus DNA segment, Chr 11, ERATO Doi 498, expressed (D11Ertd498e), mRNA.
scl013728.1_329-S	20.4404	1.24	Mark2	NM_007928.1	Mus musculus MAP/microtubule affinity-regulating kinase 2 (Mark2), mRNA.
scl39705.9.188_7-S	20.3489	1.54	6820428D13	NM_177752.2	Mus musculus hypothetical protein 6820428D13 (6820428D13), mRNA.
scl48826.10.1_4-S	20.2782	2.38	Mefv	NM_019453.1	Mus musculus Mediterranean fever (Mefv), mRNA.
scl066989.6_1-S	20.2522	1.31	2410004N11Rik	NM_025888.2	Mus musculus RIKEN cDNA 2410004N11 gene (2410004N11Rik), mRNA.
scl18719.12.1_11-S	20.1896	11.96	Hdc	NM_008230	Mus musculus histidine decarboxylase (Hdc), mRNA.
scl0018728.1_38-S	20.0412	1.54	Pira5		

GENES DOWN-REGULATED BY OPP IN SPLEENS

TargetID	Diff_Score	Fold_Change	Symbol	Accession	Definition
scl31155.10_11-S	-150.3732	-2.36	Mfge8	NM_008594	Mus musculus milk fat globule-EGF factor 8 protein (Mfge8), mRNA.
scl017110.1_293-S	-110.2585	-5.82	Lzp-s	NM_013590.2	Mus musculus P lysozyme structural (Lzp-s), mRNA.
scl24842.10.1_114-S	-92.4965	-1.71	Rhced	NM_011270.2	Mus musculus Rhesus blood group CE and D (Rhced), mRNA.
scl39449.6.1_88-S	-86.3639	-2.28	Cd79b	NM_008339.1	Mus musculus CD79B antigen (Cd79b), mRNA.

scl0012045.1_114-S	-85.6766	-2.31	Bcl2a1b	NM_007534	Mus musculus B-cell leukemia/lymphoma 2 related protein A1b (Bcl2a1b), mRNA.
scl49992.7.1_104-S	-77.7966	-2.21	Aif1	NM_019467.2	Mus musculus allograft inflammatory factor 1 (Aif1), mRNA.
scl0012047.1_125-S	-74.0994	-2.44	Bcl2a1d	NM_007536	Mus musculus B-cell leukemia/lymphoma 2 related protein A1d (Bcl2a1d), mRNA.
scl000673.1_3-S	-72.1369	-1.53	Prdx2	NM_011563	Mus musculus peroxiredoxin 2 (Prdx2), mRNA.
scl32668.4.1_181-S	-70.6727	-3.7	Dbp	NM_016974.1	Mus musculus D site albumin promoter binding protein (Dbp), mRNA.
scl32898.7.1_80-S	-66.458	-1.54	Blvrb	NM_144923	Mus musculus biliverdin reductase B (flavin reductase (NADPH)) (Blvrb), mRNA.
scl50010.18.1_105-S	-65.9185	-1.76	H2-Bf	NM_008198.1	Mus musculus histocompatibility 2, complement component factor B (H2-Bf), mRNA.
scl26445.6.1_1-S	-65.7973	-1.76	Igfbp7	NM_008048.1	Mus musculus insulin-like growth factor binding protein 7 (Igfbp7), mRNA.
scl0012044.1_118-S	-63.9154	-2.41	Bcl2a1a	NM_009742.2	Mus musculus B-cell leukemia/lymphoma 2 related protein A1a (Bcl2a1a), mRNA.
scl015024.2_236-S	-63.1097	-2.06	H2-T10	NM_010395.2	Mus musculus histocompatibility 2, T region locus 10 (H2-T10), mRNA.
scl44840.4_185-S	-61.3393	-3.14	Cd83	NM_009856.1	Mus musculus CD83 antigen (Cd83), mRNA.
scl24887.7_12-S	-60.4783	-1.81	Sdc3	NM_011520.2	Mus musculus syndecan 3 (Sdc3), mRNA.
scl50824.6.23_33-S	-60.1189	-2.36	H2-Eb1	NM_010382.1	Mus musculus histocompatibility 2, class II antigen E beta (H2-Eb1), mRNA.
scl40203.12_25-S	-59.9973	-1.74	Sparc	NM_009242	Mus musculus secreted acidic cysteine rich glycoprotein (Sparc), mRNA.
scl42795.16.1_26-S	-56.4908	-2.4	Evl	NM_007965.2	Mus musculus Ena-vasodilator stimulated phosphoprotein (Evl), mRNA.
scl33241.10_186-S	-52.9856	-2.06	Icsbp1	NM_008320.2	Mus musculus interferon consensus sequence binding protein 1 (Icsbp1), mRNA.
scl0002344.1_54-S	-52.5567	-1.62	Serpina1b	NM_009244.2	Mus musculus serine (or cysteine) proteinase inhibitor, clade A, member 1b (Serpina1b), mRNA.
scl50575.22.1_51-S	-52.1957	-1.96	Emr1	NM_010130.1	Mus musculus EGF-like module containing, mucin-like, hormone receptor-like sequence 1 (Emr1), mRNA.
scl0054725.2_225-S	-51.3813	-2.45	Igsf4a	NM_018770.2	Mus musculus immunoglobulin superfamily, member 4A (Igsf4a), transcript variant 3, mRNA.
scl44935.7_355-S	-51.004	-1.91	Serpinb6b	NM_011454.1	Mus musculus serine (or cysteine) proteinase inhibitor, clade B, member 6b (Serpinb6b), mRNA.
scl15950.5.1_27-S	-50.2892	-2.91	BB219290	NM_145141.1	Mus musculus expressed sequence BB219290 (BB219290), mRNA.
scl0020452.1_241-S	-49.5814	-1.67	Siat8d	NM_009183.1	Mus musculus sialyltransferase 8 (alpha-2, 8-sialyltransferase) D (Siat8d), mRNA.
scl41638.9.1_29-S	-48.4644	-2.01	Timd4	NM_178759.3	
scl45915.9_517-S	-47.8759	-2.41	Dnase1l3	NM_007870.2	Mus musculus deoxyribonuclease 1-like 3 (Dnase1l3), mRNA.
scl024047.3_30-S	-47.6093	-2.89	Ccl19	NM_011888	Mus musculus chemokine (C-C motif) ligand 19 (Ccl19), mRNA.
gi_30794511_ref_NM_01	-47.5664	-1.42	Hmbs	NM_013551.1	Mus musculus hydroxymethylbilane synthase (Hmbs), mRNA.
scl00333883.1_4-S	-47.0033	-3.02	Cd59b	NM_181858.1	Mus musculus CD59b antigen (Cd59b), mRNA.
scl40209.28.1_48-S	-46.9617	-2	Anxa6	NM_013472.2	Mus musculus annexin A6 (Anxa6), mRNA.
scl015032.1_236-S	-46.8994	-1.66	H2-T17	NM_010396	Mus musculus histocompatibility 2, T region locus 17 (H2-T17), mRNA.
scl071994.3_187-S	-45.7307	-1.87	Cnn3	NM_028044.1	Mus musculus calponin 3, acidic (Cnn3), mRNA.
scl45393.10_71-S	-45.5954	-1.4	Bnip3l	NM_009761.2	Mus musculus BCL2/adenovirus E1B 19kDa-interacting protein 3-like (Bnip3l), mRNA.
scl50024.4.1_14-S	-44.9565	-2.22	H2-Ea	NM_010381.2	Mus musculus histocompatibility 2, class II antigen E alpha (H2-Ea), mRNA.
scl068066.1_115-S	-44.9504	-1.46	D11Ertd333e	NM_026542.1	
scl0001934.1_38-S	-44.719	-1.43	Lmna	NM_019390.1	Mus musculus lamin A (Lmna), mRNA.
scl32943.6.1_71-S	-43.9534	-1.97	Cd79a	NM_007655.1	Mus musculus CD79A antigen (immunoglobulin-associated alpha) (Cd79a), mRNA.
scl24261.12.1_29-S	-43.488	-1.36	Alad	NM_008525.3	Mus musculus aminolevulinate, delta-, dehydratase (Alad), mRNA.
scl012406.1_153-S	-43.2846	-2.36	Serpinh1	NM_009825	Mus musculus serine (or cysteine) proteinase inhibitor, clade H, member 1 (Serpinh1), mRNA.
scl056620.6_30-S	-43.2818	-1.93	Clecsf10	NM_020001.1	Mus musculus C-type (calcium dependent, carbohydrate recognition domain) lectin, superfamily member 10 (Clecsf10), mRNA.
scl0012192.2_207-S	-43.255	-1.93	Zfp36l1	NM_007564.2	Mus musculus zinc finger protein 36, C3H type-like 1 (Zfp36l1), mRNA.
scl42115.9.1_121-S	-42.5817	-3.01	Asb2	NM_023049.1	Mus musculus ankyrin repeat and SOCS box-containing protein 2 (Asb2), mRNA.
scl35771.8.1_41-S	-42.224	-1.73	2410076I21Rik	XM_134948.2	Mus musculus RIKEN cDNA 2410076I21 gene (2410076I21Rik), mRNA.
scl35076.15.1_19-S	-42.0972	-1.97	Gas6	NM_019521.1	Mus musculus growth arrest specific 6 (Gas6), mRNA.
scl22880.8.1_176-S	-41.7557	-1.58	Ctss	NM_021281.1	Mus musculus cathepsin S (Ctss), mRNA.
scl0015312.2_169-S	-41.6335	-1.38	Hmgn1	NM_008251.3	Mus musculus high mobility group nucleosomal binding domain 1 (Hmgn1), mRNA.
scl39660.7_333-S	-41.342	-1.59	AI415282	NM_134021.1	Mus musculus expressed sequence AI415282 (AI415282), mRNA.
scl0012443.2_180-S	-40.6239	-3.34	Ccnd1	NM_007631.1	Mus musculus cyclin D1 (Ccnd1), mRNA.
scl015953.2_220-S	-40.1407	-1.58	Ifi47	NM_008330.1	Mus musculus interferon gamma inducible protein (Ifi47), mRNA.
scl014204.4_298-S	-39.6029	-2.8	Il4i1	NM_010215.1	Mus musculus interleukin 4 induced 1 (Il4i1), mRNA.
scl056812.9_277-S	-39.5462	-1.65	Dnajb10	NM_178055.2	Mus musculus DnaJ (Hsp40) homolog, subfamily B, member 10 (Dnajb10), mRNA.
scl000591.1_21-S	-39.5074	-1.89	Ifi30	NM_023065.2	Mus musculus interferon gamma inducible protein 30 (Ifi30), mRNA.
scl50736.3.1_4-S	-39.431	-2.86	Ubd	NM_023137.2	Mus musculus ubiquitin D (Ubd), mRNA.
scl29767.12_42-S	-38.7882	-1.46	Mgll	NM_011844.3	Mus musculus monoglyceride lipase (Mgll), mRNA.
scl42725.12.1_79-S	-38.3939	-1.81	AI132321	NM_178911.2	Mus musculus expressed sequence AI132321 (AI132321), mRNA.
scl16803.9_203-S	-38.3231	-1.68	Slc40a1	NM_016917.1	Mus musculus solute carrier family 40 (iron-regulated transporter), member 1 (Slc40a1), mRNA.
scl26112.11_493-S	-38.1338	-3.17	Dtx1	NM_008052.1	Mus musculus deltex 1 homolog (Drosophila) (Dtx1), mRNA.
scl098766.1_292-S	-38.047	-1.41	Ubadc1	NM_133835.1	Mus musculus ubiquitin associated domain containing 1 (Ubadc1), mRNA.

scl00192657.1_320-S	-37.8008	-1.75	Ell2	NM_138953.1	Mus musculus elongation factor RNA polymerase II 2 (Ell2), mRNA.
scl0017329.2_123-S	-37.7309	-3.19	Cxcl9	NM_008599.1	Mus musculus chemokine (C-X-C motif) ligand 9 (Cxcl9), mRNA.
scl55061.19.1_283-S	-37.3568	-1.39	Slc38a5	NM_172479.1	
scl0002580.1_4-S	-37.0297	-1.8	Ptp4a3	NM_008975.2	Mus musculus protein tyrosine phosphatase 4a3 (Ptp4a3), mRNA.
scl50025.7.1_85-S	-36.8415	-2.58	H2-Aa	NM_010378.2	Mus musculus histocompatibility 2, class II antigen A, alpha (H2-Aa), mRNA.
scl52495.17.1_252-S	-36.7346	-1.94	Blnk	NM_008528.3	Mus musculus B-cell linker (Blnk), mRNA.
scl38917.7_7-S	-36.6364	-1.47	Gja1	NM_010288.2	Mus musculus gap junction membrane channel protein alpha 1 (Gja1), mRNA.
scl000781.1_68-S	-36.5623	-1.79	Kif21b	NM_019962.2	Mus musculus kinesin family member 21B (Kif21b), mRNA.
scl012521.1_26-S	-36.56	-1.39	Kai1	NM_007656.1	Mus musculus kangai 1 (suppression of tumorigenicity 6, prostate) (Kai1), mRNA.
scl0000100.1_15-S	-36.498	-2.33	Tnxb	NM_031176.1	Mus musculus tenascin XB (Tnxb), mRNA.
scl0108927.4_201-S	-36.4952	-1.98	2810489O06Rik	NM_175386.3	Mus musculus RIKEN cDNA 2810489O06 gene (2810489O06Rik), mRNA.
scl016149.9_25-S	-36.2526	-2.31	Ii	NM_010545.2	Mus musculus Ia-associated invariant chain (Ii), mRNA.
scl15766.4.1_27-S	-36.0119	-1.58	1110060M21Rik	NM_025424.1	Mus musculus RIKEN cDNA 1110060M21 gene (1110060M21Rik), mRNA.
scl52537.9_557-S	-35.9963	-1.58	Lip1	NM_021460.1	Mus musculus lysosomal acid lipase 1 (Lip1), mRNA.
scl43224.11.1_2-S	-35.7321	-3.29	Coch	NM_007728.2	Mus musculus coagulation factor C homolog (Limulus polyphemus) (Coch), mRNA.
scl45133.3_447-S	-35.6225	-2.22	Ebi2	NM_183031.1	Mus musculus Epstein-Barr virus induced gene 2 (Ebi2), mRNA.
scl012988.1_164-S	-35.4879	-1.5	Csk	NM_007783.2	Mus musculus c-src tyrosine kinase (Csk), mRNA.
scl39273.6_263-S	-35.41	-1.61	Lgals3bp	NM_011150.1	Mus musculus lectin, galactoside-binding, soluble, 3 binding protein (Lgals3bp), mRNA.
scl0012934.1_152-S	-35.0037	-1.47	Dpysl2	NM_009955.2	Mus musculus dihydropyrimidinase-like 2 (Dpysl2), mRNA.
scl067869.4_264-S	-34.7112	-1.38	Paip2	NM_026420.1	Mus musculus polyadenylate-binding protein-interacting protein 2 (Paip2), mRNA.
scl078416.2_288-S	-34.6518	-2.25	Rnase6	NM_030098.1	Mus musculus ribonuclease A family, member 6 (Rnase6), mRNA.
scl15755.10.583_56-S	-34.2306	-2.15	Traf5	NM_011633.1	Mus musculus Tnf receptor-associated factor 5 (Traf5), mRNA.
scl48501.9_645-S	-34.1575	-2	Cd86	NM_019388.2	Mus musculus CD86 antigen (Cd86), mRNA.
scl30833.27_114-S	-33.7314	-1.49	St5	NM_029811.1	Mus musculus suppression of tumorigenicity 5 (St5), mRNA.
scl39506.28.1461_10-S	-33.7135	-1.44	Slc4a1	NM_011403.1	Mus musculus solute carrier family 4 (anion exchanger), member 1 (Slc4a1), mRNA.
scl019122.2_199-S	-33.7062	-1.34	Prnp	NM_011170.1	Mus musculus prion protein (Prnp), mRNA.
scl31867.15.2675_48-S	-33.6962	-1.47	Lsp1	NM_019391.1	Mus musculus lymphocyte specific 1 (Lsp1), mRNA.
scl49300.12_501-S	-33.6366	-1.67	St6gal1	NM_145933.2	Mus musculus beta galactoside alpha 2,6 sialyltransferase 1 (St6gal1), mRNA.
scl015903.3_124-S	-33.491	-1.93	Idb3	NM_008321.1	Mus musculus inhibitor of DNA binding 3 (Idb3), mRNA.
scl31518.8.1_186-S	-33.4102	-3.16	Rog	NM_021397	Mus musculus repressor of GATA (Rog), mRNA.
scl0001712.1_14-S	-33.2107	-2.35	Aif1	NM_019467.2	Mus musculus allograft inflammatory factor 1 (Aif1), mRNA.
scl51913.28_530-S	-32.8277	-2.09	Slc12a2	NM_009194.1	Mus musculus solute carrier family 12, member 2 (Slc12a2), mRNA.
scl0069104.1_104-S	-32.7629	-1.36	2700055A20Rik	NM_027314.2	
scl41212.9.1_219-S	-32.5685	-4.15	Rab34	NM_033475.2	Mus musculus RAB34, member of RAS oncogene family (Rab34), mRNA.
scl47072.5_281-S	-32.4885	-2.29	Ly6a	NM_010738.2	Mus musculus lymphocyte antigen 6 complex, locus A (Ly6a), mRNA.
scl40613.6.1_112-S	-32.3026	-1.88	Fn3k	NM_022014.1	Mus musculus fructosamine 3 kinase (Fn3k), mRNA.
scl29467.5.1_148-S	-31.5862	-1.55	Ocil	NM_053109.1	Mus musculus osteoclast inhibitory lectin (Ocil), mRNA.
scl067804.15_75-S	-31.486	-1.4	Snx2	NM_026386.1	Mus musculus sorting nexin 2 (Snx2), mRNA.
scl018477.6_2-S	-31.4275	-1.3	Prdx1	NM_011034.2	Mus musculus peroxiredoxin 1 (Prdx1), mRNA.
scl0066748.2_33-S	-31.3084	-1.56	4933404M02Rik	NM_025744.1	Mus musculus RIKEN cDNA 4933404M02 gene (4933404M02Rik), mRNA.
scl012517.2_30-S	-31.2889	-2.17	Cd72	NM_007654.1	Mus musculus CD72 antigen (Cd72), mRNA.
scl0072691.1_193-S	-31.0601	-1.58	2810048G17Rik	NM_133746.2	Mus musculus RIKEN cDNA 2810048G17 gene (2810048G17Rik), mRNA.
scl021858.1_100-S	-30.8885	-1.58	Timp2	NM_011594.2	Mus musculus tissue inhibitor of metalloproteinase 2 (Timp2), mRNA.
scl018458.3_17-S	-30.8526	-1.29	Pabpc1	NM_008774.2	Mus musculus poly A binding protein, cytoplasmic 1 (Pabpc1), mRNA.
scl014998.4_34-S	-30.8218	-2.1	H2-DMa	NM_010386	Mus musculus histocompatibility 2, class II, locus DMa (H2-DMa), mRNA.
scl0117109.5_26-S	-30.7588	-1.53	Pop5	NM_026398.2	Mus musculus processing of precursor 5, ribonuclease P/MRP family (S. cerevisiae) (Pop5), mRNA.
scl020926.1_330-S	-30.6275	-1.41	Supt6h	NM_009297.1	Mus musculus suppressor of Ty 6 homolog (S. cerevisiae) (Supt6h), mRNA.
scl38222.21_67-S	-30.4085	-2.04	Sash1	NM_175155.3	
scl016173.8_28-S	-30.2566	-1.88	Il18	NM_008360.1	Mus musculus interleukin 18 (Il18), mRNA.
scl37236.8_286-S	-30.1638	-2.14	Icam1	NM_010493.2	Mus musculus intercellular adhesion molecule (Icam1), mRNA.
scl0012709.2_191-S	-30.0998	-1.86	Ckb	NM_021273.2	Mus musculus creatine kinase, brain (Ckb), mRNA.
scl26218.7.1_61-S	-30.0063	-1.66	AW049829	NM_153571.1	Mus musculus expressed sequence AW049829 (AW049829), mRNA.
scl014790.2_94-S	-30.0029	-1.53	Grcc10	NM_013535.1	Mus musculus gene rich cluster, C10 gene (Grcc10), mRNA.
scl0019191.1_143-S	-29.9119	-1.41	Psme2b	NM_011191.1	Mus musculus protease (prosome, macropain) 28 subunit, beta, b (Psme2b), mRNA.
scl0014961.2_33-S	-29.5785	-2.32	H2-Ab1	NM_010379.2	Mus musculus histocompatibility 2, class II antigen A, beta 1 (H2-Ab1), mRNA.
scl017118.3_6-S	-29.4431	-1.91	Marcks	NM_008538	Mus musculus myristoylated alanine rich protein kinase C substrate (Marcks), mRNA.

scl32713.7.1_59-S	-29.4263	-1.51	1110007C05Rik	NM_025368.1	Mus musculus RIKEN cDNA 1110007C05 gene (1110007C05Rik), mRNA.
scl0076933.2_252-S	-29.1924	-2.51	2310061N23Rik	NM_029803.1	Mus musculus RIKEN cDNA 2310061N23 gene (2310061N23Rik), mRNA.
scl0012846.1_253-S	-29.1493	-1.38	Comt	XM_147265.1	Mus musculus catechol-O-methyltransferase (Comt), mRNA.
scl41542.4_321-S	-29.1109	-1.86	Gm2a	NM_010299.2	Mus musculus GM2 ganglioside activator protein (Gm2a), mRNA.
scl0020054.1_111-S	-29.09	-1.33	Rps15	NM_009091.1	Mus musculus ribosomal protein S15 (Rps15), mRNA.
scl0003429.1_37-S	-29.0184	-1.43	Hmbs	NM_013551	Mus musculus hydroxymethylbilane synthase (Hmbs), mRNA.
scl0114887.6_24-S	-28.917	-2.87	Rmcs1	NM_207105.1	Mus musculus response to metastatic cancers 1 (Rmcs1), mRNA.
scl17503.7.1_197-S	-28.7865	-2.69	1810037B05Rik	XM_129390.2	Mus musculus RIKEN cDNA 1810037B05 gene (1810037B05Rik), mRNA.
scl39610.3_265-S	-28.7347	-2.28	Ccr7	NM_007719.1	Mus musculus chemokine (C-C motif) receptor 7 (Ccr7), mRNA.
scl20330.11.1_76-S	-28.6342	-1.37	Blvra	NM_026678.3	Mus musculus biliverdin reductase A (Blvra), mRNA.
scl37022.6.1_3-S	-28.5439	-1.55	Cd3d	NM_013487.1	Mus musculus CD3 antigen, delta polypeptide (Cd3d), mRNA.
scl0020846.2_62-S	-28.4378	-1.75	Stat1	NM_009283.2	Mus musculus signal transducer and activator of transcription 1 (Stat1), mRNA.
scl056367.1_256-S	-28.4043	-1.35	Scoc	XM_134455.1	Mus musculus short coiled-coil protein (Scoc), mRNA.
scl15933.11.1_5-S	-28.3623	-1.97	Ly9	NM_008534.1	Mus musculus lymphocyte antigen 9 (Ly9), mRNA.
scl54851.7_89-S	-28.3337	-1.76	Bgn	NM_007542.3	Mus musculus biglycan (Bgn), mRNA.
scl0015446.2_268-S	-28.1751	-2.01	Hpgd	NM_008278.1	Mus musculus hydroxyprostaglandin dehydrogenase 15 (NAD) (Hpgd), mRNA.
scl066158.1_320-S	-28.1222	-1.73	1110012O05Rik	NM_024170	Mus musculus RIKEN cDNA 1110012O05 gene (1110012O05Rik), mRNA.
scl065972.3_20-S	-28.0439	-1.75	Ifi30	NM_023065.2	Mus musculus interferon gamma inducible protein 30 (Ifi30), mRNA.
scl0056628.1_326-S	-28.0205	-2.24	LOC56628	NM_019909.1	Mus musculus MHC (A.CA/J(H-2K-f) class I antigen (LOC56628), mRNA.
scl0018854.2_223-S	-27.9955	-1.44	Pml	NM_008884.2	Mus musculus promyelocytic leukemia (Pml), mRNA.
scl45437.13.1_73-S	-27.9769	-2.25	Blk	NM_007549.2	Mus musculus B lymphoid kinase (Blk), mRNA.
scl0022214.2_142-S	-27.6497	-1.36	Ube2h	XM_133034.1	Mus musculus ubiquitin-conjugating enzyme E2H (Ube2h), mRNA.
scl45706.11_2-S	-27.6197	-1.46	Ghitm	NM_078478.1	Mus musculus growth hormone inducible transmembrane protein (Ghitm), mRNA.
scl23946.8.1_41-S	-27.4451	-1.27	Urod	NM_009478.1	Mus musculus uroporphyrinogen decarboxylase (Urod), mRNA.
scl022259.1_112-S	-27.392	-1.75	Nr1h3	NM_013839.1	Mus musculus nuclear receptor subfamily 1, group H, member 3 (Nr1h3), mRNA.
scl0231932.2_146-S	-27.3811	-2.64	Ian3	NM_146167.2	Mus musculus immune associated nucleotide 3 (Ian3), mRNA.
scl00231440.1_287-S	-27.38	-2.21	9130213B05Rik	NM_145562.1	Mus musculus RIKEN cDNA 9130213B05 gene (9130213B05Rik), mRNA.
scl00211586.2_45-S	-27.1989	-1.53	A330080J22Rik	NM_178667.2	Mus musculus RIKEN cDNA A330080J22 gene (A330080J22Rik), mRNA.
scl00170812.1_267-S	-27.1613	-1.42	Eraf	NM_133245.1	Mus musculus erythroid associated factor (Eraf), mRNA.
scl0107321.9_13-S	-27.141	-1.75	Lpxn	NM_134152.1	Mus musculus leupaxin (Lpxn), mRNA.
scl32758.9.1_2-S	-27.138	-2.66	Siglec10	NM_172900.1	Mus musculus sialic acid binding Ig-like lectin 10 (Siglec10), mRNA.
scl53360.9.1_13-S	-27.0389	-1.67	Ms4a6b	NM_027209.2	Mus musculus membrane-spanning 4-domains, subfamily A, member 6B (Ms4a6b), mRNA.
scl40288.3_192-S	-27.0012	-1.73	Ifi1	NM_008326.1	Mus musculus interferon inducible protein 1 (Ifi1), mRNA.
scl51713.6.3_46-S	-26.8767	-1.33	Cyb5	NM_025797	Mus musculus cytochrome b-5 (Cyb5), mRNA.
scl0054613.2_126-S	-26.8305	-1.35	Siat10	NM_018784.1	Mus musculus sialyltransferase 10 (alpha-2,3-sialyltransferase VI) (Siat10), mRNA.
scl019942.4_1-S	-26.7577	-1.71	Rpl27	NM_011289	Mus musculus ribosomal protein L27 (Rpl27), mRNA.
scl0067238.2_233-S	-26.7074	-1.71	2810453I06Rik	NM_026050.1	Mus musculus RIKEN cDNA 2810453I06 gene (2810453I06Rik), mRNA.
scl19885.7_41-S	-26.3751	-1.42	Zfp313	NM_030743.3	Mus musculus zinc finger protein 313 (Zfp313), mRNA.
scl0015040.1_90-S	-26.3333	-1.5	H2-T23	NM_010398.1	Mus musculus histocompatibility 2, T region locus 23 (H2-T23), mRNA.
scl0078255.2_178-S	-26.2823	-1.92	4921528G01Rik	NM_023884.2	
scl012484.2_55-S	-26.2312	-1.37	Cd24a	NM_009846.1	Mus musculus CD24a antigen (Cd24a), mRNA.
scl32303.22_382-S	-26.1408	-1.74	Fchsd2	NM_199012.1	Mus musculus FCH and double SH3 domains 2 (Fchsd2), mRNA.
scl013136.4_3-S	-26.103	-1.87	Daf1	NM_010016.1	Mus musculus decay accelerating factor 1 (Daf1), mRNA.
scl25758.6.1_14-S	-25.9765	-2.21	1200006F02Rik	NM_027872.1	Mus musculus RIKEN cDNA 1200006F02 gene (1200006F02Rik), mRNA.
scl069046.1_193-S	-25.9232	-1.31	Hbld2	NM_026921.2	Mus musculus HESB like domain containing 2 (Hbld2), mRNA.
scl0012192.2_103-S	-25.9148	-1.89	Zfp36l1	NM_007564.2	Mus musculus zinc finger protein 36, C3H type-like 1 (Zfp36l1), mRNA.
scl23773.12.1_52-S	-25.9137	-2.2	Lck	NM_010693.1	Mus musculus lymphocyte protein tyrosine kinase (Lck), mRNA.
scl0231086.16_205-S	-25.903	-1.28	Hadhb	NM_145558.1	Mus musculus hydroxyacyl-Coenzyme A dehydrogenase/3-ketoacyl-Coenzyme A thiolase/enoyl-Coenzyme A hydratase (trifunctional protein), beta subunit (Hadhb), mRNA.
scl0020947.1_79-S	-25.7666	-1.5	Swap70	NM_009302.2	Mus musculus SWAP complex protein (Swap70), mRNA.
scl32504.5.336_28-S	-25.725	-1.51	Isg20	NM_020583.4	Mus musculus interferon-stimulated protein (Isg20), mRNA.
scl076071.13_258-S	-25.6565	-2.38	Gababrbp	NM_178394.2	
scl0110052.3_51-S	-25.2779	-1.28	Dek	NM_025900.1	Mus musculus DEK oncogene (DNA binding) (Dek), mRNA.
scl35429.19.1_59-S	-25.2566	-1.4	1300017J02Rik	NM_027918.1	Mus musculus RIKEN cDNA 1300017J02 gene (1300017J02Rik), mRNA.
scl32299.7.1_25-S	-25.2329	-1.45	Stard10	NM_019990.1	Mus musculus START domain containing 10 (Stard10), mRNA.
scl068193.4_35-S	-25.1806	-1.31	Rpl24	NM_024218.2	Mus musculus ribosomal protein L24 (Rpl24), mRNA.
scl067596.7_211-S	-25.1446	-2.44	5830405N20Rik	NM_183264.1	Mus musculus RIKEN cDNA 5830405N20 gene (5830405N20Rik), mRNA.

scl0011307.2_72-S	-25.1033	-1.54	Abcg1	NM_009593.1	Mus musculus ATP-binding cassette, sub-family G (WHITE), member 1 (Abcg1), mRNA.
scl43315.9.1_17-S	-24.9377	-1.59	Sh3yl1	NM_013709.2	Mus musculus Sh3 domain YSC-like 1 (Sh3yl1), mRNA.
scl027556.1_96-S	-24.8068	-1.26	Clic4	XM_124389.1	Mus musculus chloride intracellular channel 4 (mitochondrial) (Clic4), mRNA.
scl50197.1.198_198-S	-24.6704	-1.3	Fahd1	NM_023480.1	
scl018606.1_11-S	-24.6452	-1.83	Enpp2	NM_015744	Mus musculus ectonucleotide pyrophosphatase/phosphodiesterase 2 (Enpp2), mRNA.
scl020335.2_33-S	-24.4364	-1.41	Sec61g	XM_122171.1	Mus musculus SEC61, gamma subunit (Sec61g), mRNA.
scl40219.8.1_51-S	-24.2646	-1.85	Pdlim4	NM_019417.1	Mus musculus PDZ and LIM domain 4 (Pdlim4), mRNA.
scl0014128.2_101-S	-24.24	-8.5	Fcer2a	NM_013517.1	Mus musculus Fc receptor, IgE, low affinity II, alpha polypeptide (Fcer2a), mRNA.
scl21235.24_380-S	-24.1835	-2.63	BC026657	NM_029895.3	
scl052840.5_2-S	-24.1384	-1.62	D2Bwg0891e	NM_026797.1	Mus musculus DNA segment, Chr 2, Brigham & Womens Genetics 0891 expressed (D2Bwg0891e), mRNA.
scl015000.6_157-S	-24.0775	-2.18	H2-DMb2	NM_010388	Mus musculus histocompatibility 2, class II, locus Mb2 (H2-DMb2), mRNA.
scl45535.5_307-S	-23.9962	-1.6	1810034K20Rik	NM_023397.3	Mus musculus RIKEN cDNA 1810034K20 gene (1810034K20Rik), mRNA.
scl46922.6_432-S	-23.9761	-2.14	Tnfrsf13c	NM_028075.1	Mus musculus tumor necrosis factor receptor superfamily, member 13c (Tnfrsf13c), mRNA.
scl023792.26_1-S	-23.9476	-2.85	Adam23	NM_011780.1	Mus musculus a disintegrin and metalloprotease domain 23 (Adam23), mRNA.
scl0019419.1_154-S	-23.9203	-2.5	Rasgrp1	NM_011246.2	Mus musculus RAS guanyl releasing protein 1 (Rasgrp1), mRNA.
scl52114.2_187-S	-23.8213	-2.23	Egr1	NM_007913.2	Mus musculus early growth response 1 (Egr1), mRNA.
scl0217151.1_189-S	-23.6141	-2.01	Arl12	NM_207231.1	
scl39161.6.1_317-S	-23.5671	-1.67	D730019B10Rik	NM_172785.1	
scl44899.5.1_87-S	-23.4784	-1.74	Ly86	NM_010745.1	Mus musculus lymphocyte antigen 86 (Ly86), mRNA.
scl0002648.1_0-S	-23.4385	-1.44	Mllt3	NM_027326.2	Mus musculus myeloid/lymphoid or mixed lineage-leukemia translocation to 3 homolog (Drosophila) (Mllt3), mRNA.
scl41727.3.1_77-S	-23.3725	-2.27	Hbq1	NM_175000.1	
scl013823.22_328-S	-23.3284	-1.77	Epb4.1l3	NM_013813.1	Mus musculus erythrocyte protein band 4.1-like 3 (Epb4.1l3), mRNA.
scl0093760.1_64-S	-23.2111	-1.85	Arid1a	NM_033566.1	Mus musculus AT rich interactive domain 1A (Swi1 like) (Arid1a), mRNA.
scl46686.16.1_15-S	-22.9872	-1.88	Itgb7	NM_013566.1	Mus musculus integrin beta 7 (Itgb7), mRNA.
scl45134.2_536-S	-22.9113	-2.13	Gpr18	NM_182806.1	Mus musculus G protein-coupled receptor 18 (Gpr18), mRNA.
scl018858.5_31-S	-22.8706	-1.86	Pmp22	NM_008885.1	Mus musculus peripheral myelin protein (Pmp22), mRNA.
scl0224703.1_41-S	-22.7836	-1.48	9530046H09Rik	NM_145486.2	Mus musculus RIKEN cDNA 9530046H09 gene (9530046H09Rik), mRNA.
IGKV14-126_AJ231238_I	-22.7286	-3.79	LOC243428	XM_144778.1	Mus musculus similar to Ig kappa chain (LOC243428), mRNA.
scl0015051.1_246-S	-22.5484	-1.68	H2-T9	NM_010399	Mus musculus histocompatibility 2, T region locus 9 (H2-T9), mRNA.
scl0066610.1_317-S	-22.3412	-1.83	Abi3	NM_025659.1	Mus musculus ABI gene family, member 3 (Abi3), mRNA.
scl48020.10.1_127-S	-22.3191	-2.37	2310016A09Rik	NM_181588.2	Mus musculus RIKEN cDNA 2310016A09 gene (2310016A09Rik), mRNA.
scl0029809.2_171-S	-22.3088	-1.47	Rabgap1l	NM_013862.3	
scl0240168.18_64-S	-22.2549	-2.15	Rasgrp3	NM_207246.2	Mus musculus RAS guanyl releasing protein 3 (Rasgrp3), mRNA.
IGLC2_J00595_Ig_lambd	-22.239	-2.05			
scl25458.10.1_3-S	-22.1334	-2.36	Galnt12	NM_172693.2	Mus musculus UDP-N-acetyl-alpha-D-galactosamine:polypeptide N-acetylgalactosaminyltransferase 12 (Galnt12), mRNA.
scl014468.10_101-S	-21.9766	-2.25	Gbp1	NM_010259.1	Mus musculus guanylate nucleotide binding protein 1 (Gbp1), mRNA.
scl0229499.10_22-S	-21.7004	-1.87	A230020G22Rik	NM_178165.2	Mus musculus RIKEN cDNA A230020G22 gene (A230020G22Rik), mRNA.
scl37046.4_393-S	-21.6435	-2	Thy1	NM_009382.2	Mus musculus thymus cell antigen 1, theta (Thy1), mRNA.
scl36445.9_185-S	-21.6291	-1.9	Scotin	NM_025858.1	Mus musculus scotin gene (Scotin), mRNA.
scl40428.15.1_30-S	-21.6272	-1.32	Vrk2	NM_027260.1	Mus musculus vaccinia related kinase 2 (Vrk2), mRNA.
scl45283.8.141_93-S	-21.576	-1.94	9030625A04Rik	NM_172488.1	Mus musculus RIKEN cDNA 9030625A04 gene (9030625A04Rik), mRNA.
scl019731.1_197-S	-21.5759	-1.8	Rgl1	NM_016846.2	Mus musculus ral guanine nucleotide dissociation stimulator,-like 1 (Rgl1), mRNA.
scl32592.2.658_11-S	-21.5571	-2	Ndn	NM_010882.2	Mus musculus necdin (Ndn), mRNA.
scl15925.5_61-S	-21.5192	-1.84	Pea15	NM_008556.1	Mus musculus phosphoprotein enriched in astrocytes 15 (Pea15), mRNA.
scl022142.1_236-S	-21.4566	-1.36	Tuba1	NM_011653	Mus musculus tubulin, alpha 1 (Tuba1), mRNA.
scl40832.13.1_18-S	-21.3502	-1.87	Myl4	NM_010858.3	Mus musculus myosin, light polypeptide 4, alkali; atrial, embryonic (Myl4), mRNA.
scl00228769.2_248-S	-21.3031	-1.25	Psmf1	NM_144889.1	Mus musculus proteasome (prosome, macropain) inhibitor subunit 1 (Psmf1), mRNA.
scl0013829.2_259-S	-21.298	-1.28	Epb4.9	NM_013514.2	Mus musculus erythrocyte protein band 4.9 (Epb4.9), mRNA.
scl16013.3.1_175-S	-21.1917	-3.3	Xcl1	NM_008510.1	Mus musculus chemokine (C motif) ligand 1 (Xcl1), mRNA.
scl29460.6.1_6-S	-21.1879	-2.81	Klrd1	NM_010654.1	Mus musculus killer cell lectin-like receptor, subfamily D, member 1 (Klrd1), mRNA.
scl31487.7.1_48-S	-21.1155	-1.35	6030457N17Rik	XM_133378.3	Mus musculus RIKEN cDNA 6030457N17 gene (6030457N17Rik), mRNA.
scl22076.2_386-S	-21.089	-1.33	Trim59	NM_025863.2	
scl36223.4.1187_49-S	-21.0659	-1.29	2310067E08Rik	NM_028013.1	Mus musculus RIKEN cDNA 2310067E08 gene (2310067E08Rik), mRNA.
scl34673.5.1_43-S	-21.0048	-1.34	Bst2	NM_198095.1	
scl015018.6_2-S	-20.9045	-2.49	H2-Q7	XM_359282.1	Mus musculus histocompatibility 2, Q region locus 7 (H2-Q7), mRNA.
scl016145.5_111-S	-20.8779	-1.47	Igtp	NM_018738.2	Mus musculus interferon gamma induced GTPase (Igtp), mRNA.

scl23434.8.1_51-S	-20.8197	-1.82	Mmp23	NM_011985.1	Mus musculus matrix metalloproteinase 23 (Mmp23), mRNA.
scl49439.24_604-S	-20.8184	-2.58	C2ta	NM_007575.1	Mus musculus class II transactivator (C2ta), mRNA.
scl070574.7_30-S	-20.725	-4.94	Cpm	XM_125830.3	
scl19453.6.2634_3-S	-20.6493	-1.52	A130092J06Rik	NM_175511.2	Mus musculus RIKEN cDNA A130092J06 gene (A130092J06Rik), mRNA.
scl012978.19_21-S	-20.6481	-1.69	Csf1r	NM_007779.1	Mus musculus colony stimulating factor 1 receptor (Csf1r), mRNA.
scl15839.11.1_64-S	-20.5368	-2.29	Ephx1	NM_010145.2	Mus musculus epoxide hydrolase 1, microsomal (Ephx1), mRNA.
scl16373.17.1_56-S	-20.472	-1.9	Marco	NM_010766.1	Mus musculus macrophage receptor with collagenous structure (Marco), mRNA.
scl38058.2.1_88-S	-20.454	-2.4	A630077B13Rik	NM_175449.3	Mus musculus RIKEN cDNA A630077B13 gene (A630077B13Rik), mRNA.
scl22910.4.1_66-S	-20.4438	-1.36	S100a10	NM_009112.1	Mus musculus S100 calcium binding protein A10 (calpactin) (S100a10), mRNA.
scl0110557.2_204-S	-20.4362	-2.22	H2-Q6	NM_207648	Mus musculus histocompatibility 2, Q region locus 6 (H2-Q6), mRNA.
scl0015950.1_6-S	-20.4302	-2.03	Ifi203	NM_008328.1	Mus musculus interferon activated gene 203 (Ifi203), mRNA.
scl28312.12.1_12-S	-20.4035	-1.32	Csda	NM_139117.1	Mus musculus cold shock domain protein A (Csda), mRNA.
scl39820.3.1_53-S	-20.3651	-3.25	Ccl5	NM_013653.1	Mus musculus chemokine (C-C motif) ligand 5 (Ccl5), mRNA.
scl00229622.1_168-S	-20.3117	-1.81	9430063L05Rik	NM_178080.3	Mus musculus RIKEN cDNA 9430063L05 gene (9430063L05Rik), mRNA.
scl0216792.1_314-S	-20.2032	-1.33	A230051G13Rik	NM_173785.2	
scl35577.16.1_50-S	-20.1813	-1.38	Fbxo9	NM_023605.1	Mus musculus f-box only protein 9 (Fbxo9), mRNA.
scl000996.1_9-S	-20.1553	-4.03	4921528G01Rik	NM_023884.2	Mus musculus RIKEN cDNA 4921528G01 gene (4921528G01Rik), mRNA.
scl53380.11_494-S	-20.1172	-1.24	Fads1	NM_146094.1	Mus musculus fatty acid desaturase 1 (Fads1), mRNA.
scl32195.13_319-S	-20.1025	-2.02	Swap70	NM_009302.2	Mus musculus SWAP complex protein (Swap70), mRNA.
scl19404.11_191-S	-20.0892	-2.85	Traf1	NM_009421.2	Mus musculus Tnf receptor-associated factor 1 (Traf1), mRNA.
scl0027371.2_277-S	-20.0407	-2.13	Sh2d2a	NM_021309.1	Mus musculus SH2 domain protein 2A (Sh2d2a), mRNA.

TABLE A10

GENMAPPS AND GENE ONTOLOGIES SIGNIFICANTLY CHANGED BY OPP IN SPLEENS

UP-REGULATED - GENMAPP

No.	MAPP Name	Number Changed	Number Measured	Number on MAPP	Percent Changed	Percent Present	Z Score	Permuted P Value
1	Mm_Glycolysis_Gluconeogenesis	9	42	61	21.4286	68.8525	6.7300	0.0000
2	Mm_Glycolysis_and_Gluconeogenesis	9	42	42	21.4286	100.0000	6.7300	0.0000
3	Mm_Carbon_fixation	5	16	29	31.2500	55.1724	6.3740	0.0000
4	Mm_Pentose_phosphate_pathway	6	24	47	25.0000	51.0638	6.0720	0.0000
5	Mm_Prostaglandin_synthesis_regulation	5	31	31	16.1290	100.0000	4.0950	0.0030

UP-REGULATED - GENE ONTOLOGY

No.	GO Name	GO Type	Number Changed	Number Measured	Number in GO	Percent Changed	Percent Present	Z Score	Permuted P Value
1	glycolysis	P	13	48	78	27.0833	61.5385	12.8650	0.0000
2	glucose metabolism	P	17	100	135	17.0000	74.0741	11.1590	0.0000
3	flotillin complex	C	2	2	2	100.0000	100.0000	10.2090	0.0000
4	phosphatidylinositol-4\,5-bisphosphate 3-kinase activity	F	2	3	3	66.6667	100.0000	8.2560	0.0000
5	phospholipase inhibitor activity	F	3	8	9	37.5000	88.8889	7.4130	0.0000
6	Arp2/3 protein complex	C	3	8	8	37.5000	100.0000	7.4130	0.0000
7	pentose-phosphate shunt	P	3	8	10	37.5000	80.0000	7.4130	0.0000
8	aldehyde dehydrogenase [NAD(P)+] activity	F	2	4	5	50.0000	80.0000	7.0810	0.0000
9	actin binding	F	17	207	230	8.2126	90.0000	6.7470	0.0000
10	carbohydrate metabolism	P	26	442	506	5.8824	87.3518	6.2800	0.0000
11	response to biotic stimulus	P	41	879	969	4.6644	90.7121	6.2550	0.0000
12	gluconeogenesis	P	4	20	20	20.0000	100.0000	5.9640	0.0000
13	response to pest\, pathogen or parasite	P	27	502	553	5.3785	90.7776	5.8620	0.0000
14	glutathione metabolism	P	3	15	17	20.0000	88.2353	5.1650	0.0000
15	immune response	P	32	731	793	4.3776	92.1816	5.0890	0.0000
16	response to stress	P	39	1014	1107	3.8462	91.5989	4.7650	0.0000
17	actin cytoskeleton	C	13	206	229	6.3107	89.9563	4.7080	0.0000
18	defense response	P	33	828	915	3.9855	90.4918	4.5810	0.0000
19	response to stimulus	P	65	2074	2329	3.1340	89.0511	4.5210	0.0000
20	cytoskeleton	C	28	743	859	3.7685	86.4959	3.8800	0.0000
21	catalytic activity	F	117	4696	5420	2.4915	86.6421	3.7140	0.0000
22	oxidoreductase activity	F	25	663	775	3.7707	85.5484	3.6590	0.0000
23	glyceraldehyde-3-phosphate dehydrogenase (phosphorylating) act.	F	3	6	26	50.0000	23.0769	8.6720	0.0010
24	blood coagulation	P	6	73	74	8.2192	98.6487	3.9920	0.0010
25	inflammatory response	P	12	217	230	5.5300	94.3478	3.9810	0.0010
26	chemotaxis	P	8	117	124	6.8376	94.3548	3.9580	0.0010
27	cytoplasm	C	87	3488	3969	2.4943	87.8811	3.0390	0.0010
28	actin filament polymerization	P	4	25	27	16.0000	92.5926	5.1970	0.0020
29	phospholipase A2 inhibitor activity	F	2	3	3	66.6667	100.0000	8.2560	0.0030
30	epoxide hydrolase activity	F	2	6	6	33.3333	100.0000	5.6680	0.0030
31	acute-phase response	P	4	23	26	17.3913	88.4615	5.4760	0.0030
32	hematopoietin/interferon-class (D200-domain) cytokine receptor act.	F	5	58	60	8.6207	96.6667	3.7820	0.0040
33	cytoskeleton organization and biogenesis	P	17	386	431	4.4041	89.5592	3.6920	0.0040
34	collagen catabolism	P	3	16	17	18.7500	94.1177	4.9660	0.0050
35	cell proliferation	P	22	611	648	3.6007	94.2901	3.1900	0.0050
36	actin cytoskeleton organization and biogenesis	P	9	163	171	5.5215	95.3216	3.4360	0.0060
37	kinase activity	F	25	788	887	3.1726	88.8388	2.7370	0.0060
38	pepsin A activity	F	2	7	9	28.5714	77.7778	5.1960	0.0070
39	lysosomal membrane	C	2	9	10	22.2222	90.0000	4.4900	0.0070
40	glutathione transferase activity	F	3	21	26	14.2857	80.7692	4.1840	0.0090
41	peptidoglycan metabolism	P	3	25	26	12.0000	96.1539	3.7240	0.0090
42	transferase activity	F	43	1561	1757	2.7546	88.8446	2.6790	0.0090

DOWN-REGULATED - GENMAPP

No.	MAPP Name	Number Changed	Number Measured	Number on MAPP	Percent Changed	Percent Present	Z Score	Permuted P Value
1	Mm_Heme_Biosynthesis	3	9	9	33.3333	100.0000	7.1190	0.0000
2	Mm_2-Tissues-Blood_and_Lymph	5	34	40	14.7059	85.0000	5.6790	0.0000
3	Mm_B_Cell_Receptor_NetPath_12	10	147	150	6.8027	98.0000	4.6380	0.0000
4	Mm_Porphyrin_and_chlorophyll_metabolism	3	15	58	20.0000	25.8621	5.3080	0.0010
5	Mm_T-Cell-Receptor_NetPath_11	7	124	126	5.6452	98.4127	3.2650	0.0080

DOWN-REGULATED - GENE ONTOLOGY

No.	GO Name	GO Type	Number Changed	Number Measured	Number in GO	Percent Changed	Percent Present	Z Score	Permuted P Value
1	antigen presentation\, exogenous peptide antigen	P	5	5	6	100.0000	83.3333	18.4830	0.0000
2	antigen processing\, exogenous antigen via MHC class II	P	6	9	13	66.6667	69.2308	16.4130	0.0000
3	antigen presentation\, exogenous antigen via MHC class II	P	6	10	12	60.0000	83.3333	15.5330	0.0000
4	MHC class II receptor activity	F	5	7	11	71.4286	63.6364	15.5300	0.0000
5	external side of plasma membrane	C	15	76	80	19.7368	95.0000	13.4080	0.0000
6	defense response	P	56	828	915	6.7633	90.4918	13.2130	0.0000
7	immune response	P	51	731	793	6.9767	92.1816	12.8690	0.0000
8	multivesicular body	C	4	7	10	57.1429	70.0000	12.3600	0.0000
9	antigen presentation	P	11	52	63	21.1539	82.5397	11.9400	0.0000
10	antigen presentation\, exogenous antigen	P	6	17	21	35.2941	80.9524	11.7100	0.0000
11	biliverdin reductase activity	F	2	2	2	100.0000	100.0000	11.6880	0.0000
12	MHC class II protein complex	C	2	2	2	100.0000	100.0000	11.6880	0.0000
13	antigen presentation\, endogenous antigen	P	6	22	25	27.2727	88.0000	10.1670	0.0000
14	cell surface	C	17	157	164	10.8280	95.7317	9.9140	0.0000

15	humoral immune response	P	15	134	142	11.1940	94.3662	9.5080	0.0000
16	positive regulation of T cell activation	P	8	45	45	17.7778	100.0000	9.2030	0.0000
17	T cell activation	P	12	95	101	12.6316	94.0594	9.1740	0.0000
18	antigen processing\, endogenous antigen via MHC class I	P	5	20	23	25.0000	86.9565	8.8400	0.0000
19	response to stimulus	P	74	2074	2329	3.5680	89.0511	8.7540	0.0000
20	MHC class I protein complex	C	5	25	32	20.0000	78.1250	7.7870	0.0000
21	T cell differentiation	P	6	38	41	15.7895	92.6829	7.4250	0.0000
22	MHC class I receptor activity	F	5	28	35	17.8571	80.0000	7.2900	0.0000
23	detection of pest\, pathogen or parasite	P	2	5	5	40.0000	100.0000	7.2310	0.0000
24	cellular defense response	P	9	101	103	8.9109	98.0583	6.3150	0.0000
25	positive regulation of immune response	P	8	91	95	8.7912	95.7895	5.8960	0.0000
26	plasma membrane	C	48	1634	1760	2.9376	92.8409	5.3720	0.0000
27	hemopoiesis	P	10	182	201	5.4945	90.5473	4.6120	0.0000
28	protein binding	F	94	4725	5185	1.9894	91.1283	3.8200	0.0000
29	cytoplasm	C	70	3488	3969	2.0069	87.8811	3.1960	0.0000
30	positive regulation of T cell differentiation	P	4	17	17	23.5294	100.0000	7.6410	0.0010
31	heme biosynthesis	P	3	11	11	27.2727	100.0000	7.1860	0.0010
32	integrin binding	F	5	32	34	15.6250	94.1177	6.7350	0.0010
33	sialyltransferase activity	F	3	18	19	16.6667	94.7368	5.4190	0.0010
34	antigen binding	F	4	34	63	11.7647	53.9683	5.0530	0.0010
35	humoral defense mechanism (sensu Vertebrata)	P	7	93	101	7.5269	92.0792	4.9350	0.0010
36	chemokine activity	F	4	37	43	10.8108	86.0465	4.7840	0.0010
37	copper ion binding	F	5	57	64	8.7719	89.0625	4.6490	0.0010
38	positive thymic T cell selection	P	3	7	7	42.8571	100.0000	9.1900	0.0020
39	B cell receptor complex	C	2	4	4	50.0000	100.0000	8.1450	0.0020
40	chaperone cofactor dependent protein folding	P	2	9	10	22.2222	90.0000	5.2290	0.0020
41	lysosome	C	7	123	129	5.6911	95.3488	3.9670	0.0020
42	apoptosis	P	18	573	617	3.1414	92.8687	3.4770	0.0030
43	calcium ion homeostasis	P	5	82	84	6.0976	97.6191	3.5440	0.0050
44	B cell receptor signaling pathway	P	2	7	7	28.5714	100.0000	6.0200	0.0060
45	regulation of apoptosis	P	14	403	431	3.4739	93.5035	3.4670	0.0060
46	sugar binding	F	7	173	187	4.0462	92.5134	2.8880	0.0060
47	cell proliferation	P	17	611	648	2.7823	94.2901	2.8360	0.0060
48	Ras guanyl-nucleotide exchange factor activity	F	2	9	9	22.2222	100.0000	5.2290	0.0070
49	protein-lysine 6-oxidase activity	F	2	10	11	20.0000	90.9091	4.9220	0.0070
50	integral to Golgi membrane	C	3	30	32	10.0000	93.7500	3.9340	0.0080
51	endonuclease activity	F	5	80	121	6.2500	66.1157	3.6150	0.0080
52	lipid raft	C	3	27	29	11.1111	93.1035	4.2160	0.0090

TABLE A11					
GENES UP-REGULATED BY OPP IN HEARTS					
TargetID	Diff_Score	Fold_Change	Symbol	Accession	Definition
scl48649.5.1_0-S	34.9301	1.4	5730578N08Rik	XM_148395.1	Mus musculus RIKEN cDNA 5730578N08 gene (5730578N08Rik), mRNA.
scl0380791.1_239-S	32.7668	2.55	Igh-VJ558	XM_354700	
scl0003638.1_65-S	32.7229	1.45	2310016C16Rik	NM_027127.1	Mus musculus RIKEN cDNA 2310016C16 gene (2310016C16Rik), mRNA.
scl019989.6_6-S	32.2873	1.23	Rpl7	NM_011291	Mus musculus ribosomal protein L7 (Rpl7), mRNA.
scl34621.8_577-S	31.8495	1.29	Ednra	NM_010332.1	Mus musculus endothelin receptor type A (Ednra), mRNA.
scl41548.2.100_36-S	29.3828	1.21	Hint1	NM_008248.1	Mus musculus histidine triad nucleotide binding protein 1 (Hint1), mRNA.
scl24151.12.434_2-S	29.1369	1.36	Adfp	NM_007408.2	Mus musculus adipose differentiation related protein (Adfp), mRNA.
scl066576.4_1-S	28.9736	1.2	Uqcrh	NM_025641.2	
scl41049.3_319-S	27.8967	1.66	1810057C19Rik	NM_026433.1	Mus musculus RIKEN cDNA 1810057C19 gene (1810057C19Rik), mRNA.
scl50814.22.1_1-S	27.1031	1.33	Crebl1	NM_017406.2	Mus musculus cAMP responsive element binding protein-like 1 (Crebl1), mRNA.
scl28291.1_56-S	26.9646	1.24	Tctex1	NM_009342	Mus musculus t-complex testis expressed 1 (Tctex1), mRNA.
scl47818.1_22-S	23.1615	1.4	Gpihbp1	XM_128001.2	
scl0229543.1_170-S	23.1057	1.49	C77668	NM_145540.2	Mus musculus expressed sequence C77668 (C77668), transcript variant 1, mRNA.
scl0330941.1_271-S	23.0508	1.67	AI593442	NM_177907.2	Mus musculus expressed sequence AI593442 (AI593442), mRNA.
scl018139.26_3-S	22.9847	1.27	Zfml	NM_008717.1	
scl51416.9_257-S	21.7381	1.85	Lox	NM_010728.1	Mus musculus lysyl oxidase (Lox), mRNA.
scl096875.3_23-S	21.7114	2.63	Prg4	XM_355243.1	
scl23237.22_584-S	21.1741	1.35	3110057O12Rik	NM_026622.1	Mus musculus RIKEN cDNA 3110057O12 gene (3110057O12Rik), mRNA.
scl0002332.1_7-S	20.5382	1.17	Dld	NM_007861	Mus musculus dihydrolipoamide dehydrogenase (Dld), mRNA.
scl47073.5.231_36-S	19.9005	1.41	Ly6c	NM_010741	Mus musculus lymphocyte antigen 6 complex, locus C (Ly6c), mRNA.
scl54930.9.1_1-S	19.6182	1.2	Hprt	NM_013556	
scl33493.3.1_90-S	19.424	2.26	Mt2	NM_008630.1	Mus musculus metallothionein 2 (Mt2), mRNA.
scl067267.4_0-S	19.2236	1.16	2900010M23Rik	NM_026063.1	Mus musculus RIKEN cDNA 2900010M23 gene (2900010M23Rik), mRNA.
scl020393.12_71-S	18.9391	1.35	Sgk	NM_011361.1	Mus musculus serum/glucocorticoid regulated kinase (Sgk), mRNA.
scl0319186.1_75-S	18.1757	1.23	Hist1h2bm	NM_178200	Mus musculus histone 1, H2bm (Hist1h2bm), mRNA.
scl44868.8_25-S	18.1666	1.38	Gcnt2	NM_008105.2	Mus musculus glucosaminyl (N-acetyl) transferase 2, I-branching enzyme (Gcnt2), transcript variant 3, mRNA.
scl20807.21.1_18-S	18.0519	1.26	Dncic2	NM_010064	Mus musculus dynein, cytoplasmic, intermediate chain 2 (Dncic2), mRNA.
scl50283.7.5_2-S	18.0507	1.24	Psmb1	NM_011185	Mus musculus proteasome (prosome, macropain) subunit, beta type 1 (Psmb1), mRNA.
scl0016061.1_157-S	17.9757	2.58	Igh-VJ558	XM_354700	
scl0234734.18_28-S	17.7371	1.29	Aars	NM_146217.3	Mus musculus alanyl-tRNA synthetase (Aars), mRNA.
scl0004083.1_182-S	17.7268	1.31	Cdv1	NM_009879.2	Mus musculus carnitine deficiency-associated gene expressed in ventricle 1 (Cdv1), mRNA.
scl36472.2.242_20-S	17.5393	1.24	Gpx1	NM_008160.1	Mus musculus glutathione peroxidase 1 (Gpx1), mRNA.
scl53358.2.1_26-S	17.5118	1.6	Ms4a6c	NM_028595	Mus musculus membrane-spanning 4-domains, subfamily A, member 6C (Ms4a6c), mRNA.
scl0319160.1_25-S	17.4277	1.31	Hist1h4k	NM_178211.1	Mus musculus histone 1, H4k (Hist1h4k), mRNA.
scl15961.15.1_3-S	17.3311	1.5	Uap1	NM_133806.2	Mus musculus UDP-N-acetylglucosamine pyrophosphorylase 1 (Uap1), mRNA.
scl49164.12_81-S	17.2831	1.72	Fstl1	NM_008047.2	Mus musculus follistatin-like 1 (Fstl1), mRNA.
scl36315.5_684-S	17.2196	2.34	Ccbp2	NM_021609.2	Mus musculus chemokine binding protein 2 (Ccbp2), mRNA.
scl0230514.5_86-S	16.9687	1.25	Obrgrp	NM_175036	Mus musculus leptin receptor gene-related protein (Obrgrp), mRNA.
scl29411.4.6_15-S	16.9569	1.71	Mgst1	NM_019946.3	Mus musculus microsomal glutathione S-transferase 1 (Mgst1), mRNA.
scl0319185.1_5-S	16.8637	1.26	Hist1h2bl	NM_178199	Mus musculus histone 1, H2bl (Hist1h2bl), mRNA.
scl0319187.1_0-S	16.6955	1.24	Hist1h2bn	NM_178201.1	Mus musculus histone 1, H2bn (Hist1h2bn), mRNA.
scl55015.6.1_13-S	16.6654	2.01	Timp1	NM_011593	Mus musculus tissue inhibitor of metalloproteinase 1 (Timp1), mRNA.
scl0004022.1_70-S	16.4234	1.44	Arpc1b	NM_023142.1	Mus musculus actin related protein 2/3 complex, subunit 1B (Arpc1b), mRNA.
scl000253.1_401-S	16.3195	1.22	Ctsc	NM_009982.2	Mus musculus cathepsin C (Ctsc), mRNA.
scl0003537.1_10-S	16.3161	2.01	Pcolce2	NM_029620.1	Mus musculus procollagen C-endopeptidase enhancer 2 (Pcolce2), mRNA.
scl0002126.1_21-S	16.2872	1.41	1110001A05Rik	NM_022554.1	Mus musculus RIKEN cDNA 1110001A05 gene (1110001A05Rik), mRNA.
scl024051.1_222-S	16.232	1.2	Sgcb	NM_011890.2	Mus musculus sarcoglycan, beta (dystrophin-associated glycoprotein) (Sgcb), mRNA.
scl066272.3_26-S	15.9703	1.24	1810020G14Rik	NM_025461.2	Mus musculus RIKEN cDNA 1810020G14 gene (1810020G14Rik), mRNA.
scl0012540.1_20-S	15.9437	1.2	Cdc42	NM_009861.1	Mus musculus cell division cycle 42 homolog (S. cerevisiae) (Cdc42), mRNA.
scl19390.3.3_3-S	15.7125	1.21	Ndufa8	NM_026703.1	Mus musculus NADH dehydrogenase (ubiquinone) 1 alpha subcomplex, 8 (Ndufa8), mRNA.
scl34564.9_192-S	15.6097	1.28	4930527D15Rik	NM_026350.1	Mus musculus RIKEN cDNA 4930527D15 gene (4930527D15Rik), mRNA.
scl000941.1_0-S	15.2469	1.17	Ndufs2	NM_153064.3	Mus musculus NADH dehydrogenase (ubiquinone) Fe-S protein 2 (Ndufs2), mRNA.
scl33301.5.15_0-S	15.0657	1.21	Nudt7	NM_024437	Mus musculus nudix (nucleoside diphosphate linked moiety X)-type motif 7 (Nudt7), mRNA.

scl017110.1_293-S	14.9216	1.68	Lzp-s	NM_013590.2	Mus musculus P lysozyme structural (Lzp-s), mRNA.
scl43495.3.1_6-S	14.9107	1.2	2310016C16Rik	NM_027127.1	Mus musculus RIKEN cDNA 2310016C16 gene (2310016C16Rik), mRNA.
scl31620.20_1-S	14.8757	1.37	Axl	NM_009465.2	Mus musculus AXL receptor tyrosine kinase (Axl), mRNA.
scl20107.1_2-S	14.638	1.22	Idb1	NM_010495.1	Mus musculus inhibitor of DNA binding 1 (Idb1), mRNA.
scl43090.8_531-S	14.5466	1.18	Rhoj	NM_023275.1	Mus musculus ras homolog gene family, member J (Rhoj), mRNA.
scl020716.5_261-S	14.3786	2.35	Serpina3n	NM_009252.1	Mus musculus serine (or cysteine) proteinase inhibitor, clade A, member 3N (Serpina3n), mRNA.
scl021937.10_167-S	14.3631	1.38	Tnfrsf1a	NM_011609.2	Mus musculus tumor necrosis factor receptor superfamily, member 1a (Tnfrsf1a), mRNA.
scl0019186.1_187-S	14.2782	1.16	Psme1	NM_011189.1	Mus musculus proteasome (prosome, macropain) 28 subunit, alpha (Psme1), mRNA.
scl0319180.1_4-S	14.21	1.32	Hist1h2bf	NM_178195.1	Mus musculus histone 1, H2bf (Hist1h2bf), mRNA.
scl28399.6.24_1-S	14.2029	1.31	Cd9	NM_007657.2	Mus musculus CD9 antigen (Cd9), mRNA.
scl29544.11_278-S	14.0948	2.05	Mfap5	NM_015776	Mus musculus microfibrillar associated protein 5 (Mfap5), mRNA.
scl49357.1.362_28-S	13.9738	1.39	Cldn5	XM_147222.1	Mus musculus claudin 5 (Cldn5), mRNA.
scl0002748.1_8-S	13.9724	1.17	1110060F11Rik	NM_026395.1	Mus musculus RIKEN cDNA 1110060F11 gene (1110060F11Rik), mRNA.
scl48644.15_23-S	13.963	1.42	C330012H03Rik	NM_183029.1	Mus musculus RIKEN cDNA C330012H03 gene (C330012H03Rik), mRNA.
scl00216551.2_0-S	13.9194	1.3	1110067D22Rik	NM_173752.1	Mus musculus RIKEN cDNA 1110067D22 gene (1110067D22Rik), mRNA.
scl54154.5.1_20-S	13.6671	1.32	Idh3g	NM_008323.1	Mus musculus isocitrate dehydrogenase 3 (NAD+), gamma (Idh3g), mRNA.
scl28068.2_468-S	13.5111	1.15	Fgl2	NM_008013.2	Mus musculus fibrinogen-like protein 2 (Fgl2), mRNA.
scl24620.16_177-S	13.4364	1.16	BC004012	NM_138671	Mus musculus cDNA sequence BC004012 (BC004012), mRNA.
scl27109.17.1_35-S	13.4106	1.18	Plod3	NM_011962.2	Mus musculus procollagen-lysine, 2-oxoglutarate 5-dioxygenase 3 (Plod3), mRNA.
scl34999.9.1_89-S	13.3591	2.5	B430206E18Rik	XM_284398.1	
scl014130.1_14-S	13.23	1.85	Fcgr2b	NM_010187.1	Mus musculus Fc receptor, IgG, low affinity IIb (Fcgr2b), mRNA.
scl43661.2_29-S	13.1758	1.14	F2r	NM_010169.2	Mus musculus coagulation factor II (thrombin) receptor (F2r), mRNA.
scl020719.1_146-S	13.1742	1.17	Serpinb6a	NM_009254.2	Mus musculus serine (or cysteine) proteinase inhibitor, clade B, member 6a (Serpinb6a), mRNA.
scl00104681.2_288-S	13.1543	1.55	Slc16a6	NM_134038.1	Mus musculus solute carrier family 16 (monocarboxylic acid transporters), member 6 (Slc16a6), mRNA.
scl36984.16.1_60-S	13.1507	1.6	Zw10	NM_012039.1	Mus musculus ZW10 homolog (Drosophila), centromere/kinetochore protein (Zw10), mRNA.
scl23105.14.1_23-S	13.0247	1.27	Schip1	NM_013928.2	Mus musculus schwannomin interacting protein 1 (Schip1), mRNA.
**GENES DOWN-REGULATED BY OPP IN HEARTS**					
**TargetID**	**Diff_Score**	**Fold_Change**	**Symbol**	**Accession**	**Definition**
scl0003447.1_0-S	-36.1303	-1.83	Rbm5	NM_148930.2	Mus musculus RNA binding motif protein 5 (Rbm5), mRNA.
scl19105.6_353-S	-29.8797	-1.22	Ttn	XM_130312.3	Mus musculus titin (Ttn), mRNA.
scl34581.9.1_48-S	-26.6875	-1.25	Gpsn2	NM_134118.1	Mus musculus glycoprotein, synaptic 2 (Gpsn2), mRNA.
scl30345.6_391-S	-23.3518	-1.47	Kcnd2	XM_132981.1	Mus musculus potassium voltage-gated channel, Shal-related family, member 2 (Kcnd2), mRNA.
scl0076895.2_316-S	-22.6678	-1.54	Bicd2	NM_029791.2	Mus musculus bicaudal D homolog 2 (Drosophila) (Bicd2), mRNA.
scl0020973.1_244-S	-22.6397	-1.23	Syngr2	NM_009304.1	Mus musculus synaptogyrin 2 (Syngr2), mRNA.
scl36105.10.25_13-S	-21.7663	-1.51	Tbx20	NM_194263.1	Mus musculus T-box 20 (Tbx20), transcript variant 2, mRNA.
scl0020455.1_36-S	-21.581	-1.21	Sif1		
scl27246.12.1_228-S	-21.5003	-1.34	BC035291	NM_177581.2	Mus musculus cDNA sequence BC035291 (BC035291), mRNA.
scl40597.27.1_84-S	-21.3587	-1.33	Smtn	NM_013870.1	Mus musculus smoothelin (Smtn), mRNA.
scl37421.7_120-S	-20.9459	-1.27	Rassf3	NM_138956	Mus musculus Ras association (RalGDS/AF-6) domain family 3 (Rassf3), mRNA.
scl0022070.1_31-S	-20.3442	-1.27	Tpt1	NM_009429	Mus musculus tumor protein, translationally-controlled 1 (Tpt1), mRNA.
scl42532.6_0-S	-20.1914	-1.58	Tm4sf13	NM_025359.2	Mus musculus transmembrane 4 superfamily member 13 (Tm4sf13), mRNA.
scl0017765.2_254-S	-18.8391	-1.38	Mtf2	NM_013827.1	Mus musculus metal response element binding transcription factor 2 (Mtf2), mRNA.
scl54562.12.1_64-S	-18.6104	-1.59	Alas2	NM_009653.1	Mus musculus aminolevulinic acid synthase 2, erythroid (Alas2), mRNA.
scl27262.6_658-S	-18.5102	-1.18	9130017A15Rik	NM_177242.3	Mus musculus RIKEN cDNA 9130017A15 gene (9130017A15Rik), mRNA.
scl0003527.1_242-S	-17.9742	-1.3	Ddx6	NM_007841.2	Mus musculus DEAD (Asp-Glu-Ala-Asp) box polypeptide 6 (Ddx6), mRNA.
scl17563.43_11-S	-17.6422	-1.17	Clasp1		Mus musculus CLIP associating protein 1 (Clasp1), mRNA.
scl0319476.2_26-S	-17.1425	-1.25	A930016D02Rik	NM_176920.2	Mus musculus RIKEN cDNA A930016D02 gene (A930016D02Rik), mRNA.
scl21522.25_9-S	-16.9949	-1.71	Egf	NM_010113.1	Mus musculus epidermal growth factor (Egf), mRNA.
scl067371.1_2-S	-16.6093	-1.47	2410016F19Rik	NM_026113.2	Mus musculus RIKEN cDNA 2410016F19 gene (2410016F19Rik), mRNA.
scl0224807.4_25-S	-16.4982	-1.56	BC026370	NM_198167.1	Mus musculus cDNA sequence BC026370 (BC026370), mRNA.
scl32857.8.1_60-S	-16.3244	-1.3	Lgals4	NM_010706.1	Mus musculus lectin, galactose binding, soluble 4 (Lgals4), mRNA.
scl17047.9.4_6-S	-16.0154	-1.33	BC013667	NM_172266.1	Mus musculus cDNA sequence BC013667 (BC013667), mRNA.
scl20141.21_29-S	-15.7759	-1.16	Pygb	NM_153781.1	Mus musculus brain glycogen phosphorylase (Pygb), mRNA.
scl0012725.1_238-S	-15.6553	-1.22	Clcn3		
scl26992.46_0-S	-15.4646	-1.31	AI481500	NM_133901.1	Mus musculus expressed sequence AI481500 (AI481500), mRNA.
scl32353.2.4_40-S	-15.406	-1.28	Ndufc2	NM_024220.1	

scl24818.5.937_3-S	-15.0896	-1.2	Zfp46	NM_009557.1	Mus musculus zinc finger protein 46 (Zfp46), mRNA.
scl48020.10.1_127-S	-15.0502	-1.2	2310016A09Rik	NM_181588.2	Mus musculus RIKEN cDNA 2310016A09 gene (2310016A09Rik), mRNA.
scl067956.13_1-S	-14.9232	-1.28	2410195B05Rik	NM_030241.2	Mus musculus RIKEN cDNA 2410195B05 gene (2410195B05Rik), mRNA.
scl0012192.2_103-S	-14.8896	-1.72	Zfp36l1	NM_007564.2	Mus musculus zinc finger protein 36, C3H type-like 1 (Zfp36l1), mRNA.
scl0001100.1_114-S	-14.8831	-1.2	Aqp1	NM_007472.1	Mus musculus aquaporin 1 (Aqp1), mRNA.
scl020382.2_66-S	-14.8741	-1.19	Sfrs2	NM_011358.1	Mus musculus splicing factor, arginine/serine-rich 2 (SC-35) (Sfrs2), mRNA.
scl39616.9.169_52-S	-14.8047	-1.58	Nr1d1	NM_145434.1	Mus musculus nuclear receptor subfamily 1, group D, member 1 (Nr1d1), mRNA.
scl28635.3.1_18-S	-14.5374	-1.31	Lmod3	XM_149772.3	
scl41201.7.1_21-S	-14.4234	-1.67	Vtn	NM_011707.1	Mus musculus vitronectin (Vtn), mRNA.
scl52798.4.1_101-S	-14.2845	-1.23	Nudt8	NM_025529.2	Mus musculus nudix (nucleoside diphosphate linked moiety X)-type motif 8 (Nudt8), mRNA.
scl50134.9_8-S	-14.1258	-1.2	Nudt3	NM_019837.1	Mus musculus nudix (nucleotide diphosphate linked moiety X)-type motif 3 (Nudt3), mRNA.
scl22977.3.91_193-S	-14.019	-1.27	Dpm3	XM_130951.1	Mus musculus dolichyl-phosphate mannosyltransferase polypeptide 3 (Dpm3), mRNA.
scl31603.36.1_19-S	-13.9861	-1.3	Ltbp4	NM_175641.1	Mus musculus latent transforming growth factor beta binding protein 4 (Ltbp4), mRNA.
scl34280.9.1_28-S	-13.979	-1.24	2310061C15Rik	NM_026844.2	
scl24691.6_256-S	-13.9773	-1.29	Catnbip1	NM_023465.2	Mus musculus catenin beta interacting protein 1 (Catnbip1), mRNA.
scl0015516.2_121-S	-13.908	-1.29	Hspcb	NM_008302.2	Mus musculus heat shock protein 1, beta (Hspcb), mRNA.
scl0104923.5_14-S	-13.8104	-1.23	AL024210	NM_134052.1	Mus musculus expressed sequence AL024210 (AL024210), mRNA.
scl40333.9_365-S	-13.8014	-1.34	Mat2b	NM_134017.1	Mus musculus methionine adenosyltransferase II, beta (Mat2b), mRNA.
scl0121022.3_149-S	-13.7043	-1.26	Mrps6	NM_080456.1	Mus musculus mitochondrial ribosomal protein S6 (Mrps6), mRNA.
scl54266.5_48-S	-13.5523	-1.18	Rap2c	NM_172413.1	Mus musculus RAP2C, member of RAS oncogene family (Rap2c), mRNA.
scl21064.31_2-S	-13.412	-1.18	5830434P21Rik	NM_172661.2	Mus musculus RIKEN cDNA 5830434P21 gene (5830434P21Rik), mRNA.
scl0057259.2_60-S	-13.2331	-1.52	Tob2	NM_020507.2	Mus musculus transducer of ERBB2, 2 (Tob2), mRNA.
scl0066881.2_144-S	-13.1102	-1.24	Pcyox1	NM_025823.3	Mus musculus prenylcysteine oxidase 1 (Pcyox1), mRNA.
scl068728.7_0-S	-13.0323	-1.14	Trp53inp2	NM_178111.2	
scl30455.5_27-S	-13.0113	-1.37	Cdkn1c	NM_009876.2	Mus musculus cyclin-dependent kinase inhibitor 1C (P57) (Cdkn1c), mRNA.

TABLE A12

GENMAPPS AND GENE ONTOLOGIES SIGNIFICANTLY CHANGED BY OPP IN HEARTS

UP-REGULATED - GENMAPP

No.	MAPP Name	Number Changed	Number Measured	Number on MAPP	Percent Changed	Percent Present	Z Score	Permuted P Value
1	Mm_Citrate_cycle_TCA_cycle_	2	14	27	14.2857	51.8519	5.5520	0.0060
2	Mm_Oxidative_Stress	2	25	28	8.0000	89.2857	3.9580	0.0140
3	Mm_Krebs-TCA_Cycle	2	29	29	6.8966	100.0000	3.6090	0.0240
4	Mm_Prostaglandin_synthesis_regulation	2	31	31	6.4516	100.0000	3.4580	0.0370
5	Mm_IL-3_NetPath_15	3	95	96	3.1579	98.9583	2.5250	0.0430

UP-REGULATED - GENE ONTOLOGY

No.	GO Name	GO Type	Number Changed	Number Measured	Number in GO	Percent Changed	Percent Present	Z Score	Permuted P Value
1	cytolysis	P	3	19	20	15.7895	95.0000	9.5800	0.0000
2	glutathione peroxidase activity	F	2	9	11	22.2222	81.8182	9.3620	0.0000
3	cytoplasm	C	29	3488	3969	0.8314	87.8811	3.3340	0.0010
4	NADH dehydrogenase (ubiquinone) activity	F	2	27	39	7.4074	69.2308	5.1660	0.0020
5	nucleosome assembly	P	3	66	128	4.5455	51.5625	4.7420	0.0020
6	nucleosome	C	3	56	115	5.3571	48.6957	5.2400	0.0030
7	NADH dehydrogenase activity	F	2	31	43	6.4516	72.0930	4.7710	0.0040
8	external side of plasma membrane	C	3	76	80	3.9474	95.0000	4.3400	0.0040
9	oxidoreductase activity	F	9	663	775	1.3575	85.5484	3.2890	0.0040
10	extracellular region	C	19	2219	2362	0.8562	93.9458	2.7030	0.0050
11	Rho protein signal transduction	P	3	83	90	3.6145	92.2222	4.1000	0.0060
12	chromosome	C	5	280	364	1.7857	76.9231	3.1480	0.0150
13	mitochondrion	C	8	730	822	1.0959	88.8078	2.4190	0.0240
14	positive regulation of I-kappaB kinase/NF-kappaB cascade	P	2	56	61	3.5714	91.8033	3.3190	0.0290
15	plasma membrane	C	14	1634	1760	0.8568	92.8409	2.2710	0.0290
16	extracellular space	C	16	1967	2052	0.8134	95.8577	2.2270	0.0310
17	extracellular matrix (sensu Metazoa)	C	4	256	273	1.5625	93.7729	2.4900	0.0330
18	heparin binding	F	2	55	56	3.6364	98.2143	3.3580	0.0350
19	microtubule	C	3	144	166	2.0833	86.7470	2.7620	0.0400

DOWN-REGULATED - GENMAPP

No.	MAPP Name	Number Changed	Number Measured	Number on MAPP	Percent Changed	Percent Present	Z Score	Permuted P Value
1	Mm_Id_NetPath_5	2	49	50	4.0816	98.0000	5.0940	0.0040
2	Mm_Delta-Notch_NetPath_3	2	78	80	2.5641	97.5000	3.8770	0.0130
3	Mm_Wnt_NetPath_8	2	106	109	1.8868	97.2477	3.1910	0.0310

DOWN-REGULATED - GENE ONTOLOGY

No.	GO Name	GO Type	Number Changed	Number Measured	Number in GO	Percent Changed	Percent Present	Z Score	Permuted P Value
1	intracellular	C	32	6943	8118	0.4609	85.5260	3.0670	0.0040
2	protein binding	F	23	4725	5185	0.4868	91.1283	2.6200	0.0050
3	electron transport	P	4	386	461	1.0363	83.7310	2.5880	0.0340
4	extracellular matrix (sensu Metazoa)	C	3	256	273	1.1719	93.7729	2.4910	0.0370
5	cytoplasm	C	17	3488	3969	0.4874	87.8811	2.1310	0.0410
