# Supplementary material for: Oil palm phenolics attenuate changes caused by an atherogenic diet in mice
Source: Eur J Nutr. 2012 Apr 11;52(2):443–56. doi: 10.1007/s00394-012-0346-0 (PMC3573186; doi:10.1007/s00394-012-0346-0)
Supplement: Supplementary file 4 — Supplementary material 4 (PDF 84 kb) [file 394_2012_346_MOESM4_ESM.pdf]

Article Title: Oil palm phenolics attenuate changes caused by an atherogenic diet in mice.

Journal Name: European Journal of Nutrition.

Author Names: Soon-Sen Leow, Shamala Devi Sekaran, Kalyana Sundram, YewAi Tan, Ravigadevi Sambanthamurthi.

Corresponding Author: Ravigadevi Sambanthamurthi; Malaysian Palm Oil Board, No. 6, Persiaran Institusi, Bandar Baru Bangi, 43000 Kajang, Selangor, Malaysia; E-mail: raviga@mpob.gov.my.

Supplementary Material 4

This file contains three tables which show lists of genes significantly changed by the atherogenic diet and OPP in livers, spleens and hearts of mice. Genes significantly changed by OPP were compared to those significantly changed by the atherogenic diet in terms of the direction of fold changes, with the significance of genes determined via independent comparisons. The first comparison was Atherogenic Diet + Distilled Water versus Normal Diet + Distilled Water, while the second comparison was Atherogenic Diet + OPP : Atherogenic Diet + Distilled Water. A gene was identified as significantly changed in each comparison if its |Differential Score| was more than 20, which was equivalent to a P Value of less than 0.01, with the exception of changes in the heart, in which the stringency criterion was lowered to a |Differential Score| of more than 13, which was equivalent to a P Value of less than 0.05. Positive fold changes indicate up-regulation while negative fold changes indicate down-regulation. Genes coloured red were up-regulated by the atherogenic diet but down-regulated by OPP while genes coloured blue were *vice versa*. Genes coloured black were regulated in the same direction by both factors.

Table A13 Genes significantly changed by the atherogenic diet and OPP in livers.

Table A14 Genes significantly changed by the atherogenic diet and OPP in spleens.

Table A15 Genes significantly changed by the atherogenic diet and OPP in hearts.

TABLE A13						
GENES SIGNIFICANTLY CHANGED BY THE ATHEROGENIC DIET AND OPP IN LIVERS						
TargetID	Symbol	Accession	Definition	AD+DW / ND+DW	AD+OPP / AD+DW	Change
scl068028.3_20-S	3110001N18Rik	NM_026517.1	Mus musculus RIKEN cDNA 3110001N18 gene (3110001N18Rik), mRNA.	104.83	-1.46	Y
scl31028.4.1_18-S	Aqp11	NM_175105.2	Mus musculus aquaporin 11 (Aqp11), mRNA.	101.84	-2.09	Y
scl0066881.2_144-S	Pcyox1	NM_025823.3	Mus musculus prenylcysteine oxidase 1 (Pcyox1), mRNA.	82.76	-1.52	Y
scl0013809.2_54-S	Enpep	NM_007934.1	Mus musculus glutamyl aminopeptidase (Enpep), mRNA.	62.76	-1.46	Y
gi_30794511_ref_NM_(	Hmbs	NM_013551.1	Mus musculus hydroxymethylbilane synthase (Hmbs), mRNA.	59.11	-1.41	Y
scl011958.2_29-S	Atp5k	NM_007507	Mus musculus ATP synthase, H+ transporting, mitochondrial F1F0 complex, subunit e (Atp5k), mRNA.	57.02	-1.67	Y
scl0001121.1_1-S	Ndufa5	NM_026614.1	Mus musculus NADH dehydrogenase (ubiquinone) 1 alpha subcomplex, 5 (Ndufa5), mRNA.	49.87	-1.37	Y
scl069786.2_30-S	1810034M08Rik	NM_176842.2	Mus musculus RIKEN cDNA 1810034M08 gene (1810034M08Rik), mRNA.	46.35	-1.90	Y
scl47189.8.1_128-S	Mrpl13	NM_026759.2	Mus musculus mitochondrial ribosomal protein L13 (Mrpl13), mRNA.	38.56	-1.26	Y
scl23150.4.1_11-S	Aadac	NM_023383.1	Mus musculus arylacetamide deacetylase (esterase) (Aadac), mRNA.	36.53	-1.59	Y
scl22246.19.1_4-S	Mccc1	NM_023644.2	Mus musculus methylcrotonoyl-Coenzyme A carboxylase 1 (alpha) (Mccc1), mRNA.	36.38	-1.33	Y
scl093739.4_24-S	Gabarapl2	NM_026693.2	Mus musculus GABA(A) receptor-associated protein like 2 (Gabarapl2), mRNA.	32.83	-1.65	Y
scl023986.1_240-S	Peci	NM_011868.1	Mus musculus peroxisomal delta3, delta2-enoyl-Coenzyme A isomerase (Peci), mRNA.	28.54	-1.60	Y
scl53954.7_10-S	Slc16a2	NM_009197.1	Mus musculus solute carrier family 16 (monocarboxylic acid transporters), member 2 (Slc16a2), mRNA.	24.11	-1.54	Y
scl017449.1_1-S	Mdh1	NM_008618.2	Mus musculus malate dehydrogenase 1, NAD (soluble) (Mdh1), mRNA.	23.31	-1.49	Y
scl017836.18_18-S	Mug1	NM_008645.2	Mus musculus murinoglobulin 1 (Mug1), mRNA.	22.36	-1.87	Y
scl30665.8.1_14-S	1110032O16Rik	XM_133813.4	Mus musculus RIKEN cDNA 1110032O16 gene (1110032O16Rik), mRNA.	15.98	-1.49	Y
scl0015129.1_300-S	Hbb-b1	NM_008220.2	Mus musculus hemoglobin, beta adult major chain (Hbb-b1), mRNA.	13.43	-2.21	Y
scl00353208.1_64-S	2810021G02Rik			12.54	-1.46	Y
scl0277333.1_280-S	MGC68323	NM_199472.1	Mus musculus similar to glyceraldehyde-3-phosphate dehydrogenase (phosphorylating) (EC 1.2.1.12) - mouse (MGC68323), mRNA.	11.27	-1.59	Y
scl29306.21.1_2-S	Slc25a13	NM_015829.1	Mus musculus solute carrier family 25 (mitochondrial carrier; adenine nucleotide translocator), member 13 (Slc25a13), mRNA.	10.32	-1.24	Y
scl16633.10.1_180-S	Pecr	NM_023523.3	Mus musculus peroxisomal trans-2-enoyl-CoA reductase (Pecr), mRNA.	9.87	-1.36	Y
scl056043.1_7-S	Akr1e1	NM_018859.1	Mus musculus aldo-keto reductase family 1, member E1 (Akr1e1), mRNA.	9.60	-1.52	Y
scl0069354.1_68-S	Slc38a4	NM_027052.2	Mus musculus solute carrier family 38, member 4 (Slc38a4), mRNA.	9.50	-1.37	Y
scl00232449.2_167-S	2500002K03Rik	NM_172733.1	Mus musculus RIKEN cDNA 2500002K03 gene (2500002K03Rik), mRNA.	8.64	-1.40	Y
scl015024.2_236-S	H2-T10	NM_010395.2	Mus musculus histocompatibility 2, T region locus 10 (H2-T10), mRNA.	8.47	-1.50	Y
scl0245867.4_1-S	5330414D10Rik	NM_153594.2	Mus musculus RIKEN cDNA 5330414D10 gene (5330414D10Rik), mRNA.	8.16	-1.57	Y
scl27606.14.1_65-S	Afm	NM_145146.1	Mus musculus afamin (Afm), mRNA.	7.44	-1.33	Y
scl29983.21.1_98-S	Abcg2	NM_011920.1	Mus musculus ATP-binding cassette, sub-family G (WHITE), member 2 (Abcg2), mRNA.	5.51	-2.43	Y
scl030931.1_245-S	Dyt1	NM_144884.1	Mus musculus dystonia 1 (Dyt1), mRNA.	5.46	-1.29	Y
scl31053.2.1_38-S	1810020E01Rik	NM_025460.1	Mus musculus RIKEN cDNA 1810020E01 gene (1810020E01Rik), mRNA.	5.39	-1.30	Y
scl29045.3.1_38-S	Rarres2	NM_027852.1	Mus musculus retinoic acid receptor responder (tazarotene induced) 2 (Rarres2), mRNA.	4.34	-1.34	Y
scl15766.4.1_27-S	1110060M21Rik	NM_025424.1	Mus musculus RIKEN cDNA 1110060M21 gene (1110060M21Rik), mRNA.	4.27	-1.44	Y
scl46659.10_50-S	Zfp385	NM_013866.1	Mus musculus zinc finger protein 385 (Zfp385), mRNA.	3.69	-1.27	Y
scl000022.1_12-S	Cpt2	NM_009949	Mus musculus carnitine palmitoyltransferase 2 (Cpt2), mRNA.	3.50	-1.28	Y
scl076654.7_194-S	Upp2	NM_029692.1	Mus musculus uridine phosphorylase 2 (Upp2), mRNA.	3.24	-1.40	Y
scl0017993.1_168-S	Ndufs4	NM_010887.1	Mus musculus NADH dehydrogenase (ubiquinone) Fe-S protein 4 (Ndufs4), mRNA.	2.98	-1.23	Y
scl0015040.1_90-S	H2-T23	NM_010398.1	Mus musculus histocompatibility 2, T region locus 23 (H2-T23), mRNA.	2.61	-1.33	Y
scl0076654.1_225-S	Upp2	NM_029692.1	Mus musculus uridine phosphorylase 2 (Upp2), mRNA.	2.26	-1.46	Y
scl42858.13_56-S	Rin3	NM_177620.2	Mus musculus Ras and Rab interactor 3 (Rin3), mRNA.	2.14	-1.75	Y
scl24019.5_109-S	Cpt2	NM_009949	Mus musculus carnitine palmitoyltransferase 2 (Cpt2), mRNA.	1.86	-1.25	Y
scl48561.1.13_8-S	0610012G03Rik	NM_025320	Mus musculus RIKEN cDNA 0610012G03 gene (0610012G03Rik), mRNA.	1.73	-1.32	Y
scl50689.6_424-S	Slc35b1	XM_128634.4	Mus musculus solute carrier family 35, member B1 (Slc35b1), mRNA.	1.66	-1.36	Y
scl38453.7.1_27-S	Csrp2	NM_007792.2	Mus musculus cysteine and glycine-rich protein 2 (Csrp2), mRNA.	1.66	-1.35	Y

scl37827.3_399-S	D10Ertd214e	NM_134007.2	Mus musculus DNA segment, Chr 10, ERATO Doi 214, expressed (D10Ertd214e), mRNA.	1.64	-1.54	Y
scl068195.1_129-S	Rnaset2	NM_026611.1	Mus musculus ribonuclease T2 (Rnaset2), mRNA.	1.63	-1.38	Y
scl35398.12.2_77-S	Alas1	NM_020559.1	Mus musculus aminolevulinic acid synthase 1 (Alas1), mRNA.	1.62	-1.36	Y
scl013010.2_9-S	Cst3	NM_009976.2	Mus musculus cystatin C (Cst3), mRNA.	1.62	-1.24	Y
scl27967.7.1_149-S	Abhd1	NM_021304.2	Mus musculus abhydrolase domain containing 1 (Abhd1), mRNA.	1.60	-1.25	Y
scl35702.23.1_49-S	AV340375	NM_172519.1	Mus musculus expressed sequence AV340375 (AV340375), mRNA.	1.54	-1.66	Y
scl44903.11.3_96-S	Fars1	NM_024274.1	Mus musculus phenylalanine-tRNA synthetase 1 (mitochondrial) (Fars1), mRNA.	1.50	-1.42	Y
scl0071755.2_316-S	1300018L09Rik	NM_027903.1		1.49	-1.39	Y
scl013885.9_29-S	Esd	NM_016903		1.48	-1.27	Y
scl0065111.1_63-S	Dap3	NM_022994.2	Mus musculus death associated protein 3 (Dap3), mRNA.	1.47	-1.25	Y
scl0012091.2_274-S	Glb1	NM_009752.1	Mus musculus galactosidase, beta 1 (Glb1), mRNA.	1.46	-1.41	Y
scl28480.7_231-S	Adipor2	NM_197985.2		1.46	-1.23	Y
scl051800.6_308-S	Bok	NM_016778	Mus musculus Bcl-2-related ovarian killer protein (Bok), mRNA.	1.45	-1.74	Y
scl0019299.2_231-S	Abcd3	NM_008991.1	Mus musculus ATP-binding cassette, sub-family D (ALD), member 3 (Abcd3), mRNA.	1.44	-1.42	Y
scl019122.2_199-S	Prnp	NM_011170.1	Mus musculus prion protein (Prnp), mRNA.	1.43	-1.43	Y
scl020317.1_68-S	Serpinf1	NM_011340.2	Mus musculus serine (or cysteine) proteinase inhibitor, clade F, member 1 (Serpinf1), mRNA.	1.41	-1.30	Y
scl51764.24.1_222-S	4933427L07Rik	NM_027727.1	Mus musculus RIKEN cDNA 4933427L07 gene (4933427L07Rik), mRNA.	1.39	-1.60	Y
scl0016923.1_194-S	Lnk	NM_008507.2	Mus musculus linker of T-cell receptor pathways (Lnk), mRNA.	1.37	-1.43	Y
scl28963.13.1_27-S	Nt5c3	NM_026004.1	Mus musculus 5-nucleotidase, cytosolic III (Nt5c3), mRNA.	1.33	-1.38	Y
scl24777.7.1_203-S	Akr7a5	NM_025337.2	Mus musculus aldo-keto reductase family 7, member A5 (aflatoxin aldehyde reductase) (Akr7a5), mRNA.	1.31	-1.35	Y
scl065111.1_137-S	Dap3	NM_022994.2	Mus musculus death associated protein 3 (Dap3), mRNA.	1.30	-1.24	Y
scl020382.2_66-S	Sfrs2	NM_011358.1	Mus musculus splicing factor, arginine/serine-rich 2 (SC-35) (Sfrs2), mRNA.	-3.04	1.49	Y
scl0003591.1_99-S	Keap1	NM_016679.2	Mus musculus kelch-like ECH-associated protein 1 (Keap1), mRNA.	-2.32	1.48	Y
scl49146.12.1_4-S	2610033C09Rik	NM_026407.2	Mus musculus RIKEN cDNA 2610033C09 gene (2610033C09Rik), mRNA.	-1.78	1.70	Y
scl24607.15_131-S	Dvl1	NM_010091.2	Mus musculus dishevelled, dsh homolog 1 (Drosophila) (Dvl1), mRNA.	-1.74	1.45	Y
scl0023881.1_86-S	E430034L04Rik	NM_011816.2	Mus musculus RIKEN cDNA E430034L04 gene (E430034L04Rik), mRNA.	-1.51	1.31	Y
scl52839.2_29-S	AI837181	NM_134149.1	Mus musculus expressed sequence AI837181 (AI837181), mRNA.	-1.46	1.35	Y
scl000086.1_135-S	AA959742	NM_133807.1	Mus musculus expressed sequence AA959742 (AA959742), mRNA.	16.96	1.28	N
scl20056.4.1_63-S	Map1lc3a	NM_025735.1	Mus musculus microtubule-associated protein 1 light chain 3 alpha (Map1lc3a), mRNA.	1.70	1.34	N
scl53392.18_161-S	Mta2	NM_011842.2	Mus musculus metastais-associated gene family, member 2 (Mta2), mRNA.	1.68	1.47	N
scl37659.18.259_11-S	Tra1	NM_011631.1	Mus musculus tumor rejection antigen gp96 (Tra1), mRNA.	1.60	1.35	N
scl49316.10.1_12-S	Dnajb11	XM_148071.1	Mus musculus DnaJ (Hsp40) homolog, subfamily B, member 11 (Dnajb11), mRNA.	1.52	1.89	N
scl020338.1_17-S	Sel1h	NM_011344.1	Mus musculus Sel1 (suppressor of lin-12) 1 homolog (C. elegans) (Sel1h), mRNA.	1.49	1.29	N
scl0012055.2_161-S	Bcl7c	NM_009746	Mus musculus B-cell CLL/lymphoma 7C (Bcl7c), mRNA.	1.39	1.35	N
scl00224938.2_37-S	Pja2	NM_144859.1	Mus musculus praja 2, RING-H2 motif containing (Pja2), mRNA.	1.27	1.33	N

Gene Set	Number of Genes
Unchanged	8
Changed	71
Total	79
**% Changed**	**89.87**

TABLE A14						
GENES SIGNIFICANTLY CHANGED BY THE ATHEROGENIC DIET AND OPP IN SPLEENS						
TargetID	Symbol	Accession	Definition	AD+DW / ND+DW	AD+OPP / AD+DW	Change
scl067596.7_211-S	5830405N20Rik	NM_183264.1	Mus musculus RIKEN cDNA 5830405N20 gene (5830405N20Rik), mRNA.	2.56	-2.44	Y
scl076071.13_258-S	Gababrbp	NM_178394.2		2.14	-2.38	Y
scl017110.1_293-S	Lzp-s	NM_013590.2	Mus musculus P lysozyme structural (Lzp-s), mRNA.	2.08	-5.82	Y
scl43315.9.1_17-S	Sh3yl1	NM_013709.2	Mus musculus Sh3 domain YSC-like 1 (Sh3yl1), mRNA.	2.05	-1.59	Y
scl24261.12.1_29-S	Alad	NM_008525.3	Mus musculus aminolevulinate, delta-, dehydratase (Alad), mRNA.	1.88	-1.36	Y
scl0012846.1_253-S	Comt	XM_147265.1	Mus musculus catechol-O-methyltransferase (Comt), mRNA.	1.87	-1.38	Y
scl25758.6.1_14-S	1200006F02Rik	NM_027872.1	Mus musculus RIKEN cDNA 1200006F02 gene (1200006F02Rik), mRNA.	1.85	-2.21	Y
scl0216792.1_314-S	A230051G13Rik	NM_173785.2		1.85	-1.33	Y
scl0093760.1_64-S	Arid1a	NM_033566.1	Mus musculus AT rich interactive domain 1A (Swi1 like) (Arid1a), mRNA.	1.83	-1.85	Y
scl0072691.1_193-S	2810048G17Rik	NM_133746.2	Mus musculus RIKEN cDNA 2810048G17 gene (2810048G17Rik), mRNA.	1.78	-1.58	Y
scl35771.8.1_41-S	2410076I21Rik	XM_134948.2	Mus musculus RIKEN cDNA 2410076I21 gene (2410076I21Rik), mRNA.	1.71	-1.73	Y
scl37022.6.1_3-S	Cd3d	NM_013487.1	Mus musculus CD3 antigen, delta polypeptide (Cd3d), mRNA.	1.71	-1.55	Y
scl0066610.1_317-S	Abi3	NM_025659.1	Mus musculus ABI gene family, member 3 (Abi3), mRNA.	1.69	-1.83	Y
scl0029809.2_171-S	Rabgap1l	NM_013862.3		1.68	-1.47	Y
scl39506.28.1461_10-S	Slc4a1	NM_011403.1	Mus musculus solute carrier family 4 (anion exchanger), member 1 (Slc4a1), mRNA.	1.68	-1.44	Y
scl50197.1.198_198-S	Fahd1	NM_023480.1		1.66	-1.30	Y
scl38917.7_7-S	Gja1	NM_010288.2	Mus musculus gap junction membrane channel protein alpha 1 (Gja1), mRNA.	1.64	-1.47	Y
scl45283.8.141_93-S	9030625A04Rik	NM_172488.1	Mus musculus RIKEN cDNA 9030625A04 gene (9030625A04Rik), mRNA.	1.62	-1.94	Y
scl0002648.1_0-S	Mllt3	NM_027326.2	Mus musculus myeloid/lymphoid or mixed lineage-leukemia translocation to 3 homolog (Drosophila) (Mllt3), mRNA.	1.59	-1.44	Y
scl020926.1_330-S	Supt6h	NM_009297.1	Mus musculus suppressor of Ty 6 homolog (S. cerevisiae) (Supt6h), mRNA.	1.57	-1.41	Y
scl35429.19.1_59-S	1300017J02Rik	NM_027918.1	Mus musculus RIKEN cDNA 1300017J02 gene (1300017J02Rik), mRNA.	1.56	-1.40	Y
scl41638.9.1_29-S	Timd4	NM_178759.3		1.55	-2.01	Y
scl19453.6.2634_3-S	A130092J06Rik	NM_175511.2	Mus musculus RIKEN cDNA A130092J06 gene (A130092J06Rik), mRNA.	1.51	-1.52	Y
scl0020452.1_241-S	Siat8d	NM_009183.1	Mus musculus sialyltransferase 8 (alpha-2, 8-sialyltransferase) D (Siat8d), mRNA.	1.48	-1.67	Y
scl49300.12_501-S	St6gal1	NM_145933.2	Mus musculus beta galactoside alpha 2,6 sialyltransferase 1 (St6gal1), mRNA.	1.47	-1.67	Y
scl068066.1_115-S	D11Ertd333e	NM_026542.1		1.46	-1.46	Y
scl021858.1_100-S	Timp2	NM_011594.2	Mus musculus tissue inhibitor of metalloproteinase 2 (Timp2), mRNA.	1.45	-1.58	Y
scl0020947.1_79-S	Swap70	NM_009302.2	Mus musculus SWAP complex protein (Swap70), mRNA.	1.45	-1.50	Y
scl066158.1_320-S	1110012O05Rik	NM_024170	Mus musculus RIKEN cDNA 1110012O05 gene (1110012O05Rik), mRNA.	1.44	-1.73	Y
scl0012192.2_207-S	Zfp36l1	NM_007564.2	Mus musculus zinc finger protein 36, C3H type-like 1 (Zfp36l1), mRNA.	1.43	-1.93	Y
scl32299.7.1_25-S	Stard10	NM_019990.1	Mus musculus START domain containing 10 (Stard10), mRNA.	1.43	-1.45	Y
scl23946.8.1_41-S	Urod	NM_009478.1	Mus musculus uroporphyrinogen decarboxylase (Urod), mRNA.	1.43	-1.27	Y
scl55061.19.1_283-S	Slc38a5	NM_172479.1		1.42	-1.39	Y
scl0012045.1_114-S	Bcl2a1b	NM_007534	Mus musculus B-cell leukemia/lymphoma 2 related protein A1b (Bcl2a1b), mRNA.	1.41	-2.31	Y
scl0054613.2_126-S	Siat10	NM_018784.1	Mus musculus sialyltransferase 10 (alpha-2,3-sialyltransferase VI) (Siat10), mRNA.	1.40	-1.35	Y
scl53380.11_494-S	Fads1	NM_146094.1	Mus musculus fatty acid desaturase 1 (Fads1), mRNA.	1.39	-1.24	Y
scl027556.1_96-S	Clic4	XM_124389.1	Mus musculus chloride intracellular channel 4 (mitochondrial) (Clic4), mRNA.	1.37	-1.26	Y
scl32898.7.1_80-S	Blvrb	NM_144923	Mus musculus biliverdin reductase B (flavin reductase (NADPH)) (Blvrb), mRNA.	1.35	-1.54	Y
scl0067238.2_233-S	2810453I06Rik	NM_026050.1	Mus musculus RIKEN cDNA 2810453I06 gene (2810453I06Rik), mRNA.	1.34	-1.71	Y
scl33241.10_186-S	Icsbp1	NM_008320.2	Mus musculus interferon consensus sequence binding protein 1 (Icsbp1), mRNA.	1.33	-2.06	Y
scl0231086.16_205-S	Hadhb	NM_145558.1	Mus musculus hydroxyacyl-Coenzyme A dehydrogenase/3-ketoacyl-Coenzyme A thiolase/enoyl-Coenzyme A hydratase (trifunctional protein), beta subunit (Hadhb), mRNA.	1.32	-1.28	Y
scl45706.11_2-S	Ghitm	NM_078478.1	Mus musculus growth hormone inducible transmembrane protein (Ghitm), mRNA.	1.31	-1.46	Y
scl30833.27_114-S	St5	NM_029811.1	Mus musculus suppression of tumorigenicity 5 (St5), mRNA.	1.30	-1.49	Y
scl0001934.1_38-S	Lmna	NM_019390.1	Mus musculus lamin A (Lmna), mRNA.	1.30	-1.43	Y

scl056812.9_277-S	Dnajb10	NM_178055.2	Mus musculus DnaJ (Hsp40) homolog, subfamily B, member 10 (Dnajb10), mRNA.	1.29	-1.65	Y
scl45393.10_71-S	Bnip3l	NM_009761.2	Mus musculus BCL2/adenovirus E1B 19kDa-interacting protein 3-like (Bnip3l), mRNA.	1.24	-1.40	Y
scl0016796.1_242-S	Lasp1	NM_010688.2	Mus musculus LIM and SH3 protein 1 (Lasp1), mRNA.	-2.29	2.05	Y
scl00394432.2_137-S	Ugt1a10	NM_201642		-2.13	2.48	Y
scl35222.4_425-S	Myd88	NM_010851	Mus musculus myeloid differentiation primary response gene 88 (Myd88), mRNA.	-2.02	2.10	Y
scl072042.2_8-S	Cotl1	XM_150115.1	Mus musculus coactosin-like 1 (Dictyostelium) (Cotl1), mRNA.	-1.96	1.86	Y
scl53421.25_339-S	D19Ertd703e	NM_029456.1	Mus musculus DNA segment, Chr 19, ERATO Doi 703, expressed (D19Ertd703e), mRNA.	-1.86	1.39	Y
scl011867.9_22-S	Arpc1b	NM_023142.1	Mus musculus actin related protein 2/3 complex, subunit 1B (Arpc1b), mRNA.	-1.83	1.65	Y
scl080876.2_10-S	Ifitm2	NM_030694	Mus musculus interferon induced transmembrane protein 2 (Ifitm2), mRNA.	-1.81	1.77	Y
scl30947.3_219-S	Rhog	NM_019566.2	Mus musculus ras homolog gene family, member G (Rhog), mRNA.	-1.80	1.60	Y
scl0276770.1_104-S	Eif5a	NM_181582.2	Mus musculus eukaryotic translation initiation factor 5A (Eif5a), mRNA.	-1.76	1.41	Y
scl36516.9.290_30-S	Ptk9l	NM_011876.2	Mus musculus protein tyrosine kinase 9-like (A6-related protein) (Ptk9l), mRNA.	-1.69	1.70	Y
scl50849.26.1_265-S	Myo1f	NM_053214.1	Mus musculus myosin IF (Myo1f), mRNA.	-1.63	2.04	Y
scl52691.13_82-S	Psat1	XM_129211.2	Mus musculus phosphoserine aminotransferase 1 (Psat1), mRNA.	-1.54	1.71	Y
scl068713.2_9-S	Ifitm1	NM_026820	Mus musculus interferon induced transmembrane protein 1 (Ifitm1), mRNA.	-1.52	1.56	Y
scl52675.8.401_6-S	Ostf1	NM_017375.1	Mus musculus osteoclast stimulating factor 1 (Ostf1), mRNA.	-1.52	1.44	Y
scl016541.8_1-S	Napsa	NM_008437.1		-1.47	1.43	Y
scl0004022.1_70-S	Arpc1b	NM_023142.1	Mus musculus actin related protein 2/3 complex, subunit 1B (Arpc1b), mRNA.	-1.40	1.69	Y
scl54435.12.1_121-S	Was	NM_009515.1	Mus musculus Wiskott-Aldrich syndrome homolog (human) (Was), mRNA.	-1.38	1.43	Y
scl056378.6_20-S	Arpc3	NM_019824.2	Mus musculus actin related protein 2/3 complex, subunit 3 (Arpc3), mRNA.	-1.37	1.38	Y
scl0059069.1_170-S	Tpm3	NM_022314.2	Mus musculus tropomyosin 3, gamma (Tpm3), mRNA.	-1.36	1.32	Y
scl50794.7.1_43-S	Clic1	NM_033444.1	Mus musculus chloride intracellular channel 1 (Clic1), mRNA.	-1.35	1.50	Y
scl068089.7_12-S	Arpc4	NM_026552	Mus musculus actin related protein 2/3 complex, subunit 4 (Arpc4), mRNA.	-1.32	1.57	Y
scl00230316.2_241-S	Egfl5	NM_172694.1	Mus musculus EGF-like-domain, multiple 5 (Egfl5), mRNA.	5.33	2.40	N
scl48582.2_713-S	Gp5	NM_008148.2	Mus musculus glycoprotein 5 (platelet) (Gp5), mRNA.	3.10	1.55	N
scl0023845.2_228-S	Clecsf5	NM_021364.1	Mus musculus C-type (calcium dependent, carbohydrate-recognition domain) lectin, superfamily member 5 (Clecsf5), mRNA.	3.08	2.29	N
scl39908.21.1_34-S	Cpd	NM_007754.1	Mus musculus carboxypeptidase D (Cpd), mRNA.	2.76	1.77	N
scl16186.5.1_30-S	Rgs18	NM_022881.2	Mus musculus regulator of G-protein signaling 18 (Rgs18), mRNA.	2.67	1.62	N
scl30062.4.1_1-S	Npy	NM_023456.2	Mus musculus neuropeptide Y (Npy), mRNA.	2.49	1.96	N
scl21429.6_290-S	Lmo4	NM_010723.2	Mus musculus LIM domain only 4 (Lmo4), mRNA.	2.48	1.71	N
scl52908.26.6_30-S	2610041P08Rik	NM_198008.1	Mus musculus RIKEN cDNA 2610041P08 gene (2610041P08Rik), mRNA.	2.33	1.44	N
scl35398.12.2_77-S	Alas1	NM_020559.1	Mus musculus aminolevulinic acid synthase 1 (Alas1), mRNA.	2.25	2.75	N
scl0012874.1_259-S	Cpd	NM_007754.1	Mus musculus carboxypeptidase D (Cpd), mRNA.	2.16	2.10	N
scl41300.23_511-S	Atp2a3	NM_016745.2	Mus musculus ATPase, Ca++ transporting, ubiquitous (Atp2a3), mRNA.	2.13	1.42	N
scl28709.9_141-S	Abtb1	NM_030251.1	Mus musculus ankyrin repeat and BTB (POZ) domain containing 1 (Abtb1), mRNA.	2.11	1.29	N
scl00234797.2_307-S	6430548M08Rik	NM_172286.2	Mus musculus RIKEN cDNA 6430548M08 gene (6430548M08Rik), mRNA.	2.07	1.73	N
scl28438.14_163-S	Slc2a3	NM_011401.2	Mus musculus solute carrier family 2 (facilitated glucose transporter), member 3 (Slc2a3), mRNA.	2.03	2.39	N
scl0002552.1_594-S	Triobp	NM_138579.2	Mus musculus TRIO and F-actin binding protein (Triobp), mRNA.	1.96	1.52	N
scl0018728.1_38-S	Pira5			1.96	1.54	N
scl30807.22.1_70-S	Mrvi1	NM_194464.1	Mus musculus MRV integration site 1 (Mrvi1), transcript variant 1, mRNA.	1.90	1.47	N
scl50763.13.1_199-S	Flot1	NM_008027.1	Mus musculus flotillin 1 (Flot1), mRNA.	1.89	1.70	N
scl38701.12.1_200-S	Arid3a	NM_007880.1	Mus musculus AT rich interactive domain 3A (Bright like) (Arid3a), mRNA.	1.84	2.66	N
scl0002340.1_98-S	Nin	NM_008697	Mus musculus ninein (Nin), mRNA.	1.83	1.67	N
scl0018230.2_224-S	Nxn	NM_008750.2	Mus musculus nucleoredoxin (Nxn), mRNA.	1.82	1.86	N
scl022793.9_329-S	Zyx	NM_011777.1	Mus musculus zyxin (Zyx), mRNA.	1.79	1.80	N
scl34795.4.1_9-S	Sap30	NM_021788.1	Mus musculus sin3 associated polypeptide (Sap30), mRNA.	1.75	1.67	N
scl37440.14_650-S	Irak3	NM_028679.2	Mus musculus interleukin-1 receptor-associated kinase 3 (Irak3), mRNA.	1.74	1.68	N
scl51004.4_55-S	Sepx1	NM_013759.1		1.74	1.71	N

scl012765.6_0-S	Il8rb	NM_009909.2	Mus musculus interleukin 8 receptor, beta (Il8rb), mRNA.	1.74	2.05	N
scl0023821.2_49-S	Bace1	NM_011792.3	Mus musculus beta-site APP cleaving enzyme 1 (Bace1), mRNA.	1.72	1.45	N
scl34132.19.1_1-S	Stxbp2	NM_011503.2	Mus musculus syntaxin binding protein 2 (Stxbp2), mRNA.	1.72	1.50	N
scl011964.1_274-S	Atp6v1a1	NM_007508.2	Mus musculus ATPase, H+ transporting, V1 subunit A, isoform 1 (Atp6v1a1), mRNA.	1.72	1.55	N
scl48225.31_410-S	Tiam1	NM_009384.1	Mus musculus T-cell lymphoma invasion and metastasis 1 (Tiam1), mRNA.	1.70	1.74	N
scl069583.1_127-S	Tnfsf13	NM_023517.1	Mus musculus tumor necrosis factor (ligand) superfamily, member 13 (Tnfsf13), mRNA.	1.67	1.67	N
scl0020661.1_145-S	Sort1	NM_019972	Mus musculus sortilin 1 (Sort1), mRNA.	1.65	1.89	N
scl0110253.14_323-S	Triobp	NM_138579.2	Mus musculus TRIO and F-actin binding protein (Triobp), mRNA.	1.62	1.40	N
scl067905.1_18-S	2810423O19Rik	NM_026447	Mus musculus RIKEN cDNA 2810423O19 gene (2810423O19Rik), transcript variant 1, mRNA.	1.62	1.54	N
scl23525.7_662-S	Agtrap	NM_009642.3	Mus musculus angiotensin II, type I receptor-associated protein (Agtrap), mRNA.	1.60	1.82	N
scl013728.1_329-S	Mark2	NM_007928.1	Mus musculus MAP/microtubule affinity-regulating kinase 2 (Mark2), mRNA.	1.58	1.24	N
scl27109.17.1_35-S	Plod3	NM_011962.2	Mus musculus procollagen-lysine, 2-oxoglutarate 5-dioxygenase 3 (Plod3), mRNA.	1.58	1.57	N
scl19517.6_242-S	Surf4	NM_011512.2	Mus musculus surfeit gene 4 (Surf4), mRNA.	1.56	1.31	N
scl35462.25_609-S	Pik3cb	NM_029094.1	Mus musculus phosphatidylinositol 3-kinase, catalytic, beta polypeptide (Pik3cb), mRNA.	1.55	1.32	N
scl40534.12.1_29-S	Tbrg4	NM_134011.1	Mus musculus transforming growth factor beta regulated gene 4 (Tbrg4), mRNA.	1.55	1.54	N
scl16390.7_3-S	Ralb	NM_022327.3	Mus musculus v-ral simian leukemia viral oncogene homolog B (ras related) (Ralb), mRNA.	1.55	1.70	N
scl0078334.1_222-S	2700084L06Rik	NM_198164.1	Mus musculus RIKEN cDNA 2700084L06 gene (2700084L06Rik), mRNA.	1.53	1.47	N
scl0030955.2_290-S	Pik3cg	NM_020272.1	Mus musculus phosphoinositide-3-kinase, catalytic, gamma polypeptide (Pik3cg), mRNA.	1.52	1.41	N
scl23461.27_162-S	Kcnab2	NM_010598.2	Mus musculus potassium voltage-gated channel, shaker-related subfamily, beta member 2 (Kcnab2), mRNA.	1.50	1.64	N
scl26088.14.1_16-S	Aldh2	NM_009656.1	Mus musculus aldehyde dehydrogenase 2, mitochondrial (Aldh2), mRNA.	1.47	1.34	N
scl066989.6_1-S	2410004N11Rik	NM_025888.2	Mus musculus RIKEN cDNA 2410004N11 gene (2410004N11Rik), mRNA.	1.46	1.31	N
scl24620.16_177-S	BC004012	NM_138671	Mus musculus cDNA sequence BC004012 (BC004012), mRNA.	1.46	1.32	N
scl36920.15.1_107-S	Pstpip1	NM_011193.1	Mus musculus proline-serine-threonine phosphatase-interacting protein 1 (Pstpip1), mRNA.	1.46	2.00	N
scl54141.46.3_34-S	Flna	XM_289920.2	Mus musculus filamin, alpha (Flna), mRNA.	1.45	1.37	N
scl21336.8_145-S	3110001A13Rik	NM_025626.3	Mus musculus RIKEN cDNA 3110001A13 gene (3110001A13Rik), mRNA.	1.44	1.52	N
scl48556.8.1_18-S	2010319C14Rik	NM_024464.2	Mus musculus RIKEN cDNA 2010319C14 gene (2010319C14Rik), mRNA.	1.39	1.33	N
scl013244.1_23-S	Degs	NM_007853.2	Mus musculus degenerative spermatocyte homolog (Drosophila) (Degs), mRNA.	1.36	1.29	N
scl36385.5_13-S	Cklfsf6	NM_026036.1	Mus musculus chemokine-like factor super family 6 (Cklfsf6), mRNA.	1.36	1.54	N
scl16258.13.1_64-S	Rnpep	NM_145417	Mus musculus arginyl aminopeptidase (aminopeptidase B) (Rnpep), mRNA.	1.35	1.65	N
scl016784.1_5-S	Lamp2	NM_010685.2	Mus musculus lysosomal membrane glycoprotein 2 (Lamp2), mRNA.	1.33	1.34	N
scl18197.5_22-S	Rgs19	NM_026446.2	Mus musculus regulator of G-protein signaling 19 (Rgs19), mRNA.	1.32	1.40	N
scl45684.12_151-S	D14Ertd226e	NM_145928	Mus musculus DNA segment, Chr 14, ERATO Doi 226, expressed (D14Ertd226e), mRNA.	1.29	1.52	N
scl066151.5_253-S	1110020C13Rik	NM_025385.2	Mus musculus RIKEN cDNA 1110020C13 gene (1110020C13Rik), mRNA.	1.27	1.31	N
scl0114887.6_24-S	Rmcs1	NM_207105.1	Mus musculus response to metastatic cancers 1 (Rmcs1), mRNA.	-3.00	-2.87	N
scl43224.11.1_2-S	Coch	NM_007728.2	Mus musculus coagulation factor C homolog (Limulus polyphemus) (Coch), mRNA.	-2.40	-3.29	N
scl014998.4_34-S	H2-DMa	NM_010386	Mus musculus histocompatibility 2, class II, locus DMa (H2-DMa), mRNA.	-2.29	-2.10	N
scl45915.9_517-S	Dnase1l3	NM_007870.2	Mus musculus deoxyribonuclease 1-like 3 (Dnase1l3), mRNA.	-2.16	-2.41	N
scl0066748.2_33-S	4933404M02Rik	NM_025744.1	Mus musculus RIKEN cDNA 4933404M02 gene (4933404M02Rik), mRNA.	-2.02	-1.56	N
scl0110557.2_204-S	H2-Q6	NM_207648	Mus musculus histocompatibility 2, Q region locus 6 (H2-Q6), mRNA.	-1.82	-2.22	N
scl0002580.1_4-S	Ptp4a3	NM_008975.2	Mus musculus protein tyrosine phosphatase 4a3 (Ptp4a3), mRNA.	-1.80	-1.80	N
scl000781.1_68-S	Kif21b	NM_019962.2	Mus musculus kinesin family member 21B (Kif21b), mRNA.	-1.75	-1.79	N
scl40203.12_25-S	Sparc	NM_009242	Mus musculus secreted acidic cysteine rich glycoprotein (Sparc), mRNA.	-1.67	-1.74	N
scl0013829.2_259-S	Epb4.9	NM_013514.2	Mus musculus erythrocyte protein band 4.9 (Epb4.9), mRNA.	-1.65	-1.28	N
scl056620.6_30-S	Clecsf10	NM_020001.1	Mus musculus C-type (calcium dependent, carbohydrate recognition domain) lectin, superfamily member 10 (Clecsf10), mRNA.	-1.60	-1.93	N
scl022142.1_236-S	Tuba1	NM_011653	Mus musculus tubulin, alpha 1 (Tuba1), mRNA.	-1.50	-1.36	N
scl0003429.1_37-S	Hmbs	NM_013551	Mus musculus hydroxymethylbilane synthase (Hmbs), mRNA.	-1.49	-1.43	N
scl000673.1_3-S	Prdx2	NM_011563	Mus musculus peroxiredoxin 2 (Prdx2), mRNA.	-1.48	-1.53	N
scl42795.16.1_26-S	Evl	NM_007965.2	Mus musculus Ena-vasodilator stimulated phosphoprotein (Evl), mRNA.	-1.46	-2.40	N

scl41542.4_321-S	Gm2a	NM_010299.2	Mus musculus GM2 ganglioside activator protein (Gm2a), mRNA.	-1.43	-1.86	N
scl0224703.1_41-S	9530046H09Rik	NM_145486.2	Mus musculus RIKEN cDNA 9530046H09 gene (9530046H09Rik), mRNA.	-1.33	-1.48	N
scl012988.1_164-S	Csk	NM_007783.2	Mus musculus c-src tyrosine kinase (Csk), mRNA.	-1.33	-1.50	N
scl012521.1_26-S	Kai1	NM_007656.1	Mus musculus kangai 1 (suppression of tumorigenicity 6, prostate) (Kai1), mRNA.	-1.28	-1.39	N
scl0022214.2_142-S	Ube2h	XM_133034.1	Mus musculus ubiquitin-conjugating enzyme E2H (Ube2h), mRNA.	-1.27	-1.36	N

Gene Set	Number of Genes
Unchanged	78
Changed	67
Total	145
**% Changed**	**46.21**

TABLE A15						
GENES SIGNIFICANTLY CHANGED BY THE ATHEROGENIC DIET AND OPP IN HEARTS						
TargetID	Symbol	Accession	Definition	AD+DW / ND+DW	AD+OPP / AD+DW	Change
scl40333.9_365-S	Mat2b	NM_134017.1	Mus musculus methionine adenosyltransferase II, beta (Mat2b), mRNA.	4.67	-1.34	Y
scl54562.12.1_64-S	Alas2	NM_009653.1	Mus musculus aminolevulinic acid synthase 2, erythroid (Alas2), mRNA.	2.43	-1.59	Y
scl0017765.2_254-S	Mtf2	NM_013827.1	Mus musculus metal response element binding transcription factor 2 (Mtf2), mRNA.	2.15	-1.38	Y
scl19105.6_353-S	Ttn	XM_130312.3	Mus musculus titin (Ttn), mRNA.	2.10	-1.22	Y
scl0076895.2_316-S	Bicd2	NM_029791.2	Mus musculus bicaudal D homolog 2 (Drosophila) (Bicd2), mRNA.	2.03	-1.54	Y
scl0022070.1_31-S	Tpt1	NM_009429	Mus musculus tumor protein, translationally-controlled 1 (Tpt1), mRNA.	2.02	-1.27	Y
scl42532.6_0-S	Tm4sf13	NM_025359.2	Mus musculus transmembrane 4 superfamily member 13 (Tm4sf13), mRNA.	1.99	-1.58	Y
scl32857.8.1_60-S	Lgals4	NM_010706.1	Mus musculus lectin, galactose binding, soluble 4 (Lgals4), mRNA.	1.96	-1.30	Y
scl36105.10.25_13-S	Tbx20	NM_194263.1	Mus musculus T-box 20 (Tbx20), transcript variant 2, mRNA.	1.83	-1.51	Y
scl26992.46_0-S	AI481500	NM_133901.1	Mus musculus expressed sequence AI481500 (AI481500), mRNA.	1.80	-1.31	Y
scl0003447.1_0-S	Rbm5	NM_148930.2	Mus musculus RNA binding motif protein 5 (Rbm5), mRNA.	1.77	-1.83	Y
scl17047.9.4_6-S	BC013667	NM_172266.1	Mus musculus cDNA sequence BC013667 (BC013667), mRNA.	1.72	-1.33	Y
scl0001100.1_114-S	Aqp1	NM_007472.1	Mus musculus aquaporin 1 (Aqp1), mRNA.	1.59	-1.20	Y
scl50134.9_8-S	Nudt3	NM_019837.1	Mus musculus nudix (nucleotide diphosphate linked moiety X)-type motif 3 (Nudt3), mRNA.	1.57	-1.20	Y
scl21522.25_9-S	Egf	NM_010113.1	Mus musculus epidermal growth factor (Egf), mRNA.	1.50	-1.71	Y
scl0224807.4_25-S	BC026370	NM_198167.1	Mus musculus cDNA sequence BC026370 (BC026370), mRNA.	1.42	-1.56	Y
scl27246.12.1_228-S	BC035291	NM_177581.2	Mus musculus cDNA sequence BC035291 (BC035291), mRNA.	1.42	-1.34	Y
scl0003527.1_242-S	Ddx6	NM_007841.2	Mus musculus DEAD (Asp-Glu-Ala-Asp) box polypeptide 6 (Ddx6), mRNA.	1.40	-1.30	Y
scl22977.3.91_193-S	Dpm3	XM_130951.1	Mus musculus dolichyl-phosphate mannosyltransferase polypeptide 3 (Dpm3), mRNA.	1.38	-1.27	Y
scl27262.6_658-S	9130017A15Rik	NM_177242.3	Mus musculus RIKEN cDNA 9130017A15 gene (9130017A15Rik), mRNA.	1.35	-1.18	Y
scl067371.1_2-S	2410016F19Rik	NM_026113.2	Mus musculus RIKEN cDNA 2410016F19 gene (2410016F19Rik), mRNA.	1.34	-1.47	Y
scl21064.31_2-S	5830434P21Rik	NM_172661.2	Mus musculus RIKEN cDNA 5830434P21 gene (5830434P21Rik), mRNA.	1.30	-1.18	Y
scl0015516.2_121-S	Hspcb	NM_008302.2	Mus musculus heat shock protein 1, beta (Hspcb), mRNA.	1.26	-1.29	Y
scl17563.43_11-S	Clasp1		Mus musculus CLIP associating protein 1 (Clasp1), mRNA.	1.20	-1.17	Y
scl0003537.1_10-S	Pcolce2	NM_029620.1	Mus musculus procollagen C-endopeptidase enhancer 2 (Pcolce2), mRNA.	-2.50	2.01	Y
scl29544.11_278-S	Mfap5	NM_015776	Mus musculus microfibrillar associated protein 5 (Mfap5), mRNA.	-1.89	2.05	Y
scl0230514.5_86-S	Obrgrp	NM_175036	Mus musculus leptin receptor gene-related protein (Obrgrp), mRNA.	-1.57	1.25	Y
scl33301.5.15_0-S	Nudt7	NM_024437	Mus musculus nudix (nucleoside diphosphate linked moiety X)-type motif 7 (Nudt7), mRNA.	-1.54	1.21	Y
scl28399.6.24_1-S	Cd9	NM_007657.2	Mus musculus CD9 antigen (Cd9), mRNA.	-1.53	1.31	Y
scl0003638.1_65-S	2310016C16Rik	NM_027127.1	Mus musculus RIKEN cDNA 2310016C16 gene (2310016C16Rik), mRNA.	-1.48	1.45	Y
scl020719.1_146-S	Serpinb6a	NM_009254.2	Mus musculus serine (or cysteine) proteinase inhibitor, clade B, member 6a (Serpinb6a), mRNA.	-1.44	1.17	Y
scl36472.2.242_20-S	Gpx1	NM_008160.1	Mus musculus glutathione peroxidase 1 (Gpx1), mRNA.	-1.36	1.24	Y
scl0012540.1_20-S	Cdc42	NM_009861.1	Mus musculus cell division cycle 42 homolog (S. cerevisiae) (Cdc42), mRNA.	-1.30	1.20	Y
scl0004022.1_70-S	Arpc1b	NM_023142.1	Mus musculus actin related protein 2/3 complex, subunit 1B (Arpc1b), mRNA.	-1.26	1.44	Y
scl0319186.1_75-S	Hist1h2bm	NM_178200	Mus musculus histone 1, H2bm (Hist1h2bm), mRNA.	-1.22	1.23	Y
scl000941.1_0-S	Ndufs2	NM_153064.3	Mus musculus NADH dehydrogenase (ubiquinone) Fe-S protein 2 (Ndufs2), mRNA.	-1.20	1.17	Y
scl019989.6_6-S	Rpl7	NM_011291	Mus musculus ribosomal protein L7 (Rpl7), mRNA.	-1.19	1.23	Y
scl54154.5.1_20-S	Idh3g	NM_008323.1	Mus musculus isocitrate dehydrogenase 3 (NAD+), gamma (Idh3g), mRNA.	-1.15	1.32	Y
scl53358.2.1_26-S	Ms4a6c	NM_028595	Mus musculus membrane-spanning 4-domains, subfamily A, member 6C (Ms4a6c), mRNA.	2.17	1.60	N
scl27109.17.1_35-S	Plod3	NM_011962.2	Mus musculus procollagen-lysine, 2-oxoglutarate 5-dioxygenase 3 (Plod3), mRNA.	1.76	1.18	N
scl020393.12_71-S	Sgk	NM_011361.1	Mus musculus serum/glucocorticoid regulated kinase (Sgk), mRNA.	1.61	1.35	N
scl44868.8_25-S	Gcnt2	NM_008105.2	Mus musculus glucosaminyl (N-acetyl) transferase 2, I-branching enzyme (Gcnt2), transcript variant 3, mRNA.	1.59	1.38	N
scl48644.15_23-S	C330012H03Rik	NM_183029.1	Mus musculus RIKEN cDNA C330012H03 gene (C330012H03Rik), mRNA.	1.56	1.42	N
scl55015.6.1_13-S	Timp1	NM_011593	Mus musculus tissue inhibitor of metalloproteinase 1 (Timp1), mRNA.	1.54	2.01	N

scl066576.4_1-S	Uqcrh	NM_025641.2		1.53	1.20	N
scl20807.21.1_18-S	Dncic2	NM_010064	Mus musculus dynein, cytoplasmic, intermediate chain 2 (Dncic2), mRNA.	1.53	1.26	N
scl018139.26_3-S	Zfml	NM_008717.1		1.52	1.27	N
scl23237.22_584-S	3110057O12Rik	NM_026622.1	Mus musculus RIKEN cDNA 3110057O12 gene (3110057O12Rik), mRNA.	1.52	1.35	N
scl47073.5.231_36-S	Ly6c	NM_010741	Mus musculus lymphocyte antigen 6 complex, locus C (Ly6c), mRNA.	1.45	1.41	N
scl0229543.1_170-S	C77668	NM_145540.2	Mus musculus expressed sequence C77668 (C77668), transcript variant 1, mRNA.	1.42	1.49	N
scl28291.1_56-S	Tctex1	NM_009342	Mus musculus t-complex testis expressed 1 (Tctex1), mRNA.	1.38	1.24	N
scl34564.9_192-S	4930527D15Rik	NM_026350.1	Mus musculus RIKEN cDNA 4930527D15 gene (4930527D15Rik), mRNA.	1.38	1.28	N
scl0319160.1_25-S	Hist1h4k	NM_178211.1	Mus musculus histone 1, H4k (Hist1h4k), mRNA.	1.37	1.31	N
scl34621.8_577-S	Ednra	NM_010332.1	Mus musculus endothelin receptor type A (Ednra), mRNA.	1.34	1.29	N
scl067267.4_0-S	2900010M23Rik	NM_026063.1	Mus musculus RIKEN cDNA 2900010M23 gene (2900010M23Rik), mRNA.	1.29	1.16	N
scl43495.3.1_6-S	2310016C16Rik	NM_027127.1	Mus musculus RIKEN cDNA 2310016C16 gene (2310016C16Rik), mRNA.	1.26	1.20	N
scl54930.9.1_1-S	Hprt	NM_013556		1.23	1.20	N
scl021937.10_167-S	Tnfrsf1a	NM_011609.2	Mus musculus tumor necrosis factor receptor superfamily, member 1a (Tnfrsf1a), mRNA.	1.18	1.38	N
scl30345.6_391-S	Kcnd2	XM_132981.1	Mus musculus potassium voltage-gated channel, Shal-related family, member 2 (Kcnd2), mRNA.	-1.70	-1.47	N
scl34280.9.1_28-S	2310061C15Rik	NM_026844.2		-1.48	-1.24	N
scl39616.9.169_52-S	Nr1d1	NM_145434.1	Mus musculus nuclear receptor subfamily 1, group D, member 1 (Nr1d1), mRNA.	-1.34	-1.58	N
scl34581.9.1_48-S	Gpsn2	NM_134118.1	Mus musculus glycoprotein, synaptic 2 (Gpsn2), mRNA.	-1.32	-1.25	N
scl067956.13_1-S	2410195B05Rik	NM_030241.2	Mus musculus RIKEN cDNA 2410195B05 gene (2410195B05Rik), mRNA.	-1.32	-1.28	N
scl020382.2_66-S	Sfrs2	NM_011358.1	Mus musculus splicing factor, arginine/serine-rich 2 (SC-35) (Sfrs2), mRNA.	-1.26	-1.19	N
scl37421.7_120-S	Rassf3	NM_138956	Mus musculus Ras association (RalGDS/AF-6) domain family 3 (Rassf3), mRNA.	-1.20	-1.27	N

Gene Set	Number of Genes
Unchanged	27
Changed	38
Total	65
**% Changed**	**58.46**
